# Reduction of Tooth Replacement Disproportionately Affects the Evolution of Enamel Matrix Proteins

**DOI:** 10.1007/s00239-025-10258-4

**Published:** 2025-08-07

**Authors:** John Abramyan, Gengxin Li, Hannah Khansa

**Affiliations:** 1 Department of Natural Sciences, University of Michigan–Dearborn, 4901 Evergreen Rd., Dearborn, MI 48128 USA; 2 Department of Mathematics and Statistics, University of Michigan-Dearborn, 4901 Evergreen Rd., Dearborn, MI 48128 USA

**Keywords:** Enamel matrix proteins, Acrodont, Pleurodont, Chameleon, Agamidae, Tooth replacement

## Abstract

In most vertebrates, teeth are continuously shed and replaced throughout life, while mammals and several lineages of reptiles have reduced replacement to only one or two generations. In contrast to the vast majority of their living relatives, members of the lizard families Chamaeleonidae and Agamidae have dispensed with lifelong tooth replacement, instead developing acrodont dentition that fuses to the jawbone to be used for the lifetime of the animal. Though, the loss of tooth replacement has not come without a cost. In order to mitigate the consequences that come with tooth replacement loss, mammals and acrodont lizards have evolved adaptations that strengthen enamel structure and minimize wear and tear experienced during the life of the animal. While these physical adaptations are well documented, the effect that loss of tooth replacement has had on the molecular components of teeth has not received significant attention. Here, we analyze the coding and amino acid sequences of six tooth proteins (AMBN, AMEL, AMTN, ACP4, ENAM, and MMP20) from acrodont lizards, pleurodont lizards that replace teeth, and mammals. We show that the reduction of tooth generations has disproportionately affected the evolutionary trajectory of proteins associated with enamel structure, with a particularly magnified effect on the evolution of AMEL.

**Supplementary Information:**

The online version contains supplementary material available at 10.1007/s00239-025-10258-4.

## Introduction

Vertebrate teeth are used in various capacities ranging from food capture and processing to defense and environmental manipulation; representing a critical component of their functional ecology (Gorman and Hulsey 2020). To maintain teeth in working order and to mitigate wear, most vertebrates rely on polyphyodonty, or continuous replacement of the teeth throughout the lifetime of the animal (Jernvall and Thesleff 2012; Tucker and Fraser 2014). This strategy has been maintained across the majority of extant, dentulous fishes, amphibians, and reptiles.

Despite the importance of teeth, many taxa have reduced the number of tooth replacements or even dispensed with teeth entirely. For example, many mammals with specialized diets (e.g., anteaters), as well as all “true” toads in the family Bufonidae, birds, turtles (Davit-Béal et al. 2009), and a large number of frog lineages (Paluh et al. 2021), no longer produce teeth despite evolving from a toothed ancestor. Meanwhile, the vast majority of toothed mammals only produce two generations of teeth (diphyodont), while rodents and shrews have reduced dentition to just one generation (monophyodont) (Järvinen et al. 2009; Jernvall and Thesleff 2012). Following a similar trajectory, reduction of tooth replacement has occurred in several lepidosaurian reptile families, including in Sphenodontidae (tuatara), Chamaeleonidae (chameleons), and Agamidae (dragon lizards—hereafter referred to as agamids or agamid lizards), with the latter two being sister families within the subclade Acrodonta (Pyron et al. 2013; Reeder et al. 2015), having shared a most recent common ancestor ~ 100MYA (Townsend et al. 2011; Zheng and Wiens 2016).

Members of Acrodonta exhibit direct ankylosis or fusion of teeth to the apical edge of the jawbone by mineralized tissue, resulting in “acrodont” tooth attachment that does not utilize a tooth socket (Edmund 1969; Cooper and Poole 1973; Herrel and Holanova 2008; Buchtová et al. 2013; LeBlanc et al. 2021). The fusion of teeth to the jawbone is thought to inhibit the continuous replacement of teeth. In some instances, adult teeth will even fuse to each other, leading to the formation of a sharpened dental ridge as teeth are worn down, functionally similar to the chelonian beak (Cooper and Poole 1973; Buchtová et al. 2013). Acrodont dentition is one of three recognized modes of tooth attachment in tetrapods, with the other two being pleurodont dentition, where teeth are attached to the lingual surface of the labial wall of the jaw (found in most squamates), and thecodont dentition, where teeth are implanted into sockets (found in crocodilians and mammals) (LeBlanc et al. 2021). Acrodont lizards fall within the infraorder Iguania, which consists of two monophyletic groups named after their respective tooth attachment modes: Acrodonta (Chamaeleonidae and Agamidae), which are considered monophyodont and Pleurodonta, which are polyphyodont (Reeder et al. 2015). This group is an excellent system for comparative analysis of the evolutionary trajectory of orthologous genes and proteins in physiologically different environments.

The reduction of tooth generations obviously poses some challenges, since a single generation of teeth is now expected to last for the lifetime of the animal. To mitigate this predicament, animals have evolved a number of adaptations in order to strengthen their teeth. Mammals have evolved “prismatic” enamel, where hydroxyapatite crystallites bundle in an organized pattern (Grime et al. 1979; Diekwisch et al. 2009; Line and Novaes 2017), and offer the enamel layer of the tooth a greater mechanical advantage under normal masticatory stresses over the aprismatic enamel found in reptiles, amphibians, and fishes (Carlson 1989). It is perhaps also noteworthy that the only case of prismatic enamel found outside of Mammalia occurs in the genus *Uromastyx*, a member of the Agamidae family that has also reduced tooth replacement (Cooper and Poole 1973). In order to further minimize tooth wear, a thickening of the enamel layer has occurred in mammals (Dauphin and Williams 2008; Lucas et al. 2008; Kieser et al. 2009) as well as agamid lizards (*Pogona vitticeps*—Haridy 2018 and *Uromastyx*—Cooper and Poole 1973; Throckmorton 1979). Some dental modifications are only found in acrodont lizards. Both chameleons and agamids exhibit infilling of the pulp cavity with mineralized tissue, thought to prevent pulp cavity exposure as the external surface of the tooth is worn away over the life of the animal (Throckmorton 1979; Dosedělová et al. 2016; Haridy 2018). Overall, these modifications in acrodont lizards and mammals show that the loss of tooth replacement has come at a cost that needed to be mitigated, and in some cases, mammals and reptiles have converged upon similar solutions.

Considering the aforementioned modifications of teeth in acrodont lizards and mammals, we speculated that reduction of tooth replacement would similarly affect the molecular evolution of developing teeth. Fortunately, teeth are not only unique in their development and structure but are also associated with proteins that are specific to their development. While it cannot be ignored that studies have detected these “tooth-specific” proteins in other parts of the body (Fong and Hammarström 2000; Janones et al. 2005; Deutsch et al. 2006; Spahr et al. 2006; Haze et al. 2007; Spoutil et al. 2023), it is also true that their roles in non-dental tissues must not be significant since pseudogenization of their encoding genes readily occurs in every tetrapod lineage where teeth are lost, ranging from mammals to birds, turtles, and true toads (Meredith et al. , 2009, 2011; Shaffer et al. 2013; Mu et al. 2021; Shaheen et al. 2021).

It is well known that the major structural proteins such as amelogenin (AMEL, AMELX), enamelin (ENAM), ameloblastin (AMBN), and amelotin (AMTN), as well as a number of proteinases (e.g., matrix metalloproteinase-20 [MMP20], acid phosphatase 4 [ACPT4]) are essential for the formation of enamel and are all directly or indirectly involved in controlling mineral assembly in developing tooth enamel (Lacruz et al. 2017). We chose the aforementioned proteins, and genes that encode them, because they are strongly expressed during amelogenesis, have been shown to pseudogenize in edentulous species (thereby demonstrating their primary role in tooth formation), and are found in both mammals and reptiles. AMEL, AMBN, and ENAM comprise the enamel matrix proteins (EMPs), known to make up the bulk of the secreted enamel organic matrix (Smith et al. 2017). Of the EMPs secreted by ameloblasts, 90% consists of AMEL, with the other 10% consisting of AMBN and ENAM (Termine et al. 1980). Of the non-EMP proteins, AMTN is thought to mediate attachment between the mature ameloblasts and the mineralizing enamel (Holcroft and Ganss 2011; Moffatt et al. 2014; Bartlett and Simmer 2015), as well as the attachment of the gingiva to the developing tooth (Bosshardt and Lang 2005; Moffatt et al. 2006). Additionally, Abbarin et al. (2015) have found evidence that AMTN is capable of inducing the mineralization of hydroxyapatite, the main mineral component of enamel. MMP20 and ACP4 are enzymes involved in the cleavage and processing of EMPs after secretion by ameloblasts (Simmer and Hu 2002; Liang et al. 2022). Several other tooth-specific proteins were considered for this study but have not been identified in reptile genomes thus far, including ODAM (Kawasaki and Amemiya 2014) and KLK4 (Kawasaki et al. 2014).

The aims of the present study are (I) to better understand how the reduction of tooth generations affected the evolutionary trajectory of tooth development proteins in reptiles, and (II) whether the evolutionary trajectory of these proteins in acrodont lizards converges with mammals. Furthermore, by analyzing proteins that are directly involved in enamel structure (e.g., EMPs) as well as those that are more peripherally associated with tooth development, we predict a discernable difference in the evolutionary trajectory of EMPs in lineages that exhibit a reduction of tooth replacement, akin to physical modifications of tooth and enamel structure in these groups. Through a series of computational analyses, we show here that pleurodont and acrodont lizards indeed show differences in the selection pressure across EMPs as well as AMTN. Moreover, by analyzing chameleons and agamids as separate subclades within the family Acrodonta, we uncovered previously unrecognized differences in EMP evolution and functional divergence even within lineages that do not replace teeth. Lastly, we show that a significant proportion of convergence in the molecular evolution of tooth proteins between mammals and acrodont lizards lies in the EMPs but not in the associated tooth development enzymes. Consequently, we conclude that the loss of tooth replacement places a disproportionate evolutionary pressure on EMPs, with the evolutionary trajectory of AMEL being particularly affected.

## Materials and Methods

### Sequence Acquisition and Multiple Sequence Alignment

We analyzed lizard species from the infraorder Iguania (n = 41; 24 species from 12 genera representing Acrodonta and 17 species from 9 genera representing Pleurodonta). Mammal species (n = 12) were chosen based on having an omnivorous diet, in order to avoid species that may have specialized diet or ecological niche that could induce specialization of their dentition (see supplementary information for full species list for mammals and reptiles).

Reptile genomes were searched in GenBank using the BLAST feature in the web interface for each genome. To conserve current nomenclature, we followed previously published exon numbering system of reptile EMPs established by Sire and colleagues: AMEL (Sire et al. 2005; Gasse and Sire 2015); AMBN (Gasse and Sire 2015); ENAM (Al-Hashimi et al. 2010). Exons not found by BLAST were queried within the scaffolds/contigs containing identified exons with the “Map to Reference” tool in Geneious v9.1.6 (Biomatters, Auckland, New Zealand—Kearse et al. 2012). Exons were concatenated and batch translated at: http://www.bioinformatics.org/sms2/translate.html. Multiple sequence alignments of amino acid sequences were generated using MAFFTv.7 (Multiple Alignment using Fast Fourier Transform) (https://mafft.cbrc.jp/alignment/software/; https://mafft.cbrc.jp/alignment/server/; Katoh et al. 2002), one of the most broadly used and highest ranked (in terms of accuracy, speed, and consistency) sequence alignment programs developed to date (Durand et al. 2010; Chang et al. 2014; Bawono et al. 2017). DNA coding sequence alignments were subsequently generated by converting amino acid alignments to DNA sequence alignments using the PAL2NAL tool (Suyama et al. 2006; http://www.bork.embl.de/pal2nal/).

### Percentage Identity Calculations

We performed percentage identity analysis in order to better understand the relationships between the various orthologs we are working with in this project as well as putative functional changes that may have taken place. Percentage identity values for amino acid sequences were calculated from MAFFT alignments (as described above) in the Geneious v9.1.6 software package. Amino acid sequences were utilized for percentage identity analyses in order to obtain a more accurate estimate of the changes in the functional units of proteins, and to avoid inflation of identity differences due to synonymous mutations. This and all other figures were compiled in Adobe Photoshop CS6 and Adobe Illustrator CS6 (Adobe Systems inc., San Jose, CA).

### Selection Pressure Analysis

One of our main goals was to assess differences in natural selection pressure acting on each gene between various clades in our study. For this aim, we obtained estimates of the ratio of non-synonymous to synonymous substitutions (dN/dS or ω) using the codeml program in PAML v4.8 (Yang 2007). The ω value measures selection pressure on coding nucleotide sequences. An ω estimate < 1 indicates negative or purifying selection (dN < dS), ω ≈ 1 indicates neutral selection (dS ≈ dN), and ω > 1 is considered to infer positive selection (dN > dS). To estimate ω, a tree‐based likelihood approach was implemented as described by Yang et al. (1998). A series of nested, branch‐specific codon model analyses were used to estimate selection along specific groups of branches in a species tree. MAFFT codon alignments were used for all analyses. The free‐ratio model is the most general, parameter‐rich model and allows for different ω values for each branch. The one‐ratio model is the simplest and assumes the same ω for all branches, while the two‐ratios, three‐ratios, and four‐ratios models allow for the estimation of two, three, and four ω values, respectively, within a tree. Likelihood estimates are calculated from the codon substitution model of Goldman and Yang (1994). The likelihood estimates for each run under a different model were compared using a hierarchical likelihood ratio test (LRT), which doubled the difference of two log likelihoods corresponding to two models (2Δl = 2(l_1_ − l_0_)), with the result approximating a chi‐square (χ^2^) distribution (Yang et al. 1998).

Since PAML is sensitive to gaps in sequence, Pal2Nal alignments were generated, sites with more than 50% gaps were removed and the resulting sequences were used for PAML analyses. Coding sequences for each gene were used to generate a gene tree under a GTR model using PhyML in Geneious v9.1.6. Following Álvarez-Carretero et al. (2023), an unrooted tree was used for the one-ratio model (mammals, acrodonts, pleurodonts forming a polytomy), with a rooted tree used for free-ratio as well as the two-ratio, three-ratio, and four-ratio models.

### Functional Divergence Analysis

Functional divergence analysis was performed because between-ortholog percentage identity values are low and we desired to understand if this correlated with functional change. For this, we performed functional divergence analysis of proteins using the DIVERGE 3.0beta software (Gu 1999; Gu et al. 2013). DIVERGE uses a phylogenetic tree to assess site‐specific changes in evolutionary rates between user‐defined, monophyletic subclades after a divergence event (e.g., gene duplication/speciation) to identify amino acid residues with predicted functional divergence. For DIVERGE analysis, we generated a gene tree using amino acid sequences under a GTR model using PhyML in Geneious v9.1.6. DIVERGE recognizes 2 types of divergence as described by Gu (2001). Type-I functional divergence results in a site-specific shift in evolutionary rate (Gu 1999). A typical pattern this condition manifests is high conservation of an amino acid residue in one subclade, while another evolves freely in this position, resulting in a high variability (Gu 1999; Gu et al. 2013). Type-II functional divergence does not result in a site-specific change in rate of evolution, but rather a radical change in amino acid physicochemical properties (e.g., charge, hydrophobicity, etc.) between subclades, at a particular position (Gu 2001, 2006). This condition manifests a scenario where amino acid positions show clade‐specific conservation (largely complete fixation within each), albeit different amino acids are fixed in each of the two clades, resulting in “conserved‐but‐different” residues (Gu 1999, 2006).

DIVERGE then calculates Gu’s coefficient of evolutionary functional divergence (*θ*) which ranges between 0 and 1, and measures changes in site‐specific evolutionary rates. *θ* = 0 indicates no functional divergence, with an increase in *θ* value as functional divergence increases (Gu 1999, 2001). *θ* can be interpreted as the decrease in the correlation of evolutionary rate between subclades as a result of functional divergence (Gu 1999). To calculate *θ*, DIVERGE applies a model-free method as well as a maximum likelihood method (Gu 1999). Under the model-free method the *θ* estimate is susceptible to large sampling variance due to small sample size (Gu 1999). Therefore, we used the ThetaML value in this study. According to Gu (1999), ML estimates are also slightly smaller than those of the model-free estimate, therefore also being more conservative.

In order to assess statistical significance of Type-I divergence, we used the ThetaML estimate with corresponding LRT values in the DIVERGE output; with values approximately following a chi-square distribution with 1 degree of freedom (as per the DIVERGE manual version 3.0). The statistical significance of Type-II divergence is based on the estimated *θ* and its standard error, with p-values obtained from a Z-score test (DIVERGE manual version 3.0). Along with estimating *θ*, DIVERGE also provides position‐specific posterior probability (pp) values, which can be used to predict the amino acid sites critical for divergence. Empirical cutoff for significance of pp values are established by sequentially removing the highest scoring residues from the alignment until ThetaML (for Type-I) and Theta‐II (for Type-II) are no longer significantly different from 0.

Analyses comparing mammals with lizards included alignments with our entire dataset of mammals, pleurodonts lizards, and acrodont lizards. However, this multiple sequence alignment possessed numerous gaps and since DIVERGE removes all sites with gaps, the resulting analyzed dataset was limited. Therefore, lizard-only analyses were also performed (pleurodonts vs. acrodonts; chameleons vs. agamids) to increase the number of sites under analysis since a lizard-only MAFFT alignment results in fewer gaps.

### Functional Distance Analyses

A limitation of the two-cluster analysis used for calculating functional divergence is that it cannot tell us whether an ortholog from one group exhibits a larger shift in evolutionary rate than the other. This issue is addressed by a method described by Wang and Gu (2001) that can be applied when more than two homologous gene or lineage clusters are available. The functional branch length (b_F_) indicates whether a gene in one group has retained a function or accumulated significant differences and neofunctionalization relative to its ancestral copy. In other words, if *b*_F_ ~ 0, it indicates that the evolutionary rate of each site in the diverged clade has remained nearly the same since the gene duplication or speciation event, indicating that the “derived” state is more similar to the ancestral state for this particular cluster.

As described by Wang and Gu (2001) and Gu et al. (2002), the estimated coefficients of type I functional divergence (*θ*) for all the pairs of gene or species clusters are used to create a matrix of d_F_ values. DIVERGE 3.0beta uses the “MFE Theta” value to perform this calculation. First, the functional distance (d_F_) between any two clusters is defined as d_F_ = −ln (l − Θ). Under the assumption of independence, Wang and Gu (2001) show that d_F_ is additive, that is, for clusters A and B, d_F_ (A, B) = *b*_F_ *(A)* + *b*_F_ *(B),* where *b*_F_ *(x)* is the functional branch length of a cluster *x.* Larger *b*_F_ value for a gene/species cluster indicates the evolutionary conservation may be changed at many sites. Given the d_F_ values, a standard least squares method can be implemented based on the formula d_F_ (A, B) = *b*_F_ *(A)* + *b*_F_ *(B)* to estimate *b*_F_ for each gene/species cluster. Since we use the Gu99 ThetaML estimates for this study, we decided to calculate the *b*_F_ values ourselves with the ThetaML values estimated in DIVERGE v.3.0.beta using the formula below. (See supplementary information for least squares method calculation).

Here, we introduce a simpler method of calculating *b*_F_ values between three groups, using a linear algebra calculation as opposed to the least squares method. We first calculate d_F_ values for each pair using the equation from Wang and Gu (2001): d_F_ = −ln (l − Θ). We then use a series of simple calculations to obtain the *b*_F_ value for each group (A, B, C).

$${b}_{F}\left(A\right)=\frac{{d}_{F}\left(A,B\right)+{d}_{F}\left(A,C\right)-{d}_{F}\left(B,C\right)}{2}$$

$${b}_{F}\left(B\right)=\frac{{d}_{F}\left(A,B\right)+{d}_{F}\left(B,C\right)-{d}_{F}\left(A,C\right)}{2}$$

$${b}_{F}\left(C\right)=\frac{{d}_{F}\left(A,C\right)+{d}_{F}\left(B,C\right)-{d}_{F}\left(A,B\right)}{2}$$

These formulas were tested against the output from the “Functional Distance” tab in DIVERGE 3.0 beta (which uses MFE Theta values) and obtained the same results.

### Convergence Analysis

We also wanted to test for convergence between acrodont lizards and mammals, since they both either reduced or lost tooth replacement. To detect convergent amino acid changes between these groups, we used the Profile Change with One Change (PCOC) method, which has been shown to outperform other methods of convergence analysis (Rey et al. 2018). PCOC identifies convergent shifts in amino acid “profiles” at a given site rather than change to identical amino acids, which may be too strict since several amino acids have similar physicochemical properties. Biochemical properties of amino acids are modeled as “profiles” for each position and each branch, using a vector of amino acid frequencies built from large empirical datasets. A site is characterized as being convergent when it exhibits a shift from an ancestral biochemical role to a new biochemical role that is shared by more than one convergent lineage. If a site belongs to the same profile in convergent branches and a different profile in the background, it is considered to show a profile change (PC). Additionally, the convergent site must display at least one substitution, or one change (OC), in each convergent branch. The combination of two models provides a single estimate of posterior probability of PCOC convergence. Provided with a MAFFT amino acid multiple sequence alignment, a phylogenetic tree matching the species in the alignment (generated using amino acid sequences under a GTR model using PhyML in Geneious v9.1.6.) and identification of putatively convergent clades (acrodont lizards and mammals), PCOC performs an analysis for its two models (PC and OC) for which a posterior probability threshold of 0.8 was set, ignoring gaps and ambiguities.

### Substitution Rate Analysis

Lastly, while we performed various analyses based on the differences in the sequences, we desired to test the rate at which these differences arise in different clades. For this we used baseml (implemented in PAML; Yang 2007) to estimate nucleotide substitution rates under a one-rate model (global clock), as well as three- and four-rates models (local clock). Analysis was performed according to Lemey and Posada (2009). For all baseml runs, we used a GTR (model option 7:REV in baseml) substitution model, with the same gene and species topologies used for codeml analyses. Divergence times were derived from the 52 genes and 4162 species study of Zheng and Wiens (2016). The three- and four-rates models were tested against the one-rate model using likelihood ratio tests.

## Results

### Divergence in Sequence Identity Between Acrodonta and Pleurodonta EMPs

**Fig. 1:**
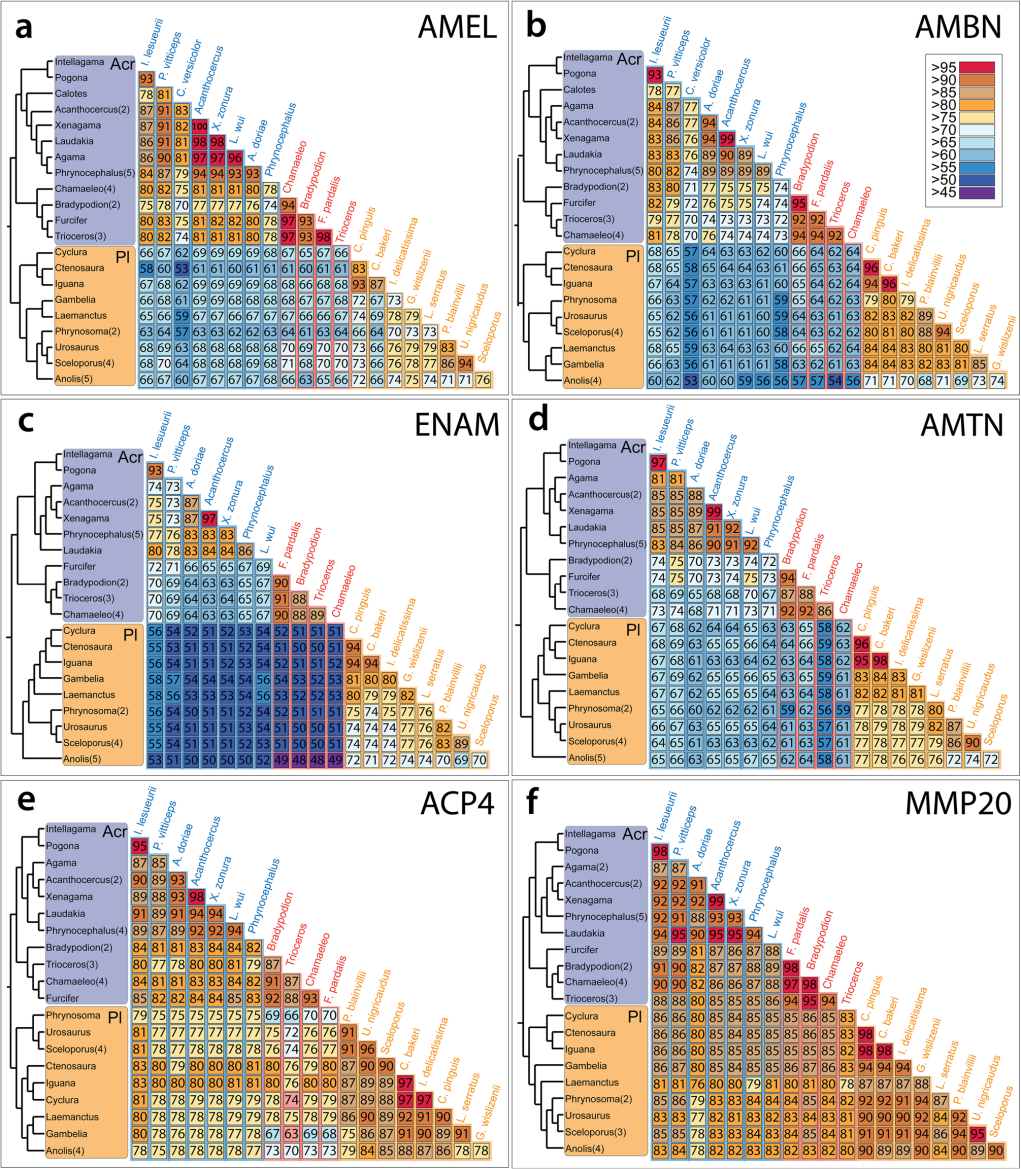
Amino acid sequence identity for (**a**) amelogenin (AMEL); (**b**) ameloblastin (AMBN); (**c**) enamelin (ENAM); (**d**) amelotin (AMTN); (**e**) acid phosphatase 4 (ACPT4); (**f**) matrix metalloproteinase-20 (MMP20). Percentage identity calculations with MAFFT alignment of full‐length amino acid sequences. Genera with a number next to the name represent an average percent identity of that number of species. Acr, Acrodonta; Pl, Pleurodonta

In order to obtain a cursory view of the proteins sequences in the study and the potential for functional differences to have arisen, we first performed a percentage identity analysis using a lizard-only dataset. An overall picture emerged of an obvious difference between pleurodonts and acrodonts within the four structural genes (AMEL, AMBN, ENAM, and AMBN), whereas ACP4 and MMP20 showed either a weak signal or none at all (Fig. 1). Moreover, while AMEL, AMBN, and AMTN exhibited identity values in the 60 percent range, ENAM identity between pleurodonts and acrodonts was lower—in the 50 range, with chameleons exhibiting values in the high 40s. According to the literature, 40–50% identity appears to be a significant boundary for functional divergence between genes (Sangar et al. 2007; Fiser 2017).

### Purifying Selection in Acrodonta EMPs

**Fig. 2:**
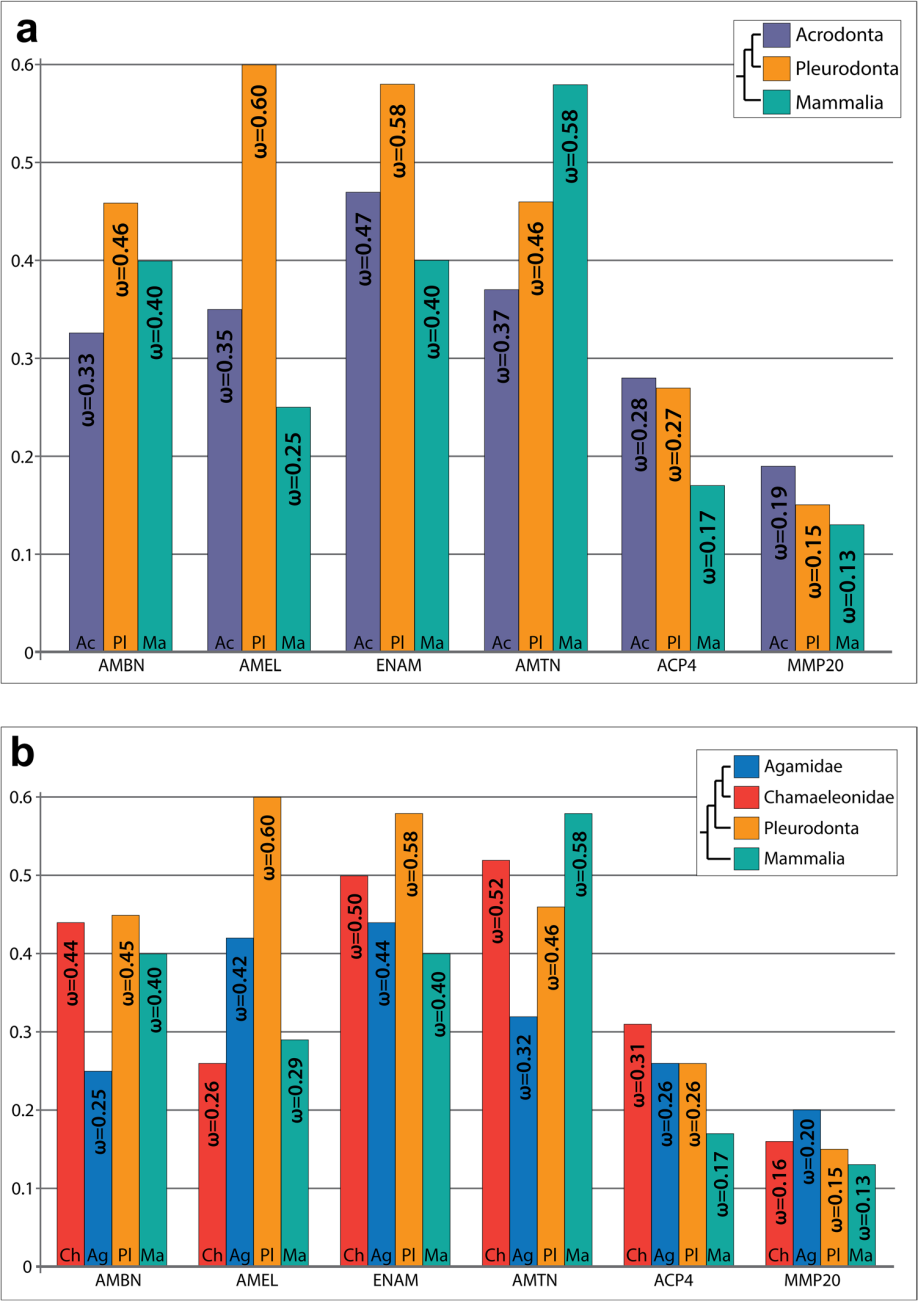
Branch-specific ω estimates. (**a**) Estimated ω ratios from three-ratios analysis: labeling Acrodonta (Ac), Pleurodonta (Pl), and Mammalia (Ma). (**b**) Estimated ω ratios from four-ratios analysis: labeling chameleons (Ch), agamids (Ag), pleurodonts (Pl), and mammals (Ma)

Selection analysis was performed across all 6 enamel genes, comparing acrodonts, pleurodonts, and mammals. Within lizards, we found that acrodonts exhibited consistently lower ω values than pleurodonts for all EMPs as well as AMTN (AMBN – ω_a_ = 0.33 vs. ω_p_ = 0.46; AMEL – ω_a_ = 0.35 vs. ω_p_ = 0.60; ENAM – ω_a_ = 0.47 vs. ω_p_ = 0.58; AMTN – ω_a_ = 0.37 vs. ω_p_ = 0.46). ACP4 (ω_a_ = 0.28 vs. ω_p_ = 0.27) and MMP20 (ω_a_ = 0.19 vs. ω_p_ = 0.15) failed to show this pattern (Fig. 2a and Table S1). Here, AMEL stood out as having the largest disparity in ω estimate between acrodonts and pleurodonts, with acrodonts showing much stronger purifying selection, while pleurodonts exhibited more relaxed selection.

Since mammals also experienced a reduction in tooth generations, we predicted that they would also exhibit a similar, lower ω value, comparable to acrodonts. Indeed within the EMPs, mammal did exhibit consistently lower ω values when compared to pleurodonts, but not always comparable to acrodonts (Fig. 2a). Compared to acrodonts, mammals exhibited a higher ω estimate for AMBN (ω_m_ = 0.40 vs. ω_a_ = 0.33), but lower in AMEL (ω_m_ = 0.25 vs. ω_a_ = 0.35) and ENAM (ω_m_ = 0.40 vs. ω_a_ = 0.47). Interestingly, mammals exhibited the highest AMTN ω estimate compared to both squamate groups. ACP4 and MMP20 showed the lowest ω estimates in mammals (ω = 0.17 and ω = 0.13, respectively), indicating strong purifying selection (Fig. 2a). For AMBN, AMEL, ENAM, AMTN, and ACP4, the three-ratios model (acrodonts-pleurodonts-mammals) fit the data significantly better than a two-ratios model, where only lizards and mammals were labeled. For MMP20, the three-ratios model did not fit the data significantly better than the two-ratio model, which only differentiated between lizards and mammals (Table S1).

We speculated that there might also be a difference between chameleons and agamids, the two clades within Acrodonta. These two groups have historically been considered similar when it comes to their dentition, but recent developmental studies have begun to reveal some physiological differences between their teeth. Therefore, we not only tested models with just two and three ω estimates for the dataset, reptiles-mammals, and acrodonts-pleurodonts-mammals, respectively, but also a model with four ω estimates: chameleons-agamids-pleurodonts-mammals. The model with four ω values fit our datasets significantly better across all EMPs plus AMTN, but not ACP4 and MMP20 (Table S1—model M2c). With the four-ratios model, chameleons exhibited the most stringent purifying selection for AMEL across all groups analyzed (ω_ch_ = 0.26, ω_agam_ = 0.42, ω_p_ = 0.60, ω_m_ = 0.29), whereas for the other genes, chameleons exhibited ω values comparable to if not higher than agamids, pleurodonts, and mammals (Fig. 2b, Table S1.). Conversely, agamids retained ω estimates lower than pleurodonts across all EMPs plus AMTN.

### EMPs Exhibit Functionally Divergence Within Squamates as Well as Acrodonta

To investigate whether there was adaptive functional diversification between the various groups in this study, we used DIVERGE 3.0 to estimate Gu’s coefficient of evolutionary functional divergence (*θ*). Across all proteins analyzed, lizards and mammals exhibited significant Type-I functional divergence: AMBN—*θ* = 0.74 ± 0.10, AMEL—*θ* = 0.49 ± 0.15, AMTN—*θ* = 0.99 ± 0.18, ENAM—*θ* = 0.70 ± 0.06, MMP20—*θ* = 0.30 ± 0.06, ACP4—*θ* = 0.36 ± 0.07, indicating a significant amount of site-specific change in evolutionary rate between the groups (Table 1), with the EMPs and AMTN generating particularly high *θ* estimates. Between these groups, AMTN stood out as being particularly different, with a high functional divergence coefficient and generally poor alignment. Assessment of lizards and mammals for Type II divergence showed significant divergence in AMBN, AMTN, and ACP4, while AMEL, ENAM, and MMP20 were not found to be significant (Table S2).

**Table 1:** Type-I functional divergence between enamel proteins calculated in DIVERGE

		**AMBN**	**AMEL**	**ENAM**	**ACP4**	**AMTN**	**MMP20**
**Iguania vs. Mammal**	**MLE *θ* ± S.E**	0.74 ± 0.10	0.49 ± 0.15	0.70 ± 0.06	0.36 ± 0.07	0.99 ± 0.18	0.30 ± 0.06
	***LRT Theta***	103.93	12.32	142.48	27.76	32.36	23.98
	***P-value***	1.06e^−24^	2.40e^−4^	3.48e^−33^	7.11e^−08^	6.61e^−09^	5.06e^−07^
	***pp-cutoff***	0.92	0.84	0.87	0.78	**	0.81
	***# of RFD***	34	3	63	8		6
**Acro. vs. Pleuro**	**MLE *θ* ± S.E**	0.17 ± 0.10	0.80 ± 0.16	0.38 ± 0.08	0.13 ± 0.11	0.41 ± 0.17	0.40 ± 0.10
	***LRT Theta***	3.05	26.66	23.40	1.33	5.39	15.74
	***P-value***	0.05	1.26e^−07^	6.85e^−07^	ns	0.01	3.85e^−05^
	***pp-cutoff***	0.55	0.91	0.67		0.82	0.76
	***# of RFD***	2	9	15		1	6
**Agam. vs. Cham**	**MLE *θ* ± S.E**	0.60 ± 0.17	0.94 ± 0.29	0.46 ± 0.14	− 0.23 ± − 0.23	0.27 ± 0.30	0.37 ± 0.22
	***LRT Theta***	12.08	10.69	11.53	1.04	0.81	2.88
	***P-value***	2.74e^−04^	5.82e^−04^	3.68e^−04^	ns	ns	ns
	***pp-cutoff***	0.77	0.95	0.65			
	***# of RFD***	7	27	7			

*θ*: the coefficients of Type-I functional divergence between two clades (ThetaML estimates); *LRT* Likelihood ratio statistic; pp-cutoff: minimum posterior probability of amino acid sites leading to functional divergence; # of RFD: predicted number of amino acid sites associated with functional divergence; S.E.: standard error; ** All analyzed sites exhibited > 0.99 posterior probability; ns: not significant

In addition to comparison between mammals and reptiles, we were also interested in functional divergence between acrodont and pleurodont lizards as well as between chameleons and agamids, in order to assess whether changes in the tooth replacement regimen within Iguania led to adaptive functional diversification. This series of analyses brought some strong patterns to light. Acrodonts vs. pleurodonts analysis showed significant Type-I functional divergence between all proteins tested except for ACP4 (Table 1), with no Type-II divergence detected (Table S3). Delving deeper into Acrodonta, we found that there was significant functional divergence between agamid and chameleon EMPs, while non-EMP proteins (AMTN, ACP4, MMP20) did not show functional divergence between these groups (Table 1). Moreover, within both lizard-only analyses, AMEL exhibited particularly high *θ* values of 0.80 ± 0.16 and 0.94 ± 0.29, along with the most functionally divergent sites (27), despite being the shortest of the protein in this study (Table 1). Incidentally, AMEL was also the only protein to exhibit significant Type-II divergence between agamids and chameleons (Table S4) (see supplementary information for divergent sites).

### Functional Distance Analysis Reveals a Large Deviation in Mammals, as Well as Chameleon EMPs

**Fig. 3:**
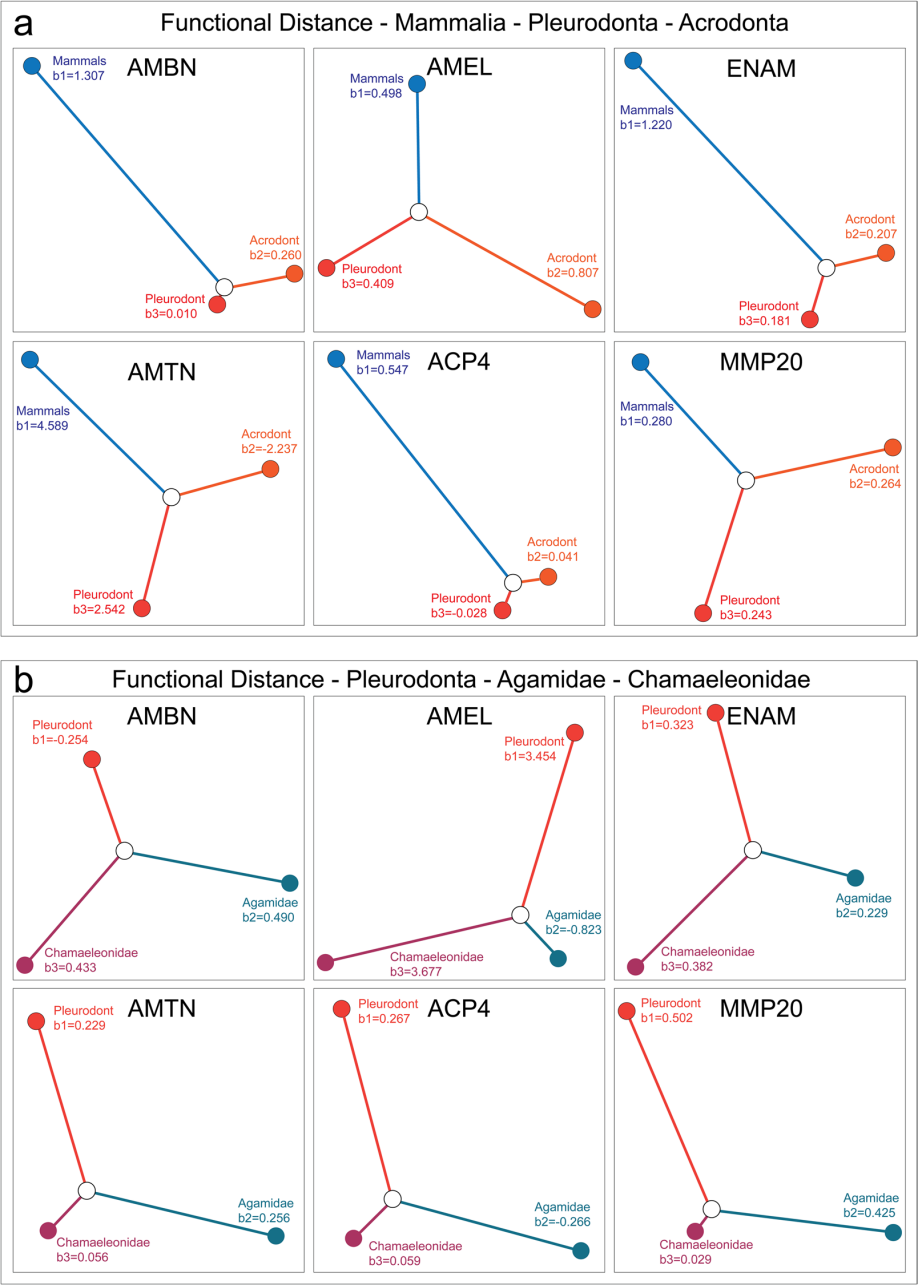
Cluster-based functional distance analysis. The diagrams illustrate the degree of the divergence of each cluster based on site-specific shifts in evolutionary rates after divergence. (**a**) Schematic representation of functional distance between mammals, pleurodonts, and acrodonts from a theoretical inferred ancestor (white circle). (**b**) Functional distance between chameleons, agamids, and pleurodonts from a theoretical inferred ancestor (white circle). Branch lengths are proportional to depicted b-values for all analyses

Delving further into the functional diversification of these proteins, we performed functional distance analysis using the *θ* estimates generated in DIVERGE. This analysis provides information on which clades the divergence took place in and how much change has occurred from the shared common ancestor. Functional distance analysis revealed a landscape where mammals largely exhibited either equal to or greater divergence values compared to the two lizard groups (b_F_ values indicate neofunctionalization compared to the ancestral state in the common ancestor of mammals and reptiles) (Fig. 3a), with more moderate degrees of functional divergence in lizards. However, AMEL was once again an exception to this pattern, where a substantially larger b_F_ value was estimated for acrodont lizards compared to mammals and pleurodonts (Fig. 3a).

When the lizard-only dataset was analyzed, labeling the two acrodont groups as separate clades, a clear demarcation emerged between EMPs and non-EMP proteins. We had previously detected this patterning in our Type-I divergence analysis between Agamidae–Chamaeleonidae, however, it was unknown in which lineage the majority of divergence had taken place. By performing functional distance analysis, chameleons consistently stood out as the more divergent clade when it comes to EMPs, specifically AMEL and ENAM. Chameleons also appeared more conservative than either agamids or pleurodonts for non-EMP proteins—AMTN, ACP4, and MMP20—while agamids showed the opposite trend (Fig. 3b). Pleurodonts showed a generally high degree of functional divergence across the board, with the exception of AMBN.

### EMPs in Acrodont Lizards and Mammal Exhibit Convergence in Amino Acid Sequence

**Fig. 4:**
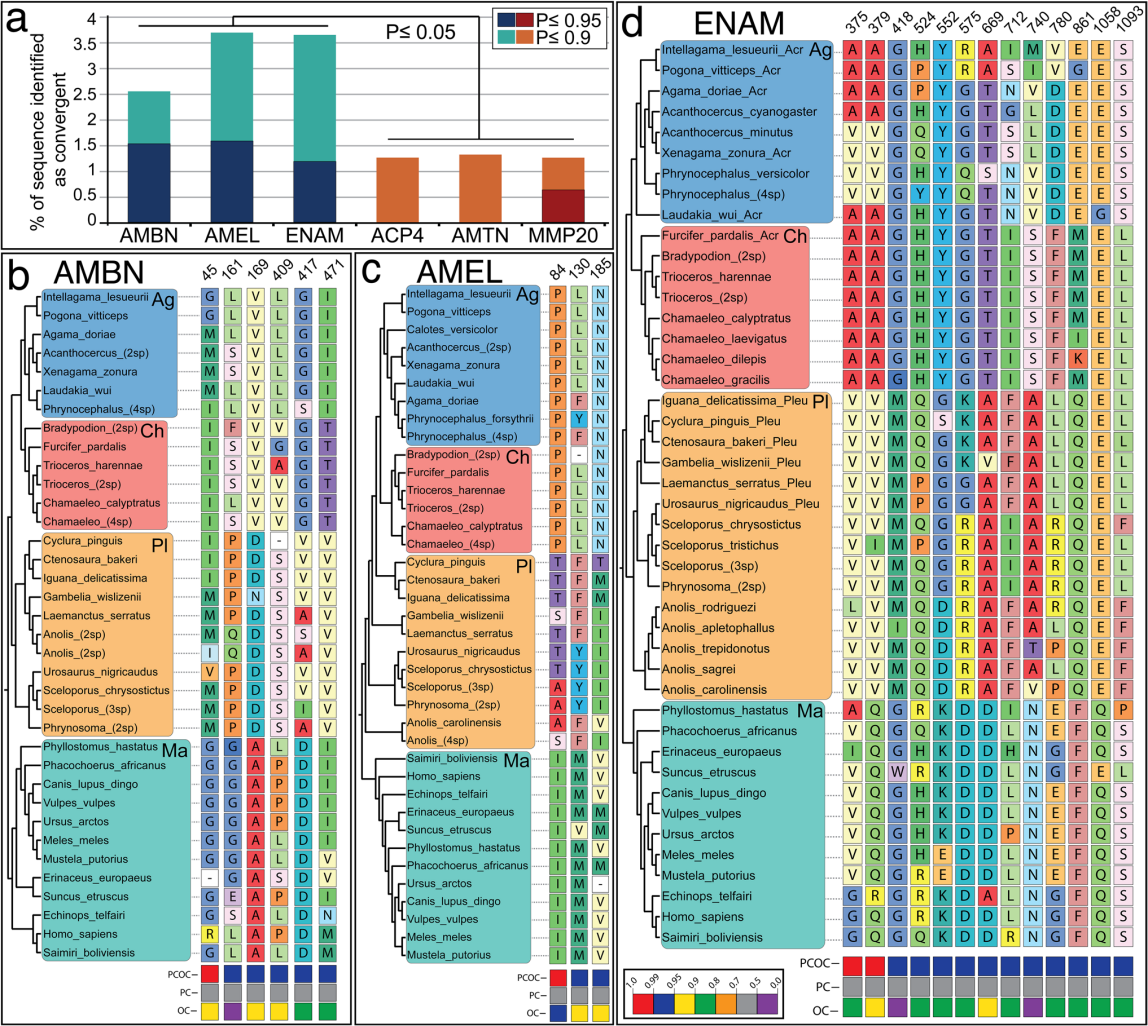
Convergent evolution analysis using the PCOC toolkit. (**a**) Graphical representation of the proportion of amino acid sequence for each protein identified as being convergent with pp cutoffs of ≥ 0.9 and ≥ 0.95 (according to the Profile Change with One Change [PCOC] model) depicted as percentage of the average amino acid sequence length of all sequences used in the analysis. (**b–d**) Sites with PCOC pp values ≥ 0.95 for EMPs. Posterior probabilities for different models are shown in different colors at the bottom of each column. Agamidae (Ag); Chamaeleonidae (Ch), pleurodonts (Pl), and mammals (Ma)

We analyzed molecular convergence between acrodont lizard and mammals using PCOC, which tests whether amino acid substitutions in a priori identified lineages (acrodonts and mammals) converge on amino acids with similar biochemical properties. At a posterior probability (PP) cutoff of 0.95, PCOC only detected convergent shifts in amino acid preferences in the three EMPs and MMP20, whereas at a cutoff of 0.9, all genes exhibited some sites with convergence, albeit to significantly different degrees (Fig. 4a). AMBN exhibited ten residues at a cutoff of pp ≥ 0.9, of which six exhibited pp ≥ 0.95. AMEL exhibited seven residues at a cutoff of pp ≥ 0.9, of which three exhibited pp ≥ 0.95. ENAM exhibited 40 residues at a cutoff of pp ≥ 0.9, of which 13 exhibited pp ≥ 0.95. These values are considerable in comparison to the average lengths of AMBN, AMEL, and ENAM sequences used in the analysis, representing 2.55%, 3.68%, and 3.64%, respectively, of total alignment length for pp ≥ 0.9 cutoff, and 1.53%, 1.58%, and 1.18% for pp ≥ 0.95 cutoff (Fig. 4a). In contrast ACP4, AMTN, and MMP20 exhibited significantly smaller proportions of convergent residues with a total of five, four, and six residues, respectively, representing just 1.26%, 1.32%, and 1.26% at pp ≥ 0.9 cutoff and 0%, 0%, and 0.63% at pp ≥ 0.95 cutoffs, respectively (Fig. 4a). (see supplementary information for all convergent sites).

### AMEL Experiences Reduced Substitution Rates When Tooth Replacement Is Reduced

**Fig. 5:**
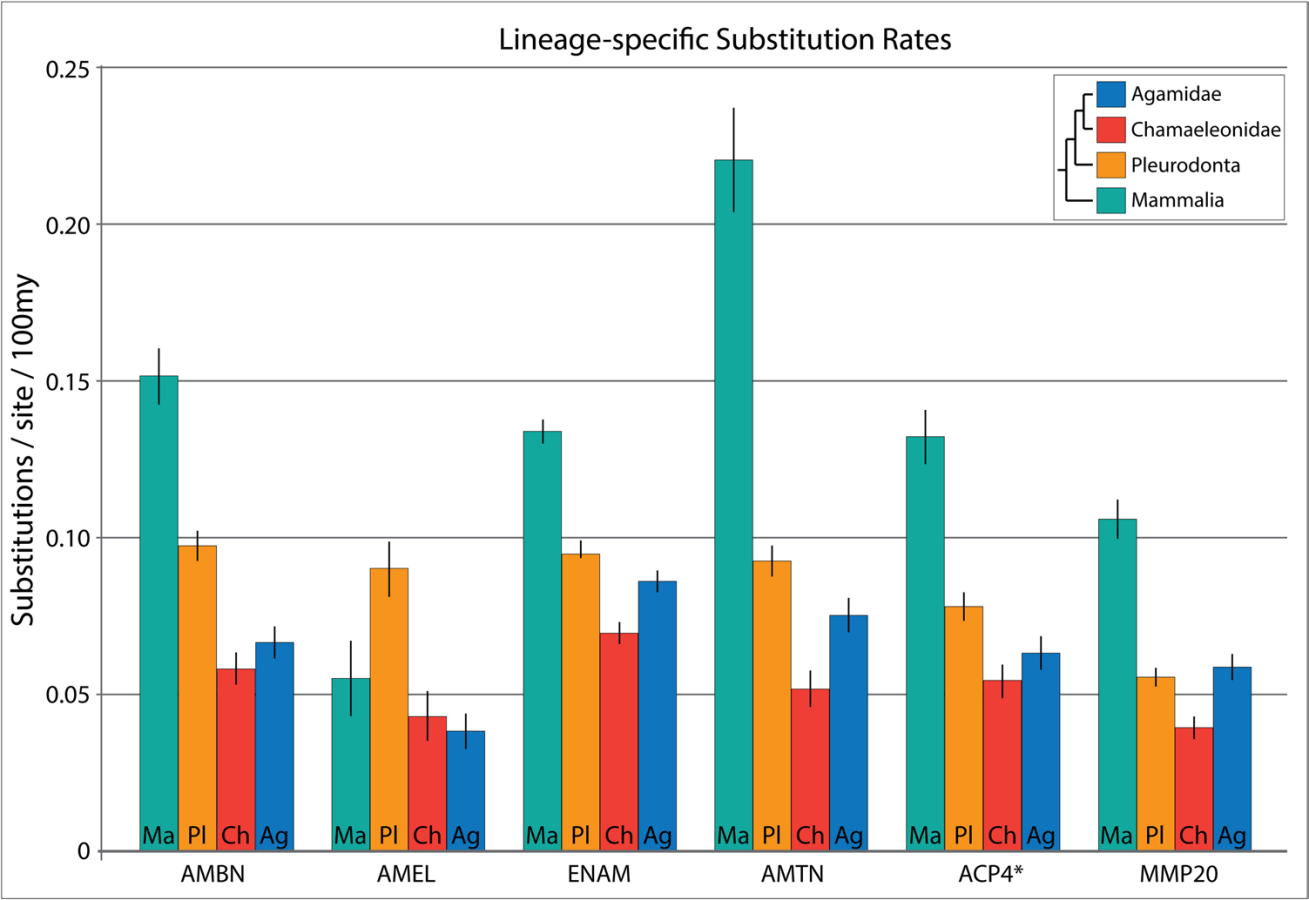
Substitution rate analysis. Estimated substitution rates from four-rates analysis. *ACP4 four-rates model was not significantly different than three-rates model. Error bars indicate standard error

Finally, we wanted to estimate substitution rate for each clade in order to assess whether divergence could be attributed to selection pressure or was simple correlated with the age of each group (e.g., older clades being more divergent). We used baseml to estimate nucleotide substitution rates, which tests nested models similar to the selection analysis in codeml. We tested a global clock model, where one molecular substitution rate was estimated for all branches in the tree against a three-rates model, allowing mammals, pleurodonts, and acrodonts to have individual substitution rates. Across all genes, likelihood ratio test revealed a significantly better fit for the three-rates model. We then compared the three-rates model against a four-rates model, once again splitting Acrodonta into chameleons and agamids. The four-rates model fit the data better for all genes except ACP4 (Table S5) (Fig. 5).

When examining the estimated substitution rates from the four-rates model, some very clear patterns appear. Across all genes except AMEL, mammals exhibit a substantially higher substitution rate when compared to lizards. Pleurodonta also exhibited higher substitution rates than acrodonts in all genes except MMP20. Once again, AMEL stood out as an outlier in this analysis, with pleurodonts exhibiting the highest estimated substitution rate, while mammals and acrodonts exhibit lower rates, comparable to each other.

## Discussion

While tooth loss seems to have occurred repeatedly across tetrapods, the loss of tooth replacement is ostensibly more rare. Moreover, we know that when teeth are lost, pseudogenization of tooth genes readily takes place, but the question of what happens to these genes and proteins when tooth generations are reduced, still remains to be described. Here, we compare monophyodont and polyphyodont lizards with diphyodont mammals, with the aim of understanding how the reduction of tooth generation affects the evolution of tooth genes and proteins within these groups.

### Divergence in the Evolutionary Trajectory of Tooth-Specific Genes Between Pleurodonta and Acrodonta

We found a significant difference between pleurodont and acrodont lizard orthologs in all analyses performed. We initially hypothesized that acrodont lizards would exhibit stronger purifying selection on their tooth-specific genes. Since acrodont teeth are for long-term use, even slightly deleterious mutations in tooth structure may have a significant negative effect on the functionality of the teeth in these lizards. This may not be the case in pleurodont lizards, where teeth can cycle as often as every 6–7 months (Grieco and Richman 2018). This hypothesis was further supported by functional distance analysis of proteins, which found that larger proportion of Type-I divergence has accumulated in the Acrodont lineage, when compared to the most recent common ancestor between pleurodonts, acrodonts, and mammals. Type-I functional divergence results in a site-specific shift in evolutionary rate (Gu 1999), which in this case is shown to have occurred in Acrodonta, presumably due to the loss of tooth replacement. Indeed, our substitution rate analysis supports this finding, revealing consistently lower rates in acrodonts compared to pleurodonts. That said we did not find any significant Type-II divergence between acrodonts and pleurodonts, showing that while rate of evolution does differ between them, this did not result in the fixation of different physicochemical properties in the amino acid sites of enamel proteins (Gu et al. 2013).

The idea of lower constraint on the tooth genes of polyphyodont reptiles and amphibians has been proposed before (Delgado et al. 2007; Assaraf-Weill et al. 2013). However, limited non-mammalian sequences have hindered a more comprehensive understanding of how tooth-specific genes evolve within the various groups. Here, we not only show that tooth-specific genes differ in selection pressure, but that a large proportion of these differences were focused on proteins directly involved in producing structural components of teeth, as opposed to simply being associated with tooth development as a process. Biochemical studies of the differences between pleurodonts and acrodont lizard EMPs and enamel, or between chameleons and agamids, would be a fascinating next step in better understanding these differences.

### Previously Unrecognized Variation Within Acrodonta

When we analyzed chameleons and agamids separately, we found differences between them that were proportionate to their cumulative difference with pleurodonts. Within squamates, lifelong tooth replacement has ostensibly been limited in only two groups of lizards: chameleons and agamids. Despite chameleons being famous for their remarkable phenotypic adaptations, they have been grouped with agamids as sister clades as far back as the early nineteenth century based on overall morphology and dentition, and modern molecular methods have further supported this idea (Pyron et al. 2013; Reeder et al. 2015; Schulte et al. 2020).

That said, the molecular differences we have identified in this study might be explained by differences in their dentition that have recently come to light. Whether the reduction of tooth replacement is synapomorphic or homoplastic in Acrodonta will require further study, however, differences between them do exist. Studies of *Agama* and *Pogona* have shown that tooth replacement was not lost completely in agamids. Members of Agamidae possess monophyodont dentition in the posterior of the jaw, with typical acrodont teeth that ankylose to the crest of the jawbone and are never replaced (Cooper et al. 1970; Salomies 2019). However, the anterior teeth in juvenile animals exhibit a pleurodont phenotype and retain a limited capacity for replacement, undergoing at least three or four generations before they also become permanent (Cooper et al. 1970; Salomies 2019). *Uromastyx* is once again unique in this aspect in that it has dispensed with even the limited tooth replacement that other agamids exhibit (Cooper and Poole 1973). Chameleons, on the other hand, exhibit a complete loss of tooth replacement, with a single tooth generation forming during embryonic development, which then becomes ankylosed or fused to the jaw in an acrodont conformation (Buchtová et al. 2013). In the chameleon, the ankylosis is so complete that the joint between the tooth and bone may even be difficult to discern (Buchtová et al. 2013). Therefore, chameleons seemingly exhibit a more complete transition to monophyodont dentition than agamids. This difference may explain why AMEL, the most abundant component of forming enamel, exhibits the strongest purifying selection in chameleons. The disparity between chameleons and agamids is also exemplified in our functional distance analysis, where chameleons exhibit strong divergence in their EMPs. However, when it comes to non-EMP proteins, Agamidae exhibit similar level of divergence to pleurodonts, while chameleons are much more conserved. This may be due to a lower selection pressure on non-EMPs, leading to a divergence pattern that correlates with the generally lower substitution rate in chameleons and higher substitution rates in agamids and pleurodonts, rather than selection pressure in one direction or the other.

### Convergence Between Acrodont Lizards and Mammals

This study was partly undertaken in order to identify convergence that may have taken place between acrodont lizards and mammals. Selection analysis did show more stringent purifying selection on EMPs in acrodont lizards and mammals when compared to pleurodonts. Among non-EMP genes, mammals exhibited stronger purifying selection except in AMTN. AMTN appears to be highly variable across tetrapods, exhibiting several exon losses in frog (*X. tropicalis*) and mammals, including loss of phosphorylated serine residues near the N-terminus, as well as a conserved Arg-Gly-Asp (RGD) motif (putative integrin-binding site) near the C-terminus as mammals evolve from monotremes to eutherians (Gasse et al. 2015). Indeed, our analyses also reflected significant variability. When convergence analysis was performed using PCOC, EMPs showed significantly higher number of convergent amino acid sites shared between mammals and acrodont lizards than non-EMP proteins, with ACP4 and AMTN showing no convergent sites at all under a 95% posterior probability cutoff.

### AMEL Evolution Is Disproportionately Affected by the Loss of Tooth Replacement

Across all analyses, we uncovered marked differences between EMPs (sometimes including AMTN) and non-structural proteins, ACP4 and MMP20. However, even within the EMPs, AMEL stood out as particularly different from the rest across the board. AMEL makes up ~ 90% extracellular matrix proteins secreted by ameloblast cells during amelogenesis (Termine et al. 1980), therefore it is logical that this protein my take on the bulk of selective pressure to modify in lineages, where tooth replacement is reduced or lost. Despite its relatively short length, alternative splicing allows for the generation of at least 16 mRNAs in the mouse (Hu et al. 1997; Bartlett et al. 2006; Li et al. 2006; Gibson 2011), and at least five known from humans (Gibson 2011). Unfortunately, because of their heterogeneity, the function of each isoform has not been described (Gibson 2011; Haruyama et al. 2011). Below we propose several hypotheses for why AMEL could be so different between the various groups in this study.

I. Non-tooth-related roles of AMEL could have skewed the evolutionary trajectory of this protein, applying selective pressure in a different direction than if it only functioned in amelogenesis. Emerging evidence suggests that amelogenins are actually multifunctional proteins and not just extracellular matrices for enamel mineralization (Gruenbaum-Cohen et al. 2009; Haruyama et al. 2011). Different isoforms of amelogenin have been found in other tissues associated with odontogenesis such as dentin, odontoblasts, cementum, periodontal ligament, and even the dental lamina before extracellular enamel or dentin is formed (Gruenbaum-Cohen et al. 2009). AMEL has also been found elsewhere in the body, not directly associated with teeth, including both mineralized and non-mineralized tissue such as osteocytes, osteoblasts, and osteoclasts and bone marrow cells in long bones, as well as the glial cells in the brain, salivary glands, megakaryocytes and macrophage, retina of the eye, peripheral ganglia, and peripheral nerve trunk (reviewed by Gruenbaum-Cohen et al. 2009). However, the “importance” of these other functions is difficult to quantify. As our group and others have previously demonstrated, amelogenin accumulates disruptive mutations and becomes a pseudogene in all lineages where teeth are lost (Meredith et al. 2013; Shaffer et al. 2013; Kawasaki et al. 2020; Shaheen et al. 2021). This phenomenon demonstrates that the most important role that amelogenin appears to play is in odontogenesis, and in any other roles it serves, it is supplanted as soon as AMEL protein becomes unnecessary for tooth formation. That said, the question of how these other “jobs” of AMEL affect bioinformatics analyses of selection and differentiation has yet to be elucidated. It is plausible that non-dental functions are influencing the evolutionary trajectory of AMEL in some capacity. Further investigation in physiological differences between the species, as well as a more comprehensive understanding of where AMEL functions, are needed before drawing further conclusions.
II. Are there structural differences in reptile enamel that affect the evolution of AMEL? Amelogenin is the predominant biomineralization protein in the early stages of amelogenesis. In mammals, amelogenin is essential for the organization of the prismatic pattern, control of crystal size and length, as well as enamel thickness (Paine et al. 2000; Gibson et al. 2001; Wright et al. 2011; Smith et al. 2016). In vitro studies show that it is capable of self-assembly into a variety of quaternary structures, oligomers, nanospheres, and nanoribbons, the shapes of which have been proposed to contribute to the formation of hydroxyapatite crystals through stabilization of amorphous calcium phosphate particles (Wiedemann-Bidlack et al. 2011; Shaw et al. 2020). Reptiles (with the exception of *Uromastyx*) are thought to develop “aprismatic” enamel. Yet, it is not implausible that more advanced analytical techniques may elucidate hidden structural differences between acrodont and pleurodont lizard enamel, as well as differences between chameleons and agamids, which could reflect the evolution of AMEL protein and gene sequence in these groups.
III. AMEL may play a role in the remodeling of alveolar bone and ankylosis of teeth to the jaw in acrodont lizards. Recent studies have shown that recombinant human amelogenin protein is capable of causing regeneration of all three tooth supporting tissues, alveolar bone, periodontal ligament, and cementum (Gruenbaum-Cohen et al. 2009). Furthermore, immunohistochemical studies suggest that amelogenin may induce recruitment of mesenchymal stem cells and/or progenitor cells, during the regeneration of the tooth supporting tissues (Deutsch et al. 2006). Amelogenin protein is also proposed to function as a signaling molecule in mesenchymal cells by suppressing osteoclastogenesis (Haruyama et al. 2011), suggesting that it may play a role in bone buildup during ankylosis of teeth to the jaw. In fact, the therapeutic application of an enamel matrix derivative rich in amelogenins resulted in the regeneration of cementum, alveolar bone, and periodontal ligament, in the experimental treatment of human periodontitis, pointing towards novel roles for amelogenin in hard tissue formation (Haruyama et al. 2011).

## Conclusion

In conclusion, we show here that the molecular components of developing teeth are indeed affected by the loss of tooth replacement. Akin to the physical modification of teeth, the molecular components of forming enamel experience changes in their selection pressure, substitution rate, and amino acid consistency. Moreover, we have revealed a substantial divergence between chameleons and agamid lizards, and their enamel matrix proteins. These differences may reflect as yet undetected differences in enamel structure in these groups. We believe that the categorization of reptile enamel as simply “aprismatic” is a byproduct of technological limitations and predict that the application of newer and more advanced techniques will likely show differences in enamel structure between the various reptile groups. We also show that amelogenin, the major component of forming enamel, appears to be most sensitive to changes in tooth replacement regimen. This is reasonable since retention of teeth for longer period of time puts evolutionary pressure directly on enamel structure. Elucidating more details of the role that amelogenin plays during amelogenesis will someday allow us to better understand how differences in amelogenin protein structure translate to changes in the physical structure of enamel.

## Supplementary Information

Below is the link to the electronic supplementary material.

Supplementary file1 (DOCX 130 KB)

Supplementary file2 (DOCX 101 KB)

Supplementary file3 (DOCX 83 KB)

Supplementary file4 (DOCX 473 KB)

Supplementary file5 (DOCX 20 KB)

Supplementary file6 (XLSX 12 KB)
