# Supplementary material for: Reduction of Tooth Replacement Disproportionately Affects the Evolution of Enamel Matrix Proteins
Source: J Mol Evol. 2025 Aug 7;93(4):494–510. doi: 10.1007/s00239-025-10258-4 (PMC12354546; doi:10.1007/s00239-025-10258-4)
Supplement: Supplementary file 1 — Supplementary file1 (DOCX 130 KB) [file 239_2025_10258_MOESM1_ESM.docx]

**Table S1** CodeML selection analysis using branch models with codon frequency model 2

| **AMBN-Model** | **ω categories** | **ω_a_** | **ω_p_** | **ω_m_** |  | **LRT** | **2Δl** | **df** |
| --- | --- | --- | --- | --- | --- | --- | --- | --- |
| Free - ratio | **-** |  |  |  |  |  |  |  |
| One - ratio | ω = 0.39 |  |  |  |  | Free *vs.* One | 214.06** | 100 |
| M2a | ω_i_, ω_m_ | ω_a + p_ = 0.38 | | 0.40 |  | One *vs.* M2a | 0.49ns | 2 |
| M2b | ω_a_, ω_p_, ω_m_ | 0.32 | 0.45 | 0.40 |  | M2a *vs.* M2b | 9.08** | 2 |
| M2c | ω_ch_, ω_agam_, ω_p_, ω_m_ | ω_ch_ = 0.44 ω_agam_ = 0.25 | 0.44 | 0.40 |  | M2b *vs.* M2c | 15.07** | 2 |
|  |  |  |  |  |  |  |  |  |
| **AMEL-Model** | **ω categories** | **ω_a_** | **ω_p_** | **ω_m_** |  | **LRT** | **2Δl** | **df** |
| Free - ratio | **-** |  |  |  |  |  |  |  |
| One - ratio | ω = 0.39 |  |  |  |  | Free *vs.* One | 146.21** | 102 |
| M2a | ω_a + p_, ω_m_ | ω_a + p_ = 0.48 | | 0.26 |  | One *vs.* M2a | 16.99** | 2 |
| M2b | ω_a_, ω_p_, ω_m_ | 0.35 | 0.60 | 0.25 |  | M2a *vs.* M2b | 8.63** | 2 |
| M2c | ω_ch_, ω_agam_, ω_p_, ω_m_ | ω_ch_ = 0.26 ω_agam_ = 0.42 | 0.60 | 0.29 |  | M2b *vs.* M2c | 5.72 * | 2 |
|  |  |  |  |  |  |  |  |  |
| **ENAM-Model** | **ω categories** | **ω_a_** | **ω_p_** | **ω_m_** |  | **LRT** | **2Δl** | **df** |
| Free - ratio | **-** |  |  |  |  |  |  |  |
| One - ratio | ω = 0.47 |  |  |  |  | Free *vs.* One | 179.28** | 100 |
| M2a | ω_a + p_, ω_m_ | ω_a + p_ = 0.52 | | 0.40 |  | One *vs.* M2a | 31.12 ** | 2 |
| M2b | ω_a_, ω_p_, ω_m_ | 0.48 | 0.58 | 0.40 |  | M2a *vs.* M2b | 12.85** | 2 |
| M2c | ω_ch_, ω_agam_, ω_p_, ω_m_ | ω_ch_ = 0.50  ω_agam_ = 0.44 | 0.58 | 0.40 |  | M2b *vs.* M2c | 5.16 * | 2 |
|  |  |  |  |  |  |  |  |  |
| **AMTN-Model** | **ω categories** | **ω_a_** | **ω_p_** | **ω_m_** |  | **LRT** | **2Δl** | **df** |
| Free - ratio | **-** |  |  |  |  |  |  |  |
| One - ratio | ω = 0.44 |  |  |  |  | Free *vs.* One | 153.97** | 100 |
| M2a | ω_a + p_, ω_m_ | ω_a + p_ = 0.39 | | 0.57 |  | One *vs.* M2a | 15.14 ** | 2 |
| M2b | ω_a_, ω_p_, ω_m_ | 0.37 | 0.46 | 0.58 |  | M2a *vs.* M2b | 17.05** | 2 |
| M2c | ω_ch_, ω_agam_, ω_p_, ω_m_ | ω_ch_ = 0.52  ω_agam_ = 0.32 | 0.46 | 0.58 |  | M2b *vs.* M2c | 7.43* | 2 |
|  |  |  |  |  |  |  |  |  |
| **ACP4-Model** | **ω categories** | **ω_a_** | **ω_p_** | **ω_m_** |  | **LRT** | **2Δl** | **df** |
| Free - ratio | **-** |  |  |  |  |  |  |  |
| One - ratio | ω = 0.22 |  |  |  |  | Free *vs.* One | 120.94** | 100 |
| M2a | ω_a + p_, ω_m_ | ω_a + p_ = 0.27 | | 0.16 |  | One *vs.* M2a | 29.84** | 2 |
| M2b | ω_a_, ω_p_, ω_m_ | 0.28 | 0.27 | 0.17 |  | M2a *vs.* M2b | 6.00 * | 2 |
| M2c | ω_ch_, ω_agam_, ω_p_, ω_m_ | ω_ch_ = 0.31  ω_agam_ = 0.26 | 0.26 | 0.17 |  | M2b *vs.* M2c | 1.12 ns | 2 |
|  |  |  |  |  |  |  |  |  |
| **MMP20-Model** | **ω categories** | **ω_a_** | **ω_p_** | **ω_m_** |  | **LRT** | **2Δl** | **df** |
| Free - ratio | **-** |  |  |  |  |  |  |  |
| One - ratio | ω = 0.15 |  |  |  |  | Free *vs.* One | 181.21** | 100 |
| M2a | ω_a + p_, ω_m_ | ω_a + p_ = 0.17 | | 0.13 |  | One *vs.* M2a | 8.07 ** | 2 |
| M2b | ω_a_, ω_p_, ω_m_ | 0.19 | 0.15 | 0.13 |  | M2a *vs.* M2b | 2.88 ns | 2 |
| M2c | ω_ch_, ω_agam_, ω_p_, ω_m_ | ω_ch_ = 0.16  ω_agam_ = 0.20 | 0.15 | 0.13 |  | M2b *vs.* M2c | 1.64 ns | 2 |

P < 0.05*, P < 0.01**, NS – not significant ; ω_a + p_ = Iguania
