# Supplementary material for: Reduction of Tooth Replacement Disproportionately Affects the Evolution of Enamel Matrix Proteins
Source: J Mol Evol. 2025 Aug 7;93(4):494–510. doi: 10.1007/s00239-025-10258-4 (PMC12354546; doi:10.1007/s00239-025-10258-4)
Supplement: Supplementary file 2 — Supplementary file2 (DOCX 101 KB) [file 239_2025_10258_MOESM2_ESM.docx]

| **ACP4** | **Iguan/Mam** |  |  |
| --- | --- | --- | --- |
| **Theta-II** | 0.15685 | **Z-score** | 2.613818158 |
| **SE Theta** | 0.060008 | Z-score P value | .008954 |
|  |  |  |  |
|  |  |  |  |
| **AMBN** | **Iguan/Mam** |  |  |
| **Theta-II** | 0.258994 | **Z-score** | 2.519592964 |
| **SE Theta** | 0.102792 | Z-score P value | 0.011752 |
|  |  |  |  |
|  |  |  |  |
| **AMEL** | **Iguan/Mam** |  |  |
| **Theta-II** | 0.034583 | **Z-score** | 0.311819813 |
| **SE Theta** | 0.110907 | Z-score P value | 0.755193 |
|  |  |  |  |
|  |  |  |  |
| **AMTN** | **Iguan/Mam** |  |  |
| **Theta-II** | 0.478917 | **Z-score** | 2.508522073 |
| **SE Theta** | 0.190916 | Z-score P value | 0.012124 |
|  |  |  |  |
|  |  |  |  |
| **ENAM** | **Iguan/Mam** |  |  |
| **Theta-II** | 0.115054 | **Z-score** | 1.144645078 |
| **SE Theta** | 0.100515 | Z-score P value | 0.252375 |
|  |  |  |  |
|  |  |  |  |
| **MMP20** | **Iguan/Mam** |  |  |
| **Theta-II** | 0.069511 | **Z-score** | 1.463975064 |
| **SE Theta** | 0.047481 | Z-score P value | 0.143221 |
|  |  |  |  |

Table S2 - DIVERGE - Type II Analysis Calculations – Iguania-Mammalia

Table S3 - DIVERGE - Type II Analysis Calculations – Acrodont-Pleurodont

| **ACP4** | **Acro/Pleuro** |  |  |
| --- | --- | --- | --- |
| **Theta-II** | -0.019856 | **Z-score** | -0.364564399 |
| **SE Theta** | 0.054465 | Z-score P value | 0.715858 |
|  |  |  |  |
|  |  |  |  |
| **AMBN** | **Acro/Pleuro** |  |  |
| **Theta-II** | 0.05841 | **Z-score** | 0.867389367 |
| **SE Theta** | 0.06734 | Z-score P value | 0.192889 |
|  |  |  |  |
|  |  |  |  |
| **AMEL** | **Acro/Pleuro** |  |  |
| **Theta-II** | 0.099395 | **Z-score** | 1.194235182 |
| **SE Theta** | 0.083229 | Z-score P value | 0.1162 |
|  |  |  |  |
|  |  |  |  |
| **AMTN** | **Acro/Pleuro** |  |  |
| **Theta-II** | 0.02573 | **Z-score** | 0.247470473 |
| **SE Theta** | 0.103972 | Z-score P value | 0.804599 |
|  |  |  |  |
|  |  |  |  |
| **ENAM** | **Acro/Pleuro** |  |  |
| **Theta-II** | -0.044543 | **Z-score** | -0.64375009 |
| **SE Theta** | 0.069193 | Z-score P value | 0.260112 |
|  |  |  |  |
|  |  |  |  |
| **MMP20** | **Acro/Pleuro** |  |  |
| **Theta-II** | 0.007141 | **Z-score** | 0.182760474 |
| **SE Theta** | 0.039073 | Z-score P value | 0.855033 |

Table S4 - DIVERGE - Type II Analysis Calculations – Agamidae- Chamaeleonidae

| **ACP4-IguaniaOnly** | **Agam/Cham** |  |  |
| --- | --- | --- | --- |
| **Theta-II** | -0.039883 | **Z-score** | -1.027065307 |
| **SE Theta** | 0.038832 | Z-score P value | 0.30442 |
|  |  |  |  |
|  |  |  |  |
| **AMBN-IguaniaOnly** | **Agam/Cham** |  |  |
| **Theta-II** | 0.020096 | **Z-score** | 0.506834805 |
| **SE Theta** | 0.03965 | Z-score P value | 0.612295 |
|  |  |  |  |
|  |  |  |  |
| **AMEL-IguaniaOnly** | **Agam/Cham** |  |  |
| **Theta-II** | 0.083376 | **Z-score** | 2.109022842 |
| **SE Theta** | 0.039533 | Z-score P value | 0.034945 |
|  |  |  |  |
|  |  |  |  |
| **AMTN-IguaniaOnly** | **Agam/Cham** |  |  |
| **Theta-II** | 0.026014 | **Z-score** | 0.430502921 |
| **SE Theta** | 0.060427 | Z-score P value | 0.666832 |
|  |  |  |  |
|  |  |  |  |
| **ENAM-IguaniaOnly** | **Agam/Cham** |  |  |
| **Theta-II** | 0.008092 | **Z-score** | 0.212444211 |
| **SE Theta** | 0.03809 | Z-score P value | 0.831795 |
|  |  |  |  |
|  |  |  |  |
| **MMP20-IguaniaOnly** | **Agam/Cham** |  |  |
| **Theta-II** | 0.009707 | **Z-score** | 0.310355853 |
| **SE Theta** | 0.031277 | Z-score P value | 0.756333 |
