# Supplementary material for: Reduction of Tooth Replacement Disproportionately Affects the Evolution of Enamel Matrix Proteins
Source: J Mol Evol. 2025 Aug 7;93(4):494–510. doi: 10.1007/s00239-025-10258-4 (PMC12354546; doi:10.1007/s00239-025-10258-4)
Supplement: Supplementary file 3 — Supplementary file3 (DOCX 83 KB) [file 239_2025_10258_MOESM3_ESM.docx]

| **ACP4** |  | **P** | | | **lnl** | | **One Rate** | **Mammal** | **Pleurodont** | **Acrodont** |
| --- | --- | --- | --- | --- | --- | --- | --- | --- | --- | --- |
| M1 - Global Clock | 1 rate: | 47 | | | -13884.09516 | | 0.088860 +- 0.004589 |  |  |  |
| M2a-Local clock | 3 rate: | 50 | | | -13817.93873 | |  | 0.132273 +- 0.008755 | 0.078157 +- 0.004694 | 0.059427 +- 0.004036 |
|  |  |  | | |  | |  | **Mammal** | **Pleurodont** | **Chameleon** |
| M2b-Local clock | 4 rates: | 51 | | | -13817.25932 | |  | 0.132273 +- 0.008765 | 0.078211 +- 0.004710 | 0.054463 +- 0.005546 |
|  | | | | | | | | | | |
| **LR-TEST** | **2∆** l | | df | P= | | **LR-TEST** | | **2∆** l |  |  |
| 1-rate vs 3-rate | 132.312872 | | 3 | 8.52E-29 | | 1-rate vs 3-rate | | 132.312872 |  |  |
| 3-rate vs 4-rate | 1.358812 | | 1 | 1.73E-01 | | 3-rate vs 4-rate | | 1.358812 |  |  |

Table S5

| **AMBN** |  | **P** | | | **lnl** | | **One Rate** | **Mammal** | **Pleurodont** | **Acrodont** |
| --- | --- | --- | --- | --- | --- | --- | --- | --- | --- | --- |
| M1 - Global Clock | 1 rate: | 49 | | | -15189.49606 | | 0.098338 +- 0.003819 |  |  |  |
| M2a-Local clock | 3 rate: | 52 | | | -15114.80041 | |  | 0.151693 +- 0.008899 | 0.095645 +- 0.004609 | 0.063903 +- 0.003496 |
|  |  |  | | |  | |  | **Mammal** | **Pleurodont** | **Chameleon** |
| M2b-Local clock | 4 rates: | 53 | | | -15108.84057 | |  | 0.151709 +- 0.008926 | 0.097561 +- 0.004807 | 0.058380 +- 0.005032 |
|  | | | | | | | | | | |
| **LR-TEST** | **2∆** l | | df | P= | | **LR-TEST** | | **2∆** l |  |  |
| 1-rate vs 3-rate | 149.391298 | | 3 | 1.77E-32 | | 1-rate vs 3-rate | | 149.391298 |  |  |
| 3-rate vs 4-rate | 11.919688 | | 1 | 2.93E-04 | | 3-rate vs 4-rate | | 11.919688 |  |  |

| **AMEL** |  | **P** | | | **lnl** | | **One Rate** | **Mammal** | **Pleurodont** | **Acrodont** |
| --- | --- | --- | --- | --- | --- | --- | --- | --- | --- | --- |
| M1 - Global Clock | 1 rate: | 51 | | | -5710.635838 | | 0.063650 +- 0.005522 |  |  |  |
| M2a-Local clock | 3 rate: | 54 | | | -5681.523855 | |  | 0.055222 +- 0.012273 | 0.089415 +- 0.008814 | 0.040703 +- 0.004879 |
|  |  |  | | |  | |  | **Mammal** | **Pleurodont** | **Chameleon** |
| M2b-Local clock | 4 rates: | 55 | | | -5679.445435 | |  | 0.055244 +- 0.012202 | 0.090234 +- 0.008810 | 0.043179 +- 0.007969 |
|  | | | | | | | | | | |
| **LR-TEST** | **2∆** l | | df | P= | | **LR-TEST** | | **2∆** l |  |  |
| 1-rate vs 3-rate | 58.223966 | | 3 | 6.92E-13 | | 1-rate vs 3-rate | | 58.223966 |  |  |
| 3-rate vs 4-rate | 4.15684 | | 1 | 2.45E-02 | | 3-rate vs 4-rate | | 4.15684 |  |  |
|  |  | |  |  | |  | |  |  |  |

| **AMTN** |  | **P** | | | **lnl** | | **One Rate** | **Mammal** | **Pleurodont** | **Acrodont** |
| --- | --- | --- | --- | --- | --- | --- | --- | --- | --- | --- |
| M1 - Global Clock | 1 rate: | 50 | | | -11938.55601 | | 0.108880 +- 0.004711 |  |  |  |
| M2a-Local clock | 3 rate: | 53 | | | -11815.47857 | |  | 0.220901 +- 0.016532 | 0.091826 +- 0.004958 | 0.066242 +- 0.004052 |
|  |  |  | | |  | |  | **Mammal** | **Pleurodont** | **Chameleon** |
| M2b-Local clock | 4 rates: | 54 | | | -11809.53288 | |  | 0.220692 +- 0.016503 | 0.092766 +- 0.005098 | 0.051888 +- 0.005815 |
|  | | | | | | | | | | |
| **LR-TEST** | **2∆** l | | df | P= | | **LR-TEST** | | **2∆** l |  |  |
| 1-rate vs 3-rate | 246.154884 | | 3 | 2.21E-53 | | 1-rate vs 3-rate | | 246.154884 |  |  |
| 3-rate vs 4-rate | 11.891378 | | 1 | 3.03E-04 | | 3-rate vs 4-rate | | 11.891378 |  |  |

| **ENAM** |  | **P** | | | **lnl** | | **One Rate** | **Mammal** | **Pleurodont** | **Acrodont** |
| --- | --- | --- | --- | --- | --- | --- | --- | --- | --- | --- |
| M1 - Global Clock | 1 rate: | 50 | | | -45254.78121 | | 0.101243 +- 0.001980 |  |  |  |
| M2a-Local clock | 3 rate: | 53 | | | -45154.80829 | |  | 0.134092 +- 0.003737 | 0.094306 +- 0.002667 | 0.079749 +- 0.002401 |
|  |  |  | | |  | |  | **Mammal** | **Pleurodont** | **Chameleon** |
| M2b-Local clock | 4 rates: | 54 | | | -45146.80565 | |  | 0.134079 +- 0.003742 | 0.094909 +- 0.002710 | 0.069631 +- 0.003532 |
|  | | | | | | | | | | |
| **LR-TEST** | **2∆** l | | df | P= | | **LR-TEST** | | **2∆** l |  |  |
| 1-rate vs 3-rate | 199.945836 | | 3 | 2.16E-43 | | 1-rate vs 3-rate | | 199.945836 |  |  |
| 3-rate vs 4-rate | 16.005282 | | 1 | 3.34E-05 | | 3-rate vs 4-rate | | 16.005282 |  |  |
|  |  | |  |  | |  | |  |  |  |

| **MMP20** |  | **P** | | | **lnl** | | **One Rate** | **Mammal** | **Pleurodont** | **Acrodont** |
| --- | --- | --- | --- | --- | --- | --- | --- | --- | --- | --- |
| M1 - Global Clock | 1 rate: | 50 | | | -15232.07643 | | 0.068734 +- 0.003221 |  |  |  |
| M2a-Local clock | 3 rate: | 53 | | | -15155.80178 | |  | 0.106140 +- 0.006238 | 0.055365 +- 0.003055 | 0.050520 +- 0.002962 |
|  |  |  | | |  | |  | **Mammal** | **Pleurodont** | **Chameleon** |
| M2b-Local clock | 4 rates: | 54 | | | -15148.81205 | |  | 0.106127 +- 0.006246 | 0.055673 +- 0.003089 | 0.039514 +- 0.003554 |
|  | | | | | | | | | | |
| **LR-TEST** | **2∆** l | | df | P= | | **LR-TEST** | | **2∆** l |  |  |
| 1-rate vs 3-rate | 152.549302 | | 3 | 3.69E-33 | | 1-rate vs 3-rate | | 152.549302 |  |  |
| 3-rate vs 4-rate | 13.979464 | | 1 | 9.83E-05 | | 3-rate vs 4-rate | | 13.979464 |  |  |
|  |  | |  |  | |  | |  |  |  |
