# Supplementary material for: Reduction of Tooth Replacement Disproportionately Affects the Evolution of Enamel Matrix Proteins
Source: J Mol Evol. 2025 Aug 7;93(4):494–510. doi: 10.1007/s00239-025-10258-4 (PMC12354546; doi:10.1007/s00239-025-10258-4)
Supplement: Supplementary file 4 — Supplementary file4 (DOCX 473 KB) [file 239_2025_10258_MOESM4_ESM.docx]

**ACP4-AA-MAFFT-Iguania+Mammal – DIVERGE + PCOC ≥ 0.9**

CLUSTAL W (1.8) multiple sequence alignment (ALTER 1.3.3)

Acanthocercus_cyanogaster_Acr MR---LSASLIS--LIIFLQAIL---PLIAEQERRLRFVILVYRHGDRSPLGTYPTDPHK 52

Acanthocercus_minutus_Acr MR---LSASLIS--LMIFLQAIL---PLIAEQERRLRFVILVYRHGDRSPLGTYPTDPHK 52

Xenagama_zonura_Acr MR---LSASLIS--LMIFLQAIL---PLIAEQERRLRFVILVYRHGDRSPLGTYPTDPHK 52

Laudakia_wui_Acr MR---LSASLTS--LMILLQAIL---PLIAEEERRLRFVILVYRHGDRSPLGTYPTDPHK 52

Phrynocephalus_forsythii_Acr MR---LSASLAS--LMILLQAIL---PLIAEQERRLRFVVLLYRHGDRSPLGTYPTDPHK 52

Phrynocephalus_guinanensis_Acr MR---LS---AS--LMILLQAIL---PLIAEQERRLRFVILVYRHGDRSPLGTYPTDPHK 49

Phrynocephalus_vlangalii_Acr MR---LSASLAS--LMILLQAIL---PLIAEQERRLRFVILVYRHGDRSPLGTYPTDPHK 52

Phrynocephalus_versicolor_Acr MR---LSASLAS--LMILLQAIL---PLIAEQERRLRFVILVYRHGDRSPLGTYPTDPHK 52

Agama_doriae_Acr MR---LSASLIS--LTVFLQAIL---PLIAEQERKLRFVILVYRHGDRSPLGTYPTDPHK 52

Intellagama_lesueurii_Acr MR---LSASLAS--LMIILQAIL---PLIAEQERRLRFVILVYRHGDRSPLGTYPTDPHK 52

Pogona_vitticeps_Acr MR---LS---AS--LIIILQAIL---PLIAEQERRLRFVILVYRHGDRSPLGTYPTDPHK 49

Bradypodion_pumilum_Acr MR---LSAALTS--LMIFLQPIL---PLTAEQERTLRFIILVYRHGDRSPLGTYPTDPHK 52

Bradypodion_ventrale_Acr MR---LSAALTS--LMIFLQPIL---PLTAEQERTLRFIILVYRHGDRSPLGTYPTDPHK 52

Chamaeleo_calyptratus_Acr MR---LSAALTS--LTIFLQTIL---PLTAEQERTLRFIVLVYRHGDRSPLGTYPTDPHK 52

Chamaeleo_dilepis_Acr MR---LSAVLTS--LMIFLQTIL---PLTAEQERTLRFIVLVYRHGDRSPLGTYPTDPHK 52

Chamaeleo_laevigatus_Acr MR---LSAALTS--LMIFLQTIL---PLTAEQERTLRFIILVYRHGDRSPLGTYPTDPHK 52

Chamaeleo_gracilis_Acr MR---LSAALTS--LMIFLQTIL---PLTAEQERTLRFSVLVYRHGDRSPLSTYPTDPHK 52

Trioceros_affinis_Acr MR---LSAVLTS--LMIFLQPIL---PLTAEQERTLRFIILVYRHGDRSPLGTYPTDPHK 52

Trioceros_harennae_Acr MR---LSAVLTS--LMIFLQPIL---PLTAEQERTLRFIILVYRHGDRSPLGTYPTDPHK 52

Furcifer_pardalis_Acr MR---LSAALTS--LMIFL---L---PLAAEQERTLRFIILVYRHGDRSPLGTYPTDPHK 49

Trioceros_balebicornutus_Acr MR---LSAVLTS--LMIFLQPIL---PLTAEQERTLRFIILVYRHGDRSPLGTYPTDPHK 52

Anolis_apletophallus_Pleu MR---LSETIPP--LMVFLQNIL---QLATGQERTLRFVTLVYRHGDRSPLGTYPTDPHK 52

Anolis_tropidonotus_Pleu MR---LSETIPH--LMIFLQNIF---QLATGQERTLRFVTLVYRHGDRSPLGTYPTDPHK 52

Anolis_carolinensis_Pleu MR---LSETLAA--LVVFLQNIL---HFTTGQERTLRFVTLVYRHGDRSPLGTYPTDPHK 52

Anolis_sagrei_ordinatus_Pleu MR---LSDTLVP--LMVFLQNVL---HLTTGQERTLRFVTLVYRHGDRSPLGTYPTDPHK 52

Ctenosaura_bakeri_Pleu MR---LSEALAP--LMIFLHDIL---HLTTGQERTLRFVTLVYRHGDRSPLGTYPTDPHK 52

Iguana_delicatissima_Pleu MR---LSEALTP--LMIFLHDIL---HLTTGQERTLRFVTLVYRHGDRSPLGTYPTDPHK 52

Cyclura_pinguis_Pleu MR---LSEALTP--LMIFLHDIL---HLTTGQERTLRFVTLVYRHGDRSPLGTYPTDPHK 52

Laemanctus_serratus_Pleu MR---LSEILTA--LMILLHNIL---HLTTGQERTLRFVTLVFRHGDRSPLGTYPTDPHK 52

Phrynosoma_blainvillii_Pleu MR---VSETLAP--LMIFIHNIF---HLTAGQERTLRSVTLVYRHGDRSPLGTYPTDPHK 52

Sceloporus_chrysostictus_Pleu MR---LSGTLAS--LMICIHNIL---HFAAGQERTLRFVTLVYRHGDRSPLGTYPTDPHK 52

Sceloporus_occidentalis_Pleu MR---LSETLAS--LMICIHNIL---HFTAGQERTLRFVTLVYRHGDRSPLGTYPTDPHK 52

Sceloporus_tristichus_Pleu MR---LSETLAS--LMICIHNSL---HFTAGQERTLRFVTLIYRHGDRSPLGTYPTDPHK 52

Sceloporus_undulatus_Pleu MR---LSETLAS--LMICIHNSL---HFTAGQERTLRFVTLIYRHGDRSPLGTYPTDPHK 52

Urosaurus_nigricaudus_Pleu MR---LPETLAS--LMVFTHNIL---HFTAGQERTLRFVTLLYRHGDRSPLGTYPTDPHK 52

Gambelia_wislizenii_Pleu MR---LSATLTP--LMIFLHNIM---HLTTAQERTLRFVTLVYRHGDRSPLGTYPTDPHK 52

Canis_lupus_dingo_ACP4 MARPGFWGLPVG---PLLLLLLLLPPPPQALTEGPLVFVAVVFRHGDRAPLASYPTDPHK 57

Vulpes_vulpes_ACP4 MARPGFWGLPAG---PLLLLLLLLPPPPQALTEGPLVFVAVVFRHGDRAPLASYPTDPHK 57

Meles_meles_ACP4 MAGPGFGGLPAG---LLLLLPLL---RPQALTEGPLVFVAVVFRHGDRAPLASYPTDPHK 54

Mustela_putorius_ACP4 MRRH-----------------------------------REVFRHGDRAPLASYPTDPHK 25

Ursus_arctos_ACP4 MAGPGFWGLPAG---LLLLLLLL---APWALAEGPLVFVAVVFRHGDRAPLASYPTDPHK 54

Phacochoerus_africanus_ACP4 MAGPGFRGHPAGP--LLLLLLLL---LRQALTEGPLVFVAVVFRHGDRAPLASYPTDPHK 55

Phyllostomus_hastatus_ACP4 MAGPGFQGHPAG---PLLLLLLL---LPQGLMEGPLVFVAVVFRHGDRAPLASYPTDPHK 54

Homo_sapiens_ACP4 MAGLGFWGHPAGP-LLLLLLLVL---PPRALPEGPLVFVALVFRHGDRAPLASYPMDPHK 56

Saimiri_boliviensis_ACP4 MAGLGFWGHPAAS--LLLLLLLL---PPWALPEGPLVFVALVFRHGDRAPLASYPTDPHK 55

Erinaceus_europaeus_ACP4 MAGPGLGGHSAA---ERLLLLLL---LPLAQAEGPLMFVAVVFRHGDRAPLASYPTDPHK 54

Echinops_telfairi_ACP4 MAGPGAQGHRAV---PLLLLLLL---GTQTVAKGPLVFVALVFRHGDRAPLASYPLDPHK 54

Suncus_etruscus_ACP4 MS--GFWGPPATPFLFLLPLLLL---PPPALPEGPLVFVALVFRHGDRAPLASYPTDPHK 55

* ::*****:**.:** ****

Acanthocercus_cyanogaster_Acr ---SEAWPQGFHQL--TEAGILQQKALGQFLRERYNGFLSPSYKPQEIYVRSTDYDRTIM 107

Acanthocercus_minutus_Acr ---SEAWPQGFHQL--TEAGILQQKALGQFLRERYNGFLSPSYKPQEIYVRSTDYDRTIM 107

Xenagama_zonura_Acr ---SEAWPQGFHQL--TEAGILQQKALGQFLRERYNGFLSPSYKPQEIYIRSTDYDRTIM 107

Laudakia_wui_Acr ---SEAWPQGFHQL--TEAGVLQQKALGQFLRERYNGFLSPSYKPQEIYVRSTDYDRTIM 107

Phrynocephalus_forsythii_Acr ---SDAWPQGFHQL--TEDGVLQQKALGQFLRERYNGFLSPSYKQQEIYIRSTDYDRTIM 107

Phrynocephalus_guinanensis_Acr ---SDAWPQGFHQL--TEAGVLQQKALGQFLRERYNGFLSPSYKQQEIYVRSTDYDRTIM 104

Phrynocephalus_vlangalii_Acr ---SDAWPQGFHQL--TEAGVLQQKVLGQFLKEKYNGFLSPSYKQQEIYVRSTDYDRTIM 107

Phrynocephalus_versicolor_Acr ---AEAWPQGFHQL--TEAGVLQQKALGQFLREIYNGFLSPSYKQQEIYVRSTDYDRTIM 107

Agama_doriae_Acr ---AEAWPQGFHQL--TKAGILQQKALGQFLRERYNGFLSPSYKPQEIYVRSTDYDRTIM 107

Intellagama_lesueurii_Acr ---SEAWPQGFHQL--TKVGILQQKVLGRFLRKRYNGFLSPSYKPQEIYVRSTDYDRTIM 107

Pogona_vitticeps_Acr ---SEAWPQGFHQL--TEVGILQQKVLGRFLRKRYNGFLSPSYKPQEIYVRSTDYDRTIM 104

Bradypodion_pumilum_Acr ---AEAWPQGFQQLRGRMVGILQQKTLGRFLRERYDGFLSPSYKRQEIYIRSTDYDRTIM 109

Bradypodion_ventrale_Acr ---AEAWPQGFQQL--TEVGILQQKTLGQFLRERYDGFLSPSYKRQEIYIRSTDYDRTIM 107

Chamaeleo_calyptratus_Acr ---AEAWPQGFQQL--TEVGILQQKTLGRFLRERYDGFLSPSYKRQEIYIRSTDYDRTIM 107

Chamaeleo_dilepis_Acr ---AEAWPQGFQQL--TEVGILQQKTLGQFLRERYDGFLSPSYQRQEIYIRSTDYDRTIM 107

Chamaeleo_laevigatus_Acr ---AEAWPQGFQQL--TEVGILQQKTLGRFLRERYDGFLSPSYKRQEIYIRSTDYDRTIM 107

Chamaeleo_gracilis_Acr ---AEAWPQGFQQL--TEVGILQQKTLGRFLRERYDGFLSPSYKRQEIYIRSTDYDRTIM 107

Trioceros_affinis_Acr ---AEAWPQGFQQL--TEIGILQQKTLGRFLRERYDGFLSPSYKRQEIYIRSTDYDRTIM 107

Trioceros_harennae_Acr ---AEAWPQGFQQL--TEIGILQQKTLGRFLRERYDGFLSPSYKRQEIYIRSTDYDRTIM 107

Furcifer_pardalis_Acr ---AEAWPQGFQQL--TEVGILQHKTLGRFLRERYDGFLSPSYKRQEIYIRSTDYDRTIM 104

Trioceros_balebicornutus_Acr ---AEAWPQGFQQL--TEIGILQQKTLGRFLRERYDGFLSPSYKRQEIYIRSTDYDRTIM 107

Anolis_apletophallus_Pleu ---AAAWPEGFQQL--TKVGILQQKALGKFLREKYDGFLSAAYKPQEIYIRSTDYDRTLM 107

Anolis_tropidonotus_Pleu ---AAAWPEGFQQL--TKVGILQQKALGKFLREKYEGFLSAAYKPQEIYIRSTDYDRTLM 107

Anolis_carolinensis_Pleu ---AAAWPEGFQQL--TKVGILQQKALGKFLRQKYDGFLSSAYKPQEIYVRSTDYDRTLM 107

Anolis_sagrei_ordinatus_Pleu ---AAPWPEGFQQL--TKVGILQQKALGKFLREKYDGFLSAAYKPQEIYIRSTDYDRTLM 107

Ctenosaura_bakeri_Pleu ---AAAWPQGFQQL--TKVGILQQKALGSFLREKYDGFLSAAYKPQEIYIRSTDYDRTIM 107

Iguana_delicatissima_Pleu ---AAAWPQGFQQL--TKVGILQQKALGRFLREKYDGFLSAAYKPQEIYIRSTDYDRTIM 107

Cyclura_pinguis_Pleu ---AAAWPQGFQQL--TKVGILQQKALGRFLREKYDGFLSAAYKPQEIYIRSTDYDRTIM 107

Laemanctus_serratus_Pleu ---AAAWPQGFQQL--TKVGILQQKALGKFLREKYDGFLSAAYKPQEIYIRSTDYDRTIM 107

Phrynosoma_blainvillii_Pleu ---ATVWPQGFQQL--TKVGISQQKALGRFLREKYDGFLSATYNPQEIYIRSRDYDRTIM 107

Sceloporus_chrysostictus_Pleu ---AAVWPQGFQQL--TKVGILQQKVLGRFLREKYDGFLSAAYKPQEIYIRSTDYDRTIM 107

Sceloporus_occidentalis_Pleu ---AAVWPQGFQQL--TKVGILQQKALGRFLRKKYDGFLSAAYKPQEIYIRSTDYDRTIM 107

Sceloporus_tristichus_Pleu ---AAVWPQGFQQL--TKIGILQQKALGRFLREKYDGFLSAAYKPQEIYIRSTDYDRTIM 107

Sceloporus_undulatus_Pleu ---AAVWPQGFQQL--TKIGILQQKALGRFLREKYDGFLSAAYKPQEIYIRSTDYDRTIM 107

Urosaurus_nigricaudus_Pleu ---AAVWPQGFQQL--TKVGILQQKALGRFLREKYDGFLSATYKPQEIYIRSTDYDRTIM 107

Gambelia_wislizenii_Pleu ---EAAWPQGFQQL--TEVGILQQKALGRILREKYNGFLSAAYKPQEIYIRSTDYDRTIM 107

Canis_lupus_dingo_ACP4 EAITALWPRGLGQL--TTEGVRQQLELGRFLRSRYEAFLSPEYRREEVYVRSTDFDRTLE 115

Vulpes_vulpes_ACP4 EAITALWPRGLGQL--TTEGVRQQLELGRFLRSRYEAFLSPEYRREEVYVRSTDFDRTLE 115

Meles_meles_ACP4 EAIAAVWPRGLGQL--TGEGVRQQLELGRFLRRRYEAFLSPEYRREEVYVRSTDFDRTLE 112

Mustela_putorius_ACP4 EAIAAVWPRGLGQL--TGEGVRQQLELGRFLRSRYEAFLSPEYRREEVYVRSTDFDRTLE 83

Ursus_arctos_ACP4 EGTTALWPRGLGQL--TGEGVRQQLELGRFLRSRYEDFLSPEYRREEVYVRSTDFDRTLE 112

Phacochoerus_africanus_ACP4 EDIATLWPRGLGQL--TREGVRQQLELGRFLRSRYKSFLSPQYRREEVYIRSTDFDRTLE 113

Phyllostomus_hastatus_ACP4 EAASSLWPRGLGQL--TSEGVRQQLELGRFLRSRYEAFLSPEYQREEVYVRSTDFDRTLE 112

Homo_sapiens_ACP4 EVASTLWPRGLGQL--TTEGVRQQLELGRFLRSRYEAFLSPEYRREEVYIRSTDFDRTLE 114

Saimiri_boliviensis_ACP4 EAASTLWPRGLGQL--TREGVRQQLELGRFLRSRYEAFLSPEYRREEVYVRSTDFDRTLE 113

Erinaceus_europaeus_ACP4 EAVSDLWPRGLGQL--TGEGVRQQLELGRFLRSRYEHFLSPEFRREEVYVRSTDFDRTLE 112

Echinops_telfairi_ACP4 EAAPTLWPRGLGQL--TGEGVRQQLELGRFLRNRYQHFLSPEYWREEVYIRSTDFDRTLE 112

Suncus_etruscus_ACP4 NDESRLWPRGLGQL--TREGVRQQLALGRFLRRRYQNFLAPEFRREEVYVRSTDFDRTLE 113

**.*: ** *: *: ** :*: *. **:. : :*:*:** *:***:

Acanthocercus_cyanogaster_Acr SAQANLMGLYPNP---DPQTDWNPIPVHTVPVKYDKLLKPPTRTCLRYQQLMEETINLPS 164

Acanthocercus_minutus_Acr SAQANLMGLYPNS---DPQIGWNPIPVHTVPVKYDKLLKPPTRTCLRYQQLMEETINLPS 164

Xenagama_zonura_Acr SAQANLMGLYPNP---DPQIGWNPIPVHTVPVKYDKLLKPPTRTCLRYQQLMEETINLPS 164

Laudakia_wui_Acr SAQANLMGLYPNP---DPHIGWSPIPVHTVPVKYDKLLKPPTRTCLRYQQLMEETINLPS 164

Phrynocephalus_forsythii_Acr SAQANLMGLYPNP---DPQIGWSPIPVHTVPVKYDKLLKPPTRACRRYQQLMEETINLPS 164

Phrynocephalus_guinanensis_Acr SAQANLMGLYPNP---DPQIGWNPIPVHTVPVKYDKLLKPPTRACRRYQQLMEETINLPS 161

Phrynocephalus_vlangalii_Acr SAQANLMGLYPNP---DPQIGWSPIPVHTVPVKYDKLLKPPTRACRRYQQLMEETINLPS 164

Phrynocephalus_versicolor_Acr SAQANLMGLYPNP---DPQIGWSPIPVHTVPVKYDKLLKPPTRTCRRYQQLMEETINLPS 164

Agama_doriae_Acr SAQANLMGLYPNP---DPQIGWSPIPVHSVPVKYDKLLKPPTRTCLRYQQLMEETINLPS 164

Intellagama_lesueurii_Acr SAQANLMGLYPNS---DPQTGWSPVPVHTVPVKYDKLLKPPTRTCLRYQQLMEETVNLPS 164

Pogona_vitticeps_Acr SAQANLMGLYPNS---DPQTGWNPIPVHTVPVKYDKLLKPPTRTCLRYQQLMEETVNLPS 161

Bradypodion_pumilum_Acr SAQANLMGLYPNS---APGIGWSPIPVHTVPVKYDKLLKPPLRSCLRYQQLMEETVNLPS 166

Bradypodion_ventrale_Acr SAQANLMGLYPNS---APGIGWSPIPVHTVPVKYDKLLKPPLRSCLRYQQLMEETVNLPS 164

Chamaeleo_calyptratus_Acr SAQANLMGLYPNS---APEIGWNPIPVHTVPVKYDKLLKPPLRSCLQYQQLMEETVNLSS 164

Chamaeleo_dilepis_Acr SAQANLMGLYPNS---APEIGWSPIPVHTVPVKYDKLLKPPLRSCLQYQRLMEETVNLPS 164

Chamaeleo_laevigatus_Acr SAQANLMGLYPNS---APEIGWSPIPVHTVPVKYDKLLKPPLRSCLQYQRLMEETVNLPS 164

Chamaeleo_gracilis_Acr SAQANLMGLYPNS---APEIGWSPIPVHTVPVKYDKLLKPPLRSCLQYQRLMEETVNLPS 164

Trioceros_affinis_Acr SAQANLMGLYPNS---APEIGWSPIPVHTVPVKYDKLLKPPLRSCLRYQQLMEETVNLPS 164

Trioceros_harennae_Acr SAQANLMGLYPNS---APEIGWSPIPVHTVPVKYDKXXXXXXXXXXXXXXXXXXXXXXXX 164

Furcifer_pardalis_Acr SAQANLMGLYPNS---VPEIGWSPIPVHTVPVKYDKLLKPPLRSCLRYQQLMEETVNLPS 161

Trioceros_balebicornutus_Acr SAQANLMGLYPNS---APEIGWSPIPVHTVPVKYDKXXXXXXXXXXXXXXXXXXXXXXXX 164

Anolis_apletophallus_Pleu SAQANLMGLYSNS---DPEIGWSPVPVHTVPIKYDKLLKSPTRTCQRYQHLMEETINLPS 164

Anolis_tropidonotus_Pleu SAQANLMGLYSNS---DPEIGWSPVPVHTVPIKYDKLLKSPTRTCQRYQHLMEETINLPS 164

Anolis_carolinensis_Pleu SAQANLMGLYSNL---DPEIGWSPVPIHTVPIKYDKLLKSPTRTCQRYQHLMEETINLPS 164

Anolis_sagrei_ordinatus_Pleu SAQANLMGLYSNS---DPETGWSPVPVHTVPIKYDKLLKSPTRTCQRYQHLMEETINLPS 164

Ctenosaura_bakeri_Pleu SAQANLMGLFPNP---DPETGWSPVPIHTVPVKYDKLLKPPTRTCQRYQQLMEETINLPS 164

Iguana_delicatissima_Pleu SAQANLMGLFPNP---DPETGWSPVPIHTVPVKYDKLLKPPTRTCQRYQQLMEETINLPS 164

Cyclura_pinguis_Pleu SAQANLMGLFPNP---DPETGWSPVPIHTVPVKYDKLLKPPTRTCQRYQRLMEETINLPS 164

Laemanctus_serratus_Pleu SAQANLMGLYPNS---DPEIGWRPVPIHTVPIKYDKLLKSPTRSCQRYQQLMEKTINLPS 164

Phrynosoma_blainvillii_Pleu SAQANLMGLYPHS---DPETGWSPVPIHTVPINYDKLLKSPTRTCHRYQQLMEETVNLPS 164

Sceloporus_chrysostictus_Pleu SAQANLMGLYPNS---DPETGWSPVPIHTVPIKYDKLLKSPTRTCHRYQQLMEETINLPS 164

Sceloporus_occidentalis_Pleu SAQANLMGLYPNL---DPETGWSPVPIHTVPIKYDKLLKSPTRTCHRYQQLMEETINLPS 164

Sceloporus_tristichus_Pleu SAQANLMGLYPNL---DPETGWSPVPIHTVPIKYDKLLKSPTRTCHRYQQLMEETINLPS 164

Sceloporus_undulatus_Pleu SAQANLMGLYPNL---DPETGWSPVPIHTVPIKYDKLLKSPTRTCHRYQQLMEETINLPS 164

Urosaurus_nigricaudus_Pleu SAQANLMGLYPNS---DPETGWSPVPIHTVPIKYDKLLKSPTRTCHRYQQLMEETINLPS 164

Gambelia_wislizenii_Pleu SAQANLMGLYPNS---DPETGWRPVPIHTVPIKYDKLLKSPTRTCQRYQQLMEETINLPS 164

Canis_lupus_dingo_ACP4 SAQANLAGLFPEAAPGRPEAAWRPIPVHTVPVTEDKLLRFPTRSCPRYHELLREATEATE 175

Vulpes_vulpes_ACP4 SAQANLAGLFPEAAPGRPEAAWRPIPVHTVPVTEDKLLRFPTRSCPRYHELLREATEATE 175

Meles_meles_ACP4 SAQANLAGLFPEAAPGRPEAAWRPIPVHTVPVTEDKLLRFPMRSCPRYHELLREATEAAE 172

Mustela_putorius_ACP4 SAQANLAGLFPEAAPGRPEAAWRPIPVHTVPITEDKLLRFPMRSCPRYHELLREATEAAE 143

Ursus_arctos_ACP4 SAQANLAGLFPEAAPGRSEAAWRPIPVHTVPVTEDKLLRFPMRSCPRYHELLREATEAAE 172

Phacochoerus_africanus_ACP4 SAQANLAGLFPEASPGRSEATWQPIPVHTVPVTEDKLLRFPTRSCPRYRELLREATEAAE 173

Phyllostomus_hastatus_ACP4 SAQANLAGLFPEAAPGRPEAAWRPIPVHTVPATEDKLLRFPTRSCPRYRELLREATEAAE 172

Homo_sapiens_ACP4 SAQANLAGLFPEAAPGSPEARWRPIPVHTVPVAEDKLLRFPMRSCPRYHELLREATEAAE 174

Saimiri_boliviensis_ACP4 SAQANLAGLFPEAAPGSPEAHWRPIPVHTVPVAEDKLLRFPMRSCPRYHELLREATEATK 173

Erinaceus_europaeus_ACP4 SAQANLAGLFPESAPGRPEAAWRPIPVHTVPVAEDKLLRFPTRSCPRYRELLREATEATE 172

Echinops_telfairi_ACP4 SALANLAGLYPEAAPGTPESSWRPIPVHTVPVTEDKLLRFPARSCPRYRELLREATEATE 172

Suncus_etruscus_ACP4 SAQANLAGLFPESGPGRPESAWRPIPVHTVPASEDKLLRFPTRNCPRYRELLREATDASD 173

** *** **:.. . * *:*:*:** **

Acanthocercus_cyanogaster_Acr YQAKMETWKGFITKMANYTGLKPEQLTLRVLWKVQDSLFCQKMHNLTLPSWATPRVLKML 224

Acanthocercus_minutus_Acr YQVKMETWKGFITKMANYTGLKPEQLTLRVLWKVQDSLFCQKMHNLTLPSWATPRVLKML 224

Xenagama_zonura_Acr YQVKMETWKGFITKMANYTGLKPEQLTLRVLWKVQDSLFCQKMHNLTLPSWATPRVLKML 224

Laudakia_wui_Acr YQAKMETWKGFITKMANYTGLKPEQLTLRALWKVQDSLFCQKMHNLTLPSWATPRVLKTL 224

Phrynocephalus_forsythii_Acr YQAKMESWKGFITEMANYTGLKPEQLTLRALWKVQDSLFCQKIHNLTLPSWAIPWVLKML 224

Phrynocephalus_guinanensis_Acr YQAKMESWKGFITEMANYTGLKPEQLTLRALWKVKDSLFCQKIHNLTLPSWAIPRVLKML 221

Phrynocephalus_vlangalii_Acr YQAKMESWKGFITEMANYTGLKPEQLTLRALWKVQDSLFCQKIHNLTLPSWATPRVLKML 224

Phrynocephalus_versicolor_Acr YQAKMESWKGFITEMANYTGLKPEQLTLRALWKVQDSLFCQKMHNLTLPSWATPRVWKML 224

Agama_doriae_Acr YQAKMETWKGFITKMANYTGLKPEQLTLRALWKVQDSLFCQRMHNLTMPGWATPRVLKML 224

Intellagama_lesueurii_Acr YQAKMETWKGFITEMANCTGLQPEQLTLRALWKVQDSLFCQKMHNLTLPSWATPQVLKTL 224

Pogona_vitticeps_Acr YQAKMETWKGFITEMANCTGLQPEQMTLRALWKVQDSLFCQKMHNLTLPSWATPQVLKTL 221

Bradypodion_pumilum_Acr YQAKMETWKGFIKEMANYTGLKPEQLTLRVLWRVHDSLFCQKMHNLTLPSWATPRVMKML 226

Bradypodion_ventrale_Acr YQAKMETWKGFIKEMANYTGLKPEQLTLRVLWRVHDSLFCQKMHNLTLPSWATPRVMKML 224

Chamaeleo_calyptratus_Acr YQAKMETWKGFIKEMANYTGLKPEQLTLRVLWRVHDSLFCQKMHNLTLPSWATPRVLKML 224

Chamaeleo_dilepis_Acr YQAKMETWKGFVKEMANYTGLKPEQLTLRVLWRVHDSLFCQKMHNLTLPSWATPRVLKML 224

Chamaeleo_laevigatus_Acr YQAKMETWKGFIKEMANYTGLKPEQLTLRVLWRVHDSLFCQKMHNLTLPSWATPRVLKML 224

Chamaeleo_gracilis_Acr YQAKMETWKGFIKEMANYTGLKPEQLTLRVLWRVHDSLFCQKMHNLTLPSWATPQVLKML 224

Trioceros_affinis_Acr YQAKMETWKGFIKEMANYTGLKPEQLTLRVLWRVHDSLFCQKMHNLTLPSWATPRVLKML 224

Trioceros_harennae_Acr XXXXXXXXXGFIKEMANYTGLKPEQLTLRVLWRVHDSLFCQKMHNLTLPSWATPRVLKML 224

Furcifer_pardalis_Acr YQAKMETWKGFIKEMANYTGLKPEQLSLRVLWRVHDSLFCQKMHNLTLPSWATPRVLKML 221

Trioceros_balebicornutus_Acr XXXXXXXXXGFIKEMANYTGLKPEQLTLRVLWRVHDSLFCQKMHNLTLPSWATPRVLKML 224

Anolis_apletophallus_Pleu YQAKMEGWKGFIREMANYTGLKLEQLTLRGLWRVHDSLFCQKIHNLTLPSWATPRVLRTL 224

Anolis_tropidonotus_Pleu YQAKMEGWKGFIKEMANYTGLKLEQLTLRGLWRVHDSLFCQKIHNLTLPGWATPRVLRTL 224

Anolis_carolinensis_Pleu YQAKMEGWKGFIREMANYTGLKMEQLTLRGLWRVHDSLFCQKVHNLTLPGWATSRVLRTL 224

Anolis_sagrei_ordinatus_Pleu YQAKMEGWKGFIREMANYTGLKLEQLTLRGLWRVHDSLFCQKIHNLTLPSWATPRVLRTL 224

Ctenosaura_bakeri_Pleu YQAKMEGWKGFIREMANYTGLKLEQLTLRGLWRVHDSLFCQKIHNLTLPSWATPQVLTTL 224

Iguana_delicatissima_Pleu YQAKMEGWKGFIREMANYTGLKLEQLTLRGLWKVHDSLFCQKIHNLTLPSWATPQVLTTL 224

Cyclura_pinguis_Pleu YQAKMEGWKGFIRKMANYTGLKLEQLTLRGLWRVHDSLLCQKIHNLTLPSWATPQVLTTL 224

Laemanctus_serratus_Pleu YQAKMEGWKGFIREMANYTGLKLEQLTLRGLWRVHDSLFCQKIHNLTLPSWATPQVLTTL 224

Phrynosoma_blainvillii_Pleu YQAKMKGWKGFTGEMSNYTGLKLEQLTLKGLWRVHDSLFCQKTHNLTLPSWATPQVLTTL 224

Sceloporus_chrysostictus_Pleu YQAKMKGWKGFIKDMSNYTGLKLEQLTLRGLWRVHDSLFCQKMHNLTLPSWATPQVLTTL 224

Sceloporus_occidentalis_Pleu YQAKMKGWKGFIKEMSNYTGLKLEQLTLRGLWRVHDSLFCQKMHNLTLPSWATPQVLTTL 224

Sceloporus_tristichus_Pleu YQAKMKGWKGFIKEMSNYTGLKLEQLTLRGLWRVHDSLFCQKMHNLTLPSWATPQVLTTL 224

Sceloporus_undulatus_Pleu YQAKMKGWKGFIKEMSNYTGLKLEQLTLRGLWRVHDSLFCQKMHNLTLPSWATPQVLTTL 224

Urosaurus_nigricaudus_Pleu YQAKMEGWKGFIKEMSNYTGLKLEQLTLRGLWRVHDSLFCQKMHNLTLPSWATPQVLTTL 224

Gambelia_wislizenii_Pleu YQAKIEGWKGFIREIANYTGLKLEQLTLRGLWRVHDSLFCQKIHNLTLPSWATPQVLTML 224

Canis_lupus_dingo_ACP4 YQTALEGWTDFLTHLENYTGLSLVGEPLRRAWKVLDTLMCQQAHGLPLPSWASPNVLRTL 235

Vulpes_vulpes_ACP4 YQTALEGWTDFLTHLENYTGLSLVGEPLRRAWKVLDTLMCQQAHGLPLPSWASPNVLRTL 235

Meles_meles_ACP4 YQTALEGWTDFLTHLENVTGLLLVGEPLRRAWKVLDTLMCQQAHGLPLPSWASPKVLRTL 232

Mustela_putorius_ACP4 YQTALEGWTDFLTHLENVTGLLLVGEPLRRAWKVLDTLICQQAHGLPLPSWASPKVLRTL 203

Ursus_arctos_ACP4 YQTALEGWTDFLTRLENYTGLSLVGEPLRRAWKVLDTLMCQQAHGLPLPSWASPNVLRTL 232

Phacochoerus_africanus_ACP4 YKSALEGWTDFLTRLENFTGLSLVGEPLRRAWKVLDTLICQQAHGLPLPSWASPDVLQTL 233

Phyllostomus_hastatus_ACP4 YQMALEGWTDFLTHLENCTGLSLVGEPLRRAWKVLDTLMCQRAHGLSLPTWASPDVLRTL 232

Homo_sapiens_ACP4 YQEALEGWTGFLSRLENFTGLSLVGEPLRRAWKVLDTLMCQQAHGLPLPAWASPDVLRTL 234

Saimiri_boliviensis_ACP4 YQEALEGWTGFLTRLENFTGLSLVGEPLRRAWKVLDTLLCQQAHGLPLPAWASPEVLQTL 233

Erinaceus_europaeus_ACP4 YQTALEGWTDFLIRLENFTGLSLVGEPLRKAWKVLDTLICQEAHGLPRPAWASPDVLRTL 232

Echinops_telfairi_ACP4 YQAALEGWTDFLIRLGNSTGLSLVGEPLRRAWKVLDTLICQKAHSLPLPAWASPDVLRTL 232

Suncus_etruscus_ACP4 YQTALDGWTDFLTRLENFTGLHLVGEPLRKAWKVLDTLICQQAHGLSLPPWASLDVMRTL 233

.* : * *** .*: *:* *:*:**. *.*. * ** * *

Acanthocercus_cyanogaster_Acr SEIEAFNVEAHVGMHSSQEKARLTGGLLLGAILSNFSKTVCRDLPLKMIMYSAHDSTLIA 284

Acanthocercus_minutus_Acr SEIEAFNVEAHVGMHSSQEKARLTGGLLLGAILSNFSKTVCRNLPLKMIMYSAHDSTLIA 284

Xenagama_zonura_Acr SEIEAFNVEAHVGMHSRQEKARLTGGLLLGAILSNFSKTVCRNLPLKMIMYSAHDSTLIA 284

Laudakia_wui_Acr SEIEAFNIEAHVGMHSSQEKARLTGGLLLGAILSNFSKTVCRDLPLKMIMYSAHDSTLIA 284

Phrynocephalus_forsythii_Acr SEIEAFNIEAHVGMHSSQEKARLTGGLLLGAILSNFSKTVCRDLPLKMIMYSAHDSTLIA 284

Phrynocephalus_guinanensis_Acr SEIEAFNIEAHVGMHSSQEKARLTGGLLLGAILSNFSKTVCRDLPLKMIMYSAHDSTLIA 281

Phrynocephalus_vlangalii_Acr SEIEAFNIEAHVGMHSSQEKARLTGGLLLGAILSNFSKTVCRDLPLKMIMYSAHDSTLIA 284

Phrynocephalus_versicolor_Acr SEIEAFNIEAHVGMHSSQEKARLTGGLLLGAILSNFSKTVCRDLPLKMIMYSAHDSTLIA 284

Agama_doriae_Acr SEIEAFNVEAHVGMHGSREKARLTGGLLLGAVLSNFSKTVCRDLPLKMIMYSAHDSTLIA 284

Intellagama_lesueurii_Acr SEIEAFNVEAHVGMHSSQEKARLTGGLLLGAILSNFSKTVCRDLPLKMIMYSAHDSTLIA 284

Pogona_vitticeps_Acr SEIEAFNVDAHVGMHSRQEKARLTGGLLLGAILSNFSKTVCRDLPLKMIMYSAHDSTLIA 281

Bradypodion_pumilum_Acr SEIEAFNVEAHMGLHSSQAKVRLAGGLLLGAILSNFSKTVCQDLPLKMIMYSAHDSTLIA 286

Bradypodion_ventrale_Acr SEIEAFNVEAHMGMHSSQAKVRLAGGLLLGAILSNFSKTVCQDLPLKMIMYSAHDSTLIA 284

Chamaeleo_calyptratus_Acr SEIEAFNVEAHVGMHSSQAKVRLAGGLLLGAILSNFSKTVCQHLPLKMIMYSAHDSTLIA 284

Chamaeleo_dilepis_Acr SEIEAFNVEAHVGMHSSQAKVRLAGGLLLGAILSNFSKTVCQDLPLKMIMYSAHDSTLIA 284

Chamaeleo_laevigatus_Acr SEIEAFNVEAHVGMHSSQAKVRLAGGLLLGAILSNFSKTVCQDLPLKMIMYSAHDSTLIA 284

Chamaeleo_gracilis_Acr SQIEAFNVEAHVGMHSSQAKVRLAGGLLLGAILSNFSKTVCQDLPLKMIMYSAHDSTLIA 284

Trioceros_affinis_Acr SEIEAFNVEAHVGMHSSQAKVRLAGGLLLGAILSNFSKTVCQDLPLKMIMYSAHDSTLIA 284

Trioceros_harennae_Acr SEIEAFNVEAHVGMHSSQAKVRLAGGLLLGAILSNFSKTVCQDLPLKMIMYSAHDSTLIA 284

Furcifer_pardalis_Acr SEIEAFNVEAHVGMHSSQAKVRLAGGLLLGAILSNFSKTVCQDLPLKMIMYSAHDSTLIA 281

Trioceros_balebicornutus_Acr SEIEAFNVEAHVGMHSSQAKVRLAGGLLLGAILSNFSKTVCQDLPLKMIMYSAHDSTLIA 284

Anolis_apletophallus_Pleu AEVEAFNVEAHVGMHASQEKVRFTGGLLLDAILTNFSKIACRDLPLKMIMFSAHDSTLIA 284

Anolis_tropidonotus_Pleu AEIEAFNVEAHVGMHASQEKVRFTGGLLLDAILSNFSKIVCRDLPLKMIMFSAHDSTLIA 284

Anolis_carolinensis_Pleu AEIEAFNVEAHVGMHASQEKVRFTGGLLLDAILSNFSKIVCRDLPLKMIMYSAHDSTLIA 284

Anolis_sagrei_ordinatus_Pleu AEIEAFNVEAHVGMHASQEKVRFTGGLLLDAILSNFSKIACRDLPLKMIMYSAHDSTLIA 284

Ctenosaura_bakeri_Pleu TEIEAFNVEAHVGMHASQEKARFTGGLLLGAILSNFSKIMCRDLPLKMIMYSAHDSTLIA 284

Iguana_delicatissima_Pleu SEIEAFNVEAHVGMHASQEKARFSGGLLLGAILSNFSKIMCRDLPLKMIMYSAHDSTLIA 284

Cyclura_pinguis_Pleu TEIEAFNVEAHVGMHASQEKARFTGGLLLGAILSNFSKIMCQDLPLKMITYSAHDSTLIA 284

Laemanctus_serratus_Pleu AEIEAFNVEAHVGMYASQEKARFTGGLLLDAILSNFSKIVCRDLPLKMIMYSAHDSTLIA 284

Phrynosoma_blainvillii_Pleu AEIETFNVDAHVGMHGTQEKARFTGGLLLGAILSNFSKIVCQDLPLKMVMYSAHDSTLIA 284

Sceloporus_chrysostictus_Pleu AEIEIFNVEAHVGMYATREKARFTGGLLLGAILSNFSKIVCRDLPQKMVMYSAHDSTLIA 284

Sceloporus_occidentalis_Pleu AEIEIFNVEAHVGMYATQEKARFTGGLLLGAILSNFSKIVCRDLPLKMVMYSAHDSTLIA 284

Sceloporus_tristichus_Pleu AEIEIFNVEAHVGMYATQEKARFTGGLLLGAILSNFSKIVCRDLPLKMVMYSAHDSTLIA 284

Sceloporus_undulatus_Pleu AEIEIFNVEAHVGMYATQEKARFTGGLLLGAILSNFSKIVCRDLPLKMVMYSAHDSTLIA 284

Urosaurus_nigricaudus_Pleu AEIEIFNVEAHVGMYATREKARFTGGLLLGAILSNFSKIVCRDMPLKMVMYSAHDSTLIA 284

Gambelia_wislizenii_Pleu AEIEAFNVEAHVGMHAHQEKARFTGGLLLDAILSNFSKIVRRELPLKMIMYSAHDSTLIA 284

Canis_lupus_dingo_ACP4 AQISALDIGAHVGPPRAAEKAQLTGGILLDAILANFSRVQRLALPLKMVMYSAHDSTLLA 295

Vulpes_vulpes_ACP4 AQISALDIGAHVGPPRAAEKAQLTGGILLDAILANFSRVQHLALPLKMVMYSAHDSTLLA 295

Meles_meles_ACP4 AQISALDIGAHVGPPRAAEKAQLTGGILLDAILANFSRVQHLGLPLKMVMYSAHDSTLLA 292

Mustela_putorius_ACP4 AQISALDIGAHVGPPRAAEKAQLTGGILLDAILANFSRAQHLGLPLKMVMYSAHDSTLLA 263

Ursus_arctos_ACP4 AQISALDIGAHVGPPRAAEKAQLTGGILLDAILGNFSRVQRLGLPLKMVMYSAHDSTLLA 292

Phacochoerus_africanus_ACP4 AQISALDIGAHVGPPRAAEKAQLTGGILLDAILANFSRVQHLGLPLKMVMYSAHDSTLLA 293

Phyllostomus_hastatus_ACP4 ARISALDIGAHVGPPRAAEKAQLTGGILLDAILANFSRVQRLGLPLKLVMYSAHDSTLLA 292

Homo_sapiens_ACP4 AQISALDIGAHVGPPRAAEKAQLTGGILLNAILANFSRVQRLGLPLKMVMYSAHDSTLLA 294

Saimiri_boliviensis_ACP4 AQISALDIGAHVGPPRAAEKAQLTGGILLNAILANFSRVQRLGLPLKMVMYSAHDSTLLA 293

Erinaceus_europaeus_ACP4 AQISALDIGAHVGPPHAAEKAQLSGGILLDAILTNFSRVQRLGLPLKMVMYSAHDSTLLA 292

Echinops_telfairi_ACP4 TQISALDIGAHVGPPRAAEKAQLTGGILLDAILANFSRTQHLGLPLKMVMYSAHDSTLLA 292

Suncus_etruscus_ACP4 AQISALDIKAHVGPPKAAEKAQLSGGILLDAILSNFSRTQRLGLPLKMVMYSAHDSTLLA 293

:.:. ::: **:* *.:::**:**.*:* ***: :* *:: :*******:*

Acanthocercus_cyanogaster_Acr LHGALGVYNGHPPPYAACHGFEFYQ------ESNNSFSVTMFYRNASNRQPYTLILPGCP 338

Acanthocercus_minutus_Acr LHGALGVYNGHPPPYAACHGFEFYQ------ESNNSFSVAMFYRNASNQQPYTLTLPGCP 338

Xenagama_zonura_Acr LHGALGVYNGHPPPYAACHGFEFYQ------ESNNSFSVAMFYRNASNQQPYTLTLPGCP 338

Laudakia_wui_Acr LQGALGVYNGHPPPYAACHGFEFYQ------ESNNSFSVAMFYRNTSDRQPHTLTLPGCP 338

Phrynocephalus_forsythii_Acr LHGALGVYNGHLPPYAACHGFEFYQ------ESNNSFSVAMFFRNTSDQQPHTLTLPGCP 338

Phrynocephalus_guinanensis_Acr LHGALGIYNGHPPPYAACHGFEFYQ------ESNNSFSVAMFYRNTSDLQPHTLTLPGCP 335

Phrynocephalus_vlangalii_Acr LHGALGIYNGHPPPYAACHGFEFYQ------ESNNSFSVAMFYRNTSDQQPHTLTLPGCP 338

Phrynocephalus_versicolor_Acr LHGALGIYNGHLPPYAACHGFEFYQ------ESNNSFSVAMFYRNTSDRQPHTLTLPGCP 338

Agama_doriae_Acr LHGALGVYNGHPPPYAACHGFEFYQ------ESNNSFSVAMFYRNASDQQPYALNLPGCS 338

Intellagama_lesueurii_Acr LQGALGVYNGHPPPYAACHGFEFYQ------ESNNSFSVAMFYRNTSDQQPHTLTLPGCP 338

Pogona_vitticeps_Acr LQGALGVYNGHPPPYAACHGFEFYQ------ETNNSFTVAMFYRNTSDQQPHTLTLPGCP 335

Bradypodion_pumilum_Acr LQGAMGVYNGHPPPYAACHGFEFYQ------ESDKSFTVAMFYRNTSKGQPHVLTLPGCP 340

Bradypodion_ventrale_Acr LQGAMGVYNGHPPPYAACYGFEFYQ------ESDKSFSVAMFYRNTSKGQPHVLTLPGCP 338

Chamaeleo_calyptratus_Acr LQGAMGVYNGHPPPYAACHGFEFYQ------ESDKSFSVVMFYRNTSKGQPHMLTLPGCP 338

Chamaeleo_dilepis_Acr LQGAMGVYNGHPPPYAACHGFEFYQ------ESDKSFSVVMFYRNTSKGQPHMLTLPGCP 338

Chamaeleo_laevigatus_Acr LQGAMGIYNGHPPPYTACHGFEFYQ------ESDKSFSVVMFYRNTSKGQPHMLTFPGCP 338

Chamaeleo_gracilis_Acr LQGAMGVYNGHPPPYAACHGFEFYQ------ESDKSFSVVMFYRNTSNGQPHMLTLPGCP 338

Trioceros_affinis_Acr LQGAMGVYNGHPPPYAACHGFEFYQ------ESDKSFSVAMFYRNTSKGQPHMLTLPGCP 338

Trioceros_harennae_Acr LQGAMGVYNGHPPPYAACHGFEFYQ------ESDKSFSVAMFYRNTSKGQPHMLTLPGCP 338

Furcifer_pardalis_Acr LQGAMGVYNGHPPPYAACHGFEFYQ------ESDKSFTVAMFYRNTSKGQPHMLTLPGCP 335

Trioceros_balebicornutus_Acr LQGAMGVYNGHPPPYAACHGFEFYQ------ESDKSFSVAMFYRNTSKGQPHMLTLPGCP 338

Anolis_apletophallus_Pleu LHGALGIYNGHPPPYAACHGFEFYQ------ETNNSFSIGMFYRNTSDQPPHVVALPGCS 338

Anolis_tropidonotus_Pleu LHGALGIYNGHPPPYAACHGFEFYQ------ETNNSFSIGMFYRNTSDQPPHVVALPGCS 338

Anolis_carolinensis_Pleu LHGALGIYNGHPPPYAACHGFEFYQ------ETNNSFSIGMFYRNTSDQPPYVVTLPGCS 338

Anolis_sagrei_ordinatus_Pleu LHGALGIYNGHPPPYAACHGFEFYQ------EANNSFSIGMFYRNMSNQPPHEVALPGCS 338

Ctenosaura_bakeri_Pleu LHGALGIYNGHPPPYAACHGFEFYK------EIDNSFSVGMFYRNTSDQPPHVLALPGCS 338

Iguana_delicatissima_Pleu LHGALGIYNGHPPPYAACHGFEFYE------EINNSFSVGMFYRNTSDQPPHVLALPGCS 338

Cyclura_pinguis_Pleu LHGALGIYNGHPPPYAACHGFEFYE------EINNSFSVGMFYRNTSDQPPHVLALPGCS 338

Laemanctus_serratus_Pleu LHGALGIYNRHPPPYAACHGFEFYQ------EVNNSFNVGMFYRNTSDQPPHVLSLPGCS 338

Phrynosoma_blainvillii_Pleu LHGALGIYNGHPPPYAACHGFEFYQ------ESNNSFSVAMFYRNTSDQPPHILALPGCS 338

Sceloporus_chrysostictus_Pleu LHGALGIYNGHPPPYAACHGFEFYQ------ERNNSFSVAMFYRNTSDQPPHVLALPGCS 338

Sceloporus_occidentalis_Pleu LHGALGLYNGHLPPYAACHGFEFYQ------ESNNSFSVAMFYRNTSDQPPHVLALPGCS 338

Sceloporus_tristichus_Pleu LHGALGLYNGHPPPYAACHGFEFYQ------ESNNSFSVAMFYRNTSDQPPHVLALPGCS 338

Sceloporus_undulatus_Pleu LHGALGLYNGHPPPYAACHGFEFYQ------ESNNSFSVAMFYRNTSDQPPHVLALPGCS 338

Urosaurus_nigricaudus_Pleu LHGALGIYNGHPPPYAACHGFEFYQ------ESNNSFSVAMFYRNTSDQPPHVLALPGCS 338

Gambelia_wislizenii_Pleu LHGALGIYNGHLPPYAACHGFEFYQ------EINNSFSVGMFYRNRSDQPPHVLDLPGCS 338

Canis_lupus_dingo_ACP4 LQGALGLYDGHTPPYAACLGFEFRRRLGDPDEDAGNITISLFYRNDSTGLPLPLSLPGCP 355

Vulpes_vulpes_ACP4 LQGALGLYDGHTPPYAACLGFEFRRRLGDPDEDAGNITISLFYRNDSTGLPLPLSLPGCP 355

Meles_meles_ACP4 LQGALGLYDGHTPPYAACLGFEFRKRVGDPDEDAGNVTISLFYRNDSTGLPLPLSLPGCP 352

Mustela_putorius_ACP4 LQGALGLYDGHTPPYAACLGFEFRRRLGDPDEDAGNVTISLFYRNDSTGLPLTLSLPGCP 323

Ursus_arctos_ACP4 LQGALGLYDGHTPPYAACLGFEFRRRLGDPDEDAGNVTISLFYRNDSTGLPLPLSLPGCP 352

Phacochoerus_africanus_ACP4 LQGALGLYDGHTPPYAACLGFEFRRHLGDLDDNRGNVTISLFYRNDSTGLPMTLKLPGCP 353

Phyllostomus_hastatus_ACP4 LQGALGLYDGHTPPYAACLGFEFRRHLGDQDGDEGNVTVSLFYRNDSAGAPLSLGLPGCP 352

Homo_sapiens_ACP4 LQGALGLYDGHTPPYAACLGFEFRKHLGNPAKDGGNVTVSLFYRNDSAHLPLPLSLPGCP 354

Saimiri_boliviensis_ACP4 LQGALGLYDGHTPPYAACLGFEFRRRLGDPDEDGGNVTISLFYRNDSAHRPLPLSLPGCP 353

Erinaceus_europaeus_ACP4 LQGALGLYDSHMPPYAACLGFEFRRRQGDLGDRGGNVSVSLFYRNDSAGRPLPLSLPGCP 352

Echinops_telfairi_ACP4 LQGALGLYDGHTPPYAACLGFEFQRHRGDQDEEEGNVTISLFYRNDSSRPPLTLRLPGCP 352

Suncus_etruscus_ACP4 LQGALGLYDGHTPPYAACLGFEFRRRLEDPDEDVGNVTVSLFYRNDSTRLPRSLSLPGCP 353

*:**:*:*: * ***:** **** . ...: :*:** * * : :***.

Acanthocercus_cyanogaster_Acr TPCPLPLFTKLTHAVIPQDWEAECQNPRRST-GNQGAFTHAHPAT--------------- 382

Acanthocercus_minutus_Acr TPCPLPLFTKLTHAVIPQDWEAECQNPRRST-GNQGAFKHLPPAT--------------- 382

Xenagama_zonura_Acr TPCPLLLFTKLTHAVIPQDWEAECQNPRRST-GNQGAFKHLPPAT--------------- 382

Laudakia_wui_Acr TPCPLLLFTKLTHAVVPQDWEAECQNPQRST-GNQGAFKHTNPAT--------------- 382

Phrynocephalus_forsythii_Acr TPCPLPLFTKLTHAVVPQDWEAECQNPQRSP-GNQGAFKHTYPAT--------------- 382

Phrynocephalus_guinanensis_Acr TPCPLPLFTKLTHAVVPQDWEAECQNPQRSP-GNQGAFQHTYPAT--------------- 379

Phrynocephalus_vlangalii_Acr TPCPLPLFTKLTHAVVPQDWEAECQNPQRSP-GKQGAFQHTYPAT--------------- 382

Phrynocephalus_versicolor_Acr TPCPLPLFTKLTHAVVPQDWEAECQNPQRSP-GNQGAFKYKYPAT--------------- 382

Agama_doriae_Acr TPCPLPLFTKLTHAVIPQDWEAECQHPRRST-GNPGAFKHARPAT--------------- 382

Intellagama_lesueurii_Acr TPCPLPLFTKLTRAVVPPDWDAECQNPQSST-GNESLHTHTHILRP-------------- 383

Pogona_vitticeps_Acr THCPLPLFTKLTRAVVPQDWDAECQNPQNRT-GNESLHTHTHTVTLVLKVE--------- 385

Bradypodion_pumilum_Acr TPCPLALFTNLTHAVVPQDWDAECQTPQRST-GN-KATTSAQF-FLCYIL---------- 387

Bradypodion_ventrale_Acr TPCPLALFTNLTRAAVPQDWDAECQTPQIST-GNKATTSAVLL-VLYSIP-NICNPHGNK 395

Chamaeleo_calyptratus_Acr TPCPLARFTNLIRAXVPQDWDAECQTTQRST-GK-KATTSIQPFFLCCIP-NIGNPHGDQ 395

Chamaeleo_dilepis_Acr TPCPLALFTNLIRAVVPQDWDAECQTTQRST-GN-KATTSIQPSFLCCIP-NIGNPHGAI 395

Chamaeleo_laevigatus_Acr TPCPLALFTNLIRAVVPQDWDAECQTTQRST-GNKKATASI------------------- 378

Chamaeleo_gracilis_Acr TPCPLALFTNLIRAVVPQDWDAECQTTQ-ST-GNKKAATSIQPFFLCCIP-NIGNPHGD- 394

Trioceros_affinis_Acr APCPLVLFTNLTRAVVPQDWDAECQTPQRST-GNKKAATSTQP-FLCCIL---------- 386

Trioceros_harennae_Acr APCPLALFTNLTHAVVPQDWDAECQTPPRST-GNKKAATSTQP-FLCCIL---------- 386

Furcifer_pardalis_Acr TPCPLVLFTNLTRAVVPQDWDAECQTPQRST-GNKKATTSTQPYFLCCILFCIGNPHGNQ 394

Trioceros_balebicornutus_Acr APCPLALFTNLTRAVIPQDWDAECQTPQRST-GNKKAVTSTQP-FLCSEY---------- 386

Anolis_apletophallus_Pleu TPCPLSRFIQLTRAVIPHDWETECQNPQKST-GNKSPPPPSRNMVE-SKFTSVLNTCFVG 396

Anolis_tropidonotus_Pleu TPCPLSRFIQLTRAVIPHDWETECQNPQKST-GN-------------------------- 371

Anolis_carolinensis_Pleu TPCPLPRFIQLTHTVIPHDWETECQNPQKST-GNTPPPKKKPGKC--------------- 382

Anolis_sagrei_ordinatus_Pleu TPCPLPRFIQLTRAVIPRDWETECQNPQKST-GRTVTALAVAVAL--------------- 382

Ctenosaura_bakeri_Pleu TPCPLPQFIHLTSAVVPHDWDAECQNPQKST-GNKNPHTQL------------------- 378

Iguana_delicatissima_Pleu TPCPLPQFIHLTSVVVPNDWDAECQNPQKST-GNKKTHTQP------------------- 378

Cyclura_pinguis_Pleu TPYPLPQFIHLTSVVVPHDWDAECQNLQKST-GNKNPHTQP------------------- 378

Laemanctus_serratus_Pleu TPCPLPRFIHLTRAVIPHDWDAECQNPQKST-GNKNPHSSPQ------------------ 379

Phrynosoma_blainvillii_Pleu TPCPLPRFIHLTRPVVPHDWDAECQNPRKST-GNKSPHCQPPISLSEFKFTPVLNTVFQD 397

Sceloporus_chrysostictus_Pleu TPCPLLRFIHLTRPVVPHDWDAECQNPQKST-GNKSPHCQPQICSSEFKIYTCP------ 391

Sceloporus_occidentalis_Pleu TPCPLPRFIHLTRPVVPHDWDAECQNPRKNT-GNKSPHCQL------------------- 378

Sceloporus_tristichus_Pleu TPCPLPRFIHLTRPVVPHDWDAECQNPRKST-GNKSPHCQL------------------- 378

Sceloporus_undulatus_Pleu TPCPLPRFIHLTRPVVPHDWDAECQNPRKST-GNKSPHCQL------------------- 378

Urosaurus_nigricaudus_Pleu TPCPLPRFIHLTRPVVPHDWDAECQNPQKST-GNKSPHCQPQISFSEFKIYTCP------ 391

Gambelia_wislizenii_Pleu TPCPLPQFIHLTRAVVPHDWDAECQNPQKTT-GNKSPHSNLRRLSE-LKNLHLSLTLFCR 396

Canis_lupus_dingo_ACP4 GACPLGRFRQLTAPARPPAHGVPCHGSHEPA-TPAATVVPLLAGAV-------------- 400

Vulpes_vulpes_ACP4 GACPLGRFRQLTAPARPPAHGVPCHGSHEPA-TPAATVVPLLAGAV-------------- 400

Meles_meles_ACP4 GACPLARFRQLTAPARPPAHGVPCHGSREPA-TPAATVVPLLAGAV-------------- 397

Mustela_putorius_ACP4 GACPLARFRQLTAPARPPAHGVPCHGSHEPA-TPAATVVPLLAGAV-------------- 368

Ursus_arctos_ACP4 GACPLGRFRQLTAPARPPAHGVPCHGSREPA-TPAATVVPLLSGAV-------------- 397

Phacochoerus_africanus_ACP4 APCSLGRFRQLTAPARPPAQGIPCHGSHEPA-TPAATVVPLLSGAV-------------- 398

Phyllostomus_hastatus_ACP4 VPCPLGRFRQLTAPARPPARGVPCHSSHEPA-APTAAVVPLLAGAV-------------- 397

Homo_sapiens_ACP4 APCPLGRFYQLTAPARPPAHGVSCHGPYEAA-IPPAPVVPLLAGAV-------------- 399

Saimiri_boliviensis_ACP4 VPCPLGRFHQLTAPARPPAHGVSCHGPYEAA-IPPAPVVPLLAGAV-------------- 398

Erinaceus_europaeus_ACP4 SPCPLGRFHQLTAPARPPEHGIPCHSLREPA-RHAAAAVPLLAGTV-------------- 397

Echinops_telfairi_ACP4 DPCTLGRFRQLTAPARPPAHGIPCHSLHEPAATPAATMVPLLAGAV-------------- 398

Suncus_etruscus_ACP4 GPCPLGRFHQLTAPARPPAQGIPCHGPQQPE--PPTTVVSLLAGAV-------------- 397

.* * :* * *:

Acanthocercus_cyanogaster_Acr --------------------------------------------------

Acanthocercus_minutus_Acr --------------------------------------------------

Xenagama_zonura_Acr --------------------------------------------------

Laudakia_wui_Acr --------------------------------------------------

Phrynocephalus_forsythii_Acr --------------------------------------------------

Phrynocephalus_guinanensis_Acr --------------------------------------------------

Phrynocephalus_vlangalii_Acr --------------------------------------------------

Phrynocephalus_versicolor_Acr --------------------------------------------------

Agama_doriae_Acr --------------------------------------------------

Intellagama_lesueurii_Acr --------------------------------------------------

Pogona_vitticeps_Acr --------------------------------------------------

Bradypodion_pumilum_Acr -------------------FRILAILMAISHDCPENITQ----------- 407

Bradypodion_ventrale_Acr P------------------------------------------------- 396

Chamaeleo_calyptratus_Acr P------------------------------------------------- 396

Chamaeleo_dilepis_Acr IIVWGLLHGKLG---STLNKFMPMTLLALGKNAFLADDIA---------- 432

Chamaeleo_laevigatus_Acr --------------------------------------------------

Chamaeleo_gracilis_Acr --------------------------------------------------

Trioceros_affinis_Acr -------------------FRILAILMAISH------------------- 398

Trioceros_harennae_Acr -------------------FRILAILMAISH------------------- 398

Furcifer_pardalis_Acr P------------------------------------------------- 395

Trioceros_balebicornutus_Acr ------------------WQSSWRSAM----------------------- 395

Anolis_apletophallus_Pleu RT-------------VTALAVAVALLSILLIGVGVLYCRR---------- 423

Anolis_tropidonotus_Pleu --------------------------------------------------

Anolis_carolinensis_Pleu --------------------------------------------------

Anolis_sagrei_ordinatus_Pleu -------------------------LSLMLIGVGVLYCRS---------- 397

Ctenosaura_bakeri_Pleu --------------------------------------------------

Iguana_delicatissima_Pleu --------------------------------------------------

Cyclura_pinguis_Pleu --------------------------------------------------

Laemanctus_serratus_Pleu --------------------------------------------------

Phrynosoma_blainvillii_Pleu AQ------------------------------------------------ 399

Sceloporus_chrysostictus_Pleu --------------------------------------------------

Sceloporus_occidentalis_Pleu --------------------------------------------------

Sceloporus_tristichus_Pleu --------------------------------------------------

Sceloporus_undulatus_Pleu --------------------------------------------------

Urosaurus_nigricaudus_Pleu --------------------------------------------------

Gambelia_wislizenii_Pleu THGNSSGCGSRSAEHVTNWSGDSVLQKIMRRGSHGNNEKF--------PS 438

Canis_lupus_dingo_ACP4 -----------------------AVLAALSMVLGLLAWRPSCLRALGGPV 427

Vulpes_vulpes_ACP4 -----------------------AVLAALSMVLGLLAWRPSCLRALGGPV 427

Meles_meles_ACP4 -----------------------AVLAVLSVGLGLLAWRPSCLRAWGGPV 424

Mustela_putorius_ACP4 -----------------------AVLAALSVGLGLLAWRPSCLRAWGRPV 395

Ursus_arctos_ACP4 -----------------------AVLAALSMGLGLLAWRPSCLRAWGGPV 424

Phacochoerus_africanus_ACP4 -----------------------AVLAVLSMGLGLMAWRPGCLRAWGGPV 425

Phyllostomus_hastatus_ACP4 -----------------------AVLAALSLGLGLLAWRPGCLRAWGDPV 424

Homo_sapiens_ACP4 -----------------------AVLVALSLGLGLLAWRPGCLRALGGPV 426

Saimiri_boliviensis_ACP4 -----------------------AVLVALSLGLGLLAWRPGCLRAFGGPV 425

Erinaceus_europaeus_ACP4 -----------------------AVLAALSLGLGVLAWRPGCLRAWGGPV 424

Echinops_telfairi_ACP4 -----------------------AVLAALSMGLGLLAWRPSCPGAWGGPV 425

Suncus_etruscus_ACP4 -----------------------VALALLSAGLGLLVYRPGCLRAWGGPV 424

**ACP4-AA-MAFFT-Iguania – DIVERGE-NO Significant sites**

CLUSTAL W (1.8) multiple sequence alignment (ALTER 1.3.3)

Acanthocercus_cyanogaster_Acr MRLSASLISLIIFLQAILPLIAEQERRLRFVILVYRHGDRSPLGTYPTDPHKSEAWPQGF 60

Acanthocercus_minutus_Acr MRLSASLISLMIFLQAILPLIAEQERRLRFVILVYRHGDRSPLGTYPTDPHKSEAWPQGF 60

Xenagama_zonura_Acr MRLSASLISLMIFLQAILPLIAEQERRLRFVILVYRHGDRSPLGTYPTDPHKSEAWPQGF 60

Laudakia_wui_Acr MRLSASLTSLMILLQAILPLIAEEERRLRFVILVYRHGDRSPLGTYPTDPHKSEAWPQGF 60

Phrynocephalus_forsythii_Acr MRLSASLASLMILLQAILPLIAEQERRLRFVVLLYRHGDRSPLGTYPTDPHKSDAWPQGF 60

Phrynocephalus_guinanensis_Acr MRLS---ASLMILLQAILPLIAEQERRLRFVILVYRHGDRSPLGTYPTDPHKSDAWPQGF 57

Phrynocephalus_vlangalii_Acr MRLSASLASLMILLQAILPLIAEQERRLRFVILVYRHGDRSPLGTYPTDPHKSDAWPQGF 60

Phrynocephalus_versicolor_Acr MRLSASLASLMILLQAILPLIAEQERRLRFVILVYRHGDRSPLGTYPTDPHKAEAWPQGF 60

Agama_doriae_Acr MRLSASLISLTVFLQAILPLIAEQERKLRFVILVYRHGDRSPLGTYPTDPHKAEAWPQGF 60

Intellagama_lesueurii_Acr MRLSASLASLMIILQAILPLIAEQERRLRFVILVYRHGDRSPLGTYPTDPHKSEAWPQGF 60

Pogona_vitticeps_Acr MRLS---ASLIIILQAILPLIAEQERRLRFVILVYRHGDRSPLGTYPTDPHKSEAWPQGF 57

Bradypodion_pumilum_Acr MRLSAALTSLMIFLQPILPLTAEQERTLRFIILVYRHGDRSPLGTYPTDPHKAEAWPQGF 60

Bradypodion_ventrale_Acr MRLSAALTSLMIFLQPILPLTAEQERTLRFIILVYRHGDRSPLGTYPTDPHKAEAWPQGF 60

Chamaeleo_calyptratus_Acr MRLSAALTSLTIFLQTILPLTAEQERTLRFIVLVYRHGDRSPLGTYPTDPHKAEAWPQGF 60

Chamaeleo_dilepis_Acr MRLSAVLTSLMIFLQTILPLTAEQERTLRFIVLVYRHGDRSPLGTYPTDPHKAEAWPQGF 60

Chamaeleo_laevigatus_Acr MRLSAALTSLMIFLQTILPLTAEQERTLRFIILVYRHGDRSPLGTYPTDPHKAEAWPQGF 60

Chamaeleo_gracilis_Acr MRLSAALTSLMIFLQTILPLTAEQERTLRFSVLVYRHGDRSPLSTYPTDPHKAEAWPQGF 60

Trioceros_affinis_Acr MRLSAVLTSLMIFLQPILPLTAEQERTLRFIILVYRHGDRSPLGTYPTDPHKAEAWPQGF 60

Trioceros_harennae_Acr MRLSAVLTSLMIFLQPILPLTAEQERTLRFIILVYRHGDRSPLGTYPTDPHKAEAWPQGF 60

Furcifer_pardalis_Acr MRLSAALTSLMIFL---LPLAAEQERTLRFIILVYRHGDRSPLGTYPTDPHKAEAWPQGF 57

Trioceros_balebicornutus_Acr MRLSAVLTSLMIFLQPILPLTAEQERTLRFIILVYRHGDRSPLGTYPTDPHKAEAWPQGF 60

Anolis_apletophallus_Pleu MRLSETIPPLMVFLQNILQLATGQERTLRFVTLVYRHGDRSPLGTYPTDPHKAAAWPEGF 60

Anolis_tropidonotus_Pleu MRLSETIPHLMIFLQNIFQLATGQERTLRFVTLVYRHGDRSPLGTYPTDPHKAAAWPEGF 60

Anolis_carolinensis_Pleu MRLSETLAALVVFLQNILHFTTGQERTLRFVTLVYRHGDRSPLGTYPTDPHKAAAWPEGF 60

Anolis_sagrei_ordinatus_Pleu MRLSDTLVPLMVFLQNVLHLTTGQERTLRFVTLVYRHGDRSPLGTYPTDPHKAAPWPEGF 60

Ctenosaura_bakeri_Pleu MRLSEALAPLMIFLHDILHLTTGQERTLRFVTLVYRHGDRSPLGTYPTDPHKAAAWPQGF 60

Iguana_delicatissima_Pleu MRLSEALTPLMIFLHDILHLTTGQERTLRFVTLVYRHGDRSPLGTYPTDPHKAAAWPQGF 60

Cyclura_pinguis_Pleu MRLSEALTPLMIFLHDILHLTTGQERTLRFVTLVYRHGDRSPLGTYPTDPHKAAAWPQGF 60

Laemanctus_serratus_Pleu MRLSEILTALMILLHNILHLTTGQERTLRFVTLVFRHGDRSPLGTYPTDPHKAAAWPQGF 60

Phrynosoma_blainvillii_Pleu MRVSETLAPLMIFIHNIFHLTAGQERTLRSVTLVYRHGDRSPLGTYPTDPHKATVWPQGF 60

Sceloporus_chrysostictus_Pleu MRLSGTLASLMICIHNILHFAAGQERTLRFVTLVYRHGDRSPLGTYPTDPHKAAVWPQGF 60

Sceloporus_occidentalis_Pleu MRLSETLASLMICIHNILHFTAGQERTLRFVTLVYRHGDRSPLGTYPTDPHKAAVWPQGF 60

Sceloporus_tristichus_Pleu MRLSETLASLMICIHNSLHFTAGQERTLRFVTLIYRHGDRSPLGTYPTDPHKAAVWPQGF 60

Sceloporus_undulatus_Pleu MRLSETLASLMICIHNSLHFTAGQERTLRFVTLIYRHGDRSPLGTYPTDPHKAAVWPQGF 60

Urosaurus_nigricaudus_Pleu MRLPETLASLMVFTHNILHFTAGQERTLRFVTLLYRHGDRSPLGTYPTDPHKAAVWPQGF 60

Gambelia_wislizenii_Pleu MRLSATLTPLMIFLHNIMHLTTAQERTLRFVTLVYRHGDRSPLGTYPTDPHKEAAWPQGF 60

**:. * : : : : :** ** *::********.******** **:**

Acanthocercus_cyanogaster_Acr HQL--TEAGILQQKALGQFLRERYNGFLSPSYKPQEIYVRSTDYDRTIMSAQANLMGLYP 118

Acanthocercus_minutus_Acr HQL--TEAGILQQKALGQFLRERYNGFLSPSYKPQEIYVRSTDYDRTIMSAQANLMGLYP 118

Xenagama_zonura_Acr HQL--TEAGILQQKALGQFLRERYNGFLSPSYKPQEIYIRSTDYDRTIMSAQANLMGLYP 118

Laudakia_wui_Acr HQL--TEAGVLQQKALGQFLRERYNGFLSPSYKPQEIYVRSTDYDRTIMSAQANLMGLYP 118

Phrynocephalus_forsythii_Acr HQL--TEDGVLQQKALGQFLRERYNGFLSPSYKQQEIYIRSTDYDRTIMSAQANLMGLYP 118

Phrynocephalus_guinanensis_Acr HQL--TEAGVLQQKALGQFLRERYNGFLSPSYKQQEIYVRSTDYDRTIMSAQANLMGLYP 115

Phrynocephalus_vlangalii_Acr HQL--TEAGVLQQKVLGQFLKEKYNGFLSPSYKQQEIYVRSTDYDRTIMSAQANLMGLYP 118

Phrynocephalus_versicolor_Acr HQL--TEAGVLQQKALGQFLREIYNGFLSPSYKQQEIYVRSTDYDRTIMSAQANLMGLYP 118

Agama_doriae_Acr HQL--TKAGILQQKALGQFLRERYNGFLSPSYKPQEIYVRSTDYDRTIMSAQANLMGLYP 118

Intellagama_lesueurii_Acr HQL--TKVGILQQKVLGRFLRKRYNGFLSPSYKPQEIYVRSTDYDRTIMSAQANLMGLYP 118

Pogona_vitticeps_Acr HQL--TEVGILQQKVLGRFLRKRYNGFLSPSYKPQEIYVRSTDYDRTIMSAQANLMGLYP 115

Bradypodion_pumilum_Acr QQLRGRMVGILQQKTLGRFLRERYDGFLSPSYKRQEIYIRSTDYDRTIMSAQANLMGLYP 120

Bradypodion_ventrale_Acr QQL--TEVGILQQKTLGQFLRERYDGFLSPSYKRQEIYIRSTDYDRTIMSAQANLMGLYP 118

Chamaeleo_calyptratus_Acr QQL--TEVGILQQKTLGRFLRERYDGFLSPSYKRQEIYIRSTDYDRTIMSAQANLMGLYP 118

Chamaeleo_dilepis_Acr QQL--TEVGILQQKTLGQFLRERYDGFLSPSYQRQEIYIRSTDYDRTIMSAQANLMGLYP 118

Chamaeleo_laevigatus_Acr QQL--TEVGILQQKTLGRFLRERYDGFLSPSYKRQEIYIRSTDYDRTIMSAQANLMGLYP 118

Chamaeleo_gracilis_Acr QQL--TEVGILQQKTLGRFLRERYDGFLSPSYKRQEIYIRSTDYDRTIMSAQANLMGLYP 118

Trioceros_affinis_Acr QQL--TEIGILQQKTLGRFLRERYDGFLSPSYKRQEIYIRSTDYDRTIMSAQANLMGLYP 118

Trioceros_harennae_Acr QQL--TEIGILQQKTLGRFLRERYDGFLSPSYKRQEIYIRSTDYDRTIMSAQANLMGLYP 118

Furcifer_pardalis_Acr QQL--TEVGILQHKTLGRFLRERYDGFLSPSYKRQEIYIRSTDYDRTIMSAQANLMGLYP 115

Trioceros_balebicornutus_Acr QQL--TEIGILQQKTLGRFLRERYDGFLSPSYKRQEIYIRSTDYDRTIMSAQANLMGLYP 118

Anolis_apletophallus_Pleu QQL--TKVGILQQKALGKFLREKYDGFLSAAYKPQEIYIRSTDYDRTLMSAQANLMGLYS 118

Anolis_tropidonotus_Pleu QQL--TKVGILQQKALGKFLREKYEGFLSAAYKPQEIYIRSTDYDRTLMSAQANLMGLYS 118

Anolis_carolinensis_Pleu QQL--TKVGILQQKALGKFLRQKYDGFLSSAYKPQEIYVRSTDYDRTLMSAQANLMGLYS 118

Anolis_sagrei_ordinatus_Pleu QQL--TKVGILQQKALGKFLREKYDGFLSAAYKPQEIYIRSTDYDRTLMSAQANLMGLYS 118

Ctenosaura_bakeri_Pleu QQL--TKVGILQQKALGSFLREKYDGFLSAAYKPQEIYIRSTDYDRTIMSAQANLMGLFP 118

Iguana_delicatissima_Pleu QQL--TKVGILQQKALGRFLREKYDGFLSAAYKPQEIYIRSTDYDRTIMSAQANLMGLFP 118

Cyclura_pinguis_Pleu QQL--TKVGILQQKALGRFLREKYDGFLSAAYKPQEIYIRSTDYDRTIMSAQANLMGLFP 118

Laemanctus_serratus_Pleu QQL--TKVGILQQKALGKFLREKYDGFLSAAYKPQEIYIRSTDYDRTIMSAQANLMGLYP 118

Phrynosoma_blainvillii_Pleu QQL--TKVGISQQKALGRFLREKYDGFLSATYNPQEIYIRSRDYDRTIMSAQANLMGLYP 118

Sceloporus_chrysostictus_Pleu QQL--TKVGILQQKVLGRFLREKYDGFLSAAYKPQEIYIRSTDYDRTIMSAQANLMGLYP 118

Sceloporus_occidentalis_Pleu QQL--TKVGILQQKALGRFLRKKYDGFLSAAYKPQEIYIRSTDYDRTIMSAQANLMGLYP 118

Sceloporus_tristichus_Pleu QQL--TKIGILQQKALGRFLREKYDGFLSAAYKPQEIYIRSTDYDRTIMSAQANLMGLYP 118

Sceloporus_undulatus_Pleu QQL--TKIGILQQKALGRFLREKYDGFLSAAYKPQEIYIRSTDYDRTIMSAQANLMGLYP 118

Urosaurus_nigricaudus_Pleu QQL--TKVGILQQKALGRFLREKYDGFLSATYKPQEIYIRSTDYDRTIMSAQANLMGLYP 118

Gambelia_wislizenii_Pleu QQL--TEVGILQQKALGRILREKYNGFLSAAYKPQEIYIRSTDYDRTIMSAQANLMGLYP 118

:** *: *:*.** :*:: *:****.:*: ****:** *****:**********:.

Acanthocercus_cyanogaster_Acr NPDPQTDWNPIPVHTVPVKYDKLLKPPTRTCLRYQQLMEETINLPSYQAKMETWKGFITK 178

Acanthocercus_minutus_Acr NSDPQIGWNPIPVHTVPVKYDKLLKPPTRTCLRYQQLMEETINLPSYQVKMETWKGFITK 178

Xenagama_zonura_Acr NPDPQIGWNPIPVHTVPVKYDKLLKPPTRTCLRYQQLMEETINLPSYQVKMETWKGFITK 178

Laudakia_wui_Acr NPDPHIGWSPIPVHTVPVKYDKLLKPPTRTCLRYQQLMEETINLPSYQAKMETWKGFITK 178

Phrynocephalus_forsythii_Acr NPDPQIGWSPIPVHTVPVKYDKLLKPPTRACRRYQQLMEETINLPSYQAKMESWKGFITE 178

Phrynocephalus_guinanensis_Acr NPDPQIGWNPIPVHTVPVKYDKLLKPPTRACRRYQQLMEETINLPSYQAKMESWKGFITE 175

Phrynocephalus_vlangalii_Acr NPDPQIGWSPIPVHTVPVKYDKLLKPPTRACRRYQQLMEETINLPSYQAKMESWKGFITE 178

Phrynocephalus_versicolor_Acr NPDPQIGWSPIPVHTVPVKYDKLLKPPTRTCRRYQQLMEETINLPSYQAKMESWKGFITE 178

Agama_doriae_Acr NPDPQIGWSPIPVHSVPVKYDKLLKPPTRTCLRYQQLMEETINLPSYQAKMETWKGFITK 178

Intellagama_lesueurii_Acr NSDPQTGWSPVPVHTVPVKYDKLLKPPTRTCLRYQQLMEETVNLPSYQAKMETWKGFITE 178

Pogona_vitticeps_Acr NSDPQTGWNPIPVHTVPVKYDKLLKPPTRTCLRYQQLMEETVNLPSYQAKMETWKGFITE 175

Bradypodion_pumilum_Acr NSAPGIGWSPIPVHTVPVKYDKLLKPPLRSCLRYQQLMEETVNLPSYQAKMETWKGFIKE 180

Bradypodion_ventrale_Acr NSAPGIGWSPIPVHTVPVKYDKLLKPPLRSCLRYQQLMEETVNLPSYQAKMETWKGFIKE 178

Chamaeleo_calyptratus_Acr NSAPEIGWNPIPVHTVPVKYDKLLKPPLRSCLQYQQLMEETVNLSSYQAKMETWKGFIKE 178

Chamaeleo_dilepis_Acr NSAPEIGWSPIPVHTVPVKYDKLLKPPLRSCLQYQRLMEETVNLPSYQAKMETWKGFVKE 178

Chamaeleo_laevigatus_Acr NSAPEIGWSPIPVHTVPVKYDKLLKPPLRSCLQYQRLMEETVNLPSYQAKMETWKGFIKE 178

Chamaeleo_gracilis_Acr NSAPEIGWSPIPVHTVPVKYDKLLKPPLRSCLQYQRLMEETVNLPSYQAKMETWKGFIKE 178

Trioceros_affinis_Acr NSAPEIGWSPIPVHTVPVKYDKLLKPPLRSCLRYQQLMEETVNLPSYQAKMETWKGFIKE 178

Trioceros_harennae_Acr NSAPEIGWSPIPVHTVPVKYDKXXXXXXXXXXXXXXXXXXXXXXXXXXXXXXXXXGFIKE 178

Furcifer_pardalis_Acr NSVPEIGWSPIPVHTVPVKYDKLLKPPLRSCLRYQQLMEETVNLPSYQAKMETWKGFIKE 175

Trioceros_balebicornutus_Acr NSAPEIGWSPIPVHTVPVKYDKXXXXXXXXXXXXXXXXXXXXXXXXXXXXXXXXXGFIKE 178

Anolis_apletophallus_Pleu NSDPEIGWSPVPVHTVPIKYDKLLKSPTRTCQRYQHLMEETINLPSYQAKMEGWKGFIRE 178

Anolis_tropidonotus_Pleu NSDPEIGWSPVPVHTVPIKYDKLLKSPTRTCQRYQHLMEETINLPSYQAKMEGWKGFIKE 178

Anolis_carolinensis_Pleu NLDPEIGWSPVPIHTVPIKYDKLLKSPTRTCQRYQHLMEETINLPSYQAKMEGWKGFIRE 178

Anolis_sagrei_ordinatus_Pleu NSDPETGWSPVPVHTVPIKYDKLLKSPTRTCQRYQHLMEETINLPSYQAKMEGWKGFIRE 178

Ctenosaura_bakeri_Pleu NPDPETGWSPVPIHTVPVKYDKLLKPPTRTCQRYQQLMEETINLPSYQAKMEGWKGFIRE 178

Iguana_delicatissima_Pleu NPDPETGWSPVPIHTVPVKYDKLLKPPTRTCQRYQQLMEETINLPSYQAKMEGWKGFIRE 178

Cyclura_pinguis_Pleu NPDPETGWSPVPIHTVPVKYDKLLKPPTRTCQRYQRLMEETINLPSYQAKMEGWKGFIRK 178

Laemanctus_serratus_Pleu NSDPEIGWRPVPIHTVPIKYDKLLKSPTRSCQRYQQLMEKTINLPSYQAKMEGWKGFIRE 178

Phrynosoma_blainvillii_Pleu HSDPETGWSPVPIHTVPINYDKLLKSPTRTCHRYQQLMEETVNLPSYQAKMKGWKGFTGE 178

Sceloporus_chrysostictus_Pleu NSDPETGWSPVPIHTVPIKYDKLLKSPTRTCHRYQQLMEETINLPSYQAKMKGWKGFIKD 178

Sceloporus_occidentalis_Pleu NLDPETGWSPVPIHTVPIKYDKLLKSPTRTCHRYQQLMEETINLPSYQAKMKGWKGFIKE 178

Sceloporus_tristichus_Pleu NLDPETGWSPVPIHTVPIKYDKLLKSPTRTCHRYQQLMEETINLPSYQAKMKGWKGFIKE 178

Sceloporus_undulatus_Pleu NLDPETGWSPVPIHTVPIKYDKLLKSPTRTCHRYQQLMEETINLPSYQAKMKGWKGFIKE 178

Urosaurus_nigricaudus_Pleu NSDPETGWSPVPIHTVPIKYDKLLKSPTRTCHRYQQLMEETINLPSYQAKMEGWKGFIKE 178

Gambelia_wislizenii_Pleu NSDPETGWRPVPIHTVPIKYDKLLKSPTRTCQRYQQLMEETINLPSYQAKIEGWKGFIRE 178

: * .* *:*:*:**::*** ** .

Acanthocercus_cyanogaster_Acr MANYTGLKPEQLTLRVLWKVQDSLFCQKMHNLTLPSWATPRVLKMLSEIEAFNVEAHVGM 238

Acanthocercus_minutus_Acr MANYTGLKPEQLTLRVLWKVQDSLFCQKMHNLTLPSWATPRVLKMLSEIEAFNVEAHVGM 238

Xenagama_zonura_Acr MANYTGLKPEQLTLRVLWKVQDSLFCQKMHNLTLPSWATPRVLKMLSEIEAFNVEAHVGM 238

Laudakia_wui_Acr MANYTGLKPEQLTLRALWKVQDSLFCQKMHNLTLPSWATPRVLKTLSEIEAFNIEAHVGM 238

Phrynocephalus_forsythii_Acr MANYTGLKPEQLTLRALWKVQDSLFCQKIHNLTLPSWAIPWVLKMLSEIEAFNIEAHVGM 238

Phrynocephalus_guinanensis_Acr MANYTGLKPEQLTLRALWKVKDSLFCQKIHNLTLPSWAIPRVLKMLSEIEAFNIEAHVGM 235

Phrynocephalus_vlangalii_Acr MANYTGLKPEQLTLRALWKVQDSLFCQKIHNLTLPSWATPRVLKMLSEIEAFNIEAHVGM 238

Phrynocephalus_versicolor_Acr MANYTGLKPEQLTLRALWKVQDSLFCQKMHNLTLPSWATPRVWKMLSEIEAFNIEAHVGM 238

Agama_doriae_Acr MANYTGLKPEQLTLRALWKVQDSLFCQRMHNLTMPGWATPRVLKMLSEIEAFNVEAHVGM 238

Intellagama_lesueurii_Acr MANCTGLQPEQLTLRALWKVQDSLFCQKMHNLTLPSWATPQVLKTLSEIEAFNVEAHVGM 238

Pogona_vitticeps_Acr MANCTGLQPEQMTLRALWKVQDSLFCQKMHNLTLPSWATPQVLKTLSEIEAFNVDAHVGM 235

Bradypodion_pumilum_Acr MANYTGLKPEQLTLRVLWRVHDSLFCQKMHNLTLPSWATPRVMKMLSEIEAFNVEAHMGL 240

Bradypodion_ventrale_Acr MANYTGLKPEQLTLRVLWRVHDSLFCQKMHNLTLPSWATPRVMKMLSEIEAFNVEAHMGM 238

Chamaeleo_calyptratus_Acr MANYTGLKPEQLTLRVLWRVHDSLFCQKMHNLTLPSWATPRVLKMLSEIEAFNVEAHVGM 238

Chamaeleo_dilepis_Acr MANYTGLKPEQLTLRVLWRVHDSLFCQKMHNLTLPSWATPRVLKMLSEIEAFNVEAHVGM 238

Chamaeleo_laevigatus_Acr MANYTGLKPEQLTLRVLWRVHDSLFCQKMHNLTLPSWATPRVLKMLSEIEAFNVEAHVGM 238

Chamaeleo_gracilis_Acr MANYTGLKPEQLTLRVLWRVHDSLFCQKMHNLTLPSWATPQVLKMLSQIEAFNVEAHVGM 238

Trioceros_affinis_Acr MANYTGLKPEQLTLRVLWRVHDSLFCQKMHNLTLPSWATPRVLKMLSEIEAFNVEAHVGM 238

Trioceros_harennae_Acr MANYTGLKPEQLTLRVLWRVHDSLFCQKMHNLTLPSWATPRVLKMLSEIEAFNVEAHVGM 238

Furcifer_pardalis_Acr MANYTGLKPEQLSLRVLWRVHDSLFCQKMHNLTLPSWATPRVLKMLSEIEAFNVEAHVGM 235

Trioceros_balebicornutus_Acr MANYTGLKPEQLTLRVLWRVHDSLFCQKMHNLTLPSWATPRVLKMLSEIEAFNVEAHVGM 238

Anolis_apletophallus_Pleu MANYTGLKLEQLTLRGLWRVHDSLFCQKIHNLTLPSWATPRVLRTLAEVEAFNVEAHVGM 238

Anolis_tropidonotus_Pleu MANYTGLKLEQLTLRGLWRVHDSLFCQKIHNLTLPGWATPRVLRTLAEIEAFNVEAHVGM 238

Anolis_carolinensis_Pleu MANYTGLKMEQLTLRGLWRVHDSLFCQKVHNLTLPGWATSRVLRTLAEIEAFNVEAHVGM 238

Anolis_sagrei_ordinatus_Pleu MANYTGLKLEQLTLRGLWRVHDSLFCQKIHNLTLPSWATPRVLRTLAEIEAFNVEAHVGM 238

Ctenosaura_bakeri_Pleu MANYTGLKLEQLTLRGLWRVHDSLFCQKIHNLTLPSWATPQVLTTLTEIEAFNVEAHVGM 238

Iguana_delicatissima_Pleu MANYTGLKLEQLTLRGLWKVHDSLFCQKIHNLTLPSWATPQVLTTLSEIEAFNVEAHVGM 238

Cyclura_pinguis_Pleu MANYTGLKLEQLTLRGLWRVHDSLLCQKIHNLTLPSWATPQVLTTLTEIEAFNVEAHVGM 238

Laemanctus_serratus_Pleu MANYTGLKLEQLTLRGLWRVHDSLFCQKIHNLTLPSWATPQVLTTLAEIEAFNVEAHVGM 238

Phrynosoma_blainvillii_Pleu MSNYTGLKLEQLTLKGLWRVHDSLFCQKTHNLTLPSWATPQVLTTLAEIETFNVDAHVGM 238

Sceloporus_chrysostictus_Pleu MSNYTGLKLEQLTLRGLWRVHDSLFCQKMHNLTLPSWATPQVLTTLAEIEIFNVEAHVGM 238

Sceloporus_occidentalis_Pleu MSNYTGLKLEQLTLRGLWRVHDSLFCQKMHNLTLPSWATPQVLTTLAEIEIFNVEAHVGM 238

Sceloporus_tristichus_Pleu MSNYTGLKLEQLTLRGLWRVHDSLFCQKMHNLTLPSWATPQVLTTLAEIEIFNVEAHVGM 238

Sceloporus_undulatus_Pleu MSNYTGLKLEQLTLRGLWRVHDSLFCQKMHNLTLPSWATPQVLTTLAEIEIFNVEAHVGM 238

Urosaurus_nigricaudus_Pleu MSNYTGLKLEQLTLRGLWRVHDSLFCQKMHNLTLPSWATPQVLTTLAEIEIFNVEAHVGM 238

Gambelia_wislizenii_Pleu IANYTGLKLEQLTLRGLWRVHDSLFCQKIHNLTLPSWATPQVLTMLAEIEAFNVEAHVGM 238

::* ***: **::*: **:*:***:**: ****:*.** . * *:::* **::**:*:

Acanthocercus_cyanogaster_Acr HSSQEKARLTGGLLLGAILSNFSKTVCRDLPLKMIMYSAHDSTLIALHGALGVYNGHPPP 298

Acanthocercus_minutus_Acr HSSQEKARLTGGLLLGAILSNFSKTVCRNLPLKMIMYSAHDSTLIALHGALGVYNGHPPP 298

Xenagama_zonura_Acr HSRQEKARLTGGLLLGAILSNFSKTVCRNLPLKMIMYSAHDSTLIALHGALGVYNGHPPP 298

Laudakia_wui_Acr HSSQEKARLTGGLLLGAILSNFSKTVCRDLPLKMIMYSAHDSTLIALQGALGVYNGHPPP 298

Phrynocephalus_forsythii_Acr HSSQEKARLTGGLLLGAILSNFSKTVCRDLPLKMIMYSAHDSTLIALHGALGVYNGHLPP 298

Phrynocephalus_guinanensis_Acr HSSQEKARLTGGLLLGAILSNFSKTVCRDLPLKMIMYSAHDSTLIALHGALGIYNGHPPP 295

Phrynocephalus_vlangalii_Acr HSSQEKARLTGGLLLGAILSNFSKTVCRDLPLKMIMYSAHDSTLIALHGALGIYNGHPPP 298

Phrynocephalus_versicolor_Acr HSSQEKARLTGGLLLGAILSNFSKTVCRDLPLKMIMYSAHDSTLIALHGALGIYNGHLPP 298

Agama_doriae_Acr HGSREKARLTGGLLLGAVLSNFSKTVCRDLPLKMIMYSAHDSTLIALHGALGVYNGHPPP 298

Intellagama_lesueurii_Acr HSSQEKARLTGGLLLGAILSNFSKTVCRDLPLKMIMYSAHDSTLIALQGALGVYNGHPPP 298

Pogona_vitticeps_Acr HSRQEKARLTGGLLLGAILSNFSKTVCRDLPLKMIMYSAHDSTLIALQGALGVYNGHPPP 295

Bradypodion_pumilum_Acr HSSQAKVRLAGGLLLGAILSNFSKTVCQDLPLKMIMYSAHDSTLIALQGAMGVYNGHPPP 300

Bradypodion_ventrale_Acr HSSQAKVRLAGGLLLGAILSNFSKTVCQDLPLKMIMYSAHDSTLIALQGAMGVYNGHPPP 298

Chamaeleo_calyptratus_Acr HSSQAKVRLAGGLLLGAILSNFSKTVCQHLPLKMIMYSAHDSTLIALQGAMGVYNGHPPP 298

Chamaeleo_dilepis_Acr HSSQAKVRLAGGLLLGAILSNFSKTVCQDLPLKMIMYSAHDSTLIALQGAMGVYNGHPPP 298

Chamaeleo_laevigatus_Acr HSSQAKVRLAGGLLLGAILSNFSKTVCQDLPLKMIMYSAHDSTLIALQGAMGIYNGHPPP 298

Chamaeleo_gracilis_Acr HSSQAKVRLAGGLLLGAILSNFSKTVCQDLPLKMIMYSAHDSTLIALQGAMGVYNGHPPP 298

Trioceros_affinis_Acr HSSQAKVRLAGGLLLGAILSNFSKTVCQDLPLKMIMYSAHDSTLIALQGAMGVYNGHPPP 298

Trioceros_harennae_Acr HSSQAKVRLAGGLLLGAILSNFSKTVCQDLPLKMIMYSAHDSTLIALQGAMGVYNGHPPP 298

Furcifer_pardalis_Acr HSSQAKVRLAGGLLLGAILSNFSKTVCQDLPLKMIMYSAHDSTLIALQGAMGVYNGHPPP 295

Trioceros_balebicornutus_Acr HSSQAKVRLAGGLLLGAILSNFSKTVCQDLPLKMIMYSAHDSTLIALQGAMGVYNGHPPP 298

Anolis_apletophallus_Pleu HASQEKVRFTGGLLLDAILTNFSKIACRDLPLKMIMFSAHDSTLIALHGALGIYNGHPPP 298

Anolis_tropidonotus_Pleu HASQEKVRFTGGLLLDAILSNFSKIVCRDLPLKMIMFSAHDSTLIALHGALGIYNGHPPP 298

Anolis_carolinensis_Pleu HASQEKVRFTGGLLLDAILSNFSKIVCRDLPLKMIMYSAHDSTLIALHGALGIYNGHPPP 298

Anolis_sagrei_ordinatus_Pleu HASQEKVRFTGGLLLDAILSNFSKIACRDLPLKMIMYSAHDSTLIALHGALGIYNGHPPP 298

Ctenosaura_bakeri_Pleu HASQEKARFTGGLLLGAILSNFSKIMCRDLPLKMIMYSAHDSTLIALHGALGIYNGHPPP 298

Iguana_delicatissima_Pleu HASQEKARFSGGLLLGAILSNFSKIMCRDLPLKMIMYSAHDSTLIALHGALGIYNGHPPP 298

Cyclura_pinguis_Pleu HASQEKARFTGGLLLGAILSNFSKIMCQDLPLKMITYSAHDSTLIALHGALGIYNGHPPP 298

Laemanctus_serratus_Pleu YASQEKARFTGGLLLDAILSNFSKIVCRDLPLKMIMYSAHDSTLIALHGALGIYNRHPPP 298

Phrynosoma_blainvillii_Pleu HGTQEKARFTGGLLLGAILSNFSKIVCQDLPLKMVMYSAHDSTLIALHGALGIYNGHPPP 298

Sceloporus_chrysostictus_Pleu YATREKARFTGGLLLGAILSNFSKIVCRDLPQKMVMYSAHDSTLIALHGALGIYNGHPPP 298

Sceloporus_occidentalis_Pleu YATQEKARFTGGLLLGAILSNFSKIVCRDLPLKMVMYSAHDSTLIALHGALGLYNGHLPP 298

Sceloporus_tristichus_Pleu YATQEKARFTGGLLLGAILSNFSKIVCRDLPLKMVMYSAHDSTLIALHGALGLYNGHPPP 298

Sceloporus_undulatus_Pleu YATQEKARFTGGLLLGAILSNFSKIVCRDLPLKMVMYSAHDSTLIALHGALGLYNGHPPP 298

Urosaurus_nigricaudus_Pleu YATREKARFTGGLLLGAILSNFSKIVCRDMPLKMVMYSAHDSTLIALHGALGIYNGHPPP 298

Gambelia_wislizenii_Pleu HAHQEKARFTGGLLLDAILSNFSKIVRRELPLKMIMYSAHDSTLIALHGALGIYNGHLPP 298

:. : *.*::*****.*:*:**** :.:* **: :**********:**:*:** * **

Acanthocercus_cyanogaster_Acr YAACHGFEFYQESNNSFSVTMFYRNASNRQPYTLILPGCPTPCPLPLFTKLTHAVIPQDW 358

Acanthocercus_minutus_Acr YAACHGFEFYQESNNSFSVAMFYRNASNQQPYTLTLPGCPTPCPLPLFTKLTHAVIPQDW 358

Xenagama_zonura_Acr YAACHGFEFYQESNNSFSVAMFYRNASNQQPYTLTLPGCPTPCPLLLFTKLTHAVIPQDW 358

Laudakia_wui_Acr YAACHGFEFYQESNNSFSVAMFYRNTSDRQPHTLTLPGCPTPCPLLLFTKLTHAVVPQDW 358

Phrynocephalus_forsythii_Acr YAACHGFEFYQESNNSFSVAMFFRNTSDQQPHTLTLPGCPTPCPLPLFTKLTHAVVPQDW 358

Phrynocephalus_guinanensis_Acr YAACHGFEFYQESNNSFSVAMFYRNTSDLQPHTLTLPGCPTPCPLPLFTKLTHAVVPQDW 355

Phrynocephalus_vlangalii_Acr YAACHGFEFYQESNNSFSVAMFYRNTSDQQPHTLTLPGCPTPCPLPLFTKLTHAVVPQDW 358

Phrynocephalus_versicolor_Acr YAACHGFEFYQESNNSFSVAMFYRNTSDRQPHTLTLPGCPTPCPLPLFTKLTHAVVPQDW 358

Agama_doriae_Acr YAACHGFEFYQESNNSFSVAMFYRNASDQQPYALNLPGCSTPCPLPLFTKLTHAVIPQDW 358

Intellagama_lesueurii_Acr YAACHGFEFYQESNNSFSVAMFYRNTSDQQPHTLTLPGCPTPCPLPLFTKLTRAVVPPDW 358

Pogona_vitticeps_Acr YAACHGFEFYQETNNSFTVAMFYRNTSDQQPHTLTLPGCPTHCPLPLFTKLTRAVVPQDW 355

Bradypodion_pumilum_Acr YAACHGFEFYQESDKSFTVAMFYRNTSKGQPHVLTLPGCPTPCPLALFTNLTHAVVPQDW 360

Bradypodion_ventrale_Acr YAACYGFEFYQESDKSFSVAMFYRNTSKGQPHVLTLPGCPTPCPLALFTNLTRAAVPQDW 358

Chamaeleo_calyptratus_Acr YAACHGFEFYQESDKSFSVVMFYRNTSKGQPHMLTLPGCPTPCPLARFTNLIRAXVPQDW 358

Chamaeleo_dilepis_Acr YAACHGFEFYQESDKSFSVVMFYRNTSKGQPHMLTLPGCPTPCPLALFTNLIRAVVPQDW 358

Chamaeleo_laevigatus_Acr YTACHGFEFYQESDKSFSVVMFYRNTSKGQPHMLTFPGCPTPCPLALFTNLIRAVVPQDW 358

Chamaeleo_gracilis_Acr YAACHGFEFYQESDKSFSVVMFYRNTSNGQPHMLTLPGCPTPCPLALFTNLIRAVVPQDW 358

Trioceros_affinis_Acr YAACHGFEFYQESDKSFSVAMFYRNTSKGQPHMLTLPGCPAPCPLVLFTNLTRAVVPQDW 358

Trioceros_harennae_Acr YAACHGFEFYQESDKSFSVAMFYRNTSKGQPHMLTLPGCPAPCPLALFTNLTHAVVPQDW 358

Furcifer_pardalis_Acr YAACHGFEFYQESDKSFTVAMFYRNTSKGQPHMLTLPGCPTPCPLVLFTNLTRAVVPQDW 355

Trioceros_balebicornutus_Acr YAACHGFEFYQESDKSFSVAMFYRNTSKGQPHMLTLPGCPAPCPLALFTNLTRAVIPQDW 358

Anolis_apletophallus_Pleu YAACHGFEFYQETNNSFSIGMFYRNTSDQPPHVVALPGCSTPCPLSRFIQLTRAVIPHDW 358

Anolis_tropidonotus_Pleu YAACHGFEFYQETNNSFSIGMFYRNTSDQPPHVVALPGCSTPCPLSRFIQLTRAVIPHDW 358

Anolis_carolinensis_Pleu YAACHGFEFYQETNNSFSIGMFYRNTSDQPPYVVTLPGCSTPCPLPRFIQLTHTVIPHDW 358

Anolis_sagrei_ordinatus_Pleu YAACHGFEFYQEANNSFSIGMFYRNMSNQPPHEVALPGCSTPCPLPRFIQLTRAVIPRDW 358

Ctenosaura_bakeri_Pleu YAACHGFEFYKEIDNSFSVGMFYRNTSDQPPHVLALPGCSTPCPLPQFIHLTSAVVPHDW 358

Iguana_delicatissima_Pleu YAACHGFEFYEEINNSFSVGMFYRNTSDQPPHVLALPGCSTPCPLPQFIHLTSVVVPNDW 358

Cyclura_pinguis_Pleu YAACHGFEFYEEINNSFSVGMFYRNTSDQPPHVLALPGCSTPYPLPQFIHLTSVVVPHDW 358

Laemanctus_serratus_Pleu YAACHGFEFYQEVNNSFNVGMFYRNTSDQPPHVLSLPGCSTPCPLPRFIHLTRAVIPHDW 358

Phrynosoma_blainvillii_Pleu YAACHGFEFYQESNNSFSVAMFYRNTSDQPPHILALPGCSTPCPLPRFIHLTRPVVPHDW 358

Sceloporus_chrysostictus_Pleu YAACHGFEFYQERNNSFSVAMFYRNTSDQPPHVLALPGCSTPCPLLRFIHLTRPVVPHDW 358

Sceloporus_occidentalis_Pleu YAACHGFEFYQESNNSFSVAMFYRNTSDQPPHVLALPGCSTPCPLPRFIHLTRPVVPHDW 358

Sceloporus_tristichus_Pleu YAACHGFEFYQESNNSFSVAMFYRNTSDQPPHVLALPGCSTPCPLPRFIHLTRPVVPHDW 358

Sceloporus_undulatus_Pleu YAACHGFEFYQESNNSFSVAMFYRNTSDQPPHVLALPGCSTPCPLPRFIHLTRPVVPHDW 358

Urosaurus_nigricaudus_Pleu YAACHGFEFYQESNNSFSVAMFYRNTSDQPPHVLALPGCSTPCPLPRFIHLTRPVVPHDW 358

Gambelia_wislizenii_Pleu YAACHGFEFYQEINNSFSVGMFYRNRSDQPPHVLDLPGCSTPCPLPQFIHLTRAVVPHDW 358

*:**:*****:* ::**.: **:** *. *: : :***.: ** * :* :* **

Acanthocercus_cyanogaster_Acr EAECQNPRRSTGNQGAFTHAHPAT------------------------------------ 382

Acanthocercus_minutus_Acr EAECQNPRRSTGNQGAFKHLPPAT------------------------------------ 382

Xenagama_zonura_Acr EAECQNPRRSTGNQGAFKHLPPAT------------------------------------ 382

Laudakia_wui_Acr EAECQNPQRSTGNQGAFKHTNPAT------------------------------------ 382

Phrynocephalus_forsythii_Acr EAECQNPQRSPGNQGAFKHTYPAT------------------------------------ 382

Phrynocephalus_guinanensis_Acr EAECQNPQRSPGNQGAFQHTYPAT------------------------------------ 379

Phrynocephalus_vlangalii_Acr EAECQNPQRSPGKQGAFQHTYPAT------------------------------------ 382

Phrynocephalus_versicolor_Acr EAECQNPQRSPGNQGAFKYKYPAT------------------------------------ 382

Agama_doriae_Acr EAECQHPRRSTGNPGAFKHARPAT------------------------------------ 382

Intellagama_lesueurii_Acr DAECQNPQSSTGNESLHTHTHILRP----------------------------------- 383

Pogona_vitticeps_Acr DAECQNPQNRTGNESLHTHTHTVTLVLKVE------------------------------ 385

Bradypodion_pumilum_Acr DAECQTPQRSTGN-KATTSAQF-FLCYIL------------------------------- 387

Bradypodion_ventrale_Acr DAECQTPQISTGNKATTSAVLL-VLYSIP-NICNPHGNKP-------------------- 396

Chamaeleo_calyptratus_Acr DAECQTTQRSTGK-KATTSIQPFFLCCIP-NIGNPHGDQP-------------------- 396

Chamaeleo_dilepis_Acr DAECQTTQRSTGN-KATTSIQPSFLCCIP-NIGNPHGAIII----------VWGLLHGKL 406

Chamaeleo_laevigatus_Acr DAECQTTQRSTGNKKATASI---------------------------------------- 378

Chamaeleo_gracilis_Acr DAECQTTQ-STGNKKAATSIQPFFLCCIP-NIGNPHGD---------------------- 394

Trioceros_affinis_Acr DAECQTPQRSTGNKKAATSTQP-FLCCIL------------------------------- 386

Trioceros_harennae_Acr DAECQTPPRSTGNKKAATSTQP-FLCCIL------------------------------- 386

Furcifer_pardalis_Acr DAECQTPQRSTGNKKATTSTQPYFLCCILFCIGNPHGNQP-------------------- 395

Trioceros_balebicornutus_Acr DAECQTPQRSTGNKKAVTSTQP-FLCSEY------------------------------W 387

Anolis_apletophallus_Pleu ETECQNPQKSTGNKSPPPPSR-NMVESKFTSVLNTCFVGRT-------------VTALAV 404

Anolis_tropidonotus_Pleu ETECQNPQKSTGN----------------------------------------------- 371

Anolis_carolinensis_Pleu ETECQNPQKSTGNTPPPKKKPGKC------------------------------------ 382

Anolis_sagrei_ordinatus_Pleu ETECQNPQKSTGRTVTALAVAVALLSLMLIGVG--------------------------- 391

Ctenosaura_bakeri_Pleu DAECQNPQKSTGNKNPHTQL---------------------------------------- 378

Iguana_delicatissima_Pleu DAECQNPQKSTGNKKTHTQP---------------------------------------- 378

Cyclura_pinguis_Pleu DAECQNLQKSTGNKNPHTQP---------------------------------------- 378

Laemanctus_serratus_Pleu DAECQNPQKSTGNKNPHSSPQ--------------------------------------- 379

Phrynosoma_blainvillii_Pleu DAECQNPRKSTGNKSPHCQPPISLSEFKFTPVLNTVFQDAQ------------------- 399

Sceloporus_chrysostictus_Pleu DAECQNPQKSTGNKSPHCQPQICSSEFKIYTCP--------------------------- 391

Sceloporus_occidentalis_Pleu DAECQNPRKNTGNKSPHCQL---------------------------------------- 378

Sceloporus_tristichus_Pleu DAECQNPRKSTGNKSPHCQL---------------------------------------- 378

Sceloporus_undulatus_Pleu DAECQNPRKSTGNKSPHCQL---------------------------------------- 378

Urosaurus_nigricaudus_Pleu DAECQNPQKSTGNKSPHCQPQISFSEFKIYTCP--------------------------- 391

Gambelia_wislizenii_Pleu DAECQNPQKTTGNKSPHSNLR-RLSELKNLHLSLTLFCRTHGNSSGCGSRSAEHVTNWSG 417

::*** .*.

Acanthocercus_cyanogaster_Acr --------------------------

Acanthocercus_minutus_Acr --------------------------

Xenagama_zonura_Acr --------------------------

Laudakia_wui_Acr --------------------------

Phrynocephalus_forsythii_Acr --------------------------

Phrynocephalus_guinanensis_Acr --------------------------

Phrynocephalus_vlangalii_Acr --------------------------

Phrynocephalus_versicolor_Acr --------------------------

Agama_doriae_Acr --------------------------

Intellagama_lesueurii_Acr --------------------------

Pogona_vitticeps_Acr --------------------------

Bradypodion_pumilum_Acr FRILAILMAISHDCPENITQ------ 407

Bradypodion_ventrale_Acr --------------------------

Chamaeleo_calyptratus_Acr --------------------------

Chamaeleo_dilepis_Acr GSTLNKFMPMTLLALGKNAFLADDIA 432

Chamaeleo_laevigatus_Acr --------------------------

Chamaeleo_gracilis_Acr --------------------------

Trioceros_affinis_Acr FRILAILMAISH-------------- 398

Trioceros_harennae_Acr FRILAILMAISH-------------- 398

Furcifer_pardalis_Acr --------------------------

Trioceros_balebicornutus_Acr QSSWRSAM------------------ 395

Anolis_apletophallus_Pleu AVALLSILLIGVGVLYCRR------- 423

Anolis_tropidonotus_Pleu --------------------------

Anolis_carolinensis_Pleu --------------------------

Anolis_sagrei_ordinatus_Pleu -------------VLYCRS------- 397

Ctenosaura_bakeri_Pleu --------------------------

Iguana_delicatissima_Pleu --------------------------

Cyclura_pinguis_Pleu --------------------------

Laemanctus_serratus_Pleu --------------------------

Phrynosoma_blainvillii_Pleu --------------------------

Sceloporus_chrysostictus_Pleu --------------------------

Sceloporus_occidentalis_Pleu --------------------------

Sceloporus_tristichus_Pleu --------------------------

Sceloporus_undulatus_Pleu --------------------------

Urosaurus_nigricaudus_Pleu --------------------------

Gambelia_wislizenii_Pleu DSVLQKIMRRGSHGNNEKFPS----- 438

**AMBN-AA-MAFFT-Iguania+Mammal – DIVERGE + PCOC ≥ 0.9**

CLUSTAL W (1.8) multiple sequence alignment (ALTER 1.3.3)

Acanthocercus_cyanogaster_Acr MKQWILVSSLLGICSALPILPQHAGIPGIGSISLETMRQMAGQNMPNGLSQYAMPYSYGG 60

Acanthocercus_minutus_Acr MKQWILVSSLLGICSALPILPQHAGIPGIGSISLETMRQMAGQNMPNGLSQYAMPYSYGG 60

Xenagama_zonura_Acr MKQWILVSSLLGICSALPILPQHAGIPGIGSISLETMRQMAGQNMPNGLSQYVMPYSYGG 60

Agama_doriae_AMBN_Acr MKQWILVSSLLGICSALPILPQHAGIPGLGSISLETMRQMGGQNMPNGLSQYAMPYSYGG 60

Laudakia_wui_Acr MKQWILVSSLLGICSALPILPQHAGIPGIGSISLETMRQMGGQNMPNGLPQYAMPYRYGG 60

Phrynocephalus_forsythii_Acr MKQWILVSSLLGLCSALPILPQHAGIPGIGSISLETMRQMSGQNIPHGLPQYAMPYSYGG 60

Phrynocephalus_guinanensis_Acr MKQWILVSSLLGLCSALPILPQHAGIPGIGSISLETMRQMSGQNIPHGLPQYAMPYSYGG 60

Phrynocephalus_vlangalii_Acr MKQWILVSSLLGLCSALPILPQHAGIPGIGSISLETMRQMSGQNIPHGLPQYAMPYSYGG 60

Phrynocephalus_versicolor_Acr MKQWILVSSLLGLCSALPILPQHAGIPGIGSISLETMRQMSGQNIPHGLPQYAMPYSYGG 60

Intellagama_lesueurii_Acr MKQWILVSSLLGICSALPILPQHAGIPGIGSISLETMRQMGGRNGPNALSQYAMPYSYGG 60

Pogona_vitticeps_Acr MKQWILVSSLLGICSALPILPQHAGIPGLGSISLETMRQMGGRNGPNALSQYAMPYSYGD 60

Bradypodion_pumilum_Acr MKQWILVSSLLGICSALPILPQHAGVPGMGSISLETMRQMSGGNIPSVLQQYAMPYNYAG 60

Bradypodion_ventrale_Acr MKQWILVSSLLGICSALPILPQHAGVPGMGSISLETMRQMSGGNIPSVLQQYAMPYNYAG 60

Chamaeleo_calyptratus_Acr MKQWILVSSLLGICSAVPILPQHSGIPGMGSISLETMRQMSGGNIPSVLPQYAMPYNYAG 60

Chamaeleo_dilepis_Acr MKQWILLSSLLGICSALPILPQHSGIPGMGSISLETMRQMSGGNIPSVLPQYAMPYNYAG 60

Chamaeleo_gracilis_Acr MKQWILLSSLLGICSALPILPQHSGIPGMGSISLETMRQMSGGNIPSVLPQYAMPYNYAG 60

Chamaeleo_laevigatus_Acr MKQWIFVSCLLGICSALPILPQHSGIPGMGSISLETMRQMSGGNIPNVLPQYAMPYNYAG 60

Furcifer_pardalis_Acr MKQWILVSSLLGICSALPILPQHAGIPGMGSISLETMRQMSGGNIPSVLPQYAMPYNYGG 60

Trioceros_affinis_AMBN_Acr MKQWILVSSLLGICSALPILPQHAGIPGMGSISFETMRQMSGGNIPSVLPQYAMPYNYPG 60

Trioceros_balebicornutus_Acr MKQWILFSSLLGICYALPILPQHAGIPGMGSISLETMRQMSGGNIPNVLPQYAMPYNYPG 60

Trioceros_harennae_Acr MKQWILFSSLLGICSALPILPQHAGIPGMGSISLETMRQMSGGNIPNVLPQYAMPYNYPG 60

Anolis_apletophallus_Pleu MKQWMLVSSLLGICSALPLFPQHAGIPGMGSISLERTRQLGGLNMPPAPPQ-ALPYSYAD 59

Anolis_rodriguezii_Pleu MKQWMLVSSLLGICSALPLFPQHAGIPGMGSISLERTRQLGGLNMPPAPPQ-ALPYSYAD 59

Anolis_carolinensis_Pleu MKQWMLVSSLLGICSALPLFPQHAGIPGMGSISLERTRQLGGLNIPPAVPQ-ALPYSYAD 59

Anolis_sagrei_ordinatus_Pleu MKQWMLVSSLLGICSALPLFPQHAGIPGMGSISLERTRQLGGLNIPSAPPQ-ALPYSY-D 58

Ctenosaura_bakeri_Pleu MKQWMLVSSLLGICSALPILPQHAGIPGMGSISLERMRQLGGQNIPNVLSQYASPYSYAD 60

Iguana_delicatissima_Pleu MKQWMLISSLLGICSALPILPQHAGIPGMGSISLERMRQLGGQNIPNVLPQYASPYSYAD 60

Cyclura_pinguis_Pleu MKQWMLVSSLLGICSALPILPQHAGIPGMGSISLERMRQLGGQNIPNVLSQYASPYSYAD 60

Gambelia_wislizenii_Pleu MKQWMIVSSLLGICSALPILPQHIRIPGMGSISLERMRQLGGPDMPNALPQYAPPYSYAD 60

Laemanctus_serratus_Pleur MKQWILVSSLLGICSALPILPQHTGIPGMGSISLERMRQLGGPNMPNVLPQYAPPYGYAD 60

Phrynosoma_blainvillii_Pleu MKQWILVSSLLGICSALPILPQHVGIPGMGSISLEGMKQLAGANMPNVLPQYAPPYGYAN 60

Phrynosoma_platyrhinos_Pleu MKQWILVSSLLGICSALPILPQHVGIPGMGSISLEGMKQLAGANMPNVLPQYAPPYGYAN 60

Sceloporus_chrysostictus_Pleur MKQWILVSSLLGICSAVPILPQHAGIPGMGSISLEGMRQVAGANMPNVLPQYASPYGYAN 60

Sceloporus_occidentalis_Pleu MKQWILVSSLLGICSALPILPQHAGIPGMGSISLEGMRQMTGANMPNVLPQYASRYGFAN 60

Sceloporus_tristichus_Pleu MKQWILVSSLLGICSALPILPQHAGIPGMGSISLEGMRQMAGANMPNVLPQYASPYGFAN 60

Sceloporus_undulatus_Pleu MKQWILVSSLLGICSALPILPQHAGIPGMGSISLEGMRQMAGANMPNVLPQYASPYGFAN 60

Urosaurus_nigricaudus_Pleu MKQWILVSSLLGICSALPILPQHAGIPGMGSISLEGMRQVAGANVPNVLPQYASPYGYAN 60

Canis_lupus_dingo_AMBN MKDLVLILCLLKMSSAVPVFPQQPGTPGMASLSLETMRQLGSLQGLNMLSQYS-RFGFGK 59

Vulpes_vulpes_AMBN MKDLVLILCLLKMSSAVPVFPQQPGTPGMASLSLETMRQLGSLQGLNMLSQYS-RFGFGK 59

Ursus_arctos_AMBN MKDLILILCLLGMSSAVPVFPRQPGTPGMASLSLETMRQLGSLQGLNMLSQYS-RFGFGK 59

Meles_meles_AMBN MKDLILILCLLEMSSAVPVFPQQPGTPGMASLSLETMRQLGSLQGLNMLSQYS-RYGFGK 59

Mustela_putorius_AMBN MKDLILILCVLEMSSAVPVFPQQPGTPGMASLSLETMRQLGSLQGLNMLSQYS-RFGFGK 59

Phacochoerus_africanus_AMBN MKDMVLILCLLKMSSAVPAFPRQPGTPGVASLSLETMRQLGSLQGLNMLSQYS-RFGFGK 59

Phyllostomus_hastatus_AMBN MKNLVLILYLLEMSSAVPVFPQQPGTPGMASLSLETMRQLSTLQGLNMLSQYS-KFGLGK 59

Homo_sapiens_AMBN MKDLILILCLLEMSFAVPFFPQQSGTPGMASLSLETMRQLGSLQRLNTLSQYS-RYGFGK 59

Saimiri_boliviensis_AMBN MKDLILILGLLKMSFAVPLFPQQSGTPGMASLSLETMRQLGSLQGLNTLSQYS-RFGFGK 59

Echinops_telfairi_AMBN ------------------MFPRQPGTPGMASLSLETMRQLGSLQGLNILPQYS-RFGFGK 41

Suncus_etruscus_AMBN MKELILILCLLKMSSAVPAFPQQSGTPGMASLSLETMRQLGSLQGLNLLSQYS-RYGFGK 59

Erinaceus_europaeus_AMBN MKDLILILYLLEMCSAVPVFPKQPGMPGMASLSLE----------------YS-RFGFGK 43

:*:: **:.*:*:* :

Acanthocercus_cyanogaster_Acr LLFPPWIHNHLPPHSSFYWMRQ-RPQDHETQRYEYTMPVHPPPLPSQQTPTL--LQRP-- 115

Acanthocercus_minutus_Acr LLFPPWIHNHLPPHSSFYWMRQ-RPQDHETQRYEYTMPVHPPPLPSQQTPTL--LQRP-- 115

Xenagama_zonura_Acr LLFPPWIHNHLPPHSSFYWMRQ-RPQDHETQRYEYTMPVHPPPLPSQQTPTL--LQRP-- 115

Agama_doriae_AMBN_Acr MLFPSWIHNHLPPHSSFYWMRQ-RPQDHETQRYEYTMPVHPPPLPSQQTPTL--LQRP-- 115

Laudakia_wui_Acr FLFPPWMHNHLPPHSSFYWMRP-RPQDHETQRYEYTLPVHPPPLPSQQTPTL--LQRP-- 115

Phrynocephalus_forsythii_Acr FLFPPWIHNHLPPHSSFYWTRQ-RPQDHETQRYEYTLPVHPPPLPSQQTPTL--LQRP-- 115

Phrynocephalus_guinanensis_Acr FLFPPWIHNHLPPHSSFYWTRQ-RPQDHETQRYEYTLPVHPPPLPSQQTPTL--LQRP-- 115

Phrynocephalus_vlangalii_Acr FLFPPWIHNHLPPHSSFYWTRQ-RPQDHETQRYEYTLPVHPPPLPSQQTPTL--LQRP-- 115

Phrynocephalus_versicolor_Acr FLFPPWIHNHLPAHSSFYWMRQ-RPQDHETQRYEYTLPVHPPPLPSQQTPTL--LQRP-- 115

Intellagama_lesueurii_Acr LFFPPWMHNNLPPHSSFYWLRQ-RPQDHETQRYEYTMPVHPPPLPSQQTPTL--LQQPGI 117

Pogona_vitticeps_Acr LFFPPWMHNHLPPHSSFYWLRQ-RPQDHETQRYEYTMPVHPPPLPSQQTPTL--LQRPGI 117

Bradypodion_pumilum_Acr LYFPPWMHNHLPSHPSFYWLRQ-RPQDHGTQQYEYTMPVHPPPLPSQQTPIL--LQRPGI 117

Bradypodion_ventrale_Acr LYFPPWMHNHLPSHPSFYWLRQ-RPQDHGTQQYEYTMPVHPPPLPSQQTPIL--LQRPGI 117

Chamaeleo_calyptratus_Acr LYFPPWMHNHLPSHPSFYWLRQ-RPQDHGTQRYEYTMPVHPPPLPSQQTPIL--LQRSGI 117

Chamaeleo_dilepis_Acr LYFPPWMHNHLPSHPSFYWLRQ-RPQDHGTQRYEYTMPVHPPPLPSQQTPIL--LQRPGV 117

Chamaeleo_gracilis_Acr LYFPPWMHNQLPSHPSFYWLRQ-RPQDHGTQRYEYTMPVHPPPLPSQQTPIL--LQRPGI 117

Chamaeleo_laevigatus_Acr LYFPPWMHNHLPSHPSFYWLRQ-RPQDHGTQRYEYTMPVHPPPLPSQQTPIL--LQRPGI 117

Furcifer_pardalis_Acr LYFPPWMHNHLPSHPSFYWLRQ-RPQDHGTQRYEYTMPVHPPPLPSQQTPIL--LQRPGI 117

Trioceros_affinis_AMBN_Acr LYFPPWMHNHLPSHPSFYWLRQ-RPQDHETQRYEYTMPVHPPPLPSQQTPIL--LQRPGI 117

Trioceros_balebicornutus_Acr LYFPPWMHNHLPSHPSFYWLRQ-KPQDHGTQRYEYTMPVHPPPLPSQQTPIL--LQQPGI 117

Trioceros_harennae_Acr LYFPPWMHNHLPSHPSFYWLRQ-KPQDHGTQRYEYTMPVHPPPLPSQQTPIL--LQRPGN 117

Anolis_apletophallus_Pleu PFHSMWGRSFLPSH-TFLWMTH-GPQRQETQQYEYAMPVHPPPLPSLQPSIQ--FSEPRL 115

Anolis_rodriguezii_Pleu PFHSMWGRSFLPSH-TFLWMTH-GPQRQETQQYEYAMPVHPPPLPSLQPSIQ--FAEPRL 115

Anolis_carolinensis_Pleu PFHSMWGRSFLPSH-TFLWMTQGGPQRQDTQQYEYAMPVHPPPLPSLQTPIQ--FAEPRL 116

Anolis_sagrei_ordinatus_Pleu PFHSMWGRSFLPSH-TFLWMTQ-GPQRQETQQYEYAMPVHPPPLPSLQPAIQ--FSESRL 114

Ctenosaura_bakeri_Pleu PFHYMWGHSLLPSH-AFYWMRQ-RSQPHETQQYEYSMPVHPPPLPSIQTPTQ--FQQPGI 116

Iguana_delicatissima_Pleu PFHHMWGHSLLPSH-AFYWMRQ-RSQPHETQQYEYSMPVHPPPLPSIQTPTQ--FQQPGI 116

Cyclura_pinguis_Pleu PFHYMWGHSLLPSH-AFYWMRQ-RSQPHETQQYEYSMPVHPPPLPSIQTPTQ--FQQPGI 116

Gambelia_wislizenii_Pleu PYYSMWGRSFLPSH-GFYWMRQ-RPQPHETQQYEYSMPVHPPPLPSLQTPTQ--FQQPGI 116

Laemanctus_serratus_Pleur PFHAMWGRSFLPSH-GFYWMRQ-RSQPHETQQYEYSMPVHPPPLPSLQTPTQ--FQQPGI 116

Phrynosoma_blainvillii_Pleu TLNSLWMHGFLPPH-TFYWRSQ-RAQPHETQQYEYSMPVHPPPLPSLQTPIQ--FQQPGI 116

Phrynosoma_platyrhinos_Pleu TLNSLWMHGFLPPH-TFYWRSQ-RAQPHETQQYEYSMPVHPPPLPSLQTPIQ--FQQPGI 116

Sceloporus_chrysostictus_Pleur GFHSMWTHGFLPSH-AFYWRRQ-RAQPHETQQYEYSMPVHPPPLPSLQTPIQ--FQQPGI 116

Sceloporus_occidentalis_Pleu TFPSMWTHGFLPSH-GFYWRRQ-RAQPHETQQYEYSMPVHPPPLPSLQTPIQ--FQQPGI 116

Sceloporus_tristichus_Pleu AFPSMWTHGFLPSH-GFYWRRQ-RAQPHETQQYEYSMPVHPPPLPSLQTPIQ--FQQPGI 116

Sceloporus_undulatus_Pleu TFPSMWTHGIFPSH-GFYWRRQ-RAQPHETQQYEYSMPVHPPPLPSLQTPIQ--FQQPGI 116

Urosaurus_nigricaudus_Pleu PFHSMWTHGFLPSH-TFYWMRQ-RAQPHETQQYEYSMPVHPPPLPSLQTPIQ--FQQPGI 116

Canis_lupus_dingo_AMBN SFNSLWMHGLLPPHSSFPWM---RPREHETQQYEYSLPVHPPPLPS-QPSLQ--PQQPG- 112

Vulpes_vulpes_AMBN SFNSLWMHGLLPPHSSFPWM---RPREHETQQYEYSLPVHPPPLPS-QPSLQ--PQQPG- 112

Ursus_arctos_AMBN SFNSLWLHGLLPPHSSFPWM---RPREHETQQYEYSLPVHPPPLPS-QPSLQ--PQQPG- 112

Meles_meles_AMBN SFNSLWMHGLLPPHSSFPWM---RPREHETQQYEYSLPVHPPPLPS-QPSLQ--PQQPG- 112

Mustela_putorius_AMBN SFNSLWMHGLLPPHSSFPWM---RPREHETQQYEYSLPVHPPPLPS-QPSPQ--PQQPG- 112

Phacochoerus_africanus_AMBN SFNSLWMHGLLPPHSSFQWM---RPREHETQQYEYSLPVHPPPLPS-QPSLQ--PQQPG- 112

Phyllostomus_hastatus_AMBN SLNSLWMHGLLPAHPPFPWI---RPREHETQQYEYSLPVHPPPLPS-QPSLH--PQLPG- 112

Homo_sapiens_AMBN SFNSLWMHGLLPPHSSLPWM---RPREHETQQYEYSLPVHPPPLPS-QPSLK--PQQPG- 112

Saimiri_boliviensis_AMBN SFNSLWMHGLLPPHSSFPWM---RPREHETQQYEYSLPVHPPPLPS-QPSLK--PQQPG- 112

Echinops_telfairi_AMBN SFNSLWMHGLLPPHSSFPWM---RPREHETQQYEYSLPVHPPPLPS-QQSLQ--PQQSG- 94

Suncus_etruscus_AMBN SFNSLWLQGLLPPHSSLPWM---RPREHETQQYEYALPVHP---PS-QPSLQDGSHLPG- 111

Erinaceus_europaeus_AMBN SFNSLWMNGLLPPNPSFPWM---RPREHETQQYEYSLPVHPPPLPS-QPSLQ--SHQPG- 96

* .. :*.: : * .: : **:***::**** ** * . .

Acanthocercus_cyanogaster_Acr -EQPHYQ------FTDLAPTMPVQLQQYEMQPPF-QQK-LSPEGNQPIVPQKKQLP-LDR 165

Acanthocercus_minutus_Acr -EQPHYQ------FTDLAPTMPVQLQQYEMQPPF-QQK-LSPEGNQPIVPQKKRLP-LDR 165

Xenagama_zonura_Acr -EQPHYQ------FTDLAPTMPVQLQQYEMQPPF-QQK-LSPEGNQPIVPQKKRLP-LDR 165

Agama_doriae_AMBN_Acr -EQPHYQ------FTDLAPTMPVQIQQYEMQPPF-QQK-VLPEGNQPLVPQKKRLP-LDR 165

Laudakia_wui_Acr -EQPHYQ------FTDLAPTMPVQLQQYELQPPF-QQK-LLPEGNQPVVPQKKQPP-LDR 165

Phrynocephalus_forsythii_Acr -EQPHYQ------FTDLAPTMPVQLQQYEMQPPF-QQK-LLPEGNQPFVPQKKQLP-MDR 165

Phrynocephalus_guinanensis_Acr -EQPHYQ------FTDLAPTMPVQLQQYEMQPPF-QQK-LLPEGNQPFVPQKKQLP-VDR 165

Phrynocephalus_vlangalii_Acr -EQPHYQ------FTDLAPTMPVQLQQYEMQPPF-QQK-LLPEGNQPFVPQKKQLP-VDR 165

Phrynocephalus_versicolor_Acr -EQPHYQ------FTDLTPTMPVQLQQYEMQPPF-QQK-LLPEGNQPLVPQKKQPP-VDR 165

Intellagama_lesueurii_Acr PEQPHYQ------FTDLTPTMQVQLQQYEMQPPF-QQM-PLPEGNQPVVPQKKRPP-LDR 168

Pogona_vitticeps_Acr PEQPHYQ------FTDLTPTMQAQLQQYEMQPPF-QQT-PLPEGNQPVVPQKKRPP-LDR 168

Bradypodion_pumilum_Acr QEQPHYQ------FIDLTPTK--QVQQYEMQPPF-QQN-PFPEGNQPVVPQKQLLP-SDR 166

Bradypodion_ventrale_Acr QEQPHYQ------FIDLTPTK--QVQQYEMQPPF-QQK-PFPEGNQPVVPQKQLLP-SDR 166

Chamaeleo_calyptratus_Acr QEQPHYQ------FIDLAPTK--QVQQYEMQPPF-QQK-PLPEGNQPVVPQKQLLP-SDR 166

Chamaeleo_dilepis_Acr QEQPHYQ------FIDLAPTK--QVQQYEMQPPF-QQK-PSPEGNQPLVPQKQLLP-SDR 166

Chamaeleo_gracilis_Acr QEQPHYQ------FIDLAPTK--QVQQYEMQPPF-QQK-PSPEGNQPLVPQKQLLP-SDR 166

Chamaeleo_laevigatus_Acr QEQPHYQ------FIDLTPTK--QVQQYEMQPPF-QQK-PSPEGNQPVVPQKQLLP-SDR 166

Furcifer_pardalis_Acr QEQPHYQ------FIDLTPTK--QVQQYEMRPPF-QQN-PSPDGNQPVVPQKQLLS-SDR 166

Trioceros_affinis_AMBN_Acr QEQPHYQ------FIDLSPTK--QVQQYEMQPPF-QQK-PSLEGSQPVVPQKQLLS-LDR 166

Trioceros_balebicornutus_Acr QEQPHYQ------FIDLTPTK--QVQQYEMQPPF-QQK-PSPEGNQPVVPQKQLLS-SDR 166

Trioceros_harennae_Acr QEQPHYQ------FIDLTPTK--QVQQYEMQPPF-QQK-PSPEGNQPVVPQKQLLS-SDR 166

Anolis_apletophallus_Pleu PEQPFFQ------IANLIPTRQVQIPHYNIRPRI-EQTFPQPEAYPPVDPQQQPQP-TER 167

Anolis_rodriguezii_Pleu PEQPFFQ------IANLIPTRQVQIPHYNIQPRI-EQTFPQPEAYPPVDPQQQPQP-TER 167

Anolis_carolinensis_Pleu PEQPFFQ------ITNLIPTRQVQIPRYAIQPSI-QQTFQQVEAHPPVDPQQQPLP-AER 168

Anolis_sagrei_ordinatus_Pleu PEQPFFQP-----FTNLFPARQLQIPQYNIQPPI-QQTFPQLEAQPAVDPQQQPLP-AER 167

Ctenosaura_bakeri_Pleu QEQPHYQ------VTNLIPTRQVQIQQYDVQPPI-QQKFSPPEGNQPADPQKQPLQ-TER 168

Iguana_delicatissima_Pleu QEQPHYQ------VTNLIPTRQVQIQQYDVQPQI-QQKFPPPEGNQPADPQQQPLP-TER 168

Cyclura_pinguis_Pleu QEQPHYQ------VTNLIPTRQVQVQQYDVQPPI-QQKFPPPEGNQPADPQQQPQP-TER 168

Gambelia_wislizenii_Pleu QEQPHYQ------VTNLIPTRQVQLQQYDVQPPI-QQNFPPPEGNQPINPQQQALP-AER 168

Laemanctus_serratus_Pleur QEQAHYQ------ITNQLPTRQVRLQLYDVQPPI-QQKFPPLEGKQPVDPQQQPLP-AER 168

Phrynosoma_blainvillii_Pleu QEQPHYQ------VLNQIPTRQVWLQLYDGQPPI-QQKFPPPEPNQPVDPQQQLLP-GER 168

Phrynosoma_platyrhinos_Pleu QEQPHYQ------VLNQIPTRQVWLQLYDGQPPI-QQKFPPTEPNQPVDPQQQPLP-GDK 168

Sceloporus_chrysostictus_Pleur QEQPHYQ------VLNQIPTRQVWVHPYDVQPSI-QQQFPPPEANQPVDPQQQTLT-GER 168

Sceloporus_occidentalis_Pleu QEQPPYQ------VLNQIPTRQVWLHPYDVQPSI-QQKFPPPEAKQPVDPQQQTLP-GER 168

Sceloporus_tristichus_Pleu QEQPPYQ------VLNQIPTRQVWLHPYDVQPSI-QQKFPPPEANQPVDPQQQTLP-GER 168

Sceloporus_undulatus_Pleu QEQPPYQ------VLNQIPTRQVWLHPYDVQPSI-QQKFPPPEANQPVDPQQQTLP-GER 168

Urosaurus_nigricaudus_Pleu QEQSHYQ------VLNQIPTRQVWLHPYDVQPSI-QQKFPPPEANQPVDPQQQTLP-GER 168

Canis_lupus_dingo_AMBN -QKPFLQSAIVTDIQDTAQKRGTQPPVYQGQPPL-QQT-EGPMLEQQVAPSDKPPK-AE- 167

Vulpes_vulpes_AMBN -QKPFLQSAIVTDIQDTAQKGGPQPPVYQGQPPL-QQT-EGPMLEQQVAPSDKPPK-AE- 167

Ursus_arctos_AMBN -QKPFLQSAIVTDIRDTAPKGGPQRPVYQGQPPL-QQK-EGPMLEQQVAPSDKPPK-AE- 167

Meles_meles_AMBN -QKPFLQSALVTDVQDTAQKGGPQPPVYQGQPPL-QQA-EGPMVEQQVAPTDKPPK-TE- 167

Mustela_putorius_AMBN -QKPFLQSALLTDGQDTAPKEGPQPPAYQGQPPL-QQA-EGPMLEQQVAPTDKPPK-AE- 167

Phacochoerus_africanus_AMBN -QKPFLQPTVVTSIQNPVQKGVPQPPIYQGHPPL-QQV-EGPMVQQQVAPSEKPPE-AEL 168

Phyllostomus_hastatus_AMBN -QKPFLQPTVVTAIQNTAQKGGPQPPVYQGQPPL-HQE-EGPMIQQQVAPSDKPPK-TE- 167

Homo_sapiens_AMBN -LKPFLQSAAATTNQATALKEALQPPIHLGHLPL-QEG-ELPLVQQQVAPSDKPPK-PEL 168

Saimiri_boliviensis_AMBN -LKPFLQSAAGTANQATAQK-ALQPPIHLGQLLL-QEG-ELPPIEQQVAPSDKPPK-PEL 167

Echinops_telfairi_AMBN -PKPFPVPTDATAIQDPAQKAGPQPPIHLGFPPL-QQA-ESPAILQQVAPSEKPPK---- 147

Suncus_etruscus_AMBN -KKPYLLPTVATAVQNPVQKGGPQPPIYHRQPPLQQQA-EEPIMEQQVAPSDMPPK-EEI 168

Erinaceus_europaeus_AMBN -QKPFHLPTVETAIQNTIQKGGPQLPVYQGHPPL-QQA-EGPMIQQQVAPSDTPPKEAEL 153

:. : : .: * .

Acanthocercus_cyanogaster_Acr L-----TQQ-------------------------------------IFPGLHGTQQNQA- 182

Acanthocercus_minutus_Acr L-----TQQ-------------------------------------IFPGLHGTQQDQA- 182

Xenagama_zonura_Acr L-----TQQ-------------------------------------IFPGLHGTQQDQA- 182

Agama_doriae_AMBN_Acr L-----TQQ-------------------------------------ILPGLHGTQQDQV- 182

Laudakia_wui_Acr L-----IQQX-----------------------XXXXXXXXXXXXXIFPGLHGTQQDQV- 196

Phrynocephalus_forsythii_Acr L-----TQQ-------------------------------------IFPGLHGTQQDQS- 182

Phrynocephalus_guinanensis_Acr L-----TQQX-----------------------XXXXXXXXXXXXXIFPGLHGTQQDQS- 196

Phrynocephalus_vlangalii_Acr L-----TQQ-------------------------------------IFPGLHGTQQDQS- 182

Phrynocephalus_versicolor_Acr L-----TQQ-------------------------------------IFPGLHGTQQGQA- 182

Intellagama_lesueurii_Acr Q-----TQQS-----------------------PTVQEYLGTLGQTIYPGLHGNQQDQA- 199

Pogona_vitticeps_Acr Q-----TQQ-------------------------------------IYLGLHGNQQDQA- 185

Bradypodion_pumilum_Acr Q-----TQQS-----------------------PTIQEYSGALGQTIYPGFHGAQPDQV- 197

Bradypodion_ventrale_Acr Q-----TQQS-----------------------PTIQEYSGALGQTIYPGFHGAQPDQV- 197

Chamaeleo_calyptratus_Acr Q-----TQQS-----------------------PIIQEYSGALGQTIYPGLHGAQTDQV- 197

Chamaeleo_dilepis_Acr Q-----TQQS-----------------------PIIQEYSGALGQTIYPGLHGAQTDQV- 197

Chamaeleo_gracilis_Acr Q-----TQQS-----------------------PIIQEYSGALGQTIYPGLHGAQTDQV- 197

Chamaeleo_laevigatus_Acr Q-----TPQS-----------------------PIIQEYSGALGQTIYPGLHGAQTDQV- 197

Furcifer_pardalis_Acr Q-----TQQS-----------------------STIQEYSGALGQTIYPGLHGAQPDQV- 197

Trioceros_affinis_AMBN_Acr Q-----TQQS-----------------------PIIQEYSGALGQT-------------- 184

Trioceros_balebicornutus_Acr Q-----TQQS-----------------------PIIQEYSGALGQTIYPGLNGAQPDQV- 197

Trioceros_harennae_Acr Q-----TQQS-----------------------PIIQEYSGALGQTIYPGLNGAQPDQV- 197

Anolis_apletophallus_Pleu Q-----TNQ-------------------------------------IYSVLHGTQQDPS- 184

Anolis_rodriguezii_Pleu Q-----TNQ-------------------------------------IYSVLHGTQQDPS- 184

Anolis_carolinensis_Pleu Q-----THQ-------------------------------------IYPALHGTQQDPS- 185

Anolis_sagrei_ordinatus_Pleu Q-----THQ-------------------------------------IYPALHGTQQDPS- 184

Ctenosaura_bakeri_Pleu Q-----TQQL-----------------------STVQERSGTFGQTIYPGLHGTQQDPS- 199

Iguana_delicatissima_Pleu Q-----TQQL-----------------------STVQERSGTFGQTIYPGLHGTQQDPS- 199

Cyclura_pinguis_Pleu Q-----TQQL-----------------------STVQERSGTFGQTIYPGLHGTQQDPS- 199

Gambelia_wislizenii_Pleu QK----SQQL-----------------------STVQEHSGTFGQTIFPGLHGTQQDPS- 200

Laemanctus_serratus_Pleur Q-----TQQL-----------------------STVQERSGTFGQIIYPGLHGTQQDPS- 199

Phrynosoma_blainvillii_Pleu Q-----TQQL-----------------------STVQEHKGTFGQTIYPGLHGTQQDPS- 199

Phrynosoma_platyrhinos_Pleu Q-----TQQL-----------------------STVQEHTRAFGQTIYPGLHGTQQDPS- 199

Sceloporus_chrysostictus_Pleur Q-----TQQIYPGL------------------HGTQQDPS---QQSIYPGLHGTQQDPS- 201

Sceloporus_occidentalis_Pleu Q-----TQQL-----------------------STVQEHTGTFGQTFYPGLHGTQQDPS- 199

Sceloporus_tristichus_Pleu Q-----TQQL-----------------------STVQEHTGTFGQTFYPGLHGTQQDPS- 199

Sceloporus_undulatus_Pleu Q-----TQQL-----------------------STVQEHTGTFGQTFYPGLHGTQQDPS- 199

Urosaurus_nigricaudus_Pleu Q-----TQQL-----------------------STVQEHTGTFGQTFYPGLHGTQQDPS- 199

Canis_lupus_dingo_AMBN ---------------------------------LPGMDFAEPQGPSVFQIARLISRGPM- 193

Vulpes_vulpes_AMBN ---------------------------------LPGMDFAEPQGPSVFQIARLISRGPM- 193

Ursus_arctos_AMBN ---------------------------------LPGMDFADPEGPSVFQIARLISQGPM- 193

Meles_meles_AMBN ---------------------------------LPGMDFAELQGPSVFQIARLISQGPM- 193

Mustela_putorius_AMBN ---------------------------------LPGMDFADLQGPSVFQIARLISRGPM- 193

Phacochoerus_africanus_AMBN HFVRF-DCSV-ILIFLM---------------QLPGLDFADPQDPSMFPIARLISQGPV- 210

Phyllostomus_hastatus_AMBN ---------------------------------LPGMDFAGPQDPSIFTIARLISQGPI- 193

Homo_sapiens_AMBN PGVDFADPQG-PSLPGMDFPDPQGP-------SLPGLDFADPQGSTIFQIARLISHGPM- 219

Saimiri_boliviensis_AMBN PGIDFADPQG-PSLPRMDFADPQDP-------SIPGLDFADPQGPSIFQIARLISRGPM- 218

Echinops_telfairi_AMBN --------------------------------------------PEIFQIARLISQGPV- 162

Suncus_etruscus_AMBN PGMDFAETQG-PG---MDFTVPQDTGMDFAVTQEPGMNFAEPRGPSLFQIARLISRGPL- 223

Erinaceus_europaeus_AMBN PRMDFADPQD-PSLPRMDFADPQDP-------SLPQMDFADPQITSLFPIARLIARGPIA 205

Acanthocercus_cyanogaster_Acr -QSPP--QQS---VYPNLYYMPYVANQGAAPGRLGIVSSEEMQGGGYGAPAYQAPGPDLF 236

Acanthocercus_minutus_Acr -QSPP--QQS---VYPNLYYMPYVANQGAAPGRLGIVSSEEMQGGGYGAPAYQAPGPDLF 236

Xenagama_zonura_Acr -QSPP--QQS---VYPNLYYMPYVANQGAAPGRLGIVSSEEMQGGGYGAPAYQAPGPDVF 236

Agama_doriae_AMBN_Acr -QSPQ--QQS---IYPNLYYMPYVANQGAAPGRLGIVSSEEMQGGGFGPPAYQAPGPDLF 236

Laudakia_wui_Acr -QSSP--QQS---MYPNLYYMPYVANQGAAPGRLGIVSSEEMQGGGFGAPAYQAPGPDLF 250

Phrynocephalus_forsythii_Acr -QSPP--QQS---IHPNLYYMPYIANQGAAPGRLGIVSSEEMQGGGFGAPAYQAPGPDLF 236

Phrynocephalus_guinanensis_Acr -QSPQ--QQS---IHPNLYYMPYAANQGAAPGRLGIVSSEEMQGGGFGAPAYQAPGPDLF 250

Phrynocephalus_vlangalii_Acr -QSPQ--QQS---IHPNLYYMPYVANQGAAPGRLGIVSSEEMQGGGFGAPAYQAPGPDLF 236

Phrynocephalus_versicolor_Acr -QSPQ--QQS---IYPNLYYMPYMANQGAAPGRLGIVSSEEMQGGGFGAPAYQAPGPDLF 236

Intellagama_lesueurii_Acr -QPPP--QQS---IYPNLYYMPYVANQGAAPGRLGIVSSEEMQGGGFGPPAYQAPGPDLF 253

Pogona_vitticeps_Acr -QPPP--QQS---IYPNLYYMPYVANQGAAPGRLGIVSSEEMQGGGFGPPAYQAPGPDLF 239

Bradypodion_pumilum_Acr -QPPQ--QQS---IYPNLYYMPYGANQGAAPGRLGIVSSEEMQGGGFGPPAYQAPGPDLF 251

Bradypodion_ventrale_Acr -QPPQ--QQS---IYPNLYYMPYGANQGAAPGRLGIVSSEEMQGGGFGPPAYQAPGPDLF 251

Chamaeleo_calyptratus_Acr -QQPQ--QTI---IYPSLYYMPYGANQGAAPGRLGIVSSEEMQGGGFGPPAYQAPGPDLF 251

Chamaeleo_dilepis_Acr -QQPQ--XTI---IYPSLYYMPYRANQGAAPGRLGIVSSEEMQGGGFGPPAYQAPGPDLF 251

Chamaeleo_gracilis_Acr -QQPQ--QQP---IYPSLYYMPYGANQGAAPGRLGIVSSEEMQGGGFGPPAYQAPGPDLF 251

Chamaeleo_laevigatus_Acr -QQPQ--XTI---IYPSLYYMPYGANQGAAPGRLGIVSSEEMQGGGFGPPAYQVPGPDLY 251

Furcifer_pardalis_Acr -QQPQ--QQS---IYPNLYYMPYGANQGAAPGRLGIVSSEEMPGGGFGPPAYQAPGPDLF 251

Trioceros_affinis_AMBN_Acr -------------IYPNLYYMPYGTNQGAVPGRLGIVSSEEMQGGGFGPPAYQAPGPDLF 231

Trioceros_balebicornutus_Acr -QQPQ--XTI---IYPNLYYMPYGANQRAASGRLGIVSSEEMQGGGFGPPAYQAPGPDLF 251

Trioceros_harennae_Acr -QQPQ--XTI---IYPNLYYMPYGANQGAAPGRLGIVSSEEMQGGGFGPPAYQALGPDLF 251

Anolis_apletophallus_Pleu QQQQQ--QQS---MYPSLYYLPYVANQGAAPARLGIVSSEEMQGGGFGAPAYRAAGPDLF 239

Anolis_rodriguezii_Pleu -QQQQ--QQS---MYPSLYYLPYVANQGAAPARLGIVSSEEMQGGGFGAPAYRAAGPDLF 238

Anolis_carolinensis_Pleu -QQLQ--QQS---IYPSLYYMPYVANQGAAPARQGIVSSEEMQGGGFGAPAYRAPGPDLF 239

Anolis_sagrei_ordinatus_Pleu -QQQQ--QQQ--PMYPSLYYLPYVANQGAAPARLGIVSSEEMQGGGYGAPAYRAAGPDLF 239

Ctenosaura_bakeri_Pleu -QQLQ--QRS---MYPHLYYLPYVANQGGAPARLGIVSSEEMQGGGFGAPAYQAAGTDPF 253

Iguana_delicatissima_Pleu -QQLQ--QRS---VYPHLYYLPYVANQGGAPARLGIVSSEEMQGGGFGAPAYQAAGTDPF 253

Cyclura_pinguis_Pleu -QQLQ--QRS---MYPHLYYLPYVANQGGAPARLGIVSSEEMQGGGFGAPAYQAAGTDPF 253

Gambelia_wislizenii_Pleu -QQLQ--QQS---MYPNLYYLPYVANQGVAPGRLGIVSSEEMQGGGFGAPAYRAAGTDLF 254

Laemanctus_serratus_Pleur -QQPQ--QRP--VMYPSLYYLPYVANQGGAPARMGIVSSEEMQGGGFGAPAYRAAGPDFY 254

Phrynosoma_blainvillii_Pleu -------QRS---MLPNLYYLPYVANQGGTPARIGIVSSEEMQGGGFGAPAYRAAGTDLF 249

Phrynosoma_platyrhinos_Pleu -------QRS---MLLNLYYLPYVANQGGTPARMGIVSSEEMQGGGFGAPAYRAAGTDLF 249

Sceloporus_chrysostictus_Pleur -------QQS---MLPSLYYLPYVANQGGAPARMGIVSSEEMQGGGFGAPAYRAAGTDLF 251

Sceloporus_occidentalis_Pleu -------QRS---MLPNLYYLPYVANQGGAPARMGIVSSEEMQGGGFGAPAYRAAGTDLF 249

Sceloporus_tristichus_Pleu -------QRSVSLMLPNLYYLPYVANQGGAPARMGIVSSEEMQGGGFGAPAYRAAGTDLF 252

Sceloporus_undulatus_Pleu -------QRSVSLMLPNLYYLPYVANQGGAPARMGIVSSEEMQGGGFGAPAYRAAGTDLF 252

Urosaurus_nigricaudus_Pleu -------QRSVSLMLPSLYYLPYIANQGGAPARMGIVSSEEMQGGGFGAPAYRAAGTDLF 252

Canis_lupus_dingo_AMBN -PQNK--PSP---LYPGIFYMSYGANQLNAPGRLGIMSSEEMAGGRGSPMAYGAMFPGFG 247

Vulpes_vulpes_AMBN -PQNK--PSP---LYPGIFYMSYGANQLNAPGRLGIMSSEEMAGGRGSPMAYGAMFPGFG 247

Ursus_arctos_AMBN -PQNK--PSP---LYPGIFYMSYGANQLNAPGRLGIMSSEEMAGGRGGPMAYGAMFPGFG 247

Meles_meles_AMBN -PQNK--PSP---LYPGIFYMSYGANQLNAPARLGIMSSEEMAGGRGGPMAYGAMFPGFG 247

Mustela_putorius_AMBN -PQNK--PSS---LYPGIFYMSYGANQLNAPARLGIMSSEEMAGGRGSPMAYGAIFPGFG 247

Phacochoerus_africanus_AMBN -PQDK--PSP---LYPGMFYMSYGANQLNAPARLGILSSEEMAGGRGGPLAYGAMFPGFG 264

Phyllostomus_hastatus_AMBN -PQNK--PSP---LFPGMFYMSYGANQLSAPTRLGIMSSEEMMGGRGGPMTYGAMFPGFG 247

Homo_sapiens_AMBN -PQNK--QSP---LYPGMLYVPFGANQLNAPARLGIMSSEEVAGGREDPMAYGAMFPGFG 273

Saimiri_boliviensis_AMBN -PQNK--QSP---LYSGMFYVPFGANQLNAPARLGIMSSEEMAGGRGGPMGYGAMFPGFG 272

Echinops_telfairi_AMBN -PPNKQSQSP---LYPGMFYMSYGANQLNAPGRLGIMSSEEMAGGRGGPMAYGAMFPGFE 218

Suncus_etruscus_AMBN -SQNK--AAS---LYPGIFYMSYGANQLNAPGRIGFMSSEEMPGGRGSPMAYGSMFSRLG 277

Erinaceus_europaeus_AMBN VPQNK--PSS---LFPGMFYMSYGANQLNAPGRLGIMSSEEMPGGREGPTAYGAMFPGFR 260

: : *:.: :** .. * *::****: ** .. * .

Acanthocercus_cyanogaster_Acr GTDARLRSM--ALNQP--GDYTVE-DDQLGITEQPNVKGEPNVG------------AN-- 277

Acanthocercus_minutus_Acr GTDARLRSM--ALNQP--GDYTVE-DDQLGITEQPNVKGEPNVG------------AN-- 277

Xenagama_zonura_Acr GTDARLRSM--ALNQP--GDYTVE-DDQLGITEQPNVKGEPNVG------------AN-- 277

Agama_doriae_AMBN_Acr GTDARLRSM--ALNQP--GDYTVE-DDQLGITEQPNVKGEPNVG------------AN-- 277

Laudakia_wui_Acr GTDARLRSM--ALNQP--GDYTVE-DDQLGITEQPNVKGEPNVG------------AN-- 291

Phrynocephalus_forsythii_Acr GTDARLRSM--VLNQP--GDYTVE-DDQLGITEQPNVKGEPNVG------------AN-- 277

Phrynocephalus_guinanensis_Acr GTDARLRSM--ALNQP--GDYTVE-DDQLGITEQPNVKGEPNVG------------AN-- 291

Phrynocephalus_vlangalii_Acr GTDARLRSM--ALNQP--GDYTVE-DDQLGITEQPNVKGEPNVG------------AN-- 277

Phrynocephalus_versicolor_Acr GTDARLRSM--ALNQP--GDYTVE-DDQLGITEQPNVKAEPNVG------------AN-- 277

Intellagama_lesueurii_Acr ATDARLRHM--ALNQP--GDYTVE-DDQLGITEQPDVKGGANVG------------AN-- 294

Pogona_vitticeps_Acr ATDARLRHM--ALNQP--GDYTVE-DDQLGITEQPDVKGGANVG------------AN-- 280

Bradypodion_pumilum_Acr ATGARLRDM--ALNQP--GDYTVE-DDQLGITEQPDVKGGANAG------------AN-- 292

Bradypodion_ventrale_Acr ATGARLRDM--ALNQP--GDYTVE-DDQLGITEQPDVKGGANAG------------AN-- 292

Chamaeleo_calyptratus_Acr ATGARLRDM--ALNQP--GDYTVE-DDQLGITEQPDVKGGANAG------------AN-- 292

Chamaeleo_dilepis_Acr ATGARLRDM--ALNQP--GDYTVE-DDQLGITEQPDVKAGANAG------------AN-- 292

Chamaeleo_gracilis_Acr ATAAKLRDM--ALNQP--GDYTVE-DDQLGITEQPDVKGGANAG------------AN-- 292

Chamaeleo_laevigatus_Acr ATGARLRDM--ALNQP--GDYTVE-DDQLGITEQPDVKGGANAG------------AN-- 292

Furcifer_pardalis_Acr ATGARLRDM--ALNQP--GDYTVE-DDQLGITEQPDVKGGANAG------------AN-- 292

Trioceros_affinis_AMBN_Acr ATGARLRDM--ALNQP--GDYTVE-DDQLGITEQPDVKGGANAG------------AN-- 272

Trioceros_balebicornutus_Acr ATGARLRDM--ALNQP--GDYTVE-DDQLGITEQPDVKGGANAG------------AN-- 292

Trioceros_harennae_Acr ATGARLRDM--ALNQP--GDYTVE-DDQLGITEQPDVKGGANAG------------AN-- 292

Anolis_apletophallus_Pleu PMDTRFGNS--PLNQP--GDYTVE-DDQLGITEQPDVKGGANAG------------AG-- 280

Anolis_rodriguezii_Pleu PMDTRFGNS--PLNQP--GDYTVE-DDQLGITEQPDVKGGANAG------------AG-- 279

Anolis_carolinensis_Pleu AMDTRFGNS--PLNQP--GDYTVE-DDTLGITEQPDVKGGANAG------------AN-- 280

Anolis_sagrei_ordinatus_Pleu PMDTRFGNS--PLNQP--GDYTVE-DDQLGITEQPDVKGGANAG------------AN-- 280

Ctenosaura_bakeri_Pleu AMNARFGNS--PLNQP--GDYTVE-DDQLGITEQPDVPGGAKAG------------TN-- 294

Iguana_delicatissima_Pleu AMNARFENS--PLNQP--GDYTVE-DDQLGITEQPDMPGGAKAG------------TN-- 294

Cyclura_pinguis_Pleu AMNARFGNS--PLNQP--GDYTVE-DDQLGITEQPDVRGGAKAG------------TN-- 294

Gambelia_wislizenii_Pleu AMDARFGNS--PLNQP--GDYTVE-DDQLGITEQPDVQGGANAG------------TN-- 295

Laemanctus_serratus_Pleur PMDTRFGNS--PLNQP--GDYTVE-DDQLGITEQPDVQGGANAG------------TN-- 295

Phrynosoma_blainvillii_Pleu AMNARFGNS--PLNQP--GDYTVE-DDQLGITEQPDVKGGANAG------------TN-- 290

Phrynosoma_platyrhinos_Pleu AMDARFGNS--PLNQP--GDYTVE-DDQLGITEQPDVKGGANAG------------TN-- 290

Sceloporus_chrysostictus_Pleur AMDARFGNS--PLNQP--GDYTVE-DDQLGITEQPDVKGGANAG------------TN-- 292

Sceloporus_occidentalis_Pleu AMDARFGNS--PLNQP--GDYTVE-DDQLGITEQPDVKGGANAG------------TN-- 290

Sceloporus_tristichus_Pleu AMDARFGNS--PLNQP--GDYTVE-DDQLGITEQPDVKGGANAG------------TN-- 293

Sceloporus_undulatus_Pleu AMDARFGNS--PLNQP--GDYTVE-DDQLGITEQPDVKGGANAG------------TN-- 293

Urosaurus_nigricaudus_Pleu AMDARFGNS--PLNQP--GDYTVE-DDQLGITEQPDVKGGANAG------------TN-- 293

Canis_lupus_dingo_AMBN GMRPNLGGM--PHNPGMGGDFTLEFDSPVAGTKGPEKGEGGAQG------SPM-PDVN-- 296

Vulpes_vulpes_AMBN GMRPNLGGM--PHNPGMGGDFTLEFDSPVAGTKGPEKGEGGAQG------SPM-PDVN-- 296

Ursus_arctos_AMBN GMRPNLGGM--PHNPAMGGDFTLEFDSPVAGTKGPEKGEGGAQG------SPM-PDGD-- 296

Meles_meles_AMBN GMKPNLGGM--PHNPAMGGDFTLEFDSPVAGTKGPEKGEGGAQG------SPL-TEVD-- 296

Mustela_putorius_AMBN GMRPHLGGM--PHNPAMGGDFTLEFDSPVAGTKGPEKGEGGAQG------SPL-TELD-- 296

Phacochoerus_africanus_AMBN GMRPNLGGM--PPNSAKGGDFTLEFDSPAAGTKGPEKGEGGAEG------SPA-AEAN-- 313

Phyllostomus_hastatus_AMBN GMRPSLGGM--PHHPGMGGDFTLEFDTPMAATKGPEKGEGVVHS------SPVPPEAN-- 297

Homo_sapiens_AMBN GMRPGFEGM--PHNPAMGGDFTLEFDSPVAATKGPENEEGGAQG------SPM-PEAN-- 322

Saimiri_boliviensis_AMBN GMRPSFGGM--PHNPAMGGDLTLEFDSPVAATKSPEKVEGGAQG------SPM-PEAN-- 321

Echinops_telfairi_AMBN GIRPGFGGM--QPNQGKGGDFTLEFDTPFA-TKGPEKGEGGAHG------APM-PEAN-- 266

Suncus_etruscus_AMBN GLRPNLAGLPKPQNPAKGGDFTLEFDTPVSVTKGPEKGEGGAQY------PTV-EKGEEE 330

Erinaceus_europaeus_AMBN GMRPNFGRM--HQNSPKGGDFTVEFDTP-AVTKGQEKGEGGKEGEGDPHHSRL-PERN-- 314

. : : ** *:* * . *: :

Acanthocercus_cyanogaster_Acr ---PPGRGNPIHPLE----GSPVIGSLP----NANGYLLNPAGQSKGPLGDPQATVGSLA 326

Acanthocercus_minutus_Acr ---PSGRGNPIHPLE----GSPVIGSLP----NANGYLLNPAGQSKGPLGDPQATVGSLA 326

Xenagama_zonura_Acr ---PSGRGNPIHPLE----GSPVIGSLP----NANGYLLNPAGQSKGPLGDPQATVGSLA 326

Agama_doriae_AMBN_Acr ---PAGRGNPIHPLE----GSPVVGASP----NANGYLLNPAGQSKGPLGDPQATVGSLA 326

Laudakia_wui_Acr ---PTGRGNPIHPLE----GNPVIGASP----NANGYLLNPAGQSKGPLGDPQATVGSLA 340

Phrynocephalus_forsythii_Acr ---PTGRGNPIHPLE----GNPVIGASP----NADGYLLNPAGQSKGPLGDPQATVSSLA 326

Phrynocephalus_guinanensis_Acr ---PTGRGNPIHPLD----GNPVIGASP----NADGYLLNPAGQSKGPLGDPQATVSSLA 340

Phrynocephalus_vlangalii_Acr ---PTGRGNPIHPLE----GNPVIGASP----NADGYLLNPAGQSKGPLGDPQATVSSLA 326

Phrynocephalus_versicolor_Acr ---PTGRGNPIHPLE----GNPVIGASP----NADGYLLNPAGQSKGPLGDPQVTVSSLA 326

Intellagama_lesueurii_Acr ---PAGKGNPINPLE----GNPAIGASP----NANSYLLHPAGQSKGPLGEPQATGGPLA 343

Pogona_vitticeps_Acr ---PAGKGNPISPLE----GNPAIGASP----NANSYLLHPAGQSKGPLGEPQATGGPLA 329

Bradypodion_pumilum_Acr ---PSGQGNPIIPIE----GNPAVGASP----NVNHYLLNPAGQSKGPVGAPYGTAGPLA 341

Bradypodion_ventrale_Acr ---PSGQGNPIIPVE----GNPAVGASP----NVNHYLLNPAGQSKGPVGAPYGTAGPLA 341

Chamaeleo_calyptratus_Acr ---PSGQGNPIIPIE----GNPAVGTSA----NVNHYLLNPAGQSKGPVVAPYGTPGPLA 341

Chamaeleo_dilepis_Acr ---PSGQGNPIIPVE----GNPAVGASA----NVNHYLLNPAGQSKGPVGAPYGTAGPLA 341

Chamaeleo_gracilis_Acr ---PSGQGNPIIPVE----GNPAVGASA----NVNHYLLNPAGQSKGPVGAPYGTAGPLA 341

Chamaeleo_laevigatus_Acr ---PSGQGNPVIPAE----GNPAVGGSA----NVNHYLLNPAGQSKGPVGAPYGTAGPLA 341

Furcifer_pardalis_Acr ---PSGQGNPIIPIE----GNPAVGASA----NVNHYLLNPAGQSKGPGGAPHGTAGPLA 341

Trioceros_affinis_AMBN_Acr ---PSGQGNPIIPVE----GNPAVGASA----NVNHYLLNPAGQSKGPVGAPYGTAGPLA 321

Trioceros_balebicornutus_Acr ---PSGQGNPIIPVE----GNPAVGASA----NVNHYLLNPAGQSKGPVGAPYGTAGPLA 341

Trioceros_harennae_Acr ---PSGQGNPIIPVE----GNPAVGASA----NVNHYLLNPAGQSKGPAGAPYGTAGPLA 341

Anolis_apletophallus_Pleu ---PSGKGNNAVLLD----GTQG-GSLP----NANSHLFNPAGQSQGLSGLAQATVSPHA 328

Anolis_rodriguezii_Pleu ---PSGKGNNAVLLD----GTQG-GALP----NANSHLFNPAGQSQGLSGLAHATVSPHA 327

Anolis_carolinensis_Pleu ---PSGKGNNAILLD----GTQG-GALP----NANSHLFSPAGQSQGLSGLAQATAAPHA 328

Anolis_sagrei_ordinatus_Pleu ---PSGKGNNAVLLD----GTQG-GALP----NPNSHLFNPAGQSQGLSGLAQATMAPHA 328

Ctenosaura_bakeri_Pleu ---PAARGNNAIPVD----G----AALP----NANSHLLSPAGQSQGLSGVAQATAVPLA 339

Iguana_delicatissima_Pleu ---PAARGNSAIPVD----G----AALP----NANSHLLSPAGQSQGLSGVAQATTVPLA 339

Cyclura_pinguis_Pleu ---PAARGNNAIPVD----G----AALP----NANSHLLGPAGQSQ---GVTQATAVPLA 336

Gambelia_wislizenii_Pleu ---PSGKGNNAILAN----GNQD-GALP----NANSHLLNPAGQSQGLSGVARDTAVPLA 343

Laemanctus_serratus_Pleur ---PSGKGTNAILVD----GNQ--GALP----NANSHLFSPAGQSQGLSGVAQATAAPLA 342

Phrynosoma_blainvillii_Pleu ---PAGQGNNVIPID----GSQS-GTLP----NANSHLLSPAGQSQGLSGIAQATEAPLP 338

Phrynosoma_platyrhinos_Pleu ---PAGQGNNVIPID----GSQG-GALP----NAHSHLLSPAGQSQGLSGVAQATAAPLP 338

Sceloporus_chrysostictus_Pleur ---PSGQGNNGIPID----GSQG-GALP----NANSHLLSPAGQSQGLSGVAQATGVPLP 340

Sceloporus_occidentalis_Pleu ---PSGQGNNAIPVD----GSQG-GALL----NANSHLLSPAGQSQGLSGVAQATGIPLP 338

Sceloporus_tristichus_Pleu ---PSGQGNNAIPVD----GSQG-GALL----NANSHLLSPAGQSQGLSGVAQATGIPLP 341

Sceloporus_undulatus_Pleu ---PSGQGNNAIPVD----GSQG-GALL----NANSHLLSPAGQSQGLSGVAQATGIPLP 341

Urosaurus_nigricaudus_Pleu ---PSGQGNNAIPVD----GSQG-GALP----NANSHLLSPAGQSQGLSGVIQATGVPLP 341

Canis_lupus_dingo_AMBN ---PANPENPALLTE-LAPGALG-GLLAHPKDNDPSLARGPAGQSGGPPRVTPADADPLM 351

Vulpes_vulpes_AMBN ---PANPENPALLTE-VAPGALG-GLLAHPKDNDPSLARGPAGQSGGPPRVTPADADPLM 351

Ursus_arctos_AMBN ---PANLENPDLLPG-VAPGALG-GLLAHPKDDEPGLARGPAGQSGGPPRVTPADADPLM 351

Meles_meles_AMBN ---PANPENPALLPE-VAPGALG-GLLAHPKGNDPNMARGPAGQIGGPLRVTPADADPLM 351

Mustela_putorius_AMBN ---PANPESPALLPE-GAPGALG-GLLAHPKGNDPNMARGPAGQSGGPLRVTPADADPLM 351

Phacochoerus_africanus_AMBN ---TADPESPALFSE-VASGVLG-GLLANPKGKIPNLARGPAGRSRGPPGVTPADADPLM 368

Phyllostomus_hastatus_AMBN ---PVNPENPVLLPEAVVPGALG-GLLALPKDNIPSLTGDPTEDNNGSLRVTPAAADPLM 353

Homo_sapiens_AMBN ---PDNLENPAFLTE-LEPAPHA-GLLALPKDDIPGLPRSPSGKMKGLPSVTPAAADPLM 377

Saimiri_boliviensis_AMBN ---PDNPENPAVFPD-LEPAAHA-GLLALPKDDVSSLPRSPSGKRKGALSVTPAAADPLM 376

Echinops_telfairi_AMBN ---PANAENPALLSE-VAPGAHG-ALLAFPKGLFPSLARGPAGQSRGPLRVTPEVADPMV 321

Suncus_etruscus_AMBN TQPPTNRENRAFLPE-VSYGALG-GLLAPPKDHIPSQARGPAGKNWGFPEVTADGVDPLM 388

Erinaceus_europaeus_AMBN ---RLSPENPALFQEAAALEALG-GFLASPKDNIPSLAMGPAGLIREPSKITPEAADPLM 370

. . *: .

Acanthocercus_cyanogaster_Acr PEGLP----VLDAAGTAMPL--------DPTMFPDTMFPGNAGNEAGLSPIG-QDMWHFQ 373

Acanthocercus_minutus_Acr PEGLP----VLDAAGTAMPL--------DPTMFPDTMFPGNAGNEAGLSPIG-QDMWHFQ 373

Xenagama_zonura_Acr PEGLP----VLDAAGTAMPL--------DPTMFPDTMFPGNAGNEAGLSPIG-QDMWHFQ 373

Agama_doriae_AMBN_Acr PEGLP----VLDAAGTAMPL--------DPTPFPDTMFPANAGNEAGLSPIG-QDMWHFQ 373

Laudakia_wui_Acr PEGLP----VMDAAGTAMPL--------DPTTFPDTMFPASAGNEAGLSPIG-HDMWHFQ 387

Phrynocephalus_forsythii_Acr PEGLP----VWDAAGTAMPL--------DPTTFPDTMFPASSGNEAGLSPIG-QDMWHFQ 373

Phrynocephalus_guinanensis_Acr PEGLP----VWDAAGTAMPL--------DPTTFPDTMFPASSGNEAGLSPIG-QDMWHFQ 387

Phrynocephalus_vlangalii_Acr PEGLP----VWDAAGTAMPL--------DPTTFPDTMFPASSGNEAGLSPIG-QDMWHFQ 373

Phrynocephalus_versicolor_Acr PEGLP----VLDAAGTAMPL--------DPTTFPDTMFPASSGNEAGLSPIG-QDMWHFQ 373

Intellagama_lesueurii_Acr PEGLP----VLDAAGTTMPL--------DPTMFPDTMFPTDVGNGAGLSPIG-QDMWNFQ 390

Pogona_vitticeps_Acr PEGLP----VVDAAGTAMPL--------DPTTFPDTMFPTDVGNGAGLSPIG-QDMWHFQ 376

Bradypodion_pumilum_Acr PEGLP----VLEVAGTTIPL--------DPTTFPDTTFPTNVRNEAGLSQTG-QDLWYFQ 388

Bradypodion_ventrale_Acr PEGLP----VLEVAGTTIPL--------DPTTFPDTTFPTNVGNEAGLSQTG-QDLWYFQ 388

Chamaeleo_calyptratus_Acr PEGLP----VLEVAGTTIPL--------DPTMFPDTTFPTDVGNEAGLSQTG-QDSWYFQ 388

Chamaeleo_dilepis_Acr PEGLP----VLEVAGTTIPL--------EPTMFPDTTFPTDVGNEPGLSQTG-QDLWYFQ 388

Chamaeleo_gracilis_Acr PEGLP----VLEAAGTTIPL--------EPTMFPDTTFPTDVGNEAGLSQTG-QDLWYFQ 388

Chamaeleo_laevigatus_Acr PEGLP----VLEVAGTTIPL--------DPTMFPDTTFPTDVGNEAGLSQTG-QDLWYFQ 388

Furcifer_pardalis_Acr PEGLP----VLEVAGTTIPL--------DPTMFPDTTFPTDVGNEPGLSQTG-QDLWYFQ 388

Trioceros_affinis_AMBN_Acr PSGLP----VLEVAGTAIPL--------DPTMFPDTTFPNDVGNEAGLSQTG-QDLWYFQ 368

Trioceros_balebicornutus_Acr AAGLP----VLEVAGTAIPL--------DPTIFPDTTFPNDVGNEAGLSQTG-QDLWYFQ 388

Trioceros_harennae_Acr AAGLP----VLEVAGTAIPL--------DPTIFPDTTFPNDVGNEAGLSQTG-QDLWYFQ 388

Anolis_apletophallus_Pleu TQG---PFLPLDAADPSMPL--------DSTMFPD-LYTTNVGNEAGLAPVG-QDAWHFQ 375

Anolis_rodriguezii_Pleu TQG---PFLPLDAADPSMPL--------DSTMFPD-LYTTNVGNEAGLAHVG-QDAWHFQ 374

Anolis_carolinensis_Pleu TQG---PFLPLDAADPSMPL--------DAPMFPD-LYTSNVGNEAGLAQVG-QDAWHFQ 375

Anolis_sagrei_ordinatus_Pleu TQG---PFLSLDAADPSMPL--------DSTMFPD-LYTTNVGNEPGLAHVG-QDAWHFQ 375

Ctenosaura_bakeri_Pleu PQGAPDSFLPLDAADPTGPL--------DPTMFPD-VYTTNAGNEAGMARVG-QDAWHFQ 389

Iguana_delicatissima_Pleu PQGAPDSFLPLDAADPTGPL--------NPTMFPD-VYTTNAGNEAGMARVG-QDTWRFQ 389

Cyclura_pinguis_Pleu PQGGPGSFLPLDATDPTGPL--------DPTMFPD--YTTNAGNEAGMAHVG-QDAWHFQ 385

Gambelia_wislizenii_Pleu TQGTPGSFLPLDAVDPTMPL--------DPTMLPD-IYTTNVGNEAGLAHVG-QDVWHFQ 393

Laemanctus_serratus_Pleur TQGPPGNFLPLDAADPTMPF--------DPTAFPD-LYTTNAGNEAGLAHVG-QDVWHFQ 392

Phrynosoma_blainvillii_Pleu TQGLPGSFLPLDSADPTMPL--------DPTIFPD-IYTTNAGNEAGLAQVG-QDVWHFQ 388

Phrynosoma_platyrhinos_Pleu TQGLPGGFLPLDSADPTIPL--------DPTTFPD-IYTTNSGNEAGLAQVG-QDVWHFQ 388

Sceloporus_chrysostictus_Pleur TQGLPGSFLPLDSADPTIPL--------DPTMFPD-IYTANAGNEAGLAQVG-QDAWHFQ 390

Sceloporus_occidentalis_Pleu TQGLPGSFLPLDSADPTMPL--------DPTMFPD-TYTANAGNEAGMAQVG-QDVWHFQ 388

Sceloporus_tristichus_Pleu TQGLPGSFLPLDSADPTMSL--------DPTMFPD-TYTANAGNEAGMAQVG-QDVWHFQ 391

Sceloporus_undulatus_Pleu TQGLPGSFLPLDSADPTMSL--------DPTMFPD-TYTANAGNEAGMAQVG-QDVWHFQ 391

Urosaurus_nigricaudus_Pleu TQGLPGSFLPLDSADPTMPL--------DPTMFPD-IYTANAGNEAGMAQVG-QDAWHFQ 391

Canis_lupus_dingo_AMBN TPELADIYETY-GADVTTPL---EETPTDTTVIPDTQQTLMPENKAQQPQIM-HDGWHFQ 406

Vulpes_vulpes_AMBN TPELADIYETY-GADVTTPL---EETPTDTTVIPDTEQTLMPENKAQQPQIM-HDGWHFQ 406

Ursus_arctos_AMBN TPGLADLYETY-GADVTTPL---EETPTDTTAVPDTQQTLMPESKAQQPQIM-HDVWHFQ 406

Meles_meles_AMBN TPGLADIYENY-GADVTTPL---EETPTDTTAIPDTQQTSMPENNAQQPQIM-HDVWHFQ 406

Mustela_putorius_AMBN TPGLADIYENY-GADVTTPL---EETPTDTTASPDTQQTLMPENNAQQPQVM-HDVWRFQ 406

Phacochoerus_africanus_AMBN TPGLADAYETY-GADETTTLGLQEEMTMDSTVTPYSEHTSMPGNKAQQPQIK-HDAWRFQ 426

Phyllostomus_hastatus_AMBN TPGLADVYEPY-SVDMTTPL---GETTMDTTVTPDTEQTSMPGNKAQQPHIM-QNVWHFQ 408

Homo_sapiens_AMBN TPELADVYRTY-DADMTTSVDFQEEATMDTTMAPNSLQTSMPGNKAQEPEMM-HDAWHFQ 435

Saimiri_boliviensis_AMBN TPELADVYGTY-GADMTTSMDFQEEATMDTTMAPNSLQTSMPGNKAQEPEMM-HDTWHFQ 434

Echinops_telfairi_AMBN TPGLADVYGTY-GADMTTPLGLQGEGTTDPTMTPDVSQTSAQGNKAEQPPNM-HKGWHFQ 379

Suncus_etruscus_AMBN TPGLADIYEPY-GADVTTPLGLQGESTTDTTMSPDTEQTAMPENKSQKAQIIHHNAWHFP 447

Erinaceus_europaeus_AMBN TPGLADVYETY-GADMTTPLNLQGESTTDSTTTMDIQETSTPGNKEEQPQVM-HDAWRFQ 428

. .. : .. :.. . . . :. * *

Acanthocercus_cyanogaster_Acr EP 375

Acanthocercus_minutus_Acr EP 375

Xenagama_zonura_Acr EP 375

Agama_doriae_AMBN_Acr EP 375

Laudakia_wui_Acr EP 389

Phrynocephalus_forsythii_Acr EP 375

Phrynocephalus_guinanensis_Acr EP 389

Phrynocephalus_vlangalii_Acr EP 375

Phrynocephalus_versicolor_Acr EP 375

Intellagama_lesueurii_Acr EP 392

Pogona_vitticeps_Acr EP 378

Bradypodion_pumilum_Acr AP 390

Bradypodion_ventrale_Acr AP 390

Chamaeleo_calyptratus_Acr AP 390

Chamaeleo_dilepis_Acr AP 390

Chamaeleo_gracilis_Acr AP 390

Chamaeleo_laevigatus_Acr AP 390

Furcifer_pardalis_Acr AP 390

Trioceros_affinis_AMBN_Acr AP 370

Trioceros_balebicornutus_Acr AP 390

Trioceros_harennae_Acr AP 390

Anolis_apletophallus_Pleu EP 377

Anolis_rodriguezii_Pleu EP 376

Anolis_carolinensis_Pleu EP 377

Anolis_sagrei_ordinatus_Pleu EP 377

Ctenosaura_bakeri_Pleu EP 391

Iguana_delicatissima_Pleu EP 391

Cyclura_pinguis_Pleu EP 387

Gambelia_wislizenii_Pleu EP 395

Laemanctus_serratus_Pleur EP 394

Phrynosoma_blainvillii_Pleu EP 390

Phrynosoma_platyrhinos_Pleu EP 390

Sceloporus_chrysostictus_Pleur EP 392

Sceloporus_occidentalis_Pleu EP 390

Sceloporus_tristichus_Pleu EP 393

Sceloporus_undulatus_Pleu EP 393

Urosaurus_nigricaudus_Pleu EP 393

Canis_lupus_dingo_AMBN EP 408

Vulpes_vulpes_AMBN EP 408

Ursus_arctos_AMBN EP 408

Meles_meles_AMBN EP 408

Mustela_putorius_AMBN EP 408

Phacochoerus_africanus_AMBN EP 428

Phyllostomus_hastatus_AMBN EP 410

Homo_sapiens_AMBN EP 437

Saimiri_boliviensis_AMBN EP 436

Echinops_telfairi_AMBN EP 381

Suncus_etruscus_AMBN EP 449

Erinaceus_europaeus_AMBN EP 430

*

**AMBN-AA-MAFFT-Iguania+Mammal – DIVERGE-Acro-Pleuro DIVERGE-Agam-CHam**

CLUSTAL W (1.8) multiple sequence alignment (ALTER 1.3.3)

Acanthocercus_cyanogaster_Acr MKQWILVSSLLGICSALPILPQHAGIPGIGSISLETMRQMAGQNMPNGLSQYAMPYSYGG 60

Acanthocercus_minutus_Acr MKQWILVSSLLGICSALPILPQHAGIPGIGSISLETMRQMAGQNMPNGLSQYAMPYSYGG 60

Xenagama_zonura_Acr MKQWILVSSLLGICSALPILPQHAGIPGIGSISLETMRQMAGQNMPNGLSQYVMPYSYGG 60

Agama_doriae_AMBN_Acr MKQWILVSSLLGICSALPILPQHAGIPGLGSISLETMRQMGGQNMPNGLSQYAMPYSYGG 60

Laudakia_wui_Acr MKQWILVSSLLGICSALPILPQHAGIPGIGSISLETMRQMGGQNMPNGLPQYAMPYRYGG 60

Phrynocephalus_forsythii_Acr MKQWILVSSLLGLCSALPILPQHAGIPGIGSISLETMRQMSGQNIPHGLPQYAMPYSYGG 60

Phrynocephalus_guinanensis_Acr MKQWILVSSLLGLCSALPILPQHAGIPGIGSISLETMRQMSGQNIPHGLPQYAMPYSYGG 60

Phrynocephalus_vlangalii_Acr MKQWILVSSLLGLCSALPILPQHAGIPGIGSISLETMRQMSGQNIPHGLPQYAMPYSYGG 60

Phrynocephalus_versicolor_Acr MKQWILVSSLLGLCSALPILPQHAGIPGIGSISLETMRQMSGQNIPHGLPQYAMPYSYGG 60

Intellagama_lesueurii_Acr MKQWILVSSLLGICSALPILPQHAGIPGIGSISLETMRQMGGRNGPNALSQYAMPYSYGG 60

Pogona_vitticeps_Acr MKQWILVSSLLGICSALPILPQHAGIPGLGSISLETMRQMGGRNGPNALSQYAMPYSYGD 60

Bradypodion_pumilum_Acr MKQWILVSSLLGICSALPILPQHAGVPGMGSISLETMRQMSGGNIPSVLQQYAMPYNYAG 60

Bradypodion_ventrale_Acr MKQWILVSSLLGICSALPILPQHAGVPGMGSISLETMRQMSGGNIPSVLQQYAMPYNYAG 60

Chamaeleo_calyptratus_Acr MKQWILVSSLLGICSAVPILPQHSGIPGMGSISLETMRQMSGGNIPSVLPQYAMPYNYAG 60

Chamaeleo_dilepis_Acr MKQWILLSSLLGICSALPILPQHSGIPGMGSISLETMRQMSGGNIPSVLPQYAMPYNYAG 60

Chamaeleo_gracilis_Acr MKQWILLSSLLGICSALPILPQHSGIPGMGSISLETMRQMSGGNIPSVLPQYAMPYNYAG 60

Chamaeleo_laevigatus_Acr MKQWIFVSCLLGICSALPILPQHSGIPGMGSISLETMRQMSGGNIPNVLPQYAMPYNYAG 60

Furcifer_pardalis_Acr MKQWILVSSLLGICSALPILPQHAGIPGMGSISLETMRQMSGGNIPSVLPQYAMPYNYGG 60

Trioceros_affinis_AMBN_Acr MKQWILVSSLLGICSALPILPQHAGIPGMGSISFETMRQMSGGNIPSVLPQYAMPYNYPG 60

Trioceros_balebicornutus_Acr MKQWILFSSLLGICYALPILPQHAGIPGMGSISLETMRQMSGGNIPNVLPQYAMPYNYPG 60

Trioceros_harennae_Acr MKQWILFSSLLGICSALPILPQHAGIPGMGSISLETMRQMSGGNIPNVLPQYAMPYNYPG 60

Anolis_apletophallus_Pleu MKQWMLVSSLLGICSALPLFPQHAGIPGMGSISLERTRQLGGLNMPPAPPQ-ALPYSYAD 59

Anolis_rodriguezii_Pleu MKQWMLVSSLLGICSALPLFPQHAGIPGMGSISLERTRQLGGLNMPPAPPQ-ALPYSYAD 59

Anolis_carolinensis_Pleu MKQWMLVSSLLGICSALPLFPQHAGIPGMGSISLERTRQLGGLNIPPAVPQ-ALPYSYAD 59

Anolis_sagrei_ordinatus_Pleu MKQWMLVSSLLGICSALPLFPQHAGIPGMGSISLERTRQLGGLNIPSAPPQ-ALPYSY-D 58

Ctenosaura_bakeri_Pleu MKQWMLVSSLLGICSALPILPQHAGIPGMGSISLERMRQLGGQNIPNVLSQYASPYSYAD 60

Iguana_delicatissima_Pleu MKQWMLISSLLGICSALPILPQHAGIPGMGSISLERMRQLGGQNIPNVLPQYASPYSYAD 60

Cyclura_pinguis_Pleu MKQWMLVSSLLGICSALPILPQHAGIPGMGSISLERMRQLGGQNIPNVLSQYASPYSYAD 60

Gambelia_wislizenii_Pleu MKQWMIVSSLLGICSALPILPQHIRIPGMGSISLERMRQLGGPDMPNALPQYAPPYSYAD 60

Laemanctus_serratus_Pleur MKQWILVSSLLGICSALPILPQHTGIPGMGSISLERMRQLGGPNMPNVLPQYAPPYGYAD 60

Phrynosoma_blainvillii_Pleu MKQWILVSSLLGICSALPILPQHVGIPGMGSISLEGMKQLAGANMPNVLPQYAPPYGYAN 60

Phrynosoma_platyrhinos_Pleu MKQWILVSSLLGICSALPILPQHVGIPGMGSISLEGMKQLAGANMPNVLPQYAPPYGYAN 60

Sceloporus_chrysostictus_Pleur MKQWILVSSLLGICSAVPILPQHAGIPGMGSISLEGMRQVAGANMPNVLPQYASPYGYAN 60

Sceloporus_occidentalis_Pleu MKQWILVSSLLGICSALPILPQHAGIPGMGSISLEGMRQMTGANMPNVLPQYASRYGFAN 60

Sceloporus_tristichus_Pleu MKQWILVSSLLGICSALPILPQHAGIPGMGSISLEGMRQMAGANMPNVLPQYASPYGFAN 60

Sceloporus_undulatus_Pleu MKQWILVSSLLGICSALPILPQHAGIPGMGSISLEGMRQMAGANMPNVLPQYASPYGFAN 60

Urosaurus_nigricaudus_Pleu MKQWILVSSLLGICSALPILPQHAGIPGMGSISLEGMRQVAGANVPNVLPQYASPYGYAN 60

****::.*.***:* *:*::*** :**:****:* :*: * : * * . * : .

Acanthocercus_cyanogaster_Acr LLFPPWIHNHLPPHSSFYWMRQ-RPQDHETQRYEYTMPVHPPPLPSQQTPTLLQRP---E 116

Acanthocercus_minutus_Acr LLFPPWIHNHLPPHSSFYWMRQ-RPQDHETQRYEYTMPVHPPPLPSQQTPTLLQRP---E 116

Xenagama_zonura_Acr LLFPPWIHNHLPPHSSFYWMRQ-RPQDHETQRYEYTMPVHPPPLPSQQTPTLLQRP---E 116

Agama_doriae_AMBN_Acr MLFPSWIHNHLPPHSSFYWMRQ-RPQDHETQRYEYTMPVHPPPLPSQQTPTLLQRP---E 116

Laudakia_wui_Acr FLFPPWMHNHLPPHSSFYWMRP-RPQDHETQRYEYTLPVHPPPLPSQQTPTLLQRP---E 116

Phrynocephalus_forsythii_Acr FLFPPWIHNHLPPHSSFYWTRQ-RPQDHETQRYEYTLPVHPPPLPSQQTPTLLQRP---E 116

Phrynocephalus_guinanensis_Acr FLFPPWIHNHLPPHSSFYWTRQ-RPQDHETQRYEYTLPVHPPPLPSQQTPTLLQRP---E 116

Phrynocephalus_vlangalii_Acr FLFPPWIHNHLPPHSSFYWTRQ-RPQDHETQRYEYTLPVHPPPLPSQQTPTLLQRP---E 116

Phrynocephalus_versicolor_Acr FLFPPWIHNHLPAHSSFYWMRQ-RPQDHETQRYEYTLPVHPPPLPSQQTPTLLQRP---E 116

Intellagama_lesueurii_Acr LFFPPWMHNNLPPHSSFYWLRQ-RPQDHETQRYEYTMPVHPPPLPSQQTPTLLQQPGIPE 119

Pogona_vitticeps_Acr LFFPPWMHNHLPPHSSFYWLRQ-RPQDHETQRYEYTMPVHPPPLPSQQTPTLLQRPGIPE 119

Bradypodion_pumilum_Acr LYFPPWMHNHLPSHPSFYWLRQ-RPQDHGTQQYEYTMPVHPPPLPSQQTPILLQRPGIQE 119

Bradypodion_ventrale_Acr LYFPPWMHNHLPSHPSFYWLRQ-RPQDHGTQQYEYTMPVHPPPLPSQQTPILLQRPGIQE 119

Chamaeleo_calyptratus_Acr LYFPPWMHNHLPSHPSFYWLRQ-RPQDHGTQRYEYTMPVHPPPLPSQQTPILLQRSGIQE 119

Chamaeleo_dilepis_Acr LYFPPWMHNHLPSHPSFYWLRQ-RPQDHGTQRYEYTMPVHPPPLPSQQTPILLQRPGVQE 119

Chamaeleo_gracilis_Acr LYFPPWMHNQLPSHPSFYWLRQ-RPQDHGTQRYEYTMPVHPPPLPSQQTPILLQRPGIQE 119

Chamaeleo_laevigatus_Acr LYFPPWMHNHLPSHPSFYWLRQ-RPQDHGTQRYEYTMPVHPPPLPSQQTPILLQRPGIQE 119

Furcifer_pardalis_Acr LYFPPWMHNHLPSHPSFYWLRQ-RPQDHGTQRYEYTMPVHPPPLPSQQTPILLQRPGIQE 119

Trioceros_affinis_AMBN_Acr LYFPPWMHNHLPSHPSFYWLRQ-RPQDHETQRYEYTMPVHPPPLPSQQTPILLQRPGIQE 119

Trioceros_balebicornutus_Acr LYFPPWMHNHLPSHPSFYWLRQ-KPQDHGTQRYEYTMPVHPPPLPSQQTPILLQQPGIQE 119

Trioceros_harennae_Acr LYFPPWMHNHLPSHPSFYWLRQ-KPQDHGTQRYEYTMPVHPPPLPSQQTPILLQRPGNQE 119

Anolis_apletophallus_Pleu PFHSMWGRSFLPSH-TFLWMTH-GPQRQETQQYEYAMPVHPPPLPSLQPSIQFSEPRLPE 117

Anolis_rodriguezii_Pleu PFHSMWGRSFLPSH-TFLWMTH-GPQRQETQQYEYAMPVHPPPLPSLQPSIQFAEPRLPE 117

Anolis_carolinensis_Pleu PFHSMWGRSFLPSH-TFLWMTQGGPQRQDTQQYEYAMPVHPPPLPSLQTPIQFAEPRLPE 118

Anolis_sagrei_ordinatus_Pleu PFHSMWGRSFLPSH-TFLWMTQ-GPQRQETQQYEYAMPVHPPPLPSLQPAIQFSESRLPE 116

Ctenosaura_bakeri_Pleu PFHYMWGHSLLPSH-AFYWMRQ-RSQPHETQQYEYSMPVHPPPLPSIQTPTQFQQPGIQE 118

Iguana_delicatissima_Pleu PFHHMWGHSLLPSH-AFYWMRQ-RSQPHETQQYEYSMPVHPPPLPSIQTPTQFQQPGIQE 118

Cyclura_pinguis_Pleu PFHYMWGHSLLPSH-AFYWMRQ-RSQPHETQQYEYSMPVHPPPLPSIQTPTQFQQPGIQE 118

Gambelia_wislizenii_Pleu PYYSMWGRSFLPSH-GFYWMRQ-RPQPHETQQYEYSMPVHPPPLPSLQTPTQFQQPGIQE 118

Laemanctus_serratus_Pleur PFHAMWGRSFLPSH-GFYWMRQ-RSQPHETQQYEYSMPVHPPPLPSLQTPTQFQQPGIQE 118

Phrynosoma_blainvillii_Pleu TLNSLWMHGFLPPH-TFYWRSQ-RAQPHETQQYEYSMPVHPPPLPSLQTPIQFQQPGIQE 118

Phrynosoma_platyrhinos_Pleu TLNSLWMHGFLPPH-TFYWRSQ-RAQPHETQQYEYSMPVHPPPLPSLQTPIQFQQPGIQE 118

Sceloporus_chrysostictus_Pleur GFHSMWTHGFLPSH-AFYWRRQ-RAQPHETQQYEYSMPVHPPPLPSLQTPIQFQQPGIQE 118

Sceloporus_occidentalis_Pleu TFPSMWTHGFLPSH-GFYWRRQ-RAQPHETQQYEYSMPVHPPPLPSLQTPIQFQQPGIQE 118

Sceloporus_tristichus_Pleu AFPSMWTHGFLPSH-GFYWRRQ-RAQPHETQQYEYSMPVHPPPLPSLQTPIQFQQPGIQE 118

Sceloporus_undulatus_Pleu TFPSMWTHGIFPSH-GFYWRRQ-RAQPHETQQYEYSMPVHPPPLPSLQTPIQFQQPGIQE 118

Urosaurus_nigricaudus_Pleu PFHSMWTHGFLPSH-TFYWMRQ-RAQPHETQQYEYSMPVHPPPLPSLQTPIQFQQPGIQE 118

* :. :*.* * * .* : **:***::********* *.. : .. *

Acanthocercus_cyanogaster_Acr QPHYQ-FTDLAPTMPVQLQQYEMQPPFQQK-LSPEGNQPIVPQKKQLPLDRL-TQQ---- 169

Acanthocercus_minutus_Acr QPHYQ-FTDLAPTMPVQLQQYEMQPPFQQK-LSPEGNQPIVPQKKRLPLDRL-TQQ---- 169

Xenagama_zonura_Acr QPHYQ-FTDLAPTMPVQLQQYEMQPPFQQK-LSPEGNQPIVPQKKRLPLDRL-TQQ---- 169

Agama_doriae_AMBN_Acr QPHYQ-FTDLAPTMPVQIQQYEMQPPFQQK-VLPEGNQPLVPQKKRLPLDRL-TQQ---- 169

Laudakia_wui_Acr QPHYQ-FTDLAPTMPVQLQQYELQPPFQQK-LLPEGNQPVVPQKKQPPLDRL-IQQX--- 170

Phrynocephalus_forsythii_Acr QPHYQ-FTDLAPTMPVQLQQYEMQPPFQQK-LLPEGNQPFVPQKKQLPMDRL-TQQ---- 169

Phrynocephalus_guinanensis_Acr QPHYQ-FTDLAPTMPVQLQQYEMQPPFQQK-LLPEGNQPFVPQKKQLPVDRL-TQQX--- 170

Phrynocephalus_vlangalii_Acr QPHYQ-FTDLAPTMPVQLQQYEMQPPFQQK-LLPEGNQPFVPQKKQLPVDRL-TQQ---- 169

Phrynocephalus_versicolor_Acr QPHYQ-FTDLTPTMPVQLQQYEMQPPFQQK-LLPEGNQPLVPQKKQPPVDRL-TQQ---- 169

Intellagama_lesueurii_Acr QPHYQ-FTDLTPTMQVQLQQYEMQPPFQQM-PLPEGNQPVVPQKKRPPLDRQ-TQQS--- 173

Pogona_vitticeps_Acr QPHYQ-FTDLTPTMQAQLQQYEMQPPFQQT-PLPEGNQPVVPQKKRPPLDRQ-TQQ---- 172

Bradypodion_pumilum_Acr QPHYQ-FIDLTPTK--QVQQYEMQPPFQQN-PFPEGNQPVVPQKQLLPSDRQ-TQQS--- 171

Bradypodion_ventrale_Acr QPHYQ-FIDLTPTK--QVQQYEMQPPFQQK-PFPEGNQPVVPQKQLLPSDRQ-TQQS--- 171

Chamaeleo_calyptratus_Acr QPHYQ-FIDLAPTK--QVQQYEMQPPFQQK-PLPEGNQPVVPQKQLLPSDRQ-TQQS--- 171

Chamaeleo_dilepis_Acr QPHYQ-FIDLAPTK--QVQQYEMQPPFQQK-PSPEGNQPLVPQKQLLPSDRQ-TQQS--- 171

Chamaeleo_gracilis_Acr QPHYQ-FIDLAPTK--QVQQYEMQPPFQQK-PSPEGNQPLVPQKQLLPSDRQ-TQQS--- 171

Chamaeleo_laevigatus_Acr QPHYQ-FIDLTPTK--QVQQYEMQPPFQQK-PSPEGNQPVVPQKQLLPSDRQ-TPQS--- 171

Furcifer_pardalis_Acr QPHYQ-FIDLTPTK--QVQQYEMRPPFQQN-PSPDGNQPVVPQKQLLSSDRQ-TQQS--- 171

Trioceros_affinis_AMBN_Acr QPHYQ-FIDLSPTK--QVQQYEMQPPFQQK-PSLEGSQPVVPQKQLLSLDRQ-TQQS--- 171

Trioceros_balebicornutus_Acr QPHYQ-FIDLTPTK--QVQQYEMQPPFQQK-PSPEGNQPVVPQKQLLSSDRQ-TQQS--- 171

Trioceros_harennae_Acr QPHYQ-FIDLTPTK--QVQQYEMQPPFQQK-PSPEGNQPVVPQKQLLSSDRQ-TQQS--- 171

Anolis_apletophallus_Pleu QPFFQ-IANLIPTRQVQIPHYNIRPRIEQTFPQPEAYPPVDPQQQPQPTERQ-TNQ---- 171

Anolis_rodriguezii_Pleu QPFFQ-IANLIPTRQVQIPHYNIQPRIEQTFPQPEAYPPVDPQQQPQPTERQ-TNQ---- 171

Anolis_carolinensis_Pleu QPFFQ-ITNLIPTRQVQIPRYAIQPSIQQTFQQVEAHPPVDPQQQPLPAERQ-THQ---- 172

Anolis_sagrei_ordinatus_Pleu QPFFQPFTNLFPARQLQIPQYNIQPPIQQTFPQLEAQPAVDPQQQPLPAERQ-THQ---- 171

Ctenosaura_bakeri_Pleu QPHYQ-VTNLIPTRQVQIQQYDVQPPIQQKFSPPEGNQPADPQKQPLQTERQ-TQQL--- 173

Iguana_delicatissima_Pleu QPHYQ-VTNLIPTRQVQIQQYDVQPQIQQKFPPPEGNQPADPQQQPLPTERQ-TQQL--- 173

Cyclura_pinguis_Pleu QPHYQ-VTNLIPTRQVQVQQYDVQPPIQQKFPPPEGNQPADPQQQPQPTERQ-TQQL--- 173

Gambelia_wislizenii_Pleu QPHYQ-VTNLIPTRQVQLQQYDVQPPIQQNFPPPEGNQPINPQQQALPAERQKSQQL--- 174

Laemanctus_serratus_Pleur QAHYQ-ITNQLPTRQVRLQLYDVQPPIQQKFPPLEGKQPVDPQQQPLPAERQ-TQQL--- 173

Phrynosoma_blainvillii_Pleu QPHYQ-VLNQIPTRQVWLQLYDGQPPIQQKFPPPEPNQPVDPQQQLLPGERQ-TQQL--- 173

Phrynosoma_platyrhinos_Pleu QPHYQ-VLNQIPTRQVWLQLYDGQPPIQQKFPPTEPNQPVDPQQQPLPGDKQ-TQQL--- 173

Sceloporus_chrysostictus_Pleur QPHYQ-VLNQIPTRQVWVHPYDVQPSIQQQFPPPEANQPVDPQQQTLTGERQ-TQQIYPG 176

Sceloporus_occidentalis_Pleu QPPYQ-VLNQIPTRQVWLHPYDVQPSIQQKFPPPEAKQPVDPQQQTLPGERQ-TQQL--- 173

Sceloporus_tristichus_Pleu QPPYQ-VLNQIPTRQVWLHPYDVQPSIQQKFPPPEANQPVDPQQQTLPGERQ-TQQL--- 173

Sceloporus_undulatus_Pleu QPPYQ-VLNQIPTRQVWLHPYDVQPSIQQKFPPPEANQPVDPQQQTLPGERQ-TQQL--- 173

Urosaurus_nigricaudus_Pleu QSHYQ-VLNQIPTRQVWLHPYDVQPSIQQKFPPPEANQPVDPQQQTLPGERQ-TQQL--- 173

*. :* . : *: : * :* ::* : . **:: :: *

Acanthocercus_cyanogaster_Acr ---------------IFPGLHGTQQNQA-QSPPQQS---VYPNLYYMPYVANQGAAPGRL 210

Acanthocercus_minutus_Acr ---------------IFPGLHGTQQDQA-QSPPQQS---VYPNLYYMPYVANQGAAPGRL 210

Xenagama_zonura_Acr ---------------IFPGLHGTQQDQA-QSPPQQS---VYPNLYYMPYVANQGAAPGRL 210

Agama_doriae_AMBN_Acr ---------------ILPGLHGTQQDQV-QSPQQQS---IYPNLYYMPYVANQGAAPGRL 210

Laudakia_wui_Acr --XXXXXXXXXXXXXIFPGLHGTQQDQV-QSSPQQS---MYPNLYYMPYVANQGAAPGRL 224

Phrynocephalus_forsythii_Acr ---------------IFPGLHGTQQDQS-QSPPQQS---IHPNLYYMPYIANQGAAPGRL 210

Phrynocephalus_guinanensis_Acr --XXXXXXXXXXXXXIFPGLHGTQQDQS-QSPQQQS---IHPNLYYMPYAANQGAAPGRL 224

Phrynocephalus_vlangalii_Acr ---------------IFPGLHGTQQDQS-QSPQQQS---IHPNLYYMPYVANQGAAPGRL 210

Phrynocephalus_versicolor_Acr ---------------IFPGLHGTQQGQA-QSPQQQS---IYPNLYYMPYMANQGAAPGRL 210

Intellagama_lesueurii_Acr --PTVQEYLGTLGQTIYPGLHGNQQDQA-QPPPQQS---IYPNLYYMPYVANQGAAPGRL 227

Pogona_vitticeps_Acr ---------------IYLGLHGNQQDQA-QPPPQQS---IYPNLYYMPYVANQGAAPGRL 213

Bradypodion_pumilum_Acr --PTIQEYSGALGQTIYPGFHGAQPDQV-QPPQQQS---IYPNLYYMPYGANQGAAPGRL 225

Bradypodion_ventrale_Acr --PTIQEYSGALGQTIYPGFHGAQPDQV-QPPQQQS---IYPNLYYMPYGANQGAAPGRL 225

Chamaeleo_calyptratus_Acr --PIIQEYSGALGQTIYPGLHGAQTDQV-QQPQQTI---IYPSLYYMPYGANQGAAPGRL 225

Chamaeleo_dilepis_Acr --PIIQEYSGALGQTIYPGLHGAQTDQV-QQPQXTI---IYPSLYYMPYRANQGAAPGRL 225

Chamaeleo_gracilis_Acr --PIIQEYSGALGQTIYPGLHGAQTDQV-QQPQQQP---IYPSLYYMPYGANQGAAPGRL 225

Chamaeleo_laevigatus_Acr --PIIQEYSGALGQTIYPGLHGAQTDQV-QQPQXTI---IYPSLYYMPYGANQGAAPGRL 225

Furcifer_pardalis_Acr --STIQEYSGALGQTIYPGLHGAQPDQV-QQPQQQS---IYPNLYYMPYGANQGAAPGRL 225

Trioceros_affinis_AMBN_Acr --PIIQEYSGALGQT------------------------IYPNLYYMPYGTNQGAVPGRL 205

Trioceros_balebicornutus_Acr --PIIQEYSGALGQTIYPGLNGAQPDQV-QQPQXTI---IYPNLYYMPYGANQRAASGRL 225

Trioceros_harennae_Acr --PIIQEYSGALGQTIYPGLNGAQPDQV-QQPQXTI---IYPNLYYMPYGANQGAAPGRL 225

Anolis_apletophallus_Pleu ---------------IYSVLHGTQQDPSQQQQQQQS---MYPSLYYLPYVANQGAAPARL 213

Anolis_rodriguezii_Pleu ---------------IYSVLHGTQQDPS-QQQQQQS---MYPSLYYLPYVANQGAAPARL 212

Anolis_carolinensis_Pleu ---------------IYPALHGTQQDPS-QQLQQQS---IYPSLYYMPYVANQGAAPARQ 213

Anolis_sagrei_ordinatus_Pleu ---------------IYPALHGTQQDPS-QQQQQQQ--PMYPSLYYLPYVANQGAAPARL 213

Ctenosaura_bakeri_Pleu --STVQERSGTFGQTIYPGLHGTQQDPS-QQLQQRS---MYPHLYYLPYVANQGGAPARL 227

Iguana_delicatissima_Pleu --STVQERSGTFGQTIYPGLHGTQQDPS-QQLQQRS---VYPHLYYLPYVANQGGAPARL 227

Cyclura_pinguis_Pleu --STVQERSGTFGQTIYPGLHGTQQDPS-QQLQQRS---MYPHLYYLPYVANQGGAPARL 227

Gambelia_wislizenii_Pleu --STVQEHSGTFGQTIFPGLHGTQQDPS-QQLQQQS---MYPNLYYLPYVANQGVAPGRL 228

Laemanctus_serratus_Pleur --STVQERSGTFGQIIYPGLHGTQQDPS-QQPQQRP--VMYPSLYYLPYVANQGGAPARM 228

Phrynosoma_blainvillii_Pleu --STVQEHKGTFGQTIYPGLHGTQQDPS-----QRS---MLPNLYYLPYVANQGGTPARI 223

Phrynosoma_platyrhinos_Pleu --STVQEHTRAFGQTIYPGLHGTQQDPS-----QRS---MLLNLYYLPYVANQGGTPARM 223

Sceloporus_chrysostictus_Pleur LHGTQQDPS---QQSIYPGLHGTQQDPS-----QQS---MLPSLYYLPYVANQGGAPARM 225

Sceloporus_occidentalis_Pleu --STVQEHTGTFGQTFYPGLHGTQQDPS-----QRS---MLPNLYYLPYVANQGGAPARM 223

Sceloporus_tristichus_Pleu --STVQEHTGTFGQTFYPGLHGTQQDPS-----QRSVSLMLPNLYYLPYVANQGGAPARM 226

Sceloporus_undulatus_Pleu --STVQEHTGTFGQTFYPGLHGTQQDPS-----QRSVSLMLPNLYYLPYVANQGGAPARM 226

Urosaurus_nigricaudus_Pleu --STVQEHTGTFGQTFYPGLHGTQQDPS-----QRSVSLMLPSLYYLPYIANQGGAPARM 226

: ***:** :** ...*

Acanthocercus_cyanogaster_Acr GIVSSEEMQGGGYGAPAYQAPGPDLFGTDARLRSMALNQPGDYTVEDDQLGITEQPNVKG 270

Acanthocercus_minutus_Acr GIVSSEEMQGGGYGAPAYQAPGPDLFGTDARLRSMALNQPGDYTVEDDQLGITEQPNVKG 270

Xenagama_zonura_Acr GIVSSEEMQGGGYGAPAYQAPGPDVFGTDARLRSMALNQPGDYTVEDDQLGITEQPNVKG 270

Agama_doriae_AMBN_Acr GIVSSEEMQGGGFGPPAYQAPGPDLFGTDARLRSMALNQPGDYTVEDDQLGITEQPNVKG 270

Laudakia_wui_Acr GIVSSEEMQGGGFGAPAYQAPGPDLFGTDARLRSMALNQPGDYTVEDDQLGITEQPNVKG 284

Phrynocephalus_forsythii_Acr GIVSSEEMQGGGFGAPAYQAPGPDLFGTDARLRSMVLNQPGDYTVEDDQLGITEQPNVKG 270

Phrynocephalus_guinanensis_Acr GIVSSEEMQGGGFGAPAYQAPGPDLFGTDARLRSMALNQPGDYTVEDDQLGITEQPNVKG 284

Phrynocephalus_vlangalii_Acr GIVSSEEMQGGGFGAPAYQAPGPDLFGTDARLRSMALNQPGDYTVEDDQLGITEQPNVKG 270

Phrynocephalus_versicolor_Acr GIVSSEEMQGGGFGAPAYQAPGPDLFGTDARLRSMALNQPGDYTVEDDQLGITEQPNVKA 270

Intellagama_lesueurii_Acr GIVSSEEMQGGGFGPPAYQAPGPDLFATDARLRHMALNQPGDYTVEDDQLGITEQPDVKG 287

Pogona_vitticeps_Acr GIVSSEEMQGGGFGPPAYQAPGPDLFATDARLRHMALNQPGDYTVEDDQLGITEQPDVKG 273

Bradypodion_pumilum_Acr GIVSSEEMQGGGFGPPAYQAPGPDLFATGARLRDMALNQPGDYTVEDDQLGITEQPDVKG 285

Bradypodion_ventrale_Acr GIVSSEEMQGGGFGPPAYQAPGPDLFATGARLRDMALNQPGDYTVEDDQLGITEQPDVKG 285

Chamaeleo_calyptratus_Acr GIVSSEEMQGGGFGPPAYQAPGPDLFATGARLRDMALNQPGDYTVEDDQLGITEQPDVKG 285

Chamaeleo_dilepis_Acr GIVSSEEMQGGGFGPPAYQAPGPDLFATGARLRDMALNQPGDYTVEDDQLGITEQPDVKA 285

Chamaeleo_gracilis_Acr GIVSSEEMQGGGFGPPAYQAPGPDLFATAAKLRDMALNQPGDYTVEDDQLGITEQPDVKG 285

Chamaeleo_laevigatus_Acr GIVSSEEMQGGGFGPPAYQVPGPDLYATGARLRDMALNQPGDYTVEDDQLGITEQPDVKG 285

Furcifer_pardalis_Acr GIVSSEEMPGGGFGPPAYQAPGPDLFATGARLRDMALNQPGDYTVEDDQLGITEQPDVKG 285

Trioceros_affinis_AMBN_Acr GIVSSEEMQGGGFGPPAYQAPGPDLFATGARLRDMALNQPGDYTVEDDQLGITEQPDVKG 265

Trioceros_balebicornutus_Acr GIVSSEEMQGGGFGPPAYQAPGPDLFATGARLRDMALNQPGDYTVEDDQLGITEQPDVKG 285

Trioceros_harennae_Acr GIVSSEEMQGGGFGPPAYQALGPDLFATGARLRDMALNQPGDYTVEDDQLGITEQPDVKG 285

Anolis_apletophallus_Pleu GIVSSEEMQGGGFGAPAYRAAGPDLFPMDTRFGNSPLNQPGDYTVEDDQLGITEQPDVKG 273

Anolis_rodriguezii_Pleu GIVSSEEMQGGGFGAPAYRAAGPDLFPMDTRFGNSPLNQPGDYTVEDDQLGITEQPDVKG 272

Anolis_carolinensis_Pleu GIVSSEEMQGGGFGAPAYRAPGPDLFAMDTRFGNSPLNQPGDYTVEDDTLGITEQPDVKG 273

Anolis_sagrei_ordinatus_Pleu GIVSSEEMQGGGYGAPAYRAAGPDLFPMDTRFGNSPLNQPGDYTVEDDQLGITEQPDVKG 273

Ctenosaura_bakeri_Pleu GIVSSEEMQGGGFGAPAYQAAGTDPFAMNARFGNSPLNQPGDYTVEDDQLGITEQPDVPG 287

Iguana_delicatissima_Pleu GIVSSEEMQGGGFGAPAYQAAGTDPFAMNARFENSPLNQPGDYTVEDDQLGITEQPDMPG 287

Cyclura_pinguis_Pleu GIVSSEEMQGGGFGAPAYQAAGTDPFAMNARFGNSPLNQPGDYTVEDDQLGITEQPDVRG 287

Gambelia_wislizenii_Pleu GIVSSEEMQGGGFGAPAYRAAGTDLFAMDARFGNSPLNQPGDYTVEDDQLGITEQPDVQG 288

Laemanctus_serratus_Pleur GIVSSEEMQGGGFGAPAYRAAGPDFYPMDTRFGNSPLNQPGDYTVEDDQLGITEQPDVQG 288

Phrynosoma_blainvillii_Pleu GIVSSEEMQGGGFGAPAYRAAGTDLFAMNARFGNSPLNQPGDYTVEDDQLGITEQPDVKG 283

Phrynosoma_platyrhinos_Pleu GIVSSEEMQGGGFGAPAYRAAGTDLFAMDARFGNSPLNQPGDYTVEDDQLGITEQPDVKG 283

Sceloporus_chrysostictus_Pleur GIVSSEEMQGGGFGAPAYRAAGTDLFAMDARFGNSPLNQPGDYTVEDDQLGITEQPDVKG 285

Sceloporus_occidentalis_Pleu GIVSSEEMQGGGFGAPAYRAAGTDLFAMDARFGNSPLNQPGDYTVEDDQLGITEQPDVKG 283

Sceloporus_tristichus_Pleu GIVSSEEMQGGGFGAPAYRAAGTDLFAMDARFGNSPLNQPGDYTVEDDQLGITEQPDVKG 286

Sceloporus_undulatus_Pleu GIVSSEEMQGGGFGAPAYRAAGTDLFAMDARFGNSPLNQPGDYTVEDDQLGITEQPDVKG 286

Urosaurus_nigricaudus_Pleu GIVSSEEMQGGGFGAPAYRAAGTDLFAMDARFGNSPLNQPGDYTVEDDQLGITEQPDVKG 286

******** ***:*.***:. *.* : ::: ************ *******:: .

Acanthocercus_cyanogaster_Acr EPNVGANPPGRGNPIHPLEGSPVIGSLPNANGYLLNPAGQSKGPLGDPQATVGSLAPEGL 330

Acanthocercus_minutus_Acr EPNVGANPSGRGNPIHPLEGSPVIGSLPNANGYLLNPAGQSKGPLGDPQATVGSLAPEGL 330

Xenagama_zonura_Acr EPNVGANPSGRGNPIHPLEGSPVIGSLPNANGYLLNPAGQSKGPLGDPQATVGSLAPEGL 330

Agama_doriae_AMBN_Acr EPNVGANPAGRGNPIHPLEGSPVVGASPNANGYLLNPAGQSKGPLGDPQATVGSLAPEGL 330

Laudakia_wui_Acr EPNVGANPTGRGNPIHPLEGNPVIGASPNANGYLLNPAGQSKGPLGDPQATVGSLAPEGL 344

Phrynocephalus_forsythii_Acr EPNVGANPTGRGNPIHPLEGNPVIGASPNADGYLLNPAGQSKGPLGDPQATVSSLAPEGL 330

Phrynocephalus_guinanensis_Acr EPNVGANPTGRGNPIHPLDGNPVIGASPNADGYLLNPAGQSKGPLGDPQATVSSLAPEGL 344

Phrynocephalus_vlangalii_Acr EPNVGANPTGRGNPIHPLEGNPVIGASPNADGYLLNPAGQSKGPLGDPQATVSSLAPEGL 330

Phrynocephalus_versicolor_Acr EPNVGANPTGRGNPIHPLEGNPVIGASPNADGYLLNPAGQSKGPLGDPQVTVSSLAPEGL 330

Intellagama_lesueurii_Acr GANVGANPAGKGNPINPLEGNPAIGASPNANSYLLHPAGQSKGPLGEPQATGGPLAPEGL 347

Pogona_vitticeps_Acr GANVGANPAGKGNPISPLEGNPAIGASPNANSYLLHPAGQSKGPLGEPQATGGPLAPEGL 333

Bradypodion_pumilum_Acr GANAGANPSGQGNPIIPIEGNPAVGASPNVNHYLLNPAGQSKGPVGAPYGTAGPLAPEGL 345

Bradypodion_ventrale_Acr GANAGANPSGQGNPIIPVEGNPAVGASPNVNHYLLNPAGQSKGPVGAPYGTAGPLAPEGL 345

Chamaeleo_calyptratus_Acr GANAGANPSGQGNPIIPIEGNPAVGTSANVNHYLLNPAGQSKGPVVAPYGTPGPLAPEGL 345

Chamaeleo_dilepis_Acr GANAGANPSGQGNPIIPVEGNPAVGASANVNHYLLNPAGQSKGPVGAPYGTAGPLAPEGL 345

Chamaeleo_gracilis_Acr GANAGANPSGQGNPIIPVEGNPAVGASANVNHYLLNPAGQSKGPVGAPYGTAGPLAPEGL 345

Chamaeleo_laevigatus_Acr GANAGANPSGQGNPVIPAEGNPAVGGSANVNHYLLNPAGQSKGPVGAPYGTAGPLAPEGL 345

Furcifer_pardalis_Acr GANAGANPSGQGNPIIPIEGNPAVGASANVNHYLLNPAGQSKGPGGAPHGTAGPLAPEGL 345

Trioceros_affinis_AMBN_Acr GANAGANPSGQGNPIIPVEGNPAVGASANVNHYLLNPAGQSKGPVGAPYGTAGPLAPSGL 325

Trioceros_balebicornutus_Acr GANAGANPSGQGNPIIPVEGNPAVGASANVNHYLLNPAGQSKGPVGAPYGTAGPLAAAGL 345

Trioceros_harennae_Acr GANAGANPSGQGNPIIPVEGNPAVGASANVNHYLLNPAGQSKGPAGAPYGTAGPLAAAGL 345

Anolis_apletophallus_Pleu GANAGAGPSGKGNNAVLLDGTQG-GSLPNANSHLFNPAGQSQGLSGLAQATVSPHATQG- 331

Anolis_rodriguezii_Pleu GANAGAGPSGKGNNAVLLDGTQG-GALPNANSHLFNPAGQSQGLSGLAHATVSPHATQG- 330

Anolis_carolinensis_Pleu GANAGANPSGKGNNAILLDGTQG-GALPNANSHLFSPAGQSQGLSGLAQATAAPHATQG- 331

Anolis_sagrei_ordinatus_Pleu GANAGANPSGKGNNAVLLDGTQG-GALPNPNSHLFNPAGQSQGLSGLAQATMAPHATQG- 331

Ctenosaura_bakeri_Pleu GAKAGTNPAARGNNAIPVDG----AALPNANSHLLSPAGQSQGLSGVAQATAVPLAPQGA 343

Iguana_delicatissima_Pleu GAKAGTNPAARGNSAIPVDG----AALPNANSHLLSPAGQSQGLSGVAQATTVPLAPQGA 343

Cyclura_pinguis_Pleu GAKAGTNPAARGNNAIPVDG----AALPNANSHLLGPAGQSQ---GVTQATAVPLAPQGG 340

Gambelia_wislizenii_Pleu GANAGTNPSGKGNNAILANGNQD-GALPNANSHLLNPAGQSQGLSGVARDTAVPLATQGT 347

Laemanctus_serratus_Pleur GANAGTNPSGKGTNAILVDGNQ--GALPNANSHLFSPAGQSQGLSGVAQATAAPLATQGP 346

Phrynosoma_blainvillii_Pleu GANAGTNPAGQGNNVIPIDGSQS-GTLPNANSHLLSPAGQSQGLSGIAQATEAPLPTQGL 342

Phrynosoma_platyrhinos_Pleu GANAGTNPAGQGNNVIPIDGSQG-GALPNAHSHLLSPAGQSQGLSGVAQATAAPLPTQGL 342

Sceloporus_chrysostictus_Pleur GANAGTNPSGQGNNGIPIDGSQG-GALPNANSHLLSPAGQSQGLSGVAQATGVPLPTQGL 344

Sceloporus_occidentalis_Pleu GANAGTNPSGQGNNAIPVDGSQG-GALLNANSHLLSPAGQSQGLSGVAQATGIPLPTQGL 342

Sceloporus_tristichus_Pleu GANAGTNPSGQGNNAIPVDGSQG-GALLNANSHLLSPAGQSQGLSGVAQATGIPLPTQGL 345

Sceloporus_undulatus_Pleu GANAGTNPSGQGNNAIPVDGSQG-GALLNANSHLLSPAGQSQGLSGVAQATGIPLPTQGL 345

Urosaurus_nigricaudus_Pleu GANAGTNPSGQGNNAIPVDGSQG-GALPNANSHLLSPAGQSQGLSGVIQATGVPLPTQGL 345

.:.*:.*..:*. :* . * . :*: *****: * . .. *

Acanthocercus_cyanogaster_Acr P----VLDAAGTAMPLDPTMFPDTMFPGNAGNEAGLSPIGQDMWHFQEP 375

Acanthocercus_minutus_Acr P----VLDAAGTAMPLDPTMFPDTMFPGNAGNEAGLSPIGQDMWHFQEP 375

Xenagama_zonura_Acr P----VLDAAGTAMPLDPTMFPDTMFPGNAGNEAGLSPIGQDMWHFQEP 375

Agama_doriae_AMBN_Acr P----VLDAAGTAMPLDPTPFPDTMFPANAGNEAGLSPIGQDMWHFQEP 375

Laudakia_wui_Acr P----VMDAAGTAMPLDPTTFPDTMFPASAGNEAGLSPIGHDMWHFQEP 389

Phrynocephalus_forsythii_Acr P----VWDAAGTAMPLDPTTFPDTMFPASSGNEAGLSPIGQDMWHFQEP 375

Phrynocephalus_guinanensis_Acr P----VWDAAGTAMPLDPTTFPDTMFPASSGNEAGLSPIGQDMWHFQEP 389

Phrynocephalus_vlangalii_Acr P----VWDAAGTAMPLDPTTFPDTMFPASSGNEAGLSPIGQDMWHFQEP 375

Phrynocephalus_versicolor_Acr P----VLDAAGTAMPLDPTTFPDTMFPASSGNEAGLSPIGQDMWHFQEP 375

Intellagama_lesueurii_Acr P----VLDAAGTTMPLDPTMFPDTMFPTDVGNGAGLSPIGQDMWNFQEP 392

Pogona_vitticeps_Acr P----VVDAAGTAMPLDPTTFPDTMFPTDVGNGAGLSPIGQDMWHFQEP 378

Bradypodion_pumilum_Acr P----VLEVAGTTIPLDPTTFPDTTFPTNVRNEAGLSQTGQDLWYFQAP 390

Bradypodion_ventrale_Acr P----VLEVAGTTIPLDPTTFPDTTFPTNVGNEAGLSQTGQDLWYFQAP 390

Chamaeleo_calyptratus_Acr P----VLEVAGTTIPLDPTMFPDTTFPTDVGNEAGLSQTGQDSWYFQAP 390

Chamaeleo_dilepis_Acr P----VLEVAGTTIPLEPTMFPDTTFPTDVGNEPGLSQTGQDLWYFQAP 390

Chamaeleo_gracilis_Acr P----VLEAAGTTIPLEPTMFPDTTFPTDVGNEAGLSQTGQDLWYFQAP 390

Chamaeleo_laevigatus_Acr P----VLEVAGTTIPLDPTMFPDTTFPTDVGNEAGLSQTGQDLWYFQAP 390

Furcifer_pardalis_Acr P----VLEVAGTTIPLDPTMFPDTTFPTDVGNEPGLSQTGQDLWYFQAP 390

Trioceros_affinis_AMBN_Acr P----VLEVAGTAIPLDPTMFPDTTFPNDVGNEAGLSQTGQDLWYFQAP 370

Trioceros_balebicornutus_Acr P----VLEVAGTAIPLDPTIFPDTTFPNDVGNEAGLSQTGQDLWYFQAP 390

Trioceros_harennae_Acr P----VLEVAGTAIPLDPTIFPDTTFPNDVGNEAGLSQTGQDLWYFQAP 390

Anolis_apletophallus_Pleu --PFLPLDAADPSMPLDSTMFPD-LYTTNVGNEAGLAPVGQDAWHFQEP 377

Anolis_rodriguezii_Pleu --PFLPLDAADPSMPLDSTMFPD-LYTTNVGNEAGLAHVGQDAWHFQEP 376

Anolis_carolinensis_Pleu --PFLPLDAADPSMPLDAPMFPD-LYTSNVGNEAGLAQVGQDAWHFQEP 377

Anolis_sagrei_ordinatus_Pleu --PFLSLDAADPSMPLDSTMFPD-LYTTNVGNEPGLAHVGQDAWHFQEP 377

Ctenosaura_bakeri_Pleu PDSFLPLDAADPTGPLDPTMFPD-VYTTNAGNEAGMARVGQDAWHFQEP 391

Iguana_delicatissima_Pleu PDSFLPLDAADPTGPLNPTMFPD-VYTTNAGNEAGMARVGQDTWRFQEP 391

Cyclura_pinguis_Pleu PGSFLPLDATDPTGPLDPTMFPD--YTTNAGNEAGMAHVGQDAWHFQEP 387

Gambelia_wislizenii_Pleu PGSFLPLDAVDPTMPLDPTMLPD-IYTTNVGNEAGLAHVGQDVWHFQEP 395

Laemanctus_serratus_Pleur PGNFLPLDAADPTMPFDPTAFPD-LYTTNAGNEAGLAHVGQDVWHFQEP 394

Phrynosoma_blainvillii_Pleu PGSFLPLDSADPTMPLDPTIFPD-IYTTNAGNEAGLAQVGQDVWHFQEP 390

Phrynosoma_platyrhinos_Pleu PGGFLPLDSADPTIPLDPTTFPD-IYTTNSGNEAGLAQVGQDVWHFQEP 390

Sceloporus_chrysostictus_Pleur PGSFLPLDSADPTIPLDPTMFPD-IYTANAGNEAGLAQVGQDAWHFQEP 392

Sceloporus_occidentalis_Pleu PGSFLPLDSADPTMPLDPTMFPD-TYTANAGNEAGMAQVGQDVWHFQEP 390

Sceloporus_tristichus_Pleu PGSFLPLDSADPTMSLDPTMFPD-TYTANAGNEAGMAQVGQDVWHFQEP 393

Sceloporus_undulatus_Pleu PGSFLPLDSADPTMSLDPTMFPD-TYTANAGNEAGMAQVGQDVWHFQEP 393

Urosaurus_nigricaudus_Pleu PGSFLPLDSADPTMPLDPTMFPD-IYTANAGNEAGMAQVGQDAWHFQEP 393

: ...: .::.. :** :. . * .*:: *:* * ** *

**AMEL-AA-MAFFT-Iguania+Mammal – DIVERGE + PCOC ≥ 0.9**

CLUSTAL W (1.8) multiple sequence alignment (ALTER 1.3.3)

Acanthocercus_cyanogaster_AMEL_Acr MEGWTLIMCLLCTTLAVPLPQ---HPGFINFSYE-----------------VMTPLKWYQ 40

Acanthocercus_minutus_AMLE_Acr MEGWTLIMCLLCTTLAVPLPQ---HPGFINFSYE-----------------VMTPLKWYQ 40

Xenagama_zonura_AMLE_Acr MEGWTLIMCLLCTTLAVPLPQ---HPGFINFSYE-----------------VMTPLKWYQ 40

Laudakia_wui_Acr MEGWTLIMCLLCTTLAVPLPQ---HPGFINFSYE-----------------VMTPLKWYQ 40

Agama_doriae_AMLE_Acr MEGWTLIMCLLGTTLAVPLPQ---HPGFINFSYE-----------------VMTPLKWYQ 40

Phrynocephalus_forsythii_Acr MEGWILIMCVLSTTLAVPLPQ---HPGFINFSYE-----------------VMTPLKWYQ 40

Phrynocephalus_guinanensis_Acr MEGWILIMCVLSTTLAVPLPQ---HPGFINFSYE-----------------VMTPLKWYQ 40

Phrynocephalus_putjatai_Acr MEGWILIMCVLSTTLAVPLPQ---HPGFINFSYE-----------------VMTPLKWYQ 40

Phrynocephalus_vlangalii_Acr MEGWILIMCVLSTTLAVPLPQ---HPGFINFSYE-----------------VMTPLKWYQ 40

Phrynocephalus_versicolor_Acr MEGWTLIMCLLSTTLAVPLPQ---HPGFINFSYE-----------------VMTPLKWYQ 40

Intellagama_lesueurii_Acr MEGWTLIMSLLCTTLAIPLPQ---HPGFINFSYE-----------------VMTPLKWYQ 40

Pogona_vitticeps_Acr MEGWTLIMSLLCTTLAIPLPQ---HPGFINFSYE-----------------VMTPLKWYQ 40

Bradypodion_pumilum_Acr MECLVLIMSLLCTTFAIPLPQ---HPGFINFSYE-----------------VMTPLKWYQ 40

Bradypodion_ventrale_Acr MECLVLIMSLLCTTFAIPLPQ---HPGFINFSYE-----------------VMTPLKWYQ 40

Chamaeleo_calyptratus_AMEL_Acr MECLVLIMSLLCTTFAIPLPQ---HPGFINFSYE-----------------VMTPLKWYQ 40

Chamaeleo_laevigatus_AMEL_Acr MECLVLIMSLLCTTFAIPLPQ---HPGFINFSYE-----------------VMTPLKWYQ 40

Chamaeleo_gracilis_AMEL_Acr MECLVLIMSLLCTTFAIPLPQ---HPGFINFSYE-----------------VMTPLKWYQ 40

Chamaeleo_dilepis_AMEL_Acr MECLVLIMSLLCTTFAIPLPQ---HPGFINFSYE-----------------VMTPLKWYQ 40

Trioceros_affinis_AMEL_Acr MECLVLIMSLLCTTFAIPLPQ---HPGFINFSYE-----------------VMTPLKWYQ 40

Trioceros_balebicornutus_AMEL_Acr MECLVLIMSLLCTTFAIPLPQ---HPGFINFSYE-----------------VMTPLKWYQ 40

Trioceros_harennae_AMEL_Acr MECLVLIMSLLCTTFAIPLPQ---HPGFINFSYE-----------------VMTPLKWYQ 40

Furcifer_pardalis_Acr MECLVLIMSLLCTTFAIPLPQ---HPGFINFSYE-----------------VMTPLKWYQ 40

Anolis_apletophallus_Pleu MEGWTLIMCLLSTTFAMPLPQ---HPGYINFSYE-----------------VMTPLKWYQ 40

Anolis_rodriguezii_AMEL_Pleur MEGWTLVMCLLSTTFAMPLPQ---HPGYVNFSYE-----------------VMTPLKWYQ 40

Anolis_tropidonotus_AMEL_Pleur MEGWTLVMCLLSTTLAIPLPQ---HPGYVNFSYE-----------------VMTPLKWYQ 40

Anolis_carolinensis_Pleu MEGWTLVMCLLSTTFAIPLPQ---HPGYINFSYE-----------------VMTPLKWYQ 40

Anolis_sagrei_ordinatus_Pleu MEGWTLIVCLLSTTLAMPLPQ---HPGYVNFSYE-----------------VMTPLKWYQ 40

Phrynosoma_blainvillii_Pleu MEGWTLVMCLLSTTFAIPLPQ---HPGFINFSYE-----------------VMTPLKWYQ 40

Sceloporus_chrysostictus_AMEL_Pleur MEGWTLVMCLLSTTFAIPLPQ---HPGFINFSYE-----------------VMTPLKWYQ 40

Urosaurus_nigricaudus_Pleu MEGWTLVMCLLSTTFAIPLPQ---HPGFINFSYE-----------------VMTPLKWYQ 40

Sceloporus_occidentalis_Pleu MEGWTLVMCLLSTTFAIPLPQ---HPGFINFSYE-----------------VMTPLKWYQ 40

Sceloporus_tristichus_Pleu MEGWTLVMCLLSTTFAIPLPQ---HPGFINFSYE-----------------VMTPLKWYQ 40

Sceloporus_undulatus_Pleu MEGWTLVMCLLSTTFAIPLPQ---HPGFINFSYE-----------------VMTPLKWYQ 40

Gambelia_wislizenii_Pleu MEGWTLVMCLLSTSFAIPLPQ---HPGFINFSYE-----------------VMTPLKWYQ 40

Laemanctus_serratus_AMEL_Pleur MEIWTLVMCLLSTTFAIPLPQ---HPGFINFSYE-----------------VMTPLKWYQ 40

Iguana_delicatissima_Pleu MGGWTLVMCLLCTTLAIPLPQ---HPGFINFSYE-----------------VMTPLKWYQ 40

Cyclura_pinguis_Pleu MGGWTLVMCLLCTTLAIPLPQ---HPGFINFSYE-----------------VMTPLKWYQ 40

Canis_lupus_dingo_AMEL MGTWILFACLLGAAFAMPLPPHPGHPGYINFSYE-----------------VLTPLKWYQ 43

Vulpes_vulpes_AMEL MGTWILFACLLGAAFAMPLPPHPGHPGYINFSYE-----------------VLTPLKWYQ 43

Meles_meles_AMEL MGTWILFACLLGAAFAMPLPPHPGHPGYINFSYE-----------------VLTPLKWYQ 43

Mustela_putorius_AMEL MGTWILFACLLGAAFAMPLPPHPGHPGYINFSYE-----------------VLTPLKWYQ 43

Echinops_telfairi_AMEL MGTWILFACLLGAAFAMPLPPHPGHPGYINFSYE-----------------VLTPLKWYQ 43

Phyllostomus_hastatus_AMEL MGTWILFACLLGAAFAMPLPPHPGHPGYINFSYEWNPYFQMININRTALLSVLTPLKWYQ 60

Homo_sapiens_AMEL MGTWILFACLLGAAFAMPLPPHPGHPGYINFSYE-----------------VLTPLKWYQ 43

Saimiri_boliviensis_AMEL MGTWILFACLLGAAFAMPLPPHPGHPGYINFSYE-NSHSQAINIDRTGL--VLTPLKWYQ 57

Erinaceus_europaeus_AMEL MGTWILFACLLGAAFAMPLPPHPGHPGYINFSYE-----------------VLTPLKWYQ 43

Ursus_arctos_AMEL MGTWILFACLLGAAFTMPLPPHPGHPGYINFSYE-NSRFQATNFDRTAL--VLTPLKWYQ 57

Suncus_etruscus_AMEL MGTWILFACILGVAFSMPLPSHPGHPGYINFSYE-----------------VLTPLKWYQ 43

Phacochoerus_africanus_AMEL MGTWILFACLLGAAFSMPLPPHPGHPGYINFSYE-DSHVEVIRIDRIAF--VLTPLKWYQ 57

* *. .:* .::::*** ***::***** *:*******

Acanthocercus_cyanogaster_AMEL_Acr GVM-GHQYPRYGYEPMGGWMHHSPGSMMHQSPFQT--LHPMPPPLHQVQ-QQPPLNPHM- 95

Acanthocercus_minutus_AMLE_Acr GVM-GHQYPRYGYEPMGGWMHHSPGSMMHQSPFQT--LHPMPPPLHQVQ-QQPPLNPHM- 95

Xenagama_zonura_AMLE_Acr GVM-GHQYPRYGYEPMGGWMHHSPGSMMHQSPFQT--LHPMPPPLHQVQ-QQPPLNPHM- 95

Laudakia_wui_Acr GVM-GHQYPRYGYEPMGGWMHHSPGSMMHQSPFQT--LHPMPPPLHQVQ-QQPPLNPHM- 95

Agama_doriae_AMLE_Acr GVM-GHQYPRYGYEPMGGWMHHSPGSMMHQSPFQT--LHPMPPPLHQVQ-QQPPLNPHM- 95

Phrynocephalus_forsythii_Acr GVM-GHQYPRYGYEPMGGWMHHSPGSMMHQSPFQA--LHPMPPPLHQVQ-QQPPLNPHM- 95

Phrynocephalus_guinanensis_Acr GVM-GHQYPRYGYEPMGGWMHHSPGSMMHQSPFQA--LHPMPPPLHQVQ-QQPPLNPHM- 95

Phrynocephalus_putjatai_Acr GVM-GHQYPRYGYEPMGGWMHHSPGSMMHQSPFQA--LHPMPPPLHQVQ-QQPPLNPHM- 95

Phrynocephalus_vlangalii_Acr GVM-GHQYPRYGYEPMGGWMHHSPGSMMHQSPFQA--LHPMPPPLHQVQ-QQPPLNPHM- 95

Phrynocephalus_versicolor_Acr GVM-GHQYPRYGYEPMGGWMHHSPGSMMHQSPFQT--LHPMPPPLHQVQ-QQPPLNPHM- 95

Intellagama_lesueurii_Acr GVM-GHQYPRYGYEPMGGWMHHSPGSMMHQTPFQT--LHPMPPPLHQVQ-QQPPLNPHM- 95

Pogona_vitticeps_Acr GVM-GHQYPRYGYEPMGGWMHHSPGSMMHQAPFQT--LHPMPPPLHQVQ-QQPPLNPHM- 95

Bradypodion_pumilum_Acr GLM-GQQYPRYGYEPMGGWMHHSPGPMMHQTPFQS--LHPMSPPLHQMQ-QLPPLNPHM- 95

Bradypodion_ventrale_Acr GLM-GQQYPRYGYEPMGGWMHHSPGPMMHQTPFQS--LHPMSPPLHQMQ-QLPPLNPHM- 95

Chamaeleo_calyptratus_AMEL_Acr GLM-GQQYPRYGYEPMGGWMHHSPGPMMHQTPFQS--LHPMSPPLHQMQ-QLPPLNPHL- 95

Chamaeleo_laevigatus_AMEL_Acr GLM-GQQYPRYGYEPMGGWMHHSPGPMMHQTPFQS--LHPMSPPLHQMQ-QLPPLNPHL- 95

Chamaeleo_gracilis_AMEL_Acr GLM-GQQYPRYGYEPMGGWMHHSPGPMMHQTPFQS--LHPMSPPLHQMQ-QLPPLNPHL- 95

Chamaeleo_dilepis_AMEL_Acr GLM-GQQYPRYGYEPMGGWMHHSPGPMMHQTPFQS--LHPMSPPLHQMQ-QLPPLNPHV- 95

Trioceros_affinis_AMEL_Acr GLM-GQQYPRYGYEPMGGWMHHSPGPMMHQTPFQS--LHPMSPPLHQIQ-QLSPLNPHM- 95

Trioceros_balebicornutus_AMEL_Acr GLM-GQQYPRYGYEPMGGWMHHSPGPMMHQTPFQS--LHPMSPPLHQIQ-QLPPLNPHM- 95

Trioceros_harennae_AMEL_Acr GLM-GQQYPRYGYEPMGGWMHHSPGPMMHQTPFQS--LHPMSPPLHQIQ-QLPPLNPHM- 95

Furcifer_pardalis_Acr GLM-GQQYPRYGYEPMGGWMHHSPGPMMHQTPFQS--LHPMSPPLHQIQ-QLPPLNPHMQ 96

Anolis_apletophallus_Pleu SLV-GHQYPRYGFEPMGGWMHHASGPTMPQTTFQS--HPSMHSTFHQMQPQHPALNPHM- 96

Anolis_rodriguezii_AMEL_Pleur SLI-GHQYPRYGFEPMGGWMHHASGPTMPQTPFQS--HPSMHSTFHQMQPQHPGLNPHM- 96

Anolis_tropidonotus_AMEL_Pleur SLI-GHQYPRYGFEPMGGWMHHASGPTMPQTTFQN--HPSMHSTFHQMQPQHPALNPHM- 96

Anolis_carolinensis_Pleu SLI-GHQYPRYGFEPMGGWMHHAAGPTMHQTTFQS--HPSVHSTLHQMQPPHPALNPHM- 96

Anolis_sagrei_ordinatus_Pleu SLI-GHQYPRYSFEPMGGWMHHASGPTMHQTAFQN--NPSMHPTFHQMQPQHPVLNPHM- 96

Phrynosoma_blainvillii_Pleu SLT-GHQYPHYSYEPMGGWMHHAAGPMMHQTHFPG--LSSVHMPLHQMPPQQSHLNPQR- 96

Sceloporus_chrysostictus_AMEL_Pleur SLI-GHQYPRYSYEPMGGWMHHATGPMMHQTHFPS--LSSAHTPLHQMPPQQPHLNPQM- 96

Urosaurus_nigricaudus_Pleu SLI-GHQYPRYSYEPMGGWMHHATGPMMHQTHFPS--VSSAHTPLHQMPPQQPHLNPQM- 96

Sceloporus_occidentalis_Pleu SLI-GHQYPRYSYEPMGGWMHHAAGPMMHQTHFPS--LPSGHTPLHQMPPQQPHLNPQM- 96

Sceloporus_tristichus_Pleu SLI-GHQYPRYSYEPMGGWMHHAAGPMMHQTHFPS--LPSGHTPLHQMPPQQPHLNPQM- 96

Sceloporus_undulatus_Pleu SLI-GHQYPRYSYEPMGGWMHHAAGPMMHQTHFPS--LPSGHTPLHQMPPQQPHLNPQM- 96

Gambelia_wislizenii_Pleu SLMGGHQYPRYGYEPMGGWMHHASGPMMHQAHFQS--FPPVHSPLHQMQPQQPHQNPQM- 97

Laemanctus_serratus_AMEL_Pleur SLM-GPQYPRYGYEPMGGWMHHATAPVVPQTHFQGLHLSPVHSPFHQMQPHQTSLNPQM- 98

Iguana_delicatissima_Pleu SLM-GPQYPRYGYEPMGGWMRHATGPMMHQTHIQN--LPPMHSPIHQMQPQHQILNPQM- 96

Cyclura_pinguis_Pleu SLM-GPQYPRYGYEPMGGWMRHATGPMMHQTHIQS--LPPMHSPIHHMQPQHQILNPQM- 96

Canis_lupus_dingo_AMEL NMI-RHPYPSYGYEPMGGWLHHQIIPVLSQQNPSN----------HALQPHHHIPMVPA- 91

Vulpes_vulpes_AMEL NMI-RHPYPSYGYEPMGGWLHHQIIPVLSQQNPSN----------HALQPHHHIPMVPA- 91

Meles_meles_AMEL NMI-RHPYPSYGYEPMGGWLHHQIIPVLSQQNPPN----------HALQPHHHIPMMPA- 91

Mustela_putorius_AMEL NMI-RHPYPSYGYEPMGGWLHHQIIPVLSQQNPPN----------HALQPHHHIPMMPA- 91

Echinops_telfairi_AMEL NML-RQPYPSYGYEPMGGWLHHQIIPVLSQQLPPS----------HTLQPHHHIPMVPA- 91

Phyllostomus_hastatus_AMEL SMI-RQPYTSYGYEPMGGWLHHQIIPVLSQQNPPN----------HALQPHHHIPVVPA- 108

Homo_sapiens_AMEL S-I-RPPYPSYGYEPMGGWLHHQIIPVLSQQHPPT----------HTLQPHHHIPVVPA- 90

Saimiri_boliviensis_AMEL SMI-RPAYPSYGYEPMGGWLHHQIIPVLSQQHPPT----------HTLQPHHHIPVVPA- 105

Erinaceus_europaeus_AMEL NMI-RPPYPSYGYEPMGGWLHHQIIPVLSQQNPPS----------HALQPHHHIPMVPA- 91

Ursus_arctos_AMEL N-I-RHPYPSYGYEPMGGWLHHQIIPVLSQQNPPN----------HALQPHHHIPMVPA- 104

Suncus_etruscus_AMEL NMI-RPPYPSYGYEPMGGWLHHQIIPVLSQQNPPN----------HALQPHHHIPLVPV- 91

Phacochoerus_africanus_AMEL NMI-RHPYTSYGYEPMGGWLHHQIIPVVSQQTPQS----------HALQPHHHIPMVPA- 105

. *. *.:******::* . : * * :

Acanthocercus_cyanogaster_AMEL_Acr QLP--GHNTLVPMTGQNTLVP--QYQPAHAGPVHQPLPPVAGE---------TPMHPQA- 141

Acanthocercus_minutus_AMLE_Acr QLP--GHNTLVPMTGQNTLVP--QYQPAHAGPVHQPLPPVAGE---------PPMHPQA- 141

Xenagama_zonura_AMLE_Acr QLP--GHNTLVPMTGQNTLVP--QYQPAHAGPVHQPLPPVAGE---------PPMHPQA- 141

Laudakia_wui_Acr QLP--GHNTLVPMTGQNTLVP--QYQPAHAGPVHQPLPPVAGE---------PPMHPQP- 141

Agama_doriae_AMLE_Acr QLP--GHNTFVPMTGQNTLVP--QYQPAHAGPAHQPLPPVAGE---------QPMHPQP- 141

Phrynocephalus_forsythii_Acr QLP--GHNTYVPMTGQNTLVP--QYQPAHAGPVHQPVPPVAGE---------PPMHPQP- 141

Phrynocephalus_guinanensis_Acr QLP--GHNTFVPMTGQNTLVP--QYQPAHAGPVHQPVPPVAGE---------PPMHPQP- 141

Phrynocephalus_putjatai_Acr QLP--GHNTFVPMTGQNTLVP--QYQPAHAGPVHQPVPPVAGE---------PPMHPQP- 141

Phrynocephalus_vlangalii_Acr QLP--GHNTFVPMTGQNTLVP--QYQPAHTGPVHQPVPPVAGE---------PPMHPQP- 141

Phrynocephalus_versicolor_Acr QLP--GHNTFVPMTGQNTLVP--QYQPAHAGPVHQPVPPVAGE---------TPMHPQP- 141

Intellagama_lesueurii_Acr QLP--GHNTLVPMTGQSTLMP--QYQPAHGGPVHQPFPPVAGEHPQLPAHPNQPMHPQL- 150

Pogona_vitticeps_Acr QLP--GHNTLVPMTGQSTLTP--QYQPAHAGPVHQPFPPVAGE---------APMQPQL- 141

Bradypodion_pumilum_Acr QQP-----------GQNTLMP--QYP---AGPVHQSLPPVAAE---------PPMHPQH- 129

Bradypodion_ventrale_Acr QQP-----------GQNTLMP--QYP---AGPVHQSLPPVAAE---------PPMHPQH- 129

Chamaeleo_calyptratus_AMEL_Acr QQP--GHNTLVPMTGQNTLMP--QYP---AGPVHQPLPPVAAE---------PPMHPQH- 138

Chamaeleo_laevigatus_AMEL_Acr QQP--GHNTLVPMTGQNTLMP--QYP---AGPVHQPLPPVAAE---------PPMHPQH- 138

Chamaeleo_gracilis_AMEL_Acr QQP--GHNTLVPMTGQNTLMP--QYP---AGPVHQPLPPVAAE---------PPMHPQH- 138

Chamaeleo_dilepis_AMEL_Acr QQP--GHNTLVPMTGQNTLMP--QYP---AGPVHQPLPPVAAE---------PPMHPQH- 138

Trioceros_affinis_AMEL_Acr QQP--GHNTLVPMTGQNTLMP--QYP---AGPVHQPLPPVAAE---------PPMHPQH- 138

Trioceros_balebicornutus_AMEL_Acr QQP--GHNILVPMTGQNTLMP--QYP---AGPVHQPLPPVAAE---------PPMHPQH- 138

Trioceros_harennae_AMEL_Acr QQP--GHNILVPMTGQNTLMP--QYP---AGPVHQPLPPVAAE---------PPMHPQH- 138

Furcifer_pardalis_Acr QQP--GHNTLVPMTGQNTLMP--QYP---AGPVHQPLPPVAAE---------PPMHPQH- 139

Anolis_apletophallus_Pleu QHP--GHNSFGPMPGTNQLMP--HFQPAHGGPINHPFPPHAGE----------PMHPQH- 141

Anolis_rodriguezii_AMEL_Pleur QQP--GHNSFGPMPGTNPLMP--HFQPAHGGPINHPFPPHAGE---------HPMHPQQ- 142

Anolis_tropidonotus_AMEL_Pleur QQP--GHNSFGPMPGSNTLMP--HFQPAHGGPINHPFPPHAGE---------HPMHPQQ- 142

Anolis_carolinensis_Pleu QPP--GHNPFGPMPGQNTLMP--QFQPAHGGPIHHPFQPHAGE---------HPMHPQQ- 142

Anolis_sagrei_ordinatus_Pleu QQP--GHNSFGPMAGPNTLMP--QFQPAHGGPIQHPFSPHAGE---------HPIHPQQ- 142

Phrynosoma_blainvillii_Pleu QQP--GLNPYVPFSGQNTLMP--HFQPANGASIQQPFPPHAGE---------HPMHPQQ- 142

Sceloporus_chrysostictus_AMEL_Pleur QQP--GHNPYVPFSGQNTLMP--HFQPVNGAPVQQPLPPHAGE---------HPIHPQQ- 142

Urosaurus_nigricaudus_Pleu QQP--GHNPYGLYPGQNTLMP--HFQPANGAPVQQPLPPHAGE---------HPIHPQQ- 142

Sceloporus_occidentalis_Pleu QQP--GHNPYVPFSGQNTLIP--HFQPSNGAHIQQPLSPHAGE---------HPMHPQQ- 142

Sceloporus_tristichus_Pleu QQP--GHNPYVPFSGQNTLIP--HFQPANGAHIQQPLSPHAGE---------HPMHPQQ- 142

Sceloporus_undulatus_Pleu QQP--GHNPYVPFSGQNTLIP--HFQPPNGAHIQQPLSPHAGE---------HPMHPQQ- 142

Gambelia_wislizenii_Pleu QQP--GPNQFLPQPGQNTLMP--PFQPAHGTPVQQPIQPHAGQ-----------MHPQQ- 141

Laemanctus_serratus_AMEL_Pleur HQP--GPNPFGPLPGQNTLMP--QFHPGHGAPVQQPLQPHVGE---------HPMHPQQ- 144

Iguana_delicatissima_Pleu PQP--GHNAFVPLPGQNTLMP--QFQPAHGAL-----PPQIGE---------HPMHPQQ- 137

Cyclura_pinguis_Pleu SQP--GHNTFVPLPGQNALMP--QFQPAHGGP----LPPQIGE---------HPTHSQQ- 138

Canis_lupus_dingo_AMEL QQPVVPQQPMMPVPGQHSMTPTQHHQPNLPLPAQQPFQPQPVQ----P----QPHQPIQ- 142

Vulpes_vulpes_AMEL QQPVVPQQPMMPVPGQHSMTPTQHHQPNLPLPAQQPFQPQPVQ----P----QPHQPIQ- 142

Meles_meles_AMEL QQPVVPQQPMMPVPGQHSMTPTQHHQPNLPLPAQQPFQPQPVQ----P----QAHQPIQ- 142

Mustela_putorius_AMEL QQPVVPQQPMMPVPGQHSMTPTQHHQPNLPLPAQQPFQPQPVQ----P----QAHQPIQ- 142

Echinops_telfairi_AMEL QQPVAPQQPMMPVPGQHSMTPTQHHQPHLPPPVQQPYQPQPVQ----P----QPHQPMQ- 142

Phyllostomus_hastatus_AMEL QQPVAPQQPMMPVPGQHSMTPTQHHQPNLLPPAQQPFQPQTVQ----P----QPHQPIQ- 159

Homo_sapiens_AMEL QQPVIPQQPMMPVPGQHSMTPIQHHQPNLPPPAQQPYQPQPVQ----P----QPHQPMQ- 141

Saimiri_boliviensis_AMEL QQPVVPQQPMMPVPGQHSMIPTQHHQPNLPPPAQQPFQPQTVQ----P----QPHQPMQ- 156

Erinaceus_europaeus_AMEL QQPVVPQQPMMPVPGQHSMTPTQHHQSNLPAPAQQPYQPQPVQ----P----QPHQPMQ- 142

Ursus_arctos_AMEL QQPVVPQQPVMPVPGQHSMTPTQHHQPNLPLPAQQPFQPQPVQ----P----QPH----- 151

Suncus_etruscus_AMEL QQPVVPQQPMMPIPGQHSMTPTQHHQPNVPSPAQQPFQPQPVQ----P----QPHQPIQH 143

Phacochoerus_africanus_AMEL QQPGIPQQPMMPLPGQHSMTPTQHHQPNLPLPAQQPFQPQPVQ----P----QPHQPLQ- 156

* * : * . * :

Acanthocercus_cyanogaster_AMEL_Acr PAHPNQPMH------PQP------------PNPPMYPMQPLPPLIPDRPLESWPVPDKTK 183

Acanthocercus_minutus_AMLE_Acr PAHPNQPMH------PQP------------PNPPMYPMQPLPPLIPDRPLESWPVPDKTK 183

Xenagama_zonura_AMLE_Acr PAHPNQPMH------PQP------------PNPPMYPMQPLPPLIPDRPLESWPVPDKTK 183

Laudakia_wui_Acr PAQPNQPMH------PQP------------PNPPMHPMQPLPPLIPDIPLESWPVPDKTK 183

Agama_doriae_AMLE_Acr PAHPNQPMH------PQP------------PNPPMFPMQPLPPLIPDRPLESWPVPDKTK 183

Phrynocephalus_forsythii_Acr PAHPNQPMH------PQL------------PNPPMYPVQPLPPLIPDRPLESWPVPDKTK 183

Phrynocephalus_guinanensis_Acr PAHPNQPMH------PQL------------PNPPMYPVQPLPPMIPDRPLESWPVPDKTK 183

Phrynocephalus_putjatai_Acr PAHPNQPMH------PQL------------PNPPMYPVQPLPPLIPDRPLESWPVPDKTK 183

Phrynocephalus_vlangalii_Acr PAHPNQPMH------PQL------------PNPPMYPVQPLPPLIPDRPLESWPVPDKTK 183

Phrynocephalus_versicolor_Acr PAHPNQPMH------PQPPAHPNQPMHPQLPNPPMYPVQPLPPLIPDRPLESWPVPDKTK 195

Intellagama_lesueurii_Acr PAHPNQPMH------PQLPAH---------PNQPMFPMQPLPPLMPDTPLESWPVPDKTK 195

Pogona_vitticeps_Acr PAHPNQPMH------PQLPAH---------PNQPMFPMQPLPPLMPDTPLESWPVPDKTK 186

Bradypodion_pumilum_Acr PANPNQPMN------PQSPMN---------PNAPMFPMQPLPPLMPDTPLESWPAADKTK 174

Bradypodion_ventrale_Acr PANPNQPMN------PQSPMN---------PNAPMFPMQPLPPLMPDTPLESWPAADKTK 174

Chamaeleo_calyptratus_AMEL_Acr PANPNQPMN------PQSPMN---------PNAPMFPMQPLPPLMPDAPLESWPAADKTK 183

Chamaeleo_laevigatus_AMEL_Acr PANPNQPMN------PQSPMN---------PNAPMFPMQPLPPLMPDAPLESWPAADKTK 183

Chamaeleo_gracilis_AMEL_Acr PANPNQPMN------PQSPMN---------PNAPMFPMQPLPPLMPDTPLESWPAADKTK 183

Chamaeleo_dilepis_AMEL_Acr PANPNQQMN------PQSPMN---------PNAPMFPMQPLPPLMPDTPLESWPAADKTK 183

Trioceros_affinis_AMEL_Acr PANPNQPMN------PQSPMN---------PNAPMFPMQPLPPLMPDTPLESWPNADKTK 183

Trioceros_balebicornutus_AMEL_Acr PANPNQPMN------PQSPMN---------PNAPMFPMQPLPPLMPDTPLESWPNADKTK 183

Trioceros_harennae_AMEL_Acr PANPNQPMN------PQSPMN---------PNAPMFPMQPLPPLMPDTPLESWPNADKTK 183

Furcifer_pardalis_Acr PANPNQPMN------PQSPMN---------PNAPMFPMQPLPPLMPDTPLESWPAVDKTK 184

Anolis_apletophallus_Pleu PGNPIHPMH------PQQPAN---------PNPPIYSVQQMPPVISDTPLESWPPADKTK 186

Anolis_rodriguezii_AMEL_Pleur PGNPIHPMH------PQQPAN---------PNPPIYSVQQMPPVISDTPLESWPPADKTK 187

Anolis_tropidonotus_AMEL_Pleur PGNPIHPMH------PQQPAN---------PNPPIYSVQQMPPVIADTPLESWPPADKTK 187

Anolis_carolinensis_Pleu TGNPVHPMH------PQQPAN---------PNSPIYPVQQLPPLISDTPLESWPPADKTK 187

Anolis_sagrei_ordinatus_Pleu HGNPIHPMH------PQQPAN---------PNPPIYPVQPLPPLISDTPLETWPPADKTK 187

Phrynosoma_blainvillii_Pleu PGNPIQPMH------PQQPAN---------PNQPVYPVLQLPPMVPDTPLESWPPADKTK 187

Sceloporus_chrysostictus_AMEL_Pleur PGNPIQPMY------PQQPAN---------PNQPMFPVQQLPPMVPDTPLESWPAADKTK 187

Urosaurus_nigricaudus_Pleu PGNPIQPMY------PQQPAN---------PNQPMFPVQQLPPMVPDTPLESWPAADKTK 187

Sceloporus_occidentalis_Pleu PGNPIQPMY------PQQPAN---------PNQPMFPVQQLPPMVPDTPLESWPAADKTK 187

Sceloporus_tristichus_Pleu PGNPIQPMY------PQQPAN---------PNQPMFPVQQLPPMVPDTPLESWPAADKTK 187

Sceloporus_undulatus_Pleu PGNPIQPMY------PQQPAN---------PNQPMFPVQQLPPMVPDTPLESWPAADKTK 187

Gambelia_wislizenii_Pleu PGNPIQPMY------PQQPTN---------PNSPMYPIHPVPPLIPDTPLEPWPPADKTK 186

Laemanctus_serratus_AMEL_Pleur PGHPIQPMH------PQQPAN---------PNPPMYPVHPLPPLVPDTPLESWPPADKTK 189

Iguana_delicatissima_Pleu HGNPMQPIH------PQQPAN---------PNPPMYPVQPLAPVLPDTPLESWPAADKTK 182

Cyclura_pinguis_Pleu HGNPTQPIH------PQQPGN---------PNPPMYPVQPFAPVIPDTPLESWPAPDKTK 183

Canis_lupus_dingo_AMEL PQPPVHPIQ------PLPPQP---------PLPPMFPIQPLPPMLPDLPLEAWPATDKTK 187

Vulpes_vulpes_AMEL PQPPVHPIQ------PLPPQP---------PLPPMFPIQPLPPMLPDLPLEAWPATDKTK 187

Meles_meles_AMEL PQPPVHPIQ------PLPPQP---------PLPPMFPIQPLPPMLPDLPLEAWPATDKTK 187

Mustela_putorius_AMEL PQPPVHPIQ------PLPPQP---------PLPPMFPIQPLPPMLPDLPLEAWPATDKTK 187

Echinops_telfairi_AMEL PQPPVHPMQ------PMPPQP---------PMPPMFPMQPLPPMLTDLPMEAWPATDKTK 187

Phyllostomus_hastatus_AMEL PQPPVHPIQ------PLPPQP---------PLPPMFPMQPLPPMLPDLPLEAWPATDKTK 204

Homo_sapiens_AMEL PQPPVHPMQ------PLPPQP---------PLPPMFPMQPLPPMLPDLTLEAWPSTDKTK 186

Saimiri_boliviensis_AMEL PQPPVHPMQ------PLPPQP---------PLPPMFPMQPLPPMLPDLPLEAWPATDKTK 201

Erinaceus_europaeus_AMEL PMAPMQPMQPQSPMHPLPPQP---------PLHPIFPIQSMPAMLPDLPLEAWPATDKTK 193

Ursus_arctos_AMEL -----HPIQ------PLPPQP---------PLPPMFPIQPLPPMLPDLPLEAWPATDKTK 191

Suncus_etruscus_AMEL PQPPMHPMQ------PLPQQP---------PLNPIFPMQPM-GVIPDIPMEAWPATDKTK 187

Phacochoerus_africanus_AMEL PQSPMHPIQ------PLLPQP---------PLPPMFSMQSL---LPDLPLEAWPATDKTK 198

: : * * *:..: . :.* .:*.** ****

Acanthocercus_cyanogaster_AMEL_Acr QEEV 187

Acanthocercus_minutus_AMLE_Acr QEEV 187

Xenagama_zonura_AMLE_Acr QEEV 187

Laudakia_wui_Acr QEEV 187

Agama_doriae_AMLE_Acr QEEV 187

Phrynocephalus_forsythii_Acr QEEV 187

Phrynocephalus_guinanensis_Acr QEEV 187

Phrynocephalus_putjatai_Acr QEEV 187

Phrynocephalus_vlangalii_Acr QEEV 187

Phrynocephalus_versicolor_Acr QEEV 199

Intellagama_lesueurii_Acr QEEV 199

Pogona_vitticeps_Acr QEEV 190

Bradypodion_pumilum_Acr QEEV 178

Bradypodion_ventrale_Acr QEEV 178

Chamaeleo_calyptratus_AMEL_Acr QEEV 187

Chamaeleo_laevigatus_AMEL_Acr QEEV 187

Chamaeleo_gracilis_AMEL_Acr QEEV 187

Chamaeleo_dilepis_AMEL_Acr QEEV 187

Trioceros_affinis_AMEL_Acr QEEM 187

Trioceros_balebicornutus_AMEL_Acr QEEV 187

Trioceros_harennae_AMEL_Acr QEEV 187

Furcifer_pardalis_Acr QEEV 188

Anolis_apletophallus_Pleu QEEV 190

Anolis_rodriguezii_AMEL_Pleur QEEV 191

Anolis_tropidonotus_AMEL_Pleur QEEV 191

Anolis_carolinensis_Pleu QEEV 191

Anolis_sagrei_ordinatus_Pleu QEEV 191

Phrynosoma_blainvillii_Pleu QEEV 191

Sceloporus_chrysostictus_AMEL_Pleur QEEV 191

Urosaurus_nigricaudus_Pleu QEEV 191

Sceloporus_occidentalis_Pleu QEEV 191

Sceloporus_tristichus_Pleu QEEV 191

Sceloporus_undulatus_Pleu QEEV 191

Gambelia_wislizenii_Pleu QEEV 190

Laemanctus_serratus_AMEL_Pleur QEEV 193

Iguana_delicatissima_Pleu QEEV 186

Cyclura_pinguis_Pleu QEEV 187

Canis_lupus_dingo_AMEL REEV 191

Vulpes_vulpes_AMEL REEV 191

Meles_meles_AMEL REEV 191

Mustela_putorius_AMEL REEV 191

Echinops_telfairi_AMEL REEV 191

Phyllostomus_hastatus_AMEL REEV 208

Homo_sapiens_AMEL REEV 190

Saimiri_boliviensis_AMEL REEV 205

Erinaceus_europaeus_AMEL REEV 197

Ursus_arctos_AMEL REEV 195

Suncus_etruscus_AMEL REEV 191

Phacochoerus_africanus_AMEL REEV 202

:**:

**AMEL-AA-MAFFT-Iguania+Mammal – DIVERGE-Acro-Pleuro DIVERGE-Agam-CHam**

CLUSTAL W (1.8) multiple sequence alignment (ALTER 1.3.3)

Acanthocercus_cyanogaster_AMEL_Acr MEGWTLIMCLLCTTLAVPLPQHPGFINFSYEVMTPLKWYQGVM-GHQYPRYGYEPMGGWM 59

Acanthocercus_minutus_AMLE_Acr MEGWTLIMCLLCTTLAVPLPQHPGFINFSYEVMTPLKWYQGVM-GHQYPRYGYEPMGGWM 59

Xenagama_zonura_AMLE_Acr MEGWTLIMCLLCTTLAVPLPQHPGFINFSYEVMTPLKWYQGVM-GHQYPRYGYEPMGGWM 59

Laudakia_wui_Acr MEGWTLIMCLLCTTLAVPLPQHPGFINFSYEVMTPLKWYQGVM-GHQYPRYGYEPMGGWM 59

Agama_doriae_AMLE_Acr MEGWTLIMCLLGTTLAVPLPQHPGFINFSYEVMTPLKWYQGVM-GHQYPRYGYEPMGGWM 59

Phrynocephalus_forsythii_Acr MEGWILIMCVLSTTLAVPLPQHPGFINFSYEVMTPLKWYQGVM-GHQYPRYGYEPMGGWM 59

Phrynocephalus_guinanensis_Acr MEGWILIMCVLSTTLAVPLPQHPGFINFSYEVMTPLKWYQGVM-GHQYPRYGYEPMGGWM 59

Phrynocephalus_putjatai_Acr MEGWILIMCVLSTTLAVPLPQHPGFINFSYEVMTPLKWYQGVM-GHQYPRYGYEPMGGWM 59

Phrynocephalus_vlangalii_Acr MEGWILIMCVLSTTLAVPLPQHPGFINFSYEVMTPLKWYQGVM-GHQYPRYGYEPMGGWM 59

Phrynocephalus_versicolor_Acr MEGWTLIMCLLSTTLAVPLPQHPGFINFSYEVMTPLKWYQGVM-GHQYPRYGYEPMGGWM 59

Intellagama_lesueurii_Acr MEGWTLIMSLLCTTLAIPLPQHPGFINFSYEVMTPLKWYQGVM-GHQYPRYGYEPMGGWM 59

Pogona_vitticeps_Acr MEGWTLIMSLLCTTLAIPLPQHPGFINFSYEVMTPLKWYQGVM-GHQYPRYGYEPMGGWM 59

Bradypodion_pumilum_Acr MECLVLIMSLLCTTFAIPLPQHPGFINFSYEVMTPLKWYQGLM-GQQYPRYGYEPMGGWM 59

Bradypodion_ventrale_Acr MECLVLIMSLLCTTFAIPLPQHPGFINFSYEVMTPLKWYQGLM-GQQYPRYGYEPMGGWM 59

Chamaeleo_calyptratus_AMEL_Acr MECLVLIMSLLCTTFAIPLPQHPGFINFSYEVMTPLKWYQGLM-GQQYPRYGYEPMGGWM 59

Chamaeleo_laevigatus_AMEL_Acr MECLVLIMSLLCTTFAIPLPQHPGFINFSYEVMTPLKWYQGLM-GQQYPRYGYEPMGGWM 59

Chamaeleo_gracilis_AMEL_Acr MECLVLIMSLLCTTFAIPLPQHPGFINFSYEVMTPLKWYQGLM-GQQYPRYGYEPMGGWM 59

Chamaeleo_dilepis_AMEL_Acr MECLVLIMSLLCTTFAIPLPQHPGFINFSYEVMTPLKWYQGLM-GQQYPRYGYEPMGGWM 59

Trioceros_affinis_AMEL_Acr MECLVLIMSLLCTTFAIPLPQHPGFINFSYEVMTPLKWYQGLM-GQQYPRYGYEPMGGWM 59

Trioceros_balebicornutus_AMEL_Acr MECLVLIMSLLCTTFAIPLPQHPGFINFSYEVMTPLKWYQGLM-GQQYPRYGYEPMGGWM 59

Trioceros_harennae_AMEL_Acr MECLVLIMSLLCTTFAIPLPQHPGFINFSYEVMTPLKWYQGLM-GQQYPRYGYEPMGGWM 59

Furcifer_pardalis_Acr MECLVLIMSLLCTTFAIPLPQHPGFINFSYEVMTPLKWYQGLM-GQQYPRYGYEPMGGWM 59

Anolis_apletophallus_Pleu MEGWTLIMCLLSTTFAMPLPQHPGYINFSYEVMTPLKWYQSLV-GHQYPRYGFEPMGGWM 59

Anolis_rodriguezii_AMEL_Pleur MEGWTLVMCLLSTTFAMPLPQHPGYVNFSYEVMTPLKWYQSLI-GHQYPRYGFEPMGGWM 59

Anolis_tropidonotus_AMEL_Pleur MEGWTLVMCLLSTTLAIPLPQHPGYVNFSYEVMTPLKWYQSLI-GHQYPRYGFEPMGGWM 59

Anolis_carolinensis_Pleu MEGWTLVMCLLSTTFAIPLPQHPGYINFSYEVMTPLKWYQSLI-GHQYPRYGFEPMGGWM 59

Anolis_sagrei_ordinatus_Pleu MEGWTLIVCLLSTTLAMPLPQHPGYVNFSYEVMTPLKWYQSLI-GHQYPRYSFEPMGGWM 59

Phrynosoma_blainvillii_Pleu MEGWTLVMCLLSTTFAIPLPQHPGFINFSYEVMTPLKWYQSLT-GHQYPHYSYEPMGGWM 59

Sceloporus_chrysostictus_AMEL_Pleur MEGWTLVMCLLSTTFAIPLPQHPGFINFSYEVMTPLKWYQSLI-GHQYPRYSYEPMGGWM 59

Urosaurus_nigricaudus_Pleu MEGWTLVMCLLSTTFAIPLPQHPGFINFSYEVMTPLKWYQSLI-GHQYPRYSYEPMGGWM 59

Sceloporus_occidentalis_Pleu MEGWTLVMCLLSTTFAIPLPQHPGFINFSYEVMTPLKWYQSLI-GHQYPRYSYEPMGGWM 59

Sceloporus_tristichus_Pleu MEGWTLVMCLLSTTFAIPLPQHPGFINFSYEVMTPLKWYQSLI-GHQYPRYSYEPMGGWM 59

Sceloporus_undulatus_Pleu MEGWTLVMCLLSTTFAIPLPQHPGFINFSYEVMTPLKWYQSLI-GHQYPRYSYEPMGGWM 59

Gambelia_wislizenii_Pleu MEGWTLVMCLLSTSFAIPLPQHPGFINFSYEVMTPLKWYQSLMGGHQYPRYGYEPMGGWM 60

Laemanctus_serratus_AMEL_Pleur MEIWTLVMCLLSTTFAIPLPQHPGFINFSYEVMTPLKWYQSLM-GPQYPRYGYEPMGGWM 59

Iguana_delicatissima_Pleu MGGWTLVMCLLCTTLAIPLPQHPGFINFSYEVMTPLKWYQSLM-GPQYPRYGYEPMGGWM 59

Cyclura_pinguis_Pleu MGGWTLVMCLLCTTLAIPLPQHPGFINFSYEVMTPLKWYQSLM-GPQYPRYGYEPMGGWM 59

* *::.:* *::*:*******::**************.: * ***:*.:*******

Acanthocercus_cyanogaster_AMEL_Acr HHSPGSMMHQSPFQT--LHPMPPPLHQVQ-QQPPLNPHM-QLPGHNTLVPMTGQNTLVPQ 115

Acanthocercus_minutus_AMLE_Acr HHSPGSMMHQSPFQT--LHPMPPPLHQVQ-QQPPLNPHM-QLPGHNTLVPMTGQNTLVPQ 115

Xenagama_zonura_AMLE_Acr HHSPGSMMHQSPFQT--LHPMPPPLHQVQ-QQPPLNPHM-QLPGHNTLVPMTGQNTLVPQ 115

Laudakia_wui_Acr HHSPGSMMHQSPFQT--LHPMPPPLHQVQ-QQPPLNPHM-QLPGHNTLVPMTGQNTLVPQ 115

Agama_doriae_AMLE_Acr HHSPGSMMHQSPFQT--LHPMPPPLHQVQ-QQPPLNPHM-QLPGHNTFVPMTGQNTLVPQ 115

Phrynocephalus_forsythii_Acr HHSPGSMMHQSPFQA--LHPMPPPLHQVQ-QQPPLNPHM-QLPGHNTYVPMTGQNTLVPQ 115

Phrynocephalus_guinanensis_Acr HHSPGSMMHQSPFQA--LHPMPPPLHQVQ-QQPPLNPHM-QLPGHNTFVPMTGQNTLVPQ 115

Phrynocephalus_putjatai_Acr HHSPGSMMHQSPFQA--LHPMPPPLHQVQ-QQPPLNPHM-QLPGHNTFVPMTGQNTLVPQ 115

Phrynocephalus_vlangalii_Acr HHSPGSMMHQSPFQA--LHPMPPPLHQVQ-QQPPLNPHM-QLPGHNTFVPMTGQNTLVPQ 115

Phrynocephalus_versicolor_Acr HHSPGSMMHQSPFQT--LHPMPPPLHQVQ-QQPPLNPHM-QLPGHNTFVPMTGQNTLVPQ 115

Intellagama_lesueurii_Acr HHSPGSMMHQTPFQT--LHPMPPPLHQVQ-QQPPLNPHM-QLPGHNTLVPMTGQSTLMPQ 115

Pogona_vitticeps_Acr HHSPGSMMHQAPFQT--LHPMPPPLHQVQ-QQPPLNPHM-QLPGHNTLVPMTGQSTLTPQ 115

Bradypodion_pumilum_Acr HHSPGPMMHQTPFQS--LHPMSPPLHQMQ-QLPPLNPHM-QQP---------GQNTLMPQ 106

Bradypodion_ventrale_Acr HHSPGPMMHQTPFQS--LHPMSPPLHQMQ-QLPPLNPHM-QQP---------GQNTLMPQ 106

Chamaeleo_calyptratus_AMEL_Acr HHSPGPMMHQTPFQS--LHPMSPPLHQMQ-QLPPLNPHL-QQPGHNTLVPMTGQNTLMPQ 115

Chamaeleo_laevigatus_AMEL_Acr HHSPGPMMHQTPFQS--LHPMSPPLHQMQ-QLPPLNPHL-QQPGHNTLVPMTGQNTLMPQ 115

Chamaeleo_gracilis_AMEL_Acr HHSPGPMMHQTPFQS--LHPMSPPLHQMQ-QLPPLNPHL-QQPGHNTLVPMTGQNTLMPQ 115

Chamaeleo_dilepis_AMEL_Acr HHSPGPMMHQTPFQS--LHPMSPPLHQMQ-QLPPLNPHV-QQPGHNTLVPMTGQNTLMPQ 115

Trioceros_affinis_AMEL_Acr HHSPGPMMHQTPFQS--LHPMSPPLHQIQ-QLSPLNPHM-QQPGHNTLVPMTGQNTLMPQ 115

Trioceros_balebicornutus_AMEL_Acr HHSPGPMMHQTPFQS--LHPMSPPLHQIQ-QLPPLNPHM-QQPGHNILVPMTGQNTLMPQ 115

Trioceros_harennae_AMEL_Acr HHSPGPMMHQTPFQS--LHPMSPPLHQIQ-QLPPLNPHM-QQPGHNILVPMTGQNTLMPQ 115

Furcifer_pardalis_Acr HHSPGPMMHQTPFQS--LHPMSPPLHQIQ-QLPPLNPHMQQQPGHNTLVPMTGQNTLMPQ 116

Anolis_apletophallus_Pleu HHASGPTMPQTTFQS--HPSMHSTFHQMQPQHPALNPHM-QHPGHNSFGPMPGTNQLMPH 116

Anolis_rodriguezii_AMEL_Pleur HHASGPTMPQTPFQS--HPSMHSTFHQMQPQHPGLNPHM-QQPGHNSFGPMPGTNPLMPH 116

Anolis_tropidonotus_AMEL_Pleur HHASGPTMPQTTFQN--HPSMHSTFHQMQPQHPALNPHM-QQPGHNSFGPMPGSNTLMPH 116

Anolis_carolinensis_Pleu HHAAGPTMHQTTFQS--HPSVHSTLHQMQPPHPALNPHM-QPPGHNPFGPMPGQNTLMPQ 116

Anolis_sagrei_ordinatus_Pleu HHASGPTMHQTAFQN--NPSMHPTFHQMQPQHPVLNPHM-QQPGHNSFGPMAGPNTLMPQ 116

Phrynosoma_blainvillii_Pleu HHAAGPMMHQTHFPG--LSSVHMPLHQMPPQQSHLNPQR-QQPGLNPYVPFSGQNTLMPH 116

Sceloporus_chrysostictus_AMEL_Pleur HHATGPMMHQTHFPS--LSSAHTPLHQMPPQQPHLNPQM-QQPGHNPYVPFSGQNTLMPH 116

Urosaurus_nigricaudus_Pleu HHATGPMMHQTHFPS--VSSAHTPLHQMPPQQPHLNPQM-QQPGHNPYGLYPGQNTLMPH 116

Sceloporus_occidentalis_Pleu HHAAGPMMHQTHFPS--LPSGHTPLHQMPPQQPHLNPQM-QQPGHNPYVPFSGQNTLIPH 116

Sceloporus_tristichus_Pleu HHAAGPMMHQTHFPS--LPSGHTPLHQMPPQQPHLNPQM-QQPGHNPYVPFSGQNTLIPH 116

Sceloporus_undulatus_Pleu HHAAGPMMHQTHFPS--LPSGHTPLHQMPPQQPHLNPQM-QQPGHNPYVPFSGQNTLIPH 116

Gambelia_wislizenii_Pleu HHASGPMMHQAHFQS--FPPVHSPLHQMQPQQPHQNPQM-QQPGPNQFLPQPGQNTLMPP 117

Laemanctus_serratus_AMEL_Pleur HHATAPVVPQTHFQGLHLSPVHSPFHQMQPHQTSLNPQM-HQPGPNPFGPLPGQNTLMPQ 118

Iguana_delicatissima_Pleu RHATGPMMHQTHIQN--LPPMHSPIHQMQPQHQILNPQM-PQPGHNAFVPLPGQNTLMPQ 116

Cyclura_pinguis_Pleu RHATGPMMHQTHIQS--LPPMHSPIHHMQPQHQILNPQM-SQPGHNTFVPLPGQNALMPQ 116

:*:... : *: : . .:*:: **: * * . * *

Acanthocercus_cyanogaster_AMEL_Acr YQPAHAGPVHQPLPPVAGE---------TPMHPQAPAHPNQPMHPQ------------PP 154

Acanthocercus_minutus_AMLE_Acr YQPAHAGPVHQPLPPVAGE---------PPMHPQAPAHPNQPMHPQ------------PP 154

Xenagama_zonura_AMLE_Acr YQPAHAGPVHQPLPPVAGE---------PPMHPQAPAHPNQPMHPQ------------PP 154

Laudakia_wui_Acr YQPAHAGPVHQPLPPVAGE---------PPMHPQPPAQPNQPMHPQ------------PP 154

Agama_doriae_AMLE_Acr YQPAHAGPAHQPLPPVAGE---------QPMHPQPPAHPNQPMHPQ------------PP 154

Phrynocephalus_forsythii_Acr YQPAHAGPVHQPVPPVAGE---------PPMHPQPPAHPNQPMHPQ------------LP 154

Phrynocephalus_guinanensis_Acr YQPAHAGPVHQPVPPVAGE---------PPMHPQPPAHPNQPMHPQ------------LP 154

Phrynocephalus_putjatai_Acr YQPAHAGPVHQPVPPVAGE---------PPMHPQPPAHPNQPMHPQ------------LP 154

Phrynocephalus_vlangalii_Acr YQPAHTGPVHQPVPPVAGE---------PPMHPQPPAHPNQPMHPQ------------LP 154

Phrynocephalus_versicolor_Acr YQPAHAGPVHQPVPPVAGE---------TPMHPQPPAHPNQPMHPQPPAHPNQPMHPQLP 166

Intellagama_lesueurii_Acr YQPAHGGPVHQPFPPVAGEHPQLPAHPNQPMHPQLPAHPNQPMHPQLPA---------HP 166

Pogona_vitticeps_Acr YQPAHAGPVHQPFPPVAGE---------APMQPQLPAHPNQPMHPQLPA---------HP 157

Bradypodion_pumilum_Acr YP---AGPVHQSLPPVAAE---------PPMHPQHPANPNQPMNPQSPM---------NP 145

Bradypodion_ventrale_Acr YP---AGPVHQSLPPVAAE---------PPMHPQHPANPNQPMNPQSPM---------NP 145

Chamaeleo_calyptratus_AMEL_Acr YP---AGPVHQPLPPVAAE---------PPMHPQHPANPNQPMNPQSPM---------NP 154

Chamaeleo_laevigatus_AMEL_Acr YP---AGPVHQPLPPVAAE---------PPMHPQHPANPNQPMNPQSPM---------NP 154

Chamaeleo_gracilis_AMEL_Acr YP---AGPVHQPLPPVAAE---------PPMHPQHPANPNQPMNPQSPM---------NP 154

Chamaeleo_dilepis_AMEL_Acr YP---AGPVHQPLPPVAAE---------PPMHPQHPANPNQQMNPQSPM---------NP 154

Trioceros_affinis_AMEL_Acr YP---AGPVHQPLPPVAAE---------PPMHPQHPANPNQPMNPQSPM---------NP 154

Trioceros_balebicornutus_AMEL_Acr YP---AGPVHQPLPPVAAE---------PPMHPQHPANPNQPMNPQSPM---------NP 154

Trioceros_harennae_AMEL_Acr YP---AGPVHQPLPPVAAE---------PPMHPQHPANPNQPMNPQSPM---------NP 154

Furcifer_pardalis_Acr YP---AGPVHQPLPPVAAE---------PPMHPQHPANPNQPMNPQSPM---------NP 155

Anolis_apletophallus_Pleu FQPAHGGPINHPFPPHAGE----------PMHPQHPGNPIHPMHPQQPA---------NP 157

Anolis_rodriguezii_AMEL_Pleur FQPAHGGPINHPFPPHAGE---------HPMHPQQPGNPIHPMHPQQPA---------NP 158

Anolis_tropidonotus_AMEL_Pleur FQPAHGGPINHPFPPHAGE---------HPMHPQQPGNPIHPMHPQQPA---------NP 158

Anolis_carolinensis_Pleu FQPAHGGPIHHPFQPHAGE---------HPMHPQQTGNPVHPMHPQQPA---------NP 158

Anolis_sagrei_ordinatus_Pleu FQPAHGGPIQHPFSPHAGE---------HPIHPQQHGNPIHPMHPQQPA---------NP 158

Phrynosoma_blainvillii_Pleu FQPANGASIQQPFPPHAGE---------HPMHPQQPGNPIQPMHPQQPA---------NP 158

Sceloporus_chrysostictus_AMEL_Pleur FQPVNGAPVQQPLPPHAGE---------HPIHPQQPGNPIQPMYPQQPA---------NP 158

Urosaurus_nigricaudus_Pleu FQPANGAPVQQPLPPHAGE---------HPIHPQQPGNPIQPMYPQQPA---------NP 158

Sceloporus_occidentalis_Pleu FQPSNGAHIQQPLSPHAGE---------HPMHPQQPGNPIQPMYPQQPA---------NP 158

Sceloporus_tristichus_Pleu FQPANGAHIQQPLSPHAGE---------HPMHPQQPGNPIQPMYPQQPA---------NP 158

Sceloporus_undulatus_Pleu FQPPNGAHIQQPLSPHAGE---------HPMHPQQPGNPIQPMYPQQPA---------NP 158

Gambelia_wislizenii_Pleu FQPAHGTPVQQPIQPHAGQ-----------MHPQQPGNPIQPMYPQQPT---------NP 157

Laemanctus_serratus_AMEL_Pleur FHPGHGAPVQQPLQPHVGE---------HPMHPQQPGHPIQPMHPQQPA---------NP 160

Iguana_delicatissima_Pleu FQPAHGA-----LPPQIGE---------HPMHPQQHGNPMQPIHPQQPA---------NP 153

Cyclura_pinguis_Pleu FQPAHGGP----LPPQIGE---------HPTHSQQHGNPTQPIHPQQPG---------NP 154

: . * .: :.* .:* : : ** *

Acanthocercus_cyanogaster_AMEL_Acr NPPMYPMQPLPPLIPDRPLESWPVPDKTKQEEV 187

Acanthocercus_minutus_AMLE_Acr NPPMYPMQPLPPLIPDRPLESWPVPDKTKQEEV 187

Xenagama_zonura_AMLE_Acr NPPMYPMQPLPPLIPDRPLESWPVPDKTKQEEV 187

Laudakia_wui_Acr NPPMHPMQPLPPLIPDIPLESWPVPDKTKQEEV 187

Agama_doriae_AMLE_Acr NPPMFPMQPLPPLIPDRPLESWPVPDKTKQEEV 187

Phrynocephalus_forsythii_Acr NPPMYPVQPLPPLIPDRPLESWPVPDKTKQEEV 187

Phrynocephalus_guinanensis_Acr NPPMYPVQPLPPMIPDRPLESWPVPDKTKQEEV 187

Phrynocephalus_putjatai_Acr NPPMYPVQPLPPLIPDRPLESWPVPDKTKQEEV 187

Phrynocephalus_vlangalii_Acr NPPMYPVQPLPPLIPDRPLESWPVPDKTKQEEV 187

Phrynocephalus_versicolor_Acr NPPMYPVQPLPPLIPDRPLESWPVPDKTKQEEV 199

Intellagama_lesueurii_Acr NQPMFPMQPLPPLMPDTPLESWPVPDKTKQEEV 199

Pogona_vitticeps_Acr NQPMFPMQPLPPLMPDTPLESWPVPDKTKQEEV 190

Bradypodion_pumilum_Acr NAPMFPMQPLPPLMPDTPLESWPAADKTKQEEV 178

Bradypodion_ventrale_Acr NAPMFPMQPLPPLMPDTPLESWPAADKTKQEEV 178

Chamaeleo_calyptratus_AMEL_Acr NAPMFPMQPLPPLMPDAPLESWPAADKTKQEEV 187

Chamaeleo_laevigatus_AMEL_Acr NAPMFPMQPLPPLMPDAPLESWPAADKTKQEEV 187

Chamaeleo_gracilis_AMEL_Acr NAPMFPMQPLPPLMPDTPLESWPAADKTKQEEV 187

Chamaeleo_dilepis_AMEL_Acr NAPMFPMQPLPPLMPDTPLESWPAADKTKQEEV 187

Trioceros_affinis_AMEL_Acr NAPMFPMQPLPPLMPDTPLESWPNADKTKQEEM 187

Trioceros_balebicornutus_AMEL_Acr NAPMFPMQPLPPLMPDTPLESWPNADKTKQEEV 187

Trioceros_harennae_AMEL_Acr NAPMFPMQPLPPLMPDTPLESWPNADKTKQEEV 187

Furcifer_pardalis_Acr NAPMFPMQPLPPLMPDTPLESWPAVDKTKQEEV 188

Anolis_apletophallus_Pleu NPPIYSVQQMPPVISDTPLESWPPADKTKQEEV 190

Anolis_rodriguezii_AMEL_Pleur NPPIYSVQQMPPVISDTPLESWPPADKTKQEEV 191

Anolis_tropidonotus_AMEL_Pleur NPPIYSVQQMPPVIADTPLESWPPADKTKQEEV 191

Anolis_carolinensis_Pleu NSPIYPVQQLPPLISDTPLESWPPADKTKQEEV 191

Anolis_sagrei_ordinatus_Pleu NPPIYPVQPLPPLISDTPLETWPPADKTKQEEV 191

Phrynosoma_blainvillii_Pleu NQPVYPVLQLPPMVPDTPLESWPPADKTKQEEV 191

Sceloporus_chrysostictus_AMEL_Pleur NQPMFPVQQLPPMVPDTPLESWPAADKTKQEEV 191

Urosaurus_nigricaudus_Pleu NQPMFPVQQLPPMVPDTPLESWPAADKTKQEEV 191

Sceloporus_occidentalis_Pleu NQPMFPVQQLPPMVPDTPLESWPAADKTKQEEV 191

Sceloporus_tristichus_Pleu NQPMFPVQQLPPMVPDTPLESWPAADKTKQEEV 191

Sceloporus_undulatus_Pleu NQPMFPVQQLPPMVPDTPLESWPAADKTKQEEV 191

Gambelia_wislizenii_Pleu NSPMYPIHPVPPLIPDTPLEPWPPADKTKQEEV 190

Laemanctus_serratus_AMEL_Pleur NPPMYPVHPLPPLVPDTPLESWPPADKTKQEEV 193

Iguana_delicatissima_Pleu NPPMYPVQPLAPVLPDTPLESWPAADKTKQEEV 186

Cyclura_pinguis_Pleu NPPMYPVQPFAPVIPDTPLESWPAPDKTKQEEV 187

* *:..: ..*::.* ***.** *******:

**AMTN-AA-MAFFT-Iguania+Mammal – DIVERGE-NONE + PCOC ≥ 0.9**

CLUSTAL W (1.8) multiple sequence alignment (ALTER 1.3.3)

Acanthocercus_cyanogaster_AMTN_Acr MKIVILLFSSLGLTLSIPYNQFGRHLATSNSREILRLMQKYRAQGNIPQQTQQRPNPGVG 60

Acanthocercus_minutus_AMTN_Acr MKIVILLFSSLGLTLSLPYNQFGRHLATSNSREILRLMQKYRAQGNIPQQTQQRLNPGVG 60

Xenagama_zonura_AMTN_Acr MKIVILLFSSLGLTLSLPYNQFGRHLATSNSREILRLMQKYRAQGNIPQQTQQRPNPGVG 60

Laudakia_wui_Acr MKIVILLLSSLGLTFSLPYNQFGRHFATSNSREILRLMQKYRAQGNIPQQTQQRPNPGIG 60

Phrynocephalus_forsythii_Acr MKIVILLFSSLGLTFSLPYNQFGRHFATSNSREILRLMQKYRAQGNVPQQTQQRPNPGVG 60

Phrynocephalus_guinanensis_Acr MKIVILLFSSLGLTFSLPYNQFGRHFATSNSKEILRLMQKYRAQGNVPQQTQQRPNPGVG 60

Phrynocephalus_putjatai_Acr MKIVILLFSSLGLTFSLPYNQFGRHFATSNSREILRLMQKYRAQGNVPQQTQQRPNPGVG 60

Phrynocephalus_versicolor_Acr MKIVILLFSSLGLTFSLPYNQFGRHFATSNSREILRLMQKYRAQGNVPQQTQQRPNPGVG 60

Phrynocephalus_vlangalii_Acr MKIVILLFSSLGLTFSLPYNQFGRHFATSNSREILRLMQKYRAQGNVPQQTQQRPNPGVG 60

Agama_doriae_AMTN_Acr MKIVILLFSSLGLTFSLPYNQLRRHLATSNSREILRLMQKYRAQGNIPQQTQQRPNPGVG 60

Intellagama_lesueurii_Acr MKIVILLLFSVGLTFSLPYNQFGRLLATSNSREILRLMQKYRAQGNIPQQTQQRTNPGTG 60

Pogona_vitticeps_Acr MKIVILLLFSVGLTFSLPYNQFGRLLATSNSREILRLMQKYRAQGNNPQQTQQRPNPGTG 60

Bradypodion_pumilum_Acr MKIVILLLYSLGLTFALP---FNRLLATSNSREILRLMQKYKTQGNVPQKAQQRPNLVTR 57

Bradypodion_ventrale_Acr MKIVILLLYSLGLTFALP---FNRLLATSNSREILRLMQKYKTQGNVPQKAQQGPNLVTR 57

Furcifer_pardalis_Acr MKIVILLLYSLGLTFALP---FDRLLATSNSREILRLMQKYKTQGNVPQKAQQRPNLVTR 57

Chamaeleo_calyptratus_AMTN_Acr MKIVILLLYSLGLTFALP---FDRLLATSNSREILRLMQKYKTQGNVPQKAQQRPNLVTR 57

Chamaeleo_dilepis_AMTN_Acr MKIVILLLYSLGLTFALP---FDRLLATSNSREILRLMQKYKNQGNVPQKAQQRPNLVTR 57

Chamaeleo_gracilis_AMTN_Acr MKIVILLLYSLGLTFALP---FDRLLATSNSREILRLMQKYKTQGNVPQKAQQRPNLVTR 57

Chamaeleo_laevigatus_AMTN_Acr MKIVILLLYSLGLTFALP---FDRLLATSNSREXXXXXXXXXXXXXXXXXXXQRPNLVTR 57

Trioceros_affinis_AMTN_Acr MKVVILLLYSLGLTFALP---FDRLLATSNSREILRLMQKYKTQGNVPQKAQQRPNLVTR 57

Trioceros_balebicornutus_AMTN_Acr MKIVILLLYSLGLTFALP---XXXXXXXXXXXXILRLMQKYKTQGNVPQKAQQRPNLVTR 57

Trioceros_harennae_AMTN_Acr MKIVILLLYSLGLTFALP---FDRLLATSNSREILRLMQKYKTQGNVPQKAQQRPNLVTR 57

Anolis_apletophallus_Pleu MKIVILLLSLLGLTFCLPVNQFGRHFATSNSREILRLMQRYKAQGNVPQQTQQRSNPGIG 60

Anolis_sagrei_ordinatus_Pleu MKIVILLLSLLGLTFCLPVNQFGRRFATSNSREILRLMQRYRAQGNIPQQTQPRSNPGIG 60

Anolis_tropidonotus_AMTN_Acr MKIVILLLSLLGLTFCLPVNQFGRRFATSNSREILRLMQRYKAQGNVPQQTQQRSNPGIG 60

Anolis_carolinensis_Pleu MKIVILLLSLLGLTFCLPVNQFGRRFATSNSREILRLMQRYKAQGNVPQQTQQRSNPGIG 60

Anolis_rodriguezii_AMTN_Pleur MKIVILLLSLLGMTFCLPVNQFGRGFATSNSREILRLMQRYKAQGNVPQQTQQRSNPGIG 60

Ctenosaura_bakeri_Pleu MKIVILLLSLLGLTFCLPVNQFGRLLATSNSRERLRLMQRYKAQGNIPQQTQQRPNSGVG 60

Iguana_delicatissima_Pleu MKIVILLLSFLGLTSCLPVNQFGRLLATSNSRERLRLMQRYKAQGNIPQQTQQRPNSGVG 60

Cyclura_pinguis_Acr MKIVILLLSLLGVTFCLPVNQFGRLLATSNSRERLRLMQRYKAQGNIPQQTQQRPNSGVG 60

Gambelia_wislizenii_Acr MKIVILLLSLLGLTFCLPVNQFGRHFATSNSREILRLMQRYKAQENSPQQTQQRPNSGVG 60

Laemanctus_serratus_AMTN_Acr MKIVILLLSLLGLTFCLPVNQFGRRLATSNSREILRLMQRYKARGNIPQQTQQRPNSGVG 60

Phrynosoma_blainvillii_Pleu MNIVILLLSLMGLTFCLPVNQFGRLLATSNSREILQLMQRYKAQGNIPQQTQQRPNPGVG 60

Phrynosoma_platyrhinos_Pleu MNIVILLLSFLGMTFCLPVNQFGKLLATSNSREILQLMQRYKAQGNIPQQTQQRPNPGVG 60

Sceloparus_undulatus_Pleu MKIVILLLSLLGLTFCLPVNQFGRRLATSNSREILRL-QRYKAQGNIPQQTQQRPHPGVG 59

Sceloporus_tristichus_Pleu MKIVILLPSLLGLTFCLPVNQFGRRLATSNSREILRL-QRYKAQGNIPQQTQQRPHPGVG 59

Sceloporus_occidentalis_Pleu MKIVILLLSLLGLTFCLPVNQLGRRLATSNSREILRFMQRYKAQGNIQQQTQQRPHPGVG 60

Sceloporus_chrysostictus_AMTN_Pleur MKIVILLLSLLGLTFCLPVNQFGRRFATSNSREILRLMQKYKAQGNIPQQTQQRPNPGAG 60

Urosaurus_nigricaudus_Pleu MKIVILLLSLLGLTFGLPVKIFGRLLGTSNSREILQFMQRYKDQATIPQQPQQRPNPGVG 60

Canis_lupus_dingo_AMTN MKKD-LFIRGLREPAGIL----------------------------------AQLNPALG 25

Vulpes_vulpes_AMTN MKTMILLLYLLGSTQSLP----------------------------------AQLNPALG 26

Meles_meles_AMTN MKSMILLLYLLGSTQSLP----------------------------------TQLNPVLG 26

Mustela_putorius_AMTN MKTMILLLYLLGSTQSLP----------------------------------TQLNPVLG 26

Ursus_arctos_AMTN MKTLILLLYLLGSTRSLP----------------------------------TQLNPALG 26

Phacochoerus_africanus_AMTN MKTTILLFCLLGSTLSLP----------------------------------MQLNPVLG 26

Homo_sapiens_AMTN MRSTILLFCLLGSTRSLP-----------------------------------QLKPALG 25

Saimiri_boliviensis_AMTN MKTTILLFCLLGSTQSLP-----------------------------------QLKPALG 25

Phyllostomus_hastatus_AMTN MKTMILLFCLLGSTQSLP----------------------------------MQLTPALG 26

Erinaceus_europaeus_AMTN MKTTFLLFCLLGSTHTLP----------------------------------MQLNPALG 26

Suncus_etruscus_AMTN MKNVILLFCLLGPVKSL-----------------------------------IQINPALG 25

Echinops_telfairi_AMTN MKTIILLFCLLGSTQSLP------------------------------------------ 18

*. *: : :

Acanthocercus_cyanogaster_AMTN_Acr LPPAKLVPDQPPLANQAPNE-VIPFVWPNLPALPVLPVQTPLE------QNVAGFNVLQL 113

Acanthocercus_minutus_AMTN_Acr LPPAKLVPDQPPLANQAPNE-VIPFVWPNLPALPVLPVQTPLE------QNVAGFNVLQL 113

Xenagama_zonura_AMTN_Acr LPPPKLVPDQPPLANQAPNE-VIPFVWPNLPALPVLPVQTPLE------QNVAGFNVLQL 113

Laudakia_wui_Acr LPPAKLVPDQPPLANQVPNEQVISFGWPNLPGLPVLPAQTPLE------QTVAGFNVLQL 114

Phrynocephalus_forsythii_Acr LPPAKLVPDQPPLASQVPNE-FIPFGWPNLPGLPVLPAQTQLE------QNAAGFSVLQL 113

Phrynocephalus_guinanensis_Acr LPPAKLVPDQPPLASQVPNE-FIPFGWPNLPGLPVLPAQTPLE------QNAAGFNVLQL 113

Phrynocephalus_putjatai_Acr LPPAKLVPDQPPLASQVPNE-FIPFGWPNLPGLPVLPAQTPLE------QNAAGFNVLQL 113

Phrynocephalus_versicolor_Acr LPPAKLVPDQPPLASQVPNE-FIPFGWPNLPGLPVLPAQTPLE------QNAAGFNVLQL 113

Phrynocephalus_vlangalii_Acr LPPAKLVPDQPPLASQVPNE-FIPFGWPYLPGLPVLPAQTPLE------QNAAGFNVLQL 113

Agama_doriae_AMTN_Acr LPPLKLVPDQPPLANQVPNE-IIPFGWPNFQAFPVLPAQTPLE------QNVAGFNVLQL 113

Intellagama_lesueurii_Acr LPPAKLIPDQHPLANQVPNE-VVPFAWPNLPNFPVLPAQTPLE------QNVAGYNVLPL 113

Pogona_vitticeps_Acr LPPAKLIPDQHPLANQVPNE-VVPFAWPNFPNFPVLPAQTPLE------QNVAGYNVLPL 113

Bradypodion_pumilum_Acr LPPAKLVPDRHPLSNQVPHELVFPFEW---ANLPVLPPHIPLEQGSPNLQNFAGFNVLPM 114

Bradypodion_ventrale_Acr LPPAKLVPDRRPLSNQVPHELVFPFEW---ANLPVLPPHIPLEQGSPNLQNFAGFNVLPM 114

Furcifer_pardalis_Acr LPPAKLVPDQHPLSNQVPNELVFPFEW---ANLPVLPPHIPLEQGSPNLQNFPGFNVLPM 114

Chamaeleo_calyptratus_AMTN_Acr LPPAKLVPDQHPLSNQVPNELVFPFEW---ANLPVLPPHIPLEQGSPNLQNFAGFNVLPM 114

Chamaeleo_dilepis_AMTN_Acr LPPAKLVPDQHPLSNQVPNEMVVPFEW---ANLPVLPPHIPLEQGSPNLQNFAGFNVLPM 114

Chamaeleo_gracilis_AMTN_Acr LPPAKLVPDQHPLSNQVPNELAVPFEW---ANLPVLPPHIPLEQGSPNLQNFAGFNVLPM 114

Chamaeleo_laevigatus_AMTN_Acr LPPAKLVPDQHPLSNQVPNELVFPFEW---ANLPVLPPHIPLEQGSPNLQNFAGFNVLPM 114

Trioceros_affinis_AMTN_Acr LPPSKLVPDWQPLSNQVANEXXXXXXX---XXXXXXXXXXXXXXXXXXXXNFAGFNVLPM 114

Trioceros_balebicornutus_AMTN_Acr LPPSKLVPDWQPLSNQVANELVFPFEW---ANLPVLPPHIPLEQGSPNLQNFAGFNVLPM 114

Trioceros_harennae_AMTN_Acr LPPSKLVPGWQPLSNQVANELVFPFEW---ANLPVLPPHIPLEQGSPNLQNFAGFNVLPM 114

Anolis_apletophallus_Pleu LPPAKLVPDQHPLANHIPNE-IPAIEWP-FANYPFLTARNPQELGSPTIPNLPGLNVLPL 118

Anolis_sagrei_ordinatus_Pleu LPPAKLVPDQHPLANHIPNE-IPAIEWP-FGNYPILTAQNPQELGSPTVQNFPGFNVLPF 118

Anolis_tropidonotus_AMTN_Acr LPPAKLVPDQHPLANHIPNE-IPAIEWP-FANYPILTAQNPQELGSPTIQNFPGFNVLPL 118

Anolis_carolinensis_Pleu LPPAKLVPDQHPLANHIPSE-IPAFERP-FANYPILTAQNPQELGSPTLQNFPGFNVLPL 118

Anolis_rodriguezii_AMTN_Pleur LPPAKLVPDQHPLANHIPNE-IPAIAWP-FANYPFLTAQNPQELGTPIIQNFPGFNVLPF 118

Ctenosaura_bakeri_Pleu LPPAKLVPDQQPFANQIPNE-IPAFEWQ-FANFPVFSPQNPLELGSPNVQNFPGLSVLPL 118

Iguana_delicatissima_Pleu LPPAKLVPDQQPFTNQIPNE-IPAFEWQ-FANFPVFSPQNPLELGSPNVQNFPGLSVLPL 118

Cyclura_pinguis_Acr LPPAKLVPDQQPFANQIPNE-IPAFEWQ-FANFPVFSPQNPLELGSPNVQNFPGLNVLPL 118

Gambelia_wislizenii_Acr LPPAKLVPDQQPLANQIPNE-MLPFEWL-FPNFPIFSPQNALELGNPNVQNFPGSSVLPL 118

Laemanctus_serratus_AMTN_Acr LPPAKLVPDLQ----QIPNE-ILPFEWP-VANFPIFSPQNTLELGSPNVQNSPGFNVQPS 114

Phrynosoma_blainvillii_Pleu LPPAKLVPDQQPLGNQIPNE-ILPFELP-FANLPIFFPQNRLELGSPNVQNVPGFSVLPL 118

Phrynosoma_platyrhinos_Pleu LPPAKLVPDQQPLGNQIPNE-ILPFELP-FANFPIFFPQNRLELGSPNVQNVPGFNALPL 118

Sceloparus_undulatus_Pleu LPPAKLVPDQQLLANQIPNE-ILPFEWP-FANFPVFAPQNRLELGSPKAQNAPGFNVLPL 117

Sceloporus_tristichus_Pleu LPPAKLVPDQQLLANQIPNE-ILPFEWP-FANFPVFAPQNRLELGSPNAQNAPGFNVLPL 117

Sceloporus_occidentalis_Pleu LPPAKLVPDQQLLANQIPNE-ILPFEFP-FANFPVFAPQNRLELGSPNAQNAPGFNVLPL 118

Sceloporus_chrysostictus_AMTN_Pleur LPPAKLVPDQQILANQIPSE-ILPFELP-FTNFPVFAPQSRLELGNPNVQNAPGFNVLPL 118

Urosaurus_nigricaudus_Pleu LPPAKLVPDQQLLANQIPSE-ILPFESP-FANFPVLAPQNRLELGSPNVQNIPPFNVLPL 118

Canis_lupus_dingo_AMTN LPPTKLIPHQATLLNQQQPN-----------------------------QVFPSLSLIPL 56

Vulpes_vulpes_AMTN LPPTKLTPHQATLLNQQQPN-----------------------------QVFPSLSLIPL 57

Meles_meles_AMTN LPPTKPAPHQATLLNQQQPN-----------------------------QVFPSLSLIPL 57

Mustela_putorius_AMTN LPPTKPAPHQATLLNQQQPN-----------------------------QVLPSLSLIPL 57

Ursus_arctos_AMTN LPPTKPAPHQATLLNQQQPN-----------------------------QVFPSLSLIPL 57

Phacochoerus_africanus_AMTN LPPTKLVPDQATLRNQQQQN-----------------------------QVFPSLSLIPL 57

Homo_sapiens_AMTN LPPTKLAPDQGTLPNQQQSN-----------------------------QVFPSLSLIPL 56

Saimiri_boliviensis_AMTN LPPTKLAPDQATLLNQQQSN-----------------------------QVFPSLSLIPL 56

Phyllostomus_hastatus_AMTN LPPAQLVPDQTTLLNQQHPN-----------------------------QVFHSLSLIPL 57

Erinaceus_europaeus_AMTN LPPSKLVPDQATLLSQPQPN-----------------------------QVFPSLNLIPL 57

Suncus_etruscus_AMTN LPPISLVPDQATL-NQQQSN-----------------------------QVFSSVSLIPL 55

Echinops_telfairi_AMTN --------------------------------------------------VFPSLSLIPL 28

.

Acanthocercus_cyanogaster_AMTN_Acr PQMFPL--DVNYLVALLSALSGTETLPLAGGGLTV-----P--APP---MLPIIFTQMGP 161

Acanthocercus_minutus_AMTN_Acr PQMFPL--DVNYLVALLSALSGTETLPLAGGGLTV-----P--APP---MLPIIFTQMGP 161

Xenagama_zonura_AMTN_Acr PQMFPL--DVNYLVALLSALSGTETLPLAGGGLTV-----P--APP---MLPIIFTQMGP 161

Laudakia_wui_Acr PQMFPL--DVNYLVALLSALSGTETLPLAGGGLTV-----P--APP---MLPIIFTQMGP 162

Phrynocephalus_forsythii_Acr PQMFPL--DVNYVVALLSALSGTETLPLAGGGLTV-----P--ASP---MLPIIFTQMGP 161

Phrynocephalus_guinanensis_Acr PQMFPL--DVNYLVALLSALSGTETLPLAGGGLTV-----P--ASP---MLPIIFTQMGP 161

Phrynocephalus_putjatai_Acr PQMFPL--DVNYLVALLSALSGTETLPLAGGGLTV-----P--ASP---MLPIIFTQMGP 161

Phrynocephalus_versicolor_Acr PQMFPL--DVNYLVALLSALSGTETLPLAGGGLTV-----P--ASP---MLPIIFTQMGP 161

Phrynocephalus_vlangalii_Acr PQMFPL--DVNYLVALLSALSGTETLPLAGGGLTV-----P--ASP---MLPIIFTQMGP 161

Agama_doriae_AMTN_Acr PQMFPL--DVNYLVALLSALSGTETLPLAGGGLTV-----P--APPGFFYLPVLCTEM-- 162

Intellagama_lesueurii_Acr PQMFPL--DVNSLVALLAALSGTETLPLPGGGLTV-----P--APP---MLPVLFTQMGP 161

Pogona_vitticeps_Acr PQMFPL--DINSLVALLAALSGTETLPLPGGGLTV-----P--APP---MLPVLFTQMGP 161

Bradypodion_pumilum_Acr TQMFPL--DINYLVALLAALSGTQTLPLAAGGLTV-----P--APP---MLPIILTQMAP 162

Bradypodion_ventrale_Acr TQMLPL--DINYLVALLAALSGTQTLPLAAGGLTV-----P--APP---MLPIILTQMAP 162

Furcifer_pardalis_Acr TQMFPL--DINYLVALLTALSGTQTFPLAAGGLTV-----P--APP---MLPIILTQMGP 162

Chamaeleo_calyptratus_AMTN_Acr TQMFPL--DINYLVALLTALSGTQTLPLVAGGLTV-----P--APP---MLPIILTQMGP 162

Chamaeleo_dilepis_AMTN_Acr TQMFPL--DINYLVALLTALSGTQTLPLVAGGLTV-----P--APP---MLPIILTQMGP 162

Chamaeleo_gracilis_AMTN_Acr TQMFPL--DINYLVALLTALSGTQTLPLVAGGLTV-----P--APP---MLPIILTQMGP 162

Chamaeleo_laevigatus_AMTN_Acr TQMFPL--DINYLVALLTALSGTQTLPLVAGGLTV-----P--APP---MLPIILTQMGP 162

Trioceros_affinis_AMTN_Acr TQMFPL--DINYLVALLTALSGTQTLPLAAGGLTV-----P--SPP---MLPIILTQMGP 162

Trioceros_balebicornutus_AMTN_Acr TQMFPL--DINYLVALLTALSGTQTLPLAAGGLTV-----P--APP---MLPIILTQMGP 162

Trioceros_harennae_AMTN_Acr TQMFPL--DINYLVALLTALSGTQTLPLAAGGLTV-----P--APP---MLPIILTQMGP 162

Anolis_apletophallus_Pleu TQMVPI--DINYLALLLGALSQTQTQPLPGGPL-IPQQLLP--PQS---MLPIIFTQMGP 170

Anolis_sagrei_ordinatus_Pleu TQMVPI--DINSLALLLGALSTTQAQPLPGGGL-IPQQLLP--PPP---MFPIIFTQMGP 170

Anolis_tropidonotus_AMTN_Acr TQMVPI--DINYLALLLGALSQTQTQPLPGGGL-IPQQLLP--PQS---MLPIIFTQMGP 170

Anolis_carolinensis_Pleu TQMVPI--DINYLALLLGALSTTQTQPLPGGGLNIPQQLLPP-PQS---MFPIIFTQMGP 172

Anolis_rodriguezii_AMTN_Pleur TQMVPI--DINTLALLLGALSTTQTQPLPGGGL-IPQQLLPPLPQS---XXXXXXXXXXX 172

Ctenosaura_bakeri_Pleu NQMVPI--DINSLAVLLGAIPPSQTQPLPGGGINIPQQLLP--AQA---MLPIIFTQMGP 171

Iguana_delicatissima_Pleu NQMVPI--DINSLAVLLGAIPPSQTQPLPGGGINIPQQLLP--AQS---MLPIIFTQMGP 171

Cyclura_pinguis_Acr NQMVPI--DINSLAVLLGAISPSQTQPLPGGGINIPQQLLP--AQS---MLPIIFTQMGP 171

Gambelia_wislizenii_Acr TQMVPI--DINSLAELLRVLSATQTQPLPGGGITGPQQLLP--AQS---MLPIIFTQMGP 171

Laemanctus_serratus_AMTN_Acr NLMVPI--DINSLAMLLAALSTTQTS--MGGGITVPEQLLP--PQP---MLPIIFTQMGP 165

Phrynosoma_blainvillii_Pleu TQMAPI--DINSLAVLLGALSTAQTQP----------PLLS--PL----MFPVVFTQRGP 160

Phrynosoma_platyrhinos_Pleu TQMAPI--DINSLAVLLGALSTAQTQP----------PLLS--PL----MFPVVFTQMGP 160

Sceloparus_undulatus_Pleu TQMVPM--DINALAVLLGALSTAQIQQ----------QLLP--PQP---MLPIIFTQMGP 160

Sceloporus_tristichus_Pleu TQMVPM--DINALAVLLGALSTAQIQQ----------QLLP--PQP---MLPIIFTQMGP 160

Sceloporus_occidentalis_Pleu TQMVPM--DINALAVLLGALSTAQIQQ----------QLLS--PQP---MLPIIFTQMGP 161

Sceloporus_chrysostictus_AMTN_Pleur TQMVPM--DINALAVLLGALSAAQTQP----------QLLP--PQP---MLPIIFTQMGP 161

Urosaurus_nigricaudus_Pleu TQMVPM--DISALAVLLGALSTAQTQP----------QLLP--PQA---MLPIIFTQMGP 161

Canis_lupus_dingo_AMTN TQMFTLASDLQLLNPAAGMASGTQTLPLSLGVLNTQQQL----QPQ---MLPVIVAHLGA 109

Vulpes_vulpes_AMTN TQMFTLASDLQLLNPAAGMASGTQTLPLSLGVLNTQQQL----QPQ---MLPVIVAHLGA 110

Meles_meles_AMTN TQMFTLGSDLQLLNPAAGMPPGTQTLSLSLGGLNTQQQL----QSQ---MLPVIVAHLGA 110

Mustela_putorius_AMTN TQLFTLGSDLQLLNPAAGMPPGPQTLPLSLGVLNTQQQL----QSQ---MLPVIVAHLGA 110

Ursus_arctos_AMTN TQMFTLGSDLQLLNPAAGMAPGTQTLPLSLGGLNAQQQL----QPQ---MLPVIVAHLGT 110

Phacochoerus_africanus_AMTN THLLTLGSDLQLLNPAIGMVPGSQTLPLTLGALNVQQQL----QPQ---MIPVIVAHLGA 110

Homo_sapiens_AMTN TQMLTLGPDLHLLNPAAGMTPGTQTHPLTLGGLNVQQQL----HPH---VLPIFVTQLGA 109

Saimiri_boliviensis_AMTN TQVLTLGQDLQLLNPAAGMTPGAQTHPLTLGGLNVQQQL----QPH---MLPIFVTHLGA 109

Phyllostomus_hastatus_AMTN TQMLTLGSDLQLLNPPAGMAPAAQTLPLTLGALNMQQQL----QPQ---MLPLIVAHLGA 110

Erinaceus_europaeus_AMTN TQMLTLGSDLQLLNPTAGMTPGPQTLPLTLGGLNVQQQL----QPQ---MLPIIVAQLGA 110

Suncus_etruscus_AMTN TQMLALGSDLQLLNSPAGITPATQTLPLSLGKLNGQQPL----QPH---MLPVIVAQLGT 108

Echinops_telfairi_AMTN TQMLALGSDLQMLNTATGVGPGTQTLPVNLGGVTVQQQL----QPQ---MLPIFVAQLGT 81

: .: *: : . .:

Acanthocercus_cyanogaster_AMTN_Acr QGQVLSSEEMPPS-QVFAGVL---LPGTQG---GLLPAGQSGSLPEGQEGLLPAGQTGTN 214

Acanthocercus_minutus_AMTN_Acr QGQVLSSEEMPPS-QVFAGVL---LPGTQG---GLLPAGQSGSLPEGQEGLLPAGQTGTN 214

Xenagama_zonura_AMTN_Acr QGQVLSSEEMPPS-QVFAGVL---LPGTQG---GLLPAGQSGSLPEGQEGLLPAGQTGTN 214

Laudakia_wui_Acr QGQVLSSEEMPPS-QVFAGVL---LPGTQG---GLLPAGQSGSLPEGQEGLLPAGQTGIN 215

Phrynocephalus_forsythii_Acr QGQVLSSEEMPPS-QVFAGVL---LPGTQG---GLLPAGQSGSFPEGQEGLLPAGQTGTN 214

Phrynocephalus_guinanensis_Acr QGQVLSSEEMPPS-QVFAGVL---LPGTQG---GLLPTGQSGSFPEGQEGLLPAVQTGTN 214

Phrynocephalus_putjatai_Acr QGQVLSSEEMPPS-QVFAGVL---LPGTQG---GLLPTGQSGSFPEGQEGLLPAVQTGTN 214

Phrynocephalus_versicolor_Acr QGQVLSSEEMPPS-QVFAGVL---LPGTQG---GLLPAGQSGSFPEGQEGLLPAGQTGTN 214

Phrynocephalus_vlangalii_Acr QGQVLSSEEMPPS-QVFAGVL---LPGTQG---GLLPAGQSGSFPEGQEGLLPAGQTGTN 214

Agama_doriae_AMTN_Acr -GQVLSSEEMPPS-QVFAGVL---LPGTQG---GLLPAGQSGSLPEGQEGLLPAGQTGTS 214

Intellagama_lesueurii_Acr QGQVLSSEEMPPS-QLFAGVL---LPGTQG---GLLPAGQAGTHSEGQEGFLPAGQTGTN 214

Pogona_vitticeps_Acr QGQVLSSEEMPPS-QLFAGVL---LPGTQG---GLLPAGQAGTHSEGQEGFLPAGQTGTN 214

Bradypodion_pumilum_Acr QGQMLSSEEMSPS-QVFAGVL---LPGTQDEMKQLLPSGQTGTFPEGQEGLLPAGQTGAN 218

Bradypodion_ventrale_Acr QGQMLSSEEMSPS-QVFAGVL---FPGTQDEMKQLLPSGQTXTLPEGQEGLLPAGQTGAN 218

Furcifer_pardalis_Acr QGQMLSSEEMTPS-QVFAGVL---LPGTQDEIKQLLPSGQTGTFPEGQEGLLPAGQTGAN 218

Chamaeleo_calyptratus_AMTN_Acr QGQMLSSEEMSPS-QVFAGVL---LPGTQDEIKQLLPSGQTGTFSEGQEVLLPAGQTGAN 218

Chamaeleo_dilepis_AMTN_Acr QGQMLSSEEMSPS-QVFAGVL---LPGTQDEIKQLLPSGQTGTLSGGQEVLLPAGQTGAN 218

Chamaeleo_gracilis_AMTN_Acr QGQMLSSEEMSPS-QVFAGVL---LPGTQDEIKQLLPSGQTGTLSGGQEVLLPAGQAGAN 218

Chamaeleo_laevigatus_AMTN_Acr QGQMLSSEEMSPS-QVFAGVL---LPGTQDEIKQLLPSGQTGILSEGQEVLLPAGQTGAN 218

Trioceros_affinis_AMTN_Acr QXXXXXXXXXSPS-QVFAGVI---LPGTQDEIKQLLPSGQTGTLPEGQEGLLPAGQTGAN 218

Trioceros_balebicornutus_AMTN_Acr QXXXXXXXXXSPP-QVFAGVL---LPGTQDEIKQLLPSGQTGTLPEGQEGLLPAGQTGAN 218

Trioceros_harennae_AMTN_Acr QXXXXXXXXXSPP-QVFAGVL---LPGTQDEIKQLLPSGQTGTLPEGQEGLLPAGQTGAN 218

Anolis_apletophallus_Pleu QGAVLSSEEMPTS-PVMAFLL-PGLPGLPG---GLFPSGQSGALPEDQEDLLPAGQVRNN 225

Anolis_sagrei_ordinatus_Pleu QGTVLSSEEMPTS-QVMAAFL---LPGLPG---GLFPSGQSGALPEDQDDLLPAGQVGTN 223

Anolis_tropidonotus_AMTN_Acr QGTVLSSEEMSTS-QVMAAFL---LPGLPG---GLFPSGQSGALPEDQEGLLPAGQVGNN 223

Anolis_carolinensis_Pleu QGTVLSSEEMPTS-QVMAAFL---LPGLPG---GLFPSGQSGALPEDQDELLPAGQVGTN 225

Anolis_rodriguezii_AMTN_Pleur XGAVLSSEEMPSS-QVMAAFLLPGLPGLPG---GLFPSGQSGALPEEQEDLLPAGQVGNN 228

Ctenosaura_bakeri_Pleu QGAVLSSEEMPTS-QVFAAFL---IPGTQG---GLLPSGQAGTVSEGQEGLLPAGQAGNS 224

Iguana_delicatissima_Pleu QGTVLSSEEMPTS-QVFAAFL---IPGTQG---GLLPSGQAGTVSEGQEGLLPAGQAGNS 224

Cyclura_pinguis_Acr QGAVLSSEEMPTS-QVFAAFL---IPGTQG---GLLPSGQAGTVAEGQEGLLPAGQAGNG 224

Gambelia_wislizenii_Acr QGTVLSSEEMPVS-QVFAGFL---VPGMQG---GLFPSGQAGTIPEGQEEPLPAGQAGTN 224

Laemanctus_serratus_AMTN_Acr QGTVLSSEEMPTS-QMFAAFL---IPGTQG---GLFPSGQA--LPEGQEGLLPASQAGTN 216

Phrynosoma_blainvillii_Pleu QGTVLSSEEMPTS-QIFAGFL---VPGTQG---SLFPSGQAGTLPEGQEGLFPASQAGTN 213

Phrynosoma_platyrhinos_Pleu QGTVLSSEEMPTS-QIFAGFL---VPGTQG---GLFPSGQARTLPEGQEGLFPASQAGTN 213

Sceloparus_undulatus_Pleu QGTVLSSEEMPTS-QVFAGFL---VPGTQG---GLFPPGQA-SLPEGQEGLFPASQAGTN 212

Sceloporus_tristichus_Pleu QGTVLSSEEMPTS-QVFAGFL---VPGTQG---GLFPPGQA-SLPEGQEGLFPASQAGTN 212

Sceloporus_occidentalis_Pleu QGTVLSSEEMPTS-QVFAGFL---VPGTQG---GLFPPGQA-SLPEGQEGLFPASQAGTN 213

Sceloporus_chrysostictus_AMTN_Pleur QGTVLSSEEMPPS-QVFAGFL---VPGAQP---GLFPSGQAGTLPEGQEGLFPASQAGTN 214

Urosaurus_nigricaudus_Pleu QGTVLSSEEMPTS-QVFAGFL---VPGTQG---GLLPSGQAGSLPEGQEGLFPASQAGTN 214

Canis_lupus_dingo_AMTN HGAILSSEELPGSPQIFPGLIF--QPLFPG---AILPTSP--ANPDAQNGILPAGQAGGN 162

Vulpes_vulpes_AMTN HGAILSSEELPGSPQIFTGLIF--QPLFPG---AILPTSP--ANPDAQNGILPAGQAGGN 163

Meles_meles_AMTN HGAIISSEELPVAPQIFTGLLF--QPLFPG---SILPTSP--ANPDAQNGILPAGQARVN 163

Mustela_putorius_AMTN HGAIISSEELPVAPKIFTGLLF--QPLFPG---SILPTSP--ANPDAQNGILPAGQAGVN 163

Ursus_arctos_AMTN HGAILSSEELPVAPQIFTGLLF--QPLFPG---AILPTSP--ANPDAQNGILPAGQAGAS 163

Phacochoerus_africanus_AMTN QGAILSSEELPATRQFLTGLIF--HTLFPG---AILPPSP--ANPDAQNGIHPAGQAGAN 163

Homo_sapiens_AMTN QGTILSSEELP---QIFTSLII--HSLFPG---GILPTSQAGANPDVQDGSLPAGGAGVN 161

Saimiri_boliviensis_AMTN QGTILSSEELPLAPQIFTGLII--HSLFPG---SILPTSQAGANPNVQDGRLPAGQAGVN 164

Phyllostomus_hastatus_AMTN QGTILSSEELPGAPHIFTGLIF--QPLLPG---AILPSSQ--ANPDVQNRILPAGQAGVN 163

Erinaceus_europaeus_AMTN QGTILSSEELPVASQIL-GLLF--QPLLPG---SILSTSK--ANPDTQNGILPSGQVGAN 162

Suncus_etruscus_AMTN QGTILSSEELPVASQLFTGLIL--QPLVQG---AILSKSQ--INPDIQNGVIPAGQSKPN 161

Echinops_telfairi_AMTN QGAILSSEELPVAPQVYTGLLI--HPWFPG---AFLPTSQAGSTPEGQDGALPAGQAAAH 136

. . .: . ::. . . *: *:

Acanthocercus_cyanogaster_AMTN_Acr -------QGSLPLPEG----------TAAGIQKASPATSYIPNEPLGGPYPTPS------ 251

Acanthocercus_minutus_AMTN_Acr -------QGSLPLPEG----------TAAGIQKASPATSYIPNEPLGGPYPTPS------ 251

Xenagama_zonura_AMTN_Acr -------QGSLPLPEG----------TAAGIQKASPATSYIPNEPLGGPYPTPS------ 251

Laudakia_wui_Acr -------QGGLPLPEG----------TAAGIQKASPASSDSLNEPMGRPYPTPS------ 252

Phrynocephalus_forsythii_Acr -------QGGLPLPEG----------TAAGIQKASPATSDLLNDPMGGPYPTPS------ 251

Phrynocephalus_guinanensis_Acr -------QGGLPLPEG----------TAAGIQKASPATSDILNDPMGGPYPTLS------ 251

Phrynocephalus_putjatai_Acr -------QGGLPLPEG----------TAAGIQKASPATSDILNDPMGGPYPTPS------ 251

Phrynocephalus_versicolor_Acr -------QGGLPLPEG----------TAAGIQKASPATSDSLNDPMGGPYPTPS------ 251

Phrynocephalus_vlangalii_Acr -------QGGLPLPEG----------TAAGIQKASPATSDILNDPMGGPYPTPS------ 251

Agama_doriae_AMTN_Acr -------QGGLPLPED----------TAAGIQKASPATSNGLNEPLGGAYPTPS------ 251

Intellagama_lesueurii_Acr -------QGILPLPEG----------TAAGIQKASPATSDSLNEPVGGPYPTPS------ 251

Pogona_vitticeps_Acr -------QGNLPLPEG----------TAAGIQKASPATSDSLNEPVGGLYPTPS------ 251

Bradypodion_pumilum_Acr -------QGILPLPEG----------TAAGIQKASPATSDGLNESLGRPCPTPS------ 255

Bradypodion_ventrale_Acr -------QGILPLPEG----------TAAGIQKASPATSNGLNESVGRPCPTPS------ 255

Furcifer_pardalis_Acr -------HGILPLPEG----------TAAGIQKASPATNDGLNESMGRPCPTPS------ 255

Chamaeleo_calyptratus_AMTN_Acr -------QVILPLPEG----------TAAGIHKASPETSNGLNESVGRPCPTPS------ 255

Chamaeleo_dilepis_AMTN_Acr -------QVILPLPEG----------TAAGIHKASPATSSGLNESVGRPCPTPS------ 255

Chamaeleo_gracilis_AMTN_Acr -------QVILPLPEG----------TAAGIHKASPATSNGLHESVGRPCPTPS------ 255

Chamaeleo_laevigatus_AMTN_Acr -------QVILPLPEG----------TAAGIHKASPATSNGLNESVGRPCPTPS------ 255

Trioceros_affinis_AMTN_Acr -------HGILSLPEG----------TAAGIQKASPAASDGLNESVVRPCPTPS------ 255

Trioceros_balebicornutus_AMTN_Acr -------HGILPLPEG----------TAAGIQKASPAASDGLNESVVRPCPTPS------ 255

Trioceros_harennae_AMTN_Acr -------HGILPLPEG----------TAAGIQKASPAASDGLNESVVRPCPTPS------ 255

Anolis_apletophallus_Pleu -------QGNLPYPES----------TAAGIQKGSPTIEDGLSVAPGAFYPTPS------ 262

Anolis_sagrei_ordinatus_Pleu -------QGNLPYPES----------TAAGIQKGSPTTEEGLSVAPGAFYPTPS------ 260

Anolis_tropidonotus_AMTN_Acr -------QGNLPYPES----------TAAGIQKGSPTIEDGLSVAPGAFYPTPS------ 260

Anolis_carolinensis_Pleu -------QGNLPFPES----------TAAGIQKGSPATEDGLNVAPGAFYPTPS------ 262

Anolis_rodriguezii_AMTN_Pleur -------QGNLPYPES----------TAAGIQKGSPTIEDGLSVAPGAFYPTPS------ 265

Ctenosaura_bakeri_Pleu -------QGNLPYPES----------TAAGIQKGSPTSGDGLSEATSISNPTPS------ 261

Iguana_delicatissima_Pleu -------QSNLPYPES----------TAAGIQKGSPTSGDGLSEATSISNPTPS------ 261

Cyclura_pinguis_Acr -------QGNLPYPEG----------TAAGIQKGSPTSGDGLSEATSISNPTPS------ 261

Gambelia_wislizenii_Acr -------QGNLPYPES----------TAAGIQKGSPTTGDGLREATSISYPSPS------ 261

Laemanctus_serratus_AMTN_Acr -------QGNLPHPES----------TAAGIQKGSPTTGDGLNEATSASYLTPS------ 253

Phrynosoma_blainvillii_Pleu -------------PES----------TAAGIQKGSPTTGDGLSEATSGSYPTLS------ 244

Phrynosoma_platyrhinos_Pleu -------------PES----------TAAGIHKGSPTTGDGLSEATSGSYPTLS------ 244

Sceloparus_undulatus_Pleu -------------PEN----------TAAGIQKGSPTTGDGLNEATSGSYPTPS------ 243

Sceloporus_tristichus_Pleu -------------PEN----------TVAGIQKGSPTTGDGLNEATSGSYPTPS------ 243

Sceloporus_occidentalis_Pleu -------------PEN----------TAAGIQKGSPTTGDGLNEATSGSYPTPS------ 244

Sceloporus_chrysostictus_AMTN_Pleur -------------PES----------AAAGIQKGSPTTGDGLSEATSGSYPTPS------ 245

Urosaurus_nigricaudus_Pleu -------------PES----------TAAGIQKGFPTTGDGLNEATSGSYPTPS------ 245

Canis_lupus_dingo_AMTN PAIQGTPESFSTTPSD--TDDDFGVTAPAGIQRGTHTTQETTSGPPNGTQ---------- 210

Vulpes_vulpes_AMTN PAIQGTPESFSTTPSD--TDDDFGVTAPAGIQRGTQTTQETTSGPPNGKFSEPG------ 215

Meles_meles_AMTN PAIQGTPEGFSTTPSD--IDDDFGVTAPAGIQKGMHTTQETTTGSPNDPDSLRQ------ 215

Mustela_putorius_AMTN PAIQGTAEGFSTTPSD--IDDDFGVTAPAGIQKGMHTTQETTTGSPNGIQ---------- 211

Ursus_arctos_AMTN PAIQGTPEGFSPTPSD--TDDDFGATAPAGIQRGVHTTQETTAGPPNGKFSEPG------ 215

Phacochoerus_africanus_AMTN PAIQGTPRGPFPTSSG--TDDDFDVTTPAGLQRGTHATEETTTASPNGMQ---------- 211

Homo_sapiens_AMTN PATQGTPAGRLPTPSG--TDDDFAVTTPAGIQRSTHAIEEATTESANGIQ---------- 209

Saimiri_boliviensis_AMTN PAIQGTPAGHFPTPSS--TDDDFAMTTPAGIQRSTHAVEETTTESSNGIQ---------- 212

Phyllostomus_hastatus_AMTN PSIQGT-ESPFLTPGGTDTDDDFEVTTPAGIQRGTHIPEGTTTGSPNGVQ---------- 212

Erinaceus_europaeus_AMTN PAIQEDTEAPFSTPSG--IDDDFGMTTPAGIQRGLQPTEETTTGSPNGKCPGP------- 213

Suncus_etruscus_AMTN PDLQGTTEIPFSTPSG--IDDDFGVTTPAGIQRGFHLTEETTTESPDAVS----CIKVEN 215

Echinops_telfairi_AMTN PAIQGTPEGHLPTPSG--TDYLSGVTAPAGLQRDMHTTEEATTDGLGSEP----RQRIDD 190

. . : **:::

Acanthocercus_cyanogaster_AMTN_Acr ------------GFRQPS--AVNNPVFVEP-TGSINLEPSE--LREPPTTLVRPDTSDNQ 294

Acanthocercus_minutus_AMTN_Acr ------------GFRQPS--AVNNPVFVEP-TGSINMEPSE--LREPPTTLVRPDTSDNQ 294

Xenagama_zonura_AMTN_Acr ------------GFRQPS--AVNNPVFVEP-TGSINMEPSE--LREPPTTLVRPDTSDNQ 294

Laudakia_wui_Acr ------------GFRQPS--AVTNAVFVEP-TGSINMEPSE--LREPPTTLVRPDMSDNQ 295

Phrynocephalus_forsythii_Acr ------------GFRKPS--AVTNAVFVEP-TGSINMEPSE--LREPPTTLVRPDTSDNQ 294

Phrynocephalus_guinanensis_Acr ------------VFRKPS--AVTNAVFVEP-TGSINMEPSE--LREPPTTLVRPDTSDNQ 294

Phrynocephalus_putjatai_Acr ------------GFRKPS--AVTNAVFVEP-TGSINMEPSE--LREPPTTLVRPDTRDNQ 294

Phrynocephalus_versicolor_Acr ------------GFRKPS--AVTNAVFVEP-TGSINMEPSE--LREPPTTLVRPDTSDNQ 294

Phrynocephalus_vlangalii_Acr ------------GFRKPS--AVTNAVFVEP-TGSINMEPSE--LREPPTTLVRPDTSDNQ 294

Agama_doriae_AMTN_Acr ------------GFRQPS--AITKTVFVEP-TGSINMEPSE--LREPPTTLVRPDTSENQ 294

Intellagama_lesueurii_Acr ------------GFRQPS--AVTNTVFVEP-TGGINMEPSE--LREPPTTLVKLYKSDNQ 294

Pogona_vitticeps_Acr ------------GFRQPS--AVTNAVFVEP-TGGINMEPSE--LREPPTTLVRLNKSDNQ 294

Bradypodion_pumilum_Acr ------------GFRQPS--AATSAVFVEP-TGLINMEPSE--LREPPTTLVRLYKGDNP 298

Bradypodion_ventrale_Acr ------------GFRQPS--AATSAVFVEP-TGLINMEPSE--LREPPTTLVRLYKDDNP 298

Furcifer_pardalis_Acr ------------GFRQPS--AATSDVFVEP-TGLINMEPSE--LREPPTTLVRLDKGDNR 298

Chamaeleo_calyptratus_AMTN_Acr ------------GFRQPS--VATSAVFVEP-TGLINMEPSE--LREPPTSLVRLDNADNR 298

Chamaeleo_dilepis_AMTN_Acr ------------GFRQPS--AATSAVFVEP-TGLINMEPSE--LREPPTSLVRLDNGDNR 298

Chamaeleo_gracilis_AMTN_Acr ------------GFRQPS--AATSAVFVEP-TGLINLEPSE--LREPPTSLVRLDNGDNR 298

Chamaeleo_laevigatus_AMTN_Acr ------------GFRQPS--AATSAVFVEP-TGLINMEPSE--LREPPTSLVRLDNGDNR 298

Trioceros_affinis_AMTN_Acr ------------GFRQPS--AATSAVFVEP-TGLINMEPSE--LREPPTTLVRLDKGDNR 298

Trioceros_balebicornutus_AMTN_Acr ------------GFRQPS--AATSAVFVEP-TGLINMEPSE--LREPPTTLVRLDKGDNR 298

Trioceros_harennae_AMTN_Acr ------------GFRQPS--AATSAVFVEP-TGLINMEPSE--LREPPTTLVRLDKGDNR 298

Anolis_apletophallus_Pleu ------------GFRQPG--VVTNEVFVEP-TGGINMEPSE--LREPPTSLVGLDKGKNQ 305

Anolis_sagrei_ordinatus_Pleu ------------GFRQPG--VVTNEVFVEP-TGGINMEPSE--LREPPTSLVGLDNGKNQ 303

Anolis_tropidonotus_AMTN_Acr ------------GFRQPG--VVTNEVFVEP-TGGINMEPSE--LREPPTSLVGLDKGKNQ 303

Anolis_carolinensis_Pleu ------------GFRQPG--VVTNEVFVEP-TGGIHMEPGE--LREPPTSLVGLDKGNNQ 305

Anolis_rodriguezii_AMTN_Pleur ------------GFRQPG--VVTNEVFVEP-IGGINMEPSE--LREPPTSLVGLDKGKNE 308

Ctenosaura_bakeri_Pleu ------------GFRQPG--VVTNEVFVEP-TIDINMEPSE--LREPPTSPVRLYKGDNQ 304

Iguana_delicatissima_Pleu ------------GFRQPG--VVTNEVFVEP-TIDINMEPSE--LREPPTSPVRLYKGNDQ 304

Cyclura_pinguis_Acr ------------GFRQPG--VVTNEVFAEP-TIDINMEPSE--LREPPTSPVRFYKGNNQ 304

Gambelia_wislizenii_Acr ------------GFRQPG--VVTNEVFIEP-TVGINMEPSE--LREPPTTVARLDKGNNQ 304

Laemanctus_serratus_AMTN_Acr ------------GFRQPG--VVTNEVFVEP-TVGINMEPSE--LREPPTSLVKLDKGNNQ 296

Phrynosoma_blainvillii_Pleu ------------GIRQPG--VVTNEVFVEP-TVGINMEPSE--LREPPTSLVKLDKGNNQ 287

Phrynosoma_platyrhinos_Pleu ------------GFRQPG--VVTNEVFVEP-TVGINMEPSE--LREPPTSLVKLDKGNNQ 287

Sceloparus_undulatus_Pleu ------------GFRQPG--VVTNEVFVEP-TVGINMEPSE--LREPPTSLVKLDNGNNQ 286

Sceloporus_tristichus_Pleu ------------GFRQPG--VVTNEVFVEP-TVGINMEPSE--LREPPTSLVKLDNGNNQ 286

Sceloporus_occidentalis_Pleu ------------GFRQPG--VVTNEVFVEP-TVGINMEPSE--LREPPTSLVKLDKDNNQ 287

Sceloporus_chrysostictus_AMTN_Pleur ------------GFRHPG--VVTNEVFVEP-TVGINMEPSE--LREPPTSLVKLDKGNNQ 288

Urosaurus_nigricaudus_Pleu ------------GFRQPG--VVTNGVFVEP-TVGINMEPSE--LREPPTSLVKLDKGNNQ 288

Canis_lupus_dingo_AMTN ------------------------------------------------------------

Vulpes_vulpes_AMTN -----------KCCRSHR--RVGTGFFVLP------------------------------ 232

Meles_meles_AMTN -----------RKSQKV--------YFALPPSDANAAEPGDHALNTPPSPPRMMRNGGSI 256

Mustela_putorius_AMTN ------------------------------------------------------------

Ursus_arctos_AMTN -----------KCLRKVI-CRVETEFFLLS------------------------------ 233

Phacochoerus_africanus_AMTN ------------------------------------------------------------

Homo_sapiens_AMTN ------------------------------------------------------------

Saimiri_boliviensis_AMTN ------------------------------------------------------------

Phyllostomus_hastatus_AMTN ------------------------------------------------------------

Erinaceus_europaeus_AMTN ------------------------------------------------------------

Suncus_etruscus_AMTN QAGSKAVAFYNVSFETRHMAGIRLHLLQAK-TAYLVMSPEE--IMFAGAFLCRLKQENRK 272

Echinops_telfairi_AMTN D-----VLFHSRILEEPH--------I------FLT------------------------ 207

Acanthocercus_cyanogaster_AMTN_Acr KQPNLFTTFLRGDNYMPTTTTESKPWK--------------------------------- 321

Acanthocercus_minutus_AMTN_Acr KQPNLFPTLLRGDNYMPTTTTESKPWK--------------------------------- 321

Xenagama_zonura_AMTN_Acr KQPNLFPTLLRGDNYMPTTTTESKPWK--------------------------------- 321

Laudakia_wui_Acr KHHNVFPTLLRGDSYMPTTTTESKPWK--------------------------------- 322

Phrynocephalus_forsythii_Acr KHQKLFPTILRGDHYMATTTIKSKPWK--------------------------------- 321

Phrynocephalus_guinanensis_Acr KHQKLFPTLLRGDNYMPTTTIKSKPWK--------------------------------- 321

Phrynocephalus_putjatai_Acr KHQKLFPTLLRGDNYMPTTTIKSKPWK--------------------------------- 321

Phrynocephalus_versicolor_Acr KHQKLFPTLLRGDNYMPTTTIKSKPWK--------------------------------- 321

Phrynocephalus_vlangalii_Acr KHQKLFPTLLRGDNYMPTTTIKSKPWK--------------------------------- 321

Agama_doriae_AMTN_Acr KQPNLFPTLLRGDSYMPTTTTKSKPWK--------------------------------- 321

Intellagama_lesueurii_Acr KNQNLFQAFLRGDNHMPITTTESKPWK--------------------------------- 321

Pogona_vitticeps_Acr KHQNLFQAFLRGDNHMPITTTESKPWK--------------------------------- 321

Bradypodion_pumilum_Acr KHHNLFQALLRGDTPMPRTTTQSKPWK--------------------------------- 325

Bradypodion_ventrale_Acr KHHNLFQALLRGDTPMPRTTTQSKPWK--------------------------------- 325

Furcifer_pardalis_Acr KHHNLFQALLRGDTPMPRTTTESKPWK--------------------------------- 325

Chamaeleo_calyptratus_AMTN_Acr KHHNLFQALLRGDTPMPRTTTQSKPWK--------------------------------- 325

Chamaeleo_dilepis_AMTN_Acr KHHNLFQALLRGDTPMPRTTTQSKPWK--------------------------------- 325

Chamaeleo_gracilis_AMTN_Acr KHHNLFQALLRGDTPMPRTTTQSKPWK--------------------------------- 325

Chamaeleo_laevigatus_AMTN_Acr KHHNLFQALLRGDTPMPRTTTQSKPWK--------------------------------- 325

Trioceros_affinis_AMTN_Acr KHYNLFQALLRGDTPMPRTTTQSKPWK--------------------------------- 325

Trioceros_balebicornutus_AMTN_Acr KHYNLFQALLRGDTPMPRTTTQSKPWK--------------------------------- 325

Trioceros_harennae_AMTN_Acr KHYNLFQALLRGDTPMPRTTTQSKPWK--------------------------------- 325

Anolis_apletophallus_Pleu KHQNLSQSPVRGDSYMPMTTTASKPLK--------------------------------- 332

Anolis_sagrei_ordinatus_Pleu KHQNLSQLPVRGDSYMPMTTTASKPLK--------------------------------- 330

Anolis_tropidonotus_AMTN_Acr KHQNLSQSPVRGDSYMPMTTTASKPLK--------------------------------- 330

Anolis_carolinensis_Pleu KLQNLSQSPVRGDSYMPMTTTASKPLK--------------------------------- 332

Anolis_rodriguezii_AMTN_Pleur KHQNLSQSPVRGDSYMPMTTTASKPLK--------------------------------- 335

Ctenosaura_bakeri_Pleu KHQNLSQSPVRGDSHMPINTTESKPLK--------------------------------- 331

Iguana_delicatissima_Pleu KHQNLSQSPVRGDSHMPINTTESKPLK--------------------------------- 331

Cyclura_pinguis_Acr KYQNLSQSPVRGDSHMPINTTESKPLE--------------------------------- 331

Gambelia_wislizenii_Acr KHQNLSQSLVRGDSHMPISTTENQPLK--------------------------------- 331

Laemanctus_serratus_AMTN_Acr KYQNLSQSPVRGDSYMPIHTTESKPLK--------------------------------- 323

Phrynosoma_blainvillii_Pleu KHQNLSQSLVRGDSHIPINTTESKPLK--------------------------------- 314

Phrynosoma_platyrhinos_Pleu KHQNLSQSPVRGDSHIPINTTESKPLK--------------------------------- 314

Sceloparus_undulatus_Pleu KHQNLSQSPVRGDSHMPINTTESKPLK--------------------------------- 313

Sceloporus_tristichus_Pleu KHQNLSQSPVRGDSHMPINTTESKPLK--------------------------------- 313

Sceloporus_occidentalis_Pleu KHQNLSQSPVRGDSHMPINTTESKPLK--------------------------------- 314

Sceloporus_chrysostictus_AMTN_Pleur KHQNLSQSPVRGDSHMPINTTESKPLK--------------------------------- 315

Urosaurus_nigricaudus_Pleu KHQNLSQSPVRGDSHMPINTTESKPLK--------------------------------- 315

Canis_lupus_dingo_AMTN ------------------------------------------------------------

Vulpes_vulpes_AMTN ------------------------------------------------------------

Meles_meles_AMTN W---FSLSLIR-DSLLQYSLEGTEILKLRFLQTIAVSMKFRAGIANHNAFIGKTGNAHER 312

Mustela_putorius_AMTN ------------------------------------------------------------

Ursus_arctos_AMTN ------------------------------------------------------------

Phacochoerus_africanus_AMTN ------------------------------------------------------------

Homo_sapiens_AMTN ------------------------------------------------------------

Saimiri_boliviensis_AMTN ------------------------------------------------------------

Phyllostomus_hastatus_AMTN ------------------------------------------------------------

Erinaceus_europaeus_AMTN ------------------------------------------------------------

Suncus_etruscus_AMTN PSREQISGLL-------------------------------------------------- 282

Echinops_telfairi_AMTN ------------------------------------------------------------

Acanthocercus_cyanogaster_AMTN_Acr ------------------------------------------------------------

Acanthocercus_minutus_AMTN_Acr ------------------------------------------------------------

Xenagama_zonura_AMTN_Acr ------------------------------------------------------------

Laudakia_wui_Acr ------------------------------------------------------------

Phrynocephalus_forsythii_Acr ------------------------------------------------------------

Phrynocephalus_guinanensis_Acr ------------------------------------------------------------

Phrynocephalus_putjatai_Acr ------------------------------------------------------------

Phrynocephalus_versicolor_Acr ------------------------------------------------------------

Phrynocephalus_vlangalii_Acr ------------------------------------------------------------

Agama_doriae_AMTN_Acr ------------------------------------------------------------

Intellagama_lesueurii_Acr ------------------------------------------------------------

Pogona_vitticeps_Acr ------------------------------------------------------------

Bradypodion_pumilum_Acr ------------------------------------------------------------

Bradypodion_ventrale_Acr ------------------------------------------------------------

Furcifer_pardalis_Acr ------------------------------------------------------------

Chamaeleo_calyptratus_AMTN_Acr ------------------------------------------------------------

Chamaeleo_dilepis_AMTN_Acr ------------------------------------------------------------

Chamaeleo_gracilis_AMTN_Acr ------------------------------------------------------------

Chamaeleo_laevigatus_AMTN_Acr ------------------------------------------------------------

Trioceros_affinis_AMTN_Acr ------------------------------------------------------------

Trioceros_balebicornutus_AMTN_Acr ------------------------------------------------------------

Trioceros_harennae_AMTN_Acr ------------------------------------------------------------

Anolis_apletophallus_Pleu ------------------------------------------------------------

Anolis_sagrei_ordinatus_Pleu ------------------------------------------------------------

Anolis_tropidonotus_AMTN_Acr ------------------------------------------------------------

Anolis_carolinensis_Pleu ------------------------------------------------------------

Anolis_rodriguezii_AMTN_Pleur ------------------------------------------------------------

Ctenosaura_bakeri_Pleu ------------------------------------------------------------

Iguana_delicatissima_Pleu ------------------------------------------------------------

Cyclura_pinguis_Acr ------------------------------------------------------------

Gambelia_wislizenii_Acr ------------------------------------------------------------

Laemanctus_serratus_AMTN_Acr ------------------------------------------------------------

Phrynosoma_blainvillii_Pleu ------------------------------------------------------------

Phrynosoma_platyrhinos_Pleu ------------------------------------------------------------

Sceloparus_undulatus_Pleu ------------------------------------------------------------

Sceloporus_tristichus_Pleu ------------------------------------------------------------

Sceloporus_occidentalis_Pleu ------------------------------------------------------------

Sceloporus_chrysostictus_AMTN_Pleur ------------------------------------------------------------

Urosaurus_nigricaudus_Pleu ------------------------------------------------------------

Canis_lupus_dingo_AMTN ------------------------------------------------------------

Vulpes_vulpes_AMTN ------------------------------------------------------------

Meles_meles_AMTN SQPGVRRNKKMLVLLHPILGQSDHSEGETLFATISNNVCPPSVCARQESTWLHTHRRRPR 372

Mustela_putorius_AMTN ------------------------------------------------------------

Ursus_arctos_AMTN ------------------------------------------------------------

Phacochoerus_africanus_AMTN ------------------------------------------------------------

Homo_sapiens_AMTN ------------------------------------------------------------

Saimiri_boliviensis_AMTN ------------------------------------------------------------

Phyllostomus_hastatus_AMTN ------------------------------------------------------------

Erinaceus_europaeus_AMTN ------------------------------------------------------------

Suncus_etruscus_AMTN ------------------------------------------------------------

Echinops_telfairi_AMTN ------------------------------------------------------------

Acanthocercus_cyanogaster_AMTN_Acr ------------------AP 323

Acanthocercus_minutus_AMTN_Acr ------------------AP 323

Xenagama_zonura_AMTN_Acr ------------------AP 323

Laudakia_wui_Acr ------------------AP 324

Phrynocephalus_forsythii_Acr ------------------AP 323

Phrynocephalus_guinanensis_Acr ------------------AP 323

Phrynocephalus_putjatai_Acr ------------------AP 323

Phrynocephalus_versicolor_Acr ------------------AP 323

Phrynocephalus_vlangalii_Acr ------------------AP 323

Agama_doriae_AMTN_Acr ------------------AP 323

Intellagama_lesueurii_Acr ------------------AP 323

Pogona_vitticeps_Acr ------------------AP 323

Bradypodion_pumilum_Acr ------------------AP 327

Bradypodion_ventrale_Acr ------------------AP 327

Furcifer_pardalis_Acr ------------------AP 327

Chamaeleo_calyptratus_AMTN_Acr ------------------AP 327

Chamaeleo_dilepis_AMTN_Acr ------------------AP 327

Chamaeleo_gracilis_AMTN_Acr ------------------AP 327

Chamaeleo_laevigatus_AMTN_Acr ------------------AP 327

Trioceros_affinis_AMTN_Acr ------------------AP 327

Trioceros_balebicornutus_AMTN_Acr ------------------AP 327

Trioceros_harennae_AMTN_Acr ------------------AP 327

Anolis_apletophallus_Pleu ------------------AP 334

Anolis_sagrei_ordinatus_Pleu ------------------AP 332

Anolis_tropidonotus_AMTN_Acr ------------------AP 332

Anolis_carolinensis_Pleu ------------------AP 334

Anolis_rodriguezii_AMTN_Pleur ------------------AP 337

Ctenosaura_bakeri_Pleu ------------------AP 333

Iguana_delicatissima_Pleu ------------------AP 333

Cyclura_pinguis_Acr ------------------AP 333

Gambelia_wislizenii_Acr ------------------AP 333

Laemanctus_serratus_AMTN_Acr ------------------AP 325

Phrynosoma_blainvillii_Pleu ------------------AP 316

Phrynosoma_platyrhinos_Pleu ------------------AP 316

Sceloparus_undulatus_Pleu ------------------AP 315

Sceloporus_tristichus_Pleu ------------------AP 315

Sceloporus_occidentalis_Pleu ------------------AP 316

Sceloporus_chrysostictus_AMTN_Pleur ------------------AP 317

Urosaurus_nigricaudus_Pleu ------------------AP 317

Canis_lupus_dingo_AMTN --------------------

Vulpes_vulpes_AMTN --------------------

Meles_meles_AMTN RSDLNILRHKAEDEGEGPSP 392

Mustela_putorius_AMTN --------------------

Ursus_arctos_AMTN --------------------

Phacochoerus_africanus_AMTN --------------------

Homo_sapiens_AMTN --------------------

Saimiri_boliviensis_AMTN --------------------

Phyllostomus_hastatus_AMTN --------------------

Erinaceus_europaeus_AMTN --------------------

Suncus_etruscus_AMTN --------------------

Echinops_telfairi_AMTN --------------------

**AMTN-AA-MAFFT-Iguania+Mammal – DIVERGE-Acro-Pleuro DIVERGE-Agam-Cham-NotSignificant**

CLUSTAL W (1.8) multiple sequence alignment (ALTER 1.3.3)

Acanthocercus_cyanogaster_AMTN_Acr MKIVILLFSSLGLTLSIPYNQFGRHLATSNSREILRLMQKYRAQGNIPQQTQQRPNPGVG 60

Acanthocercus_minutus_AMTN_Acr MKIVILLFSSLGLTLSLPYNQFGRHLATSNSREILRLMQKYRAQGNIPQQTQQRLNPGVG 60

Xenagama_zonura_AMTN_Acr MKIVILLFSSLGLTLSLPYNQFGRHLATSNSREILRLMQKYRAQGNIPQQTQQRPNPGVG 60

Laudakia_wui_Acr MKIVILLLSSLGLTFSLPYNQFGRHFATSNSREILRLMQKYRAQGNIPQQTQQRPNPGIG 60

Phrynocephalus_forsythii_Acr MKIVILLFSSLGLTFSLPYNQFGRHFATSNSREILRLMQKYRAQGNVPQQTQQRPNPGVG 60

Phrynocephalus_guinanensis_Acr MKIVILLFSSLGLTFSLPYNQFGRHFATSNSKEILRLMQKYRAQGNVPQQTQQRPNPGVG 60

Phrynocephalus_putjatai_Acr MKIVILLFSSLGLTFSLPYNQFGRHFATSNSREILRLMQKYRAQGNVPQQTQQRPNPGVG 60

Phrynocephalus_versicolor_Acr MKIVILLFSSLGLTFSLPYNQFGRHFATSNSREILRLMQKYRAQGNVPQQTQQRPNPGVG 60

Phrynocephalus_vlangalii_Acr MKIVILLFSSLGLTFSLPYNQFGRHFATSNSREILRLMQKYRAQGNVPQQTQQRPNPGVG 60

Agama_doriae_AMTN_Acr MKIVILLFSSLGLTFSLPYNQLRRHLATSNSREILRLMQKYRAQGNIPQQTQQRPNPGVG 60

Intellagama_lesueurii_Acr MKIVILLLFSVGLTFSLPYNQFGRLLATSNSREILRLMQKYRAQGNIPQQTQQRTNPGTG 60

Pogona_vitticeps_Acr MKIVILLLFSVGLTFSLPYNQFGRLLATSNSREILRLMQKYRAQGNNPQQTQQRPNPGTG 60

Bradypodion_pumilum_Acr MKIVILLLYSLGLTFALP---FNRLLATSNSREILRLMQKYKTQGNVPQKAQQRPNLVTR 57

Bradypodion_ventrale_Acr MKIVILLLYSLGLTFALP---FNRLLATSNSREILRLMQKYKTQGNVPQKAQQGPNLVTR 57

Furcifer_pardalis_Acr MKIVILLLYSLGLTFALP---FDRLLATSNSREILRLMQKYKTQGNVPQKAQQRPNLVTR 57

Chamaeleo_calyptratus_AMTN_Acr MKIVILLLYSLGLTFALP---FDRLLATSNSREILRLMQKYKTQGNVPQKAQQRPNLVTR 57

Chamaeleo_dilepis_AMTN_Acr MKIVILLLYSLGLTFALP---FDRLLATSNSREILRLMQKYKNQGNVPQKAQQRPNLVTR 57

Chamaeleo_gracilis_AMTN_Acr MKIVILLLYSLGLTFALP---FDRLLATSNSREILRLMQKYKTQGNVPQKAQQRPNLVTR 57

Chamaeleo_laevigatus_AMTN_Acr MKIVILLLYSLGLTFALP---FDRLLATSNSREXXXXXXXXXXXXXXXXXXXQRPNLVTR 57

Trioceros_affinis_AMTN_Acr MKVVILLLYSLGLTFALP---FDRLLATSNSREILRLMQKYKTQGNVPQKAQQRPNLVTR 57

Trioceros_balebicornutus_AMTN_Acr MKIVILLLYSLGLTFALP---XXXXXXXXXXXXILRLMQKYKTQGNVPQKAQQRPNLVTR 57

Trioceros_harennae_AMTN_Acr MKIVILLLYSLGLTFALP---FDRLLATSNSREILRLMQKYKTQGNVPQKAQQRPNLVTR 57

Anolis_apletophallus_Pleu MKIVILLLSLLGLTFCLPVNQFGRHFATSNSREILRLMQRYKAQGNVPQQTQQRSNPGIG 60

Anolis_sagrei_ordinatus_Pleu MKIVILLLSLLGLTFCLPVNQFGRRFATSNSREILRLMQRYRAQGNIPQQTQPRSNPGIG 60

Anolis_tropidonotus_AMTN_Acr MKIVILLLSLLGLTFCLPVNQFGRRFATSNSREILRLMQRYKAQGNVPQQTQQRSNPGIG 60

Anolis_carolinensis_Pleu MKIVILLLSLLGLTFCLPVNQFGRRFATSNSREILRLMQRYKAQGNVPQQTQQRSNPGIG 60

Anolis_rodriguezii_AMTN_Pleur MKIVILLLSLLGMTFCLPVNQFGRGFATSNSREILRLMQRYKAQGNVPQQTQQRSNPGIG 60

Ctenosaura_bakeri_Pleu MKIVILLLSLLGLTFCLPVNQFGRLLATSNSRERLRLMQRYKAQGNIPQQTQQRPNSGVG 60

Iguana_delicatissima_Pleu MKIVILLLSFLGLTSCLPVNQFGRLLATSNSRERLRLMQRYKAQGNIPQQTQQRPNSGVG 60

Cyclura_pinguis_Acr MKIVILLLSLLGVTFCLPVNQFGRLLATSNSRERLRLMQRYKAQGNIPQQTQQRPNSGVG 60

Gambelia_wislizenii_Acr MKIVILLLSLLGLTFCLPVNQFGRHFATSNSREILRLMQRYKAQENSPQQTQQRPNSGVG 60

Laemanctus_serratus_AMTN_Acr MKIVILLLSLLGLTFCLPVNQFGRRLATSNSREILRLMQRYKARGNIPQQTQQRPNSGVG 60

Phrynosoma_blainvillii_Pleu MNIVILLLSLMGLTFCLPVNQFGRLLATSNSREILQLMQRYKAQGNIPQQTQQRPNPGVG 60

Phrynosoma_platyrhinos_Pleu MNIVILLLSFLGMTFCLPVNQFGKLLATSNSREILQLMQRYKAQGNIPQQTQQRPNPGVG 60

Sceloparus_undulatus_Pleu MKIVILLLSLLGLTFCLPVNQFGRRLATSNSREILRL-QRYKAQGNIPQQTQQRPHPGVG 59

Sceloporus_tristichus_Pleu MKIVILLPSLLGLTFCLPVNQFGRRLATSNSREILRL-QRYKAQGNIPQQTQQRPHPGVG 59

Sceloporus_occidentalis_Pleu MKIVILLLSLLGLTFCLPVNQLGRRLATSNSREILRFMQRYKAQGNIQQQTQQRPHPGVG 60

Sceloporus_chrysostictus_AMTN_Pleur MKIVILLLSLLGLTFCLPVNQFGRRFATSNSREILRLMQKYKAQGNIPQQTQQRPNPGAG 60

Urosaurus_nigricaudus_Pleu MKIVILLLSLLGLTFGLPVKIFGRLLGTSNSREILQFMQRYKDQATIPQQPQQRPNPGVG 60

*::**** :*:* :* :

Acanthocercus_cyanogaster_AMTN_Acr LPPAKLVPDQPPLANQAPNE-VIPFVWPNLPALPVLPVQTPLE------QNVAGFNVLQL 113

Acanthocercus_minutus_AMTN_Acr LPPAKLVPDQPPLANQAPNE-VIPFVWPNLPALPVLPVQTPLE------QNVAGFNVLQL 113

Xenagama_zonura_AMTN_Acr LPPPKLVPDQPPLANQAPNE-VIPFVWPNLPALPVLPVQTPLE------QNVAGFNVLQL 113

Laudakia_wui_Acr LPPAKLVPDQPPLANQVPNEQVISFGWPNLPGLPVLPAQTPLE------QTVAGFNVLQL 114

Phrynocephalus_forsythii_Acr LPPAKLVPDQPPLASQVPNE-FIPFGWPNLPGLPVLPAQTQLE------QNAAGFSVLQL 113

Phrynocephalus_guinanensis_Acr LPPAKLVPDQPPLASQVPNE-FIPFGWPNLPGLPVLPAQTPLE------QNAAGFNVLQL 113

Phrynocephalus_putjatai_Acr LPPAKLVPDQPPLASQVPNE-FIPFGWPNLPGLPVLPAQTPLE------QNAAGFNVLQL 113

Phrynocephalus_versicolor_Acr LPPAKLVPDQPPLASQVPNE-FIPFGWPNLPGLPVLPAQTPLE------QNAAGFNVLQL 113

Phrynocephalus_vlangalii_Acr LPPAKLVPDQPPLASQVPNE-FIPFGWPYLPGLPVLPAQTPLE------QNAAGFNVLQL 113

Agama_doriae_AMTN_Acr LPPLKLVPDQPPLANQVPNE-IIPFGWPNFQAFPVLPAQTPLE------QNVAGFNVLQL 113

Intellagama_lesueurii_Acr LPPAKLIPDQHPLANQVPNE-VVPFAWPNLPNFPVLPAQTPLE------QNVAGYNVLPL 113

Pogona_vitticeps_Acr LPPAKLIPDQHPLANQVPNE-VVPFAWPNFPNFPVLPAQTPLE------QNVAGYNVLPL 113

Bradypodion_pumilum_Acr LPPAKLVPDRHPLSNQVPHELVFPFEW---ANLPVLPPHIPLEQGSPNLQNFAGFNVLPM 114

Bradypodion_ventrale_Acr LPPAKLVPDRRPLSNQVPHELVFPFEW---ANLPVLPPHIPLEQGSPNLQNFAGFNVLPM 114

Furcifer_pardalis_Acr LPPAKLVPDQHPLSNQVPNELVFPFEW---ANLPVLPPHIPLEQGSPNLQNFPGFNVLPM 114

Chamaeleo_calyptratus_AMTN_Acr LPPAKLVPDQHPLSNQVPNELVFPFEW---ANLPVLPPHIPLEQGSPNLQNFAGFNVLPM 114

Chamaeleo_dilepis_AMTN_Acr LPPAKLVPDQHPLSNQVPNEMVVPFEW---ANLPVLPPHIPLEQGSPNLQNFAGFNVLPM 114

Chamaeleo_gracilis_AMTN_Acr LPPAKLVPDQHPLSNQVPNELAVPFEW---ANLPVLPPHIPLEQGSPNLQNFAGFNVLPM 114

Chamaeleo_laevigatus_AMTN_Acr LPPAKLVPDQHPLSNQVPNELVFPFEW---ANLPVLPPHIPLEQGSPNLQNFAGFNVLPM 114

Trioceros_affinis_AMTN_Acr LPPSKLVPDWQPLSNQVANEXXXXXXX---XXXXXXXXXXXXXXXXXXXXNFAGFNVLPM 114

Trioceros_balebicornutus_AMTN_Acr LPPSKLVPDWQPLSNQVANELVFPFEW---ANLPVLPPHIPLEQGSPNLQNFAGFNVLPM 114

Trioceros_harennae_AMTN_Acr LPPSKLVPGWQPLSNQVANELVFPFEW---ANLPVLPPHIPLEQGSPNLQNFAGFNVLPM 114

Anolis_apletophallus_Pleu LPPAKLVPDQHPLANHIPNE-IPAIEWP-FANYPFLTARNPQELGSPTIPNLPGLNVLPL 118

Anolis_sagrei_ordinatus_Pleu LPPAKLVPDQHPLANHIPNE-IPAIEWP-FGNYPILTAQNPQELGSPTVQNFPGFNVLPF 118

Anolis_tropidonotus_AMTN_Acr LPPAKLVPDQHPLANHIPNE-IPAIEWP-FANYPILTAQNPQELGSPTIQNFPGFNVLPL 118

Anolis_carolinensis_Pleu LPPAKLVPDQHPLANHIPSE-IPAFERP-FANYPILTAQNPQELGSPTLQNFPGFNVLPL 118

Anolis_rodriguezii_AMTN_Pleur LPPAKLVPDQHPLANHIPNE-IPAIAWP-FANYPFLTAQNPQELGTPIIQNFPGFNVLPF 118

Ctenosaura_bakeri_Pleu LPPAKLVPDQQPFANQIPNE-IPAFEWQ-FANFPVFSPQNPLELGSPNVQNFPGLSVLPL 118

Iguana_delicatissima_Pleu LPPAKLVPDQQPFTNQIPNE-IPAFEWQ-FANFPVFSPQNPLELGSPNVQNFPGLSVLPL 118

Cyclura_pinguis_Acr LPPAKLVPDQQPFANQIPNE-IPAFEWQ-FANFPVFSPQNPLELGSPNVQNFPGLNVLPL 118

Gambelia_wislizenii_Acr LPPAKLVPDQQPLANQIPNE-MLPFEWL-FPNFPIFSPQNALELGNPNVQNFPGSSVLPL 118

Laemanctus_serratus_AMTN_Acr LPPAKLVPDLQ----QIPNE-ILPFEWP-VANFPIFSPQNTLELGSPNVQNSPGFNVQPS 114

Phrynosoma_blainvillii_Pleu LPPAKLVPDQQPLGNQIPNE-ILPFELP-FANLPIFFPQNRLELGSPNVQNVPGFSVLPL 118

Phrynosoma_platyrhinos_Pleu LPPAKLVPDQQPLGNQIPNE-ILPFELP-FANFPIFFPQNRLELGSPNVQNVPGFNALPL 118

Sceloparus_undulatus_Pleu LPPAKLVPDQQLLANQIPNE-ILPFEWP-FANFPVFAPQNRLELGSPKAQNAPGFNVLPL 117

Sceloporus_tristichus_Pleu LPPAKLVPDQQLLANQIPNE-ILPFEWP-FANFPVFAPQNRLELGSPNAQNAPGFNVLPL 117

Sceloporus_occidentalis_Pleu LPPAKLVPDQQLLANQIPNE-ILPFEFP-FANFPVFAPQNRLELGSPNAQNAPGFNVLPL 118

Sceloporus_chrysostictus_AMTN_Pleur LPPAKLVPDQQILANQIPSE-ILPFELP-FTNFPVFAPQSRLELGNPNVQNAPGFNVLPL 118

Urosaurus_nigricaudus_Pleu LPPAKLVPDQQLLANQIPSE-ILPFESP-FANFPVLAPQNRLELGSPNVQNIPPFNVLPL 118

*** **:*. : . * . . ..

Acanthocercus_cyanogaster_AMTN_Acr PQMFPLDVNYLVALLSALSGTETLPLAGGGLTV------P--APP---MLPIIFTQMGPQ 162

Acanthocercus_minutus_AMTN_Acr PQMFPLDVNYLVALLSALSGTETLPLAGGGLTV------P--APP---MLPIIFTQMGPQ 162

Xenagama_zonura_AMTN_Acr PQMFPLDVNYLVALLSALSGTETLPLAGGGLTV------P--APP---MLPIIFTQMGPQ 162

Laudakia_wui_Acr PQMFPLDVNYLVALLSALSGTETLPLAGGGLTV------P--APP---MLPIIFTQMGPQ 163

Phrynocephalus_forsythii_Acr PQMFPLDVNYVVALLSALSGTETLPLAGGGLTV------P--ASP---MLPIIFTQMGPQ 162

Phrynocephalus_guinanensis_Acr PQMFPLDVNYLVALLSALSGTETLPLAGGGLTV------P--ASP---MLPIIFTQMGPQ 162

Phrynocephalus_putjatai_Acr PQMFPLDVNYLVALLSALSGTETLPLAGGGLTV------P--ASP---MLPIIFTQMGPQ 162

Phrynocephalus_versicolor_Acr PQMFPLDVNYLVALLSALSGTETLPLAGGGLTV------P--ASP---MLPIIFTQMGPQ 162

Phrynocephalus_vlangalii_Acr PQMFPLDVNYLVALLSALSGTETLPLAGGGLTV------P--ASP---MLPIIFTQMGPQ 162

Agama_doriae_AMTN_Acr PQMFPLDVNYLVALLSALSGTETLPLAGGGLTV------P--APPGFFYLPVLCTEM--- 162

Intellagama_lesueurii_Acr PQMFPLDVNSLVALLAALSGTETLPLPGGGLTV------P--APP---MLPVLFTQMGPQ 162

Pogona_vitticeps_Acr PQMFPLDINSLVALLAALSGTETLPLPGGGLTV------P--APP---MLPVLFTQMGPQ 162

Bradypodion_pumilum_Acr TQMFPLDINYLVALLAALSGTQTLPLAAGGLTV------P--APP---MLPIILTQMAPQ 163

Bradypodion_ventrale_Acr TQMLPLDINYLVALLAALSGTQTLPLAAGGLTV------P--APP---MLPIILTQMAPQ 163

Furcifer_pardalis_Acr TQMFPLDINYLVALLTALSGTQTFPLAAGGLTV------P--APP---MLPIILTQMGPQ 163

Chamaeleo_calyptratus_AMTN_Acr TQMFPLDINYLVALLTALSGTQTLPLVAGGLTV------P--APP---MLPIILTQMGPQ 163

Chamaeleo_dilepis_AMTN_Acr TQMFPLDINYLVALLTALSGTQTLPLVAGGLTV------P--APP---MLPIILTQMGPQ 163

Chamaeleo_gracilis_AMTN_Acr TQMFPLDINYLVALLTALSGTQTLPLVAGGLTV------P--APP---MLPIILTQMGPQ 163

Chamaeleo_laevigatus_AMTN_Acr TQMFPLDINYLVALLTALSGTQTLPLVAGGLTV------P--APP---MLPIILTQMGPQ 163

Trioceros_affinis_AMTN_Acr TQMFPLDINYLVALLTALSGTQTLPLAAGGLTV------P--SPP---MLPIILTQMGPQ 163

Trioceros_balebicornutus_AMTN_Acr TQMFPLDINYLVALLTALSGTQTLPLAAGGLTV------P--APP---MLPIILTQMGPQ 163

Trioceros_harennae_AMTN_Acr TQMFPLDINYLVALLTALSGTQTLPLAAGGLTV------P--APP---MLPIILTQMGPQ 163

Anolis_apletophallus_Pleu TQMVPIDINYLALLLGALSQTQTQPLPGGPL-IPQQLL-P--PQS---MLPIIFTQMGPQ 171

Anolis_sagrei_ordinatus_Pleu TQMVPIDINSLALLLGALSTTQAQPLPGGGL-IPQQLL-P--PPP---MFPIIFTQMGPQ 171

Anolis_tropidonotus_AMTN_Acr TQMVPIDINYLALLLGALSQTQTQPLPGGGL-IPQQLL-P--PQS---MLPIIFTQMGPQ 171

Anolis_carolinensis_Pleu TQMVPIDINYLALLLGALSTTQTQPLPGGGLNIPQQLLPP--PQS---MFPIIFTQMGPQ 173

Anolis_rodriguezii_AMTN_Pleur TQMVPIDINTLALLLGALSTTQTQPLPGGGL-IPQQLL-PPLPQS---XXXXXXXXXXXX 173

Ctenosaura_bakeri_Pleu NQMVPIDINSLAVLLGAIPPSQTQPLPGGGINIPQQLL-P--AQA---MLPIIFTQMGPQ 172

Iguana_delicatissima_Pleu NQMVPIDINSLAVLLGAIPPSQTQPLPGGGINIPQQLL-P--AQS---MLPIIFTQMGPQ 172

Cyclura_pinguis_Acr NQMVPIDINSLAVLLGAISPSQTQPLPGGGINIPQQLL-P--AQS---MLPIIFTQMGPQ 172

Gambelia_wislizenii_Acr TQMVPIDINSLAELLRVLSATQTQPLPGGGITGPQQLL-P--AQS---MLPIIFTQMGPQ 172

Laemanctus_serratus_AMTN_Acr NLMVPIDINSLAMLLAALSTTQTS--MGGGITVPEQLL-P--PQP---MLPIIFTQMGPQ 166

Phrynosoma_blainvillii_Pleu TQMAPIDINSLAVLLGALSTAQTQP----------PLL-S--PL----MFPVVFTQRGPQ 161

Phrynosoma_platyrhinos_Pleu TQMAPIDINSLAVLLGALSTAQTQP----------PLL-S--PL----MFPVVFTQMGPQ 161

Sceloparus_undulatus_Pleu TQMVPMDINALAVLLGALSTAQIQQ----------QLL-P--PQP---MLPIIFTQMGPQ 161

Sceloporus_tristichus_Pleu TQMVPMDINALAVLLGALSTAQIQQ----------QLL-P--PQP---MLPIIFTQMGPQ 161

Sceloporus_occidentalis_Pleu TQMVPMDINALAVLLGALSTAQIQQ----------QLL-S--PQP---MLPIIFTQMGPQ 162

Sceloporus_chrysostictus_AMTN_Pleur TQMVPMDINALAVLLGALSAAQTQP----------QLL-P--PQP---MLPIIFTQMGPQ 162

Urosaurus_nigricaudus_Pleu TQMVPMDISALAVLLGALSTAQTQP----------QLL-P--PQA---MLPIIFTQMGPQ 162

* *:*:. :. ** .:. :: . .

Acanthocercus_cyanogaster_AMTN_Acr GQVLSSEEMPPSQVFAGVL---LPGTQG---GLLPAGQSGSLPEGQEGLLPAGQTGTNQG 216

Acanthocercus_minutus_AMTN_Acr GQVLSSEEMPPSQVFAGVL---LPGTQG---GLLPAGQSGSLPEGQEGLLPAGQTGTNQG 216

Xenagama_zonura_AMTN_Acr GQVLSSEEMPPSQVFAGVL---LPGTQG---GLLPAGQSGSLPEGQEGLLPAGQTGTNQG 216

Laudakia_wui_Acr GQVLSSEEMPPSQVFAGVL---LPGTQG---GLLPAGQSGSLPEGQEGLLPAGQTGINQG 217

Phrynocephalus_forsythii_Acr GQVLSSEEMPPSQVFAGVL---LPGTQG---GLLPAGQSGSFPEGQEGLLPAGQTGTNQG 216

Phrynocephalus_guinanensis_Acr GQVLSSEEMPPSQVFAGVL---LPGTQG---GLLPTGQSGSFPEGQEGLLPAVQTGTNQG 216

Phrynocephalus_putjatai_Acr GQVLSSEEMPPSQVFAGVL---LPGTQG---GLLPTGQSGSFPEGQEGLLPAVQTGTNQG 216

Phrynocephalus_versicolor_Acr GQVLSSEEMPPSQVFAGVL---LPGTQG---GLLPAGQSGSFPEGQEGLLPAGQTGTNQG 216

Phrynocephalus_vlangalii_Acr GQVLSSEEMPPSQVFAGVL---LPGTQG---GLLPAGQSGSFPEGQEGLLPAGQTGTNQG 216

Agama_doriae_AMTN_Acr GQVLSSEEMPPSQVFAGVL---LPGTQG---GLLPAGQSGSLPEGQEGLLPAGQTGTSQG 216

Intellagama_lesueurii_Acr GQVLSSEEMPPSQLFAGVL---LPGTQG---GLLPAGQAGTHSEGQEGFLPAGQTGTNQG 216

Pogona_vitticeps_Acr GQVLSSEEMPPSQLFAGVL---LPGTQG---GLLPAGQAGTHSEGQEGFLPAGQTGTNQG 216

Bradypodion_pumilum_Acr GQMLSSEEMSPSQVFAGVL---LPGTQDEMKQLLPSGQTGTFPEGQEGLLPAGQTGANQG 220

Bradypodion_ventrale_Acr GQMLSSEEMSPSQVFAGVL---FPGTQDEMKQLLPSGQTXTLPEGQEGLLPAGQTGANQG 220

Furcifer_pardalis_Acr GQMLSSEEMTPSQVFAGVL---LPGTQDEIKQLLPSGQTGTFPEGQEGLLPAGQTGANHG 220

Chamaeleo_calyptratus_AMTN_Acr GQMLSSEEMSPSQVFAGVL---LPGTQDEIKQLLPSGQTGTFSEGQEVLLPAGQTGANQV 220

Chamaeleo_dilepis_AMTN_Acr GQMLSSEEMSPSQVFAGVL---LPGTQDEIKQLLPSGQTGTLSGGQEVLLPAGQTGANQV 220

Chamaeleo_gracilis_AMTN_Acr GQMLSSEEMSPSQVFAGVL---LPGTQDEIKQLLPSGQTGTLSGGQEVLLPAGQAGANQV 220

Chamaeleo_laevigatus_AMTN_Acr GQMLSSEEMSPSQVFAGVL---LPGTQDEIKQLLPSGQTGILSEGQEVLLPAGQTGANQV 220

Trioceros_affinis_AMTN_Acr XXXXXXXXXSPSQVFAGVI---LPGTQDEIKQLLPSGQTGTLPEGQEGLLPAGQTGANHG 220

Trioceros_balebicornutus_AMTN_Acr XXXXXXXXXSPPQVFAGVL---LPGTQDEIKQLLPSGQTGTLPEGQEGLLPAGQTGANHG 220

Trioceros_harennae_AMTN_Acr XXXXXXXXXSPPQVFAGVL---LPGTQDEIKQLLPSGQTGTLPEGQEGLLPAGQTGANHG 220

Anolis_apletophallus_Pleu GAVLSSEEMPTSPVMAFLL-PGLPGLPG---GLFPSGQSGALPEDQEDLLPAGQVRNNQG 227

Anolis_sagrei_ordinatus_Pleu GTVLSSEEMPTSQVMAAFL---LPGLPG---GLFPSGQSGALPEDQDDLLPAGQVGTNQG 225

Anolis_tropidonotus_AMTN_Acr GTVLSSEEMSTSQVMAAFL---LPGLPG---GLFPSGQSGALPEDQEGLLPAGQVGNNQG 225

Anolis_carolinensis_Pleu GTVLSSEEMPTSQVMAAFL---LPGLPG---GLFPSGQSGALPEDQDELLPAGQVGTNQG 227

Anolis_rodriguezii_AMTN_Pleur GAVLSSEEMPSSQVMAAFLLPGLPGLPG---GLFPSGQSGALPEEQEDLLPAGQVGNNQG 230

Ctenosaura_bakeri_Pleu GAVLSSEEMPTSQVFAAFL---IPGTQG---GLLPSGQAGTVSEGQEGLLPAGQAGNSQG 226

Iguana_delicatissima_Pleu GTVLSSEEMPTSQVFAAFL---IPGTQG---GLLPSGQAGTVSEGQEGLLPAGQAGNSQS 226

Cyclura_pinguis_Acr GAVLSSEEMPTSQVFAAFL---IPGTQG---GLLPSGQAGTVAEGQEGLLPAGQAGNGQG 226

Gambelia_wislizenii_Acr GTVLSSEEMPVSQVFAGFL---VPGMQG---GLFPSGQAGTIPEGQEEPLPAGQAGTNQG 226

Laemanctus_serratus_AMTN_Acr GTVLSSEEMPTSQMFAAFL---IPGTQG---GLFPSGQA--LPEGQEGLLPASQAGTNQG 218

Phrynosoma_blainvillii_Pleu GTVLSSEEMPTSQIFAGFL---VPGTQG---SLFPSGQAGTLPEGQEGLFPASQAGTN-- 213

Phrynosoma_platyrhinos_Pleu GTVLSSEEMPTSQIFAGFL---VPGTQG---GLFPSGQARTLPEGQEGLFPASQAGTN-- 213

Sceloparus_undulatus_Pleu GTVLSSEEMPTSQVFAGFL---VPGTQG---GLFPPGQA-SLPEGQEGLFPASQAGTN-- 212

Sceloporus_tristichus_Pleu GTVLSSEEMPTSQVFAGFL---VPGTQG---GLFPPGQA-SLPEGQEGLFPASQAGTN-- 212

Sceloporus_occidentalis_Pleu GTVLSSEEMPTSQVFAGFL---VPGTQG---GLFPPGQA-SLPEGQEGLFPASQAGTN-- 213

Sceloporus_chrysostictus_AMTN_Pleur GTVLSSEEMPPSQVFAGFL---VPGAQP---GLFPSGQAGTLPEGQEGLFPASQAGTN-- 214

Urosaurus_nigricaudus_Pleu GTVLSSEEMPTSQVFAGFL---VPGTQG---GLLPSGQAGSLPEGQEGLFPASQAGTN-- 214

. . ::* .: .** *:*.**: . *: :** *. .

Acanthocercus_cyanogaster_AMTN_Acr SLPLPEGTAAGIQKASPATSYIPNEPLGGPYPTPSGFRQPSAVNNPVFVEPTGSINLEPS 276

Acanthocercus_minutus_AMTN_Acr SLPLPEGTAAGIQKASPATSYIPNEPLGGPYPTPSGFRQPSAVNNPVFVEPTGSINMEPS 276

Xenagama_zonura_AMTN_Acr SLPLPEGTAAGIQKASPATSYIPNEPLGGPYPTPSGFRQPSAVNNPVFVEPTGSINMEPS 276

Laudakia_wui_Acr GLPLPEGTAAGIQKASPASSDSLNEPMGRPYPTPSGFRQPSAVTNAVFVEPTGSINMEPS 277

Phrynocephalus_forsythii_Acr GLPLPEGTAAGIQKASPATSDLLNDPMGGPYPTPSGFRKPSAVTNAVFVEPTGSINMEPS 276

Phrynocephalus_guinanensis_Acr GLPLPEGTAAGIQKASPATSDILNDPMGGPYPTLSVFRKPSAVTNAVFVEPTGSINMEPS 276

Phrynocephalus_putjatai_Acr GLPLPEGTAAGIQKASPATSDILNDPMGGPYPTPSGFRKPSAVTNAVFVEPTGSINMEPS 276

Phrynocephalus_versicolor_Acr GLPLPEGTAAGIQKASPATSDSLNDPMGGPYPTPSGFRKPSAVTNAVFVEPTGSINMEPS 276

Phrynocephalus_vlangalii_Acr GLPLPEGTAAGIQKASPATSDILNDPMGGPYPTPSGFRKPSAVTNAVFVEPTGSINMEPS 276

Agama_doriae_AMTN_Acr GLPLPEDTAAGIQKASPATSNGLNEPLGGAYPTPSGFRQPSAITKTVFVEPTGSINMEPS 276

Intellagama_lesueurii_Acr ILPLPEGTAAGIQKASPATSDSLNEPVGGPYPTPSGFRQPSAVTNTVFVEPTGGINMEPS 276

Pogona_vitticeps_Acr NLPLPEGTAAGIQKASPATSDSLNEPVGGLYPTPSGFRQPSAVTNAVFVEPTGGINMEPS 276

Bradypodion_pumilum_Acr ILPLPEGTAAGIQKASPATSDGLNESLGRPCPTPSGFRQPSAATSAVFVEPTGLINMEPS 280

Bradypodion_ventrale_Acr ILPLPEGTAAGIQKASPATSNGLNESVGRPCPTPSGFRQPSAATSAVFVEPTGLINMEPS 280

Furcifer_pardalis_Acr ILPLPEGTAAGIQKASPATNDGLNESMGRPCPTPSGFRQPSAATSDVFVEPTGLINMEPS 280

Chamaeleo_calyptratus_AMTN_Acr ILPLPEGTAAGIHKASPETSNGLNESVGRPCPTPSGFRQPSVATSAVFVEPTGLINMEPS 280

Chamaeleo_dilepis_AMTN_Acr ILPLPEGTAAGIHKASPATSSGLNESVGRPCPTPSGFRQPSAATSAVFVEPTGLINMEPS 280

Chamaeleo_gracilis_AMTN_Acr ILPLPEGTAAGIHKASPATSNGLHESVGRPCPTPSGFRQPSAATSAVFVEPTGLINLEPS 280

Chamaeleo_laevigatus_AMTN_Acr ILPLPEGTAAGIHKASPATSNGLNESVGRPCPTPSGFRQPSAATSAVFVEPTGLINMEPS 280

Trioceros_affinis_AMTN_Acr ILSLPEGTAAGIQKASPAASDGLNESVVRPCPTPSGFRQPSAATSAVFVEPTGLINMEPS 280

Trioceros_balebicornutus_AMTN_Acr ILPLPEGTAAGIQKASPAASDGLNESVVRPCPTPSGFRQPSAATSAVFVEPTGLINMEPS 280

Trioceros_harennae_AMTN_Acr ILPLPEGTAAGIQKASPAASDGLNESVVRPCPTPSGFRQPSAATSAVFVEPTGLINMEPS 280

Anolis_apletophallus_Pleu NLPYPESTAAGIQKGSPTIEDGLSVAPGAFYPTPSGFRQPGVVTNEVFVEPTGGINMEPS 287

Anolis_sagrei_ordinatus_Pleu NLPYPESTAAGIQKGSPTTEEGLSVAPGAFYPTPSGFRQPGVVTNEVFVEPTGGINMEPS 285

Anolis_tropidonotus_AMTN_Acr NLPYPESTAAGIQKGSPTIEDGLSVAPGAFYPTPSGFRQPGVVTNEVFVEPTGGINMEPS 285

Anolis_carolinensis_Pleu NLPFPESTAAGIQKGSPATEDGLNVAPGAFYPTPSGFRQPGVVTNEVFVEPTGGIHMEPG 287

Anolis_rodriguezii_AMTN_Pleur NLPYPESTAAGIQKGSPTIEDGLSVAPGAFYPTPSGFRQPGVVTNEVFVEPIGGINMEPS 290

Ctenosaura_bakeri_Pleu NLPYPESTAAGIQKGSPTSGDGLSEATSISNPTPSGFRQPGVVTNEVFVEPTIDINMEPS 286

Iguana_delicatissima_Pleu NLPYPESTAAGIQKGSPTSGDGLSEATSISNPTPSGFRQPGVVTNEVFVEPTIDINMEPS 286

Cyclura_pinguis_Acr NLPYPEGTAAGIQKGSPTSGDGLSEATSISNPTPSGFRQPGVVTNEVFAEPTIDINMEPS 286

Gambelia_wislizenii_Acr NLPYPESTAAGIQKGSPTTGDGLREATSISYPSPSGFRQPGVVTNEVFIEPTVGINMEPS 286

Laemanctus_serratus_AMTN_Acr NLPHPESTAAGIQKGSPTTGDGLNEATSASYLTPSGFRQPGVVTNEVFVEPTVGINMEPS 278

Phrynosoma_blainvillii_Pleu ----PESTAAGIQKGSPTTGDGLSEATSGSYPTLSGIRQPGVVTNEVFVEPTVGINMEPS 269

Phrynosoma_platyrhinos_Pleu ----PESTAAGIHKGSPTTGDGLSEATSGSYPTLSGFRQPGVVTNEVFVEPTVGINMEPS 269

Sceloparus_undulatus_Pleu ----PENTAAGIQKGSPTTGDGLNEATSGSYPTPSGFRQPGVVTNEVFVEPTVGINMEPS 268

Sceloporus_tristichus_Pleu ----PENTVAGIQKGSPTTGDGLNEATSGSYPTPSGFRQPGVVTNEVFVEPTVGINMEPS 268

Sceloporus_occidentalis_Pleu ----PENTAAGIQKGSPTTGDGLNEATSGSYPTPSGFRQPGVVTNEVFVEPTVGINMEPS 269

Sceloporus_chrysostictus_AMTN_Pleur ----PESAAAGIQKGSPTTGDGLSEATSGSYPTPSGFRHPGVVTNEVFVEPTVGINMEPS 270

Urosaurus_nigricaudus_Pleu ----PESTAAGIQKGFPTTGDGLNEATSGSYPTPSGFRQPGVVTNGVFVEPTVGINMEPS 270

**.:.***:*. * . : * :*:*.. .. ** ** *::**.

Acanthocercus_cyanogaster_AMTN_Acr ELREPPTTLVRPDTSDNQKQPNLFTTFLRGDNYMPTTTTESKPWKAP 323

Acanthocercus_minutus_AMTN_Acr ELREPPTTLVRPDTSDNQKQPNLFPTLLRGDNYMPTTTTESKPWKAP 323

Xenagama_zonura_AMTN_Acr ELREPPTTLVRPDTSDNQKQPNLFPTLLRGDNYMPTTTTESKPWKAP 323

Laudakia_wui_Acr ELREPPTTLVRPDMSDNQKHHNVFPTLLRGDSYMPTTTTESKPWKAP 324

Phrynocephalus_forsythii_Acr ELREPPTTLVRPDTSDNQKHQKLFPTILRGDHYMATTTIKSKPWKAP 323

Phrynocephalus_guinanensis_Acr ELREPPTTLVRPDTSDNQKHQKLFPTLLRGDNYMPTTTIKSKPWKAP 323

Phrynocephalus_putjatai_Acr ELREPPTTLVRPDTRDNQKHQKLFPTLLRGDNYMPTTTIKSKPWKAP 323

Phrynocephalus_versicolor_Acr ELREPPTTLVRPDTSDNQKHQKLFPTLLRGDNYMPTTTIKSKPWKAP 323

Phrynocephalus_vlangalii_Acr ELREPPTTLVRPDTSDNQKHQKLFPTLLRGDNYMPTTTIKSKPWKAP 323

Agama_doriae_AMTN_Acr ELREPPTTLVRPDTSENQKQPNLFPTLLRGDSYMPTTTTKSKPWKAP 323

Intellagama_lesueurii_Acr ELREPPTTLVKLYKSDNQKNQNLFQAFLRGDNHMPITTTESKPWKAP 323

Pogona_vitticeps_Acr ELREPPTTLVRLNKSDNQKHQNLFQAFLRGDNHMPITTTESKPWKAP 323

Bradypodion_pumilum_Acr ELREPPTTLVRLYKGDNPKHHNLFQALLRGDTPMPRTTTQSKPWKAP 327

Bradypodion_ventrale_Acr ELREPPTTLVRLYKDDNPKHHNLFQALLRGDTPMPRTTTQSKPWKAP 327

Furcifer_pardalis_Acr ELREPPTTLVRLDKGDNRKHHNLFQALLRGDTPMPRTTTESKPWKAP 327

Chamaeleo_calyptratus_AMTN_Acr ELREPPTSLVRLDNADNRKHHNLFQALLRGDTPMPRTTTQSKPWKAP 327

Chamaeleo_dilepis_AMTN_Acr ELREPPTSLVRLDNGDNRKHHNLFQALLRGDTPMPRTTTQSKPWKAP 327

Chamaeleo_gracilis_AMTN_Acr ELREPPTSLVRLDNGDNRKHHNLFQALLRGDTPMPRTTTQSKPWKAP 327

Chamaeleo_laevigatus_AMTN_Acr ELREPPTSLVRLDNGDNRKHHNLFQALLRGDTPMPRTTTQSKPWKAP 327

Trioceros_affinis_AMTN_Acr ELREPPTTLVRLDKGDNRKHYNLFQALLRGDTPMPRTTTQSKPWKAP 327

Trioceros_balebicornutus_AMTN_Acr ELREPPTTLVRLDKGDNRKHYNLFQALLRGDTPMPRTTTQSKPWKAP 327

Trioceros_harennae_AMTN_Acr ELREPPTTLVRLDKGDNRKHYNLFQALLRGDTPMPRTTTQSKPWKAP 327

Anolis_apletophallus_Pleu ELREPPTSLVGLDKGKNQKHQNLSQSPVRGDSYMPMTTTASKPLKAP 334

Anolis_sagrei_ordinatus_Pleu ELREPPTSLVGLDNGKNQKHQNLSQLPVRGDSYMPMTTTASKPLKAP 332

Anolis_tropidonotus_AMTN_Acr ELREPPTSLVGLDKGKNQKHQNLSQSPVRGDSYMPMTTTASKPLKAP 332

Anolis_carolinensis_Pleu ELREPPTSLVGLDKGNNQKLQNLSQSPVRGDSYMPMTTTASKPLKAP 334

Anolis_rodriguezii_AMTN_Pleur ELREPPTSLVGLDKGKNEKHQNLSQSPVRGDSYMPMTTTASKPLKAP 337

Ctenosaura_bakeri_Pleu ELREPPTSPVRLYKGDNQKHQNLSQSPVRGDSHMPINTTESKPLKAP 333

Iguana_delicatissima_Pleu ELREPPTSPVRLYKGNDQKHQNLSQSPVRGDSHMPINTTESKPLKAP 333

Cyclura_pinguis_Acr ELREPPTSPVRFYKGNNQKYQNLSQSPVRGDSHMPINTTESKPLEAP 333

Gambelia_wislizenii_Acr ELREPPTTVARLDKGNNQKHQNLSQSLVRGDSHMPISTTENQPLKAP 333

Laemanctus_serratus_AMTN_Acr ELREPPTSLVKLDKGNNQKYQNLSQSPVRGDSYMPIHTTESKPLKAP 325

Phrynosoma_blainvillii_Pleu ELREPPTSLVKLDKGNNQKHQNLSQSLVRGDSHIPINTTESKPLKAP 316

Phrynosoma_platyrhinos_Pleu ELREPPTSLVKLDKGNNQKHQNLSQSPVRGDSHIPINTTESKPLKAP 316

Sceloparus_undulatus_Pleu ELREPPTSLVKLDNGNNQKHQNLSQSPVRGDSHMPINTTESKPLKAP 315

Sceloporus_tristichus_Pleu ELREPPTSLVKLDNGNNQKHQNLSQSPVRGDSHMPINTTESKPLKAP 315

Sceloporus_occidentalis_Pleu ELREPPTSLVKLDKDNNQKHQNLSQSPVRGDSHMPINTTESKPLKAP 316

Sceloporus_chrysostictus_AMTN_Pleur ELREPPTSLVKLDKGNNQKHQNLSQSPVRGDSHMPINTTESKPLKAP 317

Urosaurus_nigricaudus_Pleu ELREPPTSLVKLDKGNNQKHQNLSQSPVRGDSHMPINTTESKPLKAP 317

*******: . .: * :: :*** :. * .:* :**

**ENAM-AA-MAFFT-Iguania+Mammal – DIVERGE + PCOC ≥ 0.9**

CLUSTAL W (1.8) multiple sequence alignment (ALTER 1.3.3)

Acanthocercus_cyanogaster_ENAM_Acr MKLVILY-LCLVGTMCATSLRKRRKAGFGSKSEEMMPFGAYGFLNSPQ------------ 47

Acanthocercus_minutus_ENAM_Acr MRLIILY-LCLVGTMCATPLRKRRKAGFGSKSEEMMPFGAYGFLNSPQ------------ 47

Xenagama_zonura_ENAM_Acr MRLIILY-LCLVGTMCATPLRKRRKAGFGSKSEEMMPFGAYGFLNSPQ------------ 47

Agama_doriae_ENAM_Acr MKLILLY-LCLVGTMCATPLRKRRKAGFGSKSEEMMPFGAYGFLNSPQ------------ 47

Laudakia_wui_Acr MKLIVLY-LCLLGTLCAVPLRKRRKAGFGSKSEEMMPFGAYGFLNSPH------------ 47

Phrynocephalus_forsythii_Acr MKPIILY-LCLVGTLCAIPLRKRRKAGFGSKSEEMMPFGAFGFLNSAQ------------ 47

Phrynocephalus_guinanensis_Acr MKPIILY-LCLVGTLCAIPLRKRRKAGFGSKSEEMMPFGAFGFLNSPQ------------ 47

Phrynocephalus_putjatai_Acr MKPIILY-LCLIGTLCAIPLRKRRKAGFGSKSEEMMPFGAFGFLNSPQ------------ 47

Phrynocephalus_vlangalii_Acr MKPIILY-LCLVGTLCAIPLRKRRKAGFGSKSEEMMPFGAFGFLNSPQ------------ 47

Phrynocephalus_versicolor_Acr MKPIVLY-LCLVGTLCAVPLRKRRKAGFGSKSEEMMPFGAFGFLNSPQ------------ 47

Intellagama_lesueurii_Acr MKLIVLY-LCLVGTSCAIPLRKRRKAGFGSKSEEMMPFGAYGFLNSPQ------------ 47

Pogona_vitticeps_Acr MKLFVLY-LCLVGTSCAIPLRKRRKAGFGSKSEEMMPFGAYGFLNSPQ------------ 47

Bradypodion_pumilum_Acr MKLIILC-LCLMGTTCANPLRKRRKAGFGSKSEEMMSFGAYGMLNSPQKWRSDATLFLKI 59

Bradypodion_ventrale_Acr MKLIILC-VCLMGTTCANPLRKRRKAGFGSKSEEMMSFGAYGMLNSPQKWRSDATLFLKI 59

Chamaeleo_calyptratus_ENAM_Acr MKLIILC-LCLMGTNCANPLRKRRKAGFGSKSEEMMPFGAYGMLNTP------------- 46

Chamaeleo_dilepis_ENAM_Acr MKLIILC-LCLMGTNCANPLRKRRKAGFGSKSEEMMPFGAYGMLNTP------------- 46

Chamaeleo_gracilis_ENAM_Acr MKLIILC-LCLMGTNCANPLRKRRKAGFGSKSEEMMPFGAYGMLNTP------------- 46

Chamaeleo_laevigatus_ENAM_Acr MKLIILC-LCLMGTNCANPLRRRRKAGFGSKSEEMMPFGAYGMLNTP------------- 46

Furcifer_pardalis_Acr MKFIILC-LCLMGTTCANPLRKRRKAGFGSKSEEMMPFGAYGMLNSP------------- 46

Trioceros_affinis_ENAM_Acr MKLIILC-LCLMGTTCANPLRKRRKAGFGSKSEEMMPFGAYGMLNSP------------- 46

Trioceros_balebicornutus_ENAM_Acr MKLIILC-LCLMGTTCANPLRKRRKAGFGSKSEEMMPFGAYGMLNSP------------- 46

Trioceros_harennae_ENAM_Acr MKLIILC-LCLMGTTCANPLRKRRKAGFGSKSEEMMPFGAYGMLNSP------------- 46

Anolis_apletophallus_Pleu MKQLLLY-LCLIGTSWAVRLRKPRKAGFGSKSEEMMQFGPYGYLNSPQ------------ 47

Anolis_tropidonotus_ENAM_Pleur MKQLLLY-LCLIGTSWAVRLRKPRKAGFGSKSEEMMQFGPYGYLNSPQ------------ 47

Anolis_sagrei_ordinatus_Pleu MKQLLLY-LCLIGTSWAVRLRKPRKAGFGSKSEEMMQFGPYGYLNSPQ------------ 47

Anolis_carolinensis_Pleu MKQLLLY-LCLIGTSWAVRLRKPRKAGFGSKSEEMMQYGPFGYLNSPQ------------ 47

Ctenosaura_bakeri_Pleu MKRILLY-LCLIGTAWAVLVRKPRKAGFGSKSEEMMQFGPYGYLNSPQ------------ 47

Cyclura_pinguis_Pleu MKRILLY-LCLIGTAWAVLVRKPRKAGFGSKSEEMMQFGPYGYLNSPQ------------ 47

Iguana_delicatissima_Pleu MKQILLY-LCLIGTAWAVLVRKPRKAGFGSKSEEMMQFGPYGYLNSPQ------------ 47

Gambelia_wislizenii_Pleu MKRILLY-LCLIGTSWAVLLRKPRKAGFGSKSEEMMQFGPYGYLNSPQ------------ 47

Laemanctus_serratus_ENAM_Pleur MKRIFLC-LCLIGTSWAVLLRKPRKAGFGSKSEEMMQFGPYGYLNSPQ------------ 47

Phrynosoma_blainvillii_Pleu MMLILLC-LCLTGASWAALLRKPRKAGFGSKSEEMMQFGPYGYLNSPQ------------ 47

Phrynosoma_platyrhinos_Pleu MMRILLC-LCLTGTSWAALLRKPRKAGFGSKSEEMMQFGPYGYLNSPQ------------ 47

Sceloporus_chrysostictus_ENAM_Pleur MTRILLYLLCLIGTSWAALLRKPRKAGFGSKSEEMMQFGPYGYLNSPQ------------ 48

Urosaurus_nigricaudus_Pleu MTRIFLYLLCLIGTSWAALLRKPRKAGFGSKSEEMMQFGPYGYLNSPQ------------ 48

Sceloporus_occidentalis_Pleu MMRILLYLFCLIGTSWAALLRKPRKAGFGSKSEEMMQFGPYGYLNSPQ------------ 48

Sceloporus_tristichus_Pleu MMRILLYLFCLIGTSWAALLRKPRKAGFGSKSEEMMQFGPYGYLNSPQ------------ 48

Sceloporus_undulatus_Pleu MMRILLYLFCLIGTSWAALLRKPRKAGFGSKSEEMMQFGPYGYLNSPQ------------ 48

Canis_lupus_dingo_ENAM MKILLVF-LGLLGNSFAMPMQMPRMPGFSSKSEEMMRYGQFNFMNSPH------------ 47

Vulpes_vulpes_ENAM MKILLVF-LGLLGNSFAMPMQMPRMPGFSSKSEEMMRYGQFNFMNSPH------------ 47

Meles_meles_ENAM MKILLVF-LGLLGNSVAMPMQMPRMPGFSSKSEEMMRYGQFNFMNAPH------------ 47

Mustela_putorius_ENAM MKILLVF-LGLLGNSVAMPMQMPRMPGFSSKSEEMMRYGQFNFMNAPH------------ 47

Ursus_arctos_ENAM MKILLVF-LGLLGNSVAMPMQMPRMPGFSSKSEEMMRYGQFNFMNSPH------------ 47

Phyllostomus_hastatus_ENAM MKILLVF-LGVLGNSTAMPMQMPRMPGFSSKSEEMMRYGQFNFMNSPH------------ 47

Phacochoerus_africanus_ENAM MKILLVF-LGLLGYSAAMPMQMPRMPGFSSKSEEMMRYGHFNFMNAPH------------ 47

Homo_sapiens_ENAM MKILLVF-LGLLGNSVAMPMHMPRMPGFSSKSEEMMRYNQFNFMNGPH------------ 47

Saimiri_boliviensis_ENAM MKILLVF-LGLLGNSVAMPMHMPRMPGFSSKSEEMMRYNQFNFMNSPH------------ 47

Erinaceus_europaeus_ENAM MKTLLVF-LGLLGNCIAMPMHMPRMPGFSSKSEEMSRYNQFNFMNSPH------------ 47

Suncus_etruscus_ENAM MKMLLIF-LGLLGNSIAMPMQMPPMRGFSSKSEEMMRFNQY-FMNSPH------------ 46

Echinops_telfairi_ENAM MKILMVF-LALLGNSTAMPMQMPRMTRFSSKSEEMMRYGQFNFMNSPH------------ 47

* ..: . : * * :: *.****** :. : :* .

Acanthocercus_cyanogaster_ENAM_Acr ---LSPFTASLYGYRPNYPQLFPQQPMSPLQRPFLWQPQVPVHDVARLPSQK----PQTP 100

Acanthocercus_minutus_ENAM_Acr ---LSPFTASLYGYRPSYPQLFPQQPMPPLQRPFLWQPQVPVHDVARLPSQK----PQPP 100

Xenagama_zonura_ENAM_Acr ---LSPFTASLYGYRPSYPQLFPQQPMPPLQRPFLWQPQVPVHDVARLPSQK----PQPP 100

Agama_doriae_ENAM_Acr ---LSPFAASLYGYRPNYPQLFPQQPMAPLQRPFLWQPQVP---AARLPSQK----PQPP 97

Laudakia_wui_Acr ---LSPFAASLYGYRPNYLQLFPQQPMSPLQRPFLWQQQVPVHEVTRLPSQK----PQPP 100

Phrynocephalus_forsythii_Acr ---LLPFAASLYGYRPNYPQL--------LQRPFLWQQQVPVHEVARLPSQK----PPPP 92

Phrynocephalus_guinanensis_Acr ---LSPFAASLYGYRPNYPQL--------LQRPFLWQQQVPVHEIARLPSQK----PQPP 92

Phrynocephalus_putjatai_Acr ---LSPFAASLYGYHPNYPQL--------LQRPFLWQQQVPVHEIARLPSQK----PQPP 92

Phrynocephalus_vlangalii_Acr ---LSPFAASLYGYRPNYPQL--------LQRPFLWQQQVPVHEVARLPSQK----PQPP 92

Phrynocephalus_versicolor_Acr ---LSPFAASLYGYRPNYPQL--------LQRPFLWQQQVPVHEVARLPSQK----PQPP 92

Intellagama_lesueurii_Acr ---LSQFAASLYGYRPSYPQLFPQQPMSPLPRPFLWQQQMPVHGATRLPSQNPHRLPQQP 104

Pogona_vitticeps_Acr ---LSQLAASLYGYRPSYLQLFPQQSMPPLSRPSLWQQQMPAHEATRFPSQNPHRLPQQP 104

Bradypodion_pumilum_Acr TFLLSQFAASLYGYRPSFLQMFPQQSMTPLQRPFLWQQQMPGYEAARLSPQK----PQQP 115

Bradypodion_ventrale_Acr TFLLSQFAASLYGYRPSFLQMFPQQSMTPLQRPFLWQQQMPGYEAARLSPQK----PQQP 115

Chamaeleo_calyptratus_ENAM_Acr -----QFAASLYGYRPSFLQMFPQQSMTPLQRPFLWQQPMPGYETTRLSPQK----PQQP 97

Chamaeleo_dilepis_ENAM_Acr -----QFAASLYGYRPSFLQMFPQQSVTPLQRPFLWQQQMPGYEAARLPPQR----PPQP 97

Chamaeleo_gracilis_ENAM_Acr -----QFAASLYGYRPSFLQMFPQQSMTPLQRPFLWQQQMPGYEAARLPPQR----PPQP 97

Chamaeleo_laevigatus_ENAM_Acr -----QFAASLYGYRPSFLQMFPQQSMTPLQRPFLWQQQMPGYEAARLSPQR----PQQP 97

Furcifer_pardalis_Acr -----QFAASLYGYRPSFLQMFPQQPMTPLQRPFLWQQQMPGYGAGRLSPQR----SQQP 97

Trioceros_affinis_ENAM_Acr -----QFAASLYGYRPSFLQMFPQQSTTPLQRPFLWQQQMPGYQATRLSPQR----PQQP 97

Trioceros_balebicornutus_ENAM_Acr -----QFAASLYGYRPSFLQMFPQQSMTPLQRPFLWQQQMPGYQATRLSPQR----PQQP 97

Trioceros_harennae_ENAM_Acr -----QFAASLYGYRPSFLQMFPQQSMTPLQRPFLWQQQMPGYQDTRLSPQR----PRQP 97

Anolis_apletophallus_Pleu ---LTQLAASLYGYRSGFPQMVPQQPAFPLQRFYLWPQQMPVHQSARMPQQK----PHQT 100

Anolis_tropidonotus_ENAM_Pleur ---LTQLAASLYGYRSGFPQMVPQQPAFPLQRFYLWPQQMPVHQSAQLPQQK----PHQT 100

Anolis_sagrei_ordinatus_Pleu ---LTQLAASLYGYRSGFPQMVPQQPAFPLQRFYLWPQQMPVHQSARPPQQK----PPQT 100

Anolis_carolinensis_Pleu ---LTQLAASLYGYRSGFPQMVPQQPTFPLQRFYLWPQQMPVHQSARPPQQK----AHQT 100

Ctenosaura_bakeri_Pleu ---LTQLAASLYGYRSGFPQMVHPQPAFPLQRIYLWQQQMPVHQAAKLPQQK----PHQT 100

Cyclura_pinguis_Pleu ---LTQLAASLYGYRSGFPQMVRPQPAFPLQRIYLWQQQMPVRQAARVPQQK----PHQT 100

Iguana_delicatissima_Pleu ---LTQLAASLYGYGSGFPQMVHPQPAFPLQRIYLWQQQMPVHQAAKLPQQK----PHQT 100

Gambelia_wislizenii_Pleu ---LTQLAASLYGYRSSFPQMVPQQPTFPLQRFYLWQQQMPIHQAAQLPQQK----PHQT 100

Laemanctus_serratus_ENAM_Pleur ---LTQLAASLYGYRSGLPQMVSQQPAFPLQRFYLWQQQMPVHQATRLPQQK----PHQI 100

Phrynosoma_blainvillii_Pleu ---LTQLAASLYGYHSSFPQLVPQQPAFPLKRFYLWQQQMPGHQAARPPQQK----PHQT 100

Phrynosoma_platyrhinos_Pleu ---LTQLAASLYGYHSSFPQLVPQQPAFPLKRFYLWQQQMPGHQAARPPQQK----AHQT 100

Sceloporus_chrysostictus_ENAM_Pleur ---LTQLAASLYGYRSRVPQMVPQQAAFPLQRFYLWQQQMPGHQAARLPQRK----PHQN 101

Urosaurus_nigricaudus_Pleu ---LTQLAASLYGYRSRFPQVVPQQPAFPLQRFYLWQQQMPGHQAARLPQQK----PQQN 101

Sceloporus_occidentalis_Pleu ---LTQLAASLYGYRSGFPQMVPQQPAFPLQRFYLWQQQMPGHQAAKLPQQK----PQQN 101

Sceloporus_tristichus_Pleu ---LTQLAASLYGYRSGFPQMVPQQPAFPLQRFYLWQQQMPGHQAAKLPQQK----PQQN 101

Sceloporus_undulatus_Pleu ---LTQLAASLYGYRSGFPQMVPQQPAFNLQRFYLWQQQMPGHQAAKLPQQK----PQQN 101

Canis_lupus_dingo_ENAM ---MAQL-GPLYGNS--MPQLFPQYQMP------MWPQPPPNTGH----PQK------SP 85

Vulpes_vulpes_ENAM ---MAQL-GPLYGNS--MPQLFPQYQMP------MWPQPPPNTGH----PQK------SP 85

Meles_meles_ENAM ---MAQL-GPLYGNG--MPQLFPQYQMP------MWPQPPPNTGH----PQK----PSSP 87

Mustela_putorius_ENAM ---MAQL-GPLYGNG--MPQFLPQYQMP------MWPQPPPNTGH----PQK----PSSP 87

Ursus_arctos_ENAM ---MAQL-GPLYGNG--MPPVFPQYQMP------MWPQPPPNMGH----LQK----SSPP 87

Phyllostomus_hastatus_ENAM ---LGQL-GPLYGYGAQIPQLFPQYQMP------MWPQPPPKTGR----PRK----PSSP 89

Phacochoerus_africanus_ENAM ---MAHL-GTLYGNGMQLPQFFPQYQMP------MWPQPPPNKKH----PQK-------P 86

Homo_sapiens_ENAM ---MAHL-GPFFGNG--LPQQFPQYQMP------MWPQPPPNTWH----PRK-------S 84

Saimiri_boliviensis_ENAM ---MAHL-GPFFGNGLQLPQQFPQYQMP------MWPHPPPNTLN----PQK-------P 86

Erinaceus_europaeus_ENAM ---MPHL-GPTFGNGMQMPPVMPPYQMP------MWPQPPPNAKH----PKK-------P 86

Suncus_etruscus_ENAM ---MAHL-GPMYGNGM---PLFPQYQMP------MWPQSPPNMGL----PPK----TSTP 85

Echinops_telfairi_ENAM ---MSQL-GPMYGNGLQNPQLFPQYQMP------MWPQPPP-PWH----PRK----PSSP 88

: .. :* :* * .

Acanthocercus_cyanogaster_ENAM_Acr SVAWKPNSRPQPK----PQQP---------RHQVQQPQVQV-QQP-PKPPPGAAGPFQPQ 145

Acanthocercus_minutus_ENAM_Acr SVAWKPNGRPQPK----PQQP---------RHQVQQPQVQV-QQP-PKPPPGAAGPFQPQ 145

Xenagama_zonura_ENAM_Acr SVAWKPNGRPQPK----PQQP---------RHQVQQPQVQV-QQP-PKPPPGAAGPFQPQ 145

Agama_doriae_ENAM_Acr SVAWKPNGRPQLK----PQQP---------RHQVLQPQVQV-QQP-PKLPPGAAGPYQPQ 142

Laudakia_wui_Acr SAAWKPNSRPQPKPQLPPQQP---------RHQVQQPQIQV-QQL-PKLPPNAASPLPPQ 149

Phrynocephalus_forsythii_Acr SAAWKPNSRPQPKPPLPPQQP---------RHQVQQPQIQV-QQP-PKLPPRAAGPSQPQ 141

Phrynocephalus_guinanensis_Acr SAAWKPNSRPQPKLPLPPQQP---------RHQVQQPQIQV-QQP-PKLPPGAAGPSQPQ 141

Phrynocephalus_putjatai_Acr SAAWKPNSRPQPKLPLPPQQP---------RHQVQQPQIQV-QQP-PKLPPGAAGPSQPQ 141

Phrynocephalus_vlangalii_Acr SAAWKPNSRPQPKPPLPPQQP---------RHQVQQPQIQV-QQP-PKLPPGAASPSQPQ 141

Phrynocephalus_versicolor_Acr SVAWKPNSRPQPKPPLPPQQP---------RHQVQQPQIQV-QQP-PKLSPGAATPSHPQ 141

Intellagama_lesueurii_Acr SAARRPNSRPQPKPQLPPQQP---------RHQVQQPQIKL-QQP-PKLPPSAIDPSQPQ 153

Pogona_vitticeps_Acr SAARRPNSRPQPKLQLPPQQP---------THQVQQPQIKV-QQP-PKLPPSAADPSQPQ 153

Bradypodion_pumilum_Acr PAAKRPNGR---------QQP---------RHQVLQQQPKV-LQP-PKQPPRVSSLPQPQ 155

Bradypodion_ventrale_Acr PAAKRPNGQ---------QQL---------RHQVLQQQPKV-LQP-PKQPPRVSSLPQPQ 155

Chamaeleo_calyptratus_ENAM_Acr PAAKKPNGR---------QQP---------RHQVLQQQPKV-LQP-PKQPPRVSSLPQPQ 137

Chamaeleo_dilepis_ENAM_Acr PAAKRPNGR---------QQP---------RHQVLQQQPKVLLQP-PKQPPRVSSLPQPQ 138

Chamaeleo_gracilis_ENAM_Acr PAAKRPNGR---------QPP---------RHQVLQQQPKV-LQP-PKQPPRVSSLPQPQ 137

Chamaeleo_laevigatus_ENAM_Acr PAAKRPNGR---------QQP---------RHQVLQQQPKV-LQP-PKQPPRVSSL--PQ 135

Furcifer_pardalis_Acr SAAKRPNGR---------QQP---------RHQVLQPQAKV-LQP-PKQPPRVNNLPQPQ 137

Trioceros_affinis_ENAM_Acr PAAKRPTGQ---------QQP---------RHQVLHQQPKV-VQP-PKQPPRFNSLPQPQ 137

Trioceros_balebicornutus_ENAM_Acr PAAKRPNGQ---------QQP---------RHQVLQQQPKV-LQP-PKQPPRVSSLPQPQ 137

Trioceros_harennae_ENAM_Acr PAAKRPNGQ---------QQP---------RHQVLQQQPKV-LQP-PKQPPRVSSLPQPQ 137

Anolis_apletophallus_Pleu PVSKKPNSRPQQKPRLAPQQP---------RYQ--QPQPKT-HLP-AKQPPVASHPFQPQ 147

Anolis_tropidonotus_ENAM_Pleur PVSKKPNSRPQQKPRLAPQQP---------RYQ--QPQPKI-HLP-SKQPPVATHPFQPQ 147

Anolis_sagrei_ordinatus_Pleu PVSKKANSRPQQKPRLAPQQP---------RYQ--QPQPKT-HLP-AKQPPVATQPFQPQ 147

Anolis_carolinensis_Pleu PVPKKPNSRPRLKPRLVPQQP---------RYQ--QPQPKI-HLP-AKQPPVATHPFQPQ 147

Ctenosaura_bakeri_Pleu PVSKKANSRPQPKPRLTPQQP---------RHQ--QPQPKI-QPP-RRQPQSTTSPSQPH 147

Cyclura_pinguis_Pleu PVSKKANSRPQPKPRLTPQQP---------RHQ--QPQPKI-QPP-PRQPQSTTSPSQPH 147

Iguana_delicatissima_Pleu PVSKKANSRPQPKPHLTPQQP---------RHQ--QPQPKI-QPP-PRQPQSTTSPSQPH 147

Gambelia_wislizenii_Pleu PVPKKSNSRPQPKPQLIPQQP---------RYQ--QPQPKI-HQP-PKQPQITTSPSQPH 147

Laemanctus_serratus_ENAM_Pleur PASKKPNSRPQPKPRLVPQQP---------RHQ--QPQPKI-PLP-PKQPQMTINPSQPH 147

Phrynosoma_blainvillii_Pleu PVPKKPNNRLQPKPQLTIKQLQPKIQQPQTRIP--QPQPKI-QLP-PKQPQTPTDPSETH 156

Phrynosoma_platyrhinos_Pleu PVPKNPNNRLQPKPQLTIKQLQPKIQQPQTRIP--QPKPKI-QLP-PKQPQTPTNPSETH 156

Sceloporus_chrysostictus_ENAM_Pleur PVP-----RLLPKPRLALQQPQPRIPQPQSRIP--QPQPKL-QLP-PKQPQTPTYPSQPY 152

Urosaurus_nigricaudus_Pleu PVPRKPNSWLQPKPRLTLQQPQPKIQQPQARIL--QPQPKI-QLA-PKQPQIPTYPSQPH 157

Sceloporus_occidentalis_Pleu PVPRKPNTRLPLKPQLTLQQPQPKIPQPQPRIP--QAQSKI-QLP-PKQPQTPTYPFQPH 157

Sceloporus_tristichus_Pleu PVPRKPNTRLPPKPRLTLQQPQPKIPQPQPRIP--QAQSKI-QLP-PKQPQTPTYPFQPH 157

Sceloporus_undulatus_Pleu PVPRKPNTRLPPKPRLTLQQPQPKIPQPQPRIP--QAQSKI-QLP-PKQPQTPTYPFQPH 157

Canis_lupus_dingo_ENAM SALKRQKSKTDQAPE--TQKPN-------------QPQPK--KPP-PKRPLKQPPPTPAQ 127

Vulpes_vulpes_ENAM SALKRQKSKTDQAPE--TQKP--------------QPQQK--KPP-PKRPLKQPPPTPAQ 126

Meles_meles_ENAM SAPRRQKSKTDPVPE--TQKPS-------------QPQPK--KPP-PKQPLKQPPPTPAQ 129

Mustela_putorius_ENAM SAPKRQKSKTDQVPE--TQKPS-------------QPQPK--KPP-PKRPLKQPPPTPAQ 129

Ursus_arctos_ENAM SAPKRQKSKTDQIPE--TQNPN-------------EPQPK--KPP-PKQPLKQPPPTPAQ 129

Phyllostomus_hastatus_ENAM SAPKHQ-SKTDQVPE--TQKPN-------------QPQPK--KPP-PKRPLKQPTPAPTP 130

Phacochoerus_africanus_ENAM SASKQQ-SKTDPAPE--SQKPN-------------QPQPK--TPT-PKQPLNEPSPTPTQ 127

Homo_sapiens_ENAM SAPKRH-NKTDQTQE--TQKPN-------------QTQSK--KPP-QKRPLKQPSHNQPQ 125

Saimiri_boliviensis_ENAM SGPKRH-NKTDQTQE--TQKPD-------------QTQSK--KPP-QKRPLKQPSHTPAQ 127

Erinaceus_europaeus_ENAM PSPKRP-GKTEQTPE--TQKPI-------------QTEPQ--KPPAPKQPENQPAPTPTQ 128

Suncus_etruscus_ENAM PT-----PKTDQTQE--TPKPT-------------QSPPK--KTP-PKQPAQQSPTTPTQ 122

Echinops_telfairi_ENAM SGSKRQ-SKTDQTQE--TQKPT-------------QPQPK--KPP-PKRPLKQSPQATPQ 129

. . : : . .

Acanthocercus_cyanogaster_ENAM_Acr PQGPALQPKGGKPQN--PQAFLP-------------HQQQPWHFPQMFGHGGLHPQMFGP 190

Acanthocercus_minutus_ENAM_Acr PQGPALQPKGGKPQN--PQAFPP-------------HQQQPWHFPQMFGHGGLHPQMFGP 190

Xenagama_zonura_ENAM_Acr PQGPALQPKGGKPQN--PQAFPP-------------HQQQPWHFPQMFGHGGLHPQMFGP 190

Agama_doriae_ENAM_Acr PQGPALQPKGGKPQN--PQAFLP-------------HQQQPWHFPQMFGHGGLHPQMFGP 187

Laudakia_wui_Acr PQGPALQPKGGKPQH--PQAFLP-------------HQQQPWHFPQMFGHGGVHPQLFGP 194

Phrynocephalus_forsythii_Acr PHGTAQQPKEGKSQH--PQAFLP-------------HQQQPWHFPQIFGHGGLHPQLFGP 186

Phrynocephalus_guinanensis_Acr PHGTAQQPKGGKSQH--PQAFLP-------------HQQQPWHFPQIIGHGGLHPQLFGP 186

Phrynocephalus_putjatai_Acr PHGTAQQPKGGKSQH--PQICLP-------------HQQQPWHFPQIFGHGGLHPQLLGP 186

Phrynocephalus_vlangalii_Acr PHGTAQQPKGGKSQH--LQAFLP-------------HQQQPWHFPQIFGHGGFHPQLFGP 186

Phrynocephalus_versicolor_Acr PHGPALQPKGGKSQH--PQAFLP-------------HQQQPWHFPQIFGHGGLHPQLFGP 186

Intellagama_lesueurii_Acr PQVPDLQSK----------AFLP-------------HQQQPWHFPQMFGHGGFQPQMFGP 190

Pogona_vitticeps_Acr PQLPDLQSK----------AFLP-------------HQQQPWHFPQMFGHGGFQPQMFGP 190

Bradypodion_pumilum_Acr PPVP---------------AFLP-------------HQQHPWHFPQMFGNGGFHPQPFGP 187

Bradypodion_ventrale_Acr PPVP---------------AFLP-------------HQQHPWHFPQMFGNGGFHPQPFGP 187

Chamaeleo_calyptratus_ENAM_Acr PQVP---------------AFLP-------------HQQQPWHFPQMFGHGGFHRQPFGP 169

Chamaeleo_dilepis_ENAM_Acr PQVP---------------AFLP-------------HQQQPWHFPQMFGHGGFHPQPFGP 170

Chamaeleo_gracilis_ENAM_Acr PQVP---------------AFLP-------------HQQQPWHFPQMFGHGGFHPQPFGP 169

Chamaeleo_laevigatus_ENAM_Acr PQVP---------------AFLP-------------HQQQPWHFPQMFGHGGFHPQPFGP 167

Furcifer_pardalis_Acr PQVP---------------AFLP-------------HQQQPWHFPQMFGHGGFHPQPFGP 169

Trioceros_affinis_ENAM_Acr PQAP---------------AFLP-------------HQQQPWHFSQXXXXXXXXXXXXXX 169

Trioceros_balebicornutus_ENAM_Acr PQAP---------------XXXX-------------XXXXXXXXXXMFGHGGFHPQPFGP 169

Trioceros_harennae_ENAM_Acr PQAS---------------XXXX-------------XXXXXXXXXXMFGHEGFHPQPFGP 169

Anolis_apletophallus_Pleu PQVPVQPPKGEKPQQ--PQAFPP-------------HIQQPWQYPQIFGNGAFQPQLFNP 192

Anolis_tropidonotus_ENAM_Pleur PQVPVQPPKAEKPQQ--PQAFPP-------------HIQQPWQYPQIFGNGAFQPQLFNP 192

Anolis_sagrei_ordinatus_Pleu PQVPVQPPKGEKPQQ--PQAFPP-------------HTQQPWQFPQIFSHGAFQPQLFNP 192

Anolis_carolinensis_Pleu PQTPIQPPKGEKPQQ--PQAFPP-------------HPQQPWQFPQIFGHGGFQPQLFNP 192

Ctenosaura_bakeri_Pleu PHGPVQQPKGEKPQQ--PQAFPP-------------HQQPPWHFPQIFGHGGFQPPLFSP 192

Cyclura_pinguis_Pleu PHGPVQQPKGEKPQQ--PQAFPP-------------HQQPPWHFPQIFGHGGFQPPLFSP 192

Iguana_delicatissima_Pleu PHGPVQQPNGEKPQP--PQAFPP-------------HQQPPWHFPQIFGHGGFQPPLFSP 192

Gambelia_wislizenii_Pleu PRVPVQQPKGEKQQQ--PQAFPP-------------HQHQPWHFPQIFGHGGFQPQLFSP 192

Laemanctus_serratus_ENAM_Pleur PHVPVQQPKGEKQQQ--PQAFPP-------------HQQQPWHFPQIFGHGGFQPQLFSP 192

Phrynosoma_blainvillii_Pleu PHVPVQQPKEETQT----QAFSP-------------HQQQPWNFPQIFGHGSFQPQLFSP 199

Phrynosoma_platyrhinos_Pleu PHVPVQQPKEEMQTQLNKQAFSP--------------QQQPWNFPQIFGHGGFQPQLFSP 202

Sceloporus_chrysostictus_ENAM_Pleur PHVPVQQPKGEKQQQ--PQGFPP-------------HQQQPWHIPQIFGNGGFQPQLFSP 197

Urosaurus_nigricaudus_Pleu PHVPVQQPKGEKQQQ--TQGFPP-------------HQQQPWHIPQIFGHGGFQPQLFSP 202

Sceloporus_occidentalis_Pleu PHVPVQQPKGEKQQQ--TQGFPP-------------HQQQPWHIPQIFGSGGFQPQMFSP 202

Sceloporus_tristichus_Pleu PHVPVQQPKGEKQQQ--TQGFPP-------------HQQQPWHIPQIFGHGGFQPQLFSP 202

Sceloporus_undulatus_Pleu PHVPVQQPKGEKQQQ--TQGFPP-------------HQQQPWHIPQIFGHGGFQPQLFSP 202

Canis_lupus_dingo_ENAM PE--------EEGQP--PQAFPPFGNGLF------LYQQPPWQVPH-------------- 157

Vulpes_vulpes_ENAM PE--------EEGQP--PQAFPPFGNGLF------LYQQPPWQVPH-------------- 156

Meles_meles_ENAM PE--------EETQP--PQAFPPFGNGLFPPFGNGLYQQPPWQGPH-------------- 165

Mustela_putorius_ENAM PE--------EETQP--PQAFPPFGNGLFPPFSNGLYQQPPWQVPH-------------- 165

Ursus_arctos_ENAM PE--------EETQP--PQAFPPFGNGLFPPFSNGLYQQPPWQVPH-------------- 165

Phyllostomus_hastatus_ENAM PQ--------EEAQP--PQPFPPFGNGIFP------YQQPPWQIPH-------------- 160

Phacochoerus_africanus_ENAM PE--------EETQT--PQAFPPFGNGLFP------YQQPLWHVPH-------------- 157

Homo_sapiens_ENAM PE--------EEAQP--PQAFPPFGNGLFP------YQQPPWQIPQ-------------- 155

Saimiri_boliviensis_ENAM PK--------EEDQP--PQAFPPFGNGLFP------YQQPPWQIPQ-------------- 157

Erinaceus_europaeus_ENAM PQ--------EEVQT--PQAFPPFGNGLYP------YQQPPWQLPP-------------- 158

Suncus_etruscus_ENAM PQ--------EEVPP--HQAYPPFGNGLYP------F-QPPWPVPH-------------- 151

Echinops_telfairi_ENAM PT--------EEAPT--PQAFPPFNNGLFP------YQQPPWQIPQ-------------- 159

*

Acanthocercus_cyanogaster_ENAM_Acr YQGRT-PLGRPLGRPHISNEE-GAPYFGY-GFHGMGPRPA-YSEEMFEQDFEEPVEKEPP 246

Acanthocercus_minutus_ENAM_Acr YQGRT-PLGRPLGRPHISNEE-GAPYFGY-GFHGMGPRPA-YSEEMFEQDFEEPVEKEPP 246

Xenagama_zonura_ENAM_Acr YQGRT-PLGRPLGRPHISNEE-GAPYFGY-GFHGMGPRPA-YSEEMFEQDFEEPVEKEPP 246

Agama_doriae_ENAM_Acr YQGRT-PLGRPLGRPHISNEE-GAPYFGY-GFHGMGPRAA-YSEEMFEQDFEEPVEKEPP 243

Laudakia_wui_Acr YQGRT-PLGRPLGRPHVSNEE-GAPYFGY-GFHGMGPRPA-YSEEMFEQDFEEPVEKEPP 250

Phrynocephalus_forsythii_Acr YQGRT-PLGRPLGRPHVSNEE-GAPYFGY-GFHGMGTRPA-YSEEMFEQDFEEPVEKEPP 242

Phrynocephalus_guinanensis_Acr YQGRT-PLGRPLGRPHVSNEE-GAPYFGY-GFHGMGTRPA-YSEEMFEQDFEEPVEKEPP 242

Phrynocephalus_putjatai_Acr YQGRT-PLGRPLGRPHVSNEE-GAPYFGY-GFHGMGTRPA-YSEEMFEQDFEEPVEKEPP 242

Phrynocephalus_vlangalii_Acr YQGRT-PLGRPLGRPHVSNEE-GAPYFGY-GFHGMGTRPA-YSEEMFEQDFEEPVEKEPP 242

Phrynocephalus_versicolor_Acr YQGRT-PLGRPLGRPHVSNEE-GAPYFGY-GFHGMGTRPA-YSEEMFEQDFEEPVEKEPP 242

Intellagama_lesueurii_Acr YQGRT-PLGRPLGRPHVSNEE-GAPYFGY-GYHGMGHRPP-YSEEMFEQDFEEPVEKEPA 246

Pogona_vitticeps_Acr YQGRT-PLGRPLGRPHVSNEE-GAPYFGY-GYHGMGHRPP-YSEEMFEQDFEEPVEKEPA 246

Bradypodion_pumilum_Acr YQGRT-----PRGRPHVSNEE-GMPYFGY-GYHGMGGRAP-YSEEMFEQDFEEPVEKEPP 239

Bradypodion_ventrale_Acr YQGRT-----PRGRPHVSNEE-GMPYFGY-GYHGMGGRAP-YSEEMFEQDFEEPVEKEPP 239

Chamaeleo_calyptratus_ENAM_Acr YHGHT-----PRGRPHVSNEE-GIPYFGY-GYHGMGGRAP-YSEEMFEQXFEEPVEKEPX 221

Chamaeleo_dilepis_ENAM_Acr YQGRT-----PRGRPHVSNEE-GMPYFGY-GYQGMGGRAP-YSEEMFEQDFEEPVEKEPP 222

Chamaeleo_gracilis_ENAM_Acr YQGRT-----PRGRPHMSNEE-GMPYFGY-GYQGMGGRAP-YSEEMFEQDFEEPVEKEPP 221

Chamaeleo_laevigatus_ENAM_Acr YQGRT-----PRGRPHVSNEE-GMPYFGY-GYQGMGGRVP-YSEEMFEQDFEEPVEKEPP 219

Furcifer_pardalis_Acr YQGRT-----PRGRPHVSNEE-GMPYFGY-GYHGIGGRAP-YSEEMFEQDFEEPVEKEPP 221

Trioceros_affinis_ENAM_Acr XXXRT-----PRGRPHVSNEE-GMPYFGY-GYHGMGGRAP-YSEEMFEQDFEEPVEKEPP 221

Trioceros_balebicornutus_ENAM_Acr YQGRT-----PRGRPHVSNEE-GMPYFGY-GYHGMGGRAP-YSEEMFEQDFEEPVEKETP 221

Trioceros_harennae_ENAM_Acr YQGRT-----PRGRPHVSNEE-GMPYFGY-GYHGMGGRAP-YSEEMFEQDFEEPVEKEPP 221

Anolis_apletophallus_Pleu YQGRM-----PYGRPP-NSEE-GTPYFGP-GYQGMGGRPPYYSEEMFEQE-----DKEPT 239

Anolis_tropidonotus_ENAM_Pleur YQGRM-----PFGRPP-NSEE-GTPYFGP-GYQGMGGRPPYYSEEMFEQE-----DKEPT 239

Anolis_sagrei_ordinatus_Pleu YQGRM-----PFGRPP-NSEE-GTPYFGP-GYQGMGGRPPYYSEEMFEQE-----DKEPT 239

Anolis_carolinensis_Pleu YQGRM-----PFGRPP-NSEE-GNPYFGP-GYQGMGGRPPYYSEEMFEQE-----DKEAP 239

Ctenosaura_bakeri_Pleu YQGRM-----PFGQPPVSNEE-GTPYFGY-GYQGMGGRPPYYSEEMFEQDFEKPKEKEAP 245

Cyclura_pinguis_Pleu YQGHM-----PFGRPPVSNEE-GTPYFGY-GYQGMGGRPPYYSEEMFEQDFEKPKEKEAP 245

Iguana_delicatissima_Pleu YQGRM-----PFGRPPVSNEE-GTPYFGY-GYQGMGGRPPYYSEEMFEQDFEKPKEKEAP 245

Gambelia_wislizenii_Pleu YQGRM-----PLGRPPISNEE-GTPYFGY-GYQGMGGRPPYYSEEMFEQDFEKPKEEEAP 245

Laemanctus_serratus_ENAM_Pleur YQGRM-----PFGRPPISNEE-GTPYFGY-GYQGMGGRPPYYSEEMFEQDFEKPKEKEAP 245

Phrynosoma_blainvillii_Pleu YQGHM-----PFGWPPTSKEE-GTPYFGY-GYQGMGGRPPYYSEEMFEQDFEEPKEKETT 252

Phrynosoma_platyrhinos_Pleu YQGHM-----PFGRPTTSKEE-GTPYFGY-GYQGMGGRPPYYSEEMFEQDFEEPKEKETT 255

Sceloporus_chrysostictus_ENAM_Pleur YQGRM-----PFGRPPISNEE-GTPYFGY-GYQGMGGRPPYYSEEMFEQDIEEPIEKEAP 250

Urosaurus_nigricaudus_Pleu YQGRM-----PFGRPPISNEE-GTPYFGY-GYQGMGGRPPYYSEEMFEQDIEEPVEKEAP 255

Sceloporus_occidentalis_Pleu YQRRM-----PFGRPPISNEE-GTPYFGY-GYQGMGGRPPYYSEEMFEQDIEEPVEKEAP 255

Sceloporus_tristichus_Pleu YQRRV-----PFGRPPVSNEE-GTPYFGY-GYQGMGGRPPYYSEEMFEQDIEEPVEKEAP 255

Sceloporus_undulatus_Pleu YQRRM-----PFGRPPVSNEE-GTPYFGY-GYQGMGGRPPYYSEEMFEQDIEEPVEKEAP 255

Canis_lupus_dingo_ENAM ---RV-PP--GYGRPPASNEEGGNPYFGYFGYQGFGGRPPYYSEEMFEQDFEKPKEKDPP 211

Vulpes_vulpes_ENAM ---RV-PP--GYGRPPVSNEEGGNPYFGYFGYQGFGGRPPYYSEEMFEQDFEKPKEKDPP 210

Meles_meles_ENAM ---KV-PP--GYGRPPVSNEEGGNPYFGYFGYQGFGGRPPYYSEEMFEQDFEKPKEEDPP 219

Mustela_putorius_ENAM ---RV-PP--GYGRPPVSNEEGGNPYFGYFGYQGFGGRPPYYSEEMFEQDFEKPKEEDPP 219

Ursus_arctos_ENAM ---RV-PP--GYGRPPGSNEEGGNPYFGYFGYQGFGGRPPYYSEEMFEQDFEKPKEEDPP 219

Phyllostomus_hastatus_ENAM ---RI-PP--GYGRPPVSNEEGGNPYFGYFGYQGFGGRPPYYSEEMFEQDFEKPKEEDPP 214

Phacochoerus_africanus_ENAM ---RI-PP--GYGRPPTSNEEGGNPYFGFFGYHGFGGRPPYYSEEMFEQDFEKPKEKDPP 211

Homo_sapiens_ENAM ---RLPPP--GYGRPPISNEEGGNPYFGYFGYHGFGGRPPYYSEEMFEQDFEKPKEEDPP 210

Saimiri_boliviensis_ENAM ---RVPPP--GYGRPPISNEEGGNPYFGYFGYHGFGGRPPYYSEEMFEQDFEKPKEEDPP 212

Erinaceus_europaeus_ENAM ---RI-PP--GFGRPPGSNEEGGNPYFGYFGFHGFGGRPPYYSEEMFEQDFEKPKEKDPP 212

Suncus_etruscus_ENAM ---RM-PP--GYGRPPTSNEDGGNPYFGYFGFHGFGGRPPYYSEEMFEQDFEKPKEKDPP 205

Echinops_telfairi_ENAM ---RV-PP--GYGRPPISNEEGGNPYFGYFGYHGFGGRPPYYSEEMFEQDFEKPKEKDPP 213

: * * ..*: * **** *::*:* * . ******** :::.

Acanthocercus_cyanogaster_ENAM_Acr K-ETPATSPVTNSTVPDTNTTVSNPASQGGNATISGASATGNGVNPLGLQSKYVEGNGFS 305

Acanthocercus_minutus_ENAM_Acr K-ETPATSPVTNSTVPDTNTTVSNPASQGGNATISGASATGNGANPLGLQSKFVDGNGFS 305

Xenagama_zonura_ENAM_Acr K-ETPATSPVTNSTVPDTNTTVSNPASQGGNATISGASATGNGANPLGLQSKFVDGNGFS 305

Agama_doriae_ENAM_Acr K-ETPATSPVTNSTVPDTNATVPNPANQGGNATISGASATGNGANPLGLQIKHADGNGFS 302

Laudakia_wui_Acr K-ETPATSPVTNSTAADTNATVSNPASQGGNATISGASTTGNGANPLGLQSKFVEGNGFS 309

Phrynocephalus_forsythii_Acr K-ETPASSPVTNSTVPDTNTTVPNPASQGENATLSGASVTGNGANPMGLQSKFIDGNGFS 301

Phrynocephalus_guinanensis_Acr K-ETPASSPVTNSTVPDTNTTVPNPASQGENATLSGASATGNGANPMGLQSKFVGGNGFS 301

Phrynocephalus_putjatai_Acr K-ETPASSPVTNSTVPDTNTTVPNPASQGENATLSGASATGNGANPMGLQSKFVGGNGFS 301

Phrynocephalus_vlangalii_Acr K-ETPASSPVTNSTVPDTNTTVPNPASQGENATLSGASATGNGANPMGLQSKFVDGNGFS 301

Phrynocephalus_versicolor_Acr K-ETPASSPVTNSTVPDTNATVPNPASQGENATLSGTSATGNGANPLGLQGKFVDGNGFS 301

Intellagama_lesueurii_Acr K-ETPATNPVTNSTVSDTNTTVSNPASQGGNATISEPIATGNGANPLGLQSKLVDGNGFS 305

Pogona_vitticeps_Acr K-ETPATNPVTNSTVADTNTTVSNPASQGGNATISEPIATGNGANPLGLQSKLVDGNGFS 305

Bradypodion_pumilum_Acr K-ETPV----TNSTVPDTNTTVSNPANQGGNATISGPSVTGTAVNSLGLQSKLVDGNEIS 294

Bradypodion_ventrale_Acr K-ETPV----TNSTVPDTNTTVSNPANQGGNATISGTSVTGTAANSLGLQSKLVDGNEIS 294

Chamaeleo_calyptratus_ENAM_Acr K-ETPA----TNSTVPDTNTTVSNSANQGGNATISGPSLTGNGANSLGLQSKLVDGNGLS 276

Chamaeleo_dilepis_ENAM_Acr K-ETPA----TNSTVPDTNTTVSNPANQGGNATISGPSVTGNGANSLGLQSKSIDGNGLS 277

Chamaeleo_gracilis_ENAM_Acr K-ETPA----TNSTVPDTNTTVSNPANQGGNATISGPSVTGNGANSLGLQSKSIVGNGLS 276

Chamaeleo_laevigatus_ENAM_Acr K-ETPS----TNSTVPDTNTTVSNPANQGGNATISGSSVTGNGANSLGLQSKLIDGNVLS 274

Furcifer_pardalis_Acr K-ETPT----NNSTVPDTNTTVSNPANQGGNATISGPSVTGNGANSLGLQSKLVDGNGLS 276

Trioceros_affinis_ENAM_Acr K-ETPA----TNSTVPDTNTTASNPANQGGNVTISGPSVTGNGANSLGLQSKLVDGNVLS 276

Trioceros_balebicornutus_ENAM_Acr K-ETPA----TNSTVPDTNTTVSNPANQGRNVTISGPSVTGNGANSLGLQSKLVDGNVLS 276

Trioceros_harennae_ENAM_Acr K-ETPA----TNSTVPDTNTTVSNPANQGGNVTISGPSVTGNGANSLGLQSKLVDGNVLS 276

Anolis_apletophallus_Pleu K-ETPATDPVSNST--DTNSTISNPAGQAGNGTISGLGEKATGANSPSLQNNLVSGNAAS 296

Anolis_tropidonotus_ENAM_Pleur K-ESPATDPVSNST--DTNSTISNPASQAGNGTISGLGEKATGANSPSLQNNLVSGNAAS 296

Anolis_sagrei_ordinatus_Pleu K-ESPATDPVTNST--DTNSTISNPASQAGNGTISGLGEKAIGANSPGLQNNLVSGNAAS 296

Anolis_carolinensis_Pleu K-ESPATDPVTNST--DTNSTVSNPASQGGNGTMSGLSEKATGANSPGLQNNMFSGNAVS 296

Ctenosaura_bakeri_Pleu K-ESPSTDPVTNTTASDTNSTSSNPASQAGNGTISSPSEKATGSSSPGLQNKLVSGNEVS 304

Cyclura_pinguis_Pleu K-ESPSTDPVTNTTASDTNSTSSNPASQAGNGTISSPSEKATGPSSPGLQNKLVSGNEVS 304

Iguana_delicatissima_Pleu K-ESPSTDPVTNTTASDTNSTSSNPASQGGNGTISSPGEKATGPSSQGLQNKPVSGNEIS 304

Gambelia_wislizenii_Pleu K-ESPATDPITNTTVSDTNSTVSNPISQGGNGTISRLGEKATGANSLGIQNKLVSGNGVP 304

Laemanctus_serratus_ENAM_Pleur K-ESPATDPVTNSTASDTNSTISNPASQGGNGTISGLAEKATGPSSPGLQNKLVSGNGVP 304

Phrynosoma_blainvillii_Pleu K-ESPATDPVTNSTVSDTNSTISNPASQGGNDTVSGLGEKVTRASSPGLQNKLVSGNGAS 311

Phrynosoma_platyrhinos_Pleu K-ESPATDPVTNSTVSDTNSTISNPASQGGNDTVSGLGVKVTRASSPGLQNILVSGNGAS 314

Sceloporus_chrysostictus_ENAM_Pleur K-ESPATDPVTNSTVSDTNSTISNPSSQVGNATISGLGGKVTGAISPSLQNNLVSGNRAS 309

Urosaurus_nigricaudus_Pleu K-ESPATDPVTNSTVSDTNSTISNPVSQVGNDTISGLGGKVTGASSPSLQNKLVSGNRAS 314

Sceloporus_occidentalis_Pleu K-ESPATDPVTNSTVSDTNSTISNPAGQVGNDTISGLGGKVTGASSPSLQNKLVSGNRAS 314

Sceloporus_tristichus_Pleu K-ESPATDPVTNSTVSDTNSTISNPAGQVGNDTISGLGGKVTGASSPSLQNKLVSGNRAS 314

Sceloporus_undulatus_Pleu K-ESPATDPVTNSTVSDTNSTISNPAGQVGNDTISGLGGKVTGSSSPSLQNKLVSGNRAS 314

Canis_lupus_dingo_ENAM KAESPAAEPSGNSTGPETNSTQSN---PGGSQSGNDTSPTGNSGPGPNTVSNPTAQNGV- 267

Vulpes_vulpes_ENAM KAESPAAEPSGNSTGPETNSTQPN---PGGSQSGNDTSPTGNSGPGPNTVSNPTAQNGV- 266

Meles_meles_ENAM KTESPATEPSANSTGPETNSTQPNARSSGGSQGGNDTSPTGNSGHGTN-----TAQNGV- 273

Mustela_putorius_ENAM KTESPGTEPSANSTGPETNSTQPNARSSGGSQGGNDTSPTGNSP-GTN-----TAQNGV- 272

Ursus_arctos_ENAM KKESPAAEPSGNSTGPETNSTQSNAPNPGGSQGGNDTSPT-----GPN-----MAQNGV- 268

Phyllostomus_hastatus_ENAM KTESPPTEPSSNSTVPETNSTQANAANPSGHPGGNDTSPTGNSGQGPNAVGNPTTQNGV- 273

Phacochoerus_africanus_ENAM KTETPATEPSVNTTVPETNSTQPNAPNPR----GNDTSPTGNSGQGPNPRSNPTGQNG-- 265

Homo_sapiens_ENAM KAESPGTEPTANSTVTETNSTQPN---PKGSQGGNDTSPTGNSTPGLNTGNNPSARNGI- 266

Saimiri_boliviensis_ENAM KAESPGTEPTANST---TNSTQPN---PRGNQGGNDTSPTGNSTPRPNTGNNPPDQNGI- 265

Erinaceus_europaeus_ENAM KTESPATEPSPNSTTPETNSTQPNAPNPRGNQPGNDTNPAGNGGPGSNTVNNPAVQN--- 269

Suncus_etruscus_ENAM KEESPAAEPTSNSTAPETNSTLSNAPSPRGNPGGNDTSTTGTAASNP--------QNGV- 256

Echinops_telfairi_ENAM KKETPAADPTTNTTIPETNATQSNAASAGGSQVGNDTTTPGHG-----PGANPTAQDGV- 267

* *:* *:* **:* .* . :

Acanthocercus_cyanogaster_ENAM_Acr TPSPT-AHVSNGNEVAQDVL----EQPDHQSKSP--SVNIMQGFPPASQHS-----TGHV 353

Acanthocercus_minutus_ENAM_Acr TPSPT-AHVSSGNGVAQDVL----EQPDHQSKSP--SVNIMQRFPPASQHS-----TGHV 353

Xenagama_zonura_ENAM_Acr TPSPT-AHVSSGNGVAQDVL----EQPDHQSKSP--SVNIMQRFPPASQHS-----TGHI 353

Agama_doriae_ENAM_Acr TPSPT-AHVLSGNGAALDAL----EQPGHQSKSP--SVNIMQSFPPANQHS-----TGHI 350

Laudakia_wui_Acr TPSPT-AHVSGGNGAAQDAL----EQYGHQPKSP--NVNIMQSFPPASQHS-----TGHV 357

Phrynocephalus_forsythii_Acr TPSPP-AHVAGGNGAAQDVL----EQSGHQDKSP--SLNIMQSFPPASQHS-----AGHV 349

Phrynocephalus_guinanensis_Acr TPSPP-AHVSGGNGAAQDVL----EQSGHQDKSP--SLNIMQSFPPASQHS-----AGHI 349

Phrynocephalus_putjatai_Acr TPSPP-AHVSGGNGAAQDVL----EQSGHQDKSP--SLNIMQSFPPASQHS-----AGHI 349

Phrynocephalus_vlangalii_Acr TPSPP-AHVSGGNGAAQVVL----EQSGHQDKSP--SLNIMQSFPPASQHS-----AGHI 349

Phrynocephalus_versicolor_Acr TPSPP-AHVSGGNGAAQDVL----EQSGHQPKSP--SVNIMQSFPPASQHS-----TGHV 349

Intellagama_lesueurii_Acr TPSPT-AHVSGGNGAAQDAL----EQSSHQPKSP----NMIQSFPPASQQS-----TGHV 351

Pogona_vitticeps_Acr TPSPT-AHVSGGNGAAQDAL----EQSGHQPKSP----NMIQSFPPASQQS-----TGHV 351

Bradypodion_pumilum_Acr TPSP--THVSGGNRAAQEAL----EPSGHQPKSP--NINIIQSFPPASQLS-----AGHV 341

Bradypodion_ventrale_Acr TPSP--THVSGGNRAAQEAL----EPSGHQPKSP--NINIIQSFPPASQLS-----AGHV 341

Chamaeleo_calyptratus_ENAM_Acr TPSPT-THVSGGNGAPQEAL----EPSGHQPKSP--NVNIIQTFPPSSQLS-----TGHV 324

Chamaeleo_dilepis_ENAM_Acr TPSPT-THVSGGNGAAQEAL----EPSGHQPKSP--NVNIIQTFPPASQLS-----TGHV 325

Chamaeleo_gracilis_ENAM_Acr TPSPT-THVSDGNVAAQEAL----EPSGHQPKSP--NVNIIQTFPPASQLS-----TGHV 324

Chamaeleo_laevigatus_ENAM_Acr TPSPT-THVSGGNGAAQEAL----EPSGNQPKSP--NVNIIQTFPPASQSS-----TGQV 322

Furcifer_pardalis_Acr TPSPT-THVPGGNGAAPEAL----EPSGHQPNSP--NVNIIQSFPPASQLS-----TGHV 324

Trioceros_affinis_ENAM_Acr TPSPT-THLSGGNGAAQEAL----EPSGHQPQSQ--NVNIIQSFPPASQLS-----TGHV 324

Trioceros_balebicornutus_ENAM_Acr TPSPT-THLSGGNGAAQEAL----EPSGHQPKSP--NVNIIQSFPPASQLS-----TGHV 324

Trioceros_harennae_ENAM_Acr TPSPT-THLSGGNGAAQEAL----EPSGHQPKSP--NVNIIQSFPPASQLS-----TGHV 324

Anolis_apletophallus_Pleu TPLPT-TKASEGNGVAPNVL----EQLRHRSKVP--NGDVIQSFPSGRQHA-----TIQV 344

Anolis_tropidonotus_ENAM_Pleur TPLPT-TKVSEGNGVAPNVI----EQLRHRPKVP--NGDVIQSFPSGRQHS-----TMHV 344

Anolis_sagrei_ordinatus_Pleu TPLPT-TKASEGNGVAPNVL----EQLRHRSKVP--NGDVIQSFPSGRQHS-----TMQI 344

Anolis_carolinensis_Pleu TPLPT-TQTSEGNGVAQNVL----EQIRHRSKLP--NGDVIQSFPSGRQHS-----TMQV 344

Ctenosaura_bakeri_Pleu TALPT-TQMSEGNGVAQNVL----EQSSHRSKFP--NDHVIQSFPSGSQHS-----TMQV 352

Cyclura_pinguis_Pleu TALPT-TQMSEGNGVAQNVL----EQSSHRSKFP--NDHVIQSFPSGSQHS-----TMQV 352

Iguana_delicatissima_Pleu TALPT-TQMSEGNGVARNVL----EQSSHRPKFP--NDHVIQSFPSGSQHS-----TMQV 352

Gambelia_wislizenii_Pleu TSLPT-TEMSEGNEVAQNVL----EQSSHRSKSL--NDQVIQSFPSGSQHS-----TMQV 352

Laemanctus_serratus_ENAM_Pleur TSLPT-TQISEGNGVPQNVL----EQSSQRSNSP--NGRVIQSFPSGSQPS-----TMQL 352

Phrynosoma_blainvillii_Pleu TPSPTSTQMSEGNGVAQNVL----EQSSHRSRSP--NGHIIQHFPSDSQHS-----TMQV 360

Phrynosoma_platyrhinos_Pleu TPSPTSTQMSEGNGVAQNVL----EQSSHRSRSP--NGHIIQHFPSGSQHS-----TMQV 363

Sceloporus_chrysostictus_ENAM_Pleur TPLPTSTHMLEGNGVAQNIL----EQSSSRSRSP--NGHVIQSFPSSSQHS-----TMQV 358

Urosaurus_nigricaudus_Pleu TPFPTSTHMLEGNGVAQNVL----EQSSNRFRSS--NGHVIQSFPSSSQHS-----TMQV 363

Sceloporus_occidentalis_Pleu TPLPTSTHMLEGNGVAQSIL----EQSSHRPRSP--NGHVIQSFPSNNQHS-----TMQV 363

Sceloporus_tristichus_Pleu TPLPTSTHMLEGNGVAQNVL----EQSSHRSRSP--NGHVIQSFPSSSQHS-----TMQV 363

Sceloporus_undulatus_Pleu TPLPTSTHMLEGNGVAQNIL----EQSSHRSRSP--NGHVIQSFPSSSQHS-----TMQV 363

Canis_lupus_dingo_ENAM ISPAT-VNIS-GQGVPRTQISWGPNQPNIHENYP--NPN-IRNFPAGRQWRPTGTFMGHR 322

Vulpes_vulpes_ENAM ISPAT-VNIS-GQGVPRTQISWGPNQPNIHENYP--NPN-IRNFPAGRQWRPTGTFMGHR 321

Meles_meles_ENAM ISPPT-INVS-GQGVPRTQITWGPSQPNIRENYP--NPN-IRNFPAGRQWRPTGTIMGHR 328

Mustela_putorius_ENAM ISPPT-INVS-GQGVPRTQITWGPSQPNIRENYP--NPN-IRNFPSGRQWRPTGTIMGHR 327

Ursus_arctos_ENAM NSSPT-VNVS-GQAVPRTQIMWGPSQPNIHENYP--NPN-NRNFPVGRQWRPTGTIMGHR 323

Phyllostomus_hastatus_ENAM VPPPA-VNVS-GQGAPRSQIPWRPNQPNIRENYP--NPN-VQSFPGGRQWRPPGTATGHR 328

Phacochoerus_africanus_ENAM ---PG-VNVS-GQGVPRSQSPWGPRQTIIHENYP--NPN-IRGFPARRQWRPPGPAMGHR 317

Homo_sapiens_ENAM GPLPA-VNAS-GQGGPGSQIPWRPSQPNIRENHP--YPN-IRNFPSGRQWYFTGTVMGHR 321

Saimiri_boliviensis_ENAM SPVPA-VNAS-GQGGPGSQIPWRPSQPSIRVNHP--NPN-IRNFPSGRQWYPTGTVMGHR 320

Erinaceus_europaeus_ENAM -PSST-VNIS-GQGIPRSQIPLGRRQPNFHGNYP--NPN-IRNYPAGRQWRPTGTILGHR 323

Suncus_etruscus_ENAM IPSPT-VNVS-NPGVSRNQIPLGPRH-NFYENYP--NPN-VRSFPAGRQWRPTGTVTWQR 310

Echinops_telfairi_ENAM GSPPA-VNAS-NQGGPGNRTPWGPRQPNVYGNYPNRNPG-GRGVPAGRQWRPAGPAAGHR 324

. . . . . : * * :

Acanthocercus_cyanogaster_ENAM_Acr L----HMYKPHSEVGDARHNSLTSRGN-PFIQSESPSYNFGY-----RENLDQRGTLQN- 402

Acanthocercus_minutus_ENAM_Acr P----HMYKPHSEMGDARHNSLTSRGN-PYIQSESPSYNFGY-----RENLDQRGNLQN- 402

Xenagama_zonura_ENAM_Acr P----HMYKPHSEMGDARHNSLTSRGN-PYIQSESPSYNFGY-----RENLDQRGNLQN- 402

Agama_doriae_ENAM_Acr P----HMYKPHSEMGDARHSSLTSRGN-PYMQSERPSYNFDN-----RENLDRRGNLQN- 399

Laudakia_wui_Acr P----HIYKPHSEMGDARHNSLTSRGN-PSIQTESPPQNFGY-----RENLDRRGHLQN- 406

Phrynocephalus_forsythii_Acr P----PIYKPNSEVGDARHHSLTSRGN-P-IQTESPSHNFGY-----RDNLDQRGNLHN- 397

Phrynocephalus_guinanensis_Acr P----PIYKPNSEVGDARHHSLTSRGN-P-IQTESPSHNFGY-----RDNLDQRGNLHN- 397

Phrynocephalus_putjatai_Acr P----PIYKPNSEVGDARHHSLTSRGN-P-IQTESPSHNFGY-----RDNLDQRGNLHN- 397

Phrynocephalus_vlangalii_Acr P----PIYKPNSEVGDARHHSLTSRGN-PSIQTESPSHNFGY-----RDNLDQRGNLHN- 398

Phrynocephalus_versicolor_Acr P----PIYKPNSEMGDARHHSLTSRGN-PSIQTESPSHNFGY-----RENLDQRGNLHN- 398

Intellagama_lesueurii_Acr P----HIYNPHSEMGDARHNTLTFRGN-PSIQAENPSRSFGY-----RDNLDPRGNLQS- 400

Pogona_vitticeps_Acr P----HIYNPHSEMGDARHNTLTFRGN-PSIQAENPSRSFGY-----RDNLDPRGNLQS- 400

Bradypodion_pumilum_Acr A----HIYNSRSEMGDARYNTLTSRGN-PPIQNENKLHSFDY-----RENADPRDNFQS- 390

Bradypodion_ventrale_Acr A----HIYNSRSEMGDARYNTLTSRGN-PPIKNENKLHSFDY-----RENSDPRDNFQS- 390

Chamaeleo_calyptratus_ENAM_Acr A----HLYNPHSEMGDARYNTLTSRGNPPPIQTENQLHSFDY-----RENSDPRDNFQS- 374

Chamaeleo_dilepis_ENAM_Acr A----HLYNPPSEMGDARYNTLTSRGNPPPIQTENQFHSFDY-----RENSDPRDNFQS- 375

Chamaeleo_gracilis_ENAM_Acr A----HLYNPPSEMGDARYNTLTSRGNPPPIQTENQFHSFDY-----KENSDPRDNFQS- 374

Chamaeleo_laevigatus_ENAM_Acr A----HLYNPRSEMGDARYNTLTSRGNPPPIQTENQLHSFDY-----RE----RDNFQS- 368

Furcifer_pardalis_Acr A----HIYNPRSEMGDARYNTLTSRGN-PHVQTENRLHSFDY-----RENSDPRDNFQS- 373

Trioceros_affinis_ENAM_Acr A----HIYNPHSEMRDARYNTLTSRGN-PPIQTENQLYSFDY-----RENSDPRDHFQR- 373

Trioceros_balebicornutus_ENAM_Acr A----HIYNPRSEMRDARYNTLTSRGN-PPIQTENQLYSFDY-----REISDPRDNFQR- 373

Trioceros_harennae_ENAM_Acr A----HIYNPRSEMRDARYNTLTSRGN-PPIQTENQLYSFDY-----REISDPRDNFQR- 373

Anolis_apletophallus_Pleu S----NIYRPHERMENIKQN-LISRGN-PSIQTEDPIYPMGN-----DWNPNHRGNIQN- 392

Anolis_tropidonotus_ENAM_Pleur S----NIYRPHEHMENIKQN-LISRAN-PSIQTEDPTYPMGN-----DWNPNHRGNIQN- 392

Anolis_sagrei_ordinatus_Pleu S----NIYRPHEHMENTKQN-LISRGN-PSIQTEDPTYPMGN-----DWNPNHRSNIQN- 392

Anolis_carolinensis_Pleu Y----NIHKPHEHMENIKQN-LISRGN-PSIQSEDPTYPMGN-----GWNSNHRGNIQN- 392

Ctenosaura_bakeri_Pleu S----NIYKPSEHTGDTRQNSLISRGN-PSIQAEDPTYPLAY-----GRNPNHRDHLQS- 401

Cyclura_pinguis_Pleu S----NIYKPSEHMGDTRQNSLISRGN-PSIQAEDPTYSLVY-----GGNPNHRDHLQS- 401

Iguana_delicatissima_Pleu S----NFYKPSEHMGDTRQNSLISRGN-PSIQAEDPTYPMAY-----GRNPNHRDHLQS- 401

Gambelia_wislizenii_Pleu S----NIYKPHEHMGDTRQNTLISRGD-PSVPVEDPTYSLGY-----GRNPNYRGNLQS- 401

Laemanctus_serratus_ENAM_Pleur S----NIYKPHEHMGDTRQNTLVSRGN-PSIQAEDPEYPLGY-----GRNPNHRDNLQS- 401

Phrynosoma_blainvillii_Pleu S----NIYKPHEHMGDTRHNIPISRGN-PSIHAEDPTYPLNY-----GRNPNHRGNLQN- 409

Phrynosoma_platyrhinos_Pleu S----NIYKPHEHMGDTRHNIPISRGN-PSIHAEDPTYPLNY-----GRNPNHRGNLQN- 412

Sceloporus_chrysostictus_ENAM_Pleur S----NIYKPHEHMGDTKHDTLISRGN-PSIHAEDPTYLLSY-----GRNPNHRGNLQN- 407

Urosaurus_nigricaudus_Pleu S----HIYKPHEHIGDTKHNTLVSRGN-PSIHAEDPTYLLSY-----GRNPNHRGNLQN- 412

Sceloporus_occidentalis_Pleu S----NIYKPLEPMGDTKHNTLTSRGN-PSIHAEDSAYLLNY-----GRNPNHRDNLPN- 412

Sceloporus_tristichus_Pleu S----NIYKPLEPMGETKHNTLISREN-PSIHAEDPAYLLNY-----GRNPNQRDNLQN- 412

Sceloporus_undulatus_Pleu S----NIYKPLEPMGDTKHNTLISRGN-PSIHAEDPAYLLNY-----GRNPNRRDNLPN- 412

Canis_lupus_dingo_ENAM Q--NGPFYRNQQVQRGPRWNSFALERK-QAMRPGNPIYRKAYASTARGNSPNHAGNLGNV 379

Vulpes_vulpes_ENAM Q--NGPFYRNQQVQRGPRWNSFALERK-QAMRPGNPIYRKAYASTARGNSPNHAGNLGNV 378

Meles_meles_ENAM Q--NGPFYRNQQVQRGPRWNSFAWERK-QAVRPGNPIYRRAYASTARGNSPNHAANLGNI 385

Mustela_putorius_ENAM Q--NGPFYRNQQVQRGPRWNSFALERK-QAVRPGNPIYRRAYASTARGNSPNYAANLGNI 384

Ursus_arctos_ENAM Q--NGPFYRNQQFQRGPRWNSFALERK-QAVRPGNPIYRKAYASTARGNSPNHAGNLGNF 380

Phyllostomus_hastatus_ENAM Q--NWPFYRN-QVQRGPRWNSYALESK-QAVRPGNPTYRKAYASTARGSSPSLAGNPTNF 384

Phacochoerus_africanus_ENAM R--NGPFYRNQQIQRGPRWNSFTLEGK-QAVRPGYPTYRRIYGSTARSNPPNYAGNSANL 374

Homo_sapiens_ENAM Q--NRPFYRNQQVQRGPRWNFFAWERK-QVARPGNPVYHKAYPPTSRGNYPNYAGNPANL 378

Saimiri_boliviensis_ENAM Q--NGPFYRNQQVQSGLRWNSFAWEGK-QVARPRNPLYHIAYPSTSRGNYPNYAGNPANL 377

Erinaceus_europaeus_ENAM Q--NWPFYRTQQVQGSPRWHSFALENK-QALRPGTPFYRKAYASTARGNSPNQAGNPANF 380

Suncus_etruscus_ENAM Q--NLPFYRNQLFQRGPQWNSFALENK-QAIHPGIPFYRKAYASTAKGNSPNQAGNPANF 367

Echinops_telfairi_ENAM PYGHGPFYRTHQVPRGPRRNTFAWAGK-QAPWPGNPAYRKAYASISRGNAPNPARNPANF 383

::. : .

Acanthocercus_cyanogaster_ENAM_Acr -----------TNNNPA---LANPRHLS-YGHQEQSHLPGRNPSDQREIVPFSASDPPST 447

Acanthocercus_minutus_ENAM_Acr -----------TNNNQA---LANPRHLS-YGQQEHSHLPGRNPSDQREIVPFSASDPPST 447

Xenagama_zonura_ENAM_Acr -----------TNNNQA---LANPRHLS-YGQQEHSHLPGRNPSDQREIVPFSASDPPST 447

Agama_doriae_ENAM_Acr -----------TNNNQA---LGNPRHLS-YGPQEQPHLPGRNPSNQREIIPFPASDPPRT 444

Laudakia_wui_Acr -----------TNNNQA---LANPRRLS-YGHQEQSHLPGRNPSGQREIMPFPASDPPST 451

Phrynocephalus_forsythii_Acr -----------TNNNQA---LANPRHLS-YGYQEQSHLPGRSPSGQREIRPFPDSDTPST 442

Phrynocephalus_guinanensis_Acr -----------TNNNQA---LANPRHLS-YGYQEQSHLPGRSPSGQREIRPFPDSDTPST 442

Phrynocephalus_putjatai_Acr -----------TNNNQA---LANPRHLS-YGYQEQSHLPGRSPSGQREIRPFPDSDTPST 442

Phrynocephalus_vlangalii_Acr -----------TNNNQA---LANPRHLS-YGYQEQSHLPGRSPSGQREIRPFPDSDTPST 443

Phrynocephalus_versicolor_Acr -----------TNNNQA---LANPRHLS-YGHQEQPHLPGRSPSGQREIRPFPDSDPPST 443

Intellagama_lesueurii_Acr -----------TNSNQA---MANPRPIS-YGHQEQSHLPGRNPSGQREIMPFPTSDPPSR 445

Pogona_vitticeps_Acr -----------TNSNQA---MANPRPIS-YGPQEQSHLPGRSPSGQREIMPIPTSDPPSR 445

Bradypodion_pumilum_Acr -----------TNNNQA---LENPRPVS-YRHQEQSHFIGRSPSGQGERKHFPTSDPLSE 435

Bradypodion_ventrale_Acr -----------TNNNQA---LENPRPVS-YRHQEQSHFIGRSPSGQGERKHFPTSDPLSE 435

Chamaeleo_calyptratus_ENAM_Acr -----------TNNNQA---LENPRPIS-YRHQEQSHFLGRSPSGQGERKHFPTSKMLSE 419

Chamaeleo_dilepis_ENAM_Acr -----------TNNNQA---LENPRPIS-YRHQEQSHFLGRNPSGQGERKHFPTSEMLRE 420

Chamaeleo_gracilis_ENAM_Acr -----------TNINQA---LENPRPIS-YRHQEQSHFLGRNPSGQGERKHFPTSEMLRE 419

Chamaeleo_laevigatus_ENAM_Acr -----------TNNNQA---LENPRPIS-YRHQEQSHFLGRSPSGQGERKHFPTSEMLSE 413

Furcifer_pardalis_Acr -----------TNNNQA---LENPKPIS-YRHQEQSHFLGRSPSGQGERNHFPTSDPLSE 418

Trioceros_affinis_ENAM_Acr -----------TNDNQA---LENPRAIS-YRHQEQSHFLGRSPSGQGERKHFPTSDPLSE 418

Trioceros_balebicornutus_ENAM_Acr -----------TNNNQA---LENPRPIS-YRHQEQSHFLGRSPSGQGERKHFPTSDPLSE 418

Trioceros_harennae_ENAM_Acr -----------TNNNQA---LENPRPIS-YRHQEQSHFLGRSPSGQGERKHFPTSDPLSE 418

Anolis_apletophallus_Pleu -----------KNVNRP---STNSRHIQ-YGQQEQPPYPERNPFSQIDRVPFPSSDPVGQ 437

Anolis_tropidonotus_ENAM_Pleur -----------TNVNHP---SPNTRHIQ-YGQQEQPHYPERNTFSQIDRVPFPSSDPVGQ 437

Anolis_sagrei_ordinatus_Pleu -----------TNVNHP---STNTRHIP-YGQQEQPHYPERNPYSQIDRVPFPSSDPVGQ 437

Anolis_carolinensis_Pleu -----------TNVNNP---STNTRHIP-YGQQEQPHYPERNEFSQIDRVPFPSSDPVGQ 437

Ctenosaura_bakeri_Pleu -----------TSINHP---STNTRHIP-HGQQDQLPYPGRNPLDRRERVPFPHSEPMGQ 446

Cyclura_pinguis_Pleu -----------TSINHP---STNTRHIP-HGQQDQLPYPGRNPLGRRERVPFPHSEPVGQ 446

Iguana_delicatissima_Pleu -----------TSINHP---STNTRHIP-HRQQDQLPYPGRNPLGRRERVPFPHSEPVGQ 446

Gambelia_wislizenii_Pleu -----------TNSNHP---STNTRHAP-NGQQEQPHHPGRNPFGQRERVPFHSSDPVGQ 446

Laemanctus_serratus_ENAM_Pleur -----------TNSNHH---STNTRHVP-YEPQEQPHYPGRNLFDQRERVPFPSSDPVGQ 446

Phrynosoma_blainvillii_Pleu -----------TDSNYP---SANTRRIP-YGQQEQPHYPGRNPLGQRERMSFPSSDPVHQ 454

Phrynosoma_platyrhinos_Pleu -----------TNSNYP---SANTRLIP-YGQQEQPHYLGRNPLGQRERMSFPSSDPVHQ 457

Sceloporus_chrysostictus_ENAM_Pleur -----------TDSNHP---SANTRHIP-YGQQEQPDYPGRNPLSQRERVSFPSSDTVHQ 452

Urosaurus_nigricaudus_Pleu -----------TDSNHP---SANTRQIP-YGQQEQPHSSGRNQLSQRERVSFPSSDTVHQ 457

Sceloporus_occidentalis_Pleu -----------TDSNHP---SANTRNIP-YGQQEQPHYHERNPLSQRERVSFPSSDTVHQ 457

Sceloporus_tristichus_Pleu -----------TDSNHP---SANTRNIP-YGPQEQRHYHERNPLSQRERVSFPSSDTVHQ 457

Sceloporus_undulatus_Pleu -----------INSNHP---SANTRNIP-YGQQEQPHYHERNSLSQRERVSFPSSDTVHQ 457

Canis_lupus_dingo_ENAM RRKPQAPNKHPMGTNVA---PLGPKHGT-VVHNEKIQNPGEKPVGPKERIVIPTRDPSGP 435

Vulpes_vulpes_ENAM RRKPQGPNKHPMGTSVA---PLGPKHGT-VVHNEKIQNPGEKPVGPKERIVIPTRDPSGP 434

Meles_meles_ENAM RRKPQGPNKHPVGTNVP---PLGPKHGT-VVHNEKVQNPGEKPVGPKERTVIPTRDPSGP 441

Mustela_putorius_ENAM RRKPQGPNKHPVGTNIP---PLGPKHGT-VVRNEKVQNPGEKPVGPKERIVIPTRDPSGS 440

Ursus_arctos_ENAM RRKPQGPNKHPVGTNVA---PLSPKQGT-VVHNEKIQNPGEKPVGPKERIVIPTRDPSGP 436

Phyllostomus_hastatus_ENAM RRKPQGPSKPPVGTNVA---PLRPKHSTTVGRNEKIQNPREKPLGEKERIVIPTRDPTGS 441

Phacochoerus_africanus_ENAM RRKPEGPNKNPMVTNVA---PPGPKHGT-VDQNENIQNPREKQVSQKERTAIPTRDPSGP 430

Homo_sapiens_ENAM RRKPQGPNKHPVGTTVA---PLGPKPGP-VVRNEKIQNPKEKPLGPKEQIIVPTKNPTSP 434

Saimiri_boliviensis_ENAM RRKPQRPNKHPVRTNGT---PLGPKHDP-VVQNEKIQNPREKPLSPKEPIIVPTKTPTSP 433

Erinaceus_europaeus_ENAM KRKPQTPNKQPIGTSVA---PVAPKRDP-VGHNEKIQNPRENSLGQKERIVIPTRDPTGP 436

Suncus_etruscus_ENAM KRKAQNPNKQPLGDNVAQNVPLGAKNDI-TGRNEKSQNPKVKPVGQKEKTVTPTRDPTSP 426

Echinops_telfairi_ENAM RRKPQGPNKQPMGGNVAQ--TLATKPGT-VVRNEKIQNPRETPLGPKERIVVPTKEPTAP 440

. .: ::: . . :

Acanthocercus_cyanogaster_ENAM_Acr WNQDPVYRDN-YQHNSPL----GGHSLDPQINTLG---------------APSGMQQSNA 487

Acanthocercus_minutus_ENAM_Acr WNQDPVYRDN-YQHNSPL----GSHSLDPQINTLG---------------APSGMQQSNA 487

Xenagama_zonura_ENAM_Acr WNQDPVYRDN-YQHNSPL----GGHSLDPQINTLG---------------APSGMQQSNA 487

Agama_doriae_ENAM_Acr WNQDPVYRDN-YQHNSPL----EGHSLDPQINTLG---------------SPSGMQQSNI 484

Laudakia_wui_Acr WNQDPVYRDN-YLHNSPS----EGHSLNPQINTLG---------------FPSGMQQSNT 491

Phrynocephalus_forsythii_Acr WNQDPVYRDN-YQRNSPS----EGHSLDPQINTLQ---------------SPSGMQQSNA 482

Phrynocephalus_guinanensis_Acr WNQDPVYRDN-YQHNSPS----EGHSLDPQINTLQ---------------SPSGIQQSNA 482

Phrynocephalus_putjatai_Acr WNQDPVYRDN-YQRNSPS----EGHSLDPQINTLQ---------------SPSGIQQSNA 482

Phrynocephalus_vlangalii_Acr WNQDPVYRDN-YQRNSPS----EGHSLDPQINTLQ---------------SPSGMQQSNA 483

Phrynocephalus_versicolor_Acr WNQDPVYRDN-YQRNSPS----EGHSLDPQINTLQ---------------SPSGMQQSNA 483

Intellagama_lesueurii_Acr WNKDPVYRD--YLHNSPS----EGHSLDPEINTLR---------------QPSGMQQSNT 484

Pogona_vitticeps_Acr WNKDPVYRDN-YLHNSPP----EGHSVDPQINTLR---------------RPSGMQQPNI 485

Bradypodion_pumilum_Acr WNKDPVYGEN-YQNNSPP----EDTSLDQPIDRLG---------------QHSGMQQSNA 475

Bradypodion_ventrale_Acr WNKDPVYGEN-YQNNSPP----EGTSLDLPVDRLG---------------QHSGMQQSNA 475

Chamaeleo_calyptratus_ENAM_Acr WNKDPVYREN-YQNNSPP----EGTSLDLPIDRLG---------------QRSGMQKSNA 459

Chamaeleo_dilepis_ENAM_Acr WNKDPVYREN-YQNNSPP----EGTSLDLPIDRLG---------------QHSGMQKSNA 460

Chamaeleo_gracilis_ENAM_Acr WNKDPVYREN-YQNNSPP----EGTSLDLPIDRLG---------------QHSGMQKSNA 459

Chamaeleo_laevigatus_ENAM_Acr WNKDPVYREN-YQNNSPP----EGTSLDLPIDRLG---------------QHSGMQKSNA 453

Furcifer_pardalis_Acr WNKDPVYREN-YQNNSPP----EGTSPDLPIDRLG---------------QHSGMPQSNA 458

Trioceros_affinis_ENAM_Acr WNKDPVYKEN-YQNNSPP----EGTSLDLPIDRLG---------------QHSGMQQSNA 458

Trioceros_balebicornutus_ENAM_Acr WNKDPVYREN-YQNTSPP----EGTSLDLPIDRLG---------------QHSGMQQSNA 458

Trioceros_harennae_ENAM_Acr WNKDPVYREN-YQNTSPP----EGTSLDLPIDRLG---------------QHSGMQQSNA 458

Anolis_apletophallus_Pleu WNKDPIYRDN-DPKNSPP----EGHLLDPQFKTMR-IDNIYNERQDAERFQPSGMHQSNV 491

Anolis_tropidonotus_ENAM_Pleur WNKDPIYRDN-DPKNSPP----EGHSLDPQFKTMR-IDNIYNERQDAERFQPSGMHQSNA 491

Anolis_sagrei_ordinatus_Pleu WNKDPIYRDN-DPKNSPP----EGHPLDPQFKTMR-IDNIYNERQDSERFQPSGMHQSNT 491

Anolis_carolinensis_Pleu WNKDPIYRDN-DPKNSPP----EGHPLDPQFKTMR-IDNIYNARQDAERFQPSGMHQSNA 491

Ctenosaura_bakeri_Pleu WNKDPVYRHN-GPENSPP----EGHFLDPQFQTLKHTDNIYNAREDIERLQPSGMQQSNT 501

Cyclura_pinguis_Pleu WNKDPVYRHN-SPKNSPP----EGHFLDPQFKKLKHTDNIYNAREDIERLQPSGMQQSNT 501

Iguana_delicatissima_Pleu WNKDPVYRHN-GPKNSPP----EGHFLDPQFKTLKHTDNIYNAREDIERLQPSGMQQSNT 501

Gambelia_wislizenii_Pleu WNKDAVYRDN-GPKISPP----EGHSLDPQFKTLRQTDNAYNARENIERLQPSGMHQSNT 501

Laemanctus_serratus_ENAM_Pleur WHKDPVYRDN-GPKNSPP----EGHSLDPQFKTLGQTDNSYNAREDIERIQPSGMYQSKT 501

Phrynosoma_blainvillii_Pleu WNKEPVYRDN-GPKNSPP----ESHSLDPQFKTLRKTDNAYNEREDIESFQPSGMHKSNT 509

Phrynosoma_platyrhinos_Pleu WNKEPVYRDN-GPKNSPP----ESHSLDPQFKTLRKTDNAYNEREDIESFQPSGMHKSNT 512

Sceloporus_chrysostictus_ENAM_Pleur WNQEPVYSDN-GPKNSPP----KSHSLDSQFKTLRQTDNTYNAREDIESFQPSGMHKSNT 507

Urosaurus_nigricaudus_Pleu WNKESVYRDN-GPINSPP----KSHFLDPQFKTLRQTDNTYNAREDVESFQPSGMHKSHT 512

Sceloporus_occidentalis_Pleu WSKEPVYRHN-GPNNSPP----KSHSLDPQFKTLRESDNTYNAREDIESFQPSGMHKSNT 512

Sceloporus_tristichus_Pleu WSKEPVYRHN-GPDNSPP----KSHSLDPQFKTLRESDNIYNAREDIESFQPSGMHKSNT 512

Sceloporus_undulatus_Pleu WSKEPVYRDN-GPKNFPP----KSHSLDPQFKTLRESDNTYNAREDIESFQPPGMHKSNT 512

Canis_lupus_dingo_ENAM WRNSQDYGVN-KSNYKLSP--PESNVLVPNFNSVDQHENSYYSRGDSRRAPNSDGQTQSQ 492

Vulpes_vulpes_ENAM WRNSQDYGVN-KSNYKLSP--PESNVLVPNFNSVDQHENSYYSRGDSRRAPNSDGQTQSQ 491

Meles_meles_ENAM WRNSQDYGVN-EPNYKLPH--PESNKLVPNFNSIDQHENSYYPRGDSSRAPNSDGQTQNQ 498

Mustela_putorius_ENAM WRNSQDYGVN-EPNYKMPH--PESNMLVPNFNSIDQRENSYYPRGDSSRAPNSDGQTQNQ 497

Ursus_arctos_ENAM WKNSQDYGVN-KSNYKLPH--PESNMLVPSFNSIDQHENSYYPRGDSRRAPNSDGQTQTQ 493

Phyllostomus_hastatus_ENAM WRNSQDYGVN-KSNYKLPH--PESNALVPNFNSIDQRENSYYPRGDSRRAPNSDGQTQTQ 498

Phacochoerus_africanus_ENAM WRNSQDYGIN-KSNYKLPQ--PEDNMLVPNFNSIDQRENSYYPRGESKRAPNSDGQTQTQ 487

Homo_sapiens_ENAM WRNSQQYEVN-KSNYKLPH--SEGYMPVPNFNSVDQHENSYYPRGDSRKVPNSDGQTQSQ 491

Saimiri_boliviensis_ENAM WRNSQQYEVNIKSNYKLPH--SEGYTPVPNFNSVDQHENSYYPRGDSRKVPNSDGQTQSQ 491

Erinaceus_europaeus_ENAM WRNSQDYGIN-KSNFKPSR--PEGNMPVPNFNSIDQHENSYYPRGDSRRAPNSDGQAPSQ 493

Suncus_etruscus_ENAM WKNSQNFGVN-KSNYKLPNPQPEGNKLIPNFNSVDQHENTHYLRGDSRRAPNSDVQTQSQ 485

Echinops_telfairi_ENAM WKNSPDDGVN-KPSYKPPH--SEGNRPVPSFNSIDQHENVYYPRGDSSRVQNADGQAPNQ 497

* :. . .. : ..

Acanthocercus_cyanogaster_ENAM_Acr P--SQQGIFSATRRTPFETETHQNDWKPQLPNL-------PDREREQFPSAQNEMWSNRE 538

Acanthocercus_minutus_ENAM_Acr P--SQQGMFSATRRTPFETETHQNDWKPKLPNL-------PDRERE-----QNEMWSNRE 533

Xenagama_zonura_ENAM_Acr P--SQQGMFSATRRTPFETETHQNDWKPKLPNL-------PDRERE-----QNEMWSNRE 533

Agama_doriae_ENAM_Acr P--SQQGMFSATRRTPFEAETHQNDWKPPLPNL-------PDREREQFPSTQNQMWSNRE 535

Laudakia_wui_Acr P--SQQGMFSATRRTPFETETHQYDWKPQFPDI-------PDREREQIPSAQNQMWSNRE 542

Phrynocephalus_forsythii_Acr P--SQQGMFSATRRTPFESETHQNNWKPQLPNL-------PDHEREQFPSAQNRMWSNRE 533

Phrynocephalus_guinanensis_Acr P--SQQGMFSGTRRTPFETETHQNNWKPQLPSL-------PDREREQFPSAQNRMWSNRE 533

Phrynocephalus_putjatai_Acr P--SQQGMFSGTRRTPFETETHQNNWKPQLPSL-------PDREREQFPSAQNRMWSNRE 533

Phrynocephalus_vlangalii_Acr P--SQQGMFSATRRTPFETETHQNNWKPQLPNL-------PDREREQFPSAHNRMWSNRE 534

Phrynocephalus_versicolor_Acr P--SQQGMFSATRRTPFETETHQNNWKPQLPNL-------PDREREQFPSAQNRMWSNRE 534

Intellagama_lesueurii_Acr L--SERGIFSATRRTPFETETHWYDWKQQLPNP-------PDRERERFPSAQNQMWSSRE 535

Pogona_vitticeps_Acr L--SEQEIFPSTKRTPFETETHWYDWKQQLPNP-------PDRERERFPSAQNQMWSSRE 536

Bradypodion_pumilum_Acr H--SQQEIFSATRRTPFETEIHHYDWKQQMSSR-------PDHEREQFPPAQNQMRNNRE 526

Bradypodion_ventrale_Acr H--SQQEIFSATRRTPFETEIHQYDWKQHMSSR-------PDHEREQFPPAQNQMWNNRE 526

Chamaeleo_calyptratus_ENAM_Acr H--SQQEIFLSTRRTPLETEIYQYDWKQQMSSH-------PDHEREQFPPAQNQMWNNRE 510

Chamaeleo_dilepis_ENAM_Acr H--SQQEIFLATRRTPFEAEINQYDWKQQMSSR-------PDHEREQFPPAQNQVWNNRE 511

Chamaeleo_gracilis_ENAM_Acr H--SQQEIFLATRRTPFETEIYQYDWKQQMSSR-------PDHEREQFPPAQNQMWNNRE 510

Chamaeleo_laevigatus_ENAM_Acr H--SQQEIFLATRRTPFETEVYQYDWKQQMSSR-------PDHEREQFPPAQNQMWNNRE 504

Furcifer_pardalis_Acr H--SQQEIFSATRRTPFETEIHQYDWKQEMSSR-------PDHEREQFPPAQKQMWNNRE 509

Trioceros_affinis_ENAM_Acr H--SQQEIFSATRRTPFETEIQQYDWKQQMSSR-------PDHEREQFSRAQNQMWNNRE 509

Trioceros_balebicornutus_ENAM_Acr H--SQQEIFSATRRTPFETEIQQYDWKQQMSSR-------PDHEREQFPPAQNQMWNNRE 509

Trioceros_harennae_ENAM_Acr H--SQQEIFSATRRTPFETEIQQYDWKQQMSSR-------PDHEREQFPPAQNQMWNNRE 509

Anolis_apletophallus_Pleu L--SHQEVNSATRRTPFEIESHQYDWREQPFKH-------IDQKRE-----HSPTWSNRE 537

Anolis_tropidonotus_ENAM_Pleur L--SHQEVNSAT-RTPFEIETHQYDWREQPFKH-------IDQKREQFPPAQTPTWSNRE 541

Anolis_sagrei_ordinatus_Pleu L--SHQGVNSATRRTPFEIETHQHDWREQPFKH-------IDQKREQFLPEQTPTWSNRE 542

Anolis_carolinensis_Pleu L--SHQGVNLATRRTPFEIETHQYDWREQPFNH-------IDQKRGQFLSAQTPTWNNRE 542

Ctenosaura_bakeri_Pleu F--SYQGVISATRRTPFETEMQQYDWNDRPVNH-------PNEKREQFSPAQNPMWNNQE 552

Cyclura_pinguis_Pleu F--SNQGVISATRRTPFEMEMQQYDWKDRPFNH-------PNEKREQFSPAQNPMWNNQE 552

Iguana_delicatissima_Pleu F--SHQRVISATRRTPLEIEMQRYDWKDRPFNH-------PNKKREQFSPAENPMWNNQE 552

Gambelia_wislizenii_Pleu F--SHQGGISTTRRTPFEIETHQYDWKEQPFNH-------LDEKREQFQPAQNPMWNNQE 552

Laemanctus_serratus_ENAM_Pleur F--SHQGVISATRRTPFEIETHQYDWKEQPFNY-------PDQKREQFLPAQNPMWNNQE 552

Phrynosoma_blainvillii_Pleu F--SHQGVISNTGRTPFEIETHQYDWKEQPFNH-------PDQQKEQFLSAQNPMWNNQE 560

Phrynosoma_platyrhinos_Pleu F--SHQGVISATGRTPFEIETHQYDWKEQPFNH-------PDQQKEQFLSAQNPMWNNQE 563

Sceloporus_chrysostictus_ENAM_Pleur F--SHQGLISATGRTPFEVETHQYDWKEQPFNH-------PDQKREQFLPEENPMWNNQE 558

Urosaurus_nigricaudus_Pleu F--SHQGLISGTGRTPFETETHQYNWKEQPFNH-------PDQKREQFLPEQNPMWNNQE 563

Sceloporus_occidentalis_Pleu F--SHQGLISATGRTPFEIETHQYDWKEQPFNH-------PDQKREQFLPEQNPMWNNQE 563

Sceloporus_tristichus_Pleu F--SHQGLISATGRTPFEIETHQYDWKEQPFNH-------PDQKREQFLPEQNPMWNNQE 563

Sceloporus_undulatus_Pleu F--SHQGLISATGRTPFEIETHQYDWKEQPFNH-------PDQKREQFLPEQNPMWNNQE 563

Canis_lupus_dingo_ENAM N--LPKGIILEPRRNPYESETNRPELKHSTYQP-VFPEEIPPPAREHFPAGRN-TWHHQE 548

Vulpes_vulpes_ENAM N--LPKGIILEPRRNPYESETHRPELKHSTYQP-VFPEEIPSPAREHFPAGRN-TWHHQE 547

Meles_meles_ENAM N--LPKGIILEPRRNPYESETNRPELKHSTYQP-AYPEEIPSPAREHFPAGRN-TWNHQE 554

Mustela_putorius_ENAM N--LPKGIILEPRRNPYESETNQPELKHSTYQP-AYPEEIPSPAREHFPAGRN-TWNHQE 553

Ursus_arctos_ENAM N--LPKGIILEPRRNPYESETNQPELKHSTYQP-VYQEEIPSPAREHFPAGRN-TWNHQE 549

Phyllostomus_hastatus_ENAM N--LPRGIILEPRRIPYESETNQPELKHSTYQP-AYPEEIPPPAREQYPGGRN-TWNHQE 554

Phacochoerus_africanus_ENAM TQIIPKGIVLEPRRIPYESETNQPELKHSAYQP-VYTEGIPSPAKEHFPAGRN-TWNQQE 545

Homo_sapiens_ENAM N--LPKGIVLGSRRMPYESETNQSELKHSSYQPAVYPEEIPSPAKEHFPAGRN-TWDHQE 548

Saimiri_boliviensis_ENAM N--LPKGIVLGPRRIPYESETNQSKLKHSSYQPPVYPEEIPSSAKEDFPAGRN-TWNHQE 548

Erinaceus_europaeus_ENAM N--LPKGIVLEPKRIPYESETNGPELKQNTYPP-VYLEEIPPPERELFPVGRN-TWNHQE 549

Suncus_etruscus_ENAM N--LPKGIVLDPKRIPYKPETNAPEIKHNAYQP-VYPAEIPSLAREHFPAGRN-SWNPQE 541

Echinops_telfairi_ENAM Q--LPKGIVLEPRKSPYESETNQPELKPSAYNP-IYPEEIPFPAGGRFPPGRN-HWTHGE 553

: . : * : * . . .. *

Acanthocercus_cyanogaster_ENAM_Acr NSWTFQD-TPPRYDSRYSFNPDHQHGQATYSER---NS----YQ--QNGHPGSPRTWEER 588

Acanthocercus_minutus_ENAM_Acr NSWTFQD-TPPRYDSRYSFNPDHQHGQAIYSER---DS----YQ--QNGHPSSPRTWEER 583

Xenagama_zonura_ENAM_Acr NSWTFQD-TPPRYDSRYSFNPDHQHGQAIYSER---DS----YQ--QNGHTSSPRTWEER 583

Agama_doriae_ENAM_Acr NSWTFQD-TPPRYNSRYSLDPDHQQGQAIYSER---NS----YQ--QNGHANSPRAWEER 585

Laudakia_wui_Acr NSWTFQD-TPPRYNSMYSLDSDHQRGQAIYSER---NS----YQ--QNGHPNSPRTWEER 592

Phrynocephalus_forsythii_Acr NSWTFQD-TPPRYNSMYSLDSDHPRGHSIYSER---NS----YQ--QNGHPNSPRTXEER 583

Phrynocephalus_guinanensis_Acr NSWTFQD-TPPRYNSMYSLDSDHPRGHSIYFER---NS----YQ--QNGHPNSPRTWEER 583

Phrynocephalus_putjatai_Acr NSWTFQD-TPPRYNSMYSLDSDHPRGHSIYFER---NS----YQ--QNGHPNSPRTWEER 583

Phrynocephalus_vlangalii_Acr NSWTFQD-TPPRSNSMYSLDSDHPRGHSIYSER---NP----YQ--QNGHPNSPRTWEER 584

Phrynocephalus_versicolor_Acr NSWTFQD-SPPRYNSMYSLHSDHPRGHSIYSER---NS----YQ--QNGHPNSPRTWEER 584

Intellagama_lesueurii_Acr NSQIFQE-APPRYNSMYSLDSVHQRGQAIYSER---NSFRNPYQ--QNGHPISSRTWEER 589

Pogona_vitticeps_Acr NSQIFQD-APPRYNSMYSLDSIHQRGQEIYSER---NSFRNPYQ--QNGHPSTSRTWEER 590

Bradypodion_pumilum_Acr NSWVFQDKTPPRYKTMYSLDSTHQRGQAVYS-----------YQ--QNGRPISSGTWEER 573

Bradypodion_ventrale_Acr NSWVFQDKTPPRYKTMYSLDSTHQRGHTVYS-----------YQ--QNGRPISSGTWEER 573

Chamaeleo_calyptratus_ENAM_Acr NSRVFQDTTPPRYKTMHSLDSTHQRGQAVYS-----------HQ--QNGHPISPGTWEER 557

Chamaeleo_dilepis_ENAM_Acr NSRVFQDTTPPRYKTMYSLDSMHQRGQAVYS-----------HQ--QNGHPISPGTWEER 558

Chamaeleo_gracilis_ENAM_Acr NSQVFQDTTPPRYKTMYSLDSTHQRGQAVYS-----------HQ--QNGHPISPGTWEER 557

Chamaeleo_laevigatus_ENAM_Acr NSRVFQDTTPPRYKTMYSLDSTLQRGQAVYS-----------HQ--QSGHPISPGTWEER 551

Furcifer_pardalis_Acr NSWVFQDTTPPRYKTMHSLDSTHQRGQATFS-----------YQ--QNGHPISPGTWEER 556

Trioceros_affinis_ENAM_Acr NSWVFQDKTPPRYKTMYSLDSAHQRGQAVYS-----------YQ--QNGHPISPGTWEGR 556

Trioceros_balebicornutus_ENAM_Acr NSWVFQDKTPPRYKTMYSLDSAHQRGQAVYS-----------YQ--QNGHPISPGTWEER 556

Trioceros_harennae_ENAM_Acr NSWVFQDKTPPRYKTMYSLDSAHQRGQAVYS-----------YQ--QNGHPISPGTWEER 556

Anolis_apletophallus_Pleu DTQLFQE-APPRHRSMYSSGSFNQREISSYSGK---KS----YD--QYTHPFHPRAWEDR 587

Anolis_tropidonotus_ENAM_Pleur DTQLFQE-APPRYRSMYSSGSFNQREMPSYSGK---KS----YD--QYTHPFLPRAWEDR 591

Anolis_sagrei_ordinatus_Pleu DSRLFQE-APPRHRSMYSSGSFNQREMPSYSGK---KS----YD--QYTHPFLPRAWEDR 592

Anolis_carolinensis_Pleu ASQLFQE-APPRQSSMYSSGSFNQREMPTYSGK---KS----YN--QYTHPFPPRAWEDR 592

Ctenosaura_bakeri_Pleu DLQVFKE-APPRYNSMYSSGTFNQRELPSSLGK---TP----YG--QHIRSFPPREWEDR 602

Cyclura_pinguis_Pleu DLQVFQE-APPRYNSMYSSGTFNQRELPSSLGK---TP----YG--QHIHSFPPRAREDR 602

Iguana_delicatissima_Pleu DLQVIQE-APPRYNSMYSSGTFNQRELPSSLGK---TP----YG--QHIHSFSPRAWEDR 602

Gambelia_wislizenii_Pleu DLQVFQE-VPPRYNSMHSSGSFNQRGLPTYSGK---NS----YD--QHVHFFPPRAWEDR 602

Laemanctus_serratus_ENAM_Pleur -SQVFQE-APPRYNSMYSSGSFNQRGLPTYSEK---SP----YS--QHVHSFPPKAWEDR 601

Phrynosoma_blainvillii_Pleu DSQVFHK-APPRSSSMYSSGPFNQRGLPTYLGK---NS----YS--Q---SITPRAWEDR 607

Phrynosoma_platyrhinos_Pleu DSQVFHK-APPRHNSMYSSGPFNQRGLPTYLGK---NS----YS--Q---SIIPGAWEDR 610

Sceloporus_chrysostictus_ENAM_Pleur DSQMFQK-APPRYNSMYSSDSFNQRGLPTYLGE---NS----YS--Q---PIPPRAWEDR 605

Urosaurus_nigricaudus_Pleu DSQVFQE-APPRYNSMYASDSFNQRGLPTNLGK---NS----YS--Q---PIPPRAWDDR 610

Sceloporus_occidentalis_Pleu GSQVFQE-APPRYNSIYSSDSFNQRGLPIHLEK---NS----YS--Q---PIPPRAWEDR 610

Sceloporus_tristichus_Pleu GSQVFQE-APPRYNSVYSSDYFNQRGLPTHLEK---NS----YS--Q---PIPPRAWEDR 610

Sceloporus_undulatus_Pleu GSQVFQE-APPRYNSVYSSDYFNQRGLPTHLEK---NS----YS--Q---PIPPRAWEDR 610

Canis_lupus_dingo_ENAM ISPSFKE-DPGRQEELLPPPSQGSRGGVYYPDY---NS----YDPRENSPYLRSNRWDER 600

Vulpes_vulpes_ENAM ISPSFKE-DPGRQEELLPPPSQGSRGGVYYPDY---NS----YDPRENSPYLRSNRWDER 599

Meles_meles_ENAM ISPSFKE-DPGRQEEHLPHPSHGSRGGVYYPDY---NS----YDPRENSPYLRSNTWDER 606

Mustela_putorius_ENAM ISPSFKE-DPGRPEEHLPHPSHGSRGGVYYPDY---NS----YDPRENAPYLRSNTWDER 605

Ursus_arctos_ENAM ISPSFKE-DPGRQEEHLPHPSHGSRGGVYYPDSYYPNS----YDPRENSPYPRSNTWDER 604

Phyllostomus_hastatus_ENAM ISPPFKE-DPGRQAEHLPHPPHGSRVDVHYSDY---NP----YDPRENSPYIRSRKWDER 606

Phacochoerus_africanus_ENAM ISPPFKE-DPGRQEEHLPHLSHGSRVHVYYTDY---NP----YDPRENSPYLRSNTWYER 597

Homo_sapiens_ENAM ISPPFKE-DPGRQEEHLPHPSHGSRGSVFYPEY---NP----YDPRENSPYLRGNTWDER 600

Saimiri_boliviensis_ENAM ISPPFKE-DPGRQEEHLPHSSHGSRGSVFYPEY---NP----YYSRENSPYRRSNTWDER 600

Erinaceus_europaeus_ENAM ISPPFKE-DPRRQEEHLPRPSQGSRGGVYYPDY---NP----YTPRENVPYHRSNTWDER 601

Suncus_etruscus_ENAM ISPSFKE-DPERQEEHIPRPSLGSRGSIYYPDY---NP----YDPRENSPYLRGNTWDER 593

Echinops_telfairi_ENAM LSPTLQE-ALGRQEGPLPLPSQGSRGSVFYSEY---NP----YHPRENSPYLRSNTWEER 605

::. * . : : : *

Acanthocercus_cyanogaster_ENAM_Acr KDSPV---AGQRESLSYSPLLPSNQIQKDTYHRGVQQE---RKPYPSENPWIYEKHLQED 642

Acanthocercus_minutus_ENAM_Acr KDSPV---AGQREGLSYSPLLPSNQIQKDTYHRGVQQE---RKPYPSENPWIYEKHLQDD 637

Xenagama_zonura_ENAM_Acr KDSPV---AGQRESLSYSPLLPSNQIQKDTYHRGVQQE---RKPYPSENPWIYEKHLQDD 637

Agama_doriae_ENAM_Acr IDSPV---AGQRESLSYSPVLPSDQIQKDTYHRGVQQE---HKPYPSQNPWIYEKHLLDD 639

Laudakia_wui_Acr KDSPV---TGQREGLSYSPVLPSNQMQKDTYHRGVQQE---HEPYPSQNPWIYEKRLLDD 646

Phrynocephalus_forsythii_Acr EDSPV---TGQRESLSYSPVLPSNQMQKDTYHRGVQQE---HKPYPSQDPWIYEKHLLDD 637

Phrynocephalus_guinanensis_Acr EDSSV---TGQRESLSYSPVLPSNQMQKDTYRRGVQQE---HKPYPSQDPWIYEKHLLDD 637

Phrynocephalus_putjatai_Acr EDSSV---TGQRESLSYSPVLPSNQMQKDTYRRGVQQE---HKPYPSQDPWIYEKHLLDD 637

Phrynocephalus_vlangalii_Acr EDSSV---TGQRESLSYSPVLPSNQMQKDTYRRGIQQE---HKPHPSQDPWIYEKHLLDD 638

Phrynocephalus_versicolor_Acr EDSPV---TGQRESLSYSPVLPSNQMQKDTYRRGVQQE---HKPYPSQDPWIYENHLLDD 638

Intellagama_lesueurii_Acr KDSPVTGSTGQRERLSYSPMLPSNQMQKNTYHRSVQQE---HEAHPNQNPWIDEKHLLDV 646

Pogona_vitticeps_Acr KDSPVTGSTGQRERLSYSPILPSNQMQKNHYHRSVQQE---HEAHPNQNPWVDEKHLLDV 647

Bradypodion_pumilum_Acr KDSPIISSANQRKSLSYSPSMPSNQKQ-NTYYRSVQEE---NEPPPNQNPWTHENNLLDF 629

Bradypodion_ventrale_Acr KDSPIISSANQRKSLSYSPSMPSNQMQ-NTYYRSVQEE---NEPPPNQNPWTHENNLLDF 629

Chamaeleo_calyptratus_ENAM_Acr KDSPIISSANQRKSLSYSPSMPSNQMQ-NSYYRRVQEE---NESPPNQNPWTHENNLLDF 613

Chamaeleo_dilepis_ENAM_Acr KDSPIISSANQRKSLSYSPSMPSNQMQ-NSYYRRIQEE---NESPPNQNPWTHENNLLDF 614

Chamaeleo_gracilis_ENAM_Acr KDSPIISSTNQRKSLSYSPSIPSNQMQ-NSYYRRIQEE---NESPPNQNPWTHENNLLDF 613

Chamaeleo_laevigatus_ENAM_Acr KDSPIISSANQRKSLSYSPSMPSNHMQ-NSYYRRIQEE---NGSPPNQNPWTHENNLLDF 607

Furcifer_pardalis_Acr KDSPIISSANQRKSLSYSPSMPSNQMQ-DTYHRRVQEE---NEPPPNQNPWTHENNLLDF 612

Trioceros_affinis_ENAM_Acr KDSPIMSSANQRKSLSYSPSMPSNQMQ-NTYYRRVQEE---NEPPPNPNPWRHENNLLDF 612

Trioceros_balebicornutus_ENAM_Acr KDSPIISSAYQRKSLSYSPSMPSNQMQ-NTYYRRVQEE---NEPPPNPNPWRHENNLLDF 612

Trioceros_harennae_ENAM_Acr KDSPIISSANQRKSLSYSPSMPSNQMQ-NTYYRRVQEE---NEPPPNPNPWRHENNLLDF 612

Anolis_apletophallus_Pleu ENSPTISPSDQRESPPYSSASPSDQIQRKIYYRRIQPE---YEPYPRQDPWAREQHLLDL 644

Anolis_tropidonotus_ENAM_Pleur ENSPTISPSDQRESPPYSPTSPSDQIQRKIYYRRIQPE---YEPYPRQDPWTREQQLLDP 648

Anolis_sagrei_ordinatus_Pleu ENSPTISPSDQRESPPYSPASPSDQIQRKIYYRRIQPE---YEPYPRQDPWTRE-HLLDL 648

Anolis_carolinensis_Pleu EHSPTISPSDQRESSPYPPVSPSDQIQRNTYYRRIQPE---YEPYSRQDPWTREQHLHDP 649

Ctenosaura_bakeri_Pleu ENSPTISPSDQRESPSYSPASPSDKMQRNTYYRRVQPE---YEPYPRQDPWTREQHLVDL 659

Cyclura_pinguis_Pleu ENSPTISPSDQRESPSYSPALPSDQMQRNTYYRRVQPE---YEPYPRQDPWTREQHLLDL 659

Iguana_delicatissima_Pleu ENSPTISPSDQRESPSYSPASPSDQMQRNTYYRRVQPE---YEPYPRQDPWTHEQHLLDL 659

Gambelia_wislizenii_Pleu ENAPTIRPSDQRESRSYSPASPSEQKQRNTYYRRVQPE---YEPYPRQGPWTREQHLLDL 659

Laemanctus_serratus_ENAM_Pleur EISPTISPPDQRESPSYSPA--SDQTQRNSYYRRVQPE---YEPHPRQDPWTREQHLLDL 656

Phrynosoma_blainvillii_Pleu ENSPAISPSGQRDNPSYSPASPSHQMQRNTYYRRVQPE---YEPHPRQDPWTREQHLLDR 664

Phrynosoma_platyrhinos_Pleu ENSPAISPSGQRDNPSYSPASPSHQMQRNTYYRRVQPE---YEQHPRQDPWTREQHLLDR 667

Sceloporus_chrysostictus_ENAM_Pleur DNSPTTSPSGQRENPSYFPASPSHQMQRNTYYRRVQPE---YVPHPRQDPWTRE-HLLDR 661

Urosaurus_nigricaudus_Pleu DNSPTISPSGQRENPSYSPALPSHQMQRNAYYRRVEPE---YVPHPRQDPWTREQHLLDR 667

Sceloporus_occidentalis_Pleu DNSPTISPSGQMENPSYSPASPSHQMQRNTYYRRVQPE---YVPHPRQDPWTREQHLFDR 667

Sceloporus_tristichus_Pleu DSSPTISPSGQMENPSYSPASPSHQMQRNTYYRRVQPE---YVPHPRQDPWTREQHLFDR 667

Sceloporus_undulatus_Pleu DSSPTISPSGQMENPSYSPASPSHQIQRNTYYRRVQPE---YVPHPRQDPWTREQHLFDR 667

Canis_lupus_dingo_ENAM DDSPN--TIGQPRNSLYPINTPELKET-VPYNEEDPIDPTGDETFPGQNRWGMEEPSFKE 657

Vulpes_vulpes_ENAM DDSPN--TIGQPKNSLYPINTPELKET-VPYNEEDPIDPTGDETFPGQSRWGMEEPSFKE 656

Meles_meles_ENAM DDSPN--NIGQPQNSLYPINTPELKET-VPYNEEDPVDPAGDEHFPGQSRWGMEETSFRE 663

Mustela_putorius_ENAM DDSPN--NIGQPRNSLYPINTPELKEI-VPYNEEDPVDPAGDEHFPGQSRWGMEETSFKE 662

Ursus_arctos_ENAM DDSPN--NIGQPKNSLYPINTPELKET-VPYNEEDPLDPTGDESFPGQSRWGMEETSFKE 661

Phyllostomus_hastatus_ENAM DDSPN--TMGLPEDPLYPMNTPDPKET-VPYNEEDPVDPTGDELFPGQSKWGVEESSFKE 663

Phacochoerus_africanus_ENAM DDSPN--TMGQPENPHYPMNTPDPKET-IPYNEEDPIDPTGDEHFPGQSRWDMEELSFKE 654

Homo_sapiens_ENAM DDSPN--TMGQKESPLYPINTPDQKEI-VPYNEEDPVDPTGDEVFPGQNRWG-EELSFKG 656

Saimiri_boliviensis_ENAM EDSPN--TMGQKESPLYPINTPDQKET-VPYNEEDPIDPNGDEYFPGQNRWG-EELSFKG 656

Erinaceus_europaeus_ENAM DDSPN--TVRQPENPLYPMNTPDPKDT-VPYNEEDPADPTGDETYLGQSQWGMEESNFKG 658

Suncus_etruscus_ENAM YESSN--TMNQPESPLFPMNTPNPKET-SPYNEEGPADPNGEESFTGQSRWGMEESRFKG 650

Echinops_telfairi_ENAM GDPPN--TRGQPENRLYRMNTPDRKEI-VPYNEEDPIDPTGDEPFPRPGRWGEEESSFKG 662

.. : . : * . : . * *

Acanthocercus_cyanogaster_ENAM_Acr D---RQYKNPSYNLAQHHMHSGYPTETSPSERANLPYEEINQWTPGKRTLHPGMEYL--K 697

Acanthocercus_minutus_ENAM_Acr D---RQHKNPSYNLAQHHMHSGYPTETSPNERANLAYEEINQWTPGKRTLHPGTEYL--K 692

Xenagama_zonura_ENAM_Acr D---RQHKNPSYNLAQHHMHSGYPTETSPNERANLAYEEINQWTPGKRTLHPGTEYL--K 692

Agama_doriae_ENAM_Acr D---RQYKNPSYNLAQHHTPSGYPTESLANERANLPYEDINQWTPGKRTLRPGTDYL--K 694

Laudakia_wui_Acr D---TQYKNPSYNPAQHHMHPGYSTETPTNKRADLPYGEINQWSPGKRTLGPHTEHL--K 701

Phrynocephalus_forsythii_Acr D---RQYKNPSYNPAQHHMHPGYSTETPANERVNLPYEEINQWTPGKGTLGPGTEYL--K 692

Phrynocephalus_guinanensis_Acr D---RQYK----NPAQHHMHQGYSTETPANERVNLPYEEINQWTPGKRTLGPGTEYL--K 688

Phrynocephalus_putjatai_Acr E---RQYKNPSYNPAQHHMHQGYSTETPANERVNLPYEEINQWTPGKRTLGPGTEYL--K 692

Phrynocephalus_vlangalii_Acr D---RQYKNPSYNPAQHHMHLGYSTETPANERVNLPYEEINQWTPGKRTLGPGTEYL--K 693

Phrynocephalus_versicolor_Acr D---RQYKNPSYNPAQHHMHPGYSTETPANERVNLPYEEINQWTPGKRTLGPGTEYL--K 693

Intellagama_lesueurii_Acr D---REYKNPSYNPAQYHMHPRYSTETPPNERDNLPYEEMNQWTPEKHTLIPGTEHL--K 701

Pogona_vitticeps_Acr D---REYKNPSYNPPQYPMHPRFSTETPPNERDNLPYERMNQWTPEKHIFIPVTEHL--K 702

Bradypodion_pumilum_Acr D---RRYKNLPYDPAQHQMYSRYSTEAAANERHNLPYEKINQWAQEKHILVPSTEHL--K 684

Bradypodion_ventrale_Acr D---RRYKNLPYDPAQHQIYSRYSTEAAANERHNLPYEKINQWAQEKHILVPSTEHL--K 684

Chamaeleo_calyptratus_ENAM_Acr D---RQYKNPPYDPAQHQMYSRYSTEAAANERHNLPYEKINQWAQEKHILVPGTEHL--K 668

Chamaeleo_dilepis_ENAM_Acr D---RQYKNRPYDPAQRQMYSRYSTEAATNERHNLPYEKVNQWAQEKHILVPGTEHL--K 669

Chamaeleo_gracilis_ENAM_Acr D---RQYKNPPYDPAQHHMYSRYSTEAATNERHNLPYEKVNQWAQEKHILVPGTEHL--K 668

Chamaeleo_laevigatus_ENAM_Acr D---RQYKNPPYDPAQHQMYSRYSTEAAANERHSLPYEKINQWAQEKNILVPGTEHL--K 662

Furcifer_pardalis_Acr D---RQYKNPPYEPAQHQMYSRYSTEAAANERHNLPYEKINQWAQEKHILVPGTEHL--K 667

Trioceros_affinis_ENAM_Acr D---RQYKNAPYDPAQHQMYLRHSTEAAANERHNLPYEKINQWAQEKHIVVPGTEHL--K 667

Trioceros_balebicornutus_ENAM_Acr D---RQYKNAPYDPAQHQMYSRYSTEAAANERHNLPYEKINQWAQEKHLLVPGTEHL--K 667

Trioceros_harennae_ENAM_Acr D---RQYKNAPYDPAQHQMYSRYSTEAAAKERHNLPYKKINQWAQEKHILVPGTEHL--K 667

Anolis_apletophallus_Pleu D---NQYSNSPYNPAHHQTYPKYATENPPSEISNLPYQKINQWTQEEHPPVHTAGHL--R 699

Anolis_tropidonotus_ENAM_Pleur D---NQYSNSPYNPAHHQTYPKYATENPPSKISNLPYQKINQWTQEEHSPVHTAGHL--S 703

Anolis_sagrei_ordinatus_Pleu D---KQYNNSPYNPAHHQTYPKYATENPPSEISNLPYQKINQWTQEEHSPIQSAGHL--R 703

Anolis_carolinensis_Pleu D---NQYSNSPYNPTHHQTYQKYTTENPPSEISNLPYRKINQWTQEEHSPVHTAGHL--R 704

Ctenosaura_bakeri_Pleu K---DQYSNSPYKPTLHHAYPKYSTENRASEISNLAHEKMKQWTQEENPPVYSAAHL--R 714

Cyclura_pinguis_Pleu E---DQYSNSPYKPTHHHAYPKYSTENRASEMSNLPHEKMKQWTQDENPLVHSAAHL--R 714

Iguana_delicatissima_Pleu D---DQYGNSPYKPTHHHAYPKYSTENRASEISNLPREKMKQWTQEENPPVHSAAHL--R 714

Gambelia_wislizenii_Pleu D---NQYSNSPYNPTRHHAYPKYSTDNPASEISSLPYEKINQWTQEENPPVHSAGHL--R 714

Laemanctus_serratus_ENAM_Pleur D---NQYSNSPYNPNHPHAYPKYSTDNPTSEISNLPYDKINRWTQEEHPLIHTAGHL--R 711

Phrynosoma_blainvillii_Pleu D---NQYSNSAYNPINQHANPKYTIENPASEISNLPYEKINQWTQEEYPPVHSAGHL--R 719

Phrynosoma_platyrhinos_Pleu D---NQYSNSPYNPINQHANPKYTIENPASEISNLPYEKINQWTQEEYPPVHNAGHL--R 722

Sceloporus_chrysostictus_ENAM_Pleur D---NQYSNSQYNPTHRHANSKYTIENPASEISNLPYEKINQWTQEEHPPVHSGGHL--R 716

Urosaurus_nigricaudus_Pleu E---NQFSNSPYNPTHHSANSKYTIENPASEISNLPYEKINQWTQEEHIPVHSEGHL--R 722

Sceloporus_occidentalis_Pleu D---NQYSNSPYNPTHHHENTKYIIENPASEISNLPYEKINQWTQEERPPVHSGVHL--R 722

Sceloporus_tristichus_Pleu D---NQYSNSPHNPTHHHENTRYIIENPASEISNLPYEKINQWTQEERPPVHSGGHL--R 722

Sceloporus_undulatus_Pleu D---NQYSNSPHNPTHHHENTRYIIENPASEISNLPYEKINQWTQEERPPVHSGGHL--R 722

Canis_lupus_dingo_ENAM GPTVRHYEGEQYTVNQPKEYLPYSLDNPSKTREDFPYGEFYPWNPDENFPSYNTAPTVPP 717

Vulpes_vulpes_ENAM GPTVRHYEGEQYTVNQPKEYLPYSLDNPSKTREDFPYGEFYPWNPDENFPSYNTAPTVPP 716

Meles_meles_ENAM GPTVRHYEGEQYTSNQPKEYLPYSLDNPSKPREDFPYGEFYPWNPDENFPSYNTAPTVPP 723

Mustela_putorius_ENAM GPTVRHYEGEQYTSNQPKEYLPYSLDNPSKPREDFPYGEFYPWNPDENFPSYNTAPTVPP 722

Ursus_arctos_ENAM GPTVRHYEGEQYTSNQPKEYLPYSLDNPSKPREDFPYGEFYPWNPDEKFPSYNTAPTVPP 721

Phyllostomus_hastatus_ENAM GTTVRHYKGEQYTSNQPKEYLPYSLDNPTKPREDFPYGEYYPWNPDESFPSYNAVPTVLP 723

Phacochoerus_africanus_ENAM DPTVRHYEGEQYTSNQPKEYLPYSLDNPSKPREDFLYGEFYPWNPEENFPSYNTAPTVPS 714

Homo_sapiens_ENAM GPTVRHYEGEQYTSNQPKEYLPYSLDNPSKPREDFYYSEFYPWSPDENFPSYNTASTMPP 716

Saimiri_boliviensis_ENAM SPTVRHYEDEQYTSNQPKEYLPYSLDNPSKPKEDFYYSEFYPWSLDENIPSYNTAPTMSP 716

Erinaceus_europaeus_ENAM GPTVRQYEREQFTSNQPKDYLPYPLDNPSKPREEFPYNEFYPWNPDETFPSYNTVPSVPP 718

Suncus_etruscus_ENAM SPTIRQYEGEQFTSNQPKEYLPYTLDKPLKPSEDFPYGEFYPWNPEENFPSYNTVPSV-P 709

Echinops_telfairi_ENAM SPTLRHYESQRYASNQPKDYLPYTLENPSKPREDFPYGDFYPWGPDDHFPPYNTVPTILP 722

.. . : . .: * .

Acanthocercus_cyanogaster_ENAM_Acr QVENMPYHTN---GQQDQYMENRGSNP-PPQSSSLP-QGGSQFRERSSWVRPML------ 746

Acanthocercus_minutus_ENAM_Acr QVENMPYHTN---GQQDQYMENRGSNP-PPQSPSLP-QWGSQYGERNSWVRPML------ 741

Xenagama_zonura_ENAM_Acr QVENMPYHTN---GQQDQYMENRGSNP-PPQSPSLP-QWGSQYGERNSWVRPML------ 741

Agama_doriae_ENAM_Acr QVEHMPYHTNGMSGQKDQYMENQASNP-PPQSPSPS-QWGSQHGERNSWARPML------ 746

Laudakia_wui_Acr QVENIPYHTNGMFGQQDPNMENRGSNP-PPQSTSLS-QRESQYGERNTWIHPMP------ 753

Phrynocephalus_forsythii_Acr QVENIPYHTNGMFGQKDQYMENRGSNL-PPQSTSLS-QWGSQYGERNSWVPPML------ 744

Phrynocephalus_guinanensis_Acr QVENIPYHTNGMFGQKDQYMENRWSNP-PPQSTSLS-QRGSQYGERNSWVPPML------ 740

Phrynocephalus_putjatai_Acr QVENIPYHTNGMFGQKDQYMENRWSNP-PPQSTSLS-PRGSQYGERNSWVPPML------ 744

Phrynocephalus_vlangalii_Acr QVENIPYHTNGMFGQKDQYMENRGSNP-PPQSTSLS-QRGSQYGERNSWVPPML------ 745

Phrynocephalus_versicolor_Acr QMDNIPYHTNGMFGQKDQYMENRGSNP-PPQSTSLS-QRGSQYGERNSWVPPML------ 745

Intellagama_lesueurii_Acr QVENIPYHTNGMFRQKDQNMENQGSNS-PPQSTSLS-QRGAQYAERSTWVPPIL------ 753

Pogona_vitticeps_Acr PVENIPYHTNGMFGQKDQNMGNQGSNS-PPQSISLS-QRGAQYAERSTWVPPIL------ 754

Bradypodion_pumilum_Acr QVENIPYDTNGMFGQKDQNMMKQGSDS-PPQGTTLS-QRGAQYAERNPWVSPIL------ 736

Bradypodion_ventrale_Acr QVENIPYDTNGIFGQKDQNMMKQGSDS-PPQGTTLS-QRGAQYAERNPWVSPII------ 736

Chamaeleo_calyptratus_ENAM_Acr QVENIPYDTNGMFGQKEQNMMMQGSDS-PPQGTTLS-QRGAQYAERNPWVSPIH------ 720

Chamaeleo_dilepis_ENAM_Acr QAENIPYDTNGMFGQKEQNMITQWSDS-PPQGTTLS-QRGAHYAERNPLVSPIL------ 721

Chamaeleo_gracilis_ENAM_Acr QAENIPYDTNVMFRQKEQNMKTQGSDS-PPQGTTLS-QRGAHYAERNPWVSPIL------ 720

Chamaeleo_laevigatus_ENAM_Acr QVENIPYDTNGMFGQKEQNMMTQGSDS-PPQGTTLS-QRGAQYAERNPWVSPIL------ 714

Furcifer_pardalis_Acr HVENIPYDTNGIFGQKDHNMMKQGSDS-PPQGTTLS-QRGAQYAERNPWASPIF------ 719

Trioceros_affinis_ENAM_Acr QVENIPYDTNGMFGQKDQNMMKQGSDS-PPEGTTLS-QQGAQYAERNPRVSPIL------ 719

Trioceros_balebicornutus_ENAM_Acr QVENIPYNTNGMFEQKDRNMMKQGSDS-PPEGTTLS-QQGAQYAERKPRVSPIL------ 719

Trioceros_harennae_ENAM_Acr QVENIPYDTNGMFEQKDRNMMKQGSDS-PPEGTTLS-QQGAQYAERNPWVSPIL------ 719

Anolis_apletophallus_Pleu QVENAPYHTNSMFEQGERKFQNVGSSS-PQQSNTF--QEEAQYADRNTWIPQRV------ 750

Anolis_tropidonotus_ENAM_Pleur QVGNVPYHTNSMFEQGERTFQNVGPSS-PQQSNTF--QEEAQYAERNTWIPQRV------ 754

Anolis_sagrei_ordinatus_Pleu QVENVPYHTNSMFEQGERKFQNVRSSS-PQQSNTF--QEEAQYAERNTWFPQRV------ 754

Anolis_carolinensis_Pleu QMENVPYRTNSKFEQGERKFQNVGSGS-PQQRNTF--QEEAQYAERNTWVPQRV------ 755

Ctenosaura_bakeri_Pleu QMENVPYHTNSMFGQREREFQNVEPIS-PQQRITF--QDEAQHAERNTWIPQMV------ 765

Cyclura_pinguis_Pleu QMENVPYHTNGMFGQREREFQNVEPIS-PQQRITF--QDEAQYAERNTWIPQMV------ 765

Iguana_delicatissima_Pleu QMENVPYHTNSMFGQREREFQNVEPIS-P-QRITF--QDEAQYAERNTWIPQMV------ 764

Gambelia_wislizenii_Pleu HMENVPYHTNSMSGQRERKFQNIGSNS-PQQRISF--PEEAEYTERNTWSPQSL------ 765

Laemanctus_serratus_ENAM_Pleur QVENVPYHTNSMFGQRERKFQNVGSNS-PQQSITF--QEEAQYAEKNRWIPPRASPRG-- 766

Phrynosoma_blainvillii_Pleu QVGNAPYQSNSMFGHRERKFQNVGSNS-PQESITV--QEEAQYAERNMWIPQRV------ 770

Phrynosoma_platyrhinos_Pleu QVENVPYQSNSMFGHRERKFQNVGPNS-PQESITV--QEEAQYAERSMWVPQRV------ 773

Sceloporus_chrysostictus_ENAM_Pleur QVENVPYQSKSMLGQRERKFQNVGSSS-PQQSIAI--QEAAQYAERNTWIPQRI------ 767

Urosaurus_nigricaudus_Pleu QVENVPYQSKSMFGQRERIFQNVGANL-PQQSIAV--QEEAQYPGRNTWIPQRI------ 773

Sceloporus_occidentalis_Pleu QVENVPYQSKSMFGQRERKFQNAESNS-PQQSIAV--QEAAQYAERNSWIPQRR------ 773

Sceloporus_tristichus_Pleu QVENVPYQSKSMFGQRERKFQNAESNF-PQQSIAV--QEEAQYAERNSWIPQRR------ 773

Sceloporus_undulatus_Pleu QVENVPYQSKSMFGQRERKFQNAESNF-PQQSIAV--QEEAQYAERNSWIPQRR------ 773

Canis_lupus_dingo_ENAM PVESRGYYANNAVRQEESPLFPSWNSW-DHRVQAQGQKERQPYFNRNYWDQPTTLHKAPP 776

Vulpes_vulpes_ENAM PVESRGYYANNAVRQEESPLFPSWNSW-DHRVQAQGQKERQPFFNRNYWDQPTTSHKAPP 775

Meles_meles_ENAM PVESRGYYVNNAVGQEESPLFPSWNSW-EHKTQTQGQKERRPYFNRNFWDQPTTLHKMPP 782

Mustela_putorius_ENAM PVESRGYYVNNAVGQEESPLFPSWNSW-EHKIQTQGQKERRPYFNRNFWDQPTTLHKMPP 781

Ursus_arctos_ENAM PVESRGYYANNAVGQEESTLFPSWNSW-DHRIQTQGQKERRPYFNRNFWDQPTTLHKAPP 780

Phyllostomus_hastatus_ENAM PVESRGYYANRAVGQEEGTLFPSWNSW-DRRIEAQGQRERRPYFDRNFWDQPRNLHKAPA 782

Phacochoerus_africanus_ENAM PVESRGYYANNAVGQEESTMFPSWSSW-DPRIQAQGQKEGRPYLNRNFWDQSTNLYKTPT 773

Homo_sapiens_ENAM PIESRGYYVNNAAGPEESTLFPSRNSW-DHRIQAQGQRERRPYFNRNIWDQATHLQKAPA 775

Saimiri_boliviensis_ENAM PIESRGYYLNNAIGPEESTLFPSWNSW-DRRIQAQGQKERRPYFNRNTWDQATHLQKTPA 775

Erinaceus_europaeus_ENAM PVESRGYYASNAIGQEESSPFPSWNSW-TNRIQAQGQKERDPYFNRNFWDQSTNVYNAPT 777

Suncus_etruscus_ENAM PVENRGYYPNNAIGQEESSLFPSWSSWGDHRTQAQVEKERGPYFNRNFWDQSTSLHKGSP 769

Echinops_telfairi_ENAM PVESRGYYMENAVPPEESPVFPSWDSW-DRRIQTDGPKERGQYVNRNFWDQTTNVYNAPA 781

* . : . : . :.

Acanthocercus_cyanogaster_ENAM_Acr DPSSQKET-SLHFNSYSTDFRRKSDDDIDDTGKSTRVSAPVSSVDVAGRKHYPDTARYSI 805

Acanthocercus_minutus_ENAM_Acr DPSSQKET-SLHFNSYSTDFRRKSDYDIDDTGKSTHVSAPVSSVDVAGRRHYPDTARYSI 800

Xenagama_zonura_ENAM_Acr DPSSQKET-SLHFNSYSTDFRRKSDYDIDDTGKSTHVSAPVSSVDVAGRRHYPDTARYSI 800

Agama_doriae_ENAM_Acr DPSSQKET-SLYFNTYSTDFRRKSDDDVDDTGKSARVSAPASSVDVAGRRHYPDTGRYPK 805

Laudakia_wui_Acr GPSSRKET-SLYFNAYSTDFRRKSDDDIDDTGKTTHASAPISSVDAAGRRHYPDTVRYSK 812

Phrynocephalus_forsythii_Acr GPSSQKETSSLYFNAYSTDFRRKSDDDIDDTGKSTRVSAPVSNVNVAGRRHYPDTARYSK 804

Phrynocephalus_guinanensis_Acr GPSSQKET-SLYFNAYSTDFRRKSDDDIDDTGKSTRVSAPVSSVNVAGRRHYPDTAKYSK 799

Phrynocephalus_putjatai_Acr GPSSQKET-SLYFNAYSTDFRRKSDDDIDDTGKSTRVSAPVSSVNVAGRRHYPDTAKYSK 803

Phrynocephalus_vlangalii_Acr GPSSQKET-SLYFNAYSTDFRRKSDDDIDDTGKSTRVSAPVSSVNVAGRRHYPDTARYSK 804

Phrynocephalus_versicolor_Acr GPSSQKET-SLSFNAYSTDFRRKSDDDIDDTGKSTHVSAPVSSVNVAGRRHYPDTAKYSK 804

Intellagama_lesueurii_Acr DPSPQKET-SPYFNAYSTDLRRKSDDDIDDTGKITHVSAPLSSINVAGRRHYPDTTRYSE 812

Pogona_vitticeps_Acr GPSPQKET-SPYFNAYSTDLRRKSDDDIDDTGKITHGSAPLSSVNVAGERHYPDTRRYSE 813

Bradypodion_pumilum_Acr DPFPRKET-LPYFNTHSTDFRRKSDDDTDDVSKITQGSAPISGVNVAGRKYNPNTLRYSE 795

Bradypodion_ventrale_Acr DPFPQKET-LPYFNTHSTDFRRKSDDDTDDVSKITQGSAPISGVNVVGRKYNPNTLRYSE 795

Chamaeleo_calyptratus_ENAM_Acr NPFPQKET-LPYFNTYSTDFRRKSEDDTDDASKITQGSAPVSGVNVAGRN-NPDTLRYSE 778

Chamaeleo_dilepis_ENAM_Acr DPIPQKET-LPYFNTYSTDFRRKS----DDASKITQGSVPVSGVNVAGRRYNPDTLRYSE 776

Chamaeleo_gracilis_ENAM_Acr DPFPLKET-LPYFNTYSTDFRRKSEDDTDDASKIALGSATVSGVNVAERRYNPDTLRYSE 779

Chamaeleo_laevigatus_ENAM_Acr DPFPQKET-LPYFNTYSTDFRRKSDDDTDDASKITQGSAPVSGVNVAGRRYNPDTLRYSE 773

Furcifer_pardalis_Acr DPFPQKET-LPYFNTYSTDFRRKSDDDTDDARKITQGSAPVSSVNVAGRRYNPDTLRYSE 778

Trioceros_affinis_ENAM_Acr DPFPQKET-LPYFNTYSTDFRRKSGGDTDDASKITQGSAPISGVNVAGRRYNPDTLRYSE 778

Trioceros_balebicornutus_ENAM_Acr DPFPRKET-LPYFNTYSTDFRRKSDSDTDDASKITQGSAPVSDANVAGRRYNPDTLRYSE 778

Trioceros_harennae_ENAM_Acr DPFPRKET-LPYFNTYSTDFRRKSDGDTDDASKITQGSAPVSDVNVAGRRYNPDTLRYSE 778

Anolis_apletophallus_Pleu DPSAQKET-APYFNIYSTDFRG-NPTHAEDKERIMHVNAPMFTENAPGRRHYLDRMGYSD 808

Anolis_tropidonotus_ENAM_Pleur DPSAQKET-APYFNIYSKDFRR-NPTHAEDKERIIHVNAPIFTENAPGRRHYLDRMGYSE 812

Anolis_sagrei_ordinatus_Pleu DPSAQKET-GPYFNIYSTDFRG-NPTHVEDRERIIHVNAPISTENAPGRRHYLDRMGYSD 812

Anolis_carolinensis_Pleu DPSAQKET-APYYNIYSTDFRG-NPTHVEDRERIIHMNAPISTENAPRRRHYLDRMGYSD 813

Ctenosaura_bakeri_Pleu DPSAQKGT-PPYFNIYSTDFRR-NPTHTEDTREIIRVNTPFSRENVPGGRHYLDRTGYSD 823

Cyclura_pinguis_Pleu DPSAQKGT-PPYFNIYSTDFRR-NPTHAEDTRGIIGMNAPLSGENVPGGRHYLERTGYSD 823

Iguana_delicatissima_Pleu DPSAQKGT-QPYFNIYSTDFRR-NPTHAEDTRGIIHVNAPLSGENVPGGTHYLDRTGYSD 822

Gambelia_wislizenii_Pleu DPSAQKES-LPYLNIYSTDFRR-KPTHAEDTGGVIHLNAPISSENAPGRRHYLERRGYSD 823

Laemanctus_serratus_ENAM_Pleur DPSAQKES-PPYFNVYSTDLRR-NPAHVEDPGGIIHVNAPISSENGPGRRHYLDRMGYSD 824

Phrynosoma_blainvillii_Pleu DLSAQKET-PTYFNIYSTDIRR-NPTHAEDTGGIIHANAPISSENAPGRRHYLGRMGYSD 828

Phrynosoma_platyrhinos_Pleu DLSAQKET-PPYFNIYSTDIRR-NPTHAEDTGGIIHANTPISSENTPGRRHYLGRMGYSD 831

Sceloporus_chrysostictus_ENAM_Pleur DQSAQKET-PPYFNIYSTDVRR-NPTQAEDTGGIIHVNAPISSDNAPGRRHYLDRMGYSD 825

Urosaurus_nigricaudus_Pleu DPSAQKET-PPYFNIYSTDVRR-NPTHAEDTGGIIHVNAPISSENAPGRRHYLDRMGYSD 831

Sceloporus_occidentalis_Pleu DPSAQKET-PPYINIYSTDVRR-NPTHAEDTGGIIHVNAPISSENAPGRRHYLDRMGYSD 831

Sceloporus_tristichus_Pleu DPSAQKET-PPYINIYSTDIRR-NPTHAEDTGGIIHVNAPISNENAPGRRHYLDRMGYSD 831

Sceloporus_undulatus_Pleu DPSAQKET-PPYINIYSTDIRR-NPTHAEDTGGIIHVNAPISNENAPGRRHYLDRMGYSD 831

Canis_lupus_dingo_ENAM SPPHQKEN-QPYPSNSPAGLQK-NPTWRE--GENLNYGMQITRLNSPEGEHLAFPDLIPP 832

Vulpes_vulpes_ENAM SPPHQKEN-QPYPSNSPAGLQK-NPTWRE--GENLNYGMQITRLNSPEGEHLAFPDLIPP 831

Meles_meles_ENAM SPPHQKEN-QPYPSNSPAGLQK-NPTWHE--GENLNYGMQITRINPPEREHLAFPNLIPP 838

Mustela_putorius_ENAM SPPHQKDN-QPYPSNSPAGLQK-NPTWHE--GENLNYGMQITRLNPPEREHPASPNLIPP 837

Ursus_arctos_ENAM SPPHQKEN-QPYPSNSPAGLQK-IPTWRE--GENLNYGMQITRLNSPDGEHLAFPDLIPP 836

Phyllostomus_hastatus_ENAM SPPHQKEN-QPYPI-SPAGLQR-NPTWHE--GENLNYGMQITRLNSPEREHLAFQDLLPP 837

Phacochoerus_africanus_ENAM SSPHQKDN-QPYSNNSPAGLQK-NPTWHE--GENLNYGMQITRLNSPERDHLAFPDLIPP 829

Homo_sapiens_ENAM RPPDQKGN-QPYYSNTPAGLQK-NPIWHE--GENLNYGMQITRMNSPEREHSSFPNFIPP 831

Saimiri_boliviensis_ENAM RPPDQTGN-QPYSSNTPAGLRK-NPIRHE--GENLNYGMQITRINSPEREHSSFPDFIPR 831

Erinaceus_europaeus_ENAM NPPHQKEN-QPYSSNYPAGLQK-NPTWNE--GENLNYGMQNTRLNSPEREHSSFPDLIPS 833

Suncus_etruscus_ENAM IPPHPKEN-QPHSSNSPAGLQK-NPTWHE--GETVNYDMQMTRLNAPEREQPSFPTLIAQ 825

Echinops_telfairi_ENAM NPLDHRQN-QPYSSNSPAGLRK-NPTWLE--DETLNHGMQMTRLNSPDGEQVAFPSLMSQ 837

. . ..: : . : .

Acanthocercus_cyanogaster_ENAM_Acr HYPGRDRTITG-DSPAEPLCCGDDSPVTNEGRPVPLR-----RFPPWE-GTVSTAYSEGS 858

Acanthocercus_minutus_ENAM_Acr HYPGRDRTITG-DSPAEPLCCGDDSPVTNEGRPAPLR-----RFPPWE-GTVSTAYSEGS 853

Xenagama_zonura_ENAM_Acr HYPGRDRTITG-DSPAEPLCCGDDSPVTNEGRPAPLR-----RFPPWE-GTVSTAYSEGS 853

Agama_doriae_ENAM_Acr SYPGGDRTITG-DSPADHLCCGDDSPVMNEGRPAPLRNVPQFRFPPWE-ETVNTAYSEGS 863

Laudakia_wui_Acr DYPGEDRTITG-HSPENHLCCADDSPATKEGRPVPLRSAPQFRFPPWE-ETVSTTYSEGS 870

Phrynocephalus_forsythii_Acr DYPGEDRTITG-PSPADHLCCADDSPVMKEGRPAPLRSAPQFRFPPWE-ETVSAAYSEGS 862

Phrynocephalus_guinanensis_Acr DYPGEDKTITG-PSPADHLCCADDSPVMKEGRPAPLGSAPQFRFPPWE-ETVSTAYSEGS 857

Phrynocephalus_putjatai_Acr DYPGEDKTITG-PSPADHLCCADDSPVMKEGRPAPLGSAPQFRFPPWE-ETVSTAYSEGS 861

Phrynocephalus_vlangalii_Acr DYPGEDTTITG-PSPADHLCCADDSPVMKEGRPAPLRSAPQFRFPPWE-ETVSTAYSEGS 862

Phrynocephalus_versicolor_Acr DYPGEDKTITG-PSPADHLCCADDSPVMKEGRPAPLRSAPPFRFPPWE-ETVRTAYSEGS 862

Intellagama_lesueurii_Acr DYRGEHRTITA-HSTADHLCCGEDSPVTKEGIPAPLRSAPQFRPATWE-EKISTAYSEGS 870

Pogona_vitticeps_Acr DYRGEYRTITT-HSTADHLCCAEDSPVTKEGLPAPLRSAPQFRLAPWE-EKISAGYSEGS 871

Bradypodion_pumilum_Acr DYPREHRTTHP-HSTANHLCCVGDSPVMKEGLPAPLRSAPQFRLALWG-QTESSAYPEGS 853

Bradypodion_ventrale_Acr DYPREHRTTYP---TANHLCCVGDSPVMKEGLPAPLRSAPQFRLALWG-QTESSAYPEGS 851

Chamaeleo_calyptratus_ENAM_Acr DYPREHRTINP----TNHLCCVGDSPVTKEGLPAPLRSAPQFRLALWG-QTESSAYPEGS 833

Chamaeleo_dilepis_ENAM_Acr DYPREHRTINP----TNHLCCVGDSPVTQEGLPAPLRSAPQFRLALWG-QTESSAYPEGS 831

Chamaeleo_gracilis_ENAM_Acr DYPREHRTINP----TNHLCCVGDSSVTKEGLPAPLRSAPQFRLALWG-QTESSAYPEGS 834

Chamaeleo_laevigatus_ENAM_Acr DYPREHRTITP----TNHLCCVGDSPVTKEGLPAPLRSAPQFRLALWG-QTESSAYPEGS 828

Furcifer_pardalis_Acr DYPREHRTATP-HSTANHLCCVGDSPMTKEGLPAPLRSAPQFRLALWG-QTESSAYPEGS 836

Trioceros_affinis_ENAM_Acr DYPREHRTATP-HSTANHLCCVGDSQVTKEGLPTPLRSAPEFRFALWG-QTESSAYPEGS 836

Trioceros_balebicornutus_ENAM_Acr DYPREHRTATP-HSTANHLCCVGASQVTKEGLPTPLRSAPESRFALWG-QTESYAYPEGS 836

Trioceros_harennae_ENAM_Acr DYPREHRTATP-HSTANHLCCLGDSQVTKEGLPTPLRSAPESRFALWG-QTESSAYPEGS 836

Anolis_apletophallus_Pleu DYPKERRMITS-QSTTNQLCCADDSPVRRENRLAPLRSASQFRPVLWE-PKESATYLDDS 866

Anolis_tropidonotus_ENAM_Pleur DYPKGRRMITS-QSTTNQLCCADDSPVRRENRLAPLRSASQFRHALWE-PKESSTYPDDS 870

Anolis_sagrei_ordinatus_Pleu DYPKERRMITS-QSTTNQLCCADDSPVRRENRLAPLRSAPQFRHVLWE-PKESSTYPDDS 870

Anolis_carolinensis_Pleu DYPKERRMITS-QSTANQLCCADDSPMPRENRLAPLRSAPQFRHALWE-AKESSTYPDGS 871

Ctenosaura_bakeri_Pleu DYPTEHRMMTS-HSTPNQLCCADDPPVPRENRLAPLRNAPQFRLASWE-QKESPIYSEGS 881

Cyclura_pinguis_Pleu DYPTERRMMTS-HSTPNQLCCADDSPVPRENRLAPLRNAPQFKLASWE-QKESPIYSEGS 881

Iguana_delicatissima_Pleu DYPTERRTMTS-HSTPNQLCCADDSPVPRENRLAPLRNAPQFRLASWE-QKESPIYSEGS 880

Gambelia_wislizenii_Pleu DYPREGRMMTS-HSTANQLCCAEDSPVPRENKLAPLRSAPQFRLASWE-QKESSTYPEGS 881

Laemanctus_serratus_ENAM_Pleur DYPNERRLITS-HSRTNQLCCADDSPVPRENRLAPLRSAPAFRLASWE-QKESSTYPESS 882

Phrynosoma_blainvillii_Pleu DYQTERRMMTS-YSTANQLCCADDSPVPRENKLAPLKNAPQFRLASWE-QKESSIYPEGT 886

Phrynosoma_platyrhinos_Pleu DYQTERRMMTS-YSTANQLCCADDSPVPRENK---LKNAPQFRLASWG-QKESSIYPEGS 886

Sceloporus_chrysostictus_ENAM_Pleur DHLTERRMMTS-HSIANPLCCADDPPVPRENKLAPLRSAPQFRLASWE-QKESSIYPEGG 883

Urosaurus_nigricaudus_Pleu DYLPERRMMTS-HSIANQLCCADDPPVPRENKLAPLRSAPQFRLASWE-QKESSIYPEGS 889

Sceloporus_occidentalis_Pleu DYLTERRMMSS-PSIANQLCCADDPPVPRENKLAPLRSAPQFRLASWE-QKESSIYPEGS 889

Sceloporus_tristichus_Pleu DYLTERRMMSS-PSIANQLCCADDPPVPRENKLAPLRSAPQFRLASWE-QKESSIYPEGS 889

Sceloporus_undulatus_Pleu DYLTERRMMSS-PSIANQLCCADDPPVPRENKLAPLRSAPQFRLASWE-QKESSIYPEGS 889

Canis_lupus_dingo_ENAM SYPAGQKEAHVFHLSQRGSCCAGGSPGHKDNPLALQDYTPPFDLAPGENEDTSPLYTEDS 892

Vulpes_vulpes_ENAM SYPAGQKEAHVFHLSQRGPCCAGGSPGHKDNPLALQDYTPPFDLAPGENEDTSPLYTEDS 891

Meles_meles_ENAM SYPTGQKEAHVFHPSQRGPCCAGGSTGHKDNPLALQDYTPSFGLAPGENQDTNPLYTEDS 898

Mustela_putorius_ENAM SYPAGQKEAHVFHLSQRGPCCAGGSTGHKDNPLALQDYTPSFGLAPGENQDTNPLYTEDS 897

Ursus_arctos_ENAM SYPAGQKEAHVFHLSQRGPCCAGGSTGHKDNALALQDYTPSFGPVPGETQDTSPLYTEDS 896

Phyllostomus_hastatus_ENAM SYTLGQKEAHLFHLRQRSPCCAGGSIGHKDNPLALQDYTPSFGLVPGENQETSPLSTKDS 897

Phacochoerus_africanus_ENAM DYPGGQKESHVFHLSQRGPCCAGGSMWPKNNPLALQDYTQSFGLAPGENPDTSTGYAEDS 889

Homo_sapiens_ENAM SYPSGQKEAHLFHLSQRGSCCAGSSTGPKDNPLALQDYTPSYGLAPGENQDTSPLYTDGS 891

Saimiri_boliviensis_ENAM SYPSGQKEAHLFHLSQRGSCCAGISAGPKDNPLALQDYTPSYGLAPGENQDTSPLYTEGS 891

Erinaceus_europaeus_ENAM NHPAGQKEPHSFHQTQRGPCCASDPTRHKDNPLALQDYTPSFDLAPEENQDTSPMYTEDS 893

Suncus_etruscus_ENAM SHPAGQNEAHSFHLHHRGPCCVMDSMGQMDNPLALQDNTPSFDAAPDENQDTSLLYTEDN 885

Echinops_telfairi_ENAM SYPPEQKEANSFHPSQRSPCCAGGATGPKDNPLALQDYTPSFGLALGENQDTSPLYTEGT 897

: ** . :. ..

Acanthocercus_cyanogaster_ENAM_Acr HGKHARHAPSPAGI---QSNFPFRLSKNQKENLGTFPEGVMALQKDLPCS-KSKLSQDHH 914

Acanthocercus_minutus_ENAM_Acr HGKHARHAPSPAGI---QSNFPFRLGKNQKENLGTFPEGVMGLQKDLPCS-KSKLSQDHH 909

Xenagama_zonura_ENAM_Acr HGKHARHAPSPAGI---QSNFPFRLGKNQKENLGTFPEGVMGLQKDLPCS-KSKLSQDHH 909

Agama_doriae_ENAM_Acr HGKHARHAPSPAGI---QSNFPFRMSKNQKENLGTFPEGVMGLQKNLPCS-KSKLSQD-H 918

Laudakia_wui_Acr HGKHARHAPSPAGI---QSNFPLRMGENQKGNLGTFPEGVVGLQKNPPCS-KSKLSQD-H 925

Phrynocephalus_forsythii_Acr HGKHARHAPSPAGI---QSSFPFRMGKNHKEHLGAFPEGVVGLQKNLPCS-KSKLSQD-H 917

Phrynocephalus_guinanensis_Acr HGKHARHAPSPAGI---QSNFPFRMGKNHKEHLGAFPEGVMGQQKNLPCS-KSKLSQD-H 912

Phrynocephalus_putjatai_Acr HGKHARHAPSPAGI---QSNFPFRMGKNHKEHLGAFPEGVMGQQKNLPCS-KSKLSQD-H 916

Phrynocephalus_vlangalii_Acr HGKHARHAPSPAGI---QSNFPFRMGKNHKEHLGAFPEGVMGLQKNLPCS-KSKLSQD-H 917

Phrynocephalus_versicolor_Acr HGKHARHAPSPAGI---QSNFPFRM-----EHLGTFPEGVMGLQKNLPCS-KSKLSQD-H 912

Intellagama_lesueurii_Acr HGKHARHAPSPAGI---QSNYPFMVGKKQKENLDTFTEEATGVQKNLPCS-KSKLSQD-H 925

Pogona_vitticeps_Acr HGKHARHAPSPAGI---QSNYPFMVGKKQKENLDTFTEEAAGAQKNLPCS-KSKLSQD-H 926

Bradypodion_pumilum_Acr HGKHARHAPFPAGI---QSSQTLLMGKNQKESLGTFIEEVTGLQKKLPC---SKLSQD-D 906

Bradypodion_ventrale_Acr HGKHARHAPFPAGI---QSSQTLLMGKNQKESLGTFIEEVTGLQKKLPCS-KSKLSQD-D 906

Chamaeleo_calyptratus_ENAM_Acr HGKHARHAPFPDGI---RSSQTLLMGKNQKESLGTFIEEVRGLEKKLPCS-KSKLSQD-D 888

Chamaeleo_dilepis_ENAM_Acr HGKHARHAPFPDGI---RSSQTLLMGKNQKESLGTLIEEVTGLEKKLPCS-KSKLSQD-D 886

Chamaeleo_gracilis_ENAM_Acr HGKHARHAPFPDGI---RSSQTLLMDKNQKESLGTLIEEVTGLEKKLPCS-KSKLSQD-D 889

Chamaeleo_laevigatus_ENAM_Acr HGKHARHAPFPDGI---RSSQTLLVGKNQEESLGALI-EVTGLEKKLPCS-KSKLSQD-D 882

Furcifer_pardalis_Acr HGKHARHAPFPAGI---QSSQTLLMGKNQKESLGTFIEEVKGLQKKLPCS-KSKLSQD-G 891

Trioceros_affinis_ENAM_Acr HGKHARHAPFPAGI---QSSQTLLMGKNQKESLGTFIEEVTGLQKKLPCS-KSKLSQD-D 891

Trioceros_balebicornutus_ENAM_Acr HGKHARHAPFPAGI---QPSQTLLMGKNQKESLGTFIEEVTGLQKKLPCS-KSKLSQD-D 891

Trioceros_harennae_ENAM_Acr HGKHARHAPFPAGI---QSSQTLLMGKNQKESLGTFIEEITGLQKKLPCS-KSKLSQD-D 891

Anolis_apletophallus_Pleu HANYVRHAHSPAGI---QGNNPLKNEGRQREELGAFRMENAGSEKSPPCS-NSHLSQD-S 921

Anolis_tropidonotus_ENAM_Pleur YANYVRHAHSPAGI---QGNNPLKNEGRQREELGAFRMENAGSEKSPPCS-NSHLSQD-S 925

Anolis_sagrei_ordinatus_Pleu HSNYVRYAHSPAGI---QGNNPLKNGGREREELGTFRVENAGSENSPPCS-NSHLSQD-S 925

Anolis_carolinensis_Pleu HANYVRHAHSPAGI---QANNPLKNGGREQEELGAFRVENAGSEKSLPCS-NSHLSQD-S 926

Ctenosaura_bakeri_Pleu HAKYARHAPSPAGI---QANHPLKNGEKEQEELGAFRVENADFEKSPPCS-SAPLSKD-S 936

Cyclura_pinguis_Pleu HAKYARHAPYPAGI---QANHPLKNGEKEQEELGAFRVENAGFEKSPPCS-NAPLSKD-S 936

Iguana_delicatissima_Pleu HAKYARHAPSPAGI---QANHPLKNGKKEQEELGAFRVENAGFEKSPPCS-NAPLSKD-S 935

Gambelia_wislizenii_Pleu HAKYVRHAPSPAGI---QANRPLTNRKKEQEELGIFTVEHAGIEKSPPCS-NSQLSQD-S 936

Laemanctus_serratus_ENAM_Pleur HAKYVRHAPSPAGI---QANHPLKNGKKEREEPGLFTMENAGLEKSPPCP-NAQLSQD-D 937

Phrynosoma_blainvillii_Pleu HAKYVRHAPSPVGI---KSNQPLRNGKNEQEKLGAFRVENVDLEKSPPCS-TSQLSQD-S 941

Phrynosoma_platyrhinos_Pleu HAKYVRHAPSPVGI---KSNQPLRNGKNEQEKLGAFRVENVDLEKSPPCS-TSQLSQD-S 941

Sceloporus_chrysostictus_ENAM_Pleur HAKYVRHVPSPAGI---KSNHPLRNEIK-GEELSASRVENVDLEKSPPCS-TSQLSQD-N 937

Urosaurus_nigricaudus_Pleu HAKYVRDAPSPAGI---KSNHPLRNEIKEGEQ-GAFRVENADLEKSLPCS-TPQLSQD-S 943

Sceloporus_occidentalis_Pleu HAKYVRHVPSPAGI---KSNHPLRNGIKEGEELGAFRVENSDLEKSPPCA-TSQLSQD-S 944

Sceloporus_tristichus_Pleu HAKYVRHVPSPAGI---KSNHPLRNGIKEGEELGAFRVENADLEKSPPCA-TSQLSQD-S 944

Sceloporus_undulatus_Pleu HAKYVRHVPSPAGI---KSNHPLRNGIKEGEELGAFRVENADLEKSPPCA-TSQLSQD-S 944

Canis_lupus_dingo_ENAM HANHARDTISPASNLPGQRNSSEKRMPAESQNLSPFRDDVSTLRRNTPCSMKNQLSQR-G 951

Vulpes_vulpes_ENAM HANHARDTISPASNLPGQRNSSEKRMPGESQNLSPFRDDVSTLRRNTPCSMKNQLSQR-G 950

Meles_meles_ENAM HTKHGRHTISPTSNLPDQRNSSEKRLPGESQNPNSFRDDVSTLRRNTPCSMKNQLSQR-G 957

Mustela_putorius_ENAM HTKHARHTISPTSNLPDQRNSSEKRLPGESQNPNSFRDDVSTLRRNTPCSMKNQLSQR-G 956

Ursus_arctos_ENAM HTKHARHTISPTSNLPDQRNSSEKRLPGESQNPSPFRDDVSTLRRNTPCSMKNQLSQR-G 955

Phyllostomus_hastatus_ENAM HAKHARHTVSPTSILPTQTNSSEKRQPVESQNPSPFRDDVSTLRRNMPCSIKNQLDQR-G 956

Phacochoerus_africanus_ENAM HIKYARQTVSPTSIVPGQRNSSEKILPGESQNPSPFKDDVSTLRRSTPCSVKSQLSQR-G 948

Homo_sapiens_ENAM HTKQTRDIISPTSILPGQRNSSEKR---ESQN--PFRDGVSTLRRNTPCSIKNQLGQK-E 945

Saimiri_boliviensis_ENAM HTKHTRHVVSPTNILPVQRNSSEKR---ESQSPSPFRDDVSTLRRNTPCSIKNQLGQK-G 947

Erinaceus_europaeus_ENAM HTKHARHTISPTSSLPGQRNSSEKRPPTESQNPSPFREDVSTPRRNTPCSMKNQLGQR-G 952

Suncus_etruscus_ENAM HTKHPRHTISPTSILPNQRNSSEKTLPEEIENPSHFRDDVSTLKRNTPCSLKH------- 938

Echinops_telfairi_ENAM HTKHARHIVSPTSVLPGQRNSSEKRLPVESQNPSPFRDDVATLRRNTPCSIKNQLDQR-G 956

: : * * . : . . ... **

Acanthocercus_cyanogaster_ENAM_Acr THGTNCDTGLPSSKNIRDYGNSLRGDRH-G-IPAGIMGERE-SRRELEEAALRLT---PE 968

Acanthocercus_minutus_ENAM_Acr THGANCDTGLPSSKNIRDYGNSLRGDRH-S-IPAGIMGERE-SRRDLEEAALRLT---AE 963

Xenagama_zonura_ENAM_Acr THGANCDTGLPSSKNIRDYGNSLRGDRH-S-IPAGIMGEKE-SRRELEEAALRLS---PE 963

Agama_doriae_ENAM_Acr VHETNCKIGLPSSQNTHGYGNSLRGDTH-S-IPAGIVGERE-SRREMEEAALKLT---AE 972

Laudakia_wui_Acr VHETNCETGLPPSKNTHDYGNSLRGDRH-S-IPAEIMGERE-SRRELEEAALKLV---PE 979

Phrynocephalus_forsythii_Acr VHETNCDTGLPPSQNTHDYGNSLRGDRHSS-IPAGIMEERE-SRRELEEAAVKLI---PE 972

Phrynocephalus_guinanensis_Acr VHETNCDTGLPPSQKTHDYGNSLRGDRHSS-IPAGIMEERE-SRRELEEAAVKLI---PE 967

Phrynocephalus_putjatai_Acr VHETNCDTGLPPSQKTHDYGNSLRGDRHSS-IPAGIMEERE-SRRELEEAAVKLI---PE 971

Phrynocephalus_vlangalii_Acr VHETNCDTGLPPSQNTHDYGNSLRGDRHSS-IPAGIMEERE-SRRELEEAAVKLI---PE 972

Phrynocephalus_versicolor_Acr VHETNCETGLPPSQNTHDYGNSLRGDRHSS-IPAGIMEERE-SRREQEEAAVKLI---PE 967

Intellagama_lesueurii_Acr VHETNCETGLPPSKNTHDYGNSLRGDRH-S-IPDAIMGESE-SRREFRQAALKLV---PE 979

Pogona_vitticeps_Acr THETNCETGLPPSENTHDYGNSLRGDRH-S-IPAAIMGESE-SRREFRQAAVKLV---PE 980

Bradypodion_pumilum_Acr LHETNCDTGLPALKSTHNYGNNLRGDRH-S-IPVQMMEERE-SRWELGQADLKLV---PE 960

Bradypodion_ventrale_Acr LHETNCETGLPALKSTHNYGTNLRGDRH-S-IPVQMMEERE-SRWELGQADLKLV---PE 960

Chamaeleo_calyptratus_ENAM_Acr LHETNCETGLPALKSTHNYGNNLRGDRQ-S-IPVQMMEERE-SRRELGQADLKLV---PE 942

Chamaeleo_dilepis_ENAM_Acr LRETNCETGLPALKSTHNYGNNLRGDRH-S-IPVQMMEERE-SRRELGQADLKLV---PE 940

Chamaeleo_gracilis_ENAM_Acr LRETNCETGLSALKSMHNYGSNLRGDRH-S-VPVQMMEERE-SRRELGQADLKLV---PE 943

Chamaeleo_laevigatus_ENAM_Acr LRETNCETGLPALKSMHNHGNNFRGDRY-S-IPVQMMEERE-SRRELGQADLKLV---PE 936

Furcifer_pardalis_Acr LHETNCETGLPALKSMHNYGNNLRGDRH-S-IPVQMMEERE-STRELGQADLKLV---PE 945

Trioceros_affinis_ENAM_Acr LHETNCETGLPALKRLHNYGNSLRGDRH-S-IPVQMMEERE-FRRELGQADLKLV---PE 945

Trioceros_balebicornutus_ENAM_Acr LHETNCETGLPALKRLHNYGNSLRGDRH-S-IPVQMMEERE-SRRELGQADLKLV---PE 945

Trioceros_harennae_ENAM_Acr LHETNCETGLPALKRLHNYGNSLRGDRH-S-IPVQMMEESE-SRRELGQADLKLV---PE 945

Anolis_apletophallus_Pleu KQEANFQRGSFQFRNMPCHGSNIRGDRH-N-PLAHLVGTGQ-SKREFGKATLNFL---PE 975

Anolis_tropidonotus_ENAM_Pleur KQEANFQRGSFQFRNMHCHGSNMRGDRH-N-PLAHLVGTGQ-SKREFGKSTLNYL---PE 979

Anolis_sagrei_ordinatus_Pleu KQEANFQRGLFQFRNMPCHGSNLRGDRH-N-PLAYLVGTGQ-SKREFGKATLNFL---PE 979

Anolis_carolinensis_Pleu KQEANLQRGLFKFRNMPCHGSNIRGDRH-N-PLAHLVGTGQ-S---FGRGTLNFL---PE 977

Ctenosaura_bakeri_Pleu KQEANFQSGLYQLRNMPCHGGSIRGDRH-N-ILAHLVGTSQ-SKREFEKTASKLL---PE 990

Cyclura_pinguis_Pleu KQEANFQSGLHQLRNLPCHGGSIRGDRH-N-ILAHLVGTSQ-SKREFEKTASKFL---PE 990

Iguana_delicatissima_Pleu KQEANFQSGLHQLRNMPCHGGSIRGDRH-N-ILAHLVGTSQ-SKREFEKTGSKLL---PE 989

Gambelia_wislizenii_Pleu KQEANFQNGLFQLRNIPCHGSSIRGDRH-H-FLAHLVGTSQ-SKKEFEKAALKLP---PE 990

Laemanctus_serratus_ENAM_Pleur KQEANFQNGLFQLRNMPCHGSSIRGDRH-N-ILAHLVGTSQ-SNREFEKSALKHL---PQ 991

Phrynosoma_blainvillii_Pleu KQEANFQSGLFQLRNMPCHGSTIRGDRH-D-ILAHLEGTSQ-SKRELERVPLKLL---PE 995

Phrynosoma_platyrhinos_Pleu KQEANFQSGLFQLRNMPFHGSTIRGDRH-D-ILAHLEGTSQ-SKRELERVPLKLF---PE 995

Sceloporus_chrysostictus_ENAM_Pleur KLEANFENGLREFRNMPCHGSTVRGDRH-N-ILAHLVGTSQ-SRRQSEKAPLKLL---FE 991

Urosaurus_nigricaudus_Pleu KVEANFQNGLRELRNMPCHGSTVRGDRH-N-ILARLVGTGQ-SKRQSEKASLKLL---FE 997

Sceloporus_occidentalis_Pleu KLEANFQNGHRELGNMPCHGSTIRGDRH-N-ILAHLVGTSQ-SKRQSEKAPLKLL---FE 998

Sceloporus_tristichus_Pleu KLEANFQNGHRELGNMPCHGSTIRGDRH-N-ILAHLVGTSQ-SKRQSEKAPLKLL---FE 998

Sceloporus_undulatus_Pleu KLEANFQNGHRELGNMPCHGSTIRGDRH-N-ILAHLVGTSQ-SKRQSEKASLKLL---FE 998

Canis_lupus_dingo_ENAM IMPFPEAGSL-QSKNTPCLTSDLGGDGN-N-VLEEIFEDNQLSERTVDLTPEQLVIGTPD 1008

Vulpes_vulpes_ENAM IMPFPEAGSL-QSKNTPCLTSDLGGDGN-N-VLEEIFEDNQLSERTVDLTPEQLVIGTPD 1007

Meles_meles_ENAM IMPFPEASSL-QSKNTPCLTNDLGEDGN-K-VLEPIFEGNQFNERTVDLTPEQLVIGTPD 1014

Mustela_putorius_ENAM IMPFPEASSL-QSKNTPCHTSDL-GDGN-E-VLEPIFEGNQFNERTVDLTPEQLVIGAPD 1012

Ursus_arctos_ENAM IMPFPEATSL-QSKNMPCLTSDLGGDGN-N-VLEQIFEDNQFDERTADLTPEQLVIGSPD 1012

Phyllostomus_hastatus_ENAM IMPFSEASSL-QPKNTPCLKNDPGGEGN-N-ILEQIFEGNQLNERTVDLTPEQLVIGTPD 1013

Phacochoerus_africanus_ENAM IMPLPEANSL-QSKNTPCLTSDLGGDGN-N-VLEQIFEGNQLNERTVDLTPEQLVFGTPD 1005

Homo_sapiens_ENAM IMPFPEASSL-QSKNTPCLKNDLGGDGN-N-ILEQVFEDNQLNERTVDLTPEQLVIGTPD 1002

Saimiri_boliviensis_ENAM IMPFPEAGSL-QSKNTPCLKNDLGGDGN-N-ILEQIFEDNQLSERTIDLTPEQLVIGTPD 1004

Erinaceus_europaeus_ENAM IMPFPEASSL-QSKNIPCLKSDIVGDGN-NLVLEQIFEDNQLN---VGLTPEQLVMDTAE 1007

Suncus_etruscus_ENAM ----PKANSL-PLENTPCDKND-HGNGN-N-VLEEIFEDNQFIERNVGLTPERLVINTVD 990

Echinops_telfairi_ENAM ILPFP------QSKSTPCLKSDFGGDGT-N-IVDQMFEGSQPNERTADLTPEQLVLGTPD 1008

: : : . :

Acanthocercus_cyanogaster_ENAM_Acr KSPQPQRLRSEALADENGRKEQHPALEIKRIPCFASWLKQY-LSSTGAPSGDPQHDLLHG 1027

Acanthocercus_minutus_ENAM_Acr KSPQPQRLRSEALADENGRKEQHPALEIKRIPCFASWLKQY-LSSTGAPSGDQQHKLLHG 1022

Xenagama_zonura_ENAM_Acr KSPQPQRLRSEALADENGRKEQHPALEIKRIPCFASWLKQY-LSSTGAPSGDQQHKLLHG 1022

Agama_doriae_ENAM_Acr KSPQPQRMRSESLADENGRKEQHP--KIKRIPCFASWLKQY-LSSTGAPSGDQQHNLLHG 1029

Laudakia_wui_Acr KSPEPQRIRSEALGGENGRKEQHAALEIKRIPCFASWLKQY-LSSTGAPSGDQQHNLLHG 1038

Phrynocephalus_forsythii_Acr KSPEPQRIRSEAIAGENGRKEQHAALEIKRIPCFATWIKQY-LSSTGAPSGDQQHNLLHG 1031

Phrynocephalus_guinanensis_Acr KSTEPQRIRSKALAGENGRKEQHAALEIKRIPCFASWLKQY-LSSTGAPSGDQQHNLLHG 1026

Phrynocephalus_putjatai_Acr KSTEPQRIRSKALAGENGRKEQHAALEIKRIPCFASWLKQY-LSSTGAPSGDQQHNLLHG 1030

Phrynocephalus_vlangalii_Acr KSPEPQRIRSEALAGENGRKEQHAALEIKRIPCFASWLKQY-LSSTGAPSGDQQHNLLHG 1031

Phrynocephalus_versicolor_Acr KSPEPQRIRSEALAGENGRKEQHATLEIKRIPCFASWLKQY-LSSTGAPSGDQQHNLLHG 1026

Intellagama_lesueurii_Acr KSPEPQRIRSEALASEDGRKEQHAALEIKRIPCFASWLKQY-LSSTGAPSGDQQHNLFHG 1038

Pogona_vitticeps_Acr KSPEPQRIRSQALASEDGRKEQHAALEIKRIPCFASWLKQY-LSSTGAPSGDQQHNLFHG 1039

Bradypodion_pumilum_Acr TFPGLQRIRSETLGNEDGRKEQHAAIEVKRIPCFASWLKHY-LSSTGAPSGDQKHNLFHG 1019

Bradypodion_ventrale_Acr TFPGLQRIRSETLGNEDGRKEQHAAIEVKRIPCFASWLKHY-LSSTGAPSGDQKHNLFHG 1019

Chamaeleo_calyptratus_ENAM_Acr TFPGPQMIRSETFGNEDGRKEQHTAVEVKRIPCFASWLKQY-LSSTGAPSGDQKHNLFHG 1001

Chamaeleo_dilepis_ENAM_Acr TFPGPQRIRSETFGNEEGRKEQHAAVEVKRIPCFASWLKQY-LSSTGAPSGDQKHNLFHG 999

Chamaeleo_gracilis_ENAM_Acr TFPGPQRIRSETLGNEDGRKEQHAAVEAKRIPCFASWLKQY-LSSTGAPSGDQKHNLFHG 1002

Chamaeleo_laevigatus_ENAM_Acr TFPGPQRIRSETFGNEDVRKEQHAAVEVKRIPCFANWLKQY-LSSTGAPSGDQKHNIFHG 995

Furcifer_pardalis_Acr TFPGPQRIRSETFGNEDGRKEQHAAVEVKRIPCFASWLKQY-LSSTGAPSGDQKHNLFHG 1004

Trioceros_affinis_ENAM_Acr TFPGPQRIRSETFGNEDGRKEQHAAVEVKRIPCFASWLKQY-LSSTGAPSGDQKHTLFHG 1004

Trioceros_balebicornutus_ENAM_Acr TFPGPQRIRSETFGNEDGRKEQHAAVEVKRIPCFASWLKQY-LSSTGAPSGDQKHNLFHG 1004

Trioceros_harennae_ENAM_Acr TFPGPQRIRSETFGNEDGRKEQHAAVEVKRIPCFASWLKQY-LSSTGAPSGDQKHNLFHG 1004

Anolis_apletophallus_Pleu NFPQSHGIQSEALVSADDGKE-YPSLGTKRIPCFESWLKQY-LSNTEAPAGDQQRDSFYG 1033

Anolis_tropidonotus_ENAM_Pleur KSPQSHGIQSEALVSADDGKE-YASLGTKRIPCFGSWLKQY-LSNTEAPSGDQQRDSFYG 1037

Anolis_sagrei_ordinatus_Pleu KIPQSHGIQSEALVSEDDGKE-YASLGTKRIPCFGSWLKQY-LSNTEAPSGDQQHDPFYG 1037

Anolis_carolinensis_Pleu QFPQPHGIQSEALVSEDDGKE-YAALGAKRIPCFGSWLKQY-LSNTEVPSDDQQRDPFYG 1035

Ctenosaura_bakeri_Pleu RFPQPQEIQSEALLSEDDRKEQHVALGAKRIPCFGSWLKQY-LSSTRAPSGDQQQDPFYA 1049

Cyclura_pinguis_Pleu RLPQPQGIQSEALLSEDDRKEQHVALGAKRIPCFGSWLKQY-LSSTRAPSGDQQQDPFYV 1049

Iguana_delicatissima_Pleu SLPQPQGIQSEALLSEDDRKEQQVALGAKRIPCFGSWLKQY-LSSTRAPSGDQQQDPFYA 1048

Gambelia_wislizenii_Pleu KFPQPQGIQSEALVSEDDRKKQHPALGAKRIPCFGRWLKQY-LSSTGAPSGDQQHDPFYG 1049

Laemanctus_serratus_ENAM_Pleur KLPQPQAIQSEVLVSEVDRKGQHAARAAKKIPCFGNWLKQY-LSSTGAVPGDQPDDPFYG 1050

Phrynosoma_blainvillii_Pleu KFPQSQVIESEALVSEDNRKEQHATLGAKRIPCFGSWLKQY-LSSTVAPSGDQQPDPFYG 1054

Phrynosoma_platyrhinos_Pleu KFPQPQVIQSEALVSEDNRKEQHAALGAKRIPCFGSWLKQY-LSSTGAPSGDQQPDPFYG 1054

Sceloporus_chrysostictus_ENAM_Pleur KFPRPQGIQSEALVSEDNRKEQHAALGAKRIPCFGSWLKQY-LSSTGAPSGDQLPDSFYG 1050

Urosaurus_nigricaudus_Pleu KLPRPQGIQSEALISEDNRKEQHVALEAKRIPCFGSWLKQY-LFSTGAPSGDQQPDSFYG 1056

Sceloporus_occidentalis_Pleu KFPRPQGIQSEALVSEDNRKEQHAALGAKRIPCFGNWLKQY-LSSTGAPSGDRQPNSFYG 1057

Sceloporus_tristichus_Pleu KFPQPQGIQSEALVSEDNRKEQHTALGAKRIPCFGNWLKQY-LSSTGAPSGDRQPVSYYG 1057

Sceloporus_undulatus_Pleu KFPQPQGIQSEALVSEDNRKEQHAALGAKRIPCFGNWLKQY-LSSTGAPSGDRQPNSFYG 1057

Canis_lupus_dingo_ENAM EGPEPEGIQSEVQGNEGDRQQQRPS-SIIQLPCFGSKITNYHSSSTGTPSSIGRQGPFDE 1067

Vulpes_vulpes_ENAM EGPEPEGIQSEVQGNEGDRQQQRPS-SIIQLPCFGSKITKYHSSSTGTPSSIGRQGPFDE 1066

Meles_meles_ENAM EGPQPEGIRSEVQGNEGERQQQRPS-SILQLPCFGSKIAKYYSSSTGTPSSIGRQDPFDE 1073

Mustela_putorius_ENAM EGPPPEGIRSEMQGNEGERQQQRPS-SILQLPCFGSKIAKYHSSSTGTPSSIGRQDPFDE 1071

Ursus_arctos_ENAM EGPQPEGIQSEVQGNEDEGQQQRPS-SILQLPCFGSEIAKYHSSSTGTPSSIGRQGPFDE 1071

Phyllostomus_hastatus_ENAM EGPKPEDIQSEALGNEGERQQQRPS-SILQLPCFGSKLTKYHSSSPGTPSSIGRQSPFNG 1072

Phacochoerus_africanus_ENAM EEPRPEGIPNEMQGNESERQQQRQS-SILQLPCFGSKLANYHTSSIGTPSSLGRQDSFDG 1064

Homo_sapiens_ENAM EGSNPEGIQSQVQENESERQQQRPS-NILHLPCFGSKLAKHHSSTTGTPSSDGRQSPFDG 1061

Saimiri_boliviensis_ENAM EGSDPEGIQSQIQGNESERQQQRPS-NILHLPCFSSKLAKHHSSGIGSPSSNGRQSPFDG 1063

Erinaceus_europaeus_ENAM QDPKPEGIQGEVQVNEGERQQSRPS-SILQLPCFDSRLAKYHSSDIGTPPTIGRQTSFDG 1066

Suncus_etruscus_ENAM EGPKPEGIQSEVSGKDGERQKQRLS-SILQVPCFGSVLAKYHSSSTGSPSSSGRQGPFDE 1049

Echinops_telfairi_ENAM EKSQPEAIQSEVPGSEGERQQQRPS-SIRQLPCFGSKLAKHHFSGTGAPSSNGRQDPFVG 1067

. . : .: : ::*** : :: .

Acanthocercus_cyanogaster_ENAM_Acr EAPFPTSRPNILP--PESKPISETNPSHDDVEGKHLTFSAPGEDWVERPDEATQDCLLLQ 1085

Acanthocercus_minutus_ENAM_Acr EAPFPTSRPNILP--PESKPISSTNPSHDDVEGKHLTFSTPGEDWVERPDEATQDCLLLQ 1080

Xenagama_zonura_ENAM_Acr EAPFPTSRPNILP--PESKPISSTNPSHDDVEGKHLTFSTPGEDWVERPDEATQDCLLLQ 1080

Agama_doriae_ENAM_Acr EAPFPTSKPNSL---PESKPISSTNPSHDDVEGKHLTFSAPGEDWVERPDEATPDCLLLQ 1086

Laudakia_wui_Acr EAPFPTSRPNSLP--PESKPISSTNPSHDDVEGKHLTFSAPGEEWVERPDEATPDCLLLQ 1096

Phrynocephalus_forsythii_Acr EAPFPTSRPNSLP--PESKPISSTNPSHDDIEGKHLTFSALGEEWVERPDEATPDCLLLQ 1089

Phrynocephalus_guinanensis_Acr EAPFPTSRPNSLP--PESKPISSTNPSHDDVEGKHLTFSTLGEEWVERPDEATPDCLLLQ 1084

Phrynocephalus_putjatai_Acr EAPFPTSRPNSLP--PESKPISSTNPSHDDVEGKHLTFSTLGEEWVERPDEATPDCLLLQ 1088

Phrynocephalus_vlangalii_Acr EAPFPTSRPNSLP--PESKPISSTNPSHDDIEGKHLTFSALGEEWVERPDEATPDCLLLQ 1089

Phrynocephalus_versicolor_Acr EAPFPTSRPNSLP--PESKPISSTNPSHGDVEGKHLTFSALGEEWVERPDEATPDCLLLQ 1084

Intellagama_lesueurii_Acr ETPVPTSRPNNMP--PESKPISSTNPSHDDREGKHLKFGSPGEEWVEQPNETTPDCLLLQ 1096

Pogona_vitticeps_Acr ETPVPTSRPNDMP--PESKPISSTNPSHDDREGKHLKFGSPGEEWVERPNETTPDCLLLQ 1097

Bradypodion_pumilum_Acr ETPSPTSRPNNRP--PESKPIASTNPSYDNVEEKHLTFGSPGEEWVEQPNETTPDCLLLQ 1077

Bradypodion_ventrale_Acr ETPSPTSRPNNRP--PESKPIASTNPSYDNVEEKHLKFGSPGEEWVEQPNETTPDCLLLQ 1077

Chamaeleo_calyptratus_ENAM_Acr ETPSPTNRPNSRP--PESKPIASTNPSYDNVEEKHLKFSSPGEEWVEQPNETTPDCLLLQ 1059

Chamaeleo_dilepis_ENAM_Acr ETPSPTSRPDSRP--PESKPIASTHPSYDNVEEKHLKFGSPGEEWVEQPNETTPDCLLLQ 1057

Chamaeleo_gracilis_ENAM_Acr ETPSPTSRPNSRP--PESKPIASTNPSYDNVEEKHLKFSSPGEEWVEQPNETTPDCLLLP 1060

Chamaeleo_laevigatus_ENAM_Acr ETPSPTSRPNSRP--PESKPIASTNPSYDNVEEKHLKFGSPGEEWVEQPNETTPDCLLLQ 1053

Furcifer_pardalis_Acr ETPSPTSRPNSRP--PESKPIASTNPSYDNVEEKHLKFSSPGEEWVEQPNETTPDCLLLQ 1062

Trioceros_affinis_ENAM_Acr ETPSPTSRPNSRP--PESKPIASTNPSYDHVEEKHLKFGSPGEEWVEQPNETTPDCLLLQ 1062

Trioceros_balebicornutus_ENAM_Acr ETPSPTSRPNSRP--PESKPIASTNPSYDNVEEKHLKFGSPGEEWVEQPNETTPDCLLL- 1061

Trioceros_harennae_ENAM_Acr ETPSPTSRPNSRP--PESKPIASTNPSYDNVEEKHLKFGSPGEEWVEQPNETTPDCLLLQ 1062

Anolis_apletophallus_Pleu ENQVPTERPNSVP--PKPEPITSSFLSNG-VEEKPLKINAPGEEWAEQATQSTPDCLLLQ 1090

Anolis_tropidonotus_ENAM_Pleur ENPIPTERPNSAP--PKPEPISSSFLSNG-VEEKPLKINTPGEEWAEEATQSTPDCLVLQ 1094

Anolis_sagrei_ordinatus_Pleu ENLVPTGKPNSVP--PKPEPISSSFLSNG-VEEKPLKINAPGEEWAEQATQSTPDCLLFQ 1094

Anolis_carolinensis_Pleu ENPVPTERPNSIP--PKPEPISSSFLSNG-VEEKPLKINAPEEEWAELATQSTPDCLLLQ 1092

Ctenosaura_bakeri_Pleu ENPVPTGRPNSML--QEPEPISSTYPSQD-KEENPLKINSPGEKWDEPAAQSTPDCLLLQ 1106

Cyclura_pinguis_Pleu ENPVPTGRPNSMP--QEPEPISSSYPSQD-GEEKPLKINSPGEKWDEPAAQSTPDCLLLQ 1106

Iguana_delicatissima_Pleu ENPVPTGRPNSMP--QEPEPISSTYPSQD-GEGKPLKINSPGEKWDEPAAQSTPDCLLLQ 1105

Gambelia_wislizenii_Pleu ENPIPTGRPNSMP--PEPEPISSTYPSPD-IEEKPLKINSPGDEWAEQAAQSTPDCLLLQ 1106

Laemanctus_serratus_ENAM_Pleur EGPVPTGRTSSMP--PDPEPISSTYPSQG-VEEKPLKINSPVEEWAEQAAQSTPDCLLLQ 1107

Phrynosoma_blainvillii_Pleu ENLFPTERPNSMP--PKPEPISSTYPSYD-RGEKPVKINSPGEEWAGQVAQSTPDCLLLQ 1111

Phrynosoma_platyrhinos_Pleu ENLFPTERPNSMS--PDPEPISSTYPSYD-RGEKPVKINSPGEEWAGQVAQSTPDCLLLQ 1111

Sceloporus_chrysostictus_ENAM_Pleur ESLFPTERPNSMP--PEPEPISSTYPSHD-TGEKPVKINSQGEKWAGQAAQSTPDCLLLQ 1107

Urosaurus_nigricaudus_Pleu ESLFPTERPNSRP--PEPEPISSTYPSHD-TGEKPVKINSPGEEWAGQAAQSTPDCLLLQ 1113

Sceloporus_occidentalis_Pleu ESLFPTERPNSMP--PEPEPISSTYPSHD-TGEKPVKINSPGEEWAGQAPQSTPDCLLLQ 1114

Sceloporus_tristichus_Pleu ESLFPTERPNSMP--PEPEPISSTYPSHD-AGEKPVKINSPGEEWAGQAAQSTPDCLLLQ 1114

Sceloporus_undulatus_Pleu ESLFPTKRPNSMP--PEPEPISSTYPSHD-AGEKPVKINSPGEEWAGQAAQSTPDCLLLQ 1114

Canis_lupus_dingo_ENAM EPIMPTENPNSLSRLATGAQFQSINVDPL-SADEHTPFDS--LQLGTNPQDHVQDCLLLQ 1124

Vulpes_vulpes_ENAM EPIMPTENPNSLSRLATGAQFQSINVDPL-SAGEHTPFDS--LQLGTNPQDHVQDCLLLQ 1123

Meles_meles_ENAM EPIMPTESPNSLSRLATGAEFQSINVDPL-NADEHTPFDP--LQLGTNPQDHVQDCLLLQ 1130

Mustela_putorius_ENAM EPIMPTESPNSLSRLATGAEFQSINVDPL-NADEHTPFDP--LQLGTNPQDHVQDCLLLQ 1128

Ursus_arctos_ENAM DPIMPTESPNSLSRLATGAEFQSINVDPL-NADEHTPFDS--LQLGTNPQDHVQDCLLLQ 1128

Phyllostomus_hastatus_ENAM DSLMPTEIPNSLAGLATGTEFQSINVDPL-NADEHTPFDS--LQIGTNPQDQVQDCLLLQ 1129

Phacochoerus_africanus_ENAM DPIMPTETPNSLAGLATGAQFQNINVDPL-NEDEHTPFDS--LQIGTNPQDQVQDCLLLQ 1121

Homo_sapiens_ENAM DSITPTENPNTLVELATEEQFKSINVDPL-DADEHSPFEF--LQRGTNVQDQVQDCLLLQ 1118

Saimiri_boliviensis_ENAM DSIMPTENPNTLVELATGEQFKSINVDPL-DAGEHTPFEF--LQRGTSAQDQIQDCLLLQ 1120

Erinaceus_europaeus_ENAM DPIMPTEIPNSLTGLATGEQFQSINVDTQ-NTDEHPPFDS--LQIGTNPQDQVQDCLLLQ 1123

Suncus_etruscus_ENAM NPIMPTEIPNSLDGLASEAQFQNSNVDPL-NTGEHSPFES--LQIVTNPQDQVQDCLLFQ 1106

Echinops_telfairi_ENAM DPIMPTEKPNTLADLATEAPFNSANVNTL-NEGEHPPLEP--FLKGTDPEDQVQDCLLLQ 1124

: ** .. : . . : : : ***::

Acanthocercus_cyanogaster_ENAM_Acr NE- 1087

Acanthocercus_minutus_ENAM_Acr NE- 1082

Xenagama_zonura_ENAM_Acr NE- 1082

Agama_doriae_ENAM_Acr NE- 1088

Laudakia_wui_Acr NE- 1098

Phrynocephalus_forsythii_Acr NQM 1092

Phrynocephalus_guinanensis_Acr NK- 1086

Phrynocephalus_putjatai_Acr NK- 1090

Phrynocephalus_vlangalii_Acr NK- 1091

Phrynocephalus_versicolor_Acr NK- 1086

Intellagama_lesueurii_Acr KK- 1098

Pogona_vitticeps_Acr KK- 1099

Bradypodion_pumilum_Acr NK- 1079

Bradypodion_ventrale_Acr NK- 1079

Chamaeleo_calyptratus_ENAM_Acr KI- 1061

Chamaeleo_dilepis_ENAM_Acr KK- 1059

Chamaeleo_gracilis_ENAM_Acr KK- 1062

Chamaeleo_laevigatus_ENAM_Acr K-- 1054

Furcifer_pardalis_Acr NK- 1064

Trioceros_affinis_ENAM_Acr NK- 1064

Trioceros_balebicornutus_ENAM_Acr ---

Trioceros_harennae_ENAM_Acr NK- 1064

Anolis_apletophallus_Pleu KK- 1092

Anolis_tropidonotus_ENAM_Pleur KK- 1096

Anolis_sagrei_ordinatus_Pleu KK- 1096

Anolis_carolinensis_Pleu KK- 1094

Ctenosaura_bakeri_Pleu NK- 1108

Cyclura_pinguis_Pleu NK- 1108

Iguana_delicatissima_Pleu NK- 1107

Gambelia_wislizenii_Pleu KK- 1108

Laemanctus_serratus_ENAM_Pleur NK- 1109

Phrynosoma_blainvillii_Pleu NK- 1113

Phrynosoma_platyrhinos_Pleu DK- 1113

Sceloporus_chrysostictus_ENAM_Pleur NK- 1109

Urosaurus_nigricaudus_Pleu NK- 1115

Sceloporus_occidentalis_Pleu NK- 1116

Sceloporus_tristichus_Pleu NK- 1116

Sceloporus_undulatus_Pleu NK- 1116

Canis_lupus_dingo_ENAM A-- 1125

Vulpes_vulpes_ENAM A-- 1124

Meles_meles_ENAM A-- 1131

Mustela_putorius_ENAM A-- 1129

Ursus_arctos_ENAM A-- 1129

Phyllostomus_hastatus_ENAM A-- 1130

Phacochoerus_africanus_ENAM A-- 1122

Homo_sapiens_ENAM A-- 1119

Saimiri_boliviensis_ENAM A-- 1121

Erinaceus_europaeus_ENAM A-- 1124

Suncus_etruscus_ENAM A-- 1107

Echinops_telfairi_ENAM A-- 1125

**ENAM-AA-MAFFT-Iguania+Mammal – DIVERGE-Acro-Pleuro DIVERGE-Agam-Cham**

CLUSTAL W (1.8) multiple sequence alignment (ALTER 1.3.3)

Acanthocercus_cyanogaster_ENAM_Acr MKLVILY-LCLVGTMCATSLRKRRKAGFGSKSEEMMPFGAYGFLNSPQ------------ 47

Acanthocercus_minutus_ENAM_Acr MRLIILY-LCLVGTMCATPLRKRRKAGFGSKSEEMMPFGAYGFLNSPQ------------ 47

Xenagama_zonura_ENAM_Acr MRLIILY-LCLVGTMCATPLRKRRKAGFGSKSEEMMPFGAYGFLNSPQ------------ 47

Agama_doriae_ENAM_Acr MKLILLY-LCLVGTMCATPLRKRRKAGFGSKSEEMMPFGAYGFLNSPQ------------ 47

Laudakia_wui_Acr MKLIVLY-LCLLGTLCAVPLRKRRKAGFGSKSEEMMPFGAYGFLNSPH------------ 47

Phrynocephalus_forsythii_Acr MKPIILY-LCLVGTLCAIPLRKRRKAGFGSKSEEMMPFGAFGFLNSAQ------------ 47

Phrynocephalus_guinanensis_Acr MKPIILY-LCLVGTLCAIPLRKRRKAGFGSKSEEMMPFGAFGFLNSPQ------------ 47

Phrynocephalus_putjatai_Acr MKPIILY-LCLIGTLCAIPLRKRRKAGFGSKSEEMMPFGAFGFLNSPQ------------ 47

Phrynocephalus_vlangalii_Acr MKPIILY-LCLVGTLCAIPLRKRRKAGFGSKSEEMMPFGAFGFLNSPQ------------ 47

Phrynocephalus_versicolor_Acr MKPIVLY-LCLVGTLCAVPLRKRRKAGFGSKSEEMMPFGAFGFLNSPQ------------ 47

Intellagama_lesueurii_Acr MKLIVLY-LCLVGTSCAIPLRKRRKAGFGSKSEEMMPFGAYGFLNSPQ------------ 47

Pogona_vitticeps_Acr MKLFVLY-LCLVGTSCAIPLRKRRKAGFGSKSEEMMPFGAYGFLNSPQ------------ 47

Bradypodion_pumilum_Acr MKLIILC-LCLMGTTCANPLRKRRKAGFGSKSEEMMSFGAYGMLNSPQKWRSDATLFLKI 59

Bradypodion_ventrale_Acr MKLIILC-VCLMGTTCANPLRKRRKAGFGSKSEEMMSFGAYGMLNSPQKWRSDATLFLKI 59

Chamaeleo_calyptratus_ENAM_Acr MKLIILC-LCLMGTNCANPLRKRRKAGFGSKSEEMMPFGAYGMLNTP------------- 46

Chamaeleo_dilepis_ENAM_Acr MKLIILC-LCLMGTNCANPLRKRRKAGFGSKSEEMMPFGAYGMLNTP------------- 46

Chamaeleo_gracilis_ENAM_Acr MKLIILC-LCLMGTNCANPLRKRRKAGFGSKSEEMMPFGAYGMLNTP------------- 46

Chamaeleo_laevigatus_ENAM_Acr MKLIILC-LCLMGTNCANPLRRRRKAGFGSKSEEMMPFGAYGMLNTP------------- 46

Furcifer_pardalis_Acr MKFIILC-LCLMGTTCANPLRKRRKAGFGSKSEEMMPFGAYGMLNSP------------- 46

Trioceros_affinis_ENAM_Acr MKLIILC-LCLMGTTCANPLRKRRKAGFGSKSEEMMPFGAYGMLNSP------------- 46

Trioceros_balebicornutus_ENAM_Acr MKLIILC-LCLMGTTCANPLRKRRKAGFGSKSEEMMPFGAYGMLNSP------------- 46

Trioceros_harennae_ENAM_Acr MKLIILC-LCLMGTTCANPLRKRRKAGFGSKSEEMMPFGAYGMLNSP------------- 46

Anolis_apletophallus_Pleu MKQLLLY-LCLIGTSWAVRLRKPRKAGFGSKSEEMMQFGPYGYLNSPQ------------ 47

Anolis_tropidonotus_ENAM_Pleur MKQLLLY-LCLIGTSWAVRLRKPRKAGFGSKSEEMMQFGPYGYLNSPQ------------ 47

Anolis_sagrei_ordinatus_Pleu MKQLLLY-LCLIGTSWAVRLRKPRKAGFGSKSEEMMQFGPYGYLNSPQ------------ 47

Anolis_carolinensis_Pleu MKQLLLY-LCLIGTSWAVRLRKPRKAGFGSKSEEMMQYGPFGYLNSPQ------------ 47

Ctenosaura_bakeri_Pleu MKRILLY-LCLIGTAWAVLVRKPRKAGFGSKSEEMMQFGPYGYLNSPQ------------ 47

Cyclura_pinguis_Pleu MKRILLY-LCLIGTAWAVLVRKPRKAGFGSKSEEMMQFGPYGYLNSPQ------------ 47

Iguana_delicatissima_Pleu MKQILLY-LCLIGTAWAVLVRKPRKAGFGSKSEEMMQFGPYGYLNSPQ------------ 47

Gambelia_wislizenii_Pleu MKRILLY-LCLIGTSWAVLLRKPRKAGFGSKSEEMMQFGPYGYLNSPQ------------ 47

Laemanctus_serratus_ENAM_Pleur MKRIFLC-LCLIGTSWAVLLRKPRKAGFGSKSEEMMQFGPYGYLNSPQ------------ 47

Phrynosoma_blainvillii_Pleu MMLILLC-LCLTGASWAALLRKPRKAGFGSKSEEMMQFGPYGYLNSPQ------------ 47

Phrynosoma_platyrhinos_Pleu MMRILLC-LCLTGTSWAALLRKPRKAGFGSKSEEMMQFGPYGYLNSPQ------------ 47

Sceloporus_chrysostictus_ENAM_Pleur MTRILLYLLCLIGTSWAALLRKPRKAGFGSKSEEMMQFGPYGYLNSPQ------------ 48

Urosaurus_nigricaudus_Pleu MTRIFLYLLCLIGTSWAALLRKPRKAGFGSKSEEMMQFGPYGYLNSPQ------------ 48

Sceloporus_occidentalis_Pleu MMRILLYLFCLIGTSWAALLRKPRKAGFGSKSEEMMQFGPYGYLNSPQ------------ 48

Sceloporus_tristichus_Pleu MMRILLYLFCLIGTSWAALLRKPRKAGFGSKSEEMMQFGPYGYLNSPQ------------ 48

Sceloporus_undulatus_Pleu MMRILLYLFCLIGTSWAALLRKPRKAGFGSKSEEMMQFGPYGYLNSPQ------------ 48

* ..* .** *: * :*: ************* :*.:* **:.

Acanthocercus_cyanogaster_ENAM_Acr ---LSPFTASLYGYRPNYPQLFPQQPMSPLQRPFLWQPQVPVHDVARLPSQK----PQTP 100

Acanthocercus_minutus_ENAM_Acr ---LSPFTASLYGYRPSYPQLFPQQPMPPLQRPFLWQPQVPVHDVARLPSQK----PQPP 100

Xenagama_zonura_ENAM_Acr ---LSPFTASLYGYRPSYPQLFPQQPMPPLQRPFLWQPQVPVHDVARLPSQK----PQPP 100

Agama_doriae_ENAM_Acr ---LSPFAASLYGYRPNYPQLFPQQPMAPLQRPFLWQPQVP---AARLPSQK----PQPP 97

Laudakia_wui_Acr ---LSPFAASLYGYRPNYLQLFPQQPMSPLQRPFLWQQQVPVHEVTRLPSQK----PQPP 100

Phrynocephalus_forsythii_Acr ---LLPFAASLYGYRPNYPQL--------LQRPFLWQQQVPVHEVARLPSQK----PPPP 92

Phrynocephalus_guinanensis_Acr ---LSPFAASLYGYRPNYPQL--------LQRPFLWQQQVPVHEIARLPSQK----PQPP 92

Phrynocephalus_putjatai_Acr ---LSPFAASLYGYHPNYPQL--------LQRPFLWQQQVPVHEIARLPSQK----PQPP 92

Phrynocephalus_vlangalii_Acr ---LSPFAASLYGYRPNYPQL--------LQRPFLWQQQVPVHEVARLPSQK----PQPP 92

Phrynocephalus_versicolor_Acr ---LSPFAASLYGYRPNYPQL--------LQRPFLWQQQVPVHEVARLPSQK----PQPP 92

Intellagama_lesueurii_Acr ---LSQFAASLYGYRPSYPQLFPQQPMSPLPRPFLWQQQMPVHGATRLPSQNPHRLPQQP 104

Pogona_vitticeps_Acr ---LSQLAASLYGYRPSYLQLFPQQSMPPLSRPSLWQQQMPAHEATRFPSQNPHRLPQQP 104

Bradypodion_pumilum_Acr TFLLSQFAASLYGYRPSFLQMFPQQSMTPLQRPFLWQQQMPGYEAARLSPQK----PQQP 115

Bradypodion_ventrale_Acr TFLLSQFAASLYGYRPSFLQMFPQQSMTPLQRPFLWQQQMPGYEAARLSPQK----PQQP 115

Chamaeleo_calyptratus_ENAM_Acr -----QFAASLYGYRPSFLQMFPQQSMTPLQRPFLWQQPMPGYETTRLSPQK----PQQP 97

Chamaeleo_dilepis_ENAM_Acr -----QFAASLYGYRPSFLQMFPQQSVTPLQRPFLWQQQMPGYEAARLPPQR----PPQP 97

Chamaeleo_gracilis_ENAM_Acr -----QFAASLYGYRPSFLQMFPQQSMTPLQRPFLWQQQMPGYEAARLPPQR----PPQP 97

Chamaeleo_laevigatus_ENAM_Acr -----QFAASLYGYRPSFLQMFPQQSMTPLQRPFLWQQQMPGYEAARLSPQR----PQQP 97

Furcifer_pardalis_Acr -----QFAASLYGYRPSFLQMFPQQPMTPLQRPFLWQQQMPGYGAGRLSPQR----SQQP 97

Trioceros_affinis_ENAM_Acr -----QFAASLYGYRPSFLQMFPQQSTTPLQRPFLWQQQMPGYQATRLSPQR----PQQP 97

Trioceros_balebicornutus_ENAM_Acr -----QFAASLYGYRPSFLQMFPQQSMTPLQRPFLWQQQMPGYQATRLSPQR----PQQP 97

Trioceros_harennae_ENAM_Acr -----QFAASLYGYRPSFLQMFPQQSMTPLQRPFLWQQQMPGYQDTRLSPQR----PRQP 97

Anolis_apletophallus_Pleu ---LTQLAASLYGYRSGFPQMVPQQPAFPLQRFYLWPQQMPVHQSARMPQQK----PHQT 100

Anolis_tropidonotus_ENAM_Pleur ---LTQLAASLYGYRSGFPQMVPQQPAFPLQRFYLWPQQMPVHQSAQLPQQK----PHQT 100

Anolis_sagrei_ordinatus_Pleu ---LTQLAASLYGYRSGFPQMVPQQPAFPLQRFYLWPQQMPVHQSARPPQQK----PPQT 100

Anolis_carolinensis_Pleu ---LTQLAASLYGYRSGFPQMVPQQPTFPLQRFYLWPQQMPVHQSARPPQQK----AHQT 100

Ctenosaura_bakeri_Pleu ---LTQLAASLYGYRSGFPQMVHPQPAFPLQRIYLWQQQMPVHQAAKLPQQK----PHQT 100

Cyclura_pinguis_Pleu ---LTQLAASLYGYRSGFPQMVRPQPAFPLQRIYLWQQQMPVRQAARVPQQK----PHQT 100

Iguana_delicatissima_Pleu ---LTQLAASLYGYGSGFPQMVHPQPAFPLQRIYLWQQQMPVHQAAKLPQQK----PHQT 100

Gambelia_wislizenii_Pleu ---LTQLAASLYGYRSSFPQMVPQQPTFPLQRFYLWQQQMPIHQAAQLPQQK----PHQT 100

Laemanctus_serratus_ENAM_Pleur ---LTQLAASLYGYRSGLPQMVSQQPAFPLQRFYLWQQQMPVHQATRLPQQK----PHQI 100

Phrynosoma_blainvillii_Pleu ---LTQLAASLYGYHSSFPQLVPQQPAFPLKRFYLWQQQMPGHQAARPPQQK----PHQT 100

Phrynosoma_platyrhinos_Pleu ---LTQLAASLYGYHSSFPQLVPQQPAFPLKRFYLWQQQMPGHQAARPPQQK----AHQT 100

Sceloporus_chrysostictus_ENAM_Pleur ---LTQLAASLYGYRSRVPQMVPQQAAFPLQRFYLWQQQMPGHQAARLPQRK----PHQN 101

Urosaurus_nigricaudus_Pleu ---LTQLAASLYGYRSRFPQVVPQQPAFPLQRFYLWQQQMPGHQAARLPQQK----PQQN 101

Sceloporus_occidentalis_Pleu ---LTQLAASLYGYRSGFPQMVPQQPAFPLQRFYLWQQQMPGHQAAKLPQQK----PQQN 101

Sceloporus_tristichus_Pleu ---LTQLAASLYGYRSGFPQMVPQQPAFPLQRFYLWQQQMPGHQAAKLPQQK----PQQN 101

Sceloporus_undulatus_Pleu ---LTQLAASLYGYRSGFPQMVPQQPAFNLQRFYLWQQQMPGHQAAKLPQQK----PQQN 101

::****** . *: * * ** :* : . :. .

Acanthocercus_cyanogaster_ENAM_Acr SVAWKPNSRPQPK----PQQP---------RHQVQQPQVQV-QQPPKPPPGAAGPFQPQP 146

Acanthocercus_minutus_ENAM_Acr SVAWKPNGRPQPK----PQQP---------RHQVQQPQVQV-QQPPKPPPGAAGPFQPQP 146

Xenagama_zonura_ENAM_Acr SVAWKPNGRPQPK----PQQP---------RHQVQQPQVQV-QQPPKPPPGAAGPFQPQP 146

Agama_doriae_ENAM_Acr SVAWKPNGRPQLK----PQQP---------RHQVLQPQVQV-QQPPKLPPGAAGPYQPQP 143

Laudakia_wui_Acr SAAWKPNSRPQPKPQLPPQQP---------RHQVQQPQIQV-QQLPKLPPNAASPLPPQP 150

Phrynocephalus_forsythii_Acr SAAWKPNSRPQPKPPLPPQQP---------RHQVQQPQIQV-QQPPKLPPRAAGPSQPQP 142

Phrynocephalus_guinanensis_Acr SAAWKPNSRPQPKLPLPPQQP---------RHQVQQPQIQV-QQPPKLPPGAAGPSQPQP 142

Phrynocephalus_putjatai_Acr SAAWKPNSRPQPKLPLPPQQP---------RHQVQQPQIQV-QQPPKLPPGAAGPSQPQP 142

Phrynocephalus_vlangalii_Acr SAAWKPNSRPQPKPPLPPQQP---------RHQVQQPQIQV-QQPPKLPPGAASPSQPQP 142

Phrynocephalus_versicolor_Acr SVAWKPNSRPQPKPPLPPQQP---------RHQVQQPQIQV-QQPPKLSPGAATPSHPQP 142

Intellagama_lesueurii_Acr SAARRPNSRPQPKPQLPPQQP---------RHQVQQPQIKL-QQPPKLPPSAIDPSQPQP 154

Pogona_vitticeps_Acr SAARRPNSRPQPKLQLPPQQP---------THQVQQPQIKV-QQPPKLPPSAADPSQPQP 154

Bradypodion_pumilum_Acr PAAKRPNGR---------QQP---------RHQVLQQQPKV-LQPPKQPPRVSSLPQPQP 156

Bradypodion_ventrale_Acr PAAKRPNGQ---------QQL---------RHQVLQQQPKV-LQPPKQPPRVSSLPQPQP 156

Chamaeleo_calyptratus_ENAM_Acr PAAKKPNGR---------QQP---------RHQVLQQQPKV-LQPPKQPPRVSSLPQPQP 138

Chamaeleo_dilepis_ENAM_Acr PAAKRPNGR---------QQP---------RHQVLQQQPKVLLQPPKQPPRVSSLPQPQP 139

Chamaeleo_gracilis_ENAM_Acr PAAKRPNGR---------QPP---------RHQVLQQQPKV-LQPPKQPPRVSSLPQPQP 138

Chamaeleo_laevigatus_ENAM_Acr PAAKRPNGR---------QQP---------RHQVLQQQPKV-LQPPKQPPRVSSL--PQP 136

Furcifer_pardalis_Acr SAAKRPNGR---------QQP---------RHQVLQPQAKV-LQPPKQPPRVNNLPQPQP 138

Trioceros_affinis_ENAM_Acr PAAKRPTGQ---------QQP---------RHQVLHQQPKV-VQPPKQPPRFNSLPQPQP 138

Trioceros_balebicornutus_ENAM_Acr PAAKRPNGQ---------QQP---------RHQVLQQQPKV-LQPPKQPPRVSSLPQPQP 138

Trioceros_harennae_ENAM_Acr PAAKRPNGQ---------QQP---------RHQVLQQQPKV-LQPPKQPPRVSSLPQPQP 138

Anolis_apletophallus_Pleu PVSKKPNSRPQQKPRLAPQQP---------RYQ--QPQPKT-HLPAKQPPVASHPFQPQP 148

Anolis_tropidonotus_ENAM_Pleur PVSKKPNSRPQQKPRLAPQQP---------RYQ--QPQPKI-HLPSKQPPVATHPFQPQP 148

Anolis_sagrei_ordinatus_Pleu PVSKKANSRPQQKPRLAPQQP---------RYQ--QPQPKT-HLPAKQPPVATQPFQPQP 148

Anolis_carolinensis_Pleu PVPKKPNSRPRLKPRLVPQQP---------RYQ--QPQPKI-HLPAKQPPVATHPFQPQP 148

Ctenosaura_bakeri_Pleu PVSKKANSRPQPKPRLTPQQP---------RHQ--QPQPKI-QPPRRQPQSTTSPSQPHP 148

Cyclura_pinguis_Pleu PVSKKANSRPQPKPRLTPQQP---------RHQ--QPQPKI-QPPPRQPQSTTSPSQPHP 148

Iguana_delicatissima_Pleu PVSKKANSRPQPKPHLTPQQP---------RHQ--QPQPKI-QPPPRQPQSTTSPSQPHP 148

Gambelia_wislizenii_Pleu PVPKKSNSRPQPKPQLIPQQP---------RYQ--QPQPKI-HQPPKQPQITTSPSQPHP 148

Laemanctus_serratus_ENAM_Pleur PASKKPNSRPQPKPRLVPQQP---------RHQ--QPQPKI-PLPPKQPQMTINPSQPHP 148

Phrynosoma_blainvillii_Pleu PVPKKPNNRLQPKPQLTIKQLQPKIQQPQTRIP--QPQPKI-QLPPKQPQTPTDPSETHP 157

Phrynosoma_platyrhinos_Pleu PVPKNPNNRLQPKPQLTIKQLQPKIQQPQTRIP--QPKPKI-QLPPKQPQTPTNPSETHP 157

Sceloporus_chrysostictus_ENAM_Pleur PVP-----RLLPKPRLALQQPQPRIPQPQSRIP--QPQPKL-QLPPKQPQTPTYPSQPYP 153

Urosaurus_nigricaudus_Pleu PVPRKPNSWLQPKPRLTLQQPQPKIQQPQARIL--QPQPKI-QLAPKQPQIPTYPSQPHP 158

Sceloporus_occidentalis_Pleu PVPRKPNTRLPLKPQLTLQQPQPKIPQPQPRIP--QAQSKI-QLPPKQPQTPTYPFQPHP 158

Sceloporus_tristichus_Pleu PVPRKPNTRLPPKPRLTLQQPQPKIPQPQPRIP--QAQSKI-QLPPKQPQTPTYPFQPHP 158

Sceloporus_undulatus_Pleu PVPRKPNTRLPPKPRLTLQQPQPKIPQPQPRIP--QAQSKI-QLPPKQPQTPTYPFQPHP 158

... : : : : : . . *

Acanthocercus_cyanogaster_ENAM_Acr QGPALQPKGGKPQN--PQAFLPHQQQPWHFPQMFGHGGLHPQMFGPYQGRTPLGRPLGRP 204

Acanthocercus_minutus_ENAM_Acr QGPALQPKGGKPQN--PQAFPPHQQQPWHFPQMFGHGGLHPQMFGPYQGRTPLGRPLGRP 204

Xenagama_zonura_ENAM_Acr QGPALQPKGGKPQN--PQAFPPHQQQPWHFPQMFGHGGLHPQMFGPYQGRTPLGRPLGRP 204

Agama_doriae_ENAM_Acr QGPALQPKGGKPQN--PQAFLPHQQQPWHFPQMFGHGGLHPQMFGPYQGRTPLGRPLGRP 201

Laudakia_wui_Acr QGPALQPKGGKPQH--PQAFLPHQQQPWHFPQMFGHGGVHPQLFGPYQGRTPLGRPLGRP 208

Phrynocephalus_forsythii_Acr HGTAQQPKEGKSQH--PQAFLPHQQQPWHFPQIFGHGGLHPQLFGPYQGRTPLGRPLGRP 200

Phrynocephalus_guinanensis_Acr HGTAQQPKGGKSQH--PQAFLPHQQQPWHFPQIIGHGGLHPQLFGPYQGRTPLGRPLGRP 200

Phrynocephalus_putjatai_Acr HGTAQQPKGGKSQH--PQICLPHQQQPWHFPQIFGHGGLHPQLLGPYQGRTPLGRPLGRP 200

Phrynocephalus_vlangalii_Acr HGTAQQPKGGKSQH--LQAFLPHQQQPWHFPQIFGHGGFHPQLFGPYQGRTPLGRPLGRP 200

Phrynocephalus_versicolor_Acr HGPALQPKGGKSQH--PQAFLPHQQQPWHFPQIFGHGGLHPQLFGPYQGRTPLGRPLGRP 200

Intellagama_lesueurii_Acr QVPDLQSK----------AFLPHQQQPWHFPQMFGHGGFQPQMFGPYQGRTPLGRPLGRP 204

Pogona_vitticeps_Acr QLPDLQSK----------AFLPHQQQPWHFPQMFGHGGFQPQMFGPYQGRTPLGRPLGRP 204

Bradypodion_pumilum_Acr PVP---------------AFLPHQQHPWHFPQMFGNGGFHPQPFGPYQGRT----PRGRP 197

Bradypodion_ventrale_Acr PVP---------------AFLPHQQHPWHFPQMFGNGGFHPQPFGPYQGRT----PRGRP 197

Chamaeleo_calyptratus_ENAM_Acr QVP---------------AFLPHQQQPWHFPQMFGHGGFHRQPFGPYHGHT----PRGRP 179

Chamaeleo_dilepis_ENAM_Acr QVP---------------AFLPHQQQPWHFPQMFGHGGFHPQPFGPYQGRT----PRGRP 180

Chamaeleo_gracilis_ENAM_Acr QVP---------------AFLPHQQQPWHFPQMFGHGGFHPQPFGPYQGRT----PRGRP 179

Chamaeleo_laevigatus_ENAM_Acr QVP---------------AFLPHQQQPWHFPQMFGHGGFHPQPFGPYQGRT----PRGRP 177

Furcifer_pardalis_Acr QVP---------------AFLPHQQQPWHFPQMFGHGGFHPQPFGPYQGRT----PRGRP 179

Trioceros_affinis_ENAM_Acr QAP---------------AFLPHQQQPWHFSQXXXXXXXXXXXXXXXXXRT----PRGRP 179

Trioceros_balebicornutus_ENAM_Acr QAP---------------XXXXXXXXXXXXXXMFGHGGFHPQPFGPYQGRT----PRGRP 179

Trioceros_harennae_ENAM_Acr QAS---------------XXXXXXXXXXXXXXMFGHEGFHPQPFGPYQGRT----PRGRP 179

Anolis_apletophallus_Pleu QVPVQPPKGEKPQQ--PQAFPPHIQQPWQYPQIFGNGAFQPQLFNPYQGRM----PYGRP 202

Anolis_tropidonotus_ENAM_Pleur QVPVQPPKAEKPQQ--PQAFPPHIQQPWQYPQIFGNGAFQPQLFNPYQGRM----PFGRP 202

Anolis_sagrei_ordinatus_Pleu QVPVQPPKGEKPQQ--PQAFPPHTQQPWQFPQIFSHGAFQPQLFNPYQGRM----PFGRP 202

Anolis_carolinensis_Pleu QTPIQPPKGEKPQQ--PQAFPPHPQQPWQFPQIFGHGGFQPQLFNPYQGRM----PFGRP 202

Ctenosaura_bakeri_Pleu HGPVQQPKGEKPQQ--PQAFPPHQQPPWHFPQIFGHGGFQPPLFSPYQGRM----PFGQP 202

Cyclura_pinguis_Pleu HGPVQQPKGEKPQQ--PQAFPPHQQPPWHFPQIFGHGGFQPPLFSPYQGHM----PFGRP 202

Iguana_delicatissima_Pleu HGPVQQPNGEKPQP--PQAFPPHQQPPWHFPQIFGHGGFQPPLFSPYQGRM----PFGRP 202

Gambelia_wislizenii_Pleu RVPVQQPKGEKQQQ--PQAFPPHQHQPWHFPQIFGHGGFQPQLFSPYQGRM----PLGRP 202

Laemanctus_serratus_ENAM_Pleur HVPVQQPKGEKQQQ--PQAFPPHQQQPWHFPQIFGHGGFQPQLFSPYQGRM----PFGRP 202

Phrynosoma_blainvillii_Pleu HVPVQQPKEETQT----QAFSPHQQQPWNFPQIFGHGSFQPQLFSPYQGHM----PFGWP 209

Phrynosoma_platyrhinos_Pleu HVPVQQPKEEMQTQLNKQAFSP-QQQPWNFPQIFGHGGFQPQLFSPYQGHM----PFGRP 212

Sceloporus_chrysostictus_ENAM_Pleur HVPVQQPKGEKQQQ--PQGFPPHQQQPWHIPQIFGNGGFQPQLFSPYQGRM----PFGRP 207

Urosaurus_nigricaudus_Pleu HVPVQQPKGEKQQQ--TQGFPPHQQQPWHIPQIFGHGGFQPQLFSPYQGRM----PFGRP 212

Sceloporus_occidentalis_Pleu HVPVQQPKGEKQQQ--TQGFPPHQQQPWHIPQIFGSGGFQPQMFSPYQRRM----PFGRP 212

Sceloporus_tristichus_Pleu HVPVQQPKGEKQQQ--TQGFPPHQQQPWHIPQIFGHGGFQPQLFSPYQRRV----PFGRP 212

Sceloporus_undulatus_Pleu HVPVQQPKGEKQQQ--TQGFPPHQQQPWHIPQIFGHGGFQPQLFSPYQRRM----PFGRP 212

. : * * *

Acanthocercus_cyanogaster_ENAM_Acr HISNEEGAPYFGYGFHGMGPRPA-YSEEMFEQDFEEPVEKEPPKETPATSPVTNSTVPDT 263

Acanthocercus_minutus_ENAM_Acr HISNEEGAPYFGYGFHGMGPRPA-YSEEMFEQDFEEPVEKEPPKETPATSPVTNSTVPDT 263

Xenagama_zonura_ENAM_Acr HISNEEGAPYFGYGFHGMGPRPA-YSEEMFEQDFEEPVEKEPPKETPATSPVTNSTVPDT 263

Agama_doriae_ENAM_Acr HISNEEGAPYFGYGFHGMGPRAA-YSEEMFEQDFEEPVEKEPPKETPATSPVTNSTVPDT 260

Laudakia_wui_Acr HVSNEEGAPYFGYGFHGMGPRPA-YSEEMFEQDFEEPVEKEPPKETPATSPVTNSTAADT 267

Phrynocephalus_forsythii_Acr HVSNEEGAPYFGYGFHGMGTRPA-YSEEMFEQDFEEPVEKEPPKETPASSPVTNSTVPDT 259

Phrynocephalus_guinanensis_Acr HVSNEEGAPYFGYGFHGMGTRPA-YSEEMFEQDFEEPVEKEPPKETPASSPVTNSTVPDT 259

Phrynocephalus_putjatai_Acr HVSNEEGAPYFGYGFHGMGTRPA-YSEEMFEQDFEEPVEKEPPKETPASSPVTNSTVPDT 259

Phrynocephalus_vlangalii_Acr HVSNEEGAPYFGYGFHGMGTRPA-YSEEMFEQDFEEPVEKEPPKETPASSPVTNSTVPDT 259

Phrynocephalus_versicolor_Acr HVSNEEGAPYFGYGFHGMGTRPA-YSEEMFEQDFEEPVEKEPPKETPASSPVTNSTVPDT 259

Intellagama_lesueurii_Acr HVSNEEGAPYFGYGYHGMGHRPP-YSEEMFEQDFEEPVEKEPAKETPATNPVTNSTVSDT 263

Pogona_vitticeps_Acr HVSNEEGAPYFGYGYHGMGHRPP-YSEEMFEQDFEEPVEKEPAKETPATNPVTNSTVADT 263

Bradypodion_pumilum_Acr HVSNEEGMPYFGYGYHGMGGRAP-YSEEMFEQDFEEPVEKEPPKETPV----TNSTVPDT 252

Bradypodion_ventrale_Acr HVSNEEGMPYFGYGYHGMGGRAP-YSEEMFEQDFEEPVEKEPPKETPV----TNSTVPDT 252

Chamaeleo_calyptratus_ENAM_Acr HVSNEEGIPYFGYGYHGMGGRAP-YSEEMFEQXFEEPVEKEPXKETPA----TNSTVPDT 234

Chamaeleo_dilepis_ENAM_Acr HVSNEEGMPYFGYGYQGMGGRAP-YSEEMFEQDFEEPVEKEPPKETPA----TNSTVPDT 235

Chamaeleo_gracilis_ENAM_Acr HMSNEEGMPYFGYGYQGMGGRAP-YSEEMFEQDFEEPVEKEPPKETPA----TNSTVPDT 234

Chamaeleo_laevigatus_ENAM_Acr HVSNEEGMPYFGYGYQGMGGRVP-YSEEMFEQDFEEPVEKEPPKETPS----TNSTVPDT 232

Furcifer_pardalis_Acr HVSNEEGMPYFGYGYHGIGGRAP-YSEEMFEQDFEEPVEKEPPKETPT----NNSTVPDT 234

Trioceros_affinis_ENAM_Acr HVSNEEGMPYFGYGYHGMGGRAP-YSEEMFEQDFEEPVEKEPPKETPA----TNSTVPDT 234

Trioceros_balebicornutus_ENAM_Acr HVSNEEGMPYFGYGYHGMGGRAP-YSEEMFEQDFEEPVEKETPKETPA----TNSTVPDT 234

Trioceros_harennae_ENAM_Acr HVSNEEGMPYFGYGYHGMGGRAP-YSEEMFEQDFEEPVEKEPPKETPA----TNSTVPDT 234

Anolis_apletophallus_Pleu P-NSEEGTPYFGPGYQGMGGRPPYYSEEMFEQE-----DKEPTKETPATDPVSNST--DT 254

Anolis_tropidonotus_ENAM_Pleur P-NSEEGTPYFGPGYQGMGGRPPYYSEEMFEQE-----DKEPTKESPATDPVSNST--DT 254

Anolis_sagrei_ordinatus_Pleu P-NSEEGTPYFGPGYQGMGGRPPYYSEEMFEQE-----DKEPTKESPATDPVTNST--DT 254

Anolis_carolinensis_Pleu P-NSEEGNPYFGPGYQGMGGRPPYYSEEMFEQE-----DKEAPKESPATDPVTNST--DT 254

Ctenosaura_bakeri_Pleu PVSNEEGTPYFGYGYQGMGGRPPYYSEEMFEQDFEKPKEKEAPKESPSTDPVTNTTASDT 262

Cyclura_pinguis_Pleu PVSNEEGTPYFGYGYQGMGGRPPYYSEEMFEQDFEKPKEKEAPKESPSTDPVTNTTASDT 262

Iguana_delicatissima_Pleu PVSNEEGTPYFGYGYQGMGGRPPYYSEEMFEQDFEKPKEKEAPKESPSTDPVTNTTASDT 262

Gambelia_wislizenii_Pleu PISNEEGTPYFGYGYQGMGGRPPYYSEEMFEQDFEKPKEEEAPKESPATDPITNTTVSDT 262

Laemanctus_serratus_ENAM_Pleur PISNEEGTPYFGYGYQGMGGRPPYYSEEMFEQDFEKPKEKEAPKESPATDPVTNSTASDT 262

Phrynosoma_blainvillii_Pleu PTSKEEGTPYFGYGYQGMGGRPPYYSEEMFEQDFEEPKEKETTKESPATDPVTNSTVSDT 269

Phrynosoma_platyrhinos_Pleu TTSKEEGTPYFGYGYQGMGGRPPYYSEEMFEQDFEEPKEKETTKESPATDPVTNSTVSDT 272

Sceloporus_chrysostictus_ENAM_Pleur PISNEEGTPYFGYGYQGMGGRPPYYSEEMFEQDIEEPIEKEAPKESPATDPVTNSTVSDT 267

Urosaurus_nigricaudus_Pleu PISNEEGTPYFGYGYQGMGGRPPYYSEEMFEQDIEEPVEKEAPKESPATDPVTNSTVSDT 272

Sceloporus_occidentalis_Pleu PISNEEGTPYFGYGYQGMGGRPPYYSEEMFEQDIEEPVEKEAPKESPATDPVTNSTVSDT 272

Sceloporus_tristichus_Pleu PVSNEEGTPYFGYGYQGMGGRPPYYSEEMFEQDIEEPVEKEAPKESPATDPVTNSTVSDT 272

Sceloporus_undulatus_Pleu PVSNEEGTPYFGYGYQGMGGRPPYYSEEMFEQDIEEPVEKEAPKESPATDPVTNSTVSDT 272

..*** **** *::*:* * . ******** ::*. **:* .*:* **

Acanthocercus_cyanogaster_ENAM_Acr NTTVSNPASQGGNATISGASATGNGVNPLGLQSKYVEGNGFSTPSPT-AHVSNGNEVAQD 322

Acanthocercus_minutus_ENAM_Acr NTTVSNPASQGGNATISGASATGNGANPLGLQSKFVDGNGFSTPSPT-AHVSSGNGVAQD 322

Xenagama_zonura_ENAM_Acr NTTVSNPASQGGNATISGASATGNGANPLGLQSKFVDGNGFSTPSPT-AHVSSGNGVAQD 322

Agama_doriae_ENAM_Acr NATVPNPANQGGNATISGASATGNGANPLGLQIKHADGNGFSTPSPT-AHVLSGNGAALD 319

Laudakia_wui_Acr NATVSNPASQGGNATISGASTTGNGANPLGLQSKFVEGNGFSTPSPT-AHVSGGNGAAQD 326

Phrynocephalus_forsythii_Acr NTTVPNPASQGENATLSGASVTGNGANPMGLQSKFIDGNGFSTPSPP-AHVAGGNGAAQD 318

Phrynocephalus_guinanensis_Acr NTTVPNPASQGENATLSGASATGNGANPMGLQSKFVGGNGFSTPSPP-AHVSGGNGAAQD 318

Phrynocephalus_putjatai_Acr NTTVPNPASQGENATLSGASATGNGANPMGLQSKFVGGNGFSTPSPP-AHVSGGNGAAQD 318

Phrynocephalus_vlangalii_Acr NTTVPNPASQGENATLSGASATGNGANPMGLQSKFVDGNGFSTPSPP-AHVSGGNGAAQV 318

Phrynocephalus_versicolor_Acr NATVPNPASQGENATLSGTSATGNGANPLGLQGKFVDGNGFSTPSPP-AHVSGGNGAAQD 318

Intellagama_lesueurii_Acr NTTVSNPASQGGNATISEPIATGNGANPLGLQSKLVDGNGFSTPSPT-AHVSGGNGAAQD 322

Pogona_vitticeps_Acr NTTVSNPASQGGNATISEPIATGNGANPLGLQSKLVDGNGFSTPSPT-AHVSGGNGAAQD 322

Bradypodion_pumilum_Acr NTTVSNPANQGGNATISGPSVTGTAVNSLGLQSKLVDGNEISTPSP--THVSGGNRAAQE 310

Bradypodion_ventrale_Acr NTTVSNPANQGGNATISGTSVTGTAANSLGLQSKLVDGNEISTPSP--THVSGGNRAAQE 310

Chamaeleo_calyptratus_ENAM_Acr NTTVSNSANQGGNATISGPSLTGNGANSLGLQSKLVDGNGLSTPSPT-THVSGGNGAPQE 293

Chamaeleo_dilepis_ENAM_Acr NTTVSNPANQGGNATISGPSVTGNGANSLGLQSKSIDGNGLSTPSPT-THVSGGNGAAQE 294

Chamaeleo_gracilis_ENAM_Acr NTTVSNPANQGGNATISGPSVTGNGANSLGLQSKSIVGNGLSTPSPT-THVSDGNVAAQE 293

Chamaeleo_laevigatus_ENAM_Acr NTTVSNPANQGGNATISGSSVTGNGANSLGLQSKLIDGNVLSTPSPT-THVSGGNGAAQE 291

Furcifer_pardalis_Acr NTTVSNPANQGGNATISGPSVTGNGANSLGLQSKLVDGNGLSTPSPT-THVPGGNGAAPE 293

Trioceros_affinis_ENAM_Acr NTTASNPANQGGNVTISGPSVTGNGANSLGLQSKLVDGNVLSTPSPT-THLSGGNGAAQE 293

Trioceros_balebicornutus_ENAM_Acr NTTVSNPANQGRNVTISGPSVTGNGANSLGLQSKLVDGNVLSTPSPT-THLSGGNGAAQE 293

Trioceros_harennae_ENAM_Acr NTTVSNPANQGGNVTISGPSVTGNGANSLGLQSKLVDGNVLSTPSPT-THLSGGNGAAQE 293

Anolis_apletophallus_Pleu NSTISNPAGQAGNGTISGLGEKATGANSPSLQNNLVSGNAASTPLPT-TKASEGNGVAPN 313

Anolis_tropidonotus_ENAM_Pleur NSTISNPASQAGNGTISGLGEKATGANSPSLQNNLVSGNAASTPLPT-TKVSEGNGVAPN 313

Anolis_sagrei_ordinatus_Pleu NSTISNPASQAGNGTISGLGEKAIGANSPGLQNNLVSGNAASTPLPT-TKASEGNGVAPN 313

Anolis_carolinensis_Pleu NSTVSNPASQGGNGTMSGLSEKATGANSPGLQNNMFSGNAVSTPLPT-TQTSEGNGVAQN 313

Ctenosaura_bakeri_Pleu NSTSSNPASQAGNGTISSPSEKATGSSSPGLQNKLVSGNEVSTALPT-TQMSEGNGVAQN 321

Cyclura_pinguis_Pleu NSTSSNPASQAGNGTISSPSEKATGPSSPGLQNKLVSGNEVSTALPT-TQMSEGNGVAQN 321

Iguana_delicatissima_Pleu NSTSSNPASQGGNGTISSPGEKATGPSSQGLQNKPVSGNEISTALPT-TQMSEGNGVARN 321

Gambelia_wislizenii_Pleu NSTVSNPISQGGNGTISRLGEKATGANSLGIQNKLVSGNGVPTSLPT-TEMSEGNEVAQN 321

Laemanctus_serratus_ENAM_Pleur NSTISNPASQGGNGTISGLAEKATGPSSPGLQNKLVSGNGVPTSLPT-TQISEGNGVPQN 321

Phrynosoma_blainvillii_Pleu NSTISNPASQGGNDTVSGLGEKVTRASSPGLQNKLVSGNGASTPSPTSTQMSEGNGVAQN 329

Phrynosoma_platyrhinos_Pleu NSTISNPASQGGNDTVSGLGVKVTRASSPGLQNILVSGNGASTPSPTSTQMSEGNGVAQN 332

Sceloporus_chrysostictus_ENAM_Pleur NSTISNPSSQVGNATISGLGGKVTGAISPSLQNNLVSGNRASTPLPTSTHMLEGNGVAQN 327

Urosaurus_nigricaudus_Pleu NSTISNPVSQVGNDTISGLGGKVTGASSPSLQNKLVSGNRASTPFPTSTHMLEGNGVAQN 332

Sceloporus_occidentalis_Pleu NSTISNPAGQVGNDTISGLGGKVTGASSPSLQNKLVSGNRASTPLPTSTHMLEGNGVAQS 332

Sceloporus_tristichus_Pleu NSTISNPAGQVGNDTISGLGGKVTGASSPSLQNKLVSGNRASTPLPTSTHMLEGNGVAQN 332

Sceloporus_undulatus_Pleu NSTISNPAGQVGNDTISGLGGKVTGSSSPSLQNKLVSGNRASTPLPTSTHMLEGNGVAQN 332

*:* .*. .* * *:* . . .:* ** .*. * :. ** ..

Acanthocercus_cyanogaster_ENAM_Acr VLEQPDHQSKSPSVNIMQGFPPASQHSTGHVLHMYKPHSEVGDARHNSLTSRGN-PFIQS 381

Acanthocercus_minutus_ENAM_Acr VLEQPDHQSKSPSVNIMQRFPPASQHSTGHVPHMYKPHSEMGDARHNSLTSRGN-PYIQS 381

Xenagama_zonura_ENAM_Acr VLEQPDHQSKSPSVNIMQRFPPASQHSTGHIPHMYKPHSEMGDARHNSLTSRGN-PYIQS 381

Agama_doriae_ENAM_Acr ALEQPGHQSKSPSVNIMQSFPPANQHSTGHIPHMYKPHSEMGDARHSSLTSRGN-PYMQS 378

Laudakia_wui_Acr ALEQYGHQPKSPNVNIMQSFPPASQHSTGHVPHIYKPHSEMGDARHNSLTSRGN-PSIQT 385

Phrynocephalus_forsythii_Acr VLEQSGHQDKSPSLNIMQSFPPASQHSAGHVPPIYKPNSEVGDARHHSLTSRGN-P-IQT 376

Phrynocephalus_guinanensis_Acr VLEQSGHQDKSPSLNIMQSFPPASQHSAGHIPPIYKPNSEVGDARHHSLTSRGN-P-IQT 376

Phrynocephalus_putjatai_Acr VLEQSGHQDKSPSLNIMQSFPPASQHSAGHIPPIYKPNSEVGDARHHSLTSRGN-P-IQT 376

Phrynocephalus_vlangalii_Acr VLEQSGHQDKSPSLNIMQSFPPASQHSAGHIPPIYKPNSEVGDARHHSLTSRGN-PSIQT 377

Phrynocephalus_versicolor_Acr VLEQSGHQPKSPSVNIMQSFPPASQHSTGHVPPIYKPNSEMGDARHHSLTSRGN-PSIQT 377

Intellagama_lesueurii_Acr ALEQSSHQPKSP--NMIQSFPPASQQSTGHVPHIYNPHSEMGDARHNTLTFRGN-PSIQA 379

Pogona_vitticeps_Acr ALEQSGHQPKSP--NMIQSFPPASQQSTGHVPHIYNPHSEMGDARHNTLTFRGN-PSIQA 379

Bradypodion_pumilum_Acr ALEPSGHQPKSPNINIIQSFPPASQLSAGHVAHIYNSRSEMGDARYNTLTSRGN-PPIQN 369

Bradypodion_ventrale_Acr ALEPSGHQPKSPNINIIQSFPPASQLSAGHVAHIYNSRSEMGDARYNTLTSRGN-PPIKN 369

Chamaeleo_calyptratus_ENAM_Acr ALEPSGHQPKSPNVNIIQTFPPSSQLSTGHVAHLYNPHSEMGDARYNTLTSRGNPPPIQT 353

Chamaeleo_dilepis_ENAM_Acr ALEPSGHQPKSPNVNIIQTFPPASQLSTGHVAHLYNPPSEMGDARYNTLTSRGNPPPIQT 354

Chamaeleo_gracilis_ENAM_Acr ALEPSGHQPKSPNVNIIQTFPPASQLSTGHVAHLYNPPSEMGDARYNTLTSRGNPPPIQT 353

Chamaeleo_laevigatus_ENAM_Acr ALEPSGNQPKSPNVNIIQTFPPASQSSTGQVAHLYNPRSEMGDARYNTLTSRGNPPPIQT 351

Furcifer_pardalis_Acr ALEPSGHQPNSPNVNIIQSFPPASQLSTGHVAHIYNPRSEMGDARYNTLTSRGN-PHVQT 352

Trioceros_affinis_ENAM_Acr ALEPSGHQPQSQNVNIIQSFPPASQLSTGHVAHIYNPHSEMRDARYNTLTSRGN-PPIQT 352

Trioceros_balebicornutus_ENAM_Acr ALEPSGHQPKSPNVNIIQSFPPASQLSTGHVAHIYNPRSEMRDARYNTLTSRGN-PPIQT 352

Trioceros_harennae_ENAM_Acr ALEPSGHQPKSPNVNIIQSFPPASQLSTGHVAHIYNPRSEMRDARYNTLTSRGN-PPIQT 352

Anolis_apletophallus_Pleu VLEQLRHRSKVPNGDVIQSFPSGRQHATIQVSNIYRPHERMENIKQN-LISRGN-PSIQT 371

Anolis_tropidonotus_ENAM_Pleur VIEQLRHRPKVPNGDVIQSFPSGRQHSTMHVSNIYRPHEHMENIKQN-LISRAN-PSIQT 371

Anolis_sagrei_ordinatus_Pleu VLEQLRHRSKVPNGDVIQSFPSGRQHSTMQISNIYRPHEHMENTKQN-LISRGN-PSIQT 371

Anolis_carolinensis_Pleu VLEQIRHRSKLPNGDVIQSFPSGRQHSTMQVYNIHKPHEHMENIKQN-LISRGN-PSIQS 371

Ctenosaura_bakeri_Pleu VLEQSSHRSKFPNDHVIQSFPSGSQHSTMQVSNIYKPSEHTGDTRQNSLISRGN-PSIQA 380

Cyclura_pinguis_Pleu VLEQSSHRSKFPNDHVIQSFPSGSQHSTMQVSNIYKPSEHMGDTRQNSLISRGN-PSIQA 380

Iguana_delicatissima_Pleu VLEQSSHRPKFPNDHVIQSFPSGSQHSTMQVSNFYKPSEHMGDTRQNSLISRGN-PSIQA 380

Gambelia_wislizenii_Pleu VLEQSSHRSKSLNDQVIQSFPSGSQHSTMQVSNIYKPHEHMGDTRQNTLISRGD-PSVPV 380

Laemanctus_serratus_ENAM_Pleur VLEQSSQRSNSPNGRVIQSFPSGSQPSTMQLSNIYKPHEHMGDTRQNTLVSRGN-PSIQA 380

Phrynosoma_blainvillii_Pleu VLEQSSHRSRSPNGHIIQHFPSDSQHSTMQVSNIYKPHEHMGDTRHNIPISRGN-PSIHA 388

Phrynosoma_platyrhinos_Pleu VLEQSSHRSRSPNGHIIQHFPSGSQHSTMQVSNIYKPHEHMGDTRHNIPISRGN-PSIHA 391

Sceloporus_chrysostictus_ENAM_Pleur ILEQSSSRSRSPNGHVIQSFPSSSQHSTMQVSNIYKPHEHMGDTKHDTLISRGN-PSIHA 386

Urosaurus_nigricaudus_Pleu VLEQSSNRFRSSNGHVIQSFPSSSQHSTMQVSHIYKPHEHIGDTKHNTLVSRGN-PSIHA 391

Sceloporus_occidentalis_Pleu ILEQSSHRPRSPNGHVIQSFPSNNQHSTMQVSNIYKPLEPMGDTKHNTLTSRGN-PSIHA 391

Sceloporus_tristichus_Pleu VLEQSSHRSRSPNGHVIQSFPSSSQHSTMQVSNIYKPLEPMGETKHNTLISREN-PSIHA 391

Sceloporus_undulatus_Pleu ILEQSSHRSRSPNGHVIQSFPSSSQHSTMQVSNIYKPLEPMGDTKHNTLISRGN-PSIHA 391

:* : . ::* **. * :: :: ::.. . : : * : * :

Acanthocercus_cyanogaster_ENAM_Acr ESPSYNFGYRENLDQRGTLQNTNNNPALANPRHLSYGHQEQSHLPGRNPSDQREIVPFSA 441

Acanthocercus_minutus_ENAM_Acr ESPSYNFGYRENLDQRGNLQNTNNNQALANPRHLSYGQQEHSHLPGRNPSDQREIVPFSA 441

Xenagama_zonura_ENAM_Acr ESPSYNFGYRENLDQRGNLQNTNNNQALANPRHLSYGQQEHSHLPGRNPSDQREIVPFSA 441

Agama_doriae_ENAM_Acr ERPSYNFDNRENLDRRGNLQNTNNNQALGNPRHLSYGPQEQPHLPGRNPSNQREIIPFPA 438

Laudakia_wui_Acr ESPPQNFGYRENLDRRGHLQNTNNNQALANPRRLSYGHQEQSHLPGRNPSGQREIMPFPA 445

Phrynocephalus_forsythii_Acr ESPSHNFGYRDNLDQRGNLHNTNNNQALANPRHLSYGYQEQSHLPGRSPSGQREIRPFPD 436

Phrynocephalus_guinanensis_Acr ESPSHNFGYRDNLDQRGNLHNTNNNQALANPRHLSYGYQEQSHLPGRSPSGQREIRPFPD 436

Phrynocephalus_putjatai_Acr ESPSHNFGYRDNLDQRGNLHNTNNNQALANPRHLSYGYQEQSHLPGRSPSGQREIRPFPD 436

Phrynocephalus_vlangalii_Acr ESPSHNFGYRDNLDQRGNLHNTNNNQALANPRHLSYGYQEQSHLPGRSPSGQREIRPFPD 437

Phrynocephalus_versicolor_Acr ESPSHNFGYRENLDQRGNLHNTNNNQALANPRHLSYGHQEQPHLPGRSPSGQREIRPFPD 437

Intellagama_lesueurii_Acr ENPSRSFGYRDNLDPRGNLQSTNSNQAMANPRPISYGHQEQSHLPGRNPSGQREIMPFPT 439

Pogona_vitticeps_Acr ENPSRSFGYRDNLDPRGNLQSTNSNQAMANPRPISYGPQEQSHLPGRSPSGQREIMPIPT 439

Bradypodion_pumilum_Acr ENKLHSFDYRENADPRDNFQSTNNNQALENPRPVSYRHQEQSHFIGRSPSGQGERKHFPT 429

Bradypodion_ventrale_Acr ENKLHSFDYRENSDPRDNFQSTNNNQALENPRPVSYRHQEQSHFIGRSPSGQGERKHFPT 429

Chamaeleo_calyptratus_ENAM_Acr ENQLHSFDYRENSDPRDNFQSTNNNQALENPRPISYRHQEQSHFLGRSPSGQGERKHFPT 413

Chamaeleo_dilepis_ENAM_Acr ENQFHSFDYRENSDPRDNFQSTNNNQALENPRPISYRHQEQSHFLGRNPSGQGERKHFPT 414

Chamaeleo_gracilis_ENAM_Acr ENQFHSFDYKENSDPRDNFQSTNINQALENPRPISYRHQEQSHFLGRNPSGQGERKHFPT 413

Chamaeleo_laevigatus_ENAM_Acr ENQLHSFDYRE----RDNFQSTNNNQALENPRPISYRHQEQSHFLGRSPSGQGERKHFPT 407

Furcifer_pardalis_Acr ENRLHSFDYRENSDPRDNFQSTNNNQALENPKPISYRHQEQSHFLGRSPSGQGERNHFPT 412

Trioceros_affinis_ENAM_Acr ENQLYSFDYRENSDPRDHFQRTNDNQALENPRAISYRHQEQSHFLGRSPSGQGERKHFPT 412

Trioceros_balebicornutus_ENAM_Acr ENQLYSFDYREISDPRDNFQRTNNNQALENPRPISYRHQEQSHFLGRSPSGQGERKHFPT 412

Trioceros_harennae_ENAM_Acr ENQLYSFDYREISDPRDNFQRTNNNQALENPRPISYRHQEQSHFLGRSPSGQGERKHFPT 412

Anolis_apletophallus_Pleu EDPIYPMGNDWNPNHRGNIQNKNVNRPSTNSRHIQYGQQEQPPYPERNPFSQIDRVPFPS 431

Anolis_tropidonotus_ENAM_Pleur EDPTYPMGNDWNPNHRGNIQNTNVNHPSPNTRHIQYGQQEQPHYPERNTFSQIDRVPFPS 431

Anolis_sagrei_ordinatus_Pleu EDPTYPMGNDWNPNHRSNIQNTNVNHPSTNTRHIPYGQQEQPHYPERNPYSQIDRVPFPS 431

Anolis_carolinensis_Pleu EDPTYPMGNGWNSNHRGNIQNTNVNNPSTNTRHIPYGQQEQPHYPERNEFSQIDRVPFPS 431

Ctenosaura_bakeri_Pleu EDPTYPLAYGRNPNHRDHLQSTSINHPSTNTRHIPHGQQDQLPYPGRNPLDRRERVPFPH 440

Cyclura_pinguis_Pleu EDPTYSLVYGGNPNHRDHLQSTSINHPSTNTRHIPHGQQDQLPYPGRNPLGRRERVPFPH 440

Iguana_delicatissima_Pleu EDPTYPMAYGRNPNHRDHLQSTSINHPSTNTRHIPHRQQDQLPYPGRNPLGRRERVPFPH 440

Gambelia_wislizenii_Pleu EDPTYSLGYGRNPNYRGNLQSTNSNHPSTNTRHAPNGQQEQPHHPGRNPFGQRERVPFHS 440

Laemanctus_serratus_ENAM_Pleur EDPEYPLGYGRNPNHRDNLQSTNSNHHSTNTRHVPYEPQEQPHYPGRNLFDQRERVPFPS 440

Phrynosoma_blainvillii_Pleu EDPTYPLNYGRNPNHRGNLQNTDSNYPSANTRRIPYGQQEQPHYPGRNPLGQRERMSFPS 448

Phrynosoma_platyrhinos_Pleu EDPTYPLNYGRNPNHRGNLQNTNSNYPSANTRLIPYGQQEQPHYLGRNPLGQRERMSFPS 451

Sceloporus_chrysostictus_ENAM_Pleur EDPTYLLSYGRNPNHRGNLQNTDSNHPSANTRHIPYGQQEQPDYPGRNPLSQRERVSFPS 446

Urosaurus_nigricaudus_Pleu EDPTYLLSYGRNPNHRGNLQNTDSNHPSANTRQIPYGQQEQPHSSGRNQLSQRERVSFPS 451

Sceloporus_occidentalis_Pleu EDSAYLLNYGRNPNHRDNLPNTDSNHPSANTRNIPYGQQEQPHYHERNPLSQRERVSFPS 451

Sceloporus_tristichus_Pleu EDPAYLLNYGRNPNQRDNLQNTDSNHPSANTRNIPYGPQEQRHYHERNPLSQRERVSFPS 451

Sceloporus_undulatus_Pleu EDPAYLLNYGRNPNRRDNLPNINSNHPSANTRNIPYGQQEQPHYHERNSLSQRERVSFPS 451

* : *. : . * *.: *:: *. .: : :

Acanthocercus_cyanogaster_ENAM_Acr SDPPSTWNQDPVYRDNYQHNSPLGGHSLDPQINTLG---------------APSGMQQSN 486

Acanthocercus_minutus_ENAM_Acr SDPPSTWNQDPVYRDNYQHNSPLGSHSLDPQINTLG---------------APSGMQQSN 486

Xenagama_zonura_ENAM_Acr SDPPSTWNQDPVYRDNYQHNSPLGGHSLDPQINTLG---------------APSGMQQSN 486

Agama_doriae_ENAM_Acr SDPPRTWNQDPVYRDNYQHNSPLEGHSLDPQINTLG---------------SPSGMQQSN 483

Laudakia_wui_Acr SDPPSTWNQDPVYRDNYLHNSPSEGHSLNPQINTLG---------------FPSGMQQSN 490

Phrynocephalus_forsythii_Acr SDTPSTWNQDPVYRDNYQRNSPSEGHSLDPQINTLQ---------------SPSGMQQSN 481

Phrynocephalus_guinanensis_Acr SDTPSTWNQDPVYRDNYQHNSPSEGHSLDPQINTLQ---------------SPSGIQQSN 481

Phrynocephalus_putjatai_Acr SDTPSTWNQDPVYRDNYQRNSPSEGHSLDPQINTLQ---------------SPSGIQQSN 481

Phrynocephalus_vlangalii_Acr SDTPSTWNQDPVYRDNYQRNSPSEGHSLDPQINTLQ---------------SPSGMQQSN 482

Phrynocephalus_versicolor_Acr SDPPSTWNQDPVYRDNYQRNSPSEGHSLDPQINTLQ---------------SPSGMQQSN 482

Intellagama_lesueurii_Acr SDPPSRWNKDPVYRD-YLHNSPSEGHSLDPEINTLR---------------QPSGMQQSN 483

Pogona_vitticeps_Acr SDPPSRWNKDPVYRDNYLHNSPPEGHSVDPQINTLR---------------RPSGMQQPN 484

Bradypodion_pumilum_Acr SDPLSEWNKDPVYGENYQNNSPPEDTSLDQPIDRLG---------------QHSGMQQSN 474

Bradypodion_ventrale_Acr SDPLSEWNKDPVYGENYQNNSPPEGTSLDLPVDRLG---------------QHSGMQQSN 474

Chamaeleo_calyptratus_ENAM_Acr SKMLSEWNKDPVYRENYQNNSPPEGTSLDLPIDRLG---------------QRSGMQKSN 458

Chamaeleo_dilepis_ENAM_Acr SEMLREWNKDPVYRENYQNNSPPEGTSLDLPIDRLG---------------QHSGMQKSN 459

Chamaeleo_gracilis_ENAM_Acr SEMLREWNKDPVYRENYQNNSPPEGTSLDLPIDRLG---------------QHSGMQKSN 458

Chamaeleo_laevigatus_ENAM_Acr SEMLSEWNKDPVYRENYQNNSPPEGTSLDLPIDRLG---------------QHSGMQKSN 452

Furcifer_pardalis_Acr SDPLSEWNKDPVYRENYQNNSPPEGTSPDLPIDRLG---------------QHSGMPQSN 457

Trioceros_affinis_ENAM_Acr SDPLSEWNKDPVYKENYQNNSPPEGTSLDLPIDRLG---------------QHSGMQQSN 457

Trioceros_balebicornutus_ENAM_Acr SDPLSEWNKDPVYRENYQNTSPPEGTSLDLPIDRLG---------------QHSGMQQSN 457

Trioceros_harennae_ENAM_Acr SDPLSEWNKDPVYRENYQNTSPPEGTSLDLPIDRLG---------------QHSGMQQSN 457

Anolis_apletophallus_Pleu SDPVGQWNKDPIYRDNDPKNSPPEGHLLDPQFKTMR-IDNIYNERQDAERFQPSGMHQSN 490

Anolis_tropidonotus_ENAM_Pleur SDPVGQWNKDPIYRDNDPKNSPPEGHSLDPQFKTMR-IDNIYNERQDAERFQPSGMHQSN 490

Anolis_sagrei_ordinatus_Pleu SDPVGQWNKDPIYRDNDPKNSPPEGHPLDPQFKTMR-IDNIYNERQDSERFQPSGMHQSN 490

Anolis_carolinensis_Pleu SDPVGQWNKDPIYRDNDPKNSPPEGHPLDPQFKTMR-IDNIYNARQDAERFQPSGMHQSN 490

Ctenosaura_bakeri_Pleu SEPMGQWNKDPVYRHNGPENSPPEGHFLDPQFQTLKHTDNIYNAREDIERLQPSGMQQSN 500

Cyclura_pinguis_Pleu SEPVGQWNKDPVYRHNSPKNSPPEGHFLDPQFKKLKHTDNIYNAREDIERLQPSGMQQSN 500

Iguana_delicatissima_Pleu SEPVGQWNKDPVYRHNGPKNSPPEGHFLDPQFKTLKHTDNIYNAREDIERLQPSGMQQSN 500

Gambelia_wislizenii_Pleu SDPVGQWNKDAVYRDNGPKISPPEGHSLDPQFKTLRQTDNAYNARENIERLQPSGMHQSN 500

Laemanctus_serratus_ENAM_Pleur SDPVGQWHKDPVYRDNGPKNSPPEGHSLDPQFKTLGQTDNSYNAREDIERIQPSGMYQSK 500

Phrynosoma_blainvillii_Pleu SDPVHQWNKEPVYRDNGPKNSPPESHSLDPQFKTLRKTDNAYNEREDIESFQPSGMHKSN 508

Phrynosoma_platyrhinos_Pleu SDPVHQWNKEPVYRDNGPKNSPPESHSLDPQFKTLRKTDNAYNEREDIESFQPSGMHKSN 511

Sceloporus_chrysostictus_ENAM_Pleur SDTVHQWNQEPVYSDNGPKNSPPKSHSLDSQFKTLRQTDNTYNAREDIESFQPSGMHKSN 506

Urosaurus_nigricaudus_Pleu SDTVHQWNKESVYRDNGPINSPPKSHFLDPQFKTLRQTDNTYNAREDVESFQPSGMHKSH 511

Sceloporus_occidentalis_Pleu SDTVHQWSKEPVYRHNGPNNSPPKSHSLDPQFKTLRESDNTYNAREDIESFQPSGMHKSN 511

Sceloporus_tristichus_Pleu SDTVHQWSKEPVYRHNGPDNSPPKSHSLDPQFKTLRESDNIYNAREDIESFQPSGMHKSN 511

Sceloporus_undulatus_Pleu SDTVHQWSKEPVYRDNGPKNFPPKSHSLDPQFKTLRESDNTYNAREDIESFQPPGMHKSN 511

*. * ::.:* . * . : .. : .*: :.:

Acanthocercus_cyanogaster_ENAM_Acr APSQQGIFSATRRTPFETETHQNDWKPQLPNLPDREREQFPSAQNEMWSNRENSWTFQD- 545

Acanthocercus_minutus_ENAM_Acr APSQQGMFSATRRTPFETETHQNDWKPKLPNLPDRERE-----QNEMWSNRENSWTFQD- 540

Xenagama_zonura_ENAM_Acr APSQQGMFSATRRTPFETETHQNDWKPKLPNLPDRERE-----QNEMWSNRENSWTFQD- 540

Agama_doriae_ENAM_Acr IPSQQGMFSATRRTPFEAETHQNDWKPPLPNLPDREREQFPSTQNQMWSNRENSWTFQD- 542

Laudakia_wui_Acr TPSQQGMFSATRRTPFETETHQYDWKPQFPDIPDREREQIPSAQNQMWSNRENSWTFQD- 549

Phrynocephalus_forsythii_Acr APSQQGMFSATRRTPFESETHQNNWKPQLPNLPDHEREQFPSAQNRMWSNRENSWTFQD- 540

Phrynocephalus_guinanensis_Acr APSQQGMFSGTRRTPFETETHQNNWKPQLPSLPDREREQFPSAQNRMWSNRENSWTFQD- 540

Phrynocephalus_putjatai_Acr APSQQGMFSGTRRTPFETETHQNNWKPQLPSLPDREREQFPSAQNRMWSNRENSWTFQD- 540

Phrynocephalus_vlangalii_Acr APSQQGMFSATRRTPFETETHQNNWKPQLPNLPDREREQFPSAHNRMWSNRENSWTFQD- 541

Phrynocephalus_versicolor_Acr APSQQGMFSATRRTPFETETHQNNWKPQLPNLPDREREQFPSAQNRMWSNRENSWTFQD- 541

Intellagama_lesueurii_Acr TLSERGIFSATRRTPFETETHWYDWKQQLPNPPDRERERFPSAQNQMWSSRENSQIFQE- 542

Pogona_vitticeps_Acr ILSEQEIFPSTKRTPFETETHWYDWKQQLPNPPDRERERFPSAQNQMWSSRENSQIFQD- 543

Bradypodion_pumilum_Acr AHSQQEIFSATRRTPFETEIHHYDWKQQMSSRPDHEREQFPPAQNQMRNNRENSWVFQDK 534

Bradypodion_ventrale_Acr AHSQQEIFSATRRTPFETEIHQYDWKQHMSSRPDHEREQFPPAQNQMWNNRENSWVFQDK 534

Chamaeleo_calyptratus_ENAM_Acr AHSQQEIFLSTRRTPLETEIYQYDWKQQMSSHPDHEREQFPPAQNQMWNNRENSRVFQDT 518

Chamaeleo_dilepis_ENAM_Acr AHSQQEIFLATRRTPFEAEINQYDWKQQMSSRPDHEREQFPPAQNQVWNNRENSRVFQDT 519

Chamaeleo_gracilis_ENAM_Acr AHSQQEIFLATRRTPFETEIYQYDWKQQMSSRPDHEREQFPPAQNQMWNNRENSQVFQDT 518

Chamaeleo_laevigatus_ENAM_Acr AHSQQEIFLATRRTPFETEVYQYDWKQQMSSRPDHEREQFPPAQNQMWNNRENSRVFQDT 512

Furcifer_pardalis_Acr AHSQQEIFSATRRTPFETEIHQYDWKQEMSSRPDHEREQFPPAQKQMWNNRENSWVFQDT 517

Trioceros_affinis_ENAM_Acr AHSQQEIFSATRRTPFETEIQQYDWKQQMSSRPDHEREQFSRAQNQMWNNRENSWVFQDK 517

Trioceros_balebicornutus_ENAM_Acr AHSQQEIFSATRRTPFETEIQQYDWKQQMSSRPDHEREQFPPAQNQMWNNRENSWVFQDK 517

Trioceros_harennae_ENAM_Acr AHSQQEIFSATRRTPFETEIQQYDWKQQMSSRPDHEREQFPPAQNQMWNNRENSWVFQDK 517

Anolis_apletophallus_Pleu VLSHQEVNSATRRTPFEIESHQYDWREQPFKHIDQKRE-----HSPTWSNREDTQLFQE- 544

Anolis_tropidonotus_ENAM_Pleur ALSHQEVNSAT-RTPFEIETHQYDWREQPFKHIDQKREQFPPAQTPTWSNREDTQLFQE- 548

Anolis_sagrei_ordinatus_Pleu TLSHQGVNSATRRTPFEIETHQHDWREQPFKHIDQKREQFLPEQTPTWSNREDSRLFQE- 549

Anolis_carolinensis_Pleu ALSHQGVNLATRRTPFEIETHQYDWREQPFNHIDQKRGQFLSAQTPTWNNREASQLFQE- 549

Ctenosaura_bakeri_Pleu TFSYQGVISATRRTPFETEMQQYDWNDRPVNHPNEKREQFSPAQNPMWNNQEDLQVFKE- 559

Cyclura_pinguis_Pleu TFSNQGVISATRRTPFEMEMQQYDWKDRPFNHPNEKREQFSPAQNPMWNNQEDLQVFQE- 559

Iguana_delicatissima_Pleu TFSHQRVISATRRTPLEIEMQRYDWKDRPFNHPNKKREQFSPAENPMWNNQEDLQVIQE- 559

Gambelia_wislizenii_Pleu TFSHQGGISTTRRTPFEIETHQYDWKEQPFNHLDEKREQFQPAQNPMWNNQEDLQVFQE- 559

Laemanctus_serratus_ENAM_Pleur TFSHQGVISATRRTPFEIETHQYDWKEQPFNYPDQKREQFLPAQNPMWNNQE-SQVFQE- 558

Phrynosoma_blainvillii_Pleu TFSHQGVISNTGRTPFEIETHQYDWKEQPFNHPDQQKEQFLSAQNPMWNNQEDSQVFHK- 567

Phrynosoma_platyrhinos_Pleu TFSHQGVISATGRTPFEIETHQYDWKEQPFNHPDQQKEQFLSAQNPMWNNQEDSQVFHK- 570

Sceloporus_chrysostictus_ENAM_Pleur TFSHQGLISATGRTPFEVETHQYDWKEQPFNHPDQKREQFLPEENPMWNNQEDSQMFQK- 565

Urosaurus_nigricaudus_Pleu TFSHQGLISGTGRTPFETETHQYNWKEQPFNHPDQKREQFLPEQNPMWNNQEDSQVFQE- 570

Sceloporus_occidentalis_Pleu TFSHQGLISATGRTPFEIETHQYDWKEQPFNHPDQKREQFLPEQNPMWNNQEGSQVFQE- 570

Sceloporus_tristichus_Pleu TFSHQGLISATGRTPFEIETHQYDWKEQPFNHPDQKREQFLPEQNPMWNNQEGSQVFQE- 570

Sceloporus_undulatus_Pleu TFSHQGLISATGRTPFEIETHQYDWKEQPFNHPDQKREQFLPEQNPMWNNQEGSQVFQE- 570

* : * ***:* * :*. . :.:: .. ..:* ::.

Acanthocercus_cyanogaster_ENAM_Acr TPPRYDSRYSFNPDHQHGQATYSERNS----YQQNGHPGSPRTWEERKDSPV---AGQRE 598

Acanthocercus_minutus_ENAM_Acr TPPRYDSRYSFNPDHQHGQAIYSERDS----YQQNGHPSSPRTWEERKDSPV---AGQRE 593

Xenagama_zonura_ENAM_Acr TPPRYDSRYSFNPDHQHGQAIYSERDS----YQQNGHTSSPRTWEERKDSPV---AGQRE 593

Agama_doriae_ENAM_Acr TPPRYNSRYSLDPDHQQGQAIYSERNS----YQQNGHANSPRAWEERIDSPV---AGQRE 595

Laudakia_wui_Acr TPPRYNSMYSLDSDHQRGQAIYSERNS----YQQNGHPNSPRTWEERKDSPV---TGQRE 602

Phrynocephalus_forsythii_Acr TPPRYNSMYSLDSDHPRGHSIYSERNS----YQQNGHPNSPRTXEEREDSPV---TGQRE 593

Phrynocephalus_guinanensis_Acr TPPRYNSMYSLDSDHPRGHSIYFERNS----YQQNGHPNSPRTWEEREDSSV---TGQRE 593

Phrynocephalus_putjatai_Acr TPPRYNSMYSLDSDHPRGHSIYFERNS----YQQNGHPNSPRTWEEREDSSV---TGQRE 593

Phrynocephalus_vlangalii_Acr TPPRSNSMYSLDSDHPRGHSIYSERNP----YQQNGHPNSPRTWEEREDSSV---TGQRE 594

Phrynocephalus_versicolor_Acr SPPRYNSMYSLHSDHPRGHSIYSERNS----YQQNGHPNSPRTWEEREDSPV---TGQRE 594

Intellagama_lesueurii_Acr APPRYNSMYSLDSVHQRGQAIYSERNSFRNPYQQNGHPISSRTWEERKDSPVTGSTGQRE 602

Pogona_vitticeps_Acr APPRYNSMYSLDSIHQRGQEIYSERNSFRNPYQQNGHPSTSRTWEERKDSPVTGSTGQRE 603

Bradypodion_pumilum_Acr TPPRYKTMYSLDSTHQRGQAVYS--------YQQNGRPISSGTWEERKDSPIISSANQRK 586

Bradypodion_ventrale_Acr TPPRYKTMYSLDSTHQRGHTVYS--------YQQNGRPISSGTWEERKDSPIISSANQRK 586

Chamaeleo_calyptratus_ENAM_Acr TPPRYKTMHSLDSTHQRGQAVYS--------HQQNGHPISPGTWEERKDSPIISSANQRK 570

Chamaeleo_dilepis_ENAM_Acr TPPRYKTMYSLDSMHQRGQAVYS--------HQQNGHPISPGTWEERKDSPIISSANQRK 571

Chamaeleo_gracilis_ENAM_Acr TPPRYKTMYSLDSTHQRGQAVYS--------HQQNGHPISPGTWEERKDSPIISSTNQRK 570

Chamaeleo_laevigatus_ENAM_Acr TPPRYKTMYSLDSTLQRGQAVYS--------HQQSGHPISPGTWEERKDSPIISSANQRK 564

Furcifer_pardalis_Acr TPPRYKTMHSLDSTHQRGQATFS--------YQQNGHPISPGTWEERKDSPIISSANQRK 569

Trioceros_affinis_ENAM_Acr TPPRYKTMYSLDSAHQRGQAVYS--------YQQNGHPISPGTWEGRKDSPIMSSANQRK 569

Trioceros_balebicornutus_ENAM_Acr TPPRYKTMYSLDSAHQRGQAVYS--------YQQNGHPISPGTWEERKDSPIISSAYQRK 569

Trioceros_harennae_ENAM_Acr TPPRYKTMYSLDSAHQRGQAVYS--------YQQNGHPISPGTWEERKDSPIISSANQRK 569

Anolis_apletophallus_Pleu APPRHRSMYSSGSFNQREISSYSGKKS----YDQYTHPFHPRAWEDRENSPTISPSDQRE 600

Anolis_tropidonotus_ENAM_Pleur APPRYRSMYSSGSFNQREMPSYSGKKS----YDQYTHPFLPRAWEDRENSPTISPSDQRE 604

Anolis_sagrei_ordinatus_Pleu APPRHRSMYSSGSFNQREMPSYSGKKS----YDQYTHPFLPRAWEDRENSPTISPSDQRE 605

Anolis_carolinensis_Pleu APPRQSSMYSSGSFNQREMPTYSGKKS----YNQYTHPFPPRAWEDREHSPTISPSDQRE 605

Ctenosaura_bakeri_Pleu APPRYNSMYSSGTFNQRELPSSLGKTP----YGQHIRSFPPREWEDRENSPTISPSDQRE 615

Cyclura_pinguis_Pleu APPRYNSMYSSGTFNQRELPSSLGKTP----YGQHIHSFPPRAREDRENSPTISPSDQRE 615

Iguana_delicatissima_Pleu APPRYNSMYSSGTFNQRELPSSLGKTP----YGQHIHSFSPRAWEDRENSPTISPSDQRE 615

Gambelia_wislizenii_Pleu VPPRYNSMHSSGSFNQRGLPTYSGKNS----YDQHVHFFPPRAWEDRENAPTIRPSDQRE 615

Laemanctus_serratus_ENAM_Pleur APPRYNSMYSSGSFNQRGLPTYSEKSP----YSQHVHSFPPKAWEDREISPTISPPDQRE 614

Phrynosoma_blainvillii_Pleu APPRSSSMYSSGPFNQRGLPTYLGKNS----YSQ---SITPRAWEDRENSPAISPSGQRD 620

Phrynosoma_platyrhinos_Pleu APPRHNSMYSSGPFNQRGLPTYLGKNS----YSQ---SIIPGAWEDRENSPAISPSGQRD 623

Sceloporus_chrysostictus_ENAM_Pleur APPRYNSMYSSDSFNQRGLPTYLGENS----YSQ---PIPPRAWEDRDNSPTTSPSGQRE 618

Urosaurus_nigricaudus_Pleu APPRYNSMYASDSFNQRGLPTNLGKNS----YSQ---PIPPRAWDDRDNSPTISPSGQRE 623

Sceloporus_occidentalis_Pleu APPRYNSIYSSDSFNQRGLPIHLEKNS----YSQ---PIPPRAWEDRDNSPTISPSGQME 623

Sceloporus_tristichus_Pleu APPRYNSVYSSDYFNQRGLPTHLEKNS----YSQ---PIPPRAWEDRDSSPTISPSGQME 623

Sceloporus_undulatus_Pleu APPRYNSVYSSDYFNQRGLPTHLEKNS----YSQ---PIPPRAWEDRDSSPTISPSGQME 623

*** : :: : : * . : * :. . * .

Acanthocercus_cyanogaster_ENAM_Acr SLSYSPLLPSNQIQKDTYHRGVQQERKPYPSENPWIYEKHLQEDDRQYKNPSYNLAQHHM 658

Acanthocercus_minutus_ENAM_Acr GLSYSPLLPSNQIQKDTYHRGVQQERKPYPSENPWIYEKHLQDDDRQHKNPSYNLAQHHM 653

Xenagama_zonura_ENAM_Acr SLSYSPLLPSNQIQKDTYHRGVQQERKPYPSENPWIYEKHLQDDDRQHKNPSYNLAQHHM 653

Agama_doriae_ENAM_Acr SLSYSPVLPSDQIQKDTYHRGVQQEHKPYPSQNPWIYEKHLLDDDRQYKNPSYNLAQHHT 655

Laudakia_wui_Acr GLSYSPVLPSNQMQKDTYHRGVQQEHEPYPSQNPWIYEKRLLDDDTQYKNPSYNPAQHHM 662

Phrynocephalus_forsythii_Acr SLSYSPVLPSNQMQKDTYHRGVQQEHKPYPSQDPWIYEKHLLDDDRQYKNPSYNPAQHHM 653

Phrynocephalus_guinanensis_Acr SLSYSPVLPSNQMQKDTYRRGVQQEHKPYPSQDPWIYEKHLLDDDRQYK----NPAQHHM 649

Phrynocephalus_putjatai_Acr SLSYSPVLPSNQMQKDTYRRGVQQEHKPYPSQDPWIYEKHLLDDERQYKNPSYNPAQHHM 653

Phrynocephalus_vlangalii_Acr SLSYSPVLPSNQMQKDTYRRGIQQEHKPHPSQDPWIYEKHLLDDDRQYKNPSYNPAQHHM 654

Phrynocephalus_versicolor_Acr SLSYSPVLPSNQMQKDTYRRGVQQEHKPYPSQDPWIYENHLLDDDRQYKNPSYNPAQHHM 654

Intellagama_lesueurii_Acr RLSYSPMLPSNQMQKNTYHRSVQQEHEAHPNQNPWIDEKHLLDVDREYKNPSYNPAQYHM 662

Pogona_vitticeps_Acr RLSYSPILPSNQMQKNHYHRSVQQEHEAHPNQNPWVDEKHLLDVDREYKNPSYNPPQYPM 663

Bradypodion_pumilum_Acr SLSYSPSMPSNQKQ-NTYYRSVQEENEPPPNQNPWTHENNLLDFDRRYKNLPYDPAQHQM 645

Bradypodion_ventrale_Acr SLSYSPSMPSNQMQ-NTYYRSVQEENEPPPNQNPWTHENNLLDFDRRYKNLPYDPAQHQI 645

Chamaeleo_calyptratus_ENAM_Acr SLSYSPSMPSNQMQ-NSYYRRVQEENESPPNQNPWTHENNLLDFDRQYKNPPYDPAQHQM 629

Chamaeleo_dilepis_ENAM_Acr SLSYSPSMPSNQMQ-NSYYRRIQEENESPPNQNPWTHENNLLDFDRQYKNRPYDPAQRQM 630

Chamaeleo_gracilis_ENAM_Acr SLSYSPSIPSNQMQ-NSYYRRIQEENESPPNQNPWTHENNLLDFDRQYKNPPYDPAQHHM 629

Chamaeleo_laevigatus_ENAM_Acr SLSYSPSMPSNHMQ-NSYYRRIQEENGSPPNQNPWTHENNLLDFDRQYKNPPYDPAQHQM 623

Furcifer_pardalis_Acr SLSYSPSMPSNQMQ-DTYHRRVQEENEPPPNQNPWTHENNLLDFDRQYKNPPYEPAQHQM 628

Trioceros_affinis_ENAM_Acr SLSYSPSMPSNQMQ-NTYYRRVQEENEPPPNPNPWRHENNLLDFDRQYKNAPYDPAQHQM 628

Trioceros_balebicornutus_ENAM_Acr SLSYSPSMPSNQMQ-NTYYRRVQEENEPPPNPNPWRHENNLLDFDRQYKNAPYDPAQHQM 628

Trioceros_harennae_ENAM_Acr SLSYSPSMPSNQMQ-NTYYRRVQEENEPPPNPNPWRHENNLLDFDRQYKNAPYDPAQHQM 628

Anolis_apletophallus_Pleu SPPYSSASPSDQIQRKIYYRRIQPEYEPYPRQDPWAREQHLLDLDNQYSNSPYNPAHHQT 660

Anolis_tropidonotus_ENAM_Pleur SPPYSPTSPSDQIQRKIYYRRIQPEYEPYPRQDPWTREQQLLDPDNQYSNSPYNPAHHQT 664

Anolis_sagrei_ordinatus_Pleu SPPYSPASPSDQIQRKIYYRRIQPEYEPYPRQDPWTRE-HLLDLDKQYNNSPYNPAHHQT 664

Anolis_carolinensis_Pleu SSPYPPVSPSDQIQRNTYYRRIQPEYEPYSRQDPWTREQHLHDPDNQYSNSPYNPTHHQT 665

Ctenosaura_bakeri_Pleu SPSYSPASPSDKMQRNTYYRRVQPEYEPYPRQDPWTREQHLVDLKDQYSNSPYKPTLHHA 675

Cyclura_pinguis_Pleu SPSYSPALPSDQMQRNTYYRRVQPEYEPYPRQDPWTREQHLLDLEDQYSNSPYKPTHHHA 675

Iguana_delicatissima_Pleu SPSYSPASPSDQMQRNTYYRRVQPEYEPYPRQDPWTHEQHLLDLDDQYGNSPYKPTHHHA 675

Gambelia_wislizenii_Pleu SRSYSPASPSEQKQRNTYYRRVQPEYEPYPRQGPWTREQHLLDLDNQYSNSPYNPTRHHA 675

Laemanctus_serratus_ENAM_Pleur SPSYSPA--SDQTQRNSYYRRVQPEYEPHPRQDPWTREQHLLDLDNQYSNSPYNPNHPHA 672

Phrynosoma_blainvillii_Pleu NPSYSPASPSHQMQRNTYYRRVQPEYEPHPRQDPWTREQHLLDRDNQYSNSAYNPINQHA 680

Phrynosoma_platyrhinos_Pleu NPSYSPASPSHQMQRNTYYRRVQPEYEQHPRQDPWTREQHLLDRDNQYSNSPYNPINQHA 683

Sceloporus_chrysostictus_ENAM_Pleur NPSYFPASPSHQMQRNTYYRRVQPEYVPHPRQDPWTRE-HLLDRDNQYSNSQYNPTHRHA 677

Urosaurus_nigricaudus_Pleu NPSYSPALPSHQMQRNAYYRRVEPEYVPHPRQDPWTREQHLLDRENQFSNSPYNPTHHSA 683

Sceloporus_occidentalis_Pleu NPSYSPASPSHQMQRNTYYRRVQPEYVPHPRQDPWTREQHLFDRDNQYSNSPYNPTHHHE 683

Sceloporus_tristichus_Pleu NPSYSPASPSHQMQRNTYYRRVQPEYVPHPRQDPWTREQHLFDRDNQYSNSPHNPTHHHE 683

Sceloporus_undulatus_Pleu NPSYSPASPSHQIQRNTYYRRVQPEYVPHPRQDPWTREQHLFDRDNQYSNSPHNPTHHHE 683

.* . *.: * . * * :: * . .** * .* : . .. .

Acanthocercus_cyanogaster_ENAM_Acr HSGYPTETSPSERANLPYEEINQWTPGKRTLHPGMEYLKQVENMPYHTN---GQQDQYME 715

Acanthocercus_minutus_ENAM_Acr HSGYPTETSPNERANLAYEEINQWTPGKRTLHPGTEYLKQVENMPYHTN---GQQDQYME 710

Xenagama_zonura_ENAM_Acr HSGYPTETSPNERANLAYEEINQWTPGKRTLHPGTEYLKQVENMPYHTN---GQQDQYME 710

Agama_doriae_ENAM_Acr PSGYPTESLANERANLPYEDINQWTPGKRTLRPGTDYLKQVEHMPYHTNGMSGQKDQYME 715

Laudakia_wui_Acr HPGYSTETPTNKRADLPYGEINQWSPGKRTLGPHTEHLKQVENIPYHTNGMFGQQDPNME 722

Phrynocephalus_forsythii_Acr HPGYSTETPANERVNLPYEEINQWTPGKGTLGPGTEYLKQVENIPYHTNGMFGQKDQYME 713

Phrynocephalus_guinanensis_Acr HQGYSTETPANERVNLPYEEINQWTPGKRTLGPGTEYLKQVENIPYHTNGMFGQKDQYME 709

Phrynocephalus_putjatai_Acr HQGYSTETPANERVNLPYEEINQWTPGKRTLGPGTEYLKQVENIPYHTNGMFGQKDQYME 713

Phrynocephalus_vlangalii_Acr HLGYSTETPANERVNLPYEEINQWTPGKRTLGPGTEYLKQVENIPYHTNGMFGQKDQYME 714

Phrynocephalus_versicolor_Acr HPGYSTETPANERVNLPYEEINQWTPGKRTLGPGTEYLKQMDNIPYHTNGMFGQKDQYME 714

Intellagama_lesueurii_Acr HPRYSTETPPNERDNLPYEEMNQWTPEKHTLIPGTEHLKQVENIPYHTNGMFRQKDQNME 722

Pogona_vitticeps_Acr HPRFSTETPPNERDNLPYERMNQWTPEKHIFIPVTEHLKPVENIPYHTNGMFGQKDQNMG 723

Bradypodion_pumilum_Acr YSRYSTEAAANERHNLPYEKINQWAQEKHILVPSTEHLKQVENIPYDTNGMFGQKDQNMM 705

Bradypodion_ventrale_Acr YSRYSTEAAANERHNLPYEKINQWAQEKHILVPSTEHLKQVENIPYDTNGIFGQKDQNMM 705

Chamaeleo_calyptratus_ENAM_Acr YSRYSTEAAANERHNLPYEKINQWAQEKHILVPGTEHLKQVENIPYDTNGMFGQKEQNMM 689

Chamaeleo_dilepis_ENAM_Acr YSRYSTEAATNERHNLPYEKVNQWAQEKHILVPGTEHLKQAENIPYDTNGMFGQKEQNMI 690

Chamaeleo_gracilis_ENAM_Acr YSRYSTEAATNERHNLPYEKVNQWAQEKHILVPGTEHLKQAENIPYDTNVMFRQKEQNMK 689

Chamaeleo_laevigatus_ENAM_Acr YSRYSTEAAANERHSLPYEKINQWAQEKNILVPGTEHLKQVENIPYDTNGMFGQKEQNMM 683

Furcifer_pardalis_Acr YSRYSTEAAANERHNLPYEKINQWAQEKHILVPGTEHLKHVENIPYDTNGIFGQKDHNMM 688

Trioceros_affinis_ENAM_Acr YLRHSTEAAANERHNLPYEKINQWAQEKHIVVPGTEHLKQVENIPYDTNGMFGQKDQNMM 688

Trioceros_balebicornutus_ENAM_Acr YSRYSTEAAANERHNLPYEKINQWAQEKHLLVPGTEHLKQVENIPYNTNGMFEQKDRNMM 688

Trioceros_harennae_ENAM_Acr YSRYSTEAAAKERHNLPYKKINQWAQEKHILVPGTEHLKQVENIPYDTNGMFEQKDRNMM 688

Anolis_apletophallus_Pleu YPKYATENPPSEISNLPYQKINQWTQEEHPPVHTAGHLRQVENAPYHTNSMFEQGERKFQ 720

Anolis_tropidonotus_ENAM_Pleur YPKYATENPPSKISNLPYQKINQWTQEEHSPVHTAGHLSQVGNVPYHTNSMFEQGERTFQ 724

Anolis_sagrei_ordinatus_Pleu YPKYATENPPSEISNLPYQKINQWTQEEHSPIQSAGHLRQVENVPYHTNSMFEQGERKFQ 724

Anolis_carolinensis_Pleu YQKYTTENPPSEISNLPYRKINQWTQEEHSPVHTAGHLRQMENVPYRTNSKFEQGERKFQ 725

Ctenosaura_bakeri_Pleu YPKYSTENRASEISNLAHEKMKQWTQEENPPVYSAAHLRQMENVPYHTNSMFGQREREFQ 735

Cyclura_pinguis_Pleu YPKYSTENRASEMSNLPHEKMKQWTQDENPLVHSAAHLRQMENVPYHTNGMFGQREREFQ 735

Iguana_delicatissima_Pleu YPKYSTENRASEISNLPREKMKQWTQEENPPVHSAAHLRQMENVPYHTNSMFGQREREFQ 735

Gambelia_wislizenii_Pleu YPKYSTDNPASEISSLPYEKINQWTQEENPPVHSAGHLRHMENVPYHTNSMSGQRERKFQ 735

Laemanctus_serratus_ENAM_Pleur YPKYSTDNPTSEISNLPYDKINRWTQEEHPLIHTAGHLRQVENVPYHTNSMFGQRERKFQ 732

Phrynosoma_blainvillii_Pleu NPKYTIENPASEISNLPYEKINQWTQEEYPPVHSAGHLRQVGNAPYQSNSMFGHRERKFQ 740

Phrynosoma_platyrhinos_Pleu NPKYTIENPASEISNLPYEKINQWTQEEYPPVHNAGHLRQVENVPYQSNSMFGHRERKFQ 743

Sceloporus_chrysostictus_ENAM_Pleur NSKYTIENPASEISNLPYEKINQWTQEEHPPVHSGGHLRQVENVPYQSKSMLGQRERKFQ 737

Urosaurus_nigricaudus_Pleu NSKYTIENPASEISNLPYEKINQWTQEEHIPVHSEGHLRQVENVPYQSKSMFGQRERIFQ 743

Sceloporus_occidentalis_Pleu NTKYIIENPASEISNLPYEKINQWTQEERPPVHSGVHLRQVENVPYQSKSMFGQRERKFQ 743

Sceloporus_tristichus_Pleu NTRYIIENPASEISNLPYEKINQWTQEERPPVHSGGHLRQVENVPYQSKSMFGQRERKFQ 743

Sceloporus_undulatus_Pleu NTRYIIENPASEISNLPYEKINQWTQEERPPVHSGGHLRQVENVPYQSKSMFGQRERKFQ 743

. : ..: .*. :::*: : :* : ** :: : : :

Acanthocercus_cyanogaster_ENAM_Acr NRGSNPPPQSSSLPQGGSQFRERSSWVRPML----DPSSQKET-SLHFNSYSTDFRRKSD 770

Acanthocercus_minutus_ENAM_Acr NRGSNPPPQSPSLPQWGSQYGERNSWVRPML----DPSSQKET-SLHFNSYSTDFRRKSD 765

Xenagama_zonura_ENAM_Acr NRGSNPPPQSPSLPQWGSQYGERNSWVRPML----DPSSQKET-SLHFNSYSTDFRRKSD 765

Agama_doriae_ENAM_Acr NQASNPPPQSPSPSQWGSQHGERNSWARPML----DPSSQKET-SLYFNTYSTDFRRKSD 770

Laudakia_wui_Acr NRGSNPPPQSTSLSQRESQYGERNTWIHPMP----GPSSRKET-SLYFNAYSTDFRRKSD 777

Phrynocephalus_forsythii_Acr NRGSNLPPQSTSLSQWGSQYGERNSWVPPML----GPSSQKETSSLYFNAYSTDFRRKSD 769

Phrynocephalus_guinanensis_Acr NRWSNPPPQSTSLSQRGSQYGERNSWVPPML----GPSSQKET-SLYFNAYSTDFRRKSD 764

Phrynocephalus_putjatai_Acr NRWSNPPPQSTSLSPRGSQYGERNSWVPPML----GPSSQKET-SLYFNAYSTDFRRKSD 768

Phrynocephalus_vlangalii_Acr NRGSNPPPQSTSLSQRGSQYGERNSWVPPML----GPSSQKET-SLYFNAYSTDFRRKSD 769

Phrynocephalus_versicolor_Acr NRGSNPPPQSTSLSQRGSQYGERNSWVPPML----GPSSQKET-SLSFNAYSTDFRRKSD 769

Intellagama_lesueurii_Acr NQGSNSPPQSTSLSQRGAQYAERSTWVPPIL----DPSPQKET-SPYFNAYSTDLRRKSD 777

Pogona_vitticeps_Acr NQGSNSPPQSISLSQRGAQYAERSTWVPPIL----GPSPQKET-SPYFNAYSTDLRRKSD 778

Bradypodion_pumilum_Acr KQGSDSPPQGTTLSQRGAQYAERNPWVSPIL----DPFPRKET-LPYFNTHSTDFRRKSD 760

Bradypodion_ventrale_Acr KQGSDSPPQGTTLSQRGAQYAERNPWVSPII----DPFPQKET-LPYFNTHSTDFRRKSD 760

Chamaeleo_calyptratus_ENAM_Acr MQGSDSPPQGTTLSQRGAQYAERNPWVSPIH----NPFPQKET-LPYFNTYSTDFRRKSE 744

Chamaeleo_dilepis_ENAM_Acr TQWSDSPPQGTTLSQRGAHYAERNPLVSPIL----DPIPQKET-LPYFNTYSTDFRRKS- 744

Chamaeleo_gracilis_ENAM_Acr TQGSDSPPQGTTLSQRGAHYAERNPWVSPIL----DPFPLKET-LPYFNTYSTDFRRKSE 744

Chamaeleo_laevigatus_ENAM_Acr TQGSDSPPQGTTLSQRGAQYAERNPWVSPIL----DPFPQKET-LPYFNTYSTDFRRKSD 738

Furcifer_pardalis_Acr KQGSDSPPQGTTLSQRGAQYAERNPWASPIF----DPFPQKET-LPYFNTYSTDFRRKSD 743

Trioceros_affinis_ENAM_Acr KQGSDSPPEGTTLSQQGAQYAERNPRVSPIL----DPFPQKET-LPYFNTYSTDFRRKSG 743

Trioceros_balebicornutus_ENAM_Acr KQGSDSPPEGTTLSQQGAQYAERKPRVSPIL----DPFPRKET-LPYFNTYSTDFRRKSD 743

Trioceros_harennae_ENAM_Acr KQGSDSPPEGTTLSQQGAQYAERNPWVSPIL----DPFPRKET-LPYFNTYSTDFRRKSD 743

Anolis_apletophallus_Pleu NVGSSSPQQSNTF-QEEAQYADRNTWIPQRV----DPSAQKET-APYFNIYSTDFRG-NP 773

Anolis_tropidonotus_ENAM_Pleur NVGPSSPQQSNTF-QEEAQYAERNTWIPQRV----DPSAQKET-APYFNIYSKDFRR-NP 777

Anolis_sagrei_ordinatus_Pleu NVRSSSPQQSNTF-QEEAQYAERNTWFPQRV----DPSAQKET-GPYFNIYSTDFRG-NP 777

Anolis_carolinensis_Pleu NVGSGSPQQRNTF-QEEAQYAERNTWVPQRV----DPSAQKET-APYYNIYSTDFRG-NP 778

Ctenosaura_bakeri_Pleu NVEPISPQQRITF-QDEAQHAERNTWIPQMV----DPSAQKGT-PPYFNIYSTDFRR-NP 788

Cyclura_pinguis_Pleu NVEPISPQQRITF-QDEAQYAERNTWIPQMV----DPSAQKGT-PPYFNIYSTDFRR-NP 788

Iguana_delicatissima_Pleu NVEPISP-QRITF-QDEAQYAERNTWIPQMV----DPSAQKGT-QPYFNIYSTDFRR-NP 787

Gambelia_wislizenii_Pleu NIGSNSPQQRISF-PEEAEYTERNTWSPQSL----DPSAQKES-LPYLNIYSTDFRR-KP 788

Laemanctus_serratus_ENAM_Pleur NVGSNSPQQSITF-QEEAQYAEKNRWIPPRASPRGDPSAQKES-PPYFNVYSTDLRR-NP 789

Phrynosoma_blainvillii_Pleu NVGSNSPQESITV-QEEAQYAERNMWIPQRV----DLSAQKET-PTYFNIYSTDIRR-NP 793

Phrynosoma_platyrhinos_Pleu NVGPNSPQESITV-QEEAQYAERSMWVPQRV----DLSAQKET-PPYFNIYSTDIRR-NP 796

Sceloporus_chrysostictus_ENAM_Pleur NVGSSSPQQSIAI-QEAAQYAERNTWIPQRI----DQSAQKET-PPYFNIYSTDVRR-NP 790

Urosaurus_nigricaudus_Pleu NVGANLPQQSIAV-QEEAQYPGRNTWIPQRI----DPSAQKET-PPYFNIYSTDVRR-NP 796

Sceloporus_occidentalis_Pleu NAESNSPQQSIAV-QEAAQYAERNSWIPQRR----DPSAQKET-PPYINIYSTDVRR-NP 796

Sceloporus_tristichus_Pleu NAESNFPQQSIAV-QEEAQYAERNSWIPQRR----DPSAQKET-PPYINIYSTDIRR-NP 796

Sceloporus_undulatus_Pleu NAESNFPQQSIAV-QEEAQYAERNSWIPQRR----DPSAQKET-PPYINIYSTDIRR-NP 796

. * : : :.. :. . . * : * :*.*.* .

Acanthocercus_cyanogaster_ENAM_Acr DDIDDTGKSTRVSAPVSSVDVAGRKHYPDTARYSIHYPGRDRTITGDSPAEPLCCGDDSP 830

Acanthocercus_minutus_ENAM_Acr YDIDDTGKSTHVSAPVSSVDVAGRRHYPDTARYSIHYPGRDRTITGDSPAEPLCCGDDSP 825

Xenagama_zonura_ENAM_Acr YDIDDTGKSTHVSAPVSSVDVAGRRHYPDTARYSIHYPGRDRTITGDSPAEPLCCGDDSP 825

Agama_doriae_ENAM_Acr DDVDDTGKSARVSAPASSVDVAGRRHYPDTGRYPKSYPGGDRTITGDSPADHLCCGDDSP 830

Laudakia_wui_Acr DDIDDTGKTTHASAPISSVDAAGRRHYPDTVRYSKDYPGEDRTITGHSPENHLCCADDSP 837

Phrynocephalus_forsythii_Acr DDIDDTGKSTRVSAPVSNVNVAGRRHYPDTARYSKDYPGEDRTITGPSPADHLCCADDSP 829

Phrynocephalus_guinanensis_Acr DDIDDTGKSTRVSAPVSSVNVAGRRHYPDTAKYSKDYPGEDKTITGPSPADHLCCADDSP 824

Phrynocephalus_putjatai_Acr DDIDDTGKSTRVSAPVSSVNVAGRRHYPDTAKYSKDYPGEDKTITGPSPADHLCCADDSP 828

Phrynocephalus_vlangalii_Acr DDIDDTGKSTRVSAPVSSVNVAGRRHYPDTARYSKDYPGEDTTITGPSPADHLCCADDSP 829

Phrynocephalus_versicolor_Acr DDIDDTGKSTHVSAPVSSVNVAGRRHYPDTAKYSKDYPGEDKTITGPSPADHLCCADDSP 829

Intellagama_lesueurii_Acr DDIDDTGKITHVSAPLSSINVAGRRHYPDTTRYSEDYRGEHRTITAHSTADHLCCGEDSP 837

Pogona_vitticeps_Acr DDIDDTGKITHGSAPLSSVNVAGERHYPDTRRYSEDYRGEYRTITTHSTADHLCCAEDSP 838

Bradypodion_pumilum_Acr DDTDDVSKITQGSAPISGVNVAGRKYNPNTLRYSEDYPREHRTTHPHSTANHLCCVGDSP 820

Bradypodion_ventrale_Acr DDTDDVSKITQGSAPISGVNVVGRKYNPNTLRYSEDYPREHRTTYP--TANHLCCVGDSP 818

Chamaeleo_calyptratus_ENAM_Acr DDTDDASKITQGSAPVSGVNVAGRN-NPDTLRYSEDYPREHRTINP---TNHLCCVGDSP 800

Chamaeleo_dilepis_ENAM_Acr ---DDASKITQGSVPVSGVNVAGRRYNPDTLRYSEDYPREHRTINP---TNHLCCVGDSP 798

Chamaeleo_gracilis_ENAM_Acr DDTDDASKIALGSATVSGVNVAERRYNPDTLRYSEDYPREHRTINP---TNHLCCVGDSS 801

Chamaeleo_laevigatus_ENAM_Acr DDTDDASKITQGSAPVSGVNVAGRRYNPDTLRYSEDYPREHRTITP---TNHLCCVGDSP 795

Furcifer_pardalis_Acr DDTDDARKITQGSAPVSSVNVAGRRYNPDTLRYSEDYPREHRTATPHSTANHLCCVGDSP 803

Trioceros_affinis_ENAM_Acr GDTDDASKITQGSAPISGVNVAGRRYNPDTLRYSEDYPREHRTATPHSTANHLCCVGDSQ 803

Trioceros_balebicornutus_ENAM_Acr SDTDDASKITQGSAPVSDANVAGRRYNPDTLRYSEDYPREHRTATPHSTANHLCCVGASQ 803

Trioceros_harennae_ENAM_Acr GDTDDASKITQGSAPVSDVNVAGRRYNPDTLRYSEDYPREHRTATPHSTANHLCCLGDSQ 803

Anolis_apletophallus_Pleu THAEDKERIMHVNAPMFTENAPGRRHYLDRMGYSDDYPKERRMITSQSTTNQLCCADDSP 833

Anolis_tropidonotus_ENAM_Pleur THAEDKERIIHVNAPIFTENAPGRRHYLDRMGYSEDYPKGRRMITSQSTTNQLCCADDSP 837

Anolis_sagrei_ordinatus_Pleu THVEDRERIIHVNAPISTENAPGRRHYLDRMGYSDDYPKERRMITSQSTTNQLCCADDSP 837

Anolis_carolinensis_Pleu THVEDRERIIHMNAPISTENAPRRRHYLDRMGYSDDYPKERRMITSQSTANQLCCADDSP 838

Ctenosaura_bakeri_Pleu THTEDTREIIRVNTPFSRENVPGGRHYLDRTGYSDDYPTEHRMMTSHSTPNQLCCADDPP 848

Cyclura_pinguis_Pleu THAEDTRGIIGMNAPLSGENVPGGRHYLERTGYSDDYPTERRMMTSHSTPNQLCCADDSP 848

Iguana_delicatissima_Pleu THAEDTRGIIHVNAPLSGENVPGGTHYLDRTGYSDDYPTERRTMTSHSTPNQLCCADDSP 847

Gambelia_wislizenii_Pleu THAEDTGGVIHLNAPISSENAPGRRHYLERRGYSDDYPREGRMMTSHSTANQLCCAEDSP 848

Laemanctus_serratus_ENAM_Pleur AHVEDPGGIIHVNAPISSENGPGRRHYLDRMGYSDDYPNERRLITSHSRTNQLCCADDSP 849

Phrynosoma_blainvillii_Pleu THAEDTGGIIHANAPISSENAPGRRHYLGRMGYSDDYQTERRMMTSYSTANQLCCADDSP 853

Phrynosoma_platyrhinos_Pleu THAEDTGGIIHANTPISSENTPGRRHYLGRMGYSDDYQTERRMMTSYSTANQLCCADDSP 856

Sceloporus_chrysostictus_ENAM_Pleur TQAEDTGGIIHVNAPISSDNAPGRRHYLDRMGYSDDHLTERRMMTSHSIANPLCCADDPP 850

Urosaurus_nigricaudus_Pleu THAEDTGGIIHVNAPISSENAPGRRHYLDRMGYSDDYLPERRMMTSHSIANQLCCADDPP 856

Sceloporus_occidentalis_Pleu THAEDTGGIIHVNAPISSENAPGRRHYLDRMGYSDDYLTERRMMSSPSIANQLCCADDPP 856

Sceloporus_tristichus_Pleu THAEDTGGIIHVNAPISNENAPGRRHYLDRMGYSDDYLTERRMMSSPSIANQLCCADDPP 856

Sceloporus_undulatus_Pleu THAEDTGGIIHVNAPISNENAPGRRHYLDRMGYSDDYLTERRMMSSPSIANQLCCADDPP 856

:* ... : *. : : *** .

Acanthocercus_cyanogaster_ENAM_Acr VTNEGRPVPLR-----RFPPWEGTVSTAYSEGSHGKHARHAPSPAGIQSNFPFRLSKNQK 885

Acanthocercus_minutus_ENAM_Acr VTNEGRPAPLR-----RFPPWEGTVSTAYSEGSHGKHARHAPSPAGIQSNFPFRLGKNQK 880

Xenagama_zonura_ENAM_Acr VTNEGRPAPLR-----RFPPWEGTVSTAYSEGSHGKHARHAPSPAGIQSNFPFRLGKNQK 880

Agama_doriae_ENAM_Acr VMNEGRPAPLRNVPQFRFPPWEETVNTAYSEGSHGKHARHAPSPAGIQSNFPFRMSKNQK 890

Laudakia_wui_Acr ATKEGRPVPLRSAPQFRFPPWEETVSTTYSEGSHGKHARHAPSPAGIQSNFPLRMGENQK 897

Phrynocephalus_forsythii_Acr VMKEGRPAPLRSAPQFRFPPWEETVSAAYSEGSHGKHARHAPSPAGIQSSFPFRMGKNHK 889

Phrynocephalus_guinanensis_Acr VMKEGRPAPLGSAPQFRFPPWEETVSTAYSEGSHGKHARHAPSPAGIQSNFPFRMGKNHK 884

Phrynocephalus_putjatai_Acr VMKEGRPAPLGSAPQFRFPPWEETVSTAYSEGSHGKHARHAPSPAGIQSNFPFRMGKNHK 888

Phrynocephalus_vlangalii_Acr VMKEGRPAPLRSAPQFRFPPWEETVSTAYSEGSHGKHARHAPSPAGIQSNFPFRMGKNHK 889

Phrynocephalus_versicolor_Acr VMKEGRPAPLRSAPPFRFPPWEETVRTAYSEGSHGKHARHAPSPAGIQSNFPFRM----- 884

Intellagama_lesueurii_Acr VTKEGIPAPLRSAPQFRPATWEEKISTAYSEGSHGKHARHAPSPAGIQSNYPFMVGKKQK 897

Pogona_vitticeps_Acr VTKEGLPAPLRSAPQFRLAPWEEKISAGYSEGSHGKHARHAPSPAGIQSNYPFMVGKKQK 898

Bradypodion_pumilum_Acr VMKEGLPAPLRSAPQFRLALWGQTESSAYPEGSHGKHARHAPFPAGIQSSQTLLMGKNQK 880

Bradypodion_ventrale_Acr VMKEGLPAPLRSAPQFRLALWGQTESSAYPEGSHGKHARHAPFPAGIQSSQTLLMGKNQK 878

Chamaeleo_calyptratus_ENAM_Acr VTKEGLPAPLRSAPQFRLALWGQTESSAYPEGSHGKHARHAPFPDGIRSSQTLLMGKNQK 860

Chamaeleo_dilepis_ENAM_Acr VTQEGLPAPLRSAPQFRLALWGQTESSAYPEGSHGKHARHAPFPDGIRSSQTLLMGKNQK 858

Chamaeleo_gracilis_ENAM_Acr VTKEGLPAPLRSAPQFRLALWGQTESSAYPEGSHGKHARHAPFPDGIRSSQTLLMDKNQK 861

Chamaeleo_laevigatus_ENAM_Acr VTKEGLPAPLRSAPQFRLALWGQTESSAYPEGSHGKHARHAPFPDGIRSSQTLLVGKNQE 855

Furcifer_pardalis_Acr MTKEGLPAPLRSAPQFRLALWGQTESSAYPEGSHGKHARHAPFPAGIQSSQTLLMGKNQK 863

Trioceros_affinis_ENAM_Acr VTKEGLPTPLRSAPEFRFALWGQTESSAYPEGSHGKHARHAPFPAGIQSSQTLLMGKNQK 863

Trioceros_balebicornutus_ENAM_Acr VTKEGLPTPLRSAPESRFALWGQTESYAYPEGSHGKHARHAPFPAGIQPSQTLLMGKNQK 863

Trioceros_harennae_ENAM_Acr VTKEGLPTPLRSAPESRFALWGQTESSAYPEGSHGKHARHAPFPAGIQSSQTLLMGKNQK 863

Anolis_apletophallus_Pleu VRRENRLAPLRSASQFRPVLWEPKESATYLDDSHANYVRHAHSPAGIQGNNPLKNEGRQR 893

Anolis_tropidonotus_ENAM_Pleur VRRENRLAPLRSASQFRHALWEPKESSTYPDDSYANYVRHAHSPAGIQGNNPLKNEGRQR 897

Anolis_sagrei_ordinatus_Pleu VRRENRLAPLRSAPQFRHVLWEPKESSTYPDDSHSNYVRYAHSPAGIQGNNPLKNGGRER 897

Anolis_carolinensis_Pleu MPRENRLAPLRSAPQFRHALWEAKESSTYPDGSHANYVRHAHSPAGIQANNPLKNGGREQ 898

Ctenosaura_bakeri_Pleu VPRENRLAPLRNAPQFRLASWEQKESPIYSEGSHAKYARHAPSPAGIQANHPLKNGEKEQ 908

Cyclura_pinguis_Pleu VPRENRLAPLRNAPQFKLASWEQKESPIYSEGSHAKYARHAPYPAGIQANHPLKNGEKEQ 908

Iguana_delicatissima_Pleu VPRENRLAPLRNAPQFRLASWEQKESPIYSEGSHAKYARHAPSPAGIQANHPLKNGKKEQ 907

Gambelia_wislizenii_Pleu VPRENKLAPLRSAPQFRLASWEQKESSTYPEGSHAKYVRHAPSPAGIQANRPLTNRKKEQ 908

Laemanctus_serratus_ENAM_Pleur VPRENRLAPLRSAPAFRLASWEQKESSTYPESSHAKYVRHAPSPAGIQANHPLKNGKKER 909

Phrynosoma_blainvillii_Pleu VPRENKLAPLKNAPQFRLASWEQKESSIYPEGTHAKYVRHAPSPVGIKSNQPLRNGKNEQ 913

Phrynosoma_platyrhinos_Pleu VPRENK---LKNAPQFRLASWGQKESSIYPEGSHAKYVRHAPSPVGIKSNQPLRNGKNEQ 913

Sceloporus_chrysostictus_ENAM_Pleur VPRENKLAPLRSAPQFRLASWEQKESSIYPEGGHAKYVRHVPSPAGIKSNHPLRNEIK-G 909

Urosaurus_nigricaudus_Pleu VPRENKLAPLRSAPQFRLASWEQKESSIYPEGSHAKYVRDAPSPAGIKSNHPLRNEIKEG 916

Sceloporus_occidentalis_Pleu VPRENKLAPLRSAPQFRLASWEQKESSIYPEGSHAKYVRHVPSPAGIKSNHPLRNGIKEG 916

Sceloporus_tristichus_Pleu VPRENKLAPLRSAPQFRLASWEQKESSIYPEGSHAKYVRHVPSPAGIKSNHPLRNGIKEG 916

Sceloporus_undulatus_Pleu VPRENKLAPLRSAPQFRLASWEQKESSIYPEGSHAKYVRHVPSPAGIKSNHPLRNGIKEG 916

.*. * : * . * :. :.::.* . * **: . .:

Acanthocercus_cyanogaster_ENAM_Acr ENLGTFPEGVMALQKDLPCSKSKLSQDHHTHGTNCDTGLPSSKNIRDYGNSLRGDRH-GI 944

Acanthocercus_minutus_ENAM_Acr ENLGTFPEGVMGLQKDLPCSKSKLSQDHHTHGANCDTGLPSSKNIRDYGNSLRGDRH-SI 939

Xenagama_zonura_ENAM_Acr ENLGTFPEGVMGLQKDLPCSKSKLSQDHHTHGANCDTGLPSSKNIRDYGNSLRGDRH-SI 939

Agama_doriae_ENAM_Acr ENLGTFPEGVMGLQKNLPCSKSKLSQD-HVHETNCKIGLPSSQNTHGYGNSLRGDTH-SI 948

Laudakia_wui_Acr GNLGTFPEGVVGLQKNPPCSKSKLSQD-HVHETNCETGLPPSKNTHDYGNSLRGDRH-SI 955

Phrynocephalus_forsythii_Acr EHLGAFPEGVVGLQKNLPCSKSKLSQD-HVHETNCDTGLPPSQNTHDYGNSLRGDRHSSI 948

Phrynocephalus_guinanensis_Acr EHLGAFPEGVMGQQKNLPCSKSKLSQD-HVHETNCDTGLPPSQKTHDYGNSLRGDRHSSI 943

Phrynocephalus_putjatai_Acr EHLGAFPEGVMGQQKNLPCSKSKLSQD-HVHETNCDTGLPPSQKTHDYGNSLRGDRHSSI 947

Phrynocephalus_vlangalii_Acr EHLGAFPEGVMGLQKNLPCSKSKLSQD-HVHETNCDTGLPPSQNTHDYGNSLRGDRHSSI 948

Phrynocephalus_versicolor_Acr EHLGTFPEGVMGLQKNLPCSKSKLSQD-HVHETNCETGLPPSQNTHDYGNSLRGDRHSSI 943

Intellagama_lesueurii_Acr ENLDTFTEEATGVQKNLPCSKSKLSQD-HVHETNCETGLPPSKNTHDYGNSLRGDRH-SI 955

Pogona_vitticeps_Acr ENLDTFTEEAAGAQKNLPCSKSKLSQD-HTHETNCETGLPPSENTHDYGNSLRGDRH-SI 956

Bradypodion_pumilum_Acr ESLGTFIEEVTGLQKKLPC--SKLSQD-DLHETNCDTGLPALKSTHNYGNNLRGDRH-SI 936

Bradypodion_ventrale_Acr ESLGTFIEEVTGLQKKLPCSKSKLSQD-DLHETNCETGLPALKSTHNYGTNLRGDRH-SI 936

Chamaeleo_calyptratus_ENAM_Acr ESLGTFIEEVRGLEKKLPCSKSKLSQD-DLHETNCETGLPALKSTHNYGNNLRGDRQ-SI 918

Chamaeleo_dilepis_ENAM_Acr ESLGTLIEEVTGLEKKLPCSKSKLSQD-DLRETNCETGLPALKSTHNYGNNLRGDRH-SI 916

Chamaeleo_gracilis_ENAM_Acr ESLGTLIEEVTGLEKKLPCSKSKLSQD-DLRETNCETGLSALKSMHNYGSNLRGDRH-SV 919

Chamaeleo_laevigatus_ENAM_Acr ESLGALI-EVTGLEKKLPCSKSKLSQD-DLRETNCETGLPALKSMHNHGNNFRGDRY-SI 912

Furcifer_pardalis_Acr ESLGTFIEEVKGLQKKLPCSKSKLSQD-GLHETNCETGLPALKSMHNYGNNLRGDRH-SI 921

Trioceros_affinis_ENAM_Acr ESLGTFIEEVTGLQKKLPCSKSKLSQD-DLHETNCETGLPALKRLHNYGNSLRGDRH-SI 921

Trioceros_balebicornutus_ENAM_Acr ESLGTFIEEVTGLQKKLPCSKSKLSQD-DLHETNCETGLPALKRLHNYGNSLRGDRH-SI 921

Trioceros_harennae_ENAM_Acr ESLGTFIEEITGLQKKLPCSKSKLSQD-DLHETNCETGLPALKRLHNYGNSLRGDRH-SI 921

Anolis_apletophallus_Pleu EELGAFRMENAGSEKSPPCSNSHLSQD-SKQEANFQRGSFQFRNMPCHGSNIRGDRH-NP 951

Anolis_tropidonotus_ENAM_Pleur EELGAFRMENAGSEKSPPCSNSHLSQD-SKQEANFQRGSFQFRNMHCHGSNMRGDRH-NP 955

Anolis_sagrei_ordinatus_Pleu EELGTFRVENAGSENSPPCSNSHLSQD-SKQEANFQRGLFQFRNMPCHGSNLRGDRH-NP 955

Anolis_carolinensis_Pleu EELGAFRVENAGSEKSLPCSNSHLSQD-SKQEANLQRGLFKFRNMPCHGSNIRGDRH-NP 956

Ctenosaura_bakeri_Pleu EELGAFRVENADFEKSPPCSSAPLSKD-SKQEANFQSGLYQLRNMPCHGGSIRGDRH-NI 966

Cyclura_pinguis_Pleu EELGAFRVENAGFEKSPPCSNAPLSKD-SKQEANFQSGLHQLRNLPCHGGSIRGDRH-NI 966

Iguana_delicatissima_Pleu EELGAFRVENAGFEKSPPCSNAPLSKD-SKQEANFQSGLHQLRNMPCHGGSIRGDRH-NI 965

Gambelia_wislizenii_Pleu EELGIFTVEHAGIEKSPPCSNSQLSQD-SKQEANFQNGLFQLRNIPCHGSSIRGDRH-HF 966

Laemanctus_serratus_ENAM_Pleur EEPGLFTMENAGLEKSPPCPNAQLSQD-DKQEANFQNGLFQLRNMPCHGSSIRGDRH-NI 967

Phrynosoma_blainvillii_Pleu EKLGAFRVENVDLEKSPPCSTSQLSQD-SKQEANFQSGLFQLRNMPCHGSTIRGDRH-DI 971

Phrynosoma_platyrhinos_Pleu EKLGAFRVENVDLEKSPPCSTSQLSQD-SKQEANFQSGLFQLRNMPFHGSTIRGDRH-DI 971

Sceloporus_chrysostictus_ENAM_Pleur EELSASRVENVDLEKSPPCSTSQLSQD-NKLEANFENGLREFRNMPCHGSTVRGDRH-NI 967

Urosaurus_nigricaudus_Pleu EQ-GAFRVENADLEKSLPCSTPQLSQD-SKVEANFQNGLRELRNMPCHGSTVRGDRH-NI 973

Sceloporus_occidentalis_Pleu EELGAFRVENSDLEKSPPCATSQLSQD-SKLEANFQNGHRELGNMPCHGSTIRGDRH-NI 974

Sceloporus_tristichus_Pleu EELGAFRVENADLEKSPPCATSQLSQD-SKLEANFQNGHRELGNMPCHGSTIRGDRH-NI 974

Sceloporus_undulatus_Pleu EELGAFRVENADLEKSPPCATSQLSQD-SKLEANFQNGHRELGNMPCHGSTIRGDRH-NI 974

. ::. ** . **:* :* . * :* ..***

Acanthocercus_cyanogaster_ENAM_Acr PAGIMGERESRRELEEAALRLTPEKSPQPQRLRSEALADENGRKEQHPALEIKRIPCFAS 1004

Acanthocercus_minutus_ENAM_Acr PAGIMGERESRRDLEEAALRLTAEKSPQPQRLRSEALADENGRKEQHPALEIKRIPCFAS 999

Xenagama_zonura_ENAM_Acr PAGIMGEKESRRELEEAALRLSPEKSPQPQRLRSEALADENGRKEQHPALEIKRIPCFAS 999

Agama_doriae_ENAM_Acr PAGIVGERESRREMEEAALKLTAEKSPQPQRMRSESLADENGRKEQHP--KIKRIPCFAS 1006

Laudakia_wui_Acr PAEIMGERESRRELEEAALKLVPEKSPEPQRIRSEALGGENGRKEQHAALEIKRIPCFAS 1015

Phrynocephalus_forsythii_Acr PAGIMEERESRRELEEAAVKLIPEKSPEPQRIRSEAIAGENGRKEQHAALEIKRIPCFAT 1008

Phrynocephalus_guinanensis_Acr PAGIMEERESRRELEEAAVKLIPEKSTEPQRIRSKALAGENGRKEQHAALEIKRIPCFAS 1003

Phrynocephalus_putjatai_Acr PAGIMEERESRRELEEAAVKLIPEKSTEPQRIRSKALAGENGRKEQHAALEIKRIPCFAS 1007

Phrynocephalus_vlangalii_Acr PAGIMEERESRRELEEAAVKLIPEKSPEPQRIRSEALAGENGRKEQHAALEIKRIPCFAS 1008

Phrynocephalus_versicolor_Acr PAGIMEERESRREQEEAAVKLIPEKSPEPQRIRSEALAGENGRKEQHATLEIKRIPCFAS 1003

Intellagama_lesueurii_Acr PDAIMGESESRREFRQAALKLVPEKSPEPQRIRSEALASEDGRKEQHAALEIKRIPCFAS 1015

Pogona_vitticeps_Acr PAAIMGESESRREFRQAAVKLVPEKSPEPQRIRSQALASEDGRKEQHAALEIKRIPCFAS 1016

Bradypodion_pumilum_Acr PVQMMEERESRWELGQADLKLVPETFPGLQRIRSETLGNEDGRKEQHAAIEVKRIPCFAS 996

Bradypodion_ventrale_Acr PVQMMEERESRWELGQADLKLVPETFPGLQRIRSETLGNEDGRKEQHAAIEVKRIPCFAS 996

Chamaeleo_calyptratus_ENAM_Acr PVQMMEERESRRELGQADLKLVPETFPGPQMIRSETFGNEDGRKEQHTAVEVKRIPCFAS 978

Chamaeleo_dilepis_ENAM_Acr PVQMMEERESRRELGQADLKLVPETFPGPQRIRSETFGNEEGRKEQHAAVEVKRIPCFAS 976

Chamaeleo_gracilis_ENAM_Acr PVQMMEERESRRELGQADLKLVPETFPGPQRIRSETLGNEDGRKEQHAAVEAKRIPCFAS 979

Chamaeleo_laevigatus_ENAM_Acr PVQMMEERESRRELGQADLKLVPETFPGPQRIRSETFGNEDVRKEQHAAVEVKRIPCFAN 972

Furcifer_pardalis_Acr PVQMMEERESTRELGQADLKLVPETFPGPQRIRSETFGNEDGRKEQHAAVEVKRIPCFAS 981

Trioceros_affinis_ENAM_Acr PVQMMEEREFRRELGQADLKLVPETFPGPQRIRSETFGNEDGRKEQHAAVEVKRIPCFAS 981

Trioceros_balebicornutus_ENAM_Acr PVQMMEERESRRELGQADLKLVPETFPGPQRIRSETFGNEDGRKEQHAAVEVKRIPCFAS 981

Trioceros_harennae_ENAM_Acr PVQMMEESESRRELGQADLKLVPETFPGPQRIRSETFGNEDGRKEQHAAVEVKRIPCFAS 981

Anolis_apletophallus_Pleu LAHLVGTGQSKREFGKATLNFLPENFPQSHGIQSEALVSADDGKE-YPSLGTKRIPCFES 1010

Anolis_tropidonotus_ENAM_Pleur LAHLVGTGQSKREFGKSTLNYLPEKSPQSHGIQSEALVSADDGKE-YASLGTKRIPCFGS 1014

Anolis_sagrei_ordinatus_Pleu LAYLVGTGQSKREFGKATLNFLPEKIPQSHGIQSEALVSEDDGKE-YASLGTKRIPCFGS 1014

Anolis_carolinensis_Pleu LAHLVGTGQS---FGRGTLNFLPEQFPQPHGIQSEALVSEDDGKE-YAALGAKRIPCFGS 1012

Ctenosaura_bakeri_Pleu LAHLVGTSQSKREFEKTASKLLPERFPQPQEIQSEALLSEDDRKEQHVALGAKRIPCFGS 1026

Cyclura_pinguis_Pleu LAHLVGTSQSKREFEKTASKFLPERLPQPQGIQSEALLSEDDRKEQHVALGAKRIPCFGS 1026

Iguana_delicatissima_Pleu LAHLVGTSQSKREFEKTGSKLLPESLPQPQGIQSEALLSEDDRKEQQVALGAKRIPCFGS 1025

Gambelia_wislizenii_Pleu LAHLVGTSQSKKEFEKAALKLPPEKFPQPQGIQSEALVSEDDRKKQHPALGAKRIPCFGR 1026

Laemanctus_serratus_ENAM_Pleur LAHLVGTSQSNREFEKSALKHLPQKLPQPQAIQSEVLVSEVDRKGQHAARAAKKIPCFGN 1027

Phrynosoma_blainvillii_Pleu LAHLEGTSQSKRELERVPLKLLPEKFPQSQVIESEALVSEDNRKEQHATLGAKRIPCFGS 1031

Phrynosoma_platyrhinos_Pleu LAHLEGTSQSKRELERVPLKLFPEKFPQPQVIQSEALVSEDNRKEQHAALGAKRIPCFGS 1031

Sceloporus_chrysostictus_ENAM_Pleur LAHLVGTSQSRRQSEKAPLKLLFEKFPRPQGIQSEALVSEDNRKEQHAALGAKRIPCFGS 1027

Urosaurus_nigricaudus_Pleu LARLVGTGQSKRQSEKASLKLLFEKLPRPQGIQSEALISEDNRKEQHVALEAKRIPCFGS 1033

Sceloporus_occidentalis_Pleu LAHLVGTSQSKRQSEKAPLKLLFEKFPRPQGIQSEALVSEDNRKEQHAALGAKRIPCFGN 1034

Sceloporus_tristichus_Pleu LAHLVGTSQSKRQSEKAPLKLLFEKFPQPQGIQSEALVSEDNRKEQHTALGAKRIPCFGN 1034

Sceloporus_undulatus_Pleu LAHLVGTSQSKRQSEKASLKLLFEKFPQPQGIQSEALVSEDNRKEQHAALGAKRIPCFGN 1034

: : . . : . : :.*: : . * *:****

Acanthocercus_cyanogaster_ENAM_Acr WLKQYLSSTGAPSGDPQHDLLHGEAPFPTSRPNILPPESKPISETNPSHDDVEGKHLTFS 1064

Acanthocercus_minutus_ENAM_Acr WLKQYLSSTGAPSGDQQHKLLHGEAPFPTSRPNILPPESKPISSTNPSHDDVEGKHLTFS 1059

Xenagama_zonura_ENAM_Acr WLKQYLSSTGAPSGDQQHKLLHGEAPFPTSRPNILPPESKPISSTNPSHDDVEGKHLTFS 1059

Agama_doriae_ENAM_Acr WLKQYLSSTGAPSGDQQHNLLHGEAPFPTSKPNSL-PESKPISSTNPSHDDVEGKHLTFS 1065

Laudakia_wui_Acr WLKQYLSSTGAPSGDQQHNLLHGEAPFPTSRPNSLPPESKPISSTNPSHDDVEGKHLTFS 1075

Phrynocephalus_forsythii_Acr WIKQYLSSTGAPSGDQQHNLLHGEAPFPTSRPNSLPPESKPISSTNPSHDDIEGKHLTFS 1068

Phrynocephalus_guinanensis_Acr WLKQYLSSTGAPSGDQQHNLLHGEAPFPTSRPNSLPPESKPISSTNPSHDDVEGKHLTFS 1063

Phrynocephalus_putjatai_Acr WLKQYLSSTGAPSGDQQHNLLHGEAPFPTSRPNSLPPESKPISSTNPSHDDVEGKHLTFS 1067

Phrynocephalus_vlangalii_Acr WLKQYLSSTGAPSGDQQHNLLHGEAPFPTSRPNSLPPESKPISSTNPSHDDIEGKHLTFS 1068

Phrynocephalus_versicolor_Acr WLKQYLSSTGAPSGDQQHNLLHGEAPFPTSRPNSLPPESKPISSTNPSHGDVEGKHLTFS 1063

Intellagama_lesueurii_Acr WLKQYLSSTGAPSGDQQHNLFHGETPVPTSRPNNMPPESKPISSTNPSHDDREGKHLKFG 1075

Pogona_vitticeps_Acr WLKQYLSSTGAPSGDQQHNLFHGETPVPTSRPNDMPPESKPISSTNPSHDDREGKHLKFG 1076

Bradypodion_pumilum_Acr WLKHYLSSTGAPSGDQKHNLFHGETPSPTSRPNNRPPESKPIASTNPSYDNVEEKHLTFG 1056

Bradypodion_ventrale_Acr WLKHYLSSTGAPSGDQKHNLFHGETPSPTSRPNNRPPESKPIASTNPSYDNVEEKHLKFG 1056

Chamaeleo_calyptratus_ENAM_Acr WLKQYLSSTGAPSGDQKHNLFHGETPSPTNRPNSRPPESKPIASTNPSYDNVEEKHLKFS 1038

Chamaeleo_dilepis_ENAM_Acr WLKQYLSSTGAPSGDQKHNLFHGETPSPTSRPDSRPPESKPIASTHPSYDNVEEKHLKFG 1036

Chamaeleo_gracilis_ENAM_Acr WLKQYLSSTGAPSGDQKHNLFHGETPSPTSRPNSRPPESKPIASTNPSYDNVEEKHLKFS 1039

Chamaeleo_laevigatus_ENAM_Acr WLKQYLSSTGAPSGDQKHNIFHGETPSPTSRPNSRPPESKPIASTNPSYDNVEEKHLKFG 1032

Furcifer_pardalis_Acr WLKQYLSSTGAPSGDQKHNLFHGETPSPTSRPNSRPPESKPIASTNPSYDNVEEKHLKFS 1041

Trioceros_affinis_ENAM_Acr WLKQYLSSTGAPSGDQKHTLFHGETPSPTSRPNSRPPESKPIASTNPSYDHVEEKHLKFG 1041

Trioceros_balebicornutus_ENAM_Acr WLKQYLSSTGAPSGDQKHNLFHGETPSPTSRPNSRPPESKPIASTNPSYDNVEEKHLKFG 1041

Trioceros_harennae_ENAM_Acr WLKQYLSSTGAPSGDQKHNLFHGETPSPTSRPNSRPPESKPIASTNPSYDNVEEKHLKFG 1041

Anolis_apletophallus_Pleu WLKQYLSNTEAPAGDQQRDSFYGENQVPTERPNSVPPKPEPITSSFLSNG-VEEKPLKIN 1069

Anolis_tropidonotus_ENAM_Pleur WLKQYLSNTEAPSGDQQRDSFYGENPIPTERPNSAPPKPEPISSSFLSNG-VEEKPLKIN 1073

Anolis_sagrei_ordinatus_Pleu WLKQYLSNTEAPSGDQQHDPFYGENLVPTGKPNSVPPKPEPISSSFLSNG-VEEKPLKIN 1073

Anolis_carolinensis_Pleu WLKQYLSNTEVPSDDQQRDPFYGENPVPTERPNSIPPKPEPISSSFLSNG-VEEKPLKIN 1071

Ctenosaura_bakeri_Pleu WLKQYLSSTRAPSGDQQQDPFYAENPVPTGRPNSMLQEPEPISSTYPSQD-KEENPLKIN 1085

Cyclura_pinguis_Pleu WLKQYLSSTRAPSGDQQQDPFYVENPVPTGRPNSMPQEPEPISSSYPSQD-GEEKPLKIN 1085

Iguana_delicatissima_Pleu WLKQYLSSTRAPSGDQQQDPFYAENPVPTGRPNSMPQEPEPISSTYPSQD-GEGKPLKIN 1084

Gambelia_wislizenii_Pleu WLKQYLSSTGAPSGDQQHDPFYGENPIPTGRPNSMPPEPEPISSTYPSPD-IEEKPLKIN 1085

Laemanctus_serratus_ENAM_Pleur WLKQYLSSTGAVPGDQPDDPFYGEGPVPTGRTSSMPPDPEPISSTYPSQG-VEEKPLKIN 1086

Phrynosoma_blainvillii_Pleu WLKQYLSSTVAPSGDQQPDPFYGENLFPTERPNSMPPKPEPISSTYPSYD-RGEKPVKIN 1090

Phrynosoma_platyrhinos_Pleu WLKQYLSSTGAPSGDQQPDPFYGENLFPTERPNSMSPDPEPISSTYPSYD-RGEKPVKIN 1090

Sceloporus_chrysostictus_ENAM_Pleur WLKQYLSSTGAPSGDQLPDSFYGESLFPTERPNSMPPEPEPISSTYPSHD-TGEKPVKIN 1086

Urosaurus_nigricaudus_Pleu WLKQYLFSTGAPSGDQQPDSFYGESLFPTERPNSRPPEPEPISSTYPSHD-TGEKPVKIN 1092

Sceloporus_occidentalis_Pleu WLKQYLSSTGAPSGDRQPNSFYGESLFPTERPNSMPPEPEPISSTYPSHD-TGEKPVKIN 1093

Sceloporus_tristichus_Pleu WLKQYLSSTGAPSGDRQPVSYYGESLFPTERPNSMPPEPEPISSTYPSHD-AGEKPVKIN 1093

Sceloporus_undulatus_Pleu WLKQYLSSTGAPSGDRQPNSFYGESLFPTKRPNSMPPEPEPISSTYPSHD-AGEKPVKIN 1093

*:*:** .* . ..* : * ** :.. ..:**:.: * . : :.:.

Acanthocercus_cyanogaster_ENAM_Acr APGEDWVERPDEATQDCLLLQNE- 1087

Acanthocercus_minutus_ENAM_Acr TPGEDWVERPDEATQDCLLLQNE- 1082

Xenagama_zonura_ENAM_Acr TPGEDWVERPDEATQDCLLLQNE- 1082

Agama_doriae_ENAM_Acr APGEDWVERPDEATPDCLLLQNE- 1088

Laudakia_wui_Acr APGEEWVERPDEATPDCLLLQNE- 1098

Phrynocephalus_forsythii_Acr ALGEEWVERPDEATPDCLLLQNQM 1092

Phrynocephalus_guinanensis_Acr TLGEEWVERPDEATPDCLLLQNK- 1086

Phrynocephalus_putjatai_Acr TLGEEWVERPDEATPDCLLLQNK- 1090

Phrynocephalus_vlangalii_Acr ALGEEWVERPDEATPDCLLLQNK- 1091

Phrynocephalus_versicolor_Acr ALGEEWVERPDEATPDCLLLQNK- 1086

Intellagama_lesueurii_Acr SPGEEWVEQPNETTPDCLLLQKK- 1098

Pogona_vitticeps_Acr SPGEEWVERPNETTPDCLLLQKK- 1099

Bradypodion_pumilum_Acr SPGEEWVEQPNETTPDCLLLQNK- 1079

Bradypodion_ventrale_Acr SPGEEWVEQPNETTPDCLLLQNK- 1079

Chamaeleo_calyptratus_ENAM_Acr SPGEEWVEQPNETTPDCLLLQKI- 1061

Chamaeleo_dilepis_ENAM_Acr SPGEEWVEQPNETTPDCLLLQKK- 1059

Chamaeleo_gracilis_ENAM_Acr SPGEEWVEQPNETTPDCLLLPKK- 1062

Chamaeleo_laevigatus_ENAM_Acr SPGEEWVEQPNETTPDCLLLQK-- 1054

Furcifer_pardalis_Acr SPGEEWVEQPNETTPDCLLLQNK- 1064

Trioceros_affinis_ENAM_Acr SPGEEWVEQPNETTPDCLLLQNK- 1064

Trioceros_balebicornutus_ENAM_Acr SPGEEWVEQPNETTPDCLLL---- 1061

Trioceros_harennae_ENAM_Acr SPGEEWVEQPNETTPDCLLLQNK- 1064

Anolis_apletophallus_Pleu APGEEWAEQATQSTPDCLLLQKK- 1092

Anolis_tropidonotus_ENAM_Pleur TPGEEWAEEATQSTPDCLVLQKK- 1096

Anolis_sagrei_ordinatus_Pleu APGEEWAEQATQSTPDCLLFQKK- 1096

Anolis_carolinensis_Pleu APEEEWAELATQSTPDCLLLQKK- 1094

Ctenosaura_bakeri_Pleu SPGEKWDEPAAQSTPDCLLLQNK- 1108

Cyclura_pinguis_Pleu SPGEKWDEPAAQSTPDCLLLQNK- 1108

Iguana_delicatissima_Pleu SPGEKWDEPAAQSTPDCLLLQNK- 1107

Gambelia_wislizenii_Pleu SPGDEWAEQAAQSTPDCLLLQKK- 1108

Laemanctus_serratus_ENAM_Pleur SPVEEWAEQAAQSTPDCLLLQNK- 1109

Phrynosoma_blainvillii_Pleu SPGEEWAGQVAQSTPDCLLLQNK- 1113

Phrynosoma_platyrhinos_Pleu SPGEEWAGQVAQSTPDCLLLQDK- 1113

Sceloporus_chrysostictus_ENAM_Pleur SQGEKWAGQAAQSTPDCLLLQNK- 1109

Urosaurus_nigricaudus_Pleu SPGEEWAGQAAQSTPDCLLLQNK- 1115

Sceloporus_occidentalis_Pleu SPGEEWAGQAPQSTPDCLLLQNK- 1116

Sceloporus_tristichus_Pleu SPGEEWAGQAAQSTPDCLLLQNK- 1116

Sceloporus_undulatus_Pleu SPGEEWAGQAAQSTPDCLLLQNK- 1116

: :.* ::* ***::

**MMP20-AA-MAFFT-Iguania+Mammal – DIVERGE + PCOC ≥ 0.9**

CLUSTAL W (1.8) multiple sequence alignment (ALTER 1.3.3)

Acanthocercus_cyanogaster_MMP20_Acr MEHLPLYFPPFLLVLSLKYATPAPAVFALPHTASWRDLYLAQEYLDKYYTMKSGPQVGEM 60

Acanthocercus_minutus_MMP20_Acr MEHLPLYFPPFLLVLSLKYATPAPALFALPHTASWRDLYLAQEYLDKYYTPKSAPQVGEM 60

Xenagama_zonura_MMP20_Acr MEHLPLYFPPFLLVLSLKYATPAPALFALPHTASWRDLYLAQEYLDKYYTPKSGPQVGEM 60

Agama_doriae_MMP20_Acr MEHLPLYFPPFLLVLSLKYLTAAPALFALPHTSSWRDLYLAQEYLDKYYTPNSGPQVGEM 60

Laudakia_wui_Acr MEHLPLFFPPFLLVLSLKYSAPAPALFALPHTSNWRDFYLAQEYLDKYYTPKRGPQVGEM 60

Phrynocephalus_forsythii_Acr MEHLPLYFPPFLLVLSLKYSAPAPALFALPHTSSWRDLYLAQEYLDKYYTPKRGPQVGEM 60

Phrynocephalus_guinanensis_Acr MEHLPLYFPPFLLVLSLKYSAPAPALFALPHTSSWRDLYLAQEYLDKYYTPKRGPQVGEM 60

Phrynocephalus_vlangalii_Acr MEHLPLYFPPFLLVLSLKYSAPAPALFALPHTSSWRDLYLAQEYLDKYYTPKRGPQVGEM 60

Phrynocephalus_putjatai_Acr MEHLPLYFPPFLLVLSLKYSAPAPALFALPHTSSWRDLYLAQEYLDKYYTAKRGAQVGEM 60

Phrynocephalus_versicolor_Acr MEHLPLYFPPFLLVLSLKYSAPAPALFALPHTSSWRDLYLAQEYLDKYYTPS-GPQVGEM 59

Intellagama_lesueurii_Acr MEHLPLFCPPLLLVLSLKYSASAPALFALPHTSNWRDLYLAQEYLDKYYTPKGGPQIGEM 60

Pogona_vitticeps_Acr MEHLPLFCPPVLLVLSLKYSASAPALFALPHTSNWRDLYLAQEYLDKYYTPKGGPQIGEM 60

Bradypodion_pumilum_Acr MEHFSLLCPFLLLVLSLKCSAAAPALFASTHASNWRDLYLAQEYLDKYYIPKGVPQLGEM 60

Bradypodion_ventrale_Acr MEHFSLLCPFLLLVLSLKCSAAAPALFASTHASNWRDLYLAQEYLDKYYTPKGVPQLGEM 60

Chamaeleo_calyptratus_Acr MEHFSLFCPFLLLVLSLKCSAAAPALFASAHASNWRDLYLAQEYLDKYYTPKGVPQLGEM 60

Chamaeleo_dilepis_MMP20_Acr MEHFSLFCPFLLLVLSLKCSAAAPALFASTHASNWRDLYLAQEYLDKYYTPKGVPQLGEM 60

Trioceros_balebicornutus_MMP20_Acr MEHFSLFCPFLLLVLSLKCSAAAPALFASTHASNWRDLYLAQEYLDKYYTPKGVPQLGEM 60

Trioceros_harennae_MMP20_Acr MEHFSLFCPFLLLVLSLKCSAAAPALFASTHASNWRDLYLAQEYLDKYYTPKGIPQLGEM 60

Chamaeleo_gracilis_MMP20_Acr MEYFSLFCPFLLLVLSLKCSAAAPALFASTHASNWRDLYLAQEYLDKYYTPKGVPQLGEM 60

Furcifer_pardalis_Acr MEHFSLFWPFLLLVLSLKCSAAAPALFASTHASNWRDLYLAQEYLDKYYTPKGVPQLGEM 60

Chamaeleo_laevigatus_MMP20_Acr MARFSLFCPFLLLVLSLKYSAAAPALFASAHASNWRDLYLAQEYLDKYYTPKGVPQLGEM 60

Trioceros_affinis_MMP20_Acr MEHFSLFCPFLLLVLSLKCSAAAPALFASTHASNWRDLYLAQEYLDKYYTPKGVPQLGEM 60

Anolis_apletophallus_Pleu MDPFGLLWPPLLLLISLNYSAAAPALFALSQTTSWRDFYTAQEYLGKYYTPKGGHHVGEM 60

Anolis_carolinensis_Pleu MELFGLLCPPLLLLISLNYSAAAPALFALSQTSSWRDFYTAQEYLDKYYTPQEGHHVGEI 60

Anolis_sagrei_ordinatus_Pleu MELFGLLRPPLLLLISLNYSAAAPALFALSQTSSWRDFYTAQEYLDKYYTPKGGHLVGEI 60

Anolis_tropidonotus_MMP20_Pleur MELFCMFCPPLFLVLSLKCSAAAPALFALPRTSSWKDFYIAQEYLDKYYTPKGGHHVGEM 60

Ctenosaura_bakeri_MMP20_Pleur MELFGLLCPPLLLMLSLKYLAAAPALFALSQTSSWRDFYTAQEYLDKYYTPKGGHQVGEI 60

Sceloporus_occidentalis_Pleu MELLGLLCPPLLLMLSLKYVAAAPALFALSQTSSWRDFYTAQDYFDKYYTPKGGHHVGEI 60

Sceloporus_tristichus_Pleu MELLGLLCPPLLLMLSLKYLAAAPALFALSQTSSWRDFYTAQDYFDKYYTPKGGHHVGEI 60

Sceloporus_undulatus_Pleu MGLLGLLCPPLLLMLSLKYLAAAPALFALSQTSSWRDFYTAQDYFDKYYTPKGGHHVGEI 60

Urosaurus_nigricaudus_Pleu MERFGLLCPPLLLMISLKHLAAAPALFALSQTSSWKDFYTAQEYLDKYYTPKGGHHVGEM 60

Phrynosoma_blainvillii_Pleu MELFGLLCPPLLLMLSLKYLAAAPALFALSQTSSWRDFYIAQKYLDKYYTPKGGHHVGEI 60

Phrynosoma_platyrhinos_Pleu MELFSLLCPPLLLMLSLKYLAAAPALFALSKTSSWRDFYTAQEYLDKYYTTKGGHHVGEI 60

Ctenosaura_bakeri_Pleu MEFFALFCPPLLLVSSLKYSAAAPALFALSQTSSWKDFYTAQEYLDKYYTPKGRHHVGEI 60

Cyclura_pinguis_Pleu MEFFALLCPPLLLVSSLKYSAAAPALFALSQTSSWKDFYTAQEYLDKYYTPKGRHHVGEI 60

Iguana_delicatissima_Pleu MEFFALLWPPLLLVPSLKYSAAAPALFALSQTSSWKDFYTAQEYLDKYYTPKGRHHVGEI 60

Gambelia_wislizenii_Pleu MEFFVLLCPPLLLVLSLKYSAAAPALFALSQTSSWRDFYTAQEYLDKYYTPKGRHHVGEI 60

Laemanctus_serratus_MMP20_Pleur MELIGLLCPPLLLVLFLKYSAAAPALFALSQTSSWRDFYTAQEYLDKYYTPKGRHHVGEI 60

Agama_finchi_MMP20_Acr XXXLPLYFPPFLLVLSLKYSTTAPALFALPHTASWRDLYLAQEYLDKYYTPNSGLQVGEM 60

Canis_lupus_dingo_MMP20 MTVLPMCGLALLLGAALEFCTAAPSVSAAAPRTTQNKYHLAQAYLDKYYTSKAGPQVGEM 60

Vulpes_vulpes_MMP20 MTVLPTCGLALLLGAALEFCTAAPSVSAAAPRTTQNNYHLAQAYLDKYYTSKAGPQVGEM 60

Meles_meles_MMP20 MKVLPAPGLAVLLLTALELSATAPSLSAATPRTARNNYHLAQAYLDKYYTRKAGPQVGEM 60

Mustela_putorius_MMP20 MKVLPAPGLALLLLTALELSATAPSLSAATPGTSRNNYHLAQAYLDKYYTREAGPQVGEM 60

Ursus_arctos_MMP20 MKVLPASGLAVLLIAALKFSTAAPSLFAATPGTARNNFHLAQAYLDKYYTRKAGPQVGEM 60

Phacochoerus_africanus_MMP20 MKVLPASGLAVLLVTALKFSAAAPSLFAATPRTSRNNYHLAQAYLDKYYTKKGGHQVGEM 60

Erinaceus_europaeus_MMP20 MKVLPTSGFAVLLIMALKLSTAAPSLFAATPRTSRNNYHLAQEYLDRYYTKKEEYQIGEM 60

Phyllostomus_hastatus_MMP20 MKVLPASGLALLLITALNFSTAAPSLFAATPRTSKNNYRLAQAYLDKYYTRKGGHQVGEM 60

Homo_sapiens_MMP20 MKVLPASGLAVFLIMALKFSTAAPSLVAASPRTWRNNYRLAQAYLDKYYTNKEGHQIGEM 60

Saimiri_boliviensis_MMP20 MKVLPASGLAVLLIMALKFSTAAPSLVAASPRIWRNNYRLAQAYVDKYYTNKEGHQIGEM 60

Echinops_telfairi_MMP20 MKVLPASGLVVLLITALKCSTAAPSLFAATSRTSRNNYHLAQAYLEKFYPKKGEHQVGEM 60

Suncus_etruscus_MMP20 MKVLPAPSFAVLLFVTLKFSTAASSL---SSRTSRTNYHLAQAYLDKYYTKEGGPQIGEM 57

: .:* *: :.*.:: . . ** *. ::* . :**:

Acanthocercus_cyanogaster_MMP20_Acr TSGGGNSVMKKVQQMQAFFGLPITGKLDLTTMDVMRRPRCGVPDVANYRLFPGEPKWKKS 120

Acanthocercus_minutus_MMP20_Acr TSGGGNSVMKKVQQMQAFFGLPITGKLDLTTMDVMRRPRCGVPDVANYRLFPGEPKWKKS 120

Xenagama_zonura_MMP20_Acr TSGGGNSVMKKVQQMQAFFGLPITGKLDLTTMDVMRRPRCGVPDVANYRLFPGEPKWKKS 120

Agama_doriae_MMP20_Acr TSGGGNSVMKKVQQMQAFFGLPITGKLDLTTMEVMRRPRCGVPDVANYRLFPGEPKWKKN 120

Laudakia_wui_Acr TS-GGNSIIKKVQQMQAFFGLPITGKLDLTTMDVIRRPRCGVPDVANYRLFPGEPKWKKS 119

Phrynocephalus_forsythii_Acr TS-GGNSVIKMVRQMQAFFGLPITGKLDLTTMDVIRRPRCGVPDVANYRLFPGEPKWKKS 119

Phrynocephalus_guinanensis_Acr TS-GGNSVIKKVRQMQAFFGLPITGKLDLTTMDVIRRPRCGVPDVANYRLFPGEPKWKKS 119

Phrynocephalus_vlangalii_Acr TS-GGNSVIKKVRQMQAFFGLPITGKLDLTTMDVIRRPRCGVPDVANYRLFPGEPKWKKS 119

Phrynocephalus_putjatai_Acr TS-GGNSVIKKVRQMQAFFGLPITGKLDLTTMDVIRRPRCGVPDVANYRLFPGEPKWKKS 119

Phrynocephalus_versicolor_Acr TS-GGNSVIKKVRQMQAFFGLPITGKLDLTTMDVIRRPRCGVPDVANYRLFPGEPKWKKS 118

Intellagama_lesueurii_Acr TS-GGNSVIKKVQQMQAFFGLPVTGKLDFSTMDVIRRPRCGVPDVANYRLFPGEPKWKKS 119

Pogona_vitticeps_Acr TS-GGNSMIKKVQQMQAFFGLPVTGKLDFSTMDVIRRPRCGVPDVANYRLFPGEPKWKKS 119

Bradypodion_pumilum_Acr TS-GGNSMKKKVQQMQAFLGLPVTGKLDLSTMDVIRRPRCGVPDVANYRLFPGEPKWGKS 119

Bradypodion_ventrale_Acr TS-GGNSMIKKVQQMQAFLGLPVTGKLDLSTMDVIRRPRCGVPDVANYRLFPGEPKWGKS 119

Chamaeleo_calyptratus_Acr TS-GANSMIKKVQQMQAFLGLPVTGKLDLSTMDVIRRPRCGVPDVANYRLFPGEPKWKKS 119

Chamaeleo_dilepis_MMP20_Acr TS-GANSMIKKVQQMQAFLGLPVTGKLDLTTMDVIRRPRCGVPDVANYRLFPGEPKWKKS 119

Trioceros_balebicornutus_MMP20_Acr IS-GGNSMIKKVQQMQAFLGLPITGKLDLSTMEVIRRPRCGVPDVANYRLFPGEPKWKKS 119

Trioceros_harennae_MMP20_Acr TS-GGNSMIKKVQQMQAFLGLPITGKLDLSTMEVIRRPRCGVPDVANYRLFPGEPKWGKS 119

Chamaeleo_gracilis_MMP20_Acr TS-GANSMIKKVQQMQAFLGLPVTGKLDLTTMDVIRSPRCGVPDVANYRLFPGEPKWKKS 119

Furcifer_pardalis_Acr TS-GGNSMIKKVQQMQTFLGLPATGKLDLNTMDVIRRPRCGVPDVANYRLFPGEPKWEKS 119

Chamaeleo_laevigatus_MMP20_Acr TS-GANSMIKKVQQMQAFLGLPVTGKLDLSTMDAIRRPRCGVPDVANYRLFPGEPKWKKS 119

Trioceros_affinis_MMP20_Acr TS-GDNSMIKKVQQMQAFLGVPITGKLDLSTMEVIRRPRCGVPDVANYRLFPGEPKWKKN 119

Anolis_apletophallus_Pleu TS--GDSMTKKVQQMQAFFGLRVTGKLDYSTMDVIKRPRCGVPDIANYRLFPGEPKWKKN 118

Anolis_carolinensis_Pleu TS--GDSMTKKVQQMQAFFGLRVTGKLDYSTMDVIKRPRCGVPDIANYRLFPGEPKWKKN 118

Anolis_sagrei_ordinatus_Pleu TS--GDSMTKKVQQMQAFFGLRATGKLDYSTMDVIKRPRCGVPDIANYRLFPGEPKWKKN 118

Anolis_tropidonotus_MMP20_Pleur TS--GDSMTKKVQQMQAFFGLRVTGKLDYSTMDVIKRPRCGVPDIANYRLFPGEPKWKKN 118

Ctenosaura_bakeri_MMP20_Pleur IS-GGSSMTKKVQQMQAFFGLRVTGKLDYSTMDVIRKPRCGVPDIANYRLFPGEPKWKKS 119

Sceloporus_occidentalis_Pleu TS-GGNSMTKKVQQMQAFFGLQVTGKLDYSTMDVIRRPRCGVPDIANYRLFPGEPKWKKS 119

Sceloporus_tristichus_Pleu TS-GGNSMTKKVQQMQAFFGLQVTGKLDYSTMDVIRRPRCGVPDIANYRLFPGEPKWKKS 119

Sceloporus_undulatus_Pleu TS-GGNSMTKKVQQMQAFFGLQVTGKLDYSTMDVIRRPRCGVPDIANYRLFPGEPKWKKS 119

Urosaurus_nigricaudus_Pleu TS-GGNSMTKKVQQMQAFFGLQVTGKLDYSTMDVIRRPRCGVPDIANYRLFPGEPKWKKS 119

Phrynosoma_blainvillii_Pleu TS-GGNSMTKKVQQMQAFFGLRVTGKLDYSTMDVIRKPRCGVPDIANYRLFPGEPKWKKS 119

Phrynosoma_platyrhinos_Pleu TS-GGNSMTKKVQQMQAFFSLRVTGKLDYSTMDVIRRPRCGIPDIANYRLFPGEPKWKKS 119

Ctenosaura_bakeri_Pleu TS-GGNSLTKKVQQMQAFFGLRVTGKLDYSTMDIMKRPRCGVPDIANYRLFPGEPKWKKS 119

Cyclura_pinguis_Pleu TS-GGNSLTKKVQQMQAFFGLRVTGKLDYSTIDIMKRPRCGVPDIANYRLFPGEPKWKKS 119

Iguana_delicatissima_Pleu TS-GGNSLTKKVQQMQAFFGLRVTGKLDYSTMDIMKRPRCGVPDIANYRLFPGEPKWKKS 119

Gambelia_wislizenii_Pleu TS-GGNSMTKKVQQMQAFFGLQVTGKLDYSTMDVIRRPRCGVPDIANYRLFPGEPKWKKS 119

Laemanctus_serratus_MMP20_Pleur TS-GGSSMTKKVEQMQAFFGLRVTGKLDYSTMDVIKRPRCGVPDIANYRLFPGEPKWKKS 119

Agama_finchi_MMP20_Acr TSGSGNSVMKKVQQMQAFFGLPITGKLDLRTMEVMRRPRCGVPDVANYRLFPGEPKWKKS 120

Canis_lupus_dingo_MMP20 GAPGGRALIKKIKELQAFFGLRITGKLDRPTMDMIKRPRCGVPDVANYRLFPGEPKWKKN 120

Vulpes_vulpes_MMP20 GAPGGRALIKKIKELQAFFGLRITGKLDRPTMDMIKRPRCGVPDVANYRLFPGEPKWKKN 120

Meles_meles_MMP20 GAAGGRALVKKVKELQAFFGLRVTGKLDRPTMDVIKRPRCGVPDVANYRLFPGEPKWKKN 120

Mustela_putorius_MMP20 GAAGGRALVKKIKELQAFFGLRVTGKLDRPTMDVIKRPRCGVPDVANYRLFPGEPKWKKN 120

Ursus_arctos_MMP20 GAPGGRALVKKIKELQAFFGLRVTGKLDRRTMDVIKRPRCGVPDVANYRLFPGEPKWKKN 120

Phacochoerus_africanus_MMP20 VAKGGNSMVKKIKELQAFFGLRVTGKLDQTTMDVIKRPRCGVPDVANYRLFPGEPKWKKN 120

Erinaceus_europaeus_MMP20 VARGHNSMIKKIKELQAFFGLQITGKLDRTTMDVIKKPRCGVPDVANYRLFPGEPKWKKN 120

Phyllostomus_hastatus_MMP20 VARGSNSLVKKIKELQAFFGLQVTGKLDRSTMDVIKRPRCGVPDMANYRLFPGEPKWKKN 120

Homo_sapiens_MMP20 VARGSNSMIRKIKELQAFFGLQVTGKLDQTTMNVIKKPRCGVPDVANYRLFPGEPKWKKN 120

Saimiri_boliviensis_MMP20 VSRGGNSMVRKIKELQAFFGLQVTGKLDKTTMDVIKKPRCGVPDVANYRLFPGEPKWKKT 120

Echinops_telfairi_MMP20 VARGGNSMIRKIKELQAFFGLQVTGKLDQQTMNVIKKPRCGVPDVANYRLFPGEPKWKKN 120

Suncus_etruscus_MMP20 VARQRNTMVKKIKELQAFFGLQITGKLDKSTMDVIKRPRCGVPDVANYRLFPGEPKWKKN 117

: :: : :.::*:*:.: ***** *:: :: ****:**:************ *.

Acanthocercus_cyanogaster_MMP20_Acr TLTYRVKKYTSSLSHADVDKAVEMGLKAWSAAAPLNFV-KATSGEADIMISFENGDHGDS 179

Acanthocercus_minutus_MMP20_Acr TLTYRVKKYTSSLSHADVDKAVEMGLKAWSAAAPLNFV-KATSGEADIMISFENGDHGDS 179

Xenagama_zonura_MMP20_Acr TLTYRVKKYTSSLSHADVDKAVEMGLKAWSAAAPLNFL-KATSGEADIMISFENGDHGDS 179

Agama_doriae_MMP20_Acr TLTYRVKKYTSTLSHADVDKAVEMGLKAWSVAAPLNFV-KATSGEADIMISFENGDHGDS 179

Laudakia_wui_Acr TLTYRVKKYTSTLSHADVDKAVEMGLKAWSVAAPLNFV-KATSGEADIMISFENGDHGDS 178

Phrynocephalus_forsythii_Acr TLTYRVNKYTSSLSHADVDKAVEMGLKAWSVAAPLNFV-KTTSGEADIMISFENGDHGDS 178

Phrynocephalus_guinanensis_Acr TLTYRVNKYTSSLSHADVDKAVEMGLKAWSVAAPLNFV-KTTSGEADIMISFENGDHGDS 178

Phrynocephalus_vlangalii_Acr TLTYRVNKYTSSLSHADVDKAVEMGLKAWSVAAPLNFV-KTTSGEADIMISFENGDHGDS 178

Phrynocephalus_putjatai_Acr TLTYRVNKYTSSLSHADVDKAVEMGLKAWSVAAPLNFV-KTTSGEADIMISFENGDHGDS 178

Phrynocephalus_versicolor_Acr TLTYRVKKYTSSLSHADVDKAVEMGLKAWSVAAPLNFV-KTTSGEADIMISFESGDHGDS 177

Intellagama_lesueurii_Acr TLTYRVKKYTSSLSHTDVDKAVEMGLKAWSVATPLNFI-KTTSGEADIMISFENGDHGDS 178

Pogona_vitticeps_Acr TLTYRVKKYTSTLSHAEVDKAVEMGLKAWSVATPLNFI-KTISGEADIMISFENGDHGDS 178

Bradypodion_pumilum_Acr TLTYRVKKYTSSLSHADVDKAVEMGLKAWSVAAPLNFV-KTTSEEADIMISFESGDHGDS 178

Bradypodion_ventrale_Acr TLTYRVKKYTSSLSHADVDKAVEMGLKAWSVAAPLNFV-KTTSEEADIMISFESGDHGDS 178

Chamaeleo_calyptratus_Acr TLTYRVKKYTSSLSHADVDKAVEMGLKAWSVAAPLNFV-KTTSEEADIMISFESGDHGDS 178

Chamaeleo_dilepis_MMP20_Acr TLTYRVKKYTSSLSHADVDKAVEMGLKAWSVAAPLNFV-KTTSEEADIMISFESGDHGDS 178

Trioceros_balebicornutus_MMP20_Acr TLTYRVKKYTSSLSHADVDKAVEMGLKAWSVAAPLNFI-KTTSEEADIMISFESGDHGDS 178

Trioceros_harennae_MMP20_Acr TLTYRVKKYTSSLSHADVDKAVEMGLKAWSVAAPLNFIKKTTSEEADIMISFESGDHGDS 179

Chamaeleo_gracilis_MMP20_Acr TLTYRVKKYTSSLSHADVDKAVEMGLKAWSVAAPLNFV-KTTSEEADIMISFESGDHGDS 178

Furcifer_pardalis_Acr TLTYRVKKYTSSLSHADVDKAVEMGLKAWSVAAPLNFV-KTTSEEADIMISFESGDHGDS 178

Chamaeleo_laevigatus_MMP20_Acr TLTYRVKKYTSSLSHADVDKAVEMGLKAWSVAAPLNFV-KTTSEEADIMISFESGDHGDS 178

Trioceros_affinis_MMP20_Acr TLTYRVKKYTSSLSHADVDKAVEIGLKAWSVAAPLNFI-KTTSEEADIMISFESGDHGDS 178

Anolis_apletophallus_Pleu TLTYRVKKYTPSLSHAEVDKAVEMGLKAWSTAAPLNFV-KTTSGEADIVISFENGDHGDS 177

Anolis_carolinensis_Pleu TLTYRVKKYTPSLSHAEVDKAVEMGLKAWSAAAPLNFV-KTTSGEADIMISFENGDHGDS 177

Anolis_sagrei_ordinatus_Pleu TLTYRVKKYTPSLSHAEVDKAVEMGLKAWSAAAPLNFV-KTTTGEADIMISFENGDHGDS 177

Anolis_tropidonotus_MMP20_Pleur TLTYRVKKYTPSLSHAEVDKAVEMGLKAWSAAAPLNFV-KTASGEADIMISFENGDHGDS 177

Ctenosaura_bakeri_MMP20_Pleur TLTYRVKKYTSSLSHSEVDKAVEMGLKAWSTAAPLNFV-KTTSGEADIMISFENGDHGDS 178

Sceloporus_occidentalis_Pleu TLTYRVKKYTSSLSHSEVDKAVEMGLKAWSTAAPLNFV-KTTSGEADIMISFESGDHGDS 178

Sceloporus_tristichus_Pleu TLTYRVKKYTSSLSHSEVDKAVEMGLKAWSTAAPLNFV-KTTSGEADIMISFESGDHGDS 178

Sceloporus_undulatus_Pleu TLTYRVKKYTSSLSHSEVDKAVEMGLKAWSTAAPLNFV-KTTSGEADIMISFESGDHGDS 178

Urosaurus_nigricaudus_Pleu TLTYRVKKYTSSLSHSEVDKAVEMGLKAWSMAAPLNFV-KTTSGEADIMISFESGDHGDS 178

Phrynosoma_blainvillii_Pleu TLTYRVKKYTTSLSHSEVDKAVEMGLKAWSTAAPLNFV-KTTSGEADIMISFETGDHGDS 178

Phrynosoma_platyrhinos_Pleu TLTYRVKKYTTSLSHSEVDKAVEMGLKAWSTAAPLNFV-KTTSGEADIMISFESGDHGDS 178

Ctenosaura_bakeri_Pleu TLTYRVKKYTSSLSHADVDKAVEMGLRAWSTAAPLNFV-KTTSGEADIMISFESGDHGDS 178

Cyclura_pinguis_Pleu TLTYRVKKYTSSLSHADVDKAVEMGLRAWSIAAPLNFV-KTTSGEADIMISFESGDHGDS 178

Iguana_delicatissima_Pleu TLTYRVKKYTSSLSHADVDKAVEMGLRAWSTAAPLNFI-KTTSGEADIMISFESGDHGDS 178

Gambelia_wislizenii_Pleu TLTYRVKKYTSSLSHGEVDKAVEMGLKAWSTAAPLNFV-KTISGEADIMISFESGDHGDS 178

Laemanctus_serratus_MMP20_Pleur TLTYRVKKYTSSLSHAEVDKAVEMGLQAWSTAAPLNFV-KTTSGEADIMISFESGDHGDS 178

Agama_finchi_MMP20_Acr TLTYRVKKYTSTLSHADVDKAVEMGLKAWSVAAPLNFV-KAASGEADIMISFENGEHGDQ 179

Canis_lupus_dingo_MMP20 TLTYRISKYTSSMSPAEVDKAVEMALQAWGSAVPLSFI-RVNSGEADIMISFETGDHGDS 179

Vulpes_vulpes_MMP20 TLTYRISKYTSSMSPAEVDKAVEMALQAWGSAVPLSFI-RVNSGEADIMISFETGDHGDS 179

Meles_meles_MMP20 ALTYRISKYTPSMPSAEVDKAVEMALRAWSSAVPLSFV-RVDAGEADIMISFETGDHGDS 179

Mustela_putorius_MMP20 SLTYRISKYTPSMPSAEVDKAVEMALQAWSSAVPLSFV-RVNAGEADIMISFETGDHGDS 179

Ursus_arctos_MMP20 TLTYRISKYTPSMAPAEVDKAVEMALQAWSSAVPLGFV-RVNAGEADIMISFETGDHGDS 179

Phacochoerus_africanus_MMP20 TLTYRISKYTPSMTPAEVDKAMEMALQAWSSAVPLSFV-RVNAGEADIMISFETGDHGDS 179

Erinaceus_europaeus_MMP20 TLTYRISKYTSSMTSAEVDKAVEMALQAWSSAVPLNFV-KINSGEADIMISFETGDHGDS 179

Phyllostomus_hastatus_MMP20 TLTYRIAKYTSSMTSADVDKAVEMALQAWSSAVPLNFV-RINSGEADIMISFETGDHGDS 179

Homo_sapiens_MMP20 TLTYRISKYTPSMSSVEVDKAVEMALQAWSSAVPLSFV-RINSGEADIMISFENGDHGDS 179

Saimiri_boliviensis_MMP20 TLTYRISKYTPSMSPAEVDKAMGMALQAWSSAVPLNFV-RINSGEADIMISFETGDHGDS 179

Echinops_telfairi_MMP20 TLTYRISKYTSSMSSAEVDKAVEMALQAWSSAIPLNFV-KVNLGEADIMISFETGDHGDS 179

Suncus_etruscus_MMP20 TLTYRIDKYTSSMTSVEVDKAVEMALQAWSSAVPLSFV-RINSGEADIMVSFETGDHGDS 176

:****: ***.::. :****: :.*:**. * **.*: : ****::***.*:***.

Acanthocercus_cyanogaster_MMP20_Acr YPFDGPRGTLAHAFAPGDGLGGDTHFDNAEKWTMGMNGFNLFTVAAHEFGHALGLAHSTD 239

Acanthocercus_minutus_MMP20_Acr YPFDGPRGTLAHAFAPGDGLGGDTHFDNAEKWTMGMNGFNLFTVAAHEFGHALGLAHSTD 239

Xenagama_zonura_MMP20_Acr YPFDGPRGTLAHAFAPGDGLGGDTHFDNAEKWTMGMNGFNLFTVAAHEFGHALGLAHSTD 239

Agama_doriae_MMP20_Acr YPFDGPRGTLAHAFAPGDGLGGDTHFDNAEKWTMGMNGFNLFTVAAHEFGHALGLAHSTD 239

Laudakia_wui_Acr YPFDGPRGTLAHAFAPGDGLGGDTHFDNAEKWTMGMNGFNLFTVAAHEFGHALGLAHSSD 238
[truncated: 58,840 more chars]
