# Supplementary material for: Reduction of Tooth Replacement Disproportionately Affects the Evolution of Enamel Matrix Proteins
Source: J Mol Evol. 2025 Aug 7;93(4):494–510. doi: 10.1007/s00239-025-10258-4 (PMC12354546; doi:10.1007/s00239-025-10258-4)
Supplement: Supplementary file 5 — Supplementary file5 (DOCX 20 KB) [file 239_2025_10258_MOESM5_ESM.docx]

**Functional distance analyses -** Summary

**Linear Algebra Method**

For each duplicate gene cluster, the evolutionary rate at a given site may differ from that of the ancestral gene. Such sites are referred to as $F_{1}$ sites (functionally divergent), while those that maintain the same evolutionary rate are termed $F_{0}$ sites (functionally conserved). If the classification of a site as $F_{1}$ or $F_{0}$ is independent across duplicate gene clusters, the following relationship applies (as shown in Equation 1 of Wang and Gu, 2001):

$d_{F}\left( i, j \right)= b_{F}\left( i \right)+ b_{F}\left( j \right)$ (1)

Note that $d_{F}\left( i, j \right)=-ln(1-\theta_{ij})$ is a type I functional distance between clusters i and j where $\theta_{ij}$ is a probability that captures the different evolutionary rate between two genes clusters. $b_{F}\left( i \right)=-ln(1-\theta_{i})$ and $b_{F}\left( j \right)=-ln(1-\theta_{j})$ are functional branch lengths for cluster i and j, respectively, and $\theta_{i}$ is the probability of a site having a different rate from the ancestral gene.

In our functional genomics study, multiple type I functional distances were calculated, thus, the relevant functional branch lengths will be derived by the above equation (1).

Suppose a gene, AMEL, has three clusters, pleu, agam and cham. The software DIVERGE (Gu et al., 2013) is able to calculate three type I functional divergences,

$\theta_{pleu,agam}=0.8608$

$\theta_{pleu,cham}=0.9992$

$\theta_{agam,cham}=0.7168$

By the linear algebra calculation, the equations of three functional branch lengths are derived,

$$b_{F}\left( pleu \right)=\frac{d_{F}\left( pleu, cham \right)+d_{F}\left( pleu, agam \right)-d_{F}\left( agam, cham \right)}{2}$$

$$b_{F}\left( agam \right)=\frac{d_{F}\left( agam, cham \right)+d_{F}\left( pleu, agam \right)-d_{F}\left( pleu, cham \right)}{2}$$

$$b_{F}\left( cham \right)=\frac{d_{F}\left( pleu, cham \right)+d_{F}\left( agam, cham \right)-d_{F}\left( pleu, agam \right)}{2}$$

Therefore, three functional branch lengths are

$b_{F}\left( pleu \right)=3.92057$

$b_{F}\left( agam \right)=-1.948727$

$b_{F}\left( cham \right)=3.210329$

**Least Square Method**

Suppose Y = $\left( \begin{matrix} A \\ B \\ C \end{matrix} \right)=\left( \begin{matrix} d_{F}(a,b) \\ d_{F}(a,c) \\ d_{F}(b,c) \end{matrix} \right)=\left( \begin{matrix} -ln(1-\theta_{a,b}) \\ -ln(1-\theta_{a,c}) \\ -ln(1-\theta_{b,c}) \end{matrix} \right)$, Define a design matrix X =$\left( \begin{matrix} 1 & 1 & 0 \\ 1 & 0 & 1 \\ 0 & 1 & 1 \end{matrix} \right)$ and a parameter vector $\beta=\left( \begin{matrix} b_{F}(a) \\ b_{F}(b) \\ b_{F}(c) \end{matrix} \right)=\left( \begin{matrix} -ln(1-\theta_{a}) \\ -ln(1-\theta_{b}) \\ -ln(1-\theta_{c}) \end{matrix} \right)$

According to Equation (1), $d_{F}\left( i, j \right)= b_{F}\left( i \right)+ b_{F}\left( j \right)$

Y = $X\beta$ is equivalent to the above equation 1.

Now, we apply the least square estimate in the following,

$$\hat{\beta}=\left( \begin{matrix} b_{F}(a) \\ b_{F}(b) \\ b_{F}(c) \end{matrix} \right)={(X^{T}X)}^{-1}X^{T}Y=\left( \begin{matrix} \frac{3}{4}\left( A+B \right)-\frac{1}{4}\left( A+C \right)-\frac{1}{4}\left( B+C \right) \\ \frac{3}{4}\left( A+C \right)-\frac{1}{4}\left( A+B \right)-\frac{1}{4}\left( B+C \right) \\ \frac{3}{4}\left( B+C \right)-\frac{1}{4}\left( A+B \right)-\frac{1}{4}\left( A+C \right) \end{matrix} \right)$$

Back to the research data,

$\theta_{pleu,agam}=0.8608$

$\theta_{pleu,cham}=0.9992$

$\theta_{agam,cham}=0.7168$

A = -ln(1-0.8608) = 1.971844

B = -ln(1-0.9992) = 7.130899

C = -ln(1-0.7168) = 1.261602

The solution is

$$\beta=\left( \begin{matrix} b_{F}(a) \\ b_{F}(b) \\ b_{F}(c) \end{matrix} \right)=\left( \begin{matrix} \frac{3}{4}\left( A+B \right)-\frac{1}{4}\left( A+C \right)-\frac{1}{4}\left( B+C \right) \\ \frac{3}{4}\left( A+C \right)-\frac{1}{4}\left( A+B \right)-\frac{1}{4}\left( B+C \right) \\ \frac{3}{4}\left( B+C \right)-\frac{1}{4}\left( A+B \right)-\frac{1}{4}\left( A+C \right) \end{matrix} \right)=\left( \begin{matrix} 3.92057 \\ -1.948727 \\ 3.210329 \end{matrix} \right)$$

Both methods have the same results. If the data extends to four classes, we have to change design matrix X to 4x4 matrix and $\beta$ is a 4x1 vector. The least square estimate is still based on the equation.

$$\hat{\beta}={(X^{T}X)}^{-1}X^{T}Y$$
